# Measurement of abortion safety using community-based surveys: Findings from three countries

**DOI:** 10.1371/journal.pone.0223146

**Published:** 2019-11-07

**Authors:** Suzanne O. Bell, Funmilola OlaOlorun, Mridula Shankar, Danish Ahmad, Georges Guiella, Elizabeth Omoluabi, Anoop Khanna, Andoh Kouakou Hyacinthe, Caroline Moreau

**Affiliations:** 1 Department of Population, Family and Reproductive Health, Johns Hopkins Bloomberg School of Public Health, Baltimore, MD, United States of America; 2 College of Medicine, University of Ibadan, Ibadan, Nigeria; 3 Indian Institute of Health Management Research, Jaipur, India; 4 Institut Supérieur des Sciences de la Population (ISSP), Université de Ouagadougou, Ouagadougou, Burkina Faso; 5 Center for Research, Evaluation Resources and Development, Ile-Ife, Nigeria; 6 Programme National de Santé de la Mère et de l'Enfant (PNSME), Abidjan, Cote d’Ivoire; 7 Gender, Sexual and Reproductive Health, CESP Centre for Research in Epidemiology and Population Health U1018, Inserm, Villejuif, France; University of Oxford, National Perinatal Epidemiology Unit, UNITED KINGDOM

## Abstract

This study aimed to measure abortion safety in Nigeria, Cote d’Ivoire, and Rajasthan, India using population-based abortion data from representative samples of reproductive age women. Interviewers asked women separately about their experience with “pregnancy removal” and “period regulation at a time when you were worried you were pregnant”, and collected details on method(s) and source(s) of abortion. We operationalized safety along two dimensions: 1) whether the method(s) used were non-recommended and put the woman at potentially high risk of abortion related morbidity and mortality (i.e. methods other than surgery and medication abortion drugs); and 2) whether the source(s) used involved a non-clinical (or no) provider(s). We combined source and method information to categorize a woman’s abortion into one of four safety categories. In Nigeria (n = 1,800), 29.1% of abortions involved a recommended method and clinical provider, 5.4% involved a recommended method and non-clinical provider, 2.1% involved a non-recommended method and clinical provider, and 63.4% involved a non-recommended method and non-clinical provider. The corresponding estimates were 32.7%, 3.0%, 1.9%, and 62.4% in Cote d’Ivoire (n = 645) and 39.7%, 25.5%, 3.4%, and 31.4% in Rajasthan (n = 454). Results demonstrate that abortion safety, as measured by abortion related process data, is generally low but varies significantly by legal context. The policy and programmatic strategies employed to improve abortion safety and quality of care are likely to differ for women in different abortion safety categories.

## Introduction

Abortion is among the safest medical procedures when performed according to recommended guidelines [1, 2]. In high-resource countries like the United States, abortion related mortality is less than 1 death per 100,000 procedures, which is 14 times lower than the mortality rate associated with childbirth [3, 4]. Serious complications are similarly rare [5]. Despite the clinical safety of abortions performed under appropriate conditions, unsafe abortion is a leading cause of maternal death in many settings where it is legally restrictive or where provision of abortion services is inadequate [2]. Recent estimates indicate that between 8% and 15% of maternal deaths worldwide are due to unsafe abortion, resulting in tens of thousands of preventable deaths every year [6, 7]. These deaths predominantly occur in the global south in countries where women can only obtain legal, safe abortion under a narrow set of criteria, if any.

Although abortion mortality is still high in many low-resource settings, morbidity and case fatality have fallen in these contexts in recent decades, especially in Latin American countries, due to increased availability of misoprostol [8–10]. Researchers estimate that the unsafe abortion-related case fatality rate has fallen globally from 340 to 220 deaths per 100,000 procedures between 1990 and 2008 [11]. Pharmacies have been instrumental in facilitating women’s access to and use of misoprostol during this period [12]. Formal harm reduction models such as hotlines, internet based telemedicine, and in-person accompaniment models that support women self-sourcing medication abortion are also becoming more common [13–16].

The diffusion of medication abortion outside the formal health system challenges the traditional binary framing of abortion safety. The previous paradigm distinguished safe and unsafe procedures based largely on the legality of abortion in a given setting, in conjunction with a qualitative assessment of the country context [17]. Recent developments favor a more nuanced conceptualization of abortion safety that is in alignment with the World Health Organization (WHO) abortion service delivery recommendations, accounting for procedure type, source, and social context [18]. Investigators operationalized this framework in a recent study providing global and regional estimates of abortion safety, according to three categories: safe if the abortion involved recommended methods *and* providers with recommended levels of training; less safe if only one of the two conditions were met; and least safe when neither of the two conditions were met [19]. While this study is critical in acknowledging the growing proportion of low risk medication abortions performed outside of the healthcare system, the sources of information, based on facility-based data and proxies for safe abortion access, are limited in their ability to directly capture the experiences of women who seek abortion care outside of the formal healthcare system. The paucity of data on such experiences is in large part due to limitations in collecting accurate and unbiased data on women’s abortion experiences. However, our recent success in collecting population-based data on women’s abortions enable us to complement current knowledge of abortion safety.

We aimed to measure abortion safety in Nigeria and Cote d’Ivoire, where abortion is only legal to save a woman’s life, and Rajasthan, India, where women can legally access abortion under a broad range of circumstances. More specifically, we sought to assess the safety of women’s abortions in these contexts using self-reported abortion data from representative samples of reproductive age women. Having more granular data on the details of women’s abortions inside and outside health facilities allowed us to operationalize and further differentiate aspects of the WHO safety categories.

## Methods

We conducted population-based surveys of reproductive age women (15 to 49 years old) in Nigeria, Cote d’Ivoire, and Rajasthan, India as part of a larger multi-country study on family planning and reproductive health. These surveys employed a multi-stage cluster sampling design with probability proportional to size selection of geographical (enumeration) areas; additional details on the sampling strategy are described elsewhere [20]. Resident interviewers conducted face-to-face interviews with respondents using smart phones; English and local language translations of the questionnaires are provided in the supplementary materials (S1–S9 Docs). The Johns Hopkins Bloomberg School of Public Health provided ethical approval, as did the National Health Research Ethics Committee of Nigeria, the Indian Institute of Health Management Research (IIHMR) Institutional Review Board for Protection of Human Subjects in Rajasthan, and the Comite National D' Etique de la Recherche (CNER) in Cote d'Ivoire. In accordance with local IRB requirements, respondents in Nigeria and Cote d’Ivoire provided verbal consent to participate due to concerns about low literacy while respondents in Rajasthan provided written consent. For oral consent, interviewers checked a box on the smart phone to indicate receipt of respondent consent.

Given we were using population-based rather than facility-based data or other data sources that investigators used to produce the recent WHO estimates, we had to adapt the definitions and categories of abortion safety. Additionally, the data one can collect at an individual level is limited by what women can accurately and reliably report. Using these respondent-reported abortion data, we explored how different types of information affected our safety categorization of a given abortion along two dimensions; whether the method(s) used was recommended and whether the source(s) involved a clinical provider. To date, categorization of abortion safety using women’s report of their abortion experiences from a representative, non-clinical sample has not been done.

Interviewers asked women separately about whether they had ever done something to “remove a pregnancy when you were pregnant or worried you were pregnant” or “regulate your period when you were worried you were pregnant”. Interviewers did not probe to determine the lifetime number of terminations; subsequent questions were in relation to the most recent occurrence. We provide further information on this approach in another study [21]. We collected information on the method and source used if a woman reported doing only one thing to terminate the pregnancy. In instances where women reported doing more than one thing, we collected information about the first and last method and source.

In analyses for this study, we identified women as having a history of abortion if they indicated they had had a pregnancy removal or underwent period regulation. We first described the characteristics of the sample of women who reported a history of abortion in each site. We then conducted descriptive analyses of women’s abortion experiences, including number of methods used to terminate the pregnancy, type and source of only or last method used, as well as type and source of first method used when women reported doing multiple things to terminate the pregnancy.

We grouped methods into four categories: 1) surgery; 2) medication abortion (MA) drugs; 3) other pills or pills without sufficient information to categorize in the previous category; and 4) traditional or other methods (like herbal drinks, injections, alcohol, or other traditional remedies). We deemed an abortion as involving a non-recommended method if the woman at any point in the termination used a method other than surgery or MA drugs. This does not assume adherence to method specific clinical guidelines. Women were often unable to provide pill names or sufficient detail for the interviewer to categorize the pill type among available options; 9.6% of abortions in Nigeria, 11.4% of abortions in Cote d’Ivoire, and 8.2% of abortions in Rajasthan involved an unknown or “other” pill type (i.e. excluding MA, anti-malarial, antibiotic, or emergency contraception pills). We categorized these abortions as non-recommended, along with traditional or other methods.

We similarly grouped sources into four categories: 1) public facilities; 2) private facilities (including non-governmental organizations and private doctors); 3) pharmacies or chemist shops; and 4) traditional or other sources (including shops, markets, friends or relativizes, or home). We deemed the termination as involving non-clinical provider(s) if the woman sought any method from a source other than public or private facilities. We did not ask about source for women who reported methods other than surgery or pills, assuming these would have come from informal sources or providers. Similar to the method dimension, we did not make assumptions about abortion-specific provider training in categorizing the source as clinical or not.

Women who did multiple things may have used a non-recommended method or non-clinical source first or last, thus we collected information on both first and last method and source to most accurately categorize each abortion. To explore how abortion pathways (use of one method or multiple methods) altered the safety categorization related to method and source, and the impact of our decision to use information on first and last method and source, we separately measured the proportion of abortions categorized as involving a non-recommended method using: 1) information on the last method used, and; 2) information on first and last method used. We conducted the same analyses with regard to source. We combined source and method information to categorize a woman’s abortion into one of the following four safety categories: 1) recommended method(s) involving clinical source(s); 2) recommended method(s) involving at least one non-clinical source(s); 3) at least one non-recommend method(s) involving clinical source(s); and 4) at least one non-recommended method(s) involving at least one non-clinical source(s).

We present the distribution of abortion safety overall and for those that took place in the last five years. To assess whether the abortion safety distribution was statistically significantly different in the two time periods, we used chi-squared tests. We relied on previously calculated abortion rates from this study [21] for the three settings and the distribution of safety to estimate the rate of abortions involving a non-recommended method and non-clinical source, which represent the most unsafe abortions. As a final sensitivity analysis, we re-categorized all reported surgeries performed by untrained providers as potentially in the most unsafe category.

We accounted for the complex sampling design and clustering using the Taylor linearization method with survey weights to adjust for the probability of selection. All analyses were conducted using Stata version 15.1.

## Results

We present socioeconomic characteristics of women who reported a prior abortion for each country in Table 1. These included 1,810 women in Nigeria, 647 women in Cote d’Ivoire, and 457 women in Rajasthan (Table 1). In Nigeria and Rajasthan, a majority of respondents with a history of abortion were aged 25 to 34, while in Cote d’Ivoire they were similarly likely to be aged 25 to 39. Most women reporting an abortion in Nigeria had a secondary or higher education, while they were most likely to have primary or no education in Cote d’Ivoire and Rajasthan. In all three countries, women who reported an abortion were typically currently married or cohabiting and wealthy. Women in Nigeria and Cote d’Ivoire were more likely to reside in an urban area.

**Table 1:** Respondent characteristics among those who reported an abortion, by country^1^.

		Nigeria		Cote d'Ivoire		Rajasthan	
		%	N	%	N	%	N
Age							
	15–19	4.2	90	6.3	40	1.5	6
	20–24	12.8	253	16.9	107	14.3	74
	25–29	23.3	401	20.7	133	22.3	102
	30–34	22.2	388	18.5	123	23.9	109
	35–39	16.4	302	19.1	120	18.6	84
	40–44	13.6	237	10.9	74	15.1	62
	45–49	7.5	139	7.5	50	4.3	20
Education							
	Never	6.9	173	33.0	219	31.4	156
	Primary	12.3	246	32.3	220	36.7	156
	Secondary	51.1	903	26.5	158	15.2	68
	Higher	29.7	488	8.1	50	16.7	77
Marital status							
	Currently married/cohabiting	68.0	1,261	65.1	416	96.2	439
	Divorced or separated/widowed	6.3	129	6.5	45	2.4	12
	Never married	25.6	418	28.4	186	1.4	6
Religion of household (Nigeria)							
	Catholic	16.9	310	na	na	na	na
	Other Christian	63.2	982	na	na	na	na
	Muslim	18.7	485	na	na	na	na
	Other	1.3	33	na	na	na	na
Religion of household (Cote d'Ivoite)							
	Muslim	na	na	18.2	133	na	na
	Catholic	na	na	29.0	191	na	na
	Evangelical	na	na	22.7	137	na	na
	Other	na	na	20.3	125	na	na
	No religion	na	na	9.8	61	na	na
Religion of household (Rajasthan)							
	Hindu	na	na	na	na	83.4	385
	Muslim	na	na	na	na	14.4	62
	Other	na	na	na	na	2.2	10
Wealth							
	Poorest	11.1	238	14.2	96	13.6	61
	Second poorest	16.8	368	19.1	118	13.9	69
	Middle	19.5	360	14.5	109	21.8	108
	Second wealthiest	24.3	397	23.0	144	22.0	94
	Wealthiest	28.3	447	29.1	180	28.7	125
Residence							
	Rural	28.2	640	36.6	247	52.8	301
	Urban	71.8	1,170	63.4	400	47.2	156
Total		100.0	1,810	100.0	647	100.0	457

^1^Percents are weighted, Ns are unweighted. Some Ns within a characteristic do not add to the total number of respondents due to missingness.

**Fig 1:**
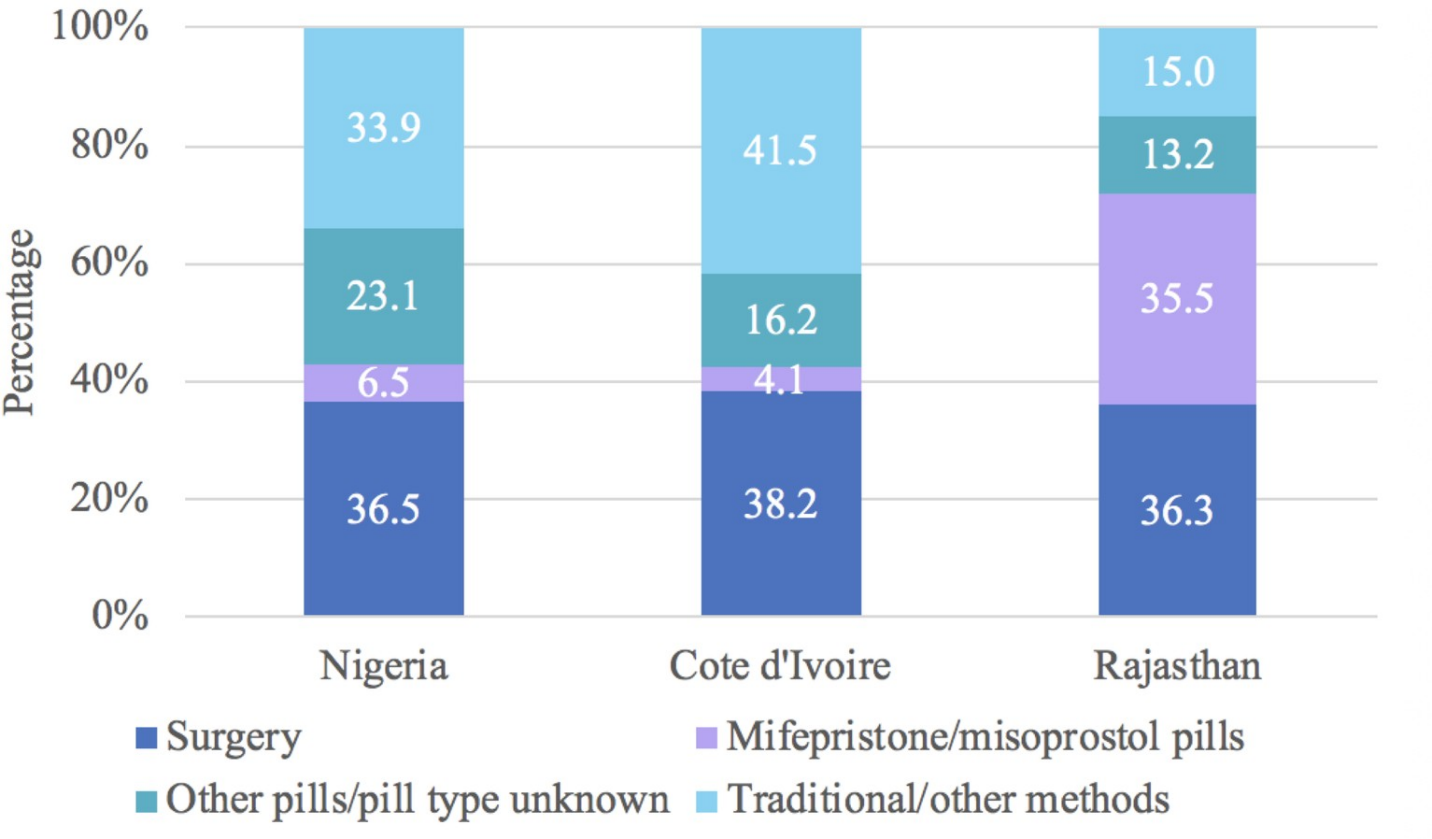
Distribution of last abortion method by country for all women who had an abortion.

**Fig 2:**
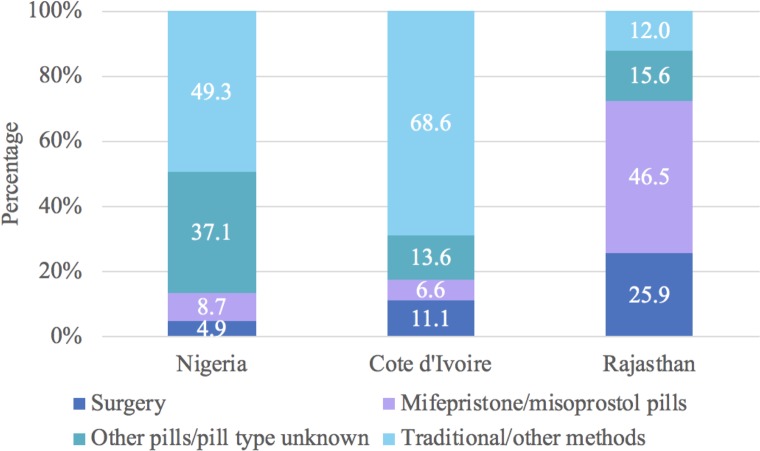
Distribution of first abortion method by country for women who did multiple things to terminate the pregnancy.

**Fig 3:**
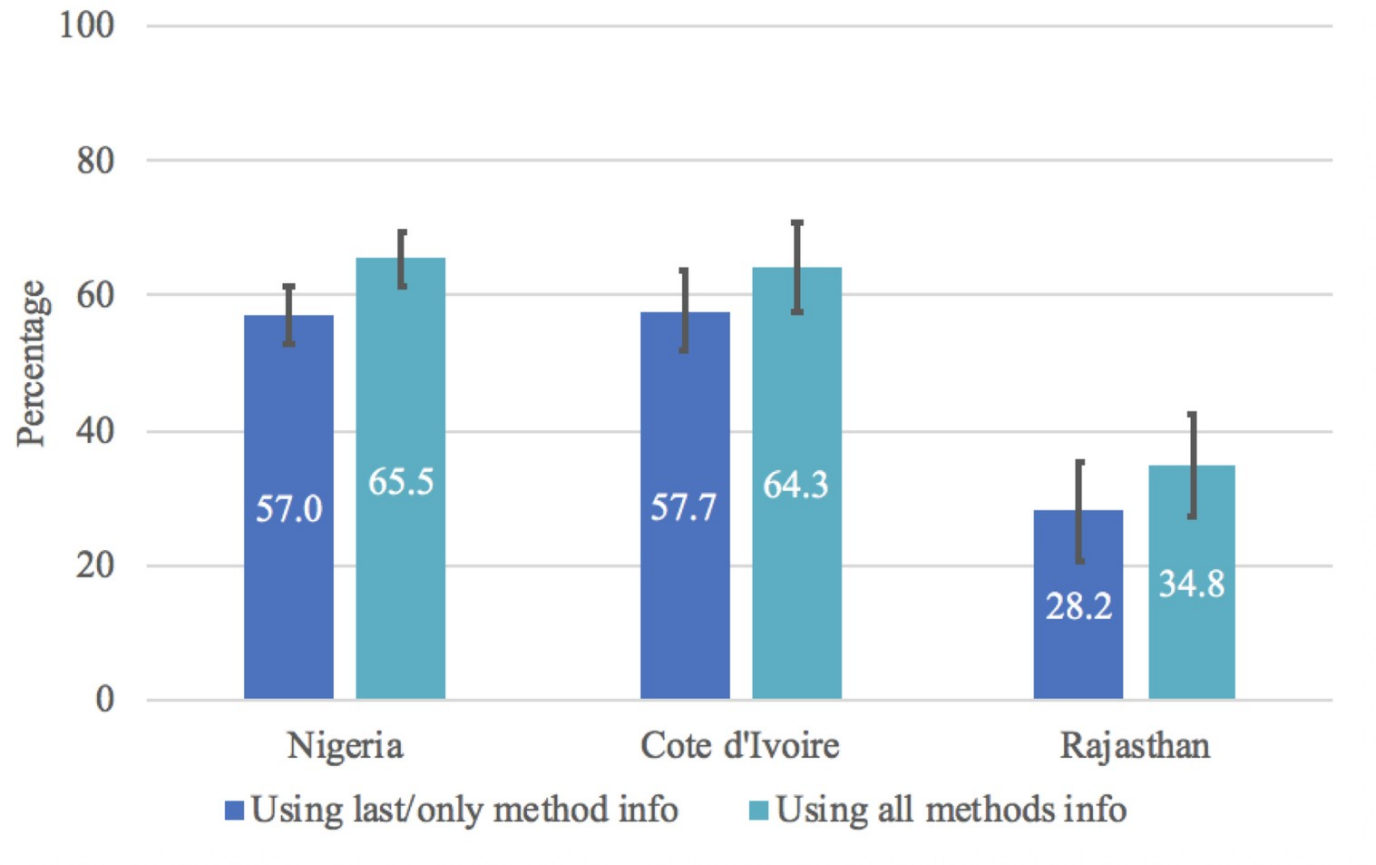
Percentage of abortions categorized as involving a non-recommended method by country.

Similar percentages of women reported doing multiple things to terminate the pregnancy in Nigeria (19.0%), Cote d’Ivoire (19.0%), and Rajasthan (16.5%). We provide the distribution of last or only method used to terminate the pregnancy for all abortions in Fig 1, as well as the first method used for women who did multiple things to terminate the pregnancy in Fig 2. Based on only and last method information, we estimate that 43.0% of women in Nigeria terminated their pregnancy using recommended methods (36.5% surgery and 6.5% MA) (Fig 1). This proportion was 42.3% in Côte d’Ivoire (38.2% surgery and 4.1% MA) and 71.8% in Rajasthan (36.3% surgery and 35.5% MA) (Fig 1). When incorporating information on the first method used for those who did multiple things (Fig 2), the percentage of all women who used only recommended methods decreased by 19.7% in Nigeria, 15.6% in Cote d’Ivoire, and 9.2% in Rajasthan (Fig 3).

**Fig 4:**
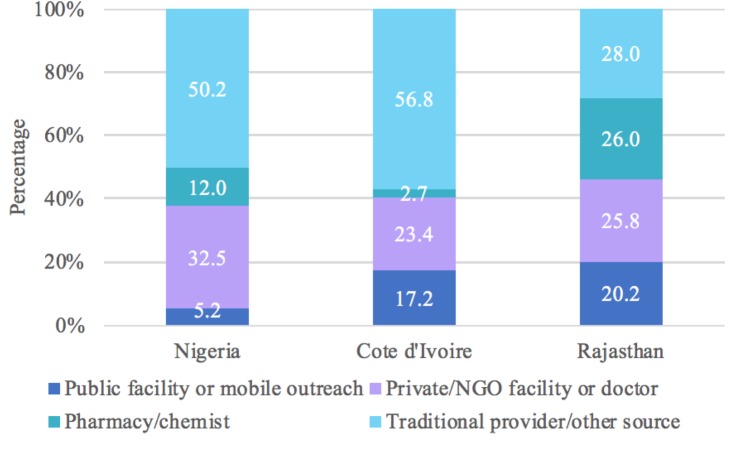
Distribution of source for last abortion method by country for all women who reported an abortion.

**Fig 5:**
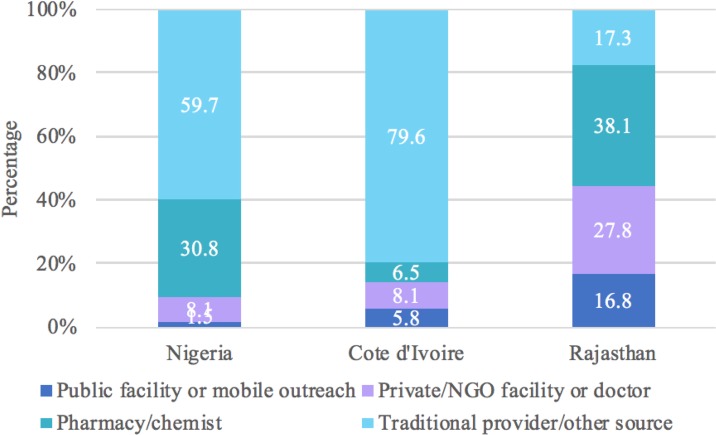
Distribution of source for first abortion method by country for women who did multiple things to terminate the pregnancy.

**Fig 6:**
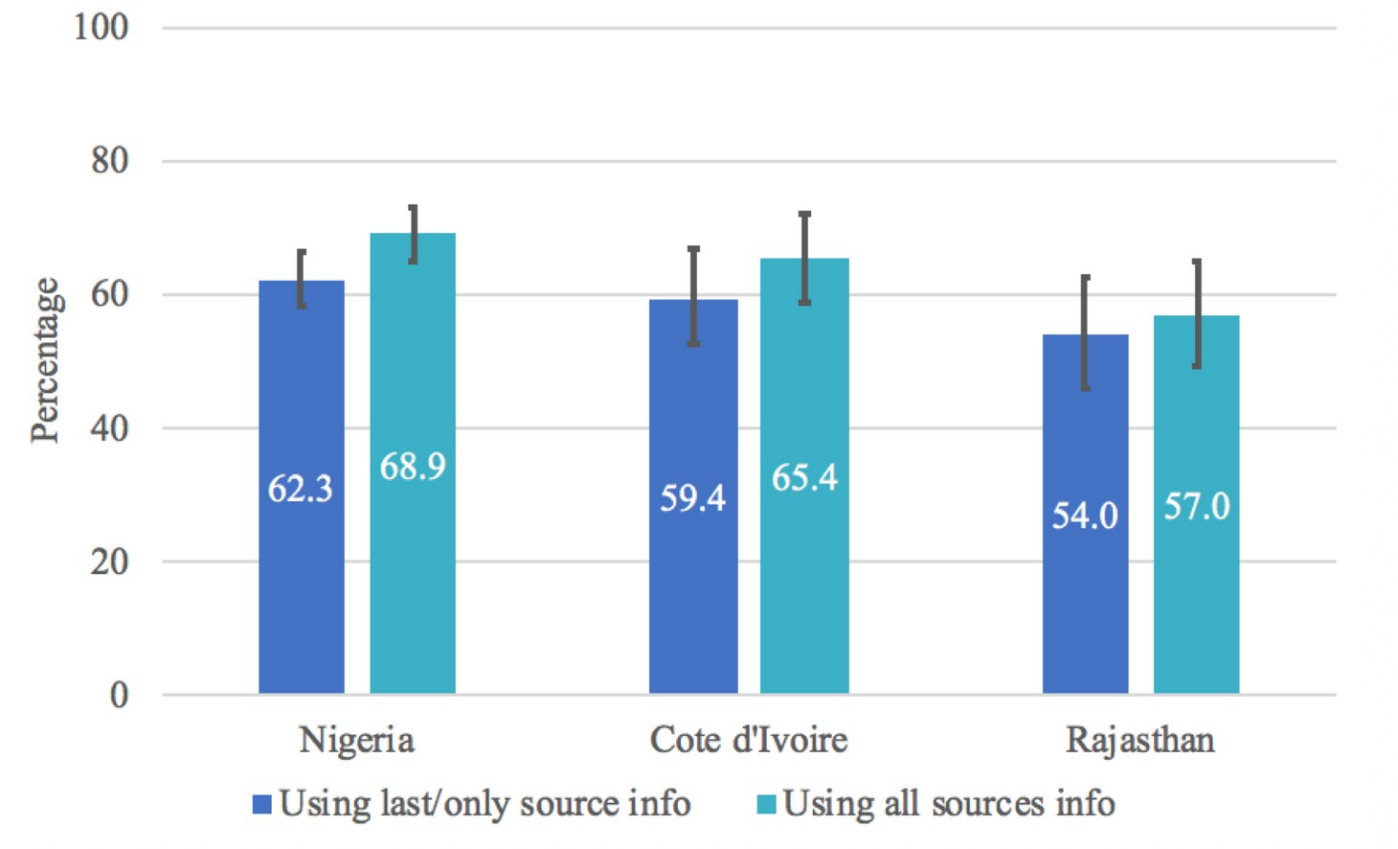
Percentage of abortions categorized as involving a non-clinical provider by country.

The most frequent source used to terminate a pregnancy in Nigeria (50.2%) and Cote d’Ivoire (56.8%) was a traditional provider or “other” source, which included shops and markets (Fig 4). In Rajasthan, women were equally likely to terminate their pregnancy at a private or NGO facility or doctor (25.8%), pharmacy or chemist shop (26.0%), or traditional provider or “other” source (28.0%) (Fig 4). Women who did multiple things to terminate the pregnancy were most likely to seek services for their first method at a traditional provider or “other” source in Nigeria and Cote d’Ivoire (59.7% and 79.6%, respectively) while women in Rajasthan were most likely to go to a pharmacy (38.1%) (Fig 5). Combining information on first and last source, we estimated that 68.9% of Nigerian women relied on a non-clinical provider at some point to terminate their pregnancy, compared to 65.4% in Cote d’Ivoire and 57.0% in Rajasthan (Fig 6).

In Table 2 we present the distribution of abortion safety according to the four safety categories previously described, relying on all method and source information. Abortions in Nigeria and Cote d’Ivoire were more unsafe than in Rajasthan. Nearly two-thirds of abortions in Nigeria and Cote d’Ivoire involved potentially high-risk non-recommended methods from non-clinical sources (63.4% and 62.4%, respectively); this is in contrast to less than a third in Rajasthan (31.7%). Also of note is the much larger proportion of abortions categorized as involving recommended methods but non-clinical providers in Rajasthan compared to the other two countries (26.7% versus 5.4% and 3.0%). Nearly all of the abortions in this category involved MA drugs. If we re-categorized the surgeries provided by non-clinical providers as a non-recommended method, the percentage of women in the most unsafe category would increase by 0.6 percentage points in Nigeria (from 56.3 to 56.9), 0.4 percentage points in Cote d’Ivoire (from 62.4 to 62.8), and 0.2 percentage points in Rajasthan (from 31.4 to 31.6).

**Table 2:** Distribution of respondent and confidante abortions by safety using all information on methods and sources, by country^1^^,^^2^.

		Overall		> 5 years ago		< = 5 years ago	
Nigeria**		%	N	%	N	%	N
Recommended, clinical provider		29.1	471	43.0	305	18.1	166
Recommended, non-clinical provider		5.4	97	3.7	29	6.8	68
Non-recommended, clinical provider		2.1	37	2.8	17	1.5	20
Non-recommended, non-clinical provider		63.4	1,196	50.5	421	73.6	774
Cote d'Ivoire**		%	N	%	N	%	N
Recommended, clinical provider		32.7	198	43.1	127	23.4	71
Recommended, non-clinical provider		3.0	21	3.7	12	2.4	9
Non-recommended, clinical provider		1.9	18	1.1	4	2.7	14
Non-recommended, non-clinical provider		62.4	408	52.2	171	71.5	237
Rajasthan*		%	N	%	N	%	N
Recommended, clinical provider		38.5	163	47.3	101	27.3	62
Recommended, non-clinical provider		26.7	137	20.0	59	35.2	78
Non-recommended, clinical provider		3.1	13	3.3	7	2.9	6
Non-recommended, non-clinical provider		31.7	142	29.4	73	34.6	69

^1^Percents are weighted, Ns are unweighted

^**2**^ Statistical significance assessed using chi-squared tests

* denotes p<0.05, and

** denotes p<0.01

The distribution of abortion safety was significantly different in the last five years, compared to less recent abortions in all three contexts. The changes in Nigeria and Cote d’Ivoire reflected an 23.1 and 19.3 percentage point increase in the non-recommended method/non-clinical provider abortions over time, respectively, while in Rajasthan there was a shift from recommended method/clinical provider abortions to recommended method/non-clinical provider abortions, which increased by 75.4% (Table 2). These changes were driven by a significant increase in the percentage of abortions reported as period regulations in the last five years compared to prior years from 17.2% to 42.3% in Nigeria, 18.9% to 36.7% in Cote d’Ivoire, and 13.9% to 17.4% in Rajasthan. Using overall abortion incidence rates [21] and the safety distribution for the last five years, we estimated the annual incidence rate of the most unsafe abortions (non-recommended method/non-clinical provider) is 36.1 per 1,000 women of reproductive age in Nigeria, 31.0 per 1,000 women in Cote d’Ivoire, and 7.8 per 1,000 women in Rajasthan.

## Discussion

Results demonstrate that abortion safety, as measured by abortion related process measures, is generally low but varies significantly by legal contexts. In Nigeria and Cote d’Ivoire, where abortion is only legal to save a woman’s life, we categorized the majority of abortions as involving non-recommended methods and involving non-clinical providers (63.4% and 62.4%, respectively). This proportion was lower but still substantial in Rajasthan (31.4%), where abortion is broadly legal. These findings corroborate existing literature demonstrating that legal restrictions on abortion primarily impact abortion safety while frequency is less affected [11, 19, 22].

Examining differences in the distribution of abortion safety among all abortions and those in the last five years, we did not find evidence that abortions are becoming safer. This appears to be in part a result of women’s increased reliance on self-sourced pills in an effort to bring back late menses, a non-trivial proportion of which we were unable to identify based on information the respondent provided. To the extent that some of these pills are in fact MA drugs that women were unable to report with sufficient specificity, or if these self-induced abortions are occurring earlier in pregnancy, the abortions in the more recent time period may in fact result in less morbidity and mortality. However, there is also a greater proportion of women using traditional or other methods in Nigeria and Cote d’Ivoire in recent years. Future research should seek to improve ways of ascertaining pill type and collect information on gestational age. Additionally, further work is needed to improve measurement of abortion-related complications and morbidities in order to improve estimation of abortion-related outcomes, which could include construct validation of abortion safety measures. Looking ahead, the lower levels of the most unsafe abortions and women’s greater reliance on self-sourced MA drugs in Rajasthan may be what we observe in other countries once availability and knowledge of these drugs becomes more widespread, regardless of whether the legal status changes.

Comparing our results to the recent WHO safety estimates of safe, less safe, and least safe [19], we generally report higher percentages of least safe abortions (using non-recommended methods/non-clinical provider as a comparison to least safe) and lower estimates of less safe abortion (proxied by our recommended method/non-clinical provider and non-recommended method/clinical provider categories). For example, WHO West Africa and South-central Asia estimates for least safe abortions were 52.1% and 12.9%, respectively, [19] while our estimates ranged between 62.4% and 63.4% in Cote d’Ivoire and Nigeria and 31.4% in Rajasthan. Inversely, WHO West Africa and South-central Asia estimates for less safe abortions were 32.6% and 44.9% [19] while our estimates were lower ranging from 4.9% to 7.5% in Cote d’Ivoire and Nigeria to 28.9% in Rajasthan. The WHO estimates are based strictly on WHO guidelines and recommendations while we defined safety based on the likelihood of experiencing negative sequelae while simultaneously providing insight into how and where women are terminating their pregnancies. As the evidence builds regarding the safety of self-sourced or pharmacy-based MA, distinctions between MA involving clinical providers versus non-clinical providers may not be necessary. Future research could collect data on information received or sought out by women related to medication abortion protocol as we ultimately seek to categorize whether the MA involved evidence-based care. In the meantime, our data allowed us to identify this group of women and their individual characteristics.

Measurement of abortion safety is principally linked to the medical risks of procedures that are non-compliant with WHO recommendations. A broader focus on quality of care, of which safety is an essential part, can help deepen and integrate our understanding of the technical and interpersonal aspects of care, both of which are essential to a client’s health and well-being [23]. However, with the expansion of medical abortion and emerging evidence on the successes of formal and informal service delivery models [13–16], current quality of care frameworks need to be adapted to apply to non-clinical providers and settings. Our framing and assessment of abortion safety using process measures derived from women’s experiences is one step towards quantifying sub-groups of women at the lower end of the risk spectrum for whom data on other measures of quality can be beneficial to improve service delivery.

While these data provide rich details on the specifics of women’s abortions, they are not without limitations. Underreporting of abortion is substantial and may be differential by method and source, which may affect the distribution of our safety estimations. Because there is no external, objective measure of abortion safety in these settings, a true validation of these findings was not possible. Using the confidante methodology, which yields higher estimates of abortion based on respondent reporting of a female confidante’s experience of abortion [21], we found similar or even higher estimates of highly unsafe abortions, i.e. non-recommended method/non-clinical provider (68.6% in Nigeria, 78.4% in Cote d’Ivoire, and 38.8% in Rajasthan) (see Bell et al 2019 for further description of the confidante methodology from which we obtained these safety estimates) [21]. However, we believe the respondent reported abortions are more likely tied to the true abortion safety distribution than the confidante data since respondents would be more likely to know about a friend’s unsafe abortion experience requiring subsequent medical treatment than her experience of a safe, uncomplicated abortion.

Another source of potential bias is misclassification. With regard to surgery, we determined that women could not provide details on the specific procedure conducted. As such, we categorized any surgical abortion as recommended, which would include dilation and curettage despite it being obsolete based on WHO clinical recommendations [1]. Similarly, women could not report on the training of a given provider, thus we relied on information about the source or location of the surgery/medicine and whether providers at that facility type (or non-facility) would be clinicians or not. Additionally, as previously indicated, a substantial number of women were unable to provide sufficient details to categorize the type of pills they used, and among those who reported use of MA drugs, we did not try to determine whether the correct dosage and other information was provided. These limitations would have led to misclassifications when categorizing abortion safety. However, these misclassifications are not likely to be influenced by any specific characteristic of the study population in a systematic manner, hence could be termed as non-differential misclassification.

Separate from misclassification is our inability to provide more nuanced classification. There is a spectrum of safety, especially among non-recommended methods by non-recommended providers. However, we were limited in our ability to more fully capture this in the current study. Some women are doing rather benign things, like drinking hot tea or spicy beverages, while others ingest toxic substances or rely on invasive methods that cause physical injuries. Our current measurement approaches lack the specificity to distinguish between these different levels of risk. While we sought to assess the relative safety of women’s abortions based on process indicators of the quality of care, the actual risk of morbidity and mortality associated with these categories is unknown. There is a need for accurate measurement of complications and morbidity in order to link these process measures with outcomes to ensure the categorization aligns with actual risks of negative sequelae.

Despite these limitations, this study has a number of strengths. The abortion data come from large, population-based studies of abortion safety across diverse settings, including legally restrictive and non-restrictive settings. Given the data come from population- as opposed to facility-based surveys, we were able to capture the substantial and growing population of women having abortions entirely outside the formal healthcare system. As such, our data are more representative of the actual range of abortion experiences in these countries. Our results also provide a more nuanced categorization of abortion safety, demonstrating the intersection between source and method.

## Conclusion

Determining the safety of abortions is critical for related service delivery and policy work. Results demonstrate that abortion safety in these countries is generally low, particularly in Nigeria and Cote d’Ivoire, where abortion is highly legally restrictive. Our results are unique in that they provide details on the specific methods and sources women are most likely to use in these contexts. The diversity of women’s abortion experiences across geographies, many of which occur outside of clinical settings, calls for an expansion of legal abortion service delivery in formal healthcare settings as well as an expansion of MA training in the informal health sector, which for many women represents the first point of contact for abortion care. For women using non-recommended/high-risk methods, regardless of the source, advocates need to disseminate information related to safe methods, like misoprostol. For women using recommended methods from clinical providers, the task of policy makers, advocates, and providers is still not complete as quality of care remains an important issue.

Measuring abortion safety at the individual level provides stakeholders with the characteristics of women whose abortions align with each of these safety categories. As such, these data enable exploration of social inequities in access to and utilization of safe abortion in future analyses, which previous abortion safety estimation approaches have precluded.

## Supporting information

S1 Doc NGR5-Female-Questionnaire-English-v36-aso. (PDF)

S2 Doc NGR5-Female-Questionnaire-Hausa-v36-aso. (PDF)

S3 Doc NGR5-Female-Questionnaire-Igbo-v36-aso. (PDF)

S4 Doc NGR5-Female-Questionnaire-Pidgin-v36-aso. (PDF)

S5 Doc NGR5-Female-Questionnaire-Yoruba-v36-aso. (PDF)

S6 Doc CIR2-Female-Questionnaire-English-v6-jkp. (PDF)

S7 Doc CIR2-Female-Questionnaire-French-v6. (PDF)

S8 Doc RJR4-Female-Questionnaire-English-v10-jkp. (PDF)

S9 Doc RJR4-Female-Questionnaire-Hindi-v10. (PDF)
