# Supplementary material for: Measurement of abortion safety using community-based surveys: Findings from three countries
Source: PLoS One. 2019 Nov 7;14(11):e0223146. doi: 10.1371/journal.pone.0223146 (PMC6837422; doi:10.1371/journal.pone.0223146)
Supplement: S2 Doc — (PDF) [file pone.0223146.s002.pdf]

S2 Doc.

NGR5-Female-Questionnaire-v36-aso.xlsx

001a. Are you in the correct household? EA: [EA entered in the Household Questionnaire] Structure #: [Structure entered in the Household Questionnaire] Household #: [Household entered in the Household Questionnaire]	Ko da yaushe<br>○ E<br>○ Aa
002. Rubuta sunanka/ki a kasa<br>*Shigar da sunan ka/ki*	002 = 0<br>____________________
003b. Rubuta ainahin ranar da lokaci	003 = 0<br>Day: __________<br>Month: __________<br>Year: __________

Wannan bayanin an samoshi ne daga cikin tambayoyin da suka shafi gidan. Duba ki tabbata wanda kike wa tambayoyin shine wanda ya kamata ki tambaya. [ODK zai fito da sunan jaha, LGA, Gundumar kidaya, lambar ginin gida da kuma lambar gidan da aka shigar a cikin tambayoyin da suka shafi gidan] Shin wannan bayanin dai dai ne?	Ko da yaushe
State: ${level1_unlinked}	Jiha<br>____________________
LGA: ${level2_unlinked}	Karamar hukuma<br>____________________
Locality: ${level3_unlinked}	Gari/unguwa<br>____________________
Gundumar kidaya: [EA]	____________________
Lambar gini	____________________
Lambar gida:[#]	____________________
004b. Shin bayanin dai-dai ne?	004 = 0<br>○ E<br>○ Aa

005. 005.Kina kokarin yiwa [sunan wanada zai amsa tambayoyi] tambayoyi, hana ke?<br>*Idan ba dai dai aka rubta ba a zabi“yes” sai a gyara tambaya ta “011.”*<br>*Idan ba mutumin bane akwai zabi biyu:*<br>*(1) A goge canjin da akayi a wannan fom din a bude fom din dayake dai dai.*<br>*Ko*<br>*(2) A nemi mutumin da sunan sa/ta ya fito ayi masa/ta tambayoyin*	Ko da yaushe<br>○ E<br>○ Aa
006. Shin ko wacce zata amsa tambaya tana nan?	Ko da yaushe<br>○ E<br>○ Aa
007. Shin ko kinsan wacce zata amsa tambayoyin?	006 = 1<br>○ Na san ta sosai<br>○ Mun san juna sosai<br>○ Bamu san juna ba sosai<br>○ Ban santa/shi ba
008. Shin ko mai amsa tambayoyin ta taba shiga cikin binciken PMA2020 na baya?	006 = 1<br>○ E<br>○ Aa<br>○ Ban sani ba<br>○ Babu amsa
SANAR YARDA<br>*A samu mace wadda take tsakanin shekaru 15-49 wadda take da alaka da wannan tambayoyin. Dole a tabbatar cewa an kebance yayin gabatar da tambayoyin. A karanta wannan gaisuwar*	(${available} = 'yes') and (not(${unlinked}) or ${proceed_with_unlinked})
Barka da war haka. Suna na	(${available} = 'yes') and (not(${unlinked}) or ${proceed_with_unlinked})

___________________________________ kuma ina aiki tareda Center for Research, Evaluation Resources (CRERD) da hadin gwiwar Jami'ar Bayero da ke Kano. Muna gudanar da wani bincikene game da kulawa da lafiyar mata da kuma haihuwa. Muna fatan zaki samu dama ki amsa tambayoyin wannan bincike. Wannan bincike zai taimaka ta wajen samar da bayanai da gwamnati zatayi amfani dasu domin su kara shiri mai kyau game da ababan kiwon lafiyan jiki. Tambayoyin zasu dauki kamar mintoci 15 zuwa 20 a kammalasu. Za'a sirrinta dukkan wani bayanai da zaki baiyana haka kuma babu zaisan irin amsoshin da zaki fada mini saifa wadda keyin aiki a cikin wannan binciken. Shigarki cikin wannan bincike ta ganin damarkice. Kina iya kin amsa duk wata tambaya da bakiso, haka kuma kina iya ficewa daga cikin wannan bincike a duk lokacin da kikaso yin hakan.

If you have any questions about the study and your right as a research participant, you may ask me now or you may also contact Dr. Elizabeth Omoluobi at Center for Research, Evaluation Resources and Development in Ile-ife, Nigeria at +2348033816486.

Amma kuma muna fata zaki yarda ki amsa tambayoyin a wannan binciken, saboda ra'oyin da zaki baiyana sunada muhimmanci. Kafin na far yimiki tambayoyi, kokina da wata tambaya a gareni game da wannan bincike?

009a. Shin na iya fara yi miki tambayoyi a halin yanzu?	(${available} = 'yes') and (not(${unlinked}) or ${proceed_with_unlinked})<br>○ E<br>○ Aa
010. 010. Sunan maiyin tambayoyin a matsayin shaida. Kin shigar da [Sunan mai yin tambayoyin]	${consent_obtained} and (${your_name_check} = 'no')<br>--

Section 1 – Bayanai akan mai amsa tambayoyin, matsayin auratayya, yanayin iyalin

Yanzu zanso nayi tambayoyi gameda kai/ke da kuma wadansu kayyayyakin da kuke dasu

	${consent_obtained}
101. A wace shekara , da kuma wanne wata aka haife ki? [<br>*Zabi 'Ba'a sani ba' domin watanni, sannan '2020' domin shekara domin nuna ba amsa daga mai amsa tambayoyin*	009a = 1
101. A wace shekara , da kuma wanne wata aka haife ki?<br>*Zabi 'Ba'a sani ba' domin watanni, sannan '2020' domin shekara domin nuna ba amsa daga mai amsa tambayoyin*	009a = 1
Wata	○ Janairu<br>○ Fabrairu<br>○ Maris<br>○ Afrilu<br>○ Mayu<br>○ Yuni<br>○ Yuli<br>○ Agusta<br>○ Satumba<br>○ Oktoba<br>○ Nuwamba<br>○ Disamba<br>○ Ban sani ba
Shekara	Year: ----------------------------------

102. Shekarun ki nawa chikkaku?	009a = 1<br>--
103. Mene ne zurfin ilimin ki? Makarantar firamare, ko sakandare, ko fiye da haka<br>*A shigarda kawai nakarantar boko. Kada a shigarda makarantun addini ko kananan kwas.*	009a = 1<br>○ Ban taba zuwa ba<br>○ Firamare<br>○ Sakandire<br>○ Sama

	Babu amsa
104. Kina da aure yanzu ko kina zaune da wani namiji kamar zaman aure?<br>*A bincika: Idan Sifili, tambaye ta ko an sake ta ne, ko sun rabu, ko kuwa ita gwanrowa ce?*	009a = 1<br>E, yanzu ina da aure<br>E, ina zama da namiji<br>Ba'a tare : Mutuwar aure / rabuwa<br>Ba'a tare : Rasuwar miji<br>Aa, baa taba zama da miji ba gaba daya<br>Babu amsa
105. Kin taba aure ko zama da na miji sau daya ko fiye da haka?	104 ≠ 5<br>Sau daya kawai<br>Fiye da sau daya<br>Babu amsa

	(${marriage_history} = 'more_than_once')
106a. A wane wata da shekara ne kika fara zama da mijin ki na farko ko kuma abokin zaman ki na farko?<br>*Zabi 'Ba'a sani ba' domin watanni, sannan '2020' domin shekara domin nuna ba amsa daga mai amsa tambayoyin*	105=2
Wata	Janairu<br>Fabrairu<br>Maris<br>Afrilu<br>Mayu<br>Yuni<br>Yuli<br>Agusta<br>Satumba<br>Oktoba<br>Nuwamba<br>Disamba<br>Ban sani ba
Shekara	Year: ________________

106b. DUBA: La'akari da abinda aka rubuta a 106a shekarun mai amsa tambayoyin ta 15 ko kasa da haka a lokacin da tayi aure.<br>Shin an shigar da 106a dai-dai?	106a.Shekarar da akayi auren fari kasa da shekara 15<br>E<br>Aa

	(${marriage_history} = 'once') or (${marriage_history} = 'more_than_once')
107a. Yanzu zan so in tambaye ki yaushe ne kika fara zama da mijin ki/ abokin zama na yanzu? A wane wata da shekara ne?<br>*Zabi 'Ba'a sani ba' domin watanni, sannan '2020' domin shekara domin nuna ba amsa daga mai amsa tambayoyin*	105 = 1 or 2
Wata	Janairu<br>Fabrairu<br>Maris<br>Afrilu<br>Mayu<br>Yuni<br>Yuli<br>Agusta<br>Satumba<br>Oktoba<br>Nuwamba<br>Disamba<br>Ban sani ba
Shekara	Year: ________________

107b. DUBA: La'akari da abinda aka rubuta a 107a shekarun mai amsa tambayoyin 15 ko kasa da haka a lokacin da tayi aure wannan aure na yanzu.<br>Shin an shigar da 107a dai-dai?	107a.Shekarar da akayi auren fari tafi 15<br>E<br>Aa

108. Shin Mijin naki, ko shi abokin zaman naki , yana da wasu mata ko kuma yana tare da wasu matan kamar zaman aure?	104 = 1 or 2<br>◯ E<br>◯ Aa<br>◯ Ban sani ba<br>◯ Babu amsa

Sashe na biyu – Haifuwa, ciki & Haihuwa fifita

Yanzu ina da tambayoyi game da duka hahuwar a kika yi a rayuwarki

200. Yanzu kuma inaso nayii miki tambayoyi a game da haife-haife a rayuwarki. Shin ko kin taba haihuwa?	009a = 1<br>◯ E<br>◯ Aa<br>◯ Babu amsa
201. How many times have you given birth?<br>*Shigarda -99 idan ba amsa.*	200 = 1<br>________________

	(${birth_events} > 1)
205. Yaushe kika samu haihuwarki ta farko?<br>*A shigar da kwanan wata. A lissafa kwanan watan ta hanyar amfani da tarihin abubuwan da suka faru idan akwai butkar hakan. A zabi “Ban sani ba idan wata ne ‘2020' idan kuma ba a samu amsa ba daga mai amsa tambayoyin*	201 > 1
Wata	◯ Janairu<br>◯ Fabrairu<br>◯ Maris<br>◯ Afrilu<br>◯ Mayu<br>◯ Yuni<br>◯ Yuli<br>◯ Agusta<br>◯ Satumba<br>◯ Oktoba<br>◯ Nuwamba<br>◯ Disamba<br>◯ Ban sani ba
Shekara	Year: ________________

	(${birth_events} > 0)
206. Yaushe ne kikayi haihuwaki ta karshe?<br>*Zabi ‘Ba'a sani ba' domin watanni, sannan ‘2020' domin shekara domin nuna ba amsa daga mai amsa tambayoyin*	201 > 1
Wata	◯ Janairu<br>◯ Fabrairu<br>◯ Maris<br>◯ Afrilu<br>◯ Mayu<br>◯ Yuni<br>◯ Yuli<br>◯ Agusta<br>◯ Satumba<br>◯ Oktoba<br>◯ Nuwamba<br>◯ Disamba<br>◯ Ban sani ba
Shekara	Year: ________________

210a. A yanzu haka, kina da ciki ne?	${consent_obtained}<br>◯ E<br>◯ Aa<br>◯ Babu tabbas<br>◯ Babu amsa

	${pregnant} = 'yes'

210b. Cikin na wata nawa ne?	210a = 1
Haihuwar baya bayannan {kwanan watan da ta haihu a baya baya}	${recent_birth} != ''
#####<br>*A shigar da adadin watanni. Shigarda -88 idan baa sani ba, -99 idan babu amsa.*	--

209. Yaushe ki ka fara ganin hailar ki ta karshe?<br>*Idan an zabi ranaku, sati, watanni ko shekaru za'a shigar da X a shafi na gaba.*	009a = 1<br>◯ X ranaku da suka wuce<br>◯ X makonni da suka wuce<br>◯ X watanni da suka wuce<br>◯ X shekaru da suka wuce<br>◯ Daina jinin al'ada<br>◯ Kafin haihuwarki ta karshe<br>◯ Bata taba yin haila ba<br>◯ Babu amsa
209a. 209a.Shiga da [Lokacin jinin al'ada]<br>*Shigarda 0 days for today, not 0 weeks/months/years.*	(${menstrual_period} = 'days') or (${menstrual_period} = 'weeks') or (${menstrual_period} = 'month …<br>--

	${ever_birth} = 'yes' or ${pregnant} = 'yes'
213a. Yanzu zan so in yi tambaya game da haihuwan ki me rai na karshennan.	201 > 0 AND 210a ≠ 1 201 > 0 AND 210a ≠ 1 201 > 0 AND 210a ≠ 1
213b. Yanzu zan so in yi tambaya game da ciki na yanzu.	210a = 1
A lokaci da kika sami cikin, ko kin so daukar cikin a lokacin, kin so ki jira sai wani lokaci a gaba, ko kuma baki so ki sake haihuwar wasu yara ba kwata-kwata?	(${birth_events} > 1 and ${pregnant} != 'yes') or (${ever_birth} = 'yes' and ${pregnant} = 'yes')
A lokacin da kika sami cikin, kin ko kin so daukar cikin a lokacin, ko kuma ba kiso ki sake haihuwar wasu yara ba kwata-kwata?	(${birth_events} = 1 and ${pregnant} != 'yes') or ((${ever_birth} = 'no') and (${pregnant} = 'yes'))
#####	◯ A lokacin nan<br>◯ Daga baya<br>◯ Babu gaba daya<br>◯ Babu amsa

Yanzu ina da tambayoyi dangane da abinda zasu zo nan gaba	${consent_obtained}
211a. Ko kina son sake haihuwar da/ya ko kuma ba ki son ki sake haihuwar wasu yayan? yarinya?	210a ≠ 1<br>◯ Ina so in samu da/'ya<br>◯ Bana bukatar 'ya'ya<br>◯ Tace bazata iya daukar ciki ba<br>◯ Ban yanke hukunci ba/ Ban sani ba<br>◯ Babu amsa
211a. Ko kina son sake haihuwar da/ya ko kuma ba ki son ki sake haihuwar wasu karin yayan?	210a ≠ 1<br>◯ Ina da wani da/'ya<br>◯ Babu wani<br>◯ Tace bazata iya daukar ciki ba<br>◯ Ban yanke hukunci ba/ Ban sani ba<br>◯ Babu amsa
211b. Bayan kin yi haihuwar da kike tsammani a halin yanzu, ko kina son ki sake haihuwar da/ya ko kuma baza ki so ki sake haihuwar wasu yaya ba?	210a = 1<br>◯ Ina da wani da/'ya<br>◯ Babu wani<br>◯ Tace bazata iya daukar ciki ba<br>◯ Ban yanke hukunci ba/ Ban sani ba<br>◯ Babu amsa
212a. Shekaru ko wata nawa ne zaki so daga yanzu kafin ki sake haihuwar da/ya?<br>*Idan aka zabi watanni ko shekaru zaa rubuta X a shafi na gaba.*<br>*A zabi "Shekaru" idan an wuce wata 36*<br>*A tabbatar an shigar da watannin/shekarun dai dai.*	211a = 1<br>◯ X watanni<br>◯ X shekaru da suka wuce<br>◯ Kwanannan/yanzu<br>◯ Tace bazata iya daukar ciki ba

	○ Wani abu daban<br>○ Ban sani ba<br>○ Babu amsa
212b. Bayan kin yi haihuwar da kike tsammani a halin yanzu, shekaru ko wata nawa ne zaki so daga yanzu kafin ki sake yin wata haihuwar?<br>*Idan aka zabi watanni ko shekaru zaa rubuta X a shafi na gaba.*<br>*A zabi "Shekaru" idan an wuce wata 36*<br>*A tabbatar an shigar da watannin/shekarun dai dai.*	211b = 1<br>○ X watanni<br>○ X shekaru da suka wuce<br>○ Kwanannan/yanzu<br>○ Tace bazata iya daukar ciki ba<br>○ Wani abu daban<br>○ Ban sani ba<br>○ Babu amsa
212c. 212c.Shigar da yawan [Watanni ko shekarun] da zaki/ka iya jira	${wait_birth_none} = 'months' or ${wait_birth_some} = 'months' or ${wait_birth_pregnant} = 'mont …<br>--

Section 3 – Hanyar tsarin iyali

Yanzu zamuyi Magana akan tsarin iyali-hanyoyin da ma'aurata zasu iya amfani das u domin su jinkirta ko kuma hana daukar ciki. Hoton wasu daga cikin hanyoyin tsaarin iyalin zai fito akan skirin din wayar. Idan mai amsa tambayoyin tace bata taba jin labarin hanyoyin ba, ko kuma taki bada amsa, karanta mata da karfi sannan kuma ki nuna mata hotunan idan akwai

301a. Shin ko kin taba jin labarin aikin da ake yiwa mata don kar su sake samun haihuwa?<br>BINCIKA: Akan yiwa mata tiyata don hana samun haihuwa.	009a = 1<br>○ E<br>○ Aa<br>○ Babu amsa
301b. Shin ko kin taba jin labarin aikin da ake yiwa maza don kar su sake samun haihuwa?<br>BINCIKA: Akan yiwa maza tiyata don hana samun haihuwa.	009a = 1<br>○ E<br>○ Aa<br>○ Babu amsa
301c. Shin ko kin taba jin wata hanya da ake bada tazarar haihuwa ta sanya ashanar fata da likita ko jami'an kiwon lafiya kan sakawa mata don hanasu daukan ciki tsawon shekara guda ko fiye da haka? BINCIKA: Akan iya sakawa mata ashanar fata guda daya ko biyu a dantsen ta wadda zai iya hanata daukar ciki na tsawon shekara daya ko fiye<br>[implant_150x300.png]	009a = 1<br>○ E<br>○ Aa<br>○ Babu amsa
301d. Shin ko kin taba jin wata hanya da ake bada tazarar haihuwa ta hanyar saka roba a mahaifa?<br>BINKCIKA: Ana sakawa mata roba a bakin mahaifarsu wanda likita ko jami'in lafiya ke yi<br>[IUD_150x300.png]	009a = 1<br>○ E<br>○ Aa<br>○ Babu amsa
301e. Shin ko kin taba jin labarin allura da likta ko jami'in kiwon lafiya kan yiwa mata don hanasu samun ciki na wata daya ko fiye da haka?<br>BINCIKA: Likita ko jami'in lafiya kan yiwa mata allura don hana daukar ciki<br>[sayana_depo_150x300.jpg]	009a = 1<br>○ E<br>○ Aa<br>○ Babu amsa
301f. Shin ko kin taba jin labari kwayoyin magani da mata kan iya sha kullum don hana daukar ciki?<br>BINCIKA: Mata na iya shan kwayoyi a kullum domin hana samun ciki<br>[pill_150x300.png]	009a = 1<br>○ E<br>○ Aa<br>○ Babu amsa
301g. Shin ko kin taba jin hanyar tsarin iyali na gaggawa?	009a = 1<br>○ E<br>○ Aa<br>○ Babu amsa
301h. Shin ko kin tabajin labarin kwaroron roba da akan sa kafin jima'i?<br>BINCIKA: Maza kan iya saka wani kwaroro na roba kafin jima'i<br>[male_condom_150x300.png]	009a = 1<br>○ E<br>○ Aa<br>○ Babu amsa
	009a = 1

301i. Shin ko kin taba jin labarin kwaroron roba na mata da akan sa kafin jima'i?<br>BINCIKA: Mata kan iya saka wani kwaroro na roba kafin jima'i<br>[female_condom_150x300.png]	E<br>Aa<br>Babu amsa
301j. Shin ko kin taba jin labarin wani fai-fai da ake sakawa a bakin mahaifa don hana daukar ciki?<br>BINCIKA: Mata kan iya saka wani fai-fai a bakin mahaifa kafin jima'i<br>[diaphragm_150x300.png]	009a = 1<br>E<br>Aa<br>Babu amsa
301k. Shin ko kin taba jin labarin wani magani magani mai kumfa ko maiko da mata kan iya sakawa a farjinsu kafin suyi jima'i?<br>BINCIKA: Mata kansa abu mai kama da mai ko kumfa a farjinsu kafin jima'I don hana daukar ciki<br>[spermicide_150x300.png]	009a = 1<br>E<br>Aa<br>Babu amsa
301l. Shin ko kin taba jin labarin wasu dabaru na kirga kwanakin daukar ciki ta hanyar amfani da wani carbi mai kala don gane ranar da zata dau ciki?<br>BINCIKA: Mata kanyi amfani da wani abu mai zagaye kamar 'ya'yan carbi mai kala-kala don gane ranakun da zata iya daukar ciki. A ranakun data gano zata iya samun ciki ita da mijinta/ abokin zamanta sukan iya amfani da kwaroron roba kafin jima'i<br>[SDM-beads_only.png]	009a = 1<br>E<br>Aa<br>Babu amsa
301m. Shin ko kin taba jin labarin mata kanyi amfani da hanyar shayar da Mama akai-akai har na tsawon wata shida don yin hakan nasa rashin ganin jinin hailarsu?	009a = 1<br>E<br>Aa<br>Babu amsa
301n. Shin kin taba jin labarin mace na iya gujewa yin jima'I dai dai lokacin da takan iya samu ko daukar ciki?<br>BINCKIA: Mace na iya kauracewa jima'I a ranakun data gano zata iya daukar ciki	009a = 1<br>E<br>Aa<br>Babu amsa
301o. Shin ko kin taba ji labarin namiji na iya zare azzakarinsa yayin jimai a dai-dai lokacin dayaji zaiyi zuwan kai?<br>BINCIKA: Maza kan lura sosai don zare azzakari kafin zuwan kai	009a = 1<br>E<br>Aa<br>Babu amsa
301p. Shin ko kin tabajin labarin wata dabara daban ko ta gargajiya wadda mata da maza ke amfani da ita don gujewa daukar ciki?	009a = 1<br>E<br>Aa<br>Babu amsa
302a. Shin a yanzu haka ke ko mijinki kuna anfani da wani abu ko yin wata dabara don jinkirta daukan ciki ko kuma gujewa daukan ciki?	210a ≠ 1 AND 009a = 1 210210a ≠ 1 AND 009a = 1 210a ≠ 1 AND 009a = 1 ≠ 1 AND 009a = 1 210a ≠ 1 AND 009a = 1<br>E<br>Aa<br>Babu amsa

	${current_user} = 'yes'
302b. Wacce hanya ko hanyoyin bada tazarar haihuwa kike amfani da ita/su?<br>BINCIKA: Akwai wani abu kuma?<br>*A zabi duk hanyoyin da aka fada. A JE KASA dan ganin duka zabin.*	302a = 1<br>Yiwa mata aiki a mahaifa<br>Yi wa namiji aiki<br>ashanar fata<br>Robar mahaifa<br>Allurar jinkirta ko hana daukar ciki<br>Kwayoyin hana daukar ciki<br>Dabarar hana daukar ciki ta gaggawa<br>Kwaroron roba na maza<br>Kwaroron roba na mata<br>fai-fai da ake sakawa a bakin mahaifa don hana daukar ciki<br>Kumfa/man shafawa dan jinkirta ko dakatar da daukar ciki<br>Carbin kirga ranakun al'ada<br>LAM<br>Kinyin jima'i lokacin al'ada

	☐ Yin inzali a waje<br>☐ Wadansu hanyoyin na al'ada<br>☐ Babu amsa

LISSAFA: HANYAR TSARIN IYALIN DA AKE AMFANI DA ITA WANNAN BAZAI FITO A SIKIRIN BA<br>*ODK zai zabo hanyar tsarin iyalin mafi inganci da mai amsa tambayoyin take amfani da ita a yanzu*	302a=1 AND 302b ≠-99<br>○ Yiwa mata aiki a mahaifa<br>○ Yi wa namiji aiki<br>○ ashanar fata<br>○ Robar mahaifa<br>○ Allurar jinkirta ko hana daukar ciki<br>○ Kwayoyin hana daukar ciki<br>○ Dabarar hana daukar ciki ta gaggawa<br>○ Kwaroron roba na maza<br>○ Kwaroron roba na mata<br>○ fai-fai da ake sakawa a bakin mahaifa don hana daukar ciki<br>○ Kumfa/man shafawa dan jinkirta ko dakatar da daukar ciki<br>○ Carbin kirga ranakun al'ada<br>○ LAM<br>○ Kinyin jima'i lokacin al'ada<br>○ Yin inzali a waje<br>○ Wadansu hanyoyin na al'ada<br>○ Babu amsa
LCL_301. BINCIKA: Shin anyi allurar ta hanyar sirinji ko karamin allurar?<br>*A nuna wa mai bada amsoshin hoton*<br>[sayana_depo_150x300.jpg]	CALC CM = 5<br>○ Sirinji<br>○ karamar allura (Sayana Press)<br>○ Babu amsa
302c. 302c.Shin mijinki/abokin zaman ki yasan kina amfani da [HANYAR TSARIN IYALIN DA KIKE AMFANI DA ITA]	302a = 1<br>○ E<br>○ Aa<br>○ Babu amsa
302c. Shin mijinki/abokin zaman ki yasan kina amfani da family planning?	302a = -99<br>○ E<br>○ Aa<br>○ Babu amsa
303. Ko jami'in kiwon lafiyar akan tazarar haihuwa ya fada miki ko maigidan ki cewa baza ki sake samun ciki ba?	302= Dandaka na maza da na mata<br>○ E<br>○ Aa<br>○ Babu amsa
305a. Kin fadi cewa ba kya amfani da wata hanyar bada tazarar haihuwa. Ko kina tsamani za ki yi amfani da shi anan gaba domin gujewan daukan ciki?	302a ≠1 AND 210a ≠1<br>○ E<br>○ Aa<br>○ Babu amsa
305b. Ko kina tsamani za ki yi amfani da shi anan gaba domin gujewan daukan ciki?	302a ≠1 AND 201a = 1<br>○ E<br>○ Aa<br>○ Babu amsa
306a. A cikin watani 12 da suka wuce, ko kin yi wani abu, ko kuwa amfani da wata hanyar hana daukan ciki?	302a ≠1<br>○ E<br>○ Aa<br>○ Babu amsa

	${recent_user} = 'yes'
306b. Wacce hanya ce kika yi amfani da ita a kwanakin da suka wuce ba da dadewa ba? BINCIKA: Akwai wani abu kuma?<br>*A zabi dabarar da tafi aiki (wadda tafi aiki a cikin lis din). A je kasa domin zabi*	306a = 1<br>○ ashanar fata<br>○ Robar mahaifa<br>○ Allurar jinkirta ko hana daukar ciki<br>○ Kwayoyin hana daukar ciki<br>○ Dabarar hana daukar ciki ta gaggawa<br>○ Kwaroron roba na maza

	○ Kwaroron roba na mata<br>○ fai-fai da ake sakawa a bakin mahaifa don hana daukar ciki<br>○ Kumfa/man shafawa dan jinkirta ko dakatar da daukar ciki<br>○ Carbin kirga ranakun al'ada<br>○ LAM<br>○ Kinyin jima'i lokacin al'ada<br>○ Yin inzali a waje<br>○ Wadansu hanyoyin na al'ada<br>○ Babu amsa

LCL_302. BINCIKA: Shin anyi allurar ta hanyarsyringe ko small needle?<br>*A nuna wa mai bada amsoshin hoton*<br>[sayana_depo_150x300.jpg]	306b = 5<br>○ Sirinji<br>○ karamar allura (Sayana Press)<br>○ Babu amsa
307. 307.Kafin ki fari amfani da [HANYAR TSARIN IYALIN DA KIKE AMFANI DA ITA A YANZU/HANYAR DA KIKA YI AMFANI DA ITA DAGA BAYA BAYANNAN], shin kin tattauna da mijinki/abokin zaman ki domin yanke hukunci akan jinkirtawa ko kuma hana daukar ciki?	302a = 1 OR 306a = 1<br>○ E<br>○ Aa<br>○ Ban sani ba<br>○ Babu amsa
308. Shin kina ganin cewar yin amfani da hanyar tsarin iyali shawarar k ice ke kadai, ko kuma shawarar mijinki ce shi kadai ko kuma ku biyu ne kuka yanke shawara a tare?	302a = 1<br>○ Mai amsa tambayoyin kawai<br>○ Miji ne kawai/Abokin zama<br>○ Yanke hukunci a tare<br>○ Wani abu daban<br>○ Babu amsa
TCI_302. Bayan ke da mijinki/ abokin zamanki wanene kuma yake da ruwa da tsaki wajen yanke shawarar kiyi amfani da hanyoyin dakatar da ko hana daukar ciki?<br>PROBE: Anybody else?<br>*Kar a karanta afili. A zabi duk wadda aka fada*	302a = 1 OR 306a = 1<br>☐ Mahafiya<br>☐ Suruka mace<br>☐ Yan uwa mata<br>☐ yan uwan miji mata<br>☐ Kaka mace<br>☐ Abokanai<br>☐ maaikacin lafiya<br>☐ Shugaban al'umma<br>☐ Shugaban addini<br>☐ Anti<br>☐ Wasu 'yan uwan<br>☐ Wani abu daban<br>☐ Babu ko daya<br>☐ Babu amsa
TCI_302x. A cikin watanni goma sha biyu da suka wuce shin ko kin ja hankalin wani aboki / yan uwa akan wata hanyar dakatar da ko hana daukar ciki?	302a = 1 OR 306a = 1<br>○ E<br>○ Aa<br>○ Ban sani ba<br>○ Babu amsa
308a. A lokaci na baya bayannan da kika karbi [CURREN METHOD /MOST RECENT METHOD], nawa kika kashe daga aljihunki, wannan ya hada da duk abin da kika biya domin ayyuka ko magungunan tsarin iyali da kuma kudin mota.<br>*A shigara da dukkanni kudin a Naira. Zai iya yiwuwa sipili ya zama amsa. Shigar da -88 idan mai amsa tambayoyin bai sani ba, -99 idan kuma bai bada amsa ba.*	302a = 1 OR 306a = 1<br>--

	${current_user} = 'yes'
309a. 309a.Tun daga wacce shekara da wata kike amfani da hanyar da kike amfani da ita yanzu kuma baki tsayaba?<br>*A kirga baya ko zaa tuna da wani abu idan da bukatar haka.*	302a = 1
Haihuwa ta baya bayannan	${recent_birth} != ''
Auren da ke da shi yanzu [mm_yyyy]	${husband_cohabit_start_recent} != ''
Wata	○ Janairu

	Fabrairu<br>Maris<br>Afrilu<br>Mayu<br>Yuni<br>Yuli<br>Agusta<br>Satumba<br>Oktoba<br>Nuwamba<br>Disamba<br>Ban sani ba
Shekara	Year: ______________

	${recent_user} = 'yes'
309b. Yaushe kika daina amfani da ${current_recent_label}?<br>*A shigar da kwanan wata. A lissafa kwanan watan ta hanyar amfani da tarihin abubuwan da suka faru idan akwai butkar hakan. A zabi “Ban sani ba idan wata ne ‘2020' idan kuma ba a samu amsa ba daga mai amsa tambayoyin*	306a = 1
Wata	Janairu<br>Fabrairu<br>Maris<br>Afrilu<br>Mayu<br>Yuni<br>Yuli<br>Agusta<br>Satumba<br>Oktoba<br>Nuwamba<br>Disamba<br>Ban sani ba
Shekara	Year: ______________

	${recent_user} = 'yes'
309c. 309c.Tun daga wacce shekara da wata kike amfani da hanyar da kike amfani da ita yanzu kuma baki tsayaba?<br>*Calculate backwards from memorable events if needed.*<br>*Select 'Do not know' for month and '2020' for year to indicate No Response.*	306a = 1
Haihuwa ta baya bayannan	${recent_birth} != ''
Auren da ke da shi yanzu [mm_yyyy]	${husband_cohabit_start_recent} != ''
Wata	Janairu<br>Fabrairu<br>Maris<br>Afrilu<br>Mayu<br>Yuni<br>Yuli<br>Agusta<br>Satumba<br>Oktoba<br>Nuwamba<br>Disamba<br>Ban sani ba
Shekara	Year: ______________

309d. 309d.Domin na tabbata na dauka dai dai kin fara amfani da () ba tsayawa daga watan ()	306a = 1<br>E<br>Aa
A KOMA BAYA A SAKE TAMBAYA GAME DA LOKACI NA KARSHE DA AKACI GABA DA AMFANI DA HANYAR	309d = 0

Abubwan da zaa iya tambaya: - Yaushe ne lokaci na karshe da akayi amfani da hanyar [METHOD]? - Tsawon wanne lokaci aka dauka ana amfani da [METHOD] ba tare da tsayawaba	
310. Me yasa kika daina amfani da ${current_recent_label}?	306a = 1<br>☐ wanda ba safai ba ne jima'i/miji tafi<br>☐ An samu ciki bayan an fara amfani da hanyar<br>☐ Ina bukatar samun ciki<br>☐ Mijina ko abokin zamana bai amince ba<br>☐ Ana bukatar hanyar datafi aiki<br>☐ Babu wata hanyar jinkirta ko hana daukar ciki a halin yanzu<br>☐ Damuwa akan lafiya<br>☐ Tsoron matsala<br>☐ Rashin hanya mai kyau/wurin yayi nisa<br>☐ Akwai tsada sosai<br>☐ Wahalar aiki<br>☐ Illa mai tsanani<br>☐ Wahala yayin da aka dauki ciki/hana daukar ciki gaba daya<br>☐ Yanayin jikina na canjawa<br>☐ Wani abu daban<br>☐ Ban sani ba<br>☐ Babu amsa

	${current_or_recent_user} and (${current_recent_method} != 'LAM') and (${current_recent_method} != …
311a. 311a.kin fara amfani da () a ina ke ko abokin zamanki ku ka samu wannan hanyar?<br>*A je kasa domin ganin dukkan zabin.*	(CALC_CM ≠ 14, 30, 31, 39, -99) OR (306b ≠ 14, 30, 31, 39, -99)<br>○ Asibitin gwamnati<br>○ Cibiyar lafiya ta gwamnati<br>○ Karamin asibitin samar da dabarun dakatar da ko hana daukar ciki<br>○ Asibitin tafi da gidan ka (na gwamnati)<br>○ TBA/Fieldworker (public)<br>○ Asibitin kudi/kilinik<br>○ Wajen bayarda magani<br>○ Kemis / Babban shagon magani<br>○ Likita ko nurse masu zaman kansu<br>○ Asibitin tafi da gidan ka (me zaman kasan)<br>○ Unguwar zoma (masu zaman kan su)<br>○ Shago<br>○ Kungiyar addini/Coci<br>○ Aboki/dan uwa ko yar uwa<br>○ NGO<br>○ Kasuwa/ 'Yan talla<br>○ Wani abu daban<br>○ Ban sani ba<br>○ Babu amsa

312a. 312a.kin karbi () ko maaikacin lafiyar da ya baki wannan hanyar yayi miki dangane da irin matsalar da zaki iya fuskanta bayan amfani da wannan hanyar?	311a ≠ .<br>○ E<br>○ Aa<br>○ Babu amsa
	312a = 1

312b. Shin an gaya miki abinda za ki yi in har kin fuskanci wata illa ko matsala?	○ E<br>○ Aa<br>○ Babu amsa
313. Shin a wannan lokacin, an fada miki wasu dabaru na tsarin iyali daban da ${current_recent_label} da za ki iya yin amfani da su?	311a ≠ . OR 311b ≠ .<br>○ E<br>○ Aa<br>○ Ban sani ba<br>○ Babu amsa
314a. A ziyarar da kikayi domin karbo dabarun samun tazarar haihuwa, ko kin samu hanyoyin da kike bukata don jinkirta daukan ciki ko hana samun ciki?	311a ≠ .<br>○ E<br>○ Aa<br>○ Babu amsa
314c. Me yasa baki samu hanyar da kike so ba?	314a = 0<br>○ Babu dabarar dakatar da ko hana daukar ciki a halin yanzu<br>○ Babu wannan dabarar dakatar da ko hana daukar ciki gaba daya<br>○ Mai bada dabarun bashi da kwarewar bayar da wannan hanya<br>○ Mai bada dabarun ya/ta bada wata hanyar ta daban<br>○ Bata cancanci wannan tsarin ba<br>○ Na yanke cewa bazan zabi kowace dabara ba<br>○ Yayi tsada da yawa<br>○ Wani abu daban<br>○ Babu amsa
315a. Wanene ya yanke hukunci na karshe gameda hanyar jinkirta ko hana daukar ciki a yayin da kika kai wannan ziyarar?	311a ≠ .<br>○ ka kadai<br>○ Mai bada dabarun<br>○ Abokin zama<br>○ Ke da mai lura da jami'in lafiyar<br>○ ku, kuma abokin tarayya<br>○ Wani abu daban<br>○ Ban sani ba<br>○ Babu amsa
315b. Wanene ya yanke hukunci na karshe gameda hanyar jinkirta ko hana daukar ciki ta yin amfani gujewa yin jima'I dai dai lokacin da takan iya samu ko daukar ciki?	311b ≠ .<br>○ ka kadai<br>○ Mai bada dabarun<br>○ Abokin zama<br>○ Ke da mai lura da jami'in lafiyar<br>○ ku, kuma abokin tarayya<br>○ Wani abu daban<br>○ Ban sani ba<br>○ Babu amsa
315b. Wanene ya yanke hukunci na karshe gameda hanyar jinkirta ko hana daukar ciki ta yin amfani da hanyar shayarwa akai-akai?	311b ≠ .<br>○ ka kadai<br>○ Mai bada dabarun<br>○ Abokin zama<br>○ Ke da mai lura da jami'in lafiyar<br>○ ku, kuma abokin tarayya<br>○ Wani abu daban<br>○ Ban sani ba<br>○ Babu amsa
316. 316.Shin zaki dawo wajen wannan mai bada tsarin iyalin?	311a ≠ 35 or 96<br>○ E<br>○ Aa<br>○ Ban sani ba<br>○ Babu amsa
317. Zaki so ki so ki tura wani daga iyalin ki ko abokai zuwa ga wurin me bada dabaran nan ko cibiyar lafiyar nan? Provider:	311 a ≠ 34 or 96<br>○ E

${provider_label}	◌ Aa<br>◌ Ban sani ba<br>◌ Babu amsa
SW_1a. Kafin ki far amfani da ${current_recent_label} ${current_recent_start} kina amfani da wani abin ko kuma wata hanyar domin hanawa ko kuma daukar ciki?	302a = 1 OR 306a = 1<br>◌ E<br>◌ Aa<br>◌ Babu amsa
SW_1b. Wacce hanyar tsarin iyalin kike amfani da ita?	SW_1a = 1<br>◌ ashanar fata<br>◌ Robar mahaifa<br>◌ Allurar jinkirta ko hana daukar ciki<br>◌ Kwayoyin hana daukar ciki<br>◌ Dabarar hana daukar ciki ta gaggawa<br>◌ Kwaroron roba na maza<br>◌ Kwaroron roba na mata<br>◌ fai-fai da ake sakawa a bakin mahaifa don hana daukar ciki<br>◌ Kumfa/man shafawa dan jinkirta ko dakatar da daukar ciki<br>◌ Carbin kirga ranakun al'ada<br>◌ LAM<br>◌ Kinyin jima'i lokacin al'ada<br>◌ Yin inzali a waje<br>◌ Wadansu hanyoyin na al'ada<br>◌ Babu amsa
PP_1. Tun bayan haihuwar danki a ${first_birth_lab} kin taba yin wani abin ko amafani da wata hanyar tsarin iyali domin hanawa ko kuma jinkirta daukar ciki?	yaron da aka Haifa a cikin shekar 2 da suka wuce da kuma 302a ≠ 1<br>◌ E<br>◌ Aa<br>◌ Babu amsa
PP_2. Har tsawon wanne lokaci ne bayan kin haihu kafin ki fara amfani da hanyar tsarin iyali?<br>*Shigar da kwana 0 idan yau ne. Zaki shigar da lamba X a skirin na gaba.*	PP_1 = 1 OR (302a = 1 AND child born in the last 2 years)<br>◌ Kwanaki X<br>◌ Sati X<br>◌ Bayan wata X<br>◌ Bayan shekara X<br>◌ Babu amsa
PP_2. A shigar da [HANYAR TSARIN IYALI]<br>*Idan yau ne kadai. Shigar da sipili amsa ba sati sipili ba ko wata sipili*	${pp_method_units} = 'days' or ${pp_method_units} = 'weeks' or ${pp_method_units} = 'months' or ${ …<br>--
PP_3. Wacce hanyar tsarin iyalin ce?	PP_2 ≠ .<br>◌ Yiwa mata aiki a mahaifa<br>◌ Yi wa namiji aiki<br>◌ ashanar fata<br>◌ Robar mahaifa<br>◌ Allurar jinkirta ko hana daukar ciki<br>◌ Kwayoyin hana daukar ciki<br>◌ Dabarar hana daukar ciki ta gaggawa<br>◌ Kwaroron roba na maza<br>◌ Kwaroron roba na mata<br>◌ fai-fai da ake sakawa a bakin mahaifa don hana daukar ciki<br>◌ Kumfa/man shafawa dan jinkirta ko dakatar da daukar ciki<br>◌ Carbin kirga ranakun al'ada<br>◌ LAM<br>◌ Kinyin jima'i lokacin al'ada<br>◌ Yin inzali a waje

	○ Wadansu hanyoyin na al'ada<br>○ Babu amsa
BINCIKA: Shin anyi allurar ne sirinji ko kuma yar karamar allura?<br>*A nuna wa mai bada amsoshin hoton*<br>[sayana_depo_150x300.jpg]	PP_3 = 5<br>○ Sirinji<br>○ karamar allura (Sayana Press)<br>○ Babu amsa
319. Kin taba yin wani abu, ko gwada wata hanya, don jinkirta daukan ciki ko kuma guje wa daukar ciki?	306a ≠ 1 OR 302a ≠ 1<br>○ E<br>○ Aa<br>○ Babu amsa
320. Kina da shekaru nawa kika fara amfani da hanyoyin hanawa ko kuma jinkirta daukar ciki? Mai amsa tambayoyin tace tana yar shekara [AGE] dai dai bayan zagayowar ranar haihuwarta.<br>*Shigar da shekarun. Shigar da -88 idan baa sani ba Shigarda -99 idan ba amsa. Ba zai zama kasa da 9 ba.*	302a = 1 OR 306a =1 OR 319 = 1<br>--
321. 321.Ya'yan ki nawa ne a raye a wannan lokacin?<br>*Shigarda -99 idan ba amsa.*	Age in 320 ≥ 9 AND 200 = 1<br>--

	${fp_ever_used} = 'yes'
322. Wacce hanya kika fara amfani da ita domin jinkirta daukar ciki ko kuma gujewa daukar ciki?<br>*Kar a karanta zabin. Aje kasa dan ganin duka zabin.*	319 = 1<br>○ Yiwa mata aiki a mahaifa<br>○ Yi wa namiji aiki<br>○ ashanar fata<br>○ Robar mahaifa<br>○ Allurar jinkirta ko hana daukar ciki<br>○ Kwayoyin hana daukar ciki<br>○ Dabarar hana daukar ciki ta gaggawa<br>○ Kwaroron roba na maza<br>○ Kwaroron roba na mata<br>○ fai-fai da ake sakawa a bakin mahaifa don hana daukar ciki<br>○ Kumfa/man shafawa dan jinkirta ko dakatar da daukar ciki<br>○ Carbin kirga ranakun al'ada<br>○ LAM<br>○ Kinyin jima’i lokacin al’ada<br>○ Yin inzali a waje<br>○ Wadansu hanyoyin na al'ada<br>○ Babu amsa

LCL_322a. BINCIKA: Shin anyi allurar ta hanyar sirinji ko yar karamar allura?<br>*A nuna wa mai bada amsoshin hoton*<br>[sayana_depo_150x300.jpg]	322 = 5<br>○ Sirinji<br>○ karamar allura (Sayana Press)<br>○ Babu amsa
322a. Have you used emergency contraception at any time in the last 12 months?<br>PROBE: As an emergency measure after unprotected sexual intercourse women can take special pills at any time within three to five days to prevent pregnancy.	32b≠ Hanyar tsarin iyali na gaggawa<br>○ E<br>○ Aa<br>○ Babu amsa

	((${current_user} = 'no')) and (((${more_children_none} = 'no_children') or …
323a. Kin bayyana cewa ba kya bukatar ki sake haihuwa nan kusa kuma bakya amfani da wata hanya domin jinkirta ko hana samun ciki.	302a = 0 AND ((212a or 212b > 2 years) OR (211a or 211b = 2))
323a. Kin bayyana cewa ba kya bukatar ki sake haihuwa kwata-kwata kuma bakya amfani da wata hanya domin jinkirta ko hana samun ciki.	302a = 0 AND ((212a or 212b > 2 years) OR (211a or 211b = 2))
323a. Kin bayyana cewa bakida bukatar haihuwar 'ya'ya k kuma bakya amfani da wata hanya domin jinkirta ko hana samun ciki.	302a = 0 AND ((212a or 212b > 2 years) OR (211a or 211b = 2))
323a. Kin bayyana cewa bakida bukatar haihuwar 'wasu ya'yan kuma bakya amfani da wata hanya domin jinkirta ko hana samun ciki.	302a = 0 AND ((212a or 212b > 2 years) OR (211a or 211b = 2))

Ko zaki iya fadamin meyasa bakya amfani da kowace irin hanya?<br>A KARA TAMBAYA: Shin akwai wani dalili?<br>*A SHIGAR DA DUKKAN DALILAN DA AKA FADA. Ba zai yi wuba a zabi "Ba aure" idan har an zabi "Eh, ida da aure a halin yanzu. Duba sauran amsoshin a kasa*	☐ Banyi aure ba<br>☐ Rashin yin jima'I akai akai/Rashin yin jimai'I gaba daya<br>☐ Daina jinin al'ada<br>☐ Subfecund / Infecund<br>☐ Bata ga haila ba tun wancan watan<br>☐ Shayar da nono<br>☐ Mijina baya nan tsawon lokaci<br>☐ An barwa Ubangiji / fatalistic<br>☐ Wadda akewa tambayar bata yarda ba<br>☐ Mijina ko abokin zamana bai bada goyon baya ba<br>☐ Wadansu wadanda ba'aso<br>☐ Addini ya hana<br>☐ Ba'a san kowacce irin hanya ba<br>☐ Ba'a san yadda za'a samu ba<br>☐ Tsoron matsala<br>☐ Damuwa akan lafiya<br>☐ Rashin hanya mai kyau/wurin yayi nisa<br>☐ Akwai tsada sosai<br>☐ Hanyar da ake bukata babu ita<br>☐ Babu wata hanyar jinkirta ko hana daukar ciki a halin yanzu<br>☐ Wahalar aiki<br>☐ Yanayin jikina na canjawa<br>☐ Wani abu daban<br>☐ Ban sani ba<br>☐ Babu amsa
323b. Shin ke kika yanke shawara kin yin amfani da hanyoyin tsarin iyali, ko kuma shawarar mijinki ce ko kuma ku biyu ne kuka yanke shawarar?	302a ≠ 1<br>○ Mai amsa tambayoyin kawai<br>○ Miji ne kawai/Abokin zama<br>○ Yanke hukunci a tare<br>○ Wani abu daban<br>○ Babu amsa
TCI_304. Bayan ke da mijinki/ abokin zamanki wanene kuma yake da ruwa da tsaki wajen yanke shawarar kada ayi amfani da hanyoyin dakatar da ko hana daukar ciki?<br>PROBE: Bincike da kowa?<br>*Kar a karanta afili. A zabi duk wadda aka fada*	306a = 0<br>☐ Mahafiya<br>☐ Suruka mace<br>☐ Yan uwa mata<br>☐ yan uwan miji mata<br>☐ Kaka mace<br>☐ Abokanai<br>☐ maaikacin lafiya<br>☐ Shugaban al'umma<br>☐ Shugaban addini<br>☐ Anti<br>☐ Wasu 'yan uwan<br>☐ Wani abu daban<br>☐ Babu ko daya<br>☐ Babu amsa
TCI_304x. A cikin watanni goma sha biyu da suka wuce shinaboki / yan uwa sun ja hankalinki akan wata hanyar dakatar da ko hana daukar ciki?	306a = 0<br>○ E<br>○ Aa<br>○ Babu amsa
324. A watanni goma sha biyu da suka gabata ko wani jami'in kiwon lafiya ya ziyarce ki domin yi miki bayani akan hanyoyin bada tazarar haihuwa?	009a = 1<br>○ E<br>○ Aa<br>○ Babu amsa
325a. A cikin watanni goma sha biyu da suka shige, ko kin ziyarci	009a = 1

wata cibiyar kiwon lafiya domin duba lafiyar ki ko yayanki?<br>*Don kowacce harka ta lafiya*	○ E<br>○ Aa<br>○ Babu amsa
325a. A watanni goma sha biyu da suka gabata shin ko kin ziyarci wata cibiyar kula da kiwon lafiya ko sansani domin ki samu magani ko kulawar kanki ko 'ya'yanki?<br>*Don kowacce harka ta lafiya*	009a = 1<br>○ E<br>○ Aa<br>○ Babu amsa
325b. Akwai wani ma'aikacin cibiyar kula da kiwon lafiyar da ya yi miki magana game da hanyoyin tsarin iyali?	325a = 1<br>○ E<br>○ Aa<br>○ Babu amsa
TCI_305. Shin ko kin taba halartar wani taro na jamaa d akayi jawabai masu dadi game da tsarin iyali?	009a = 1<br>○ E<br>○ Aa<br>○ Babu amsa
TCI_306. Shin ko kina tunanin cewa wadansu mutane daga cikin al'umma zasu ke kiranki/ka bakaken maganganu ko su daina muamala da kai/ke idan sun san ka/kina amfani da hanyar dakatar da ko hana daukar ciki?	009a = 1<br>○ E<br>○ Aa<br>○ Ban sani ba<br>○ Babu amsa
TCI_307. Shin ko kina tunanin cewa wadansu mutane daga cikin al'umma zasu ke karfafa miki/ka gwuiwa, da kuma maganganu masu dadi akan ka/ki idan sun san ka/kina amfani da hanyar dakatar da ko hana daukar ciki?	009a = 1<br>○ E<br>○ Aa<br>○ Ban sani ba<br>○ Babu amsa
TCI_308. A cikin watanni goma sha biyu da suka wuce shin ko ka/kinji daya daga cikin wadannan mutane suna magana domin goyon bayan hanyoyin dakatar da ko hana daukar ciki?<br>*A karanta duk zabin sannan a zabi amsar data dace*	009a = 1<br>☐ Ma'aikacin gwamnati (gwamnatin tarayya)<br>☐ Jiha, Karamar hukuma koshugabannin gargajiya<br>☐ Shugaban addini<br>☐ Babu ko daya a cikinsu<br>☐ Babu amsa
TCI_309. A cikin watanni goma sha biyu da suka wuce shin ko ka/kinji daya daga cikin wadannan mutane suna magana domin nuna rashin goyon bayan hanyoyin dakatar da ko hana daukar ciki?<br>*A karanta duk zabin sannan a zabi amsar data dace*	009a = 1<br>☐ Ma'aikacin gwamnati (gwamnatin tarayya)<br>☐ Jiha, Karamar hukuma koshugabannin gargajiya<br>☐ Shugaban addini<br>☐ Babu ko daya a cikinsu<br>☐ Babu amsa
TCI_309x. Mutum nawa ne daga cikin abokanka/ki na kusa kake/kike tuanin suna amfani da dabarun dakatar da ko hana daakr ciki: babu, kadan daga ciki, akasarinsu ko gaba daya?	009a = 1<br>○ Babu<br>○ Wadansu<br>○ Koda yaushe<br>○ Gaba daya<br>○ Ban sani ba<br>○ Babu amsa

			009a = 1
326. A cikin 'yan watanni da suka shige			
	E	Aa	Babu amsa
326a. Kinji batun tsarin iyali a rediyo?	○	○	○
326b. Kinji batun tsarin iyali a talabijin?	○	○	○
326c. Kin karanta batun tsarin iyali a jarida ko mujalla?	○	○	○
326d. Kin samu sako na baki ko a rubuce ta hanyar waya gameda hanyoyin bada tazarar haihuwa?	○	○	○
TCI_310. An karanto dangane da harkokin tsarin iyali daga littafin ka'idoji, takarda mai dauke da bayani ta hannu?	○	○	○
TCI_311. Anga fasta ko allo mai dauke da sako game da tsarin iyali?	○	○	○

Section 4 – Ma'amalar aure

DUBA KIGANI KO AKWAI WANI A WAJEN. KAFIN KI CI GABA, KI YI IYAKA KOKARINKI KI GA CEWAR AKWAI SIRRI

Yanzu zanyi miki tambayoyi ne akan abinda ya shafi mu'amalar aure domin a fahimci wasu al'amuran rayuwa masu muhimci. Ina mai kara tabbatar miki da cewa dukkarn amsar da kika bamu babu wanda zai ji ko ya gani. Idan har kika ga cewar akwai tambayar da bazaki amsa ba, sai ki fada min muje zuwa tamabaya ta gaba	${consent_obtained}

	${consent_obtained}
401a. Shekarun ki nawa a lokacin da ka fara jima'I(kwana da na miji)?	309a = 1
Shekarunta a halin yanzu [SHEKARA]	
Adadin haihuwar da ta zo da rai	${birth_events} > 0
Mai amsa tambayoyin tana da ciki	${pregnant} = 'yes'
Shigar da shekarun.<br>*Shigarda -77 idan bata taba jimai ba. Shigarda -99 idan ba amsa. Shigarda -88 idan baa sani ba.*	--

	((${age_at_first_sex} >= 0) or (${age_at_first_sex} =-88) or (${age_at_first_sex} =-99))
402. Yaushe ne kika yi jima'I na karshe (kwana da na miji)?	401a ≠ -77

402. 402.A shigar da [#kwanaki/sati/wata/shekara]<br>*Idan yau ne, a rubuta sifili a ranaku kawai/ ba sifili ba a satittika/watanni/shekaru.*<br>*Dole ya zama dai dai da lokacin saduwa ta farko da kuma matsayin ciki.*	401a ≠ -77<br>--
LCL_403. Ya danganta da dabi'ar jikinki, idan ace zakiyi jima'i sau daya tak ba tareda kinyi amfani da wata dabarar hana daukar ciki ba, shin a ganinki zaki iya samun juna biyu/ciki?<br>*Karanta zabi a baiyane.*	${consent_obtained}<br>○ Sosai zai iya faruwa<br>○ Zai iya faruwa<br>○ Zai iya faruwa kuma zai iya kin faruwa<br>○ Bazai faru ba<br>○ Ban sani ba<br>○ Babu amsa
LCL_404. Ya danganta da dabi'ar jikinki, idan ace zakiyi jima'i akai akai, wato ace kamar sau biyu a mako guda har tsawon shekara daya ba tareda kinyi amfani da wata dabarar hana daukar ciki ba, shin a ganinki zaki iya samun juna biyu/ciki?<br>*Karanta zabi a baiyane.*	${consent_obtained}<br>○ Sosai zai iya faruwa<br>○ Zai iya faruwa<br>○ Zai iya faruwa kuma zai iya kin faruwa<br>○ Bazai faru ba<br>○ Ban sani ba<br>○ Babu amsa

Section 6 – Kula da jinin al'ada

Now I'm going to ask you about menstrual hygiene management.

602a. A wanne waje kike canja audugar mata, tsumma ko kuma wani abun da kike amfani da shi domin tsaftace kanki?	(209 ≤ 90 days, 13 weeks or ≤ 3 months)<br>○ Tolet ta ruwa<br>○ Bandaki mai bentileshin da masai ta rami<br>○ Masai mai dabe akai<br>○ Maisa wnacce ba slap/budadden bandaki<br>○ Bandakin bokiti<br>○ Masai da ba na ruwa (composting toilet)<br>○ Bandakin da yake a rataye<br>○ Wajen kwana/Dakin kwana<br>○ Bayan gida

	○ Babu wajen yi/jeji/fili<br>○ Wani abu daban<br>○ Babu amsa
Anan kike tsaftace kan ki daga jinin al'ada:<br>*Karanta zabi a baiyane. Zabi E'e*	601 ≠ -99 nor null AND 602 ≠ -99 nor null<br>☐ A tsaftace?<br>☐ Mai zaman kansa<br>☐ Akwai kariya?<br>☐ Za'a iya kullewa<br>☐ Akwai tare da ruwa?<br>☐ Akwai tare da sabulu?<br>☐ Babu ko daya a cikinsu<br>☐ Babu amsa
603. A lokacin da kika ga al'adarki ta baya bayannan, me kikayi amfani da shi domin ki tsane jinin? BINCIKA: Akwai wani abun kuma?<br>*Do not read options aloud. Select all that apply.*	(209 ≤ 90 days, 13 weeks or ≤ 3 months)<br>☐ Audigar mata da ba'a sake amfani da ita (ta siyarwa)<br>☐ Audigar mata da za'a iya sake amfani da ita<br>☐ Sabon kyalle<br>☐ Tsumma<br>☐ Audiga<br>☐ Famfas<br>☐ Audiga<br>☐ Tolet fefa<br>☐ Kayan da ake sawa daga ciki<br>☐ Bokiti<br>☐ Wani abu daban<br>☐ Babu abinda akayi amfani dashi<br>☐ Babu amsa
604a. Shin kin wanke audugar matan, kyalle ko kuma wani abun da ake amfani da shi kuma kika sake amfani dashi/ita a lokacin da kika yi al'adar ki ta karshe?	603 = reusable sanitary pad, old cloth, underwear alone, or bucket<br>○ E<br>○ Aa<br>○ Babu amsa
604b. A lokacin da kika al'adar ki ta baya bayannan, abun da kika wanke kika sake amfani da shi, kin bari ya bushe sosai kafin ki sake amfani das hi?	604a = 1<br>○ E<br>○ Aa<br>○ Babu amsa
605. Kin ce kin yi amfani da [ODK zai fito da amsar da take 603] a lokacin da kika ga al'adar kit a karshe. A wanne waje kika jefar da wadannan abubuwan bayan kin gama amfani da ita?<br>*Do not read options aloud. Select all that apply.*	603= Audugar mata da ba'a sake amfani da ita, sabon kyalle, auduga, tampos, tolet fefa ko kuma 604a=0<br>☐ Tolet ta ruwa<br>☐ Masai/Salga<br>☐ Mazubin shara<br>☐ Konawa<br>☐ Jeji/fili<br>☐ Wani abu daban<br>☐ Babu amsa
606a. Banda aikin kin a gida, kin yi wani aikin kuma a watan day a wuce?	(209 ≤ 90 days, 13 weeks or ≤ 3 months)<br>○ E<br>○ Aa<br>○ Babu amsa
606b. Ko akwai wata rana da baki samu damar zuwa aiki ba a watan da wuce saboda kina jinin al'ada?	606a = 1<br>○ E<br>○ Aa<br>○ Babu amsa
607a. Shin kina zuwa makaranta a cikin watanni 12 da suka wuce?	(209 ≤ 90 days, 13 weeks or ≤ 3 months)<br>○ E<br>○ Aa<br>○ Babu amsa
607b. A cikin watannin 12 da suka wuce, akwai wata rana da baki je makaranta ba saboda kina al'ada?	607a = 1<br>○ E

	○ Aa<br>○ Babu amsa

Sashe 7.1 – Aminai

701. Yanzu ina so in miki wasu tambayoyi game da kawayen ki ko dangi mafi kusa. Waɗannan su ne matan da kike tattauna bayanan sirri da su. Shin su wadannan kawaye ko dangi nawa ne suke tsakanin shekarun 15 zuwa 49 wadanda suke Najeriya?.<br>*Shigar da -88 idan ba'a sani ba, -99 idan ba'a bada amsa ba.*	${consent_obtained}<br>--
702a. Yanzu ki kawo cikin zuciyarki kawarki ta biyu da tafi kusa dake wadda ke cikin Najeriya mai kimanin shekaru 15 zuwa 49, wadda kike baiyanawa bayananki na matukar sirri, wadda itama tana baiyana miki irin nata bayanan na matukar sirrinta. Ki bata wani lakabi ko wani suna na bogi domin ayi amfani dashi a dukkan lokutan da zamuyi magana a gameda ita.	${friend_count} > 0<br>--
703a. Cikakkun shekarun ${friend1_name} nawane?<br>*Shigar da -88 idan ba'a sani ba, -99 idan ba'a bada amsa ba.*	${friend_count} > 0<br>--
704a. Menene zurfin ilimin da ${friend1_name} ta halarta?	${friend_count} > 0<br>○ Ban taba zuwa ba<br>○ Firamare<br>○ Sakandire<br>○ Sama<br>○ Babu amsa
702b. Yanzu ki kawo cikin zuciyarki kawarki ta biyu da tafi kusa dake wadda ke cikin Najeriya mai kimanin shekaru 15 zuwa 49, wadda kike baiyanawa bayananki na matukar sirri, wadda itama tana baiyana miki irin nata bayanan na matukar sirrinta. Ki bata wani lakabi ko wani suna na bogi domin ayi amfani dashi a dukkan lokutan da zamuyi magana a gameda ita.	${friend_count} > 1<br>--
703b. Cikakkun shekarun ${friend2_name} nawane?<br>*Shigar da -88 idan ba'a sani ba, -99 idan ba'a bada amsa ba.*	${friend_count} > 1<br>--
704b. Menene matakin mafi kololuwa na makaranta da ${friend2_name} ta halarta?	${friend_count} > 1<br>○ Ban taba zuwa ba<br>○ Firamare<br>○ Sakandire<br>○ Sama<br>○ Babu amsa

Sashe 7.2 – Bari da zubar da ciki

BINCIKAKO AKWAI WANI/TA A KUSA. A TABBATAR DA BABU WADDA ZAI JI ABUN DA AKE TATTAUNAWA KAFIN A CI GABA.

Tambayoyin da zanyi miki yanzu sun shafi yadda mata a wannan yankin suke zubar da ciki . Wannan abubuwa ne da suke faruwa a kasan nan kuma muna so mu samu kyakkawan bayani a saukake yadda mata ke zubar da chiki. Ina so in tunatar dake cewa wannan tattara bayanan da muke yi na sirri ne matuka kuma amsoshin da kika bani ba za'a gayawa kowa ba. Idan nazo wata tambaya da baki so ki amsa sai ki gaya min domin in tsallake ta zuwa wata tambayan.<br>*Karanta zabi a baiyane.*	${consent_obtained}<br>☐ ko
705. Wasu lokutan mata sukan samu damuwa idan suka samu ciki a lokacin da basa bukatar su sameshi, kuma hakan takan sakasu suyi wani abu domin su suzubar da cikin. Bayyana irin yawaitar yin haka a wannan gari naku da kike zaune a halin yanzu.<br>*Karanta zabi a baiyane.*	${consent_obtained}<br>○ Ya yawaita sosai<br>○ Ya yawaita<br>○ Bai yawaita ba<br>○ Bai yawaita sosai ba<br>○ Ban sani ba<br>○ Babu amsa
706. Wadanne hanyoyine mata a wannan yankin da suka samu damuwa domin samun cikin da basaso ke amfani dasu domin cire cikin ko kuma su zubar da cikin?Wadanne ire-iren hanyoyi ko dabaru matan da suka samu ciki a lokacin da basu bukatar	${consent_obtained}<br>☐ Aikin tiyata<br>☐ Kwayar magani da ake kira

samunsa ke yin amfani dasu domin su zubar da cikin da basaso ko ya saka musu damuwa a wannan gari naku da kike zaune a halin yanzu? Ko akwai wasu hanyoyin kuma bayan wadanda kika ambata?<br>*Kada a karanta zabi a baiyane. A rubuta dukkan zabi da aka baiyana.*	mifepristone or misoprostol<br>☐ Kwayar magani da kike sha lokacin da kikeyin zazzabi, kamar antibiotics ko kuma maganin zazzabin malaria<br>☐ Maganin hana daukar ciki na gaggawa<br>☐ Kwayar magunguna, da ba'a san irinsu ba<br>☐ Allura<br>☐ Magungunna ko dabarun gargajiya, kamar tsirrai<br>☐ Giya<br>☐ Gishiri, maggi, or kanwa<br>☐ Lemon tsami<br>☐ Maganin tari<br>☐ Shigar da wani abu cikin farji<br>☐ Wani abu daban<br>☐ Ban sani ba<br>☐ Babu amsa
707. Wace hanya ce akafi yawan amfani da ita?	count-selected(${abt_ways}) > 1<br>○ Aikin tiyata<br>○ Kwayar magani da ake kira mifepristone or misoprostol<br>○ Kwayar magani da kike sha lokacin da kikeyin zazzabi, kamar antibiotics ko kuma maganin zazzabin malaria<br>○ Maganin hana daukar ciki na gaggawa<br>○ Kwayar magunguna, da ba'a san irinsu ba<br>○ Allura<br>○ Magungunna ko dabarun gargajiya, kamar tsirrai<br>○ Giya<br>○ Gishiri, maggi, or kanwa<br>○ Lemon tsami<br>○ Maganin tari<br>○ Shigar da wani abu cikin farji<br>○ Wani abu daban<br>○ Ban sani ba<br>○ Babu amsa<br>selected(${abt_ways}, filter) or (filter = 'always')
708. A wane wurine mata ke zuwa domin ayi musu aiki a zubar musu da ciki? Ko akwai wasu wuraren kuma bayan wadanda kika ambata?<br>*Do not read options aloud. Select all that apply.*	(selected(${abt_ways},'surgery'))<br>☐ Asibitin gwamnati<br>☐ Cibiyar lafiya ta gwamnati<br>☐ Karamin asibitin samar da dabarun dakatar da ko hana daukar ciki<br>☐ Asibitin tafi da gidan ka (na gwamnati)<br>☐ TBA/Fieldworker (public)<br>☐ Asibitin kudi/kilinik<br>☐ Wajen bayarda magani<br>☐ Kemis / Babban shagon magani<br>☐ Likita ko nurse masu zaman kansu<br>☐ Asibitin tafi da gidan ka (me zaman kasan)<br>☐ Unguwar zoma (masu zaman kan su)<br>☐ Shago

	☐ Kungiyar addini/Coci<br>☐ Aboki/dan uwa ko yar uwa<br>☐ NGO<br>☐ Kasuwa/ 'Yan talla<br>☐ Wani abu daban<br>☐ Ban sani ba<br>☐ Babu amsa
709. Daga jerin wadannan wuraren, wanne akafi zuwa?	count-selected(${abt_surg_where}) > 1<br>○ Asibitin gwamnati<br>○ Cibiyar lafiya ta gwamnati<br>○ Karamin asibitin samar da dabarun dakatar da ko hana daukar ciki<br>○ Asibitin tafi da gidan ka (na gwamnati)<br>○ TBA/Fieldworker (public)<br>○ Asibitin kudi/kilinik<br>○ Wajen bayarda magani<br>○ Kemis / Babban shagon magani<br>○ Likita ko nurse masu zaman kansu<br>○ Asibitin tafi da gidan ka (me zaman kasan)<br>○ Unguwar zoma (masu zaman kan su)<br>○ Shago<br>○ Kungiyar addini/Coci<br>○ Aboki/dan uwa ko yar uwa<br>○ NGO<br>○ Kasuwa/ 'Yan talla<br>○ Wani abu daban<br>○ Ban sani ba<br>○ Babu amsa<br>selected(${abt_surg_where}, filter) or (filter = 'always')
710. A wane wuri/wurare ne mata ke samun magungunan zubar da ciki? Ko akwai wasu wuraren kuma?	(selected(${abt_ways}, 'pills_abortion')) or (selected(${abt_ways}, 'pills_fever')) or (selected($ …<br>☐ Asibitin gwamnati<br>☐ Cibiyar lafiya ta gwamnati<br>☐ Karamin asibitin samar da dabarun dakatar da ko hana daukar ciki<br>☐ Asibitin tafi da gidan ka (na gwamnati)<br>☐ TBA/Fieldworker (public)<br>☐ Asibitin kudi/kilinik<br>☐ Wajen bayarda magani<br>☐ Kemis / Babban shagon magani<br>☐ Likita ko nurse masu zaman kansu<br>☐ Asibitin tafi da gidan ka (me zaman kasan)<br>☐ Unguwar zoma (masu zaman kan su)<br>☐ Shago<br>☐ Kungiyar addini/Coci<br>☐ Aboki/dan uwa ko yar uwa<br>☐ NGO<br>☐ Kasuwa/ 'Yan talla<br>☐ Wani abu daban<br>☐ Ban sani ba<br>☐ Babu amsa
711. Daga jerin wadannan hanyoyi, wacece akafi samun	count-selected(${abt_meds_where}) > 1

magungunan?	○ Asibitin gwamnati<br>○ Cibiyar lafiya ta gwamnati<br>○ Karamin asibitin samar da dabarun dakatar da ko hana daukar ciki<br>○ Asibitin tafi da gidan ka (na gwamnati)<br>○ TBA/Fieldworker (public)<br>○ Asibitin kudi/kilinik<br>○ Wajen bayarda magani<br>○ Kemis / Babban shagon magani<br>○ Likita ko nurse masu zaman kansu<br>○ Asibitin tafi da gidan ka (me zaman kasan)<br>○ Unguwar zoma (masu zaman kan su)<br>○ Shago<br>○ Kungiyar addini/Coci<br>○ Aboki/dan uwa ko yar uwa<br>○ NGO<br>○ Kasuwa/ 'Yan talla<br>○ Wani abu daban<br>○ Ban sani ba<br>○ Babu amsa<br>selected(${abt_meds_where}, filter) or (filter = 'always')
712a.i. Shin ${friend1_name} ko ta tabayin wani abu domin ta zubar da cikin lokacin da tasamu damuwa akan ta samu ciki?<br>*Bincika ko an samu nasarar zubar da cikin.*	○ Yes, I am certain<br>○ Yes, I think so<br>○ Aa<br>○ Ban sani ba<br>○ Babu amsa
713a.i. Ta wacce hanya tayi hakan?<br>*Idan haka ya faru fiye da sau daya, baiyana lokaci na baya baya da hakan ya faru.*<br>*Shigar da 2020 idan ba'a sani ba ko kuma ba'a bada amsa ba.'.*	(${friend1_abt_yn} = 'yes') or (${friend1_abt_yn} = 'likely')<br>Year: ________________
714a.i. Wasu lokutan mata sukan yi abubuwa da dama domin a zubar da ciki. Shin ko ${friend1_name} tayi amfani da hanyoyi fiye da guda daya domin ta cire ciki ko kuma ta zubar da ciki?	(${friend1_abt_yn} = 'yes') or (${friend1_abt_yn} = 'likely')<br>○ Yes, I am certain<br>○ Yes, I think so<br>○ Aa<br>○ Ban sani ba<br>○ Babu amsa
715a.i. Wane mataki ta fara dauka a kokarin ta na cire ko zubar da ciki?	(${friend1_abt_mult_yn} = 'yes') or (${friend1_abt_mult_yn} = 'likely')<br>○ Aikin tiyata<br>○ Kwayar magani da ake kira mifepristone or misoprostol<br>○ Kwayar magani da kike sha lokacin da kikeyin zazzabi, kamar antibiotics ko kuma maganin zazzabin malaria<br>○ Maganin hana daukar ciki na gaggawa<br>○ Kwayar magunguna, da ba'a san irinsu ba<br>○ Allura<br>○ Magungunna ko dabarun gargajiya, kamar tsirrai<br>○ Giya<br>○ Gishiri, maggi, or kanwa<br>○ Lemon tsami<br>○ Maganin tari

	◌ Shigar da wani abu cikin farji<br>◌ Wani abu daban<br>◌ Ban sani ba<br>◌ Babu amsa
715a.i. Wane abu tayi cikin ya fita ko ya zube?	(${friend1_abt_mult_yn} = 'no') or (${friend1_abt_mult_yn} = '-88')<br>◌ Aikin tiyata<br>◌ Kwayar magani da ake kira mifepristone or misoprostol<br>◌ Kwayar magani da kike sha lokacin da kikeyin zazzabi, kamar antibiotics ko kuma maganin zazzabin malaria<br>◌ Maganin hana daukar ciki na gaggawa<br>◌ Kwayar magunguna, da ba'a san irinsu ba<br>◌ Allura<br>◌ Magungunna ko dabarun gargajiya, kamar tsirrai<br>◌ Giya<br>◌ Gishiri, maggi, or kanwa<br>◌ Lemon tsami<br>◌ Maganin tari<br>◌ Shigar da wani abu cikin farji<br>◌ Wani abu daban<br>◌ Ban sani ba<br>◌ Babu amsa
716a.i. A wane wurine aka yi mata aikin?	(${friend1_abt_first} = 'surgery') or (${friend1_abt_only} = 'surgery')<br>◌ Asibitin gwamnati<br>◌ Cibiyar lafiya ta gwamnati<br>◌ Karamin asibitin samar da dabarun dakatar da ko hana daukar ciki<br>◌ Asibitin tafi da gidan ka (na gwamnati)<br>◌ TBA/Fieldworker (public)<br>◌ Asibitin kudi/kilinik<br>◌ Wajen bayarda magani<br>◌ Kemis / Babban shagon magani<br>◌ Likita ko nurse masu zaman kansu<br>◌ Asibitin tafi da gidan ka (me zaman kasan)<br>◌ Unguwar zoma (masu zaman kan su)<br>◌ Shago<br>◌ Kungiyar addini/Coci<br>◌ Aboki/dan uwa ko yar uwa<br>◌ NGO<br>◌ Kasuwa/ 'Yan talla<br>◌ Wani abu daban<br>◌ Ban sani ba<br>◌ Babu amsa
717a.i. A ina ta samu magunguna?	(${friend1_abt_first} = 'pills_abortion') or (${friend1_abt_only} = 'pills_abortion') or (${friend …<br>◌ Asibitin gwamnati<br>◌ Cibiyar lafiya ta gwamnati<br>◌ Karamin asibitin samar da dabarun dakatar da ko hana daukar ciki<br>◌ Asibitin tafi da gidan ka (na

	gwamnati)<br>◌ TBA/Fieldworker (public)<br>◌ Asibitin kudi/kilinik<br>◌ Wajen bayarda magani<br>◌ Kemis / Babban shagon magani<br>◌ Likita ko nurse masu zaman kansu<br>◌ Asibitin tafi da gidan ka (me zaman kasan)<br>◌ Unguwar zoma (masu zaman kan su)<br>◌ Shago<br>◌ Kungiyar addini/Coci<br>◌ Aboki/dan uwa ko yar uwa<br>◌ NGO<br>◌ Kasuwa/ 'Yan talla<br>◌ Wani abu daban<br>◌ Ban sani ba<br>◌ Babu amsa
718a.i. Wane abu tayi na karshe da cikin ya wanku, wato cikin ya zube?	(${friend1_abt_mult_yn} = 'yes') or (${friend1_abt_mult_yn} = 'likely')<br>◌ Aikin tiyata<br>◌ Kwayar magani da ake kira mifepristone or misoprostol<br>◌ Kwayar magani da kike sha lokacin da kikeyin zazzabi, kamar antibiotics ko kuma maganin zazzabin malaria<br>◌ Maganin hana daukar ciki na gaggawa<br>◌ Kwayar magunguna, da ba'a san irinsu ba<br>◌ Allura<br>◌ Magungunna ko dabarun gargajiya, kamar tsirrai<br>◌ Giya<br>◌ Gishiri, maggi, or kanwa<br>◌ Lemon tsami<br>◌ Maganin tari<br>◌ Shigar da wani abu cikin farji<br>◌ Wani abu daban<br>◌ Ban sani ba<br>◌ Babu amsa
719a.i. A wane wurine aka yi mata aikin?	${friend1_abt_last} = 'surgery'<br>◌ Asibitin gwamnati<br>◌ Cibiyar lafiya ta gwamnati<br>◌ Karamin asibitin samar da dabarun dakatar da ko hana daukar ciki<br>◌ Asibitin tafi da gidan ka (na gwamnati)<br>◌ TBA/Fieldworker (public)<br>◌ Asibitin kudi/kilinik<br>◌ Wajen bayarda magani<br>◌ Kemis / Babban shagon magani<br>◌ Likita ko nurse masu zaman kansu<br>◌ Asibitin tafi da gidan ka (me zaman kasan)<br>◌ Unguwar zoma (masu zaman kan su)<br>◌ Shago<br>◌ Kungiyar addini/Coci<br>◌ Aboki/dan uwa ko yar uwa

	◌ NGO<br>◌ Kasuwa/ 'Yan talla<br>◌ Wani abu daban<br>◌ Ban sani ba<br>◌ Babu amsa
720a.i. A ina ta samu magunguna?	(${friend1_abt_last} = 'pills_abortion') or (${friend1_abt_last} = 'pills_fever') or (${friend1_ab …<br>◌ Asibitin gwamnati<br>◌ Cibiyar lafiya ta gwamnati<br>◌ Karamin asibitin samar da dabarun dakatar da ko hana daukar ciki<br>◌ Asibitin tafi da gidan ka (na gwamnati)<br>◌ TBA/Fieldworker (public)<br>◌ Asibitin kudi/kilinik<br>◌ Wajen bayarda magani<br>◌ Kemis / Babban shagon magani<br>◌ Likita ko nurse masu zaman kansu<br>◌ Asibitin tafi da gidan ka (me zaman kasan)<br>◌ Unguwar zoma (masu zaman kan su)<br>◌ Shago<br>◌ Kungiyar addini/Coci<br>◌ Aboki/dan uwa ko yar uwa<br>◌ NGO<br>◌ Kasuwa/ 'Yan talla<br>◌ Wani abu daban<br>◌ Ban sani ba<br>◌ Babu amsa
721a.i. Shin ko ${friend1_name} ta samu matsaloli har kuma taje asibiti domin samun kulawa lokacin ta ake daukar matakan zubar da ciki?<br>*If the respondent already reported the friend went to a health facility in the process of removing the pregnancy, we are interested in whether the friend went back to a health facility on a separate occasion to treat complications that she may have experienced.*	(${friend1_abt_yn} = 'yes') or (${friend1_abt_yn} = 'likely')<br>◌ Yes, I am certain<br>◌ Yes, I think so<br>◌ Aa<br>◌ Ban sani ba<br>◌ Babu amsa
712a.ii. Bayan hakan ta faru da ${friend1_name}, shin ko ta tabayin wani abu domin al'adarta ta dawo lokacin da tasamu damuwa akan ta samu ciki?<br>*Probe to confirm whether the period regulation was successful. If not, select 'no.'*	${friend1_abt_yn} = 'yes'<br>◌ Yes, I am certain<br>◌ Yes, I think so<br>◌ Aa<br>◌ Ban sani ba<br>◌ Babu amsa
712a.ii. Shin ${friend1_name} ta tabayin wani abu domin al'adarta ta dawo lokacin da tasamu damuwa akan ta samu ciki?<br>*Probe to confirm whether the period regulation was successful. If not, select 'no.'*	${friend1_abt_yn} != 'yes'<br>◌ Yes, I am certain<br>◌ Yes, I think so<br>◌ Aa<br>◌ Ban sani ba<br>◌ Babu amsa
713a.ii. Ta wacce hanya tayi hakan?<br>*Idan haka ya faru fiye da sau daya, baiyana lokaci na baya baya da hakan ya faru.*<br>*Shigar da 2020 idan ba'a sani ba ko kuma ba'a bada amsa ba.'.*	(${friend1_reg_yn} = 'yes') or (${friend1_reg_yn} = 'likely')<br>Year: ______________________
714a.ii. Wasu lokutan mata sukan yi abubuwa da dama domin a zubar da ciki. Shin ko ${friend1_name} tayi amfani da hanyoyi fiye da guda daya domin ta cire ciki ko kuma ta zubar da ciki?	((${friend1_reg_year} > ${friend1_abt_year}) or (${friend1_abt_year} = '')) and ((${friend1_reg_yn …<br>◌ Yes, I am certain<br>◌ Yes, I think so<br>◌ Aa<br>◌ Ban sani ba

	Babu amsa
715a.ii. Wane mataki ta fara dauka a kokarin ta na cire ko zubar da ciki?	((${friend1_reg_year} > ${friend1_abt_year}) or (${friend1_abt_year} = '')) and ((${friend1_reg_mu …<br>Aikin tiyata<br>Kwayar magani da ake kira mifepristone or misoprostol<br>Kwayar magani da kike sha lokacin da kikeyin zazzabi, kamar antibiotics ko kuma maganin zazzabin malaria<br>Maganin hana daukar ciki na gaggawa<br>Kwayar magunguna, da ba'a san irinsu ba<br>Allura<br>Magungunna ko dabarun gargajiya, kamar tsirrai<br>Giya<br>Gishiri, maggi, or kanwa<br>Lemon tsami<br>Maganin tari<br>Shigar da wani abu cikin farji<br>Wani abu daban<br>Ban sani ba<br>Babu amsa
715a.ii. Wane abu tayi cikin ya fita ko ya zube?	((${friend1_reg_year} > ${friend1_abt_year}) or (${friend1_abt_year} = '')) and ((${friend1_reg_mu …<br>Aikin tiyata<br>Kwayar magani da ake kira mifepristone or misoprostol<br>Kwayar magani da kike sha lokacin da kikeyin zazzabi, kamar antibiotics ko kuma maganin zazzabin malaria<br>Maganin hana daukar ciki na gaggawa<br>Kwayar magunguna, da ba'a san irinsu ba<br>Allura<br>Magungunna ko dabarun gargajiya, kamar tsirrai<br>Giya<br>Gishiri, maggi, or kanwa<br>Lemon tsami<br>Maganin tari<br>Shigar da wani abu cikin farji<br>Wani abu daban<br>Ban sani ba<br>Babu amsa
716a.ii. A wane wurine aka yi mata aikin?	((${friend1_reg_year} > ${friend1_abt_year}) or (${friend1_abt_year} = '')) and ((${friend1_reg_fi …<br>Asibitin gwamnati<br>Cibiyar lafiya ta gwamnati<br>Karamin asibitin samar da dabarun dakatar da ko hana daukar ciki<br>Asibitin tafi da gidan ka (na gwamnati)<br>TBA/Fieldworker (public)

	○ Asibitin kudi/kilinik<br>○ Wajen bayarda magani<br>○ Kemis / Babban shagon magani<br>○ Likita ko nurse masu zaman kansu<br>○ Asibitin tafi da gidan ka (me zaman kasan)<br>○ Unguwar zoma (masu zaman kan su)<br>○ Shago<br>○ Kungiyar addini/Coci<br>○ Aboki/dan uwa ko yar uwa<br>○ NGO<br>○ Kasuwa/ 'Yan talla<br>○ Wani abu daban<br>○ Ban sani ba<br>○ Babu amsa
717a.ii. A ina ta samu magunguna?	((${friend1_reg_year} > ${friend1_abt_year}) or (${friend1_abt_year} = '')) and ((${friend1_reg_fi …<br>○ Asibitin gwamnati<br>○ Cibiyar lafiya ta gwamnati<br>○ Karamin asibitin samar da dabarun dakatar da ko hana daukar ciki<br>○ Asibitin tafi da gidan ka (na gwamnati)<br>○ TBA/Fieldworker (public)<br>○ Asibitin kudi/kilinik<br>○ Wajen bayarda magani<br>○ Kemis / Babban shagon magani<br>○ Likita ko nurse masu zaman kansu<br>○ Asibitin tafi da gidan ka (me zaman kasan)<br>○ Unguwar zoma (masu zaman kan su)<br>○ Shago<br>○ Kungiyar addini/Coci<br>○ Aboki/dan uwa ko yar uwa<br>○ NGO<br>○ Kasuwa/ 'Yan talla<br>○ Wani abu daban<br>○ Ban sani ba<br>○ Babu amsa
718a.ii. Wane abu tayi na karshe da cikin ya wanku, wato cikin ya zube?	((${friend1_reg_year} > ${friend1_abt_year}) or (${friend1_abt_year} = '')) and ((${friend1_reg_mu …<br>○ Aikin tiyata<br>○ Kwayar magani da ake kira mifepristone or misoprostol<br>○ Kwayar magani da kike sha lokacin da kikeyin zazzabi, kamar antibiotics ko kuma maganin zazzabin malaria<br>○ Maganin hana daukar ciki na gaggawa<br>○ Kwayar magunguna, da ba'a san irinsu ba<br>○ Allura<br>○ Magungunna ko dabarun gargajiya, kamar tsirrai<br>○ Giya<br>○ Gishiri, maggi, or kanwa<br>○ Lemon tsami

	Maganin tari<br>Shigar da wani abu cikin farji<br>Wani abu daban<br>Ban sani ba<br>Babu amsa
719a.ii. A wane wurine aka yi mata aikin?	((${friend1_reg_year} > ${friend1_abt_year}) or (${friend1_abt_year} = '')) and (${friend1_reg_las …<br>Asibitin gwamnati<br>Cibiyar lafiya ta gwamnati<br>Karamin asibitin samar da dabarun dakatar da ko hana daukar ciki<br>Asibitin tafi da gidan ka (na gwamnati)<br>TBA/Fieldworker (public)<br>Asibitin kudi/kilinik<br>Wajen bayarda magani<br>Kemis / Babban shagon magani<br>Likita ko nurse masu zaman kansu<br>Asibitin tafi da gidan ka (me zaman kasan)<br>Unguwar zoma (masu zaman kan su)<br>Shago<br>Kungiyar addini/Coci<br>Aboki/dan uwa ko yar uwa<br>NGO<br>Kasuwa/ 'Yan talla<br>Wani abu daban<br>Ban sani ba<br>Babu amsa
720a.ii. A ina ta samu magunguna?	((${friend1_reg_year} > ${friend1_abt_year}) or (${friend1_abt_year} = '')) and ((${friend1_reg_la …<br>Asibitin gwamnati<br>Cibiyar lafiya ta gwamnati<br>Karamin asibitin samar da dabarun dakatar da ko hana daukar ciki<br>Asibitin tafi da gidan ka (na gwamnati)<br>TBA/Fieldworker (public)<br>Asibitin kudi/kilinik<br>Wajen bayarda magani<br>Kemis / Babban shagon magani<br>Likita ko nurse masu zaman kansu<br>Asibitin tafi da gidan ka (me zaman kasan)<br>Unguwar zoma (masu zaman kan su)<br>Shago<br>Kungiyar addini/Coci<br>Aboki/dan uwa ko yar uwa<br>NGO<br>Kasuwa/ 'Yan talla<br>Wani abu daban<br>Ban sani ba<br>Babu amsa
721a.ii. Shin ko ${friend1_name} ta samu matsaloli har kuma taje asibiti domin samun kulawa lokacin ta ake daukar matakan zubar da ciki?	((${friend1_reg_year} > ${friend1_abt_year}) or (${friend1_abt_year} = '')) and ((${friend1_reg_yn …

If the respondent already reported the friend went to a health facility in the process of regulating her period, we are interested in whether the friend went back to a health facility on a separate occasion to treat complications that she may have experienced.	○ Yes, I am certain<br>○ Yes, I think so<br>○ Aa<br>○ Ban sani ba<br>○ Babu amsa
712b.i. Shin ${friend2_name} ko ta tabayin wani abu domin ta zubar da cikin lokacin da tasamu damuwa akan ta samu ciki?<br>*Bincika ko an samu nasarar zubar da cikin.*	○ Yes, I am certain<br>○ Yes, I think so<br>○ Aa<br>○ Ban sani ba<br>○ Babu amsa
713b.i. Ta wacce hanya tayi hakan?<br>*Idan haka ya faru fiye da sau daya, baiyana lokaci na baya baya da hakan ya faru.*<br>*Shigar da 2020 idan ba'a sani ba ko kuma ba'a bada amsa ba.'.*	(${friend2_abt_yn} = 'yes') or (${friend2_abt_yn} = 'likely')<br>Year: ________________
714b.i. Wasu lokutan mata sukan yi abubuwa da dama domin a zubar da ciki. Shin ko ${friend2_name} tayi amfani da hanyoyi fiye da guda daya domin ta cire ciki ko kuma ta zubar da ciki?	(${friend2_abt_yn} = 'yes') or (${friend2_abt_yn} = 'likely')<br>○ Yes, I am certain<br>○ Yes, I think so<br>○ Aa<br>○ Ban sani ba<br>○ Babu amsa
715b.i. Wane mataki ta fara dauka a kokarin ta na cire ko zubar da ciki?	(${friend2_abt_mult_yn} = 'yes') or (${friend2_abt_mult_yn} = 'likely')<br>○ Aikin tiyata<br>○ Kwayar magani da ake kira mifepristone or misoprostol<br>○ Kwayar magani da kike sha lokacin da kikeyin zazzabi, kamar antibiotics ko kuma maganin zazzabin malaria<br>○ Maganin hana daukar ciki na gaggawa<br>○ Kwayar magunguna, da ba'a san irinsu ba<br>○ Allura<br>○ Magungunna ko dabarun gargajiya, kamar tsirrai<br>○ Giya<br>○ Gishiri, maggi, or kanwa<br>○ Lemon tsami<br>○ Maganin tari<br>○ Shigar da wani abu cikin farji<br>○ Wani abu daban<br>○ Ban sani ba<br>○ Babu amsa
715b.i. Wane abu tayi cikin ya fita ko ya zube?	(${friend2_abt_mult_yn} = 'no') or (${friend2_abt_mult_yn} = '-88')<br>○ Aikin tiyata<br>○ Kwayar magani da ake kira mifepristone or misoprostol<br>○ Kwayar magani da kike sha lokacin da kikeyin zazzabi, kamar antibiotics ko kuma maganin zazzabin malaria<br>○ Maganin hana daukar ciki na gaggawa<br>○ Kwayar magunguna, da ba'a san irinsu ba<br>○ Allura<br>○ Magungunna ko dabarun gargajiya, kamar tsirrai<br>○ Giya

	○ Gishiri, maggi, or kanwa<br>○ Lemon tsami<br>○ Maganin tari<br>○ Shigar da wani abu cikin farji<br>○ Wani abu daban<br>○ Ban sani ba<br>○ Babu amsa
716b.i. A wane wurine aka yi mata aikin?	(${friend2_abt_first} = 'surgery') or (${friend2_abt_only} = 'surgery')<br>○ Asibitin gwamnati<br>○ Cibiyar lafiya ta gwamnati<br>○ Karamin asibitin samar da dabarun dakatar da ko hana daukar ciki<br>○ Asibitin tafi da gidan ka (na gwamnati)<br>○ TBA/Fieldworker (public)<br>○ Asibitin kudi/kilinik<br>○ Wajen bayarda magani<br>○ Kemis / Babban shagon magani<br>○ Likita ko nurse masu zaman kansu<br>○ Asibitin tafi da gidan ka (me zaman kasan)<br>○ Unguwar zoma (masu zaman kan su)<br>○ Shago<br>○ Kungiyar addini/Coci<br>○ Aboki/dan uwa ko yar uwa<br>○ NGO<br>○ Kasuwa/ 'Yan talla<br>○ Wani abu daban<br>○ Ban sani ba<br>○ Babu amsa
717b.i. A ina ta samu magunguna?	(${friend2_abt_first} = 'pills_abortion') or (${friend2_abt_only} = 'pills_abortion') or (${friend …<br>○ Asibitin gwamnati<br>○ Cibiyar lafiya ta gwamnati<br>○ Karamin asibitin samar da dabarun dakatar da ko hana daukar ciki<br>○ Asibitin tafi da gidan ka (na gwamnati)<br>○ TBA/Fieldworker (public)<br>○ Asibitin kudi/kilinik<br>○ Wajen bayarda magani<br>○ Kemis / Babban shagon magani<br>○ Likita ko nurse masu zaman kansu<br>○ Asibitin tafi da gidan ka (me zaman kasan)<br>○ Unguwar zoma (masu zaman kan su)<br>○ Shago<br>○ Kungiyar addini/Coci<br>○ Aboki/dan uwa ko yar uwa<br>○ NGO<br>○ Kasuwa/ 'Yan talla<br>○ Wani abu daban<br>○ Ban sani ba<br>○ Babu amsa
718b.i. Wane abu tayi na karshe da cikin ya wanku, wato cikin ya zube?	(${friend2_abt_mult_yn} = 'yes') or (${friend2_abt_mult_yn} = 'likely')

	◌ Aikin tiyata<br>◌ Kwayar magani da ake kira mifepristone or misoprostol<br>◌ Kwayar magani da kike sha lokacin da kikeyin zazzabi, kamar antibiotics ko kuma maganin zazzabin malaria<br>◌ Maganin hana daukar ciki na gaggawa<br>◌ Kwayar magunguna, da ba'a san irinsu ba<br>◌ Allura<br>◌ Magungunna ko dabarun gargajiya, kamar tsirrai<br>◌ Giya<br>◌ Gishiri, maggi, or kanwa<br>◌ Lemon tsami<br>◌ Maganin tari<br>◌ Shigar da wani abu cikin farji<br>◌ Wani abu daban<br>◌ Ban sani ba<br>◌ Babu amsa
719b.i. A wane wurine aka yi mata aikin?	${friend2_abt_last} = 'surgery'<br>◌ Asibitin gwamnati<br>◌ Cibiyar lafiya ta gwamnati<br>◌ Karamin asibitin samar da dabarun dakatar da ko hana daukar ciki<br>◌ Asibitin tafi da gidan ka (na gwamnati)<br>◌ TBA/Fieldworker (public)<br>◌ Asibitin kudi/kilinik<br>◌ Wajen bayarda magani<br>◌ Kemis / Babban shagon magani<br>◌ Likita ko nurse masu zaman kansu<br>◌ Asibitin tafi da gidan ka (me zaman kasan)<br>◌ Unguwar zoma (masu zaman kan su)<br>◌ Shago<br>◌ Kungiyar addini/Coci<br>◌ Aboki/dan uwa ko yar uwa<br>◌ NGO<br>◌ Kasuwa/ 'Yan talla<br>◌ Wani abu daban<br>◌ Ban sani ba<br>◌ Babu amsa
720b.i. A ina ta samu magunguna?	(${friend2_abt_last} = 'pills_abortion') or (${friend2_abt_last} = 'pills_fever') or (${friend2_ab …<br>◌ Asibitin gwamnati<br>◌ Cibiyar lafiya ta gwamnati<br>◌ Karamin asibitin samar da dabarun dakatar da ko hana daukar ciki<br>◌ Asibitin tafi da gidan ka (na gwamnati)<br>◌ TBA/Fieldworker (public)<br>◌ Asibitin kudi/kilinik<br>◌ Wajen bayarda magani<br>◌ Kemis / Babban shagon magani<br>◌ Likita ko nurse masu zaman kansu

	○ Asibitin tafi da gidan ka (me zaman kasan)<br>○ Unguwar zoma (masu zaman kan su)<br>○ Shago<br>○ Kungiyar addini/Coci<br>○ Aboki/dan uwa ko yar uwa<br>○ NGO<br>○ Kasuwa/ 'Yan talla<br>○ Wani abu daban<br>○ Ban sani ba<br>○ Babu amsa
721b.i. Shin ko ${friend2_name} ta samu matsaloli har kuma taje asibiti domin samun kulawa lokacin ta ake daukar matakan zubar da ciki?<br>*Idan mai amsa tambaya ta riga ta fada miki cewa kawar ta ta tafi asibiti yayin zubar da ciki, muna so mu sani ko kawar tata ta tafi asibitin ne a wani lokaci na daban saboda samun kulawa domin damuwar da ta samu lokacin zubar da cikin.*	(${friend2_abt_yn} = 'yes') or (${friend2_abt_yn} = 'likely')<br>○ Yes, I am certain<br>○ Yes, I think so<br>○ Aa<br>○ Ban sani ba<br>○ Babu amsa
712b.ii. Bayan hakan ta faru da ${friend2_name}, shin ko ta tabayin wani abu domin al'adarta ta dawo lokacin da tasamu damuwa akan ta samu ciki?<br>*Probe to confirm whether the period regulation was successful. If not, select 'no.'*	${friend2_abt_yn} = 'yes'<br>○ Yes, I am certain<br>○ Yes, I think so<br>○ Aa<br>○ Ban sani ba<br>○ Babu amsa
712b.ii. Shin ${friend2_name} ta tabayin wani abu domin al'adarta ta dawo lokacin da tasamu damuwa akan ta samu ciki?<br>*Probe to confirm whether the period regulation was successful. If not, select 'no.'*	${friend2_abt_yn} != 'yes'<br>○ Yes, I am certain<br>○ Yes, I think so<br>○ Aa<br>○ Ban sani ba<br>○ Babu amsa
713b.ii. Ta wacce hanya tayi hakan?<br>*Idan haka ya faru fiye da sau daya, baiyana lokaci na baya baya da hakan ya faru.*<br>*Shigar da 2020 idan ba'a sani ba ko kuma ba'a bada amsa ba.'.*	(${friend2_reg_yn} = 'yes') or (${friend2_reg_yn} = 'likely')<br>Year: ___________________
714b.ii. Wasu lokutan mata sukan yi abubuwa da dama domin a zubar da ciki. Shin ko ${friend2_name} tayi amfani da hanyoyi fiye da guda daya domin ta cire ciki ko kuma ta zubar da ciki?	((${friend2_reg_year} > ${friend2_abt_year}) or (${friend2_abt_year} = '')) and ((${friend2_reg_yn …<br>○ Yes, I am certain<br>○ Yes, I think so<br>○ Aa<br>○ Ban sani ba<br>○ Babu amsa
715b.ii. Wane mataki ta fara dauka a kokarin ta na cire ko zubar da ciki?	((${friend2_reg_year} > ${friend2_abt_year}) or (${friend2_abt_year} = '')) and ((${friend2_reg_mu …<br>○ Aikin tiyata<br>○ Kwayar magani da ake kira mifepristone or misoprostol<br>○ Kwayar magani da kike sha lokacin da kikeyin zazzabi, kamar antibiotics ko kuma maganin zazzabin malaria<br>○ Maganin hana daukar ciki na gaggawa<br>○ Kwayar magunguna, da ba'a san irinsu ba<br>○ Allura<br>○ Magungunna ko dabarun gargajiya, kamar tsirrai<br>○ Giya<br>○ Gishiri, maggi, or kanwa

	Lemon tsami<br>Maganin tari<br>Shigar da wani abu cikin farji<br>Wani abu daban<br>Ban sani ba<br>Babu amsa
715b.ii. Wane abu tayi cikin ya fita ko ya zube?	((${friend2_reg_year} > ${friend2_abt_year}) or (${friend2_abt_year} = '')) and ((${friend2_reg_mu …<br>Aikin tiyata<br>Kwayar magani da ake kira mifepristone or misoprostol<br>Kwayar magani da kike sha lokacin da kikeyin zazzabi, kamar antibiotics ko kuma maganin zazzabin malaria<br>Maganin hana daukar ciki na gaggawa<br>Kwayar magunguna, da ba'a san irinsu ba<br>Allura<br>Magungunna ko dabarun gargajiya, kamar tsirrai<br>Giya<br>Gishiri, maggi, or kanwa<br>Lemon tsami<br>Maganin tari<br>Shigar da wani abu cikin farji<br>Wani abu daban<br>Ban sani ba<br>Babu amsa
716b.ii. A wane wurine aka yi mata aikin?	((${friend2_reg_year} > ${friend2_abt_year}) or (${friend2_abt_year} = '')) and ((${friend2_reg_fi …<br>Asibitin gwamnati<br>Cibiyar lafiya ta gwamnati<br>Karamin asibitin samar da dabarun dakatar da ko hana daukar ciki<br>Asibitin tafi da gidan ka (na gwamnati)<br>TBA/Fieldworker (public)<br>Asibitin kudi/kilinik<br>Wajen bayarda magani<br>Kemis / Babban shagon magani<br>Likita ko nurse masu zaman kansu<br>Asibitin tafi da gidan ka (me zaman kasan)<br>Unguwar zoma (masu zaman kan su)<br>Shago<br>Kungiyar addini/Coci<br>Aboki/dan uwa ko yar uwa<br>NGO<br>Kasuwa/ 'Yan talla<br>Wani abu daban<br>Ban sani ba<br>Babu amsa
717b.ii. A ina ta samu magunguna?	((${friend2_reg_year} > ${friend2_abt_year}) or (${friend2_abt_year} = '')) and ((${friend2_reg_fi …<br>Asibitin gwamnati<br>Cibiyar lafiya ta gwamnati

	◌ Karamin asibitin samar da dabarun dakatar da ko hana daukar ciki<br>◌ Asibitin tafi da gidan ka (na gwamnati)<br>◌ TBA/Fieldworker (public)<br>◌ Asibitin kudi/kilinik<br>◌ Wajen bayarda magani<br>◌ Kemis / Babban shagon magani<br>◌ Likita ko nurse masu zaman kansu<br>◌ Asibitin tafi da gidan ka (me zaman kasan)<br>◌ Unguwar zoma (masu zaman kan su)<br>◌ Shago<br>◌ Kungiyar addini/Coci<br>◌ Aboki/dan uwa ko yar uwa<br>◌ NGO<br>◌ Kasuwa/ 'Yan talla<br>◌ Wani abu daban<br>◌ Ban sani ba<br>◌ Babu amsa
718b.ii. Wane abu tayi na karshe da cikin ya wanku, wato cikin ya zube?	((${friend2_reg_year} > ${friend2_abt_year}) or (${friend2_abt_year} = '')) and ((${friend2_reg_mu …<br>◌ Aikin tiyata<br>◌ Kwayar magani da ake kira mifepristone or misoprostol<br>◌ Kwayar magani da kike sha lokacin da kikeyin zazzabi, kamar antibiotics ko kuma maganin zazzabin malaria<br>◌ Maganin hana daukar ciki na gaggawa<br>◌ Kwayar magunguna, da ba'a san irinsu ba<br>◌ Allura<br>◌ Magungunna ko dabarun gargajiya, kamar tsirrai<br>◌ Giya<br>◌ Gishiri, maggi, or kanwa<br>◌ Lemon tsami<br>◌ Maganin tari<br>◌ Shigar da wani abu cikin farji<br>◌ Wani abu daban<br>◌ Ban sani ba<br>◌ Babu amsa
719b.ii. A wane wurine aka yi mata aikin?	((${friend2_reg_year} > ${friend2_abt_year}) or (${friend2_abt_year} = '')) and (${friend2_reg_las …<br>◌ Asibitin gwamnati<br>◌ Cibiyar lafiya ta gwamnati<br>◌ Karamin asibitin samar da dabarun dakatar da ko hana daukar ciki<br>◌ Asibitin tafi da gidan ka (na gwamnati)<br>◌ TBA/Fieldworker (public)<br>◌ Asibitin kudi/kilinik<br>◌ Wajen bayarda magani<br>◌ Kemis / Babban shagon magani<br>◌ Likita ko nurse masu zaman kansu

	◌ Asibitin tafi da gidan ka (me zaman kasan)<br>◌ Unguwar zoma (masu zaman kan su)<br>◌ Shago<br>◌ Kungiyar addini/Coci<br>◌ Aboki/dan uwa ko yar uwa<br>◌ NGO<br>◌ Kasuwa/ 'Yan talla<br>◌ Wani abu daban<br>◌ Ban sani ba<br>◌ Babu amsa
720b.ii. A ina ta samu magunguna?	((${friend2_reg_year} > ${friend2_abt_year}) or (${friend2_abt_year} = '')) and ((${friend2_reg_la …<br>◌ Asibitin gwamnati<br>◌ Cibiyar lafiya ta gwamnati<br>◌ Karamin asibitin samar da dabarun dakatar da ko hana daukar ciki<br>◌ Asibitin tafi da gidan ka (na gwamnati)<br>◌ TBA/Fieldworker (public)<br>◌ Asibitin kudi/kilinik<br>◌ Wajen bayarda magani<br>◌ Kemis / Babban shagon magani<br>◌ Likita ko nurse masu zaman kansu<br>◌ Asibitin tafi da gidan ka (me zaman kasan)<br>◌ Unguwar zoma (masu zaman kan su)<br>◌ Shago<br>◌ Kungiyar addini/Coci<br>◌ Aboki/dan uwa ko yar uwa<br>◌ NGO<br>◌ Kasuwa/ 'Yan talla<br>◌ Wani abu daban<br>◌ Ban sani ba<br>◌ Babu amsa
721b.ii. Shin ko ${friend2_name} ta samu matsaloli har kuma taje asibiti domin samun kulawa lokacin ta ake daukar matakan zubar da ciki?<br>*If the respondent already reported the friend went to a health facility in the process of regulating her period, we are interested in whether the friend went back to a health facility on a separate occasion to treat complications that she may have experienced.*	((${friend2_reg_year} > ${friend2_abt_year}) or (${friend2_abt_year} = '')) and ((${friend2_reg_yn …<br>◌ Yes, I am certain<br>◌ Yes, I think so<br>◌ Aa<br>◌ Ban sani ba<br>◌ Babu amsa
722a. Shin ko kin tabayin wani abu domin ki zubar da cikin lokacin da kika samu damuwa akan kin samu ciki?<br>*Bincika ko an samu nasarar zubar da cikin.*	◌ E<br>◌ Aa<br>◌ Babu amsa
723a. A wace shekara wannan ya faru?<br>*Idan haka ya faru fiye da sau daya, baiyana lokaci na baya baya da hakan ya faru.*<br>*Shigar da 2020 idan ba'a sani ba ko kuma ba'a bada amsa ba.'.*	(${self_abt_yn} = 'yes')<br>Year: ____________
724a. Shin ko kinyi abubuwa fiye da daya domin cikin ya wanku, wato cikin ya zube?	(${self_abt_yn} = 'yes')<br>◌ E<br>◌ Aa<br>◌ Babu amsa
725a. Wane abu kika fara yi?	(${self_abt_mult_yn} = 'yes')<br>◌ Aikin tiyata<br>◌ Kwayar magani da ake kira mifepristone or misoprostol

	◌ Kwayar magani da kike sha lokacin da kikeyin zazzabi, kamar antibiotics ko kuma maganin zazzabin malaria<br>◌ Maganin hana daukar ciki na gaggawa<br>◌ Kwayar magunguna, da ba'a san irinsu ba<br>◌ Allura<br>◌ Magungunna ko dabarun gargajiya, kamar tsirrai<br>◌ Giya<br>◌ Gishiri, maggi, or kanwa<br>◌ Lemon tsami<br>◌ Maganin tari<br>◌ Shigar da wani abu cikin farji<br>◌ Wani abu daban<br>◌ Ban sani ba<br>◌ Babu amsa
725a. Wane abu kika yi?	(${self_abt_mult_yn} = 'no')<br>◌ Aikin tiyata<br>◌ Kwayar magani da ake kira mifepristone or misoprostol<br>◌ Kwayar magani da kike sha lokacin da kikeyin zazzabi, kamar antibiotics ko kuma maganin zazzabin malaria<br>◌ Maganin hana daukar ciki na gaggawa<br>◌ Kwayar magunguna, da ba'a san irinsu ba<br>◌ Allura<br>◌ Magungunna ko dabarun gargajiya, kamar tsirrai<br>◌ Giya<br>◌ Gishiri, maggi, or kanwa<br>◌ Lemon tsami<br>◌ Maganin tari<br>◌ Shigar da wani abu cikin farji<br>◌ Wani abu daban<br>◌ Ban sani ba<br>◌ Babu amsa
726a. Wane wuri kika je akayi miki aiki?	(${self_abt_first} = 'surgery') or (${self_abt_only} = 'surgery')<br>◌ Asibitin gwamnati<br>◌ Cibiyar lafiya ta gwamnati<br>◌ Karamin asibitin samar da dabarun dakatar da ko hana daukar ciki<br>◌ Asibitin tafi da gidan ka (na gwamnati)<br>◌ TBA/Fieldworker (public)<br>◌ Asibitin kudi/kilinik<br>◌ Wajen bayarda magani<br>◌ Kemis / Babban shagon magani<br>◌ Likita ko nurse masu zaman kansu<br>◌ Asibitin tafi da gidan ka (me zaman kasan)<br>◌ Unguwar zoma (masu zaman kan su)<br>◌ Shago

	◌ Kungiyar addini/Coci<br>◌ Aboki/dan uwa ko yar uwa<br>◌ NGO<br>◌ Kasuwa/ 'Yan talla<br>◌ Wani abu daban<br>◌ Ban sani ba<br>◌ Babu amsa
727a. Wane wuri kika je akayi miki aiki?	(${self_abt_first} = 'pills_abortion') or (${self_abt_only} = 'pills_abortion') or (${self_abt_fir …<br>◌ Asibitin gwamnati<br>◌ Cibiyar lafiya ta gwamnati<br>◌ Karamin asibitin samar da dabarun dakatar da ko hana daukar ciki<br>◌ Asibitin tafi da gidan ka (na gwamnati)<br>◌ TBA/Fieldworker (public)<br>◌ Asibitin kudi/kilinik<br>◌ Wajen bayarda magani<br>◌ Kemis / Babban shagon magani<br>◌ Likita ko nurse masu zaman kansu<br>◌ Asibitin tafi da gidan ka (me zaman kasan)<br>◌ Unguwar zoma (masu zaman kan su)<br>◌ Shago<br>◌ Kungiyar addini/Coci<br>◌ Aboki/dan uwa ko yar uwa<br>◌ NGO<br>◌ Kasuwa/ 'Yan talla<br>◌ Wani abu daban<br>◌ Ban sani ba<br>◌ Babu amsa
728a. Wane abu kikayi a karshe?	(${self_abt_mult_yn} = 'yes')<br>◌ Aikin tiyata<br>◌ Kwayar magani da ake kira mifepristone or misoprostol<br>◌ Kwayar magani da kike sha lokacin da kikeyin zazzabi, kamar antibiotics ko kuma maganin zazzabin malaria<br>◌ Maganin hana daukar ciki na gaggawa<br>◌ Kwayar magunguna, da ba'a san irinsu ba<br>◌ Allura<br>◌ Magungunna ko dabarun gargajiya, kamar tsirrai<br>◌ Giya<br>◌ Gishiri, maggi, or kanwa<br>◌ Lemon tsami<br>◌ Maganin tari<br>◌ Shigar da wani abu cikin farji<br>◌ Wani abu daban<br>◌ Ban sani ba<br>◌ Babu amsa
729a. Wane wuri kika je akayi miki aiki?	${self_abt_last} = 'surgery'<br>◌ Asibitin gwamnati<br>◌ Cibiyar lafiya ta gwamnati<br>◌ Karamin asibitin samar da dabarun dakatar da ko hana daukar

	ciki<br>○ Asibitin tafi da gidan ka (na gwamnati)<br>○ TBA/Fieldworker (public)<br>○ Asibitin kudi/kilinik<br>○ Wajen bayarda magani<br>○ Kemis / Babban shagon magani<br>○ Likita ko nurse masu zaman kansu<br>○ Asibitin tafi da gidan ka (me zaman kasan)<br>○ Unguwar zoma (masu zaman kan su)<br>○ Shago<br>○ Kungiyar addini/Coci<br>○ Aboki/dan uwa ko yar uwa<br>○ NGO<br>○ Kasuwa/ 'Yan talla<br>○ Wani abu daban<br>○ Ban sani ba<br>○ Babu amsa
730a. Wane wuri kika je kika samu magunguna?	(${self_abt_last} = 'pills_abortion') or (${self_abt_last} = 'pills_fever') or (${self_abt_last} = …<br>○ Asibitin gwamnati<br>○ Cibiyar lafiya ta gwamnati<br>○ Karamin asibitin samar da dabarun dakatar da ko hana daukar ciki<br>○ Asibitin tafi da gidan ka (na gwamnati)<br>○ TBA/Fieldworker (public)<br>○ Asibitin kudi/kilinik<br>○ Wajen bayarda magani<br>○ Kemis / Babban shagon magani<br>○ Likita ko nurse masu zaman kansu<br>○ Asibitin tafi da gidan ka (me zaman kasan)<br>○ Unguwar zoma (masu zaman kan su)<br>○ Shago<br>○ Kungiyar addini/Coci<br>○ Aboki/dan uwa ko yar uwa<br>○ NGO<br>○ Kasuwa/ 'Yan talla<br>○ Wani abu daban<br>○ Ban sani ba<br>○ Babu amsa
731a. Shin ko kin samu wata matsala kuma kikaje asibiti domin samun kulawa a lokacin zubar da cikin?<br>*If the respondent already reported she went to a health facility in the process of removing the pregnancy, we are interested in whether she went back to a health facility on a separate occasion to treat complications that she may have experienced.*	(${self_abt_yn} = 'yes')<br>○ E<br>○ Aa<br>○ Ban sani ba<br>○ Babu amsa
732a. Aane wuri kika je akayi miki aiki?<br>*Kada a karanta zabi a baiyane. A rubuta dukkan zabi da aka baiyana.*	(${self_abt_yn} = 'yes')<br>☐ Miji/abokin zama<br>☐ Yar'uwa<br>☐ Dan'uwa<br>☐ Mahafiya<br>☐ Uba<br>☐ Dangi na dabam<br>☐ Babbar Kawa ta 1:${friend1_name}<br>☐ Babbar Kawa ta

	2:${friend2_name}<br>☐ Kawa/aboki ta/na dabam<br>☐ Wani abu daban<br>☐ Ban sani ba<br>☐ Babu amsa<br>(${friend1_name} != '' and ${friend1_name} != '-99' and filter = 'friend1') or (${friend2_name} != '' and ${friend2_name} != '-99' and filter = 'friend2') or (filter = 'always')
722b. Bayan hakan ta faru gare ki, shin ko kin tabayin wani abu domin al'adarki ta dawo lokacin da kika samu damuwa akan kin samu ciki?<br>*Probe to confirm whether the period regulation was successful. If not, select 'no.'*	${self_abt_yn} = 'yes'<br>○ E<br>○ Aa<br>○ Babu amsa
722b. Shin ko kin tabayin wani abu domin al'adarki ta dawo lokacin da kika samu damuwa akan kin samu ciki?<br>*Probe to confirm whether the period regulation was successful. If not, select 'no.'*	${self_abt_yn} != 'yes'<br>○ E<br>○ Aa<br>○ Babu amsa
723b. A wace shekara wannan ya faru?<br>*Idan haka ya faru fiye da sau daya, baiyana lokaci na baya baya da hakan ya faru.*<br>*Shigar da 2020 idan ba'a sani ba ko kuma ba'a bada amsa ba.'.*	(${self_reg_yn} = 'yes')<br>Year: ___________
724b. Shin ko kinyi abubuwa fiye da daya domin cikin ya wanku, wato cikin ya zube?	((${self_reg_year} > ${self_abt_year}) or (${self_abt_year} = '')) and ((${self_reg_yn} = 'yes'))<br>○ E<br>○ Aa<br>○ Babu amsa
725b. Wane abu kika fara yi?	((${self_reg_year} > ${self_abt_year}) or (${self_abt_year} = '')) and ((${self_reg_mult_yn} = 'ye …<br>○ Aikin tiyata<br>○ Kwayar magani da ake kira mifepristone or misoprostol<br>○ Kwayar magani da kike sha lokacin da kikeyin zazzabi, kamar antibiotics ko kuma maganin zazzabin malaria<br>○ Maganin hana daukar ciki na gaggawa<br>○ Kwayar magunguna, da ba'a san irinsu ba<br>○ Allura<br>○ Magungunna ko dabarun gargajiya, kamar tsirrai<br>○ Giya<br>○ Gishiri, maggi, or kanwa<br>○ Lemon tsami<br>○ Maganin tari<br>○ Shigar da wani abu cikin farji<br>○ Wani abu daban<br>○ Ban sani ba<br>○ Babu amsa
725b. Wane abu kika yi?	((${self_reg_year} > ${self_abt_year}) or (${self_abt_year} = '')) and ((${self_reg_mult_yn} = 'no …<br>○ Aikin tiyata<br>○ Kwayar magani da ake kira mifepristone or misoprostol<br>○ Kwayar magani da kike sha lokacin da kikeyin zazzabi, kamar antibiotics ko kuma maganin zazzabin malaria<br>○ Maganin hana daukar ciki na gaggawa

	gaggawa<br>Kwayar magunguna, da ba'a san irinsu ba<br>Allura<br>Magungunna ko dabarun gargajiya, kamar tsirrai<br>Giya<br>Gishiri, maggi, or kanwa<br>Lemon tsami<br>Maganin tari<br>Shigar da wani abu cikin farji<br>Wani abu daban<br>Ban sani ba<br>Babu amsa
726b. Wane wuri kika je akayi miki aiki?	((${self_reg_year} > ${self_abt_year}) or (${self_abt_year} = '')) and ((${self_reg_first} = 'surg …<br>Asibitin gwamnati<br>Cibiyar lafiya ta gwamnati<br>Karamin asibitin samar da dabarun dakatar da ko hana daukar ciki<br>Asibitin tafi da gidan ka (na gwamnati)<br>TBA/Fieldworker (public)<br>Asibitin kudi/kilinik<br>Wajen bayarda magani<br>Kemis / Babban shagon magani<br>Likita ko nurse masu zaman kansu<br>Asibitin tafi da gidan ka (me zaman kasan)<br>Unguwar zoma (masu zaman kan su)<br>Shago<br>Kungiyar addini/Coci<br>Aboki/dan uwa ko yar uwa<br>NGO<br>Kasuwa/ 'Yan talla<br>Wani abu daban<br>Ban sani ba<br>Babu amsa
727b. Wane wuri kika je akayi miki aiki?	((${self_reg_year} > ${self_abt_year}) or (${self_abt_year} = '')) and ((${self_reg_first} = 'pill …<br>Asibitin gwamnati<br>Cibiyar lafiya ta gwamnati<br>Karamin asibitin samar da dabarun dakatar da ko hana daukar ciki<br>Asibitin tafi da gidan ka (na gwamnati)<br>TBA/Fieldworker (public)<br>Asibitin kudi/kilinik<br>Wajen bayarda magani<br>Kemis / Babban shagon magani<br>Likita ko nurse masu zaman kansu<br>Asibitin tafi da gidan ka (me zaman kasan)<br>Unguwar zoma (masu zaman kan su)<br>Shago<br>Kungiyar addini/Coci

	Aboki/dan uwa ko yar uwa<br>NGO<br>Kasuwa/ 'Yan talla<br>Wani abu daban<br>Ban sani ba<br>Babu amsa
728b. Wane abu kikayi a karshe?	((${self_reg_year} > ${self_abt_year}) or (${self_abt_year} = '')) and ((${self_reg_mult_yn} = 'ye …<br>Aikin tiyata<br>Kwayar magani da ake kira mifepristone or misoprostol<br>Kwayar magani da kike sha lokacin da kikeyin zazzabi, kamar antibiotics ko kuma maganin zazzabin malaria<br>Maganin hana daukar ciki na gaggawa<br>Kwayar magunguna, da ba'a san irinsu ba<br>Allura<br>Magungunna ko dabarun gargajiya, kamar tsirrai<br>Giya<br>Gishiri, maggi, or kanwa<br>Lemon tsami<br>Maganin tari<br>Shigar da wani abu cikin farji<br>Wani abu daban<br>Ban sani ba<br>Babu amsa
729b. Wane wuri kika je akayi miki aiki?	((${self_reg_year} > ${self_abt_year}) or (${self_abt_year} = '')) and (${self_reg_last} = 'surger …<br>Asibitin gwamnati<br>Cibiyar lafiya ta gwamnati<br>Karamin asibitin samar da dabarun dakatar da ko hana daukar ciki<br>Asibitin tafi da gidan ka (na gwamnati)<br>TBA/Fieldworker (public)<br>Asibitin kudi/kilinik<br>Wajen bayarda magani<br>Kemis / Babban shagon magani<br>Likita ko nurse masu zaman kansu<br>Asibitin tafi da gidan ka (me zaman kasan)<br>Unguwar zoma (masu zaman kan su)<br>Shago<br>Kungiyar addini/Coci<br>Aboki/dan uwa ko yar uwa<br>NGO<br>Kasuwa/ 'Yan talla<br>Wani abu daban<br>Ban sani ba<br>Babu amsa
730b. Wane wuri kika je kika samu magunguna?	((${self_reg_year} > ${self_abt_year}) or (${self_abt_year} = '')) and ((${self_reg_last} = 'pills …<br>Asibitin gwamnati<br>Cibiyar lafiya ta gwamnati

	○ Karamin asibitin samar da dabarun dakatar da ko hana daukar ciki<br>○ Asibitin tafi da gidan ka (na gwamnati)<br>○ TBA/Fieldworker (public)<br>○ Asibitin kudi/kilinik<br>○ Wajen bayarda magani<br>○ Kemis / Babban shagon magani<br>○ Likita ko nurse masu zaman kansu<br>○ Asibitin tafi da gidan ka (me zaman kasan)<br>○ Unguwar zoma (masu zaman kan su)<br>○ Shago<br>○ Kungiyar addini/Coci<br>○ Aboki/dan uwa ko yar uwa<br>○ NGO<br>○ Kasuwa/ 'Yan talla<br>○ Wani abu daban<br>○ Ban sani ba<br>○ Babu amsa
731b. Shin ko kin samu wata matsala kuma kikaje asibiti domin samun kulawa a lokacin zubar da cikin?<br>*If the respondent already reported she went to a health facility in the process of regulating her period, we are interested in whether she went back to a health facility on a separate occasion to treat complications that she may have experienced.*	((${self_reg_year} > ${self_abt_year}) or (${self_abt_year} = '')) and ((${self_reg_yn} = 'yes'))<br>○ E<br>○ Aa<br>○ Ban sani ba<br>○ Babu amsa
732b. Aane wuri kika je akayi miki aiki?<br>*Kada a karanta zabi a baiyane. A rubuta dukkan zabi da aka baiyana.*	((${self_reg_year} > ${self_abt_year}) or (${self_abt_year} = '')) and ((${self_reg_yn} = 'yes'))<br>☐ Miji/abokin zama<br>☐ Yar'uwa<br>☐ Dan'uwa<br>☐ Mahafiya<br>☐ Uba<br>☐ Dangi na dabam<br>☐ Babbar Kawa ta 1:${friend1_name}<br>☐ Babbar Kawa ta 2:${friend2_name}<br>☐ Kawa/aboki ta/na dabam<br>☐ Wani abu daban<br>☐ Ban sani ba<br>☐ Babu amsa<br>(${friend1_name} != '' and ${friend1_name} != '-99' and filter = 'friend1') or (${friend2_name} != '' and ${friend2_name} != '-99' and filter = 'friend2') or (filter = 'always')

	${consent_obtained}
Yanzu kuma zanyi miki wasu tambayoyi na gama gari a game da zubar da ciki, da kuma ta yadda ake kallon matan da suka zubar da ciki. Baiyana mini amsoshinki ta hanyoyi kamar haka: amincewa mai karfi, amincewa, ba amincewa ba rashin amincewa, rashin amincewa, karkarfar rashin amincewa.<br>*Bincika akwati a koma kasa.*	
Pinye OK wee bido	○ ko

733. Babu illa idan mace ta zubar da cikinta matukar zai saka ta cikin matsalar rashin lafiya.	${consent_obtained}<br>○ Karfin yarda<br>○ Yarda<br>○ Babu amincewa babu rashin amincewa

	○ Ba daidai ba<br>○ Karɓa sosai<br>○ Babu amsa
734. Babu illa idan mace ta zubar da cikin da ta samu ta hanyar fyade	${consent_obtained}<br>○ Karfin yarda<br>○ Yarda<br>○ Babu amincewa babu rashin amincewa<br>○ Ba daidai ba<br>○ Karɓa sosai<br>○ Babu amsa
735. Babu illa idan mace ta zubar da cikinta domin ba zata iya daukar nauyin kula da wani/ta yaron/yarinyar ba.	${consent_obtained}<br>○ Karfin yarda<br>○ Yarda<br>○ Babu amincewa babu rashin amincewa<br>○ Ba daidai ba<br>○ Karɓa sosai<br>○ Babu amsa
736. Babu illa idan mace ta zubar da cikinta domin bata da bukatar ta sake haihuwa.	${consent_obtained}<br>○ Karfin yarda<br>○ Yarda<br>○ Babu amincewa babu rashin amincewa<br>○ Ba daidai ba<br>○ Karɓa sosai<br>○ Babu amsa
737. Matar da zubar da ciki ta jawowa iyalanta abun kunya.	${consent_obtained}<br>○ Karfin yarda<br>○ Yarda<br>○ Babu amincewa babu rashin amincewa<br>○ Ba daidai ba<br>○ Karɓa sosai<br>○ Babu amsa
738. Matar da ta zubar da ciki kada ta baiyanawa kowa.	${consent_obtained}<br>○ Karfin yarda<br>○ Yarda<br>○ Babu amincewa babu rashin amincewa<br>○ Ba daidai ba<br>○ Karɓa sosai<br>○ Babu amsa

Baiyanawar amincewa domin amsa tambayoyi a gaba

FLW_801. Mungode da bamu lokaci ki amsa tambayoyinmu.	(${self_abt_yn} = 'yes') or (${self_reg_yn} = 'yes')<br>○ E<br>○ Aa<br>○ Babu amsa
FLW_802. Shin ko kin mallaki waya?	${flw_willing} = 'yes'<br>○ E<br>○ Aa<br>○ Babu amsa
FLW_803. Shin ko zaki iya bani lambar wayar da akafi kiranki da ita?<br>*Shigar da lamba mai alkaluma 11 ba tare da rubuta lambar kasa ba. Kada a bar tsakani ko digo. Shigar da 0 idan ba'a bada amsa ba.*	${flw_number_yn} = 'yes'<br>--
FLW_804. Zakaiya mai maita wan na numba kuma?<br>*Shigar da lamba mai alkaluma 11 ba tare da rubuta lambar kasa ba.*	(${flw_number_typed} != '') and (${flw_number_typed} != '0')

Kada a bar tsakani ko digo. Shigar da 0 idan ba'a bada amsa ba.	
Thank the respondent for her time.<br>*The respondent is finished, but there are still more questions for you to complete outside the home.*	${available} = 'yes'

Location and Questionnaire result

095. Location<br>*Take a GPS point near the entrance to the household. Record location when the accuracy is smaller than 6 m.*	Ko da yaushe
096. How many times have you visited this household to interview this female respondent?	Ko da yaushe<br>○ Karo na farko<br>○ Lokaci na biyu<br>○ Lokaci na uku
097. In what language was this interview conducted?	009a = 1<br>○ Turanci<br>○ Hausa<br>○ Igbo<br>○ Yoruba<br>○ Pidgin<br>○ Wani abu daban
