# Supplementary material for: Measurement of abortion safety using community-based surveys: Findings from three countries
Source: PLoS One. 2019 Nov 7;14(11):e0223146. doi: 10.1371/journal.pone.0223146 (PMC6837422; doi:10.1371/journal.pone.0223146)
Supplement: S3 Doc — (PDF) [file pone.0223146.s003.pdf]

S3 Doc.

NGR5-Female-Questionnaire-v36-aso.xlsx

001a. Ị nọ n'ezi ezinụlọ? EA: [EA entered in the Household Questionnaire] Structure #: [Structure entered in the Household Questionnaire] Household #: [Household entered in the Household Questionnaire]	Mgbe niile<br>○ E-e<br>○ Mba
002. Debanye aha gi ebe a.<br>*Biko detuo ahagi*	002 = 0<br>--
003b. Detue nke bu ezi ubochi na oge	003 = 0<br>Day: ----------------------------------<br>Month: ----------------------------------<br>Year: ----------------------------------

Ozi ndị a sitere n'aka ajụjụ ọnụụlọ. Biko nyochaa iji jide n'aka na ị na-agba ajụjụ banyere onye ziri ezi. [ODK will display the State, LGA, Enumeration Area, Structure Number, and Household Number entered into the Household Questionnaire linked to this Female Questionnaire.] Is the above information correct?	Mgbe niile
State: ${level1_unlinked}	State: [STATE]<br>--
LGA: ${level2_unlinked}	LGA: [LGA]<br>--
Ogbe: ${level3_unlinked}	Locality: [LOCALITY]<br>--
Enumeration Area: [EA]	--
Structure number: [#]	--
Nọmba ụlọ: [#]	--
004b. Ama din a elu o bu ezi okwu?	004 = 0<br>○ E-e<br>○ Mba

005. CHECK: Ị ga-anwa ịza ajụjụ ọnụ [Name of the interviewee]. Is that correct?<br>*If misspelled, select “yes” and update the name in question “011.” If this is the wrong person, you have two options: (1) exit and ignore changes to this form. Open the correct form.*<br>*Or (2) find and interview the person whose name appears above.*	Mgbe niile<br>○ E-e<br>○ Mba
006. Onye aziza o no n auto ma dikwa na njikere iza ajuju taa?	Mgbe niile<br>○ E-e<br>○ Mba
007. I mara onye aziza ajuju a nke oma?	006 = 1<br>○ A mara m ya nke ukwu<br>○ A mara m ya nke oma<br>○ A machaghi m ya nke oma<br>○ A maghi m ya ma oli
008. Onye oziza omebugo PMA 2020 mgbe gara aga?	006 = 1<br>○ E-e<br>○ Mba<br>○ Amaghi m<br>○ Onweghi aziza
UBURU BU<br>*chota nwanyi ahu nke gbara afo iri na ise rue afo iri a no na iteghete (15-49) metutara Akwukwo Ajuju umu nwanyi a. Iju ajuju a ga-enweriri ihe mkpuchite nke onwe. Gua ekele ndia:*	(${available} = 'yes') and (not(${unlinked}) or ${proceed_with_unlinked})
Ndewo. A ha m bu______________________________ a na-aru oru na ulo oru nchoputa Etiti, Evaluation Resources na Development na ulo akwukwo Mahadum nke Bayero din a Kano. Anyi na-eme nyocha o din ala nke na ajuju umu nwanyi ajuju banyere otutu ihe gbasara ahuike nke imu nwa. O ga-amasi anyi	(${available} = 'yes') and (not(${unlinked}) or ${proceed_with_unlinked})

ma o buru na i ga-esonye na nyocha a. Ama nke a ga-enyere aka ituputara goomenti aro iji mee ka ha nwee ezigbo nkwalite nke nlete ahuike, Nyocha a na-ewe ihe di na agbata nkeji iri na ise na iri abua iji kwusi. Anyi ga-echekwa ama obula i ga-aroputara anyi nakwa a gaghi egosi ya onye obula karikwa ndi otu anyi na eme nyocha a.<br>Isonye na nyocha a abughi ihe a na-amanye amanye, o buru na abia na ajuju nke i na-achoghi iza, me ka m mara, ka m gaa na ajuju ozo. Ka o bu inwere ike ikwusi iju ajuju mgbe obula ichoro. Kama, anyi nwere akwukwe na i ga-esonye na nyocha nihi na ebum na uche gi di anyi mkpa.<br>Ugbu a inwere ihe obula i ga-aju m banyere nyocha a?	
009a. Enwere m ike i bido iju ajuju ugbu a?	(${available} = 'yes') and (not(${unlinked}) or ${proceed_with_unlinked})<br>○ E-e<br>○ Mba
010. Interviewer's name Biko dekọọ aha gị dịka onye akaebe na usoro nkwenye. Ị banyere na mbụ "[Interviewer's name]."	${consent_obtained} and (${your_name_check} = 'no')<br>____

Section 1 – Respondent's Background, Marital Status, Household characteristics

U gbua o ga-amasi m iju gi onye ibu na onodu obibi gi.

	${consent_obtained}
101. Kedu ọnwa na afọ a mụrụ gị? Oge ọ dị na ndepụta ụlọ bụ [AGE]<br>*Select 'Do not know' for month and '2020' for year to indicate 'No Response'.*	009a = 1
101. Kedu nimie onwa na afo a muru gi?<br>*Select 'Do not know' for month and '2020' for year to indicate 'No Response'.*	009a = 1
Ọnwa:	○ Jenụwarị<br>○ February<br>○ eme njem<br>○ April<br>○ Ike<br>○ June<br>○ July<br>○ August<br>○ Septemba<br>○ October<br>○ November<br>○ December<br>○ Amaghi m
Afo:	Year: ________________________________

102. Afo ole ka i di oge ikpe-azu imere ememe ncheta omumu gi?	009a = 1<br>____
103. Kedu ụlọ akwụkwọ kachasị elu ị gara?<br>*Denye soso ihe iguta na ulo akwukwo. E denye kwana bibulu mor ulo akwukwo koran mor obere akukwo.*	009a = 1<br>○ A gaghi m.<br>○ Paramari<br>○ Sekondiri<br>○ Nkedi elu<br>○ Onweghi aziza
104. I luola di ka o bu o nwere nwoke gin a ya bi ugbua?<br>*Juo: O buru mba, juo ma onye aziza o gbara alukwaghi m, o kewara ya na di ya ka o bu di ya nwuru anwu.*	009a = 1<br>○ E-e, aluola m di ugbua<br>○ E-e, mu na nwoke bi<br>○ Mba: enweghi m nnoko ugbua: Agbaram alukwaghim/anyi kewara ekewa

	◌ Anoghi na nnoko ugbua: Onye di ya nwuru<br>◌ Mba, enweghi nmeko<br>◌ Onweghi aziza
105. I lutula di ka o bu gin a nwoke o bitula otu ugboro ka o bu karia otu ugboro?	104 ≠ 5<br>◌ Nani otu ugboro<br>◌ Karia otu ugboro<br>◌ Onweghi aziza

	(${marriage_history} = 'more_than_once')
106a. Kedu nime onwa ka obu afo ibidoro ibi gi na di gi nke mbu ka o bu onye agbata obi gi?<br>*Select 'Do not know' for month and '2020' for year to indicate 'No Response'.*	105=2
Ọnwa:	◌ Jenụwarị<br>◌ February<br>◌ eme njem<br>◌ April<br>◌ Ike<br>◌ June<br>◌ July<br>◌ August<br>◌ Septemba<br>◌ October<br>◌ November<br>◌ December<br>◌ Amaghi m
Afo:	Year: ____________________

106b. CHOPUTA: Site na ihe onye ijuru ajuju denye na 106b, onye ijuru ajuju odi afor 15 kor na orubeghi mgbe oluru di izizi<br>Ị banyere 106a n'ụzọ ziri ezi?	106a afọ na alụmdi na nwunye. ≤15<br>◌ E-e<br>◌ Mba

	(${marriage_history} = 'once') or (${marriage_history} = 'more_than_once')
107a. Ugbu a o ga-amasi m iju gi banyere mgbe gi na di I na-alu ugbu a ka o bu onye agbata-obi nke inwere na nso nso a bidoro biwe. Kedu nime onwa ma o bu afo o bu?<br>*Select 'Do not know' for month and '2020' for year to indicate 'No Response'.*	105 = 1 or 2
Ọnwa:	◌ Jenụwarị<br>◌ February<br>◌ eme njem<br>◌ April<br>◌ Ike<br>◌ June<br>◌ July<br>◌ August<br>◌ Septemba<br>◌ October<br>◌ November<br>◌ December<br>◌ Amaghi m
Afo:	Year: ____________________

107b. CHOPUTA: Site na ihe asara gi e denye na 107a, onye ijuru ajuju nwe ike di ofor 15 ma obukwa na orughi ugbua ono na di. E denye kwa 107a ofuma?	107a afọ na alụmdi na nwunye ≤15<br>◌ E-e<br>◌ Mba
108. Di gi ka o bu onye agbata-obi gi o nwere nwunye ndi ozo ka o bu yana umu nwanyi ndi ozo bi di ndi na alu onwe ha?	104 = 1 or 2<br>◌ E-e<br>◌ Mba<br>◌ Amaghi m<br>◌ Onweghi aziza

Agba nke Abua – Imu nwa, Idi ime na izu oke na aru nile

Ugbu a o ga– amasi m iju maka umu nile nke imurula na ndu gi.

200. Ugbua achorom kam juagi ajuju gbasara umu nile imutara na ndu gi? E muola nwa izizi?	009a = 1<br>○ E-e<br>○ Mba<br>○ Onweghi aziza
201. How many times have you given birth?<br>*Denye -99 na nkiti*	200 = 1<br>________________

	(${birth_events} > 1)
205. Kedu mgbe imuru nwa mbu gi?<br>*Biko detuo ụbọchị nke ọmụmụ nwa. A ga-achọta ụbọchị ahụ site na ịkọ azụ site na ihe omume na-echefu echefu ma ọ bụrụ na ọ dị mkpa.*<br>*Select 'Do not know' for month and '2020' for year to indicate 'No Response'.*	201 > 1
Ọnwa:	○ Jenụwarị<br>○ February<br>○ eme njem<br>○ April<br>○ Ike<br>○ June<br>○ July<br>○ August<br>○ Septemba<br>○ October<br>○ November<br>○ December<br>○ Amaghi m
Afo:	Year: ________________

	(${birth_events} > 0)
206. Kedu mgbe imuru nwa na nso nso a?<br>*Select 'Do not know' for month and '2020' for year to indicate 'No Response'.*	201 > 1
Ọnwa:	○ Jenụwarị<br>○ February<br>○ eme njem<br>○ April<br>○ Ike<br>○ June<br>○ July<br>○ August<br>○ Septemba<br>○ October<br>○ November<br>○ December<br>○ Amaghi m
Afo:	Year: ________________

210a. I di ime ugbu a?	${consent_obtained}<br>○ E-e<br>○ Mba<br>○ Amaghim<br>○ Onweghi aziza

	${pregnant} = 'yes'
210b. I di ime onwa ole? Nwa imuru na nso nso a bu i [Ubochi imuru nwa na nso nso a]	210a = 1
Ọmụmụ kachasị nso bụ: [Date of most recent birth]	${recent_birth} != ''
#####<br>*Biko denye ogugu onwa zuru ezu. Denye -88 na imaghi, -99 na onweghi aziza.*	________________

209. Kedu oge ihu flawa ikpe azu go jiri bido?<br>*O buru na ighoro ubochi, izu uka onwa ka o bu afo, i ga etinye onu ogugu maka x na onyonyo nke na abianu.*	009a = 1<br>◌ X ubochi gara aga<br>◌ X izu uka gara aga<br>◌ X Onwa gara aga<br>◌ X afo gara aga<br>◌ Flowa adia gbazi/Nwepu akpa nwa.<br>◌ Tupu nwa ikpeazu<br>◌ Ahutubeghim flawa<br>◌ Onweghi aziza
209a. Tinye [Oge mkpiri afọ]<br>*Denye 0 na ubochi ta, obugho 0 na izu uka/onwa/afor.*	(${menstrual_period} = 'days') or (${menstrual_period} = 'weeks') or (${menstrual_period} = 'month …<br>--

	${ever_birth} = 'yes' or ${pregnant} = 'yes'
213a. Ugbua o ga-amasi m iju ga ajuju maka nwa nka ikpe azu imuru.	201 > 0 AND 210a ≠ 1 201 > 0 AND 210a ≠ 1 201 > 0 AND 210a ≠ 1
213b. Ugbu a o ga-amasi m iju gi ajuju maka ime nke i di ugbua.	210a = 1
Ngbe ahu idi ime, echoburu idi ime ngbe ahu, echoburu echetu ka odutu, kor bu na echoburu imuta umu chaa chaa	(${birth_events} > 1 and ${pregnant} != 'yes') or (${ever_birth} = 'yes' and ${pregnant} = 'yes')
Ngbe ahu idi ime, echoburu idi ime ngbe ahu, echoburu echetu ka odutu, kor bu na echoburu imuta umu chaa chaa	(${birth_events} = 1 and ${pregnant} != 'yes') or ((${ever_birth} = 'no') and (${pregnant} = 'yes'))
#####	◌ Oge ahu<br>◌ Mgbe ozo<br>◌ Odiro chaa chaa<br>◌ Onweghi aziza

Ugbua ewerem ajuju bayere odin'iru.	${consent_obtained}
211a. I ga acho I mu nwa ka I ga ano amughi nwa	210a ≠ 1<br>◌ Muta nwa<br>◌ Ochoro omumu.<br>◌ Osi na Onweghi ike ituta ime.<br>◌ Akwadobem/Amaghim<br>◌ Onweghi aziza
211a. I ga acho I mu umu ndi ozo ka I muchaala?	210a ≠ 1<br>◌ Mata nwa ozo<br>◌ Nbe ana-eme onu ogugu<br>◌ Osi na Onweghi ike ituta ime.<br>◌ Akwadobem/Amaghim<br>◌ Onweghi aziza
211b. I muchaa nwa nkea I bu na afo, I ga acho I mu ozo ka I muchaala?	210a = 1<br>◌ Mata nwa ozo<br>◌ Nbe ana-eme onu ogugu<br>◌ Osi na Onweghi ike ituta ime.<br>◌ Akwadobem/Amaghim<br>◌ Onweghi aziza
212a. Lue ole mgbe ka I ga acho I cherekwu tupu I muta nwa?<br>*If you select months or years, you will enter a number for X on the next screen.*<br>*Select "Years" if more than 36 months.*<br>*Please check that you correctly entered the value for months/years.*	211a = 1<br>◌ X Onwa<br>◌ X afo<br>◌ Ugbu ugbua<br>◌ Osi na Onweghi ike ituta ime.<br>◌ Ndi ozo<br>◌ Amaghi m<br>◌ Onweghi aziza
212b. o buru na I muchaa nwa nkea ibu na afo, kedu oge I na ele anya imu nwa ozo?<br>*If you select months or years, you will enter a number for X on the next screen.*<br>*Select "Years" if more than 36 months.*<br>*Please check that you correctly entered the value for months/years.*	211b = 1<br>◌ X Onwa<br>◌ X afo<br>◌ Ugbu ugbua<br>◌ Osi na Onweghi ike ituta ime.

	◯ Ndi ozo<br>◯ Amaghi m<br>◯ Onweghi aziza
212c. Enter the number of [Months OR Years] you would like to wait:	${wait_birth_none} = 'months' or ${wait_birth_some} = 'months' or ${wait_birth_pregnant} = 'mont …<br>--

Section 3 – Nkwekọrịta

Ugbu a, m ga-achọ ikwu banyere atụmatụ ezinụlọ - ụzọ dị iche iche ma ọ bụ ụzọ dị iche iche di na nwunye nwere ike isi mee ka igbu oge ma ọ bụ zere ime.

Ihe oyiyi ga-apụta na ihuenyo maka ụzọ ụfọdụ. Ọ bụrụ na onye ahụ na-aza ajụjụ kwuru na ọ nụbeghị ụzọ ahụ maọbụ ọ bụrụ na ọ na-achọ ịza ajụjụ, gụọ ihe nyocha ahụ ma gosi ya onyinyo ahụ, ọ bụrụ na ọ dị.

301a. Inutula maka itughari ikpu um nwanyi? PROBE: Ana eme ndi nwanyi opareshion iji gbochie ka di ha ituwa ha ime.<br>CHOPUTA: Umu nwanyi nwe ike ime opareshion maka ihusi omumu.	009a = 1<br>◯ E-e<br>◯ Mba<br>◯ Onweghi aziza
301b. Inutula maka itughari amu umu oke? JUO NKE OMA: A na-eme ndi nwoke opareshion iji gbohie ha ituwa nwanyi ime	009a = 1<br>◯ E-e<br>◯ Mba<br>◯ Onweghi aziza
301c. Inutula maka igbakwunye ihe mgbochi afo ime nime ahu? JUO NKE OMA: U mu nwanyi nwere otu ma o bu otutu umu igwentakiri nke ndia-dokinta ma o bu ndi noosu na-agbakwu nye ha na aka nke ga-egohi ha ituru ime na ihe dika otu afo ma o bu karia.<br>[implant_150x300.png]	009a = 1<br>◯ E-e<br>◯ Mba<br>◯ Onweghi aziza
301d. Inutula maka IUD? JUO NKE OMA: Umu nwanyi na-enwe ihe dika nko ma o bu eriri nke ndi dokinta ma o bu noosu na-etinye ha nime ahu<br>[IUD_150x300.png]	009a = 1<br>◯ E-e<br>◯ Mba<br>◯ Onweghi aziza
301e. Inutula maka igba ntutu? JUA NKE OMA: Umu nwanyi na-enwe ntutu nke ndi na-enwe ntutu nke ndi na-enye nleta ahuike na-agba ha iji ikwusi ha ituru ime na ihe dika ohu onwa ma o bu karia<br>[sayana_depo_150x300.jpg]	009a = 1<br>◯ E-e<br>◯ Mba<br>◯ Onweghi aziza
301f. Inutula maka mkpuru ogwu eji egbochi omumu nwa? JUO NKE OMA: Umu nwanyi nwere mkpuru ogwu ha na-anu kwa ubochi iji gbochie ha ituru ime.<br>[pill_150x300.png]	009a = 1<br>◯ E-e<br>◯ Mba<br>◯ Onweghi aziza
301g. Ị nụtụla banyere ọgwụ mgbochi mberede?<br>MGBE ỤBỤDỤ: Dịka ihe mberede mgbe ụmụ nwanyị na-enweghi mmekọahụ na-enweghị nchebe nwere ike iri ọgwụ dị iche iche n'oge ọ bụla n'ime ụbọchị atọ na ise iji gbochie ime ime.	009a = 1<br>◯ E-e<br>◯ Mba<br>◯ Onweghi aziza
301h. Inutula maka kondomu? JUO NKE OMA: Umu nwoke na ekpunye ihe dika roba na amu ha tupu ha na nwanyi enwe nmeko.<br>[male_condom_150x300.png]	009a = 1<br>◯ E-e<br>◯ Mba<br>◯ Onweghi aziza
301i. Inutula maka kondomu nke umu nwanyi? JUO NKE OMA: Umu nwanyi na-etinyekwa ihe mkpuchi na-ebe ha si anu amiri tupu ha na nwoke enwe nmeko.<br>[female_condom_150x300.png]	009a = 1<br>◯ E-e<br>◯ Mba<br>◯ Onweghi aziza
301j. Inutula maka ihe ana akpo diaphragm? JUO NKE OMA: Ihe pere npe umu nwanyi na etinye na ime ikpu tupu hana nwoke emekota.<br>[diaphragm_150x300.png]	009a = 1<br>◯ E-e<br>◯ Mba<br>◯ Onweghi aziza
301k. Inutula maka ude ka o bu ofufu nke eji egbochi idi ime? JUO NKE OMA: Umu nwanyi nwere ike ite ude na ebe ha si anu amiri tupu ha na nwoke enwe nmeko iji gbochie idi ime.<br>[spermicide_150x300.png]	009a = 1<br>◯ E-e<br>◯ Mba<br>◯ Onweghi aziza

301l. Inutula maka usoro igu ubochi ka o bu mkpuru ola? JUO NKE OMA: Nwanyi nwere ike iji eriri mkpuru ola edere uri iji mara ubochi o nwere ike idi ime. Ubochi o mara na o ga-adi ime, ya na di ya nwere ike iji kondomu ma o bu ha agaghi enwe nmeko obula.
[SDM-beads_only.png]

009a = 1

- E-e
- Mba
- Onweghi aziza

301m. Inutula maka usoro nke LAM?

009a = 1

- E-e
- Mba
- Onweghi aziza

301n. Inutula maka usoro nke iji ubochi ufodu na onwa? JUO NKE OMA: Umu nwanyi nwere ike igbochi idi ime site-na enweghi nmeko na ubochi ufodu nime onwa ha mara na ha di ime ma ha nwee nmeko

009a = 1

- E-e
- Mba
- Onweghi aziza

301o. Inutula maka usoro nke nmipu? JUO NKE OMA: Umu nwoke ga-akparahara anya nke oma ma mipu amu ha tupu ha erue klimasi..

009a = 1

- E-e
- Mba
- Onweghi aziza

301p. Inutula ma onwere uzo ozo ma o bu usoro ozo ndi nwoke ma nwanyi nwere ike iji wee gbochie afo ime?

009a = 1

- E-e
- Mba
- Onweghi aziza

302a. O nwere ihe gi na onye agbata-obi gi na enwe ugbua ka o bu usoro unu na-eji iji gbochie ma o bu cheretu ituru ime?

210a ≠ 1 AND 009a = 1 210210a ≠ 1 AND 009a = 1 210a ≠ 1 AND 009a = 1 ≠ 1 AND 009a = 1 210a ≠ 1 AND 009a = 1

- E-e
- Mba
- Onweghi aziza

${current_user} = 'yes'

302b. Kedu usoro ka o bu ufodu usoro ndi u nu na eji? Jua nke oma: Onwere ihe ozo?

Hoputa usoro nile ndi akporo aha. Kpachara anya nke oma ma hu na igosiputara usoro nile.

302a = 1

- Itughari akpa nwa nwanyi
- Itughari amu umu nwoke
- Igbakwu nye ihe nime aru
- IUD
- Igba ntutu
- Mkpuru ogwu
- Mgbochi mberede
- Kondomu umu nwoke
- Kondomu umu nwanyi
- Dayaframu
- Ude/Ofufu
- Isoro ubochi/Mkpuru ola
- LAM
- Iri ubochi ufodu na onwa
- Nmipu
- Usoro odinala ndi ozo
- Onweghi aziza

CALC_CM. CALCULATE: CURRENT METHOD

THIS WILL NOT APPEAR ON THE SCREEN

ODK ga-achọpụta usoro kachasị dị irè nke onye na-aza ajụjụ na-eji ya eme ihe site na ịhọrọ usoro kachasị elu na listi nhọrọ

302a=1 AND 302b ≠-99

- Itughari akpa nwa nwanyi
- Itughari amu umu nwoke
- Igbakwu nye ihe nime aru
- IUD
- Igba ntutu
- Mkpuru ogwu
- Mgbochi mberede
- Kondomu umu nwoke
- Kondomu umu nwanyi
- Dayaframu
- Ude/Ofufu
- Isoro ubochi/Mkpuru ola
- LAM
- Iri ubochi ufodu na onwa

	○ Nmipu<br>○ Usoro odinala ndi ozo<br>○ Onweghi aziza
LCL_301. JUO NKE OMA: Ntutu agbara ya eji onu ntutu kor obere ntutu<br>*Gosi onye aziza foto ahu*<br>[sayana_depo_150x300.jpg]	CALC CM = 5<br>○ Ntutu<br>○ Obele ntutu (sayana press)<br>○ Onweghi Aziza
302c. Di gị ma ọ bụ onye òtù ọlụlụ gị maara na ị na-eji [ECHICHE MERE]?	302a = 1<br>○ E-e<br>○ Mba<br>○ Onweghi aziza
302c. Di gị ma ọ bụ onye òtù ọlụlụ gị maara na ị na-eji atụmatụ ezinụlọ?	302a = -99<br>○ E-e<br>○ Mba<br>○ Onweghi aziza
303. Onye roputara usoro a ogwara gin a onye agbata-obi gi na usoro na-adi na nnagide?	302b = male or female sterilization<br>○ E-e<br>○ Mba<br>○ Onweghi aziza
305a. I kwuru na inaghi eji usoro mgbochi afo ime obula ugbua. I chere na I ga-eji usoro mgbochi afo ime obula na obia niru iji cheretu ka o bu gbochie idi ime?	302a ≠1 AND 210a ≠1<br>○ E-e<br>○ Mba<br>○ Onweghi aziza
305b. I chere na i ga eji usoro mgbochi afo ime mgbe obula na odini hu iji cheretu ka o bu gbochie idi ime?	302a ≠1 AND 201a = 1<br>○ E-e<br>○ Mba<br>○ Onweghi aziza
306a. Nime onwa iri na abua gara aga, onwetula ihe imere ka o bu usoro obula ijuturula iji cheretu ka o bu gbochie idi ime?	302a ≠1<br>○ E-e<br>○ Mba<br>○ Onweghi aziza

	${recent_user} = 'yes'
306b. Kedu usoro ijiri na nso nso a? JUO: Onwere ihe ozo<br>*Horo usoro nke kacha nma (nke kachasi ego na listi) Gbadata ka ihu nhoro nile.*	306a = 1<br>○ Igbakwu nye ihe nime aru<br>○ IUD<br>○ Igba ntutu<br>○ Mkpuru ogwu<br>○ Mgbochi mberede<br>○ Kondomu umu nwoke<br>○ Kondomu umu nwanyi<br>○ Dayaframu<br>○ Ude/Ofufu<br>○ Isoro ubochi/Mkpuru ola<br>○ LAM<br>○ Iri ubochi ufodu na onwa<br>○ Nmipu<br>○ Usoro odinala ndi ozo<br>○ Onweghi aziza

LCL_302. JUO NKE OMA: Ntutu agbara ya eji onu ntutu kor obere ntutu<br>*Gosi onye aziza foto ahu*<br>[sayana_depo_150x300.jpg]	306b = 5<br>○ Ntutu<br>○ Obele ntutu (sayana press)<br>○ Onweghi Aziza
307. Tupu ị malite iji [CURRENT METHOD / MOST RECENT METHOD], ọ bụrụ na ị tụlere mkpebi ime oge ma ọ bụ zere inwe ime na di gị ma ọ bụ di gị?	302a = 1 OR 306a = 1<br>○ E-e<br>○ Mba<br>○ Amaghi m<br>○ Onweghi aziza
308. Ị ga - ekwu na iji ọgwụ mgbochi ime ihe bụ mkpebi gị, karịsịa mkpebi di gị na onye òtù ọlụlụ gị maọbụ ka gị abụọ kpebiri ọnụ?	302a = 1<br>○ Obukari onye aziza<br>○ Obukari di/enyi

	○ Nkwekota nmadu nile.<br>○ Ndi ozo<br>○ Onweghi aziza
TCI_302. Oburu na obughi Di gi/enyi , kedu onye ozo na si gi tinye usoro obula gbasara omumu?<br>JUO NKE OMA: onwere onye ozo?<br>*Aguputala usoro. Hoputa ndi nile daberenu.*	302a = 1 OR 306a = 1<br>☐ Nne<br>☐ Ogo nwayi<br>☐ Umu nwanne di nwanyi<br>☐ Ogo ndi nwanne nwanyi.<br>☐ Mama Ochie<br>☐ Ndi enyi<br>☐ Onye na elokota maka ahu ike<br>☐ Onyeisi obodo<br>☐ Onye ishi ndi uka<br>☐ Anti<br>☐ Umu nwanne gi ndi ozo<br>☐ Ndi ozo<br>☐ Nbe ana-eme onu ogugu<br>☐ Onweghi Aziza
TCI_302x. Na ime onwa iri na abuo gara aga,onwego ndi enyi gi/umu nwanne gi e gwara okwu gbasara njikota ezi na ulo?	302a = 1 OR 306a = 1<br>○ E-e<br>○ Mba<br>○ Amaghi m<br>○ Onweghi aziza
308a. Oge ikpeazụ ị nwetara gị [CURREN METHOD /MOST RECENT METHOD], ego ole ka ị ga-akwụ site n'akpa uwe, tinyere ụgwọ ọ bụla a kwụrụ maka usoro, ngwaahịa ma ọ bụ ọrụ, na njem?<br>*Tinye ihe niile na Naira. Zero bụ azịza dị mma. Tinye -88 ma ọ bụrụ na onye na-aza ajụjụ amaghị, -99 maka nzaghachi.*	302a = 1 OR 306a = 1<br>--

	${current_user} = 'yes'
309a. Ebe ọ bụ na ọnwa na afọ ka ị na-eji[CURRENT METHOD / MOST RECENT METHOD]enweghị nkwụsị?<br>*Gbakoo ofuma site na nme nme gara aga ma ichoo*	302a = 1
Most Recent Birth: [mm-yyyy]	${recent_birth} != ''
Di na Nwunye: [mm-yyyy]	${husband_cohabit_start_recent} != ''
Ọnwa:	○ Jenụwarị<br>○ February<br>○ eme njem<br>○ April<br>○ Ike<br>○ June<br>○ July<br>○ August<br>○ Septemba<br>○ October<br>○ November<br>○ December<br>○ Amaghi m
Afo:	Year: ------------------------------------

	${recent_user} = 'yes'
309b. Kedu mgbe ị kwụsịrị iji [CURRENT METHOD / MOST RECENT METHOD]?<br>*Biko detuo ụbọchị. A ga-achọta ụbọchị ahụ site na ịkọ azụ site na ihe omume na-echefu echefu ma ọ bụrụ na ọ dị mkpa.*<br>*Select 'Do not know' for month and '2020' for year to indicate No Response.*	306a = 1
Ọnwa:	○ Jenụwarị<br>○ February<br>○ eme njem<br>○ April<br>○ Ike

	◯ June<br>◯ July<br>◯ August<br>◯ Septemba<br>◯ October<br>◯ November<br>◯ December<br>◯ Amaghi m
Afo:	Year:

	${recent_user} = 'yes'
309c. Kedu ọnwa na afọ ị malitere iji [CURRENT METHOD / MOST RECENT METHOD]tupu ịkwụsị?<br>*Detuo azụ site na ihe ndi nwere ncheta ma oburu na choro.*<br>*Họrọ 'Amaghị' maka ọnwa na '2020' maka afọ iji gosi Nzaghachi.*	306a = 1
Most Recent Birth: [mm-yyyy]	${recent_birth} != ''
Di na Nwunye: [mm-yyyy]	${husband_cohabit_start_recent} != ''
Ọnwa:	◯ Jenụwarị<br>◯ February<br>◯ eme njem<br>◯ April<br>◯ Ike<br>◯ June<br>◯ July<br>◯ August<br>◯ Septemba<br>◯ October<br>◯ November<br>◯ December<br>◯ Amaghi m
Afo:	Year:

309d. CHECK:Naanị ijide n'aka na m nwere nke a ziri ezi, ị jiri ya[CURRENT METHOD / MOST RECENT METHOD] nọgidere n'etiti [START DATE] and [END DATE] enweghị nkwụsị, ọ bụ nke ahụ ziri ezi?	306a = 1<br>◯ E-e<br>◯ Mba
Nachie azu na nke igafe go ka ijuputa ofuma ngbe obido na nso nso we sobe usoro<br>*Ajuju ndi ozo: -Kedu oge ozo gara aga e tinyelu (USORO}? -Kedu ngbe e tinye [USORO] na akusighi akwusi.*	309d = 0
310. Gịnị mere ị kwụsịrị iji[CURRENT METHOD / MOST RECENT METHOD?	306a = 1<br>☐ Enweghi nmeka mgbe nile/di ya anoghi na ulo<br>☐ Odiri ime mgbe o na ejiya<br>☐ Ochoro idi ime<br>☐ Di/Enyi akwadoghi ya<br>☐ Ochoro usoro nke kachasi nma<br>☐ Onweghi usoro nke di<br>☐ Ometutara ahuike<br>☐ Ujo ma oga-emeru ya ahu<br>☐ Onweghi ka a ga-esi nweta ya/ebe odi di anya<br>☐ Odi oke onu<br>☐ O maghi otu osi eji ya<br>☐ O na-emeru ahu<br>☐ O siri ike ituta ime<br>☐ O na-agbakosi odidi aku madu<br>☐ Ndi ozo<br>☐ Amaghi m<br>☐ Onweghi aziza

	${current_or_recent_user} and (${current_recent_method} != 'LAM') and

311a. I bu ụzọ malite iji [CURRENT METHOD / MOST RECENT METHOD] on [DATE FROM FQ309a OR 309c] Ebee ka gị na onye òtù ọlụlụ gị nwetara ya n'oge ahụ?<br>*Gosiputa nhoro nile.*	($\{current_recent_method\} != (CALC_CM ≠ 14, 30, 31, 39, -99) OR (306b ≠ 14, 30, 31, 39, -99)<br>○ Ulonleta ahuike goomenti<br>○ Senta ahuike nke goomenti<br>○ Ulo nleta ntazi omu mu nwa<br>○ Ulo nleta mbughari<br>○ TBA/Fieldworker (public)<br>○ Ulo nleta ahuike nke onwe<br>○ Famasi<br>○ mmiri ogwu<br>○ Dokita onweya morbu nurse<br>○ Dokinta Onwe<br>○ TBA/Fieldworker (private)<br>○ Shopu<br>○ FBO/Church<br>○ Ndi enyi/Ndi ezi na ulo<br>○ NGO<br>○ Ahia<br>○ Ndi ozo<br>○ Amaghi m<br>○ Onweghi aziza
312a. Mgbe ị nwetara gị[CURRENT METHOD / MOST RECENT METHOD],ka onye na-enye gị gwara gị banyere nsogbu ndị ị ga - enwe ma ọ bụ nsogbu ị nwere ike ịnweta na iji kwụsị oge ma ọ bụ zere inwe ime?	311a ≠ .<br>○ E-e<br>○ Mba<br>○ Onweghi aziza
312b. Agwara gi ihe i ga-eme ma o buru na ichoputa nsogbu ma o bu ihe nmekata ahu obula?	312a = 1<br>○ E-e<br>○ Mba<br>○ Onweghi aziza
313. N'oge ahụ, ndị na-eme atụmatụ maka ezinụlọ na-agwa gị banyere usoro nke atụmatụ ezinụlọ karịa nke[CURRENT METHOD / MOST RECENT METHOD]nke i nwere ike iji?	311a ≠ . OR 311b ≠ .<br>○ E-e<br>○ Mba<br>○ Amaghi m<br>○ Onweghi aziza
314a. Mgbe i gara ebe ahu, i natara usoro mgbochi afo ime nke ichoro?	311a ≠ .<br>○ E-e<br>○ Mba<br>○ Onweghi aziza
314c. Gịnị mere na ị nwetaghị usoro ị chọrọ?	314a = 0<br>○ Usoro m choro adighi ya ubochi ahu<br>○ Usoro m choro adighi ma otu<br>○ Onye nweputa a zughi azu iweputa usoro<br>○ Onye nweputa gosiri usoro ozo di iche<br>○ Ezughi m oke iji usoro ahu<br>○ Emejibeghi m o bi m iji usoro ahu<br>○ O di oke onu<br>○ Ndi ozo<br>○ Onweghi aziza
315a. Mgbe i gara ebe ahu onye tutara aro maka usoro nke i ga-onwela?	311a ≠ .<br>○ Gi onwe gi nani<br>○ Onye nweputa<br>○ Enyi gi<br>○ Gi na onye na zu gi nri.<br>○ Gi na oryi gi<br>○ Ndi ozo<br>○ Amaghi m<br>○ Onweghi aziza

315b. Kedu onye siri noo soso nke ana akpo rhythm ka unu ga e tinye?	311b ≠ .<br>○ Gi onwe gi nani<br>○ Onye nweputa<br>○ Enyi gi<br>○ Gi na onye na zu gi nri.<br>○ Gi na oryi gi<br>○ Ndi ozo<br>○ Amaghi m<br>○ Onweghi aziza
315b. Kedu onye siri noo soso nke ana akpo Lam ka unu ge tinye?	311b ≠ .<br>○ Gi onwe gi nani<br>○ Onye nweputa<br>○ Enyi gi<br>○ Gi na onye na zu gi nri.<br>○ Gi na oryi gi<br>○ Ndi ozo<br>○ Amaghi m<br>○ Onweghi aziza
316. Ị ga-alaghachi na onye ọrụ a? Provider: [Type of provider selected in 311a or 311b]	311a ≠ 35 or 96<br>○ E-e<br>○ Mba<br>○ Amaghi m<br>○ Onweghi aziza
317. 317.Ị ga-akpọ onye ikwu gị ma ọ bụ enyi gị na onye na-eweta ọrụ a? Provider: [Type of provider selected in 311a or 311b]	311 a ≠ 34 or 96<br>○ E-e<br>○ Mba<br>○ Amaghi m<br>○ Onweghi aziza
SW_1a. Tupu ị malite iji [CURRENT METHOD / MOST RECENT METHOD] in [MOIS/ANNEE],ị na-eme ihe ma ọ bụ na-eji usoro ọ bụla iji kwụsị ma ọ bụ zere inwe ime?	302a = 1 OR 306a = 1<br>○ E-e<br>○ Mba<br>○ Onweghi aziza
SW_1b. Kedu usoro ị na-eji?	SW_1a = 1<br>○ Igbakwu nye ihe nime aru<br>○ IUD<br>○ Igba ntutu<br>○ Mkpuru ogwu<br>○ Mgbochi mberede<br>○ Kondomu umu nwoke<br>○ Kondomu umu nwanyi<br>○ Dayaframu<br>○ Ude/Ofufu<br>○ Isoro ubochi/Mkpuru ola<br>○ LAM<br>○ Iri ubochi ufodu na onwa<br>○ Nmipu<br>○ Usoro odinala ndi ozo<br>○ Onweghi aziza
PP_1. Since the birth of your child in [DATE OF MOST RECENT BIRTH], have you ever done something or used any method to delay or avoid getting pregnant?	child born in last 2 years AND 302a ≠ 1<br>○ E-e<br>○ Mba<br>○ Onweghi aziza
PP_2. Ogologo oge ole ka amuchara nwa [DATE OF MOST RECENT BIRTH] ị malitere ime ihe ma ọ bụ malite iji usoro?<br>*Tinye 0 ụbọchị maka taa. Ị ga-abanye nọmba maka X na ihuenyo ọzọ.*	PP_1 = 1 OR (302a = 1 AND child born in the last 2 years)<br>○ Mgbe ụbọchị X<br>○ Mgbe Izu X<br>○ Mgbe Ọnwa ole na ole X<br>○ Mgbe Afọ X<br>○ Onweghi aziza
PP_2. Enter [METHOD].	${pp_method_units} = 'days' or<br>${pp_method_units} = 'weeks' or

Ọ bụrụ na taa, tinye ụbọchị efu, ọ bụghị izu izu / ọnwa / afọ.	${pp_method_units} = 'months' or ${ … --
PP_3. Kedu usoro a?	PP_2 ≠ . ◯ Itughari akpa nwa nwanyi<br>◯ Itughari amu umu nwoke<br>◯ Igbakwu nye ihe nime aru<br>◯ IUD<br>◯ Igba ntutu<br>◯ Mkpuru ogwu<br>◯ Mgbochi mberede<br>◯ Kondomu umu nwoke<br>◯ Kondomu umu nwanyi<br>◯ Dayaframu<br>◯ Ude/Ofufu<br>◯ Isoro ubochi/Mkpuru ola<br>◯ LAM<br>◯ Iri ubochi ufodu na onwa<br>◯ Nmipu<br>◯ Usoro odinala ndi ozo<br>◯ Onweghi aziza
LCL_PP. PROBE: Ndi ogwu a na-ebu site na sirinji ma ọ bụ obere agịga?<br>*Gosi onye aziza foto ahu*<br>[sayana_depo_150x300.jpg]	PP_3 = 5<br>◯ Ntutu<br>◯ Obele ntutu (sayana press)<br>◯ Onweghi Aziza
319. Onwetula ihe imere ka o bu gba mbo na uzo obula iji chetu ma o bu gbochie idi ime	306a ≠ 1 OR 302a ≠ 1<br>◯ E-e<br>◯ Mba<br>◯ Onweghi aziza
320. Afọ ole ka ị bụ mgbe mbụ i ji usoro iji egbu oge ma ọ bụ zere inwe ime? Onye nzaghachi ahụ kwuru na ọ bụ [AGE] afọ ndụ ya n'ụbọchị ọmụmụ ụbọchị ikpeazụ ya.<br>*Denye afor gi na afor Denye -88 ma oburu na onye aziza amaro Denye -99 na nkiti. Onweghi ike ipekari itenani*	302a = 1 OR 306a =1 OR 319 = 1<br>--
321. Ole ụmụ ole dị ndụ ị nwere n'oge ahụ, ọ bụrụ na ọ dị? Note: the respondent said that she gave birth [NUMBER OF LIFE BIRTHS] times in 201.<br>*Denye -99 na nkiti*	Age in 320 ≥ 9 AND 200 = 1<br>--

	${fp_ever_used} = 'yes'
322. Kedu usoro nke izizi ijiri iji chetu ma o bu gbochie idi ime?<br>*Aguputala nhoro nke usoro. Kama hu na igosi putara nhoro nile ka a hu ya.*	319 = 1<br>◯ Itughari akpa nwa nwanyi<br>◯ Itughari amu umu nwoke<br>◯ Igbakwu nye ihe nime aru<br>◯ IUD<br>◯ Igba ntutu<br>◯ Mkpuru ogwu<br>◯ Mgbochi mberede<br>◯ Kondomu umu nwoke<br>◯ Kondomu umu nwanyi<br>◯ Dayaframu<br>◯ Ude/Ofufu<br>◯ Isoro ubochi/Mkpuru ola<br>◯ LAM<br>◯ Iri ubochi ufodu na onwa<br>◯ Nmipu<br>◯ Usoro odinala ndi ozo<br>◯ Onweghi aziza

LCL_322a. JUO NKE OMA: Ntutu agbara ya eji onu ntutu kor obere ntutu<br>*Gosi onye aziza foto ahu*<br>[sayana_depo_150x300.jpg]	322 = 5<br>◯ Ntutu<br>◯ Obele ntutu (sayana press)<br>◯ Onweghi Aziza
322a. Ị jirila ọgwụ mgbochi mberede n'oge ọ bụla n'ime ọnwa iri na	302b ≠ emergency contraception OR 306b ≠ 8

abụọ gara aga?<br>PROBE: As an emergency measure after unprotected sexual intercourse women can take special pills at any time within three to five days to prevent pregnancy.	◯ E-e<br>◯ Mba<br>◯ Onweghi aziza

	((${current_user} = 'no')) and (((${more_children_none} = 'no_children') or …
323a. Isi na e chogho nwa ugbua, mana onwehe ihe ina eme gbasara ngbochi idi ime	302a = 0 AND ((212a or 212b > 2 years) OR (211a or 211b = 2))
323a. Isi na e chogho nwa ugbua, mana onwehe ihe ina eme gbasara ngbochi idi ime	302a = 0 AND ((212a or 212b > 2 years) OR (211a or 211b = 2))
323a. Isi na e chogho nwa ugbua, mana onwehe ihe ina eme gbasara ngbochi idi ime	302a = 0 AND ((212a or 212b > 2 years) OR (211a or 211b = 2))
323a. Isi na e chogho nwa ugbua, mana onwehe ihe ina eme gbasara ngbochi idi ime	302a = 0 AND ((212a or 212b > 2 years) OR (211a or 211b = 2))
E nwe ike egwam ihe kpatara na ongweghi usolo obula iji agbochi ituru ime.<br>JUO NKE OMA: Onwe ihe ozo kpatara.<br>*RECORD ALL REASONS MENTIONED*<br>*Cannot select "Not Married" if 104 is "Yes, currently married".*<br>*Scroll to bottom to see all choices.*	☐ Alubeghi m di<br>☐ Anyi adighi emeko mgbe nile/di m anoghi na ulo<br>☐ Flowa adia gbazi/Nwepu akpa nwa.<br>☐ Subfecund/infecund<br>☐ Ahubeghi m flawa kangbe m muchara nwa ikpeazu<br>☐ Ana m eche nwa ara<br>☐ Di anoriya na otutu ubochi<br>☐ O diri chineke/odighi nfe<br>☐ Onye Aziza ekweghi<br>☐ Di/Enyi ekweghi<br>☐ Ndi ozo ekweghi<br>☐ Okpukperechi gbochori ya<br>☐ Amaghi usoro o bula<br>☐ Amaghi ebe o si pula<br>☐ Ujo ma oga-emeru ya ahu<br>☐ Ometutara ahuike<br>☐ Onweghi ka a ga-esi nweta ya/ebe odi di anya<br>☐ Odi oke onu<br>☐ Usoro ochoro adighi ya<br>☐ Onweghi usoro nke di<br>☐ O maghi otu osi eji ya<br>☐ O na-agbakosi odidi aku madu<br>☐ Ndi ozo<br>☐ Amaghi m<br>☐ Onweghi aziza

323b. Ị ga-asị na ịghara iji ọgwụ mgbochi ime ihe bụ mkpebi gị, karịsịa mkpebi di gị / onye òtù ọlụlụ maọbụ ka gị abụọ kpebiri ọnụ?	302a ≠ 1<br>◯ Obukari onye aziza<br>◯ Obukari di/enyi<br>◯ Nkwekota nmadu nile.<br>◯ Ndi ozo<br>◯ Onweghi aziza
TCI_304. Oburu na obughi di gi/enyi gi, kedu onye ozo na si gi tinye usoro obula gbasara omumu?<br>JUO NKE OMA: Onwe echiche ozo<br>*Aguputala usoro. Hoputa ndi nile daberenu.*	306a = 0<br>☐ Nne<br>☐ Ogo nwayi<br>☐ Umu nwanne di nwanyi<br>☐ Ogo ndi nwanne nwanyi.<br>☐ Mama Ochie<br>☐ Ndi enyi<br>☐ Onye na elokota maka ahu ike<br>☐ Onyeisi obodo<br>☐ Onye ishi ndi uka<br>☐ Anti<br>☐ Umu nwanne gi ndi ozo

	☐ Ndi ozo<br>☐ Nbe ana-eme onu ogugu<br>☐ Onweghi Aziza
TCI_304x. Na ime onwa iri na abuo gara aga, onwere enyi gi/umu nwanne gi si gi tinye usoro obula gbasara omumu?	306a = 0<br>○ E-e<br>○ Mba<br>○ Onweghi aziza
324. Na ime onwa iri na abua gara agaa, onwe onye na eleta maka ahuike biara gwa gi maka ihe gbasara omumu?	009a = 1<br>○ E-e<br>○ Mba<br>○ Onweghi aziza
325a. N'ime ọnwa iri na abụọ gara aga, ị gara ileta ahụike ma ọ bụ ogige maka ilekọta onwe gị?<br>*Maka oru nleta ahuike obula*	009a = 1<br>○ E-e<br>○ Mba<br>○ Onweghi aziza
325a. Na ime onwa iri na abua gara agaa, onwe ngbe e gara hu ndi na eleta ahuike, kor bu umu gi gara?<br>*Maka oru nleta ahuike obula*	009a = 1<br>○ E-e<br>○ Mba<br>○ Onweghi aziza
325b. O nwere onye na-aru oru na ulo nleta ahuike ahu igara nke kwu oro gi okwu maka nhazi nke omumu nwa nke ezi na ulo?	325a = 1<br>○ E-e<br>○ Mba<br>○ Onweghi aziza
TCI_305. Onwe ngbe e gara n'ime afor gara aga ebe ndi obodo kowachara ofuma bayare ihe gbasara usolo omumu	009a = 1<br>○ E-e<br>○ Mba<br>○ Onweghi aziza
TCI_306. Ichee ma onwe ndi ime o bodo unu ga kpo gi ajo aha mor bu hapu ibu enyi gi ma ha choputa na e tinye usolo obula maka omumu?	009a = 1<br>○ E-e<br>○ Mba<br>○ Amaghi m<br>○ Onweghi aziza
TCI_307. Ichee ma onwe ndi obodo'a ga ekele gi, nye gi nkwado, mor gwa gi okwu di nma ma ha chuputa na e tinye usolo obula gbasara omumu?	009a = 1<br>○ E-e<br>○ Mba<br>○ Amaghi m<br>○ Onweghi aziza
TCI_308. Na ime onwa iri na abuo gara aga, onwe ngbe inuru ofu onye na ime ndia nile kwuru okwu DINMA na ebe otutu nmadu gbakoo gbasara usolo omumu?<br>*Guputa usoro nile ki horo nke bayere'n*	009a = 1<br>☐ Ndị ọrụ gọọmenti (ọkwa mba)<br>☐ Steti, LGA, mor ndi ichie<br>☐ Onye ishi ndi uka<br>☐ Onweghi nke di n'elu.<br>☐ Onweghi Aziza
TCI_309. Na ime onwa iri na abuo gara aga, inuru ofu onye n' ime ndia nile kwuru okwu DINJO gbasara usoro omumu?<br>*Guputa usoro nile ki horo nke bayere'n*	009a = 1<br>☐ Ndị ọrụ gọọmenti (ọkwa mba)<br>☐ Steti, LGA, mor ndi ichie<br>☐ Onye ishi ndi uka<br>☐ Onweghi nke di n'elu.<br>☐ Onweghi Aziza
TCI_309x. Ndi ezigbo oryigi nole na umu nwannegi ka e chee na ha tinye usolo abula gbasara omumu: onweghi, ufordu, otutu, kor hancha?	009a = 1<br>○ Onweghi<br>○ Ufodu<br>○ Karichaa<br>○ Hanile<br>○ Amaghi m<br>○ Onweghi Aziza

				009a = 1
326. Nime onwa ele na ole gara aga:				
	E-e	Mba	Onweghi aziza	
	○	○	○	

326a. Inutula maka nhazi nke omumu nwa na redio?........	○	○	○
326b. Inutula ihe obula banyere nhazi omumu nwa nke ezi na ulo na igwe onyonyo?	○	○	○
326c. Igutula maka nhazi omumu nwa nke ezi na ulo na akwukwo mgbasa ozi ka o bu magazin?........	○	○	○
326d. Onwe onye ji ekwe nti kpogi mor bu zitara gi ozi gbasara omumu?	○	○	○
TCI_310. E gutugo maka usolo omumu na nnukwu akwukwo, npepe, nke ana eke ghari eke ghari?	○	○	○
TCI_311. Ihutugo poster mor billboard ebe a kowa usoro omumu?	○	○	○

Section 4 – Mmekọahụ

Lelee nlekọta ndị ọzọ. MGBE Ị GA-AGBANYE, MEGU KWESỊRỊ ỊBỤRỤ NDỊ NA-ECHICHE.

Ugbu a, m ga-achọ ịjụ ụfọdụ ajụjụ banyere mmekọahụ iji nweta nghọta ka mma banyere nsogbu ndụ dị mkpa. Ka m mesie gị obi ike na azịza gị dị nzuzo na agaghị agwa onye ọ bụla. Ọ bụrụ na anyị ga-abịa ajụjụ ọ bụla ị na-achọghị ịza, dịka ka m mara ma anyị ga-aga ajụjụ na-esonụ.	${consent_obtained}

	${consent_obtained}
401a. Afo ole ka idi mgbe gi na nwoke mere nmeko nke izizi?	309a = 1
Current age: [AGE]	
Number of live births: [NUMBER OF LIFE BIRTHS]	${birth_events} > 0
Onye aziza di ime.	${pregnant} = 'yes'
Denye afor odi na afor<br>*Denye -77 ma oburu na ya na nwoke edinabe chacha Denye -99 na nkiti Denye -88 ma oburu na imaro*	--

	((${age_at_first_sex} >= 0) or (${age_at_first_sex} =-88) or (${age_at_first_sex} =-99))
402. Kedu mgbe ikpe azu gi na nwoke nwere nmeko?	401a ≠ -77

402. Enter [# days / weeks / months / years].<br>*Oburu taa, denye zero n'ubochi, oburo zero na izu uka/onwa/afor.*<br>*Ngbe nwoke na nwanyi mekota ga ekwetakori ma ngbe afo ime bidoo*	401a ≠ -77<br>--
LCL_403. Ọ bụrụ na gị na nwoke nwee mmeko nani out ugboro na enweghi ihe igbochi afo ime, ichere na iga aturu ime?<br>*Na-agụpụta nhọrọ nzaghachi.*	${consent_obtained}<br>○ Very likely<br>○ Somewhat likely<br>○ Equally likely and unlikely<br>○ Not likely<br>○ Amaghi m<br>○ Onweghi aziza
LCL_404. Ọ bụrụ na gị na nwoke nwee mmeko ngbenile, morbu ugboroabue na izu uka, na ime afor, ichere na iga aturu ime?<br>*Na-agụpụta nhọrọ nzaghachi.*	${consent_obtained}<br>○ Very likely<br>○ Somewhat likely<br>○ Equally likely and unlikely<br>○ Not likely<br>○ Amaghi m<br>○ Onweghi aziza

Section 6 – Menstrual Hygiene

Ugbu a, m ga-ajụ gị banyere nlekọta ịdị ọcha nke nwoke. Nke a na-agụnye iji ihe ndị nwere ihe ọmụma; ịbanye na nzuzo, ebe dị ọcha, ebe nchekwa; asa dị ka achọrọ; na ebe ị ga-eji ihe eji eme ihe.

602a. Ebee ka ị na-agbanwekarị ihe mgbochi, ákwà, ma ọ bụ ihe ndị ọzọ dị ọcha?	(209 ≤ 90 days, 13 weeks or ≤ 3 months)<br>○ Ghichaa / wukwasi ụlọ mposi<br>○ A na-eme ka ọkpụkpụ latrine dị mma<br>○ Oghere olulu na ụdọ<br>○ Ogige na-enweghị oghere ma ọ

	bụ olulu mmiri<br>○ Ụlọ mposi ịwụ<br>○ Ụlọ mposi na-edozi ahụ<br>○ Ụlọ mposi na-ekpuchi / Latrine jikọtara<br>○ Ebe ihi ụra / ime<br>○ Azu<br>○ Enweghị ebe / ohia / ubi<br>○ Ndi ozo<br>○ Onweghi aziza
Place: [MAIN PLACE FROM 602a] 602b. Mgbe ị na-achịkwa ịdị ọcha nke nwoke gị, ọ bụ ebe a:<br>*Na-agụpụta nhọrọ ọ bụla ma họrọ ma ọ bụrụ ee.*	601 ≠ -99 nor null AND 602 ≠ -99 nor null<br>☐ dị ọcha<br>☐ nzuzo<br>☐ Nchekwa?<br>☐ Enwere ike ikpochi?<br>☐ Na-enye mmiri?<br>☐ Nyere ncha?<br>☐ Onweghi nke di n'elu.<br>☐ Onweghi aziza
603. N'ime oge izu ike ikpeazụ gị, gịnị ka ị na-eji iji nakọta ma ọ bụ weghara ọbara ọbara gị? PROBE: Anything else?<br>*Aguputala usoro. Hoputa ndi nile daberenu.*	(209 ≤ 90 days, 13 weeks or ≤ 3 months)<br>☐ Ịkwụsị akwa mpempe akwụkwọ (azụmahịa)<br>☐ Azu mmiri na-ehichapụkwa<br>☐ ákwà ọhụrụ<br>☐ ochie ákwà<br>☐ Acha owu<br>☐ Ọkpụkpọ<br>☐ Ụdị<br>☐ Akwụkwọ mpempe akwụkwọ<br>☐ Nanị uwe<br>☐ Ịwụ<br>☐ Ndi ozo<br>☐ Enweghị ihe eji<br>☐ Onweghi aziza
604a. Ị na-asacha ma jigharịa ihe mgbochi, ákwà, ma ọ bụ ihe ndị ọzọ dị ọcha n'oge ị ga-eji oge ị ga-eme?	603 = reusable sanitary pad, old cloth, underwear alone, or bucket<br>○ E-e<br>○ Mba<br>○ Onweghi aziza
604b. N'ime oge izu ike gị, ọ bụ ihe ndị dị ọcha ị sacha ma mee ka ha dị ọcha kpam kpam tupu ejigharị ya?	604a = 1<br>○ E-e<br>○ Mba<br>○ Onweghi aziza
605. You mentioned that you used [ODK will display the responses from 603] during your last menstrual period. Where did you dispose of these materials after use? PROBE: Anywhere else?<br>*Aguputala usoro. Hoputa ndi nile daberenu.*	603 = disposable sanitary pad, new cloth, cotton wool, diaper, tampons, toilet paper, or other OR 604a =0<br>☐ Wụpụ ụlọ mposi<br>☐ Latrine<br>☐ Azu ahihia / ahihia<br>☐ Ọkụ<br>☐ Bush / ubi<br>☐ Ndi ozo<br>☐ Onweghi aziza
606a. Aside from your own housework, have you done any work in the last month?	(209 ≤ 90 days, 13 weeks or ≤ 3 months)<br>○ E-e<br>○ Mba<br>○ Onweghi aziza
606b. Due to your last menstrual period, were there any work days in the last month that you did not attend?	606a = 1<br>○ E-e<br>○ Mba<br>○ Onweghi aziza

607a. Did you attend school at any time in the past 12 months?	(209 ≤ 90 days, 13 weeks or ≤ 3 months)<br>○ E-e<br>○ Mba<br>○ Onweghi aziza
607b. N'ihi oge izu ike gị, ọ dị ụbọchị ọmụmụ ọ bụla n'ime ọnwa 12 gara aga na ị garaghị?	607a = 1<br>○ E-e<br>○ Mba<br>○ Onweghi aziza

Section 7.1 – Ezigbo enyi

701. Ugbu a, achọrọ m ịjụ ụfọdụ ajụjụ banyere ndị enyi gị bụ ezigbo enyi, ndị inyom ị na-ekerịta ozi nkeonwe na ndị na-akọrọ gị ozi nkeonwe ha. Umu enyi nwanyi di otua ole ka i nwere na Naijiria ndi di n'agbata afo 15 na 49<br>*Enter -88 for do not know, -99 for no response.*	${consent_obtained}<br>--
702a. Biko olee enyi gị nwanyị kacha noro gi nso na Nigeria di na agbata afo 15 na 49 onye gị na ya na akpakorita maka onwe unu. Ka odi mfe ịkọwa nwanyị a, biko nye aha ya ma ọ bụ aha efu.	${friend_count} > 0<br>--
703a. Afọ ole ka ${friend1_name} ogbara nubochi emume omumu ya nke ikpeazu?<br>*Enter -88 for do not know, -99 for no response.*	${friend_count} > 0<br>--
704a. Kedu ụlọ akwụkwọ kachasị elu ${friend1_name} ogara?	${friend_count} > 0<br>○ A gaghi m.<br>○ Paramari<br>○ Sekondiri<br>○ Nkedi elu<br>○ Onweghi aziza
702b. Biko olee enyi gị nwanyị kacha noro gi nso na Nigeria di na agbata afo 15 na 49 onye gị na ya na akpakorita maka onwe unu. Ka odi mfe ịkọwa nwanyị a, biko nye aha ya ma ọ bụ aha efu.	${friend_count} > 1<br>--
703b. Afọ ole ka ${friend2_name} ogbara nubochi emume omumu ya nke ikpeazu?<br>*Enter -88 for do not know, -99 for no response.*	${friend_count} > 1<br>--
704b. Kedu ụlọ akwụkwọ kachasị elu ${friend2_name} ogara?	${friend_count} > 1<br>○ A gaghi m.<br>○ Paramari<br>○ Sekondiri<br>○ Nkedi elu<br>○ Onweghi aziza

Section 7.2 – Abortion

CHECK FOR THE PRESENCE OF OTHERS. BEFORE CONTINUING, MAKE EVERY EFFORT TO ENSURE PRIVACY.

The next series of questions are about things women in your community do to remove a pregnancy or have an abortion. This is a common experience in Nigeria and we simply want to better understand what women do to remove a pregnancy or have an abortion. I want to remind you that this survey is completely confidential and anonymous and your responses will not be shared with anyone. If we should come to any question that you don't want to answer, just let me know and I will skip to the next question.<br>*Press OK to continue.*	${consent_obtained}<br>☐ o di mma
705. Mgbe ụfọdụ ndị inyom na-eche na ha dị ime ma ọ bụ na ha tuụrụ ime mgbe ha achọghị, ha na-eme ihe iji wepu ime ma ọ bụ emebi ime. Kedu nke na emekari na ogbe e bi ki ta?<br>*Read response options aloud.*	${consent_obtained}<br>○ Ọ dịkarị<br>○ Ọ bụ ihe nkịtị<br>○ Ọ bụghị ihe nkịtị<br>○ Ọ bụghị mgbe niile<br>○ Amaghi m<br>○ Onweghi aziza
706. Kedu uzo di iche iche umu nwanyị nọ n'ime obodo e bi ki ta, nke dị ime ma ọ bụ na-eche na ọ dị ime nwere ike isi iwepu ime? Ọ	${consent_obtained}<br>☐ Usoro eji eme oparation

nwere ihe ọzọ?<br>*Do not read options aloud. Select all that apply.*	☐ Ngwá ọgwụ akpọrọ mifepristone ma ọ bụ misoprostol<br>☐ Mkpụrụ ọgwụ ị na-ewere mgbe ị nwere ahụ ọkụ dị ka ọgwụ nje ma ọ bụ ọgwụ na-emegide malarial<br>☐ Mgbochi ọgwụ mgbochi mberede<br>☐ Mkpụrụ ọgwụ, ụdị akọwapụtaghị<br>☐ Ọgwụ Ọgbugba<br>☐ uzo omenala, dika ahihia<br>☐ Mmanya<br>☐ nnu, akpu, maggi<br>☐ oroma nkịrịsị<br>☐ Nsogbu sirop<br>☐ Fanye ihe n'ime ikpu<br>☐ Ndi ozo<br>☐ Amaghi m<br>☐ Onweghi aziza
707. Kedu out n'ime ụzọ ndị ha kacha eme?	count-selected(${abt_ways}) > 1<br>○ Usoro eji eme oparation<br>○ Ngwá ọgwụ akpọrọ mifepristone ma ọ bụ misoprostol<br>○ Mkpụrụ ọgwụ ị na-ewere mgbe ị nwere ahụ ọkụ dị ka ọgwụ nje ma ọ bụ ọgwụ na-emegide malarial<br>○ Mgbochi ọgwụ mgbochi mberede<br>○ Mkpụrụ ọgwụ, ụdị akọwapụtaghị<br>○ Ọgwụ Ọgbugba<br>○ uzo omenala, dika ahihia<br>○ Mmanya<br>○ nnu, akpu, maggi<br>○ oroma nkịrịsị<br>○ Nsogbu sirop<br>○ Fanye ihe n'ime ikpu<br>○ Ndi ozo<br>○ Amaghi m<br>○ Onweghi aziza<br>selected(${abt_ways}, filter) or (filter = 'always')
708. Ebee ka ndị inyom nọ n'ógbè gị na-aga maka usoro ịwa ahụ iji wepu ime ma ọ bụ imebi ime? N'ebe ọ bụla ọzọ?<br>*Do not read options aloud. Select all that apply.*	(selected(${abt_ways},'surgery'))<br>☐ Ulonleta ahuike goomenti<br>☐ Senta ahuike nke goomenti<br>☐ Ulo nleta ntazi omu mu nwa<br>☐ Ulo nleta mbughari<br>☐ TBA/Fieldworker (public)<br>☐ Ulo nleta ahuike nke onwe<br>☐ Famasi<br>☐ mmiri ogwu<br>☐ Dokita onweya morbu nurse<br>☐ Dokinta Onwe<br>☐ TBA/Fieldworker (private)<br>☐ Shopu<br>☐ FBO/Church<br>☐ Ndi enyi/Ndi ezi na ulo<br>☐ NGO<br>☐ Ahia<br>☐ Ndi ozo<br>☐ Amaghi m<br>☐ Onweghi aziza
709. Kedu ebe dị iche iche ha kachasị aga?	count-selected(${abt_surg_where}) > 1<br>○ Ulonleta ahuike goomenti<br>○ Senta ahuike nke goomenti<br>○ Ulo nleta ntazi omu mu nwa

- Ulo nleta mbughari
- TBA/Fieldworker (public)
- Ulo nleta ahuike nke onwe
- Famasi
- mmiri ogwu
- Dokita onweya morbu nurse
- Dokinta Onwe
- TBA/Fieldworker (private)
- Shopu
- FBO/Church
- Ndi enyi/Ndi ezi na ulo
- NGO
- Ahia
- Ndi ozo
- Amaghi m
- Onweghi aziza

selected(${abt_surg_where}, filter) or (filter = 'always')

---

710. Ebee ka ụmụ nwanyị na-esi enweta ọgwụ iji wepụ ime ma ọ bụ imebi ime? N'ebe ọ bụla ọzọ?

(selected(${abt_ways}, 'pills_abortion')) or (selected(${abt_ways}, 'pills_fever')) or (selected($ …

- Ulonleta ahuike goomenti
- Senta ahuike nke goomenti
- Ulo nleta ntazi omu mu nwa
- Ulo nleta mbughari
- TBA/Fieldworker (public)
- Ulo nleta ahuike nke onwe
- Famasi
- mmiri ogwu
- Dokita onweya morbu nurse
- Dokinta Onwe
- TBA/Fieldworker (private)
- Shopu
- FBO/Church
- Ndi enyi/Ndi ezi na ulo
- NGO
- Ahia
- Ndi ozo
- Amaghi m
- Onweghi aziza

---

711. Kedu usoro di iche iche ha kachasị eso?

count-selected(${abt_meds_where}) > 1

- Ulonleta ahuike goomenti
- Senta ahuike nke goomenti
- Ulo nleta ntazi omu mu nwa
- Ulo nleta mbughari
- TBA/Fieldworker (public)
- Ulo nleta ahuike nke onwe
- Famasi
- mmiri ogwu
- Dokita onweya morbu nurse
- Dokinta Onwe
- TBA/Fieldworker (private)
- Shopu
- FBO/Church
- Ndi enyi/Ndi ezi na ulo
- NGO
- Ahia
- Ndi ozo
- Amaghi m
- Onweghi aziza

selected(${abt_meds_where}, filter) or (filter = 'always')

712a.i. Onwego ihe ${friend1_name} mere iji wepụ afo ime mgbe ọ dị ime ma ọ bụ chee na ọ dị ime?<br>*Probe to confirm whether the pregnancy removal was successful.*	◯ E-e, amara m nke oma<br>◯ E-e, echere m otua<br>◯ Mba<br>◯ Amaghi m<br>◯ Onweghi aziza
713a.i. Kedu na afo omere ya ikpeazụ?<br>*If indicates happened more than once, specify most recent time.*<br>*Enter 2020 for 'Do not know' or 'No response'.*	(${friend1_abt_yn} = 'yes') or (${friend1_abt_yn} = 'likely')<br>Year: ________________
714a.i. Ndị inyom mgbe ụfọdụ na-eme ọtụtụ ihe iji gbochie afo ime iga n'iru .Odi otutu ihe ${friend1_name} na - eme iji wepu ime ?	(${friend1_abt_yn} = 'yes') or (${friend1_abt_yn} = 'likely')<br>◯ E-e, amara m nke oma<br>◯ E-e, echere m otua<br>◯ Mba<br>◯ Amaghi m<br>◯ Onweghi aziza
715a.i. Kedu ihe mbụ o mere iji wepụ ime ya?	(${friend1_abt_mult_yn} = 'yes') or (${friend1_abt_mult_yn} = 'likely')<br>◯ Usoro eji eme oparation<br>◯ Ngwá ọgwụ akpọrọ mifepristone ma ọ bụ misoprostol<br>◯ Mkpụrụ ọgwụ ị na-ewere mgbe ị nwere ahụ ọkụ dị ka ọgwụ nje ma ọ bụ ọgwụ na-emegide malarial<br>◯ Mgbochi ọgwụ mgbochi mberede<br>◯ Mkpụrụ ọgwụ, ụdị akọwapụtaghị<br>◯ Ọgwụ Ọgbugba<br>◯ uzo omenala, dika ahihia<br>◯ Mmanya<br>◯ nnu, akpu, maggi<br>◯ oroma nkịrịsị<br>◯ Nsogbu sirop<br>◯ Fanye ihe n'ime ikpu<br>◯ Ndi ozo<br>◯ Amaghi m<br>◯ Onweghi aziza
715a.i. Kedu ihe o mere nke wepụrụ ime ya?	(${friend1_abt_mult_yn} = 'no') or (${friend1_abt_mult_yn} = '-88')<br>◯ Usoro eji eme oparation<br>◯ Ngwá ọgwụ akpọrọ mifepristone ma ọ bụ misoprostol<br>◯ Mkpụrụ ọgwụ ị na-ewere mgbe ị nwere ahụ ọkụ dị ka ọgwụ nje ma ọ bụ ọgwụ na-emegide malarial<br>◯ Mgbochi ọgwụ mgbochi mberede<br>◯ Mkpụrụ ọgwụ, ụdị akọwapụtaghị<br>◯ Ọgwụ Ọgbugba<br>◯ uzo omenala, dika ahihia<br>◯ Mmanya<br>◯ nnu, akpu, maggi<br>◯ oroma nkịrịsị<br>◯ Nsogbu sirop<br>◯ Fanye ihe n'ime ikpu<br>◯ Ndi ozo<br>◯ Amaghi m<br>◯ Onweghi aziza
716a.i. Ebee ka ọ gara maka usoro ahụ?	(${friend1_abt_first} = 'surgery') or (${friend1_abt_only} = 'surgery')<br>◯ Ulonleta ahuike goomenti<br>◯ Senta ahuike nke goomenti<br>◯ Ulo nleta ntazi omu mu nwa<br>◯ Ulo nleta mbughari<br>◯ TBA/Fieldworker (public)

	○ Ulo nleta ahuike nke onwe<br>○ Famasi<br>○ mmiri ogwu<br>○ Dokita onweya morbu nurse<br>○ Dokinta Onwe<br>○ TBA/Fieldworker (private)<br>○ Shopu<br>○ FBO/Church<br>○ Ndi enyi/Ndi ezi na ulo<br>○ NGO<br>○ Ahia<br>○ Ndi ozo<br>○ Amaghi m<br>○ Onweghi aziza
717a.i. Ebee ka o nwetara ọgwụ?	(${friend1_abt_first} = 'pills_abortion') or (${friend1_abt_only} = 'pills_abortion') or (${friend …<br>○ Ulonleta ahuike goomenti<br>○ Senta ahuike nke goomenti<br>○ Ulo nleta ntazi omu mu nwa<br>○ Ulo nleta mbughari<br>○ TBA/Fieldworker (public)<br>○ Ulo nleta ahuike nke onwe<br>○ Famasi<br>○ mmiri ogwu<br>○ Dokita onweya morbu nurse<br>○ Dokinta Onwe<br>○ TBA/Fieldworker (private)<br>○ Shopu<br>○ FBO/Church<br>○ Ndi enyi/Ndi ezi na ulo<br>○ NGO<br>○ Ahia<br>○ Ndi ozo<br>○ Amaghi m<br>○ Onweghi aziza
718a.i. Gịnị bụ ihe ikpeazụ ọ mere nke mechara mgbe o wepụ afọ ime ahu?	(${friend1_abt_mult_yn} = 'yes') or (${friend1_abt_mult_yn} = 'likely')<br>○ Usoro eji eme oparation<br>○ Ngwá ọgwụ akpọrọ mifepristone ma ọ bụ misoprostol<br>○ Mkpụrụ ọgwụ ị na-ewere mgbe ị nwere ahụ ọkụ dị ka ọgwụ nje ma ọ bụ ọgwụ na-emegide malarial<br>○ Mgbochi ọgwụ mgbochi mberede<br>○ Mkpụrụ ọgwụ, ụdị akọwapụtaghị<br>○ Ọgwụ Ọgbugba<br>○ uzo omenala, dika ahihia<br>○ Mmanya<br>○ nnu, akpu, maggi<br>○ oroma nkịrịsị<br>○ Nsogbu sirop<br>○ Fanye ihe n'ime ikpu<br>○ Ndi ozo<br>○ Amaghi m<br>○ Onweghi aziza
719a.i. Ebee ka ọ gara maka usoro ahụ ?	${friend1_abt_last} = 'surgery'<br>○ Ulonleta ahuike goomenti<br>○ Senta ahuike nke goomenti<br>○ Ulo nleta ntazi omu mu nwa<br>○ Ulo nleta mbughari<br>○ TBA/Fieldworker (public)

	◯ Ulo nleta ahuike nke onwe<br>◯ Famasi<br>◯ mmiri ogwu<br>◯ Dokita onweya morbu nurse<br>◯ Dokinta Onwe<br>◯ TBA/Fieldworker (private)<br>◯ Shopu<br>◯ FBO/Church<br>◯ Ndi enyi/Ndi ezi na ulo<br>◯ NGO<br>◯ Ahia<br>◯ Ndi ozo<br>◯ Amaghi m<br>◯ Onweghi aziza
720a.i. Ebee ka o nwetara ọgwụ?	(${friend1_abt_last} = 'pills_abortion') or (${friend1_abt_last} = 'pills_fever') or (${friend1_ab …<br>◯ Ulonleta ahuike goomenti<br>◯ Senta ahuike nke goomenti<br>◯ Ulo nleta ntazi omu mu nwa<br>◯ Ulo nleta mbughari<br>◯ TBA/Fieldworker (public)<br>◯ Ulo nleta ahuike nke onwe<br>◯ Famasi<br>◯ mmiri ogwu<br>◯ Dokita onweya morbu nurse<br>◯ Dokinta Onwe<br>◯ TBA/Fieldworker (private)<br>◯ Shopu<br>◯ FBO/Church<br>◯ Ndi enyi/Ndi ezi na ulo<br>◯ NGO<br>◯ Ahia<br>◯ Ndi ozo<br>◯ Amaghi m<br>◯ Onweghi aziza
721a.i. ${friend1_name} O nwere nsogbu wee gaa ulo ogwu maka usoro iwepu afo ime mo obu imebi afo ime?<br>*If the respondent already reported the friend went to a health facility in the process of removing the pregnancy, we are interested in whether the friend went back to a health facility on a separate occasion to treat complications that she may have experienced.*	(${friend1_abt_yn} = 'yes') or (${friend1_abt_yn} = 'likely')<br>◯ E-e, amara m nke oma<br>◯ E-e, echere m otua<br>◯ Mba<br>◯ Amaghi m<br>◯ Onweghi aziza
712a.ii. Hapu ihe ndia, Onwetugo oge ${friend1_name} mere ihe obula iji ghazi oge nso ya ngbe ochere na odi ime?<br>*Probe to confirm whether the period regulation was successful. If not, select 'no.'*	${friend1_abt_yn} = 'yes'<br>◯ E-e, amara m nke oma<br>◯ E-e, echere m otua<br>◯ Mba<br>◯ Amaghi m<br>◯ Onweghi aziza
712a.ii. Onwetugo oge ${friend1_name} mere ihe obula iji ghazi oge nso ya ngbe ochere na odi ime?<br>*Probe to confirm whether the period regulation was successful. If not, select 'no.'*	${friend1_abt_yn} != 'yes'<br>◯ E-e, amara m nke oma<br>◯ E-e, echere m otua<br>◯ Mba<br>◯ Amaghi m<br>◯ Onweghi aziza
713a.ii. Kedu na afo omere ya ikpeazụ?<br>*If indicates happened more than once, specify most recent time.*<br>*Enter 2020 for 'Do not know' or 'No response'.*	(${friend1_reg_yn} = 'yes') or (${friend1_reg_yn} = 'likely')<br>Year: ___________
714a.ii. Ndị inyom mgbe ụfọdụ na-eme ọtụtụ ihe iji gbochie afo ime iga n'iru .Odi otutu ihe ${friend1_name} na - eme iji wepu ime ?	((${friend1_reg_year} > ${friend1_abt_year}) or (${friend1_abt_year} = '')) and ((${friend1_reg_yn …<br>◯ E-e, amara m nke oma

	◌ E-e, echere m otua<br>◌ Mba<br>◌ Amaghi m<br>◌ Onweghi aziza
715a.ii. Kedu ihe mbụ o mere iji wepụ ime ya?	((${friend1_reg_year} > ${friend1_abt_year}) or (${friend1_abt_year} = '')) and ((${friend1_reg_mu …<br>◌ Usoro eji eme oparation<br>◌ Ngwá ọgwụ akpọrọ mifepristone ma ọ bụ misoprostol<br>◌ Mkpụrụ ọgwụ ị na-ewere mgbe ị nwere ahụ ọkụ dị ka ọgwụ nje ma ọ bụ ọgwụ na-emegide malarial<br>◌ Mgbochi ọgwụ mgbochi mberede<br>◌ Mkpụrụ ọgwụ, ụdị akọwapụtaghị<br>◌ Ọgwụ Ọgbugba<br>◌ uzo omenala, dika ahihia<br>◌ Mmanya<br>◌ nnu, akpu, maggi<br>◌ oroma nkịrịsị<br>◌ Nsogbu sirop<br>◌ Fanye ihe n'ime ikpu<br>◌ Ndi ozo<br>◌ Amaghi m<br>◌ Onweghi aziza
715a.ii. Kedu ihe o mere nke wepụrụ ime ya?	((${friend1_reg_year} > ${friend1_abt_year}) or (${friend1_abt_year} = '')) and ((${friend1_reg_mu …<br>◌ Usoro eji eme oparation<br>◌ Ngwá ọgwụ akpọrọ mifepristone ma ọ bụ misoprostol<br>◌ Mkpụrụ ọgwụ ị na-ewere mgbe ị nwere ahụ ọkụ dị ka ọgwụ nje ma ọ bụ ọgwụ na-emegide malarial<br>◌ Mgbochi ọgwụ mgbochi mberede<br>◌ Mkpụrụ ọgwụ, ụdị akọwapụtaghị<br>◌ Ọgwụ Ọgbugba<br>◌ uzo omenala, dika ahihia<br>◌ Mmanya<br>◌ nnu, akpu, maggi<br>◌ oroma nkịrịsị<br>◌ Nsogbu sirop<br>◌ Fanye ihe n'ime ikpu<br>◌ Ndi ozo<br>◌ Amaghi m<br>◌ Onweghi aziza
716a.ii. Ebee ka ọ gara maka usoro ahụ?	((${friend1_reg_year} > ${friend1_abt_year}) or (${friend1_abt_year} = '')) and ((${friend1_reg_fi …<br>◌ Ulonleta ahuike goomenti<br>◌ Senta ahuike nke goomenti<br>◌ Ulo nleta ntazi omu mu nwa<br>◌ Ulo nleta mbughari<br>◌ TBA/Fieldworker (public)<br>◌ Ulo nleta ahuike nke onwe<br>◌ Famasi<br>◌ mmiri ogwu<br>◌ Dokita onweya morbu nurse<br>◌ Dokinta Onwe<br>◌ TBA/Fieldworker (private)<br>◌ Shopu<br>◌ FBO/Church<br>◌ Ndi enyi/Ndi ezi na ulo

	NGO<br>Ahia<br>Ndi ozo<br>Amaghi m<br>Onweghi aziza
717a.ii. Ebee ka o nwetara ọgwụ?	((${friend1_reg_year} > ${friend1_abt_year}) or (${friend1_abt_year} = '')) and ((${friend1_reg_fi …<br>Ulonleta ahuike goomenti<br>Senta ahuike nke goomenti<br>Ulo nleta ntazi omu mu nwa<br>Ulo nleta mbughari<br>TBA/Fieldworker (public)<br>Ulo nleta ahuike nke onwe<br>Famasi<br>mmiri ogwu<br>Dokita onweya morbu nurse<br>Dokinta Onwe<br>TBA/Fieldworker (private)<br>Shopu<br>FBO/Church<br>Ndi enyi/Ndi ezi na ulo<br>NGO<br>Ahia<br>Ndi ozo<br>Amaghi m<br>Onweghi aziza
718a.ii. Gịnị bụ ihe ikpeazụ ọ mere nke mechara mgbe o wepụ afọ ime ahu?	((${friend1_reg_year} > ${friend1_abt_year}) or (${friend1_abt_year} = '')) and ((${friend1_reg_mu …<br>Usoro eji eme oparation<br>Ngwá ọgwụ akpọrọ mifepristone ma ọ bụ misoprostol<br>Mkpụrụ ọgwụ ị na-ewere mgbe ị nwere ahụ ọkụ dị ka ọgwụ nje ma ọ bụ ọgwụ na-emegide malarial<br>Mgbochi ọgwụ mgbochi mberede<br>Mkpụrụ ọgwụ, ụdị akọwapụtaghị<br>Ọgwụ Ọgbugba<br>uzo omenala, dika ahihia<br>Mmanya<br>nnu, akpu, maggi<br>oroma nkịrịsị<br>Nsogbu sirop<br>Fanye ihe n'ime ikpu<br>Ndi ozo<br>Amaghi m<br>Onweghi aziza
719a.ii. Ebee ka ọ gara maka usoro ahụ ?	((${friend1_reg_year} > ${friend1_abt_year}) or (${friend1_abt_year} = '')) and (${friend1_reg_las …<br>Ulonleta ahuike goomenti<br>Senta ahuike nke goomenti<br>Ulo nleta ntazi omu mu nwa<br>Ulo nleta mbughari<br>TBA/Fieldworker (public)<br>Ulo nleta ahuike nke onwe<br>Famasi<br>mmiri ogwu<br>Dokita onweya morbu nurse<br>Dokinta Onwe<br>TBA/Fieldworker (private)<br>Shopu

	FBO/Church<br>Ndi enyi/Ndi ezi na ulo<br>NGO<br>Ahia<br>Ndi ozo<br>Amaghi m<br>Onweghi aziza
720a.ii. Ebee ka o nwetara ọgwụ?	((${friend1_reg_year} > ${friend1_abt_year}) or (${friend1_abt_year} = '')) and ((${friend1_reg_la …<br>Ulonleta ahuike goomenti<br>Senta ahuike nke goomenti<br>Ulo nleta ntazi omu mu nwa<br>Ulo nleta mbughari<br>TBA/Fieldworker (public)<br>Ulo nleta ahuike nke onwe<br>Famasi<br>mmiri ogwu<br>Dokita onweya morbu nurse<br>Dokinta Onwe<br>TBA/Fieldworker (private)<br>Shopu<br>FBO/Church<br>Ndi enyi/Ndi ezi na ulo<br>NGO<br>Ahia<br>Ndi ozo<br>Amaghi m<br>Onweghi aziza
721a.ii. ${friend1_name} O nwere nsogbu wee gaa ulo ogwu maka usoro iwepu afo ime mo obu imebi afo ime?<br>*If the respondent already reported the friend went to a health facility in the process of regulating her period, we are interested in whether the friend went back to a health facility on a separate occasion to treat complications that she may have experienced.*	((${friend1_reg_year} > ${friend1_abt_year}) or (${friend1_abt_year} = '')) and ((${friend1_reg_yn …<br>E-e, amara m nke oma<br>E-e, echere m otua<br>Mba<br>Amaghi m<br>Onweghi aziza
712b.i. Onwego ihe ${friend2_name} mere iji wepụ afo ime mgbe ọ dị ime ma ọ bụ chee na ọ dị ime?<br>*Probe to confirm whether the pregnancy removal was successful.*	E-e, amara m nke oma<br>E-e, echere m otua<br>Mba<br>Amaghi m<br>Onweghi aziza
713b.i. Kedu na afo omere ya ikpeazụ?<br>*If indicates happened more than once, specify most recent time.*<br>*Enter 2020 for 'Do not know' or 'No response'.*	(${friend2_abt_yn} = 'yes') or (${friend2_abt_yn} = 'likely')<br>Year: ________________
714b.i. Ndị inyom mgbe ụfọdụ na-eme ọtụtụ ihe iji gbochie afo ime iga n'iru .Odi otutu ihe ${friend2_name} na - eme iji wepu ime ?	(${friend2_abt_yn} = 'yes') or (${friend2_abt_yn} = 'likely')<br>E-e, amara m nke oma<br>E-e, echere m otua<br>Mba<br>Amaghi m<br>Onweghi aziza
715b.i. Kedu ihe mbụ o mere iji wepụ ime ya?	(${friend2_abt_mult_yn} = 'yes') or (${friend2_abt_mult_yn} = 'likely')<br>Usoro eji eme oparation<br>Ngwá ọgwụ akpọrọ mifepristone ma ọ bụ misoprostol<br>Mkpụrụ ọgwụ ị na-ewere mgbe ị nwere ahụ ọkụ dị ka ọgwụ nje ma ọ bụ ọgwụ na-emegide malarial<br>Mgbochi ọgwụ mgbochi mberede<br>Mkpụrụ ọgwụ, ụdị akọwapụtaghị

	◌ Ọgwụ Ọgbugba<br>◌ uzo omenala, dika ahihia<br>◌ Mmanya<br>◌ nnu, akpu, maggi<br>◌ oroma nkịrịsị<br>◌ Nsogbu sirop<br>◌ Fanye ihe n'ime ikpu<br>◌ Ndi ozo<br>◌ Amaghi m<br>◌ Onweghi aziza
715b.i. Kedu ihe o mere nke wepụrụ ime ya?	(${friend2_abt_mult_yn} = 'no') or (${friend2_abt_mult_yn} = '-88')<br>◌ Usoro eji eme oparation<br>◌ Ngwá ọgwụ akpọrọ mifepristone ma ọ bụ misoprostol<br>◌ Mkpụrụ ọgwụ ị na-ewere mgbe ị nwere ahụ ọkụ dị ka ọgwụ nje ma ọ bụ ọgwụ na-emegide malarial<br>◌ Mgbochi ọgwụ mgbochi mberede<br>◌ Mkpụrụ ọgwụ, ụdị akọwapụtaghị<br>◌ Ọgwụ Ọgbugba<br>◌ uzo omenala, dika ahihia<br>◌ Mmanya<br>◌ nnu, akpu, maggi<br>◌ oroma nkịrịsị<br>◌ Nsogbu sirop<br>◌ Fanye ihe n'ime ikpu<br>◌ Ndi ozo<br>◌ Amaghi m<br>◌ Onweghi aziza
716b.i. Ebee ka ọ gara maka usoro ahụ?	(${friend2_abt_first} = 'surgery') or (${friend2_abt_only} = 'surgery')<br>◌ Ulonleta ahuike goomenti<br>◌ Senta ahuike nke goomenti<br>◌ Ulo nleta ntazi omu mu nwa<br>◌ Ulo nleta mbughari<br>◌ TBA/Fieldworker (public)<br>◌ Ulo nleta ahuike nke onwe<br>◌ Famasi<br>◌ mmiri ogwu<br>◌ Dokita onweya morbu nurse<br>◌ Dokinta Onwe<br>◌ TBA/Fieldworker (private)<br>◌ Shopu<br>◌ FBO/Church<br>◌ Ndi enyi/Ndi ezi na ulo<br>◌ NGO<br>◌ Ahia<br>◌ Ndi ozo<br>◌ Amaghi m<br>◌ Onweghi aziza
717b.i. Ebee ka o nwetara ọgwụ?	(${friend2_abt_first} = 'pills_abortion') or (${friend2_abt_only} = 'pills_abortion') or (${friend …<br>◌ Ulonleta ahuike goomenti<br>◌ Senta ahuike nke goomenti<br>◌ Ulo nleta ntazi omu mu nwa<br>◌ Ulo nleta mbughari<br>◌ TBA/Fieldworker (public)<br>◌ Ulo nleta ahuike nke onwe<br>◌ Famasi<br>◌ mmiri ogwu

	◌ Dokita onweya morbu nurse<br>◌ Dokinta Onwe<br>◌ TBA/Fieldworker (private)<br>◌ Shopu<br>◌ FBO/Church<br>◌ Ndi enyi/Ndi ezi na ulo<br>◌ NGO<br>◌ Ahia<br>◌ Ndi ozo<br>◌ Amaghi m<br>◌ Onweghi aziza
718b.i. Gịnị bụ ihe ikpeazụ ọ mere nke mechara mgbe o wepụ afọ ime ahu?	(${friend2_abt_mult_yn} = 'yes') or (${friend2_abt_mult_yn} = 'likely')<br>◌ Usoro eji eme oparation<br>◌ Ngwá ọgwụ akpọrọ mifepristone ma ọ bụ misoprostol<br>◌ Mkpụrụ ọgwụ ị na-ewere mgbe ị nwere ahụ ọkụ dị ka ọgwụ nje ma ọ bụ ọgwụ na-emegide malarial<br>◌ Mgbochi ọgwụ mgbochi mberede<br>◌ Mkpụrụ ọgwụ, ụdị akọwapụtaghị<br>◌ Ọgwụ Ọgbugba<br>◌ uzo omenala, dika ahihia<br>◌ Mmanya<br>◌ nnu, akpu, maggi<br>◌ oroma nkịrịsị<br>◌ Nsogbu sirop<br>◌ Fanye ihe n'ime ikpu<br>◌ Ndi ozo<br>◌ Amaghi m<br>◌ Onweghi aziza
719b.i. Ebee ka ọ gara maka usoro ahụ ?	${friend2_abt_last} = 'surgery'<br>◌ Ulonleta ahuike goomenti<br>◌ Senta ahuike nke goomenti<br>◌ Ulo nleta ntazi omu mu nwa<br>◌ Ulo nleta mbughari<br>◌ TBA/Fieldworker (public)<br>◌ Ulo nleta ahuike nke onwe<br>◌ Famasi<br>◌ mmiri ogwu<br>◌ Dokita onweya morbu nurse<br>◌ Dokinta Onwe<br>◌ TBA/Fieldworker (private)<br>◌ Shopu<br>◌ FBO/Church<br>◌ Ndi enyi/Ndi ezi na ulo<br>◌ NGO<br>◌ Ahia<br>◌ Ndi ozo<br>◌ Amaghi m<br>◌ Onweghi aziza
720b.i. Ebee ka o nwetara ọgwụ?	(${friend2_abt_last} = 'pills_abortion') or (${friend2_abt_last} = 'pills_fever') or (${friend2_ab …<br>◌ Ulonleta ahuike goomenti<br>◌ Senta ahuike nke goomenti<br>◌ Ulo nleta ntazi omu mu nwa<br>◌ Ulo nleta mbughari<br>◌ TBA/Fieldworker (public)<br>◌ Ulo nleta ahuike nke onwe<br>◌ Famasi<br>◌ mmiri ogwu

- Dokita onweya morbu nurse
- Dokinta Onwe
- TBA/Fieldworker (private)
- Shopu
- FBO/Church
- Ndi enyi/Ndi ezi na ulo
- NGO
- Ahia
- Ndi ozo
- Amaghi m
- Onweghi aziza

721b.i. ${friend2_name} O nwere nsogbu wee gaa ulo ogwu maka usoro iwepu afo ime mo obu imebi afo ime?

Oburu na onye-aziza ekwubugo na enyi ya gara na ulo-ogwu iji nwepu afoime ya, anyi choro ima ma enyi ya ahu aloghachiri n'ulo ogwu mgbe ozor iji gwoo ihe mgbu nwere ike iso nwepu afo ime ahu

(${friend2_abt_yn} = 'yes') or (${friend2_abt_yn} = 'likely')

- E-e, amara m nke oma
- E-e, echere m otua
- Mba
- Amaghi m
- Onweghi aziza

712b.ii. Hapu ihe ndia, Onwetugo oge ${friend2_name} mere ihe obula iji ghazi oge nso ya ngbe ochere na odi ime?

Probe to confirm whether the period regulation was successful. If not, select 'no.'

${friend2_abt_yn} = 'yes'

- E-e, amara m nke oma
- E-e, echere m otua
- Mba
- Amaghi m
- Onweghi aziza

712b.ii. Onwetugo oge ${friend2_name} mere ihe obula iji ghazi oge nso ya ngbe ochere na odi ime?

Probe to confirm whether the period regulation was successful. If not, select 'no.'

${friend2_abt_yn} != 'yes'

- E-e, amara m nke oma
- E-e, echere m otua
- Mba
- Amaghi m
- Onweghi aziza

713b.ii. Kedu na afo omere ya ikpeazụ?

If indicates happened more than once, specify most recent time.

Enter 2020 for 'Do not know' or 'No response'.

(${friend2_reg_yn} = 'yes') or (${friend2_reg_yn} = 'likely')

Year: ____________________

714b.ii. Ndị inyom mgbe ụfọdụ na-eme ọtụtụ ihe iji gbochie afo ime iga n'iru .Odi otutu ihe ${friend2_name} na - eme iji wepu ime ?

((${friend2_reg_year} > ${friend2_abt_year}) or (${friend2_abt_year} = '')) and ((${friend2_reg_yn …

- E-e, amara m nke oma
- E-e, echere m otua
- Mba
- Amaghi m
- Onweghi aziza

715b.ii. Kedu ihe mbụ o mere iji wepụ ime ya?

((${friend2_reg_year} > ${friend2_abt_year}) or (${friend2_abt_year} = '')) and ((${friend2_reg_mu …

- Usoro eji eme oparation
- Ngwá ọgwụ akpọrọ mifepristone ma ọ bụ misoprostol
- Mkpụrụ ọgwụ ị na-ewere mgbe ị nwere ahụ ọkụ dị ka ọgwụ nje ma ọ bụ ọgwụ na-emegide malarial
- Mgbochi ọgwụ mgbochi mberede
- Mkpụrụ ọgwụ, ụdị akọwapụtaghị
- Ọgwụ Ọgbugba
- uzo omenala, dika ahihia
- Mmanya
- nnu, akpu, maggi
- oroma nkịrịsị
- Nsogbu sirop
- Fanye ihe n'ime ikpu
- Ndi ozo
- Amaghi m
- Onweghi aziza

715b.ii. Kedu ihe o mere nke wepụrụ ime ya?

((${friend2_reg_year} > ${friend2_abt_year}) or (${friend2_abt_year} = '')) and ((${friend2_reg_mu …

- Usoro eji eme oparation
- Ngwá ọgwụ akpọrọ mifepristone ma ọ bụ misoprostol
- Mkpụrụ ọgwụ ị na-ewere mgbe ị nwere ahụ ọkụ dị ka ọgwụ nje ma ọ bụ ọgwụ na-emegide malarial
- Mgbochi ọgwụ mgbochi mberede
- Mkpụrụ ọgwụ, ụdị akọwapụtaghị
- Ọgwụ Ọgbugba
- uzo omenala, dika ahihia
- Mmanya
- nnu, akpu, maggi
- oroma nkịrịsị
- Nsogbu sirop
- Fanye ihe n'ime ikpu
- Ndi ozo
- Amaghi m
- Onweghi aziza

716b.ii. Ebee ka ọ gara maka usoro ahụ?

((${friend2_reg_year} > ${friend2_abt_year}) or (${friend2_abt_year} = '')) and ((${friend2_reg_fi …

- Ulonleta ahuike goomenti
- Senta ahuike nke goomenti
- Ulo nleta ntazi omu mu nwa
- Ulo nleta mbughari
- TBA/Fieldworker (public)
- Ulo nleta ahuike nke onwe
- Famasi
- mmiri ogwu
- Dokita onweya morbu nurse
- Dokinta Onwe
- TBA/Fieldworker (private)
- Shopu
- FBO/Church
- Ndi enyi/Ndi ezi na ulo
- NGO
- Ahia
- Ndi ozo
- Amaghi m
- Onweghi aziza

717b.ii. Ebee ka o nwetara ọgwụ?

((${friend2_reg_year} > ${friend2_abt_year}) or (${friend2_abt_year} = '')) and ((${friend2_reg_fi …

- Ulonleta ahuike goomenti
- Senta ahuike nke goomenti
- Ulo nleta ntazi omu mu nwa
- Ulo nleta mbughari
- TBA/Fieldworker (public)
- Ulo nleta ahuike nke onwe
- Famasi
- mmiri ogwu
- Dokita onweya morbu nurse
- Dokinta Onwe
- TBA/Fieldworker (private)
- Shopu
- FBO/Church
- Ndi enyi/Ndi ezi na ulo
- NGO
- Ahia
- Ndi ozo

	○ Amaghi m<br>○ Onweghi aziza
718b.ii. Gịnị bụ ihe ikpeazụ ọ mere nke mechara mgbe o wepụ afọ ime ahu?	((${friend2_reg_year} > ${friend2_abt_year}) or (${friend2_abt_year} = '')) and ((${friend2_reg_mu …<br>○ Usoro eji eme oparation<br>○ Ngwá ọgwụ akpọrọ mifepristone ma ọ bụ misoprostol<br>○ Mkpụrụ ọgwụ ị na-ewere mgbe ị nwere ahụ ọkụ dị ka ọgwụ nje ma ọ bụ ọgwụ na-emegide malarial<br>○ Mgbochi ọgwụ mgbochi mberede<br>○ Mkpụrụ ọgwụ, ụdị akọwapụtaghị<br>○ Ọgwụ Ọgbugba<br>○ uzo omenala, dika ahihia<br>○ Mmanya<br>○ nnu, akpu, maggi<br>○ oroma nkịrịsị<br>○ Nsogbu sirop<br>○ Fanye ihe n'ime ikpu<br>○ Ndi ozo<br>○ Amaghi m<br>○ Onweghi aziza
719b.ii. Ebee ka ọ gara maka usoro ahụ ?	((${friend2_reg_year} > ${friend2_abt_year}) or (${friend2_abt_year} = '')) and (${friend2_reg_las …<br>○ Ulonleta ahuike goomenti<br>○ Senta ahuike nke goomenti<br>○ Ulo nleta ntazi omu mu nwa<br>○ Ulo nleta mbughari<br>○ TBA/Fieldworker (public)<br>○ Ulo nleta ahuike nke onwe<br>○ Famasi<br>○ mmiri ogwu<br>○ Dokita onweya morbu nurse<br>○ Dokinta Onwe<br>○ TBA/Fieldworker (private)<br>○ Shopu<br>○ FBO/Church<br>○ Ndi enyi/Ndi ezi na ulo<br>○ NGO<br>○ Ahia<br>○ Ndi ozo<br>○ Amaghi m<br>○ Onweghi aziza
720b.ii. Ebee ka o nwetara ọgwụ?	((${friend2_reg_year} > ${friend2_abt_year}) or (${friend2_abt_year} = '')) and ((${friend2_reg_la …<br>○ Ulonleta ahuike goomenti<br>○ Senta ahuike nke goomenti<br>○ Ulo nleta ntazi omu mu nwa<br>○ Ulo nleta mbughari<br>○ TBA/Fieldworker (public)<br>○ Ulo nleta ahuike nke onwe<br>○ Famasi<br>○ mmiri ogwu<br>○ Dokita onweya morbu nurse<br>○ Dokinta Onwe<br>○ TBA/Fieldworker (private)<br>○ Shopu<br>○ FBO/Church<br>○ Ndi enyi/Ndi ezi na ulo<br>○ NGO

	◌ Ahia<br>◌ Ndi ozo<br>◌ Amaghi m<br>◌ Onweghi aziza
721b.ii. ${friend2_name} O nwere nsogbu wee gaa ulo ogwu maka usoro iwepu afo ime mo obu imebi afo ime?<br>*If the respondent already reported the friend went to a health facility in the process of regulating her period, we are interested in whether the friend went back to a health facility on a separate occasion to treat complications that she may have experienced.*	((${friend2_reg_year} > ${friend2_abt_year}) or (${friend2_abt_year} = '')) and ((${friend2_reg_yn …<br>◌ E-e, amara m nke oma<br>◌ E-e, echere m otua<br>◌ Mba<br>◌ Amaghi m<br>◌ Onweghi aziza
722a. Onwego ihe mere iji wepụ afo ime mgbe ọ dị ime ma ọ bụ chee na ọ dị ime?<br>*Probe to confirm whether the pregnancy removal was successful.*	◌ E-e<br>◌ Mba<br>◌ Onweghi aziza
723a. Kedu n'afọ nkea mere ikpeazụ?<br>*If indicates happened more than once, specify most recent time.*<br>*Enter 2020 for 'Do not know' or 'No response'.*	(${self_abt_yn} = 'yes')<br>Year: ______________
724a. Odi otutu ihe imere iji wepu ime ma?	(${self_abt_yn} = 'yes')<br>◌ E-e<br>◌ Mba<br>◌ Onweghi aziza
725a. Kedu ihe mbo imere?	(${self_abt_mult_yn} = 'yes')<br>◌ Usoro eji eme oparation<br>◌ Ngwá ọgwụ akpọrọ mifepristone ma ọ bụ misoprostol<br>◌ Mkpụrụ ọgwụ ị na-ewere mgbe ị nwere ahụ ọkụ dị ka ọgwụ nje ma ọ bụ ọgwụ na-emegide malarial<br>◌ Mgbochi ọgwụ mgbochi mberede<br>◌ Mkpụrụ ọgwụ, ụdị akọwapụtaghị<br>◌ Ọgwụ Ọgbugba<br>◌ uzo omenala, dika ahihia<br>◌ Mmanya<br>◌ nnu, akpu, maggi<br>◌ oroma nkịrịsị<br>◌ Nsogbu sirop<br>◌ Fanye ihe n'ime ikpu<br>◌ Ndi ozo<br>◌ Amaghi m<br>◌ Onweghi aziza
725a. Kedu ihe ị mere?	(${self_abt_mult_yn} = 'no')<br>◌ Usoro eji eme oparation<br>◌ Ngwá ọgwụ akpọrọ mifepristone ma ọ bụ misoprostol<br>◌ Mkpụrụ ọgwụ ị na-ewere mgbe ị nwere ahụ ọkụ dị ka ọgwụ nje ma ọ bụ ọgwụ na-emegide malarial<br>◌ Mgbochi ọgwụ mgbochi mberede<br>◌ Mkpụrụ ọgwụ, ụdị akọwapụtaghị<br>◌ Ọgwụ Ọgbugba<br>◌ uzo omenala, dika ahihia<br>◌ Mmanya<br>◌ nnu, akpu, maggi<br>◌ oroma nkịrịsị<br>◌ Nsogbu sirop<br>◌ Fanye ihe n'ime ikpu<br>◌ Ndi ozo<br>◌ Amaghi m<br>◌ Onweghi aziza
726a. Ebee ka i gara maka usoro ahụ?	(${self_abt_first} = 'surgery') or (${self_abt_only} = 'surgery')

	◌ Ulonleta ahuike goomenti<br>◌ Senta ahuike nke goomenti<br>◌ Ulo nleta ntazi omu mu nwa<br>◌ Ulo nleta mbughari<br>◌ TBA/Fieldworker (public)<br>◌ Ulo nleta ahuike nke onwe<br>◌ Famasi<br>◌ mmiri ogwu<br>◌ Dokita onweya morbu nurse<br>◌ Dokinta Onwe<br>◌ TBA/Fieldworker (private)<br>◌ Shopu<br>◌ FBO/Church<br>◌ Ndi enyi/Ndi ezi na ulo<br>◌ NGO<br>◌ Ahia<br>◌ Ndi ozo<br>◌ Amaghi m<br>◌ Onweghi aziza
727a. Ebee ka ị nwetara ọgwụ?	(${self_abt_first} = 'pills_abortion') or (${self_abt_only} = 'pills_abortion') or (${self_abt_fir …<br>◌ Ulonleta ahuike goomenti<br>◌ Senta ahuike nke goomenti<br>◌ Ulo nleta ntazi omu mu nwa<br>◌ Ulo nleta mbughari<br>◌ TBA/Fieldworker (public)<br>◌ Ulo nleta ahuike nke onwe<br>◌ Famasi<br>◌ mmiri ogwu<br>◌ Dokita onweya morbu nurse<br>◌ Dokinta Onwe<br>◌ TBA/Fieldworker (private)<br>◌ Shopu<br>◌ FBO/Church<br>◌ Ndi enyi/Ndi ezi na ulo<br>◌ NGO<br>◌ Ahia<br>◌ Ndi ozo<br>◌ Amaghi m<br>◌ Onweghi aziza
728a. Kedu ihe ikpeazụ ị mere iji wepu ime ahụ?	(${self_abt_mult_yn} = 'yes')<br>◌ Usoro eji eme oparation<br>◌ Ngwá ọgwụ akpọrọ mifepristone ma ọ bụ misoprostol<br>◌ Mkpụrụ ọgwụ ị na-ewere mgbe ị nwere ahụ ọkụ dị ka ọgwụ nje ma ọ bụ ọgwụ na-emegide malarial<br>◌ Mgbochi ọgwụ mgbochi mberede<br>◌ Mkpụrụ ọgwụ, ụdị akọwapụtaghị<br>◌ Ọgwụ Ọgbugba<br>◌ uzo omenala, dika ahihia<br>◌ Mmanya<br>◌ nnu, akpu, maggi<br>◌ oroma nkịrịsị<br>◌ Nsogbu sirop<br>◌ Fanye ihe n'ime ikpu<br>◌ Ndi ozo<br>◌ Amaghi m<br>◌ Onweghi aziza
729a. Ebee ka ị gara maka usoro ahụ?	${self_abt_last} = 'surgery'<br>◌ Ulonleta ahuike goomenti

	○ Senta ahuike nke goomenti<br>○ Ulo nleta ntazi omu mu nwa<br>○ Ulo nleta mbughari<br>○ TBA/Fieldworker (public)<br>○ Ulo nleta ahuike nke onwe<br>○ Famasi<br>○ mmiri ogwu<br>○ Dokita onweya morbu nurse<br>○ Dokinta Onwe<br>○ TBA/Fieldworker (private)<br>○ Shopu<br>○ FBO/Church<br>○ Ndi enyi/Ndi ezi na ulo<br>○ NGO<br>○ Ahia<br>○ Ndi ozo<br>○ Amaghi m<br>○ Onweghi aziza
730a. Ebee ka ị nwetara ọgwụ?	(${self_abt_last} = 'pills_abortion') or (${self_abt_last} = 'pills_fever') or (${self_abt_last} = …<br>○ Ulonleta ahuike goomenti<br>○ Senta ahuike nke goomenti<br>○ Ulo nleta ntazi omu mu nwa<br>○ Ulo nleta mbughari<br>○ TBA/Fieldworker (public)<br>○ Ulo nleta ahuike nke onwe<br>○ Famasi<br>○ mmiri ogwu<br>○ Dokita onweya morbu nurse<br>○ Dokinta Onwe<br>○ TBA/Fieldworker (private)<br>○ Shopu<br>○ FBO/Church<br>○ Ndi enyi/Ndi ezi na ulo<br>○ NGO<br>○ Ahia<br>○ Ndi ozo<br>○ Amaghi m<br>○ Onweghi aziza
731a. I nwere nsogbu wee gaa ulo ogwu maka usoro iwepu afo ime mo obu imebi afo ime?<br>*If the respondent already reported she went to a health facility in the process of removing the pregnancy, we are interested in whether she went back to a health facility on a separate occasion to treat complications that she may have experienced.*	(${self_abt_yn} = 'yes')<br>○ E-e<br>○ Mba<br>○ Amaghi m<br>○ Onweghi aziza
732a. Ònye ka ị gwara banyere onodu a?<br>*Read the answer choices aloud. Select all that apply.*	(${self_abt_yn} = 'yes')<br>☐ Di / nwoke ibe<br>☐ Nwanna nwanyị<br>☐ Nwanne nwoke<br>☐ Nne<br>☐ Nna<br>☐ Onye ikwu ndị ọzọ<br>☐ Enyi nke mbu: ${friend1_name}<br>☐ Enyi nke mbu: ${friend2_name}<br>☐ Enyi ọzọ<br>☐ Ndi ozo<br>☐ Amaghi m<br>☐ Onweghi aziza<br>(${friend1_name} != '' and ${friend1_name} != '-99' and filter = 'friend1') or (${friend2_name} != '' and ${friend2_name} != '-99' and filter =

722b. Hapu ihe ndia nile, Onwetugo mgbe I mere ihe obula gbasara oge nso gi mgbe ichere na Idi ime.<br>*Probe to confirm whether the period regulation was successful. If not, select 'no.'*	'friend2') or (filter = 'always')<br>${self_abt_yn} = 'yes'<br>○ E-e<br>○ Mba<br>○ Onweghi aziza
722b. Onwetugo mgbe I mere ihe obula gbasara oge nso gi mgbe ichere na Idi ime.<br>*Probe to confirm whether the period regulation was successful. If not, select 'no.'*	${self_abt_yn} != 'yes'<br>○ E-e<br>○ Mba<br>○ Onweghi aziza
723b. Kedu n'afọ nkea mere ikpeazụ?<br>*If indicates happened more than once, specify most recent time.*<br>*Enter 2020 for 'Do not know' or 'No response'.*	(${self_reg_yn} = 'yes')<br>Year: ___________________________
724b. Odi otutu ihe imere iji wepu ime ma?	((${self_reg_year} > ${self_abt_year}) or (${self_abt_year} = '')) and ((${self_reg_yn} = 'yes'))<br>○ E-e<br>○ Mba<br>○ Onweghi aziza
725b. Kedu ihe mbo imere?	((${self_reg_year} > ${self_abt_year}) or (${self_abt_year} = '')) and ((${self_reg_mult_yn} = 'ye …<br>○ Usoro eji eme oparation<br>○ Ngwá ọgwụ akpọrọ mifepristone ma ọ bụ misoprostol<br>○ Mkpụrụ ọgwụ ị na-ewere mgbe ị nwere ahụ ọkụ dị ka ọgwụ nje ma ọ bụ ọgwụ na-emegide malarial<br>○ Mgbochi ọgwụ mgbochi mberede<br>○ Mkpụrụ ọgwụ, ụdị akọwapụtaghị<br>○ Ọgwụ Ọgbugba<br>○ uzo omenala, dika ahihia<br>○ Mmanya<br>○ nnu, akpu, maggi<br>○ oroma nkịrịsị<br>○ Nsogbu sirop<br>○ Fanye ihe n'ime ikpu<br>○ Ndi ozo<br>○ Amaghi m<br>○ Onweghi aziza
725b. Kedu ihe ị mere?	((${self_reg_year} > ${self_abt_year}) or (${self_abt_year} = '')) and ((${self_reg_mult_yn} = 'no …<br>○ Usoro eji eme oparation<br>○ Ngwá ọgwụ akpọrọ mifepristone ma ọ bụ misoprostol<br>○ Mkpụrụ ọgwụ ị na-ewere mgbe ị nwere ahụ ọkụ dị ka ọgwụ nje ma ọ bụ ọgwụ na-emegide malarial<br>○ Mgbochi ọgwụ mgbochi mberede<br>○ Mkpụrụ ọgwụ, ụdị akọwapụtaghị<br>○ Ọgwụ Ọgbugba<br>○ uzo omenala, dika ahihia<br>○ Mmanya<br>○ nnu, akpu, maggi<br>○ oroma nkịrịsị<br>○ Nsogbu sirop<br>○ Fanye ihe n'ime ikpu<br>○ Ndi ozo<br>○ Amaghi m<br>○ Onweghi aziza
726b. Ebee ka i gara maka usoro ahụ?	((${self_reg_year} > ${self_abt_year}) or (${self_abt_year} = '')) and ((${self_reg_first} = 'surg …<br>○ Ulonleta ahuike goomenti

- Senta ahuike nke goomenti
- Ulo nleta ntazi omu mu nwa
- Ulo nleta mbughari
- TBA/Fieldworker (public)
- Ulo nleta ahuike nke onwe
- Famasi
- mmiri ogwu
- Dokita onweya morbu nurse
- Dokinta Onwe
- TBA/Fieldworker (private)
- Shopu
- FBO/Church
- Ndi enyi/Ndi ezi na ulo
- NGO
- Ahia
- Ndi ozo
- Amaghi m
- Onweghi aziza

727b. Ebee ka ị nwetara ọgwụ?

((${self_reg_year} > ${self_abt_year}) or (${self_abt_year} = '')) and ((${self_reg_first} = 'pill …

- Ulonleta ahuike goomenti
- Senta ahuike nke goomenti
- Ulo nleta ntazi omu mu nwa
- Ulo nleta mbughari
- TBA/Fieldworker (public)
- Ulo nleta ahuike nke onwe
- Famasi
- mmiri ogwu
- Dokita onweya morbu nurse
- Dokinta Onwe
- TBA/Fieldworker (private)
- Shopu
- FBO/Church
- Ndi enyi/Ndi ezi na ulo
- NGO
- Ahia
- Ndi ozo
- Amaghi m
- Onweghi aziza

728b. Kedu ihe ikpeazụ ị mere iji wepu ime ahụ?

((${self_reg_year} > ${self_abt_year}) or (${self_abt_year} = '')) and ((${self_reg_mult_yn} = 'ye …

- Usoro eji eme oparation
- Ngwá ọgwụ akpọrọ mifepristone ma ọ bụ misoprostol
- Mkpụrụ ọgwụ ị na-ewere mgbe ị nwere ahụ ọkụ dị ka ọgwụ nje ma ọ bụ ọgwụ na-emegide malarial
- Mgbochi ọgwụ mgbochi mberede
- Mkpụrụ ọgwụ, ụdị akọwapụtaghị
- Ọgwụ Ọgbugba
- uzo omenala, dika ahihia
- Mmanya
- nnu, akpu, maggi
- oroma nkịrịsị
- Nsogbu sirop
- Fanye ihe n'ime ikpu
- Ndi ozo
- Amaghi m
- Onweghi aziza

729b. Ebee ka ị gara maka usoro ahụ?

((${self_reg_year} > ${self_abt_year}) or

	(${self_abt_year} = '')) and (${self_reg_last} = 'surger …<br>◯ Ulonleta ahuike goomenti<br>◯ Senta ahuike nke goomenti<br>◯ Ulo nleta ntazi omu mu nwa<br>◯ Ulo nleta mbughari<br>◯ TBA/Fieldworker (public)<br>◯ Ulo nleta ahuike nke onwe<br>◯ Famasi<br>◯ mmiri ogwu<br>◯ Dokita onweya morbu nurse<br>◯ Dokinta Onwe<br>◯ TBA/Fieldworker (private)<br>◯ Shopu<br>◯ FBO/Church<br>◯ Ndi enyi/Ndi ezi na ulo<br>◯ NGO<br>◯ Ahia<br>◯ Ndi ozo<br>◯ Amaghi m<br>◯ Onweghi aziza
730b. Ebee ka ị nwetara ọgwụ?	((${self_reg_year} > ${self_abt_year}) or (${self_abt_year} = '')) and ((${self_reg_last} = 'pills …<br>◯ Ulonleta ahuike goomenti<br>◯ Senta ahuike nke goomenti<br>◯ Ulo nleta ntazi omu mu nwa<br>◯ Ulo nleta mbughari<br>◯ TBA/Fieldworker (public)<br>◯ Ulo nleta ahuike nke onwe<br>◯ Famasi<br>◯ mmiri ogwu<br>◯ Dokita onweya morbu nurse<br>◯ Dokinta Onwe<br>◯ TBA/Fieldworker (private)<br>◯ Shopu<br>◯ FBO/Church<br>◯ Ndi enyi/Ndi ezi na ulo<br>◯ NGO<br>◯ Ahia<br>◯ Ndi ozo<br>◯ Amaghi m<br>◯ Onweghi aziza
731b. I nwere nsogbu wee gaa ulo ogwu maka usoro iwepu afo ime mo obu imebi afo ime?<br>*If the respondent already reported she went to a health facility in the process of regulating her period, we are interested in whether she went back to a health facility on a separate occasion to treat complications that she may have experienced.*	((${self_reg_year} > ${self_abt_year}) or (${self_abt_year} = '')) and ((${self_reg_yn} = 'yes'))<br>◯ E-e<br>◯ Mba<br>◯ Amaghi m<br>◯ Onweghi aziza
732b. Ònye ka ị gwara banyere onodu a?<br>*Read the answer choices aloud. Select all that apply.*	((${self_reg_year} > ${self_abt_year}) or (${self_abt_year} = '')) and ((${self_reg_yn} = 'yes'))<br>☐ Di / nwoke ibe<br>☐ Nwanna nwanyị<br>☐ Nwanne nwoke<br>☐ Nne<br>☐ Nna<br>☐ Onye ikwu ndị ọzọ<br>☐ Enyi nke mbu: ${friend1_name}<br>☐ Enyi nke mbu: ${friend2_name}<br>☐ Enyi ọzọ<br>☐ Ndi ozo

	☐ Amaghi m<br>☐ Onweghi aziza<br>(${friend1_name} != '' and ${friend1_name} != '-99' and filter = 'friend1') or (${friend2_name} != '' and ${friend2_name} != '-99' and filter = 'friend2') or (filter = 'always')

	${consent_obtained}
Ugbu anyi choro iju gi ajuju ndi ozo gbasara iwepu afo ime na out esi ahuta nwanyi wepuru afo ime ma o bu nke haziri oge ya<br>*Check box to confirm scrolled to bottom.*	
Pia Ok ka iga n'iru	○ o di mma

733. Ọ dị mma na nwanyị ga ewepu ime n'hi onodu ahu ike na ize ndụ.	${consent_obtained}<br>○ Ekwenyere m<br>○ Ekwenyeresiri m ike<br>○ Ekwenyeghi ma obu kwenye<br>○ Ekweghi m<br>○ Ajusiri m ike<br>○ Onweghi aziza
734. Ọ dị mma na nwanyị ga ewepu ime ma ọ bụrụ na afọ ime ahu bụ nke sitere na mmeko nwoke na aka ike.	${consent_obtained}<br>○ Ekwenyere m<br>○ Ekwenyeresiri m ike<br>○ Ekwenyeghi ma obu kwenye<br>○ Ekweghi m<br>○ Ajusiri m ike<br>○ Onweghi aziza
735. Ọ dị mma na nwanyị ga-ete ime ma ọ bụrụ na ọ gaghị enwe ike ịzụ nwa ahu	${consent_obtained}<br>○ Ekwenyere m<br>○ Ekwenyeresiri m ike<br>○ Ekwenyeghi ma obu kwenye<br>○ Ekweghi m<br>○ Ajusiri m ike<br>○ Onweghi aziza
736. Nwanyị nke na-ete ime na-ewetara ezi na ụlọ ya ihere.	${consent_obtained}<br>○ Ekwenyere m<br>○ Ekwenyeresiri m ike<br>○ Ekwenyeghi ma obu kwenye<br>○ Ekweghi m<br>○ Ajusiri m ike<br>○ Onweghi aziza
737. Nwanyị nke na-ete ime na-ewetara ezi na ụlọ ya ihere.	${consent_obtained}<br>○ Ekwenyere m<br>○ Ekwenyeresiri m ike<br>○ Ekwenyeghi ma obu kwenye<br>○ Ekweghi m<br>○ Ajusiri m ike<br>○ Onweghi aziza
738. Nwanyị nke na-ete ime ekwesighi igwa onye ọ bụla.	${consent_obtained}<br>○ Ekwenyere m<br>○ Ekwenyeresiri m ike<br>○ Ekwenyeghi ma obu kwenye<br>○ Ekweghi m<br>○ Ajusiri m ike<br>○ Onweghi aziza

Follow-up Consent.

FLW_801. Daalụ maka oge ị ji obiọma nye anyị.<br>Anyị nwere mmasị ịnụkwu banyere ahụmịhe ime gị, tinyere mkpebi gị na usoro usoro ị ga-eji na ebe ị ga-aga maka ọrụ, n'etiti ihe ndị ọzọ. Ị ga-adị njikere izute nwanyị ọzọ site na oru ahụ na ụbọchị ikpeazụ iji kwurịta banyere ahụmịhe a	(${self_abt_yn} = 'yes') or (${self_reg_yn} = 'yes')<br>○ E-e<br>○ Mba<br>○ Onweghi aziza

FLW_802. I nwere ekwentị?	${flw_willing} = 'yes'<br>○ E-e<br>○ Mba<br>○ Onweghi aziza
FLW_803. Enwere m ike ịnweta akara ekwentị gị ma ọ bụrụ na anyị ga - achọ ikpo gị n'ọdịnihu?<br>*Enter an 11-digit number without the country code. Do not include spaces or dashes. Enter 0 for no response.*	${flw_number_yn} = 'yes'<br>--
FLW_804. Inwere ike ikpoghari akara ekwenti gi ahu?<br>*Enter an 11-digit number without the country code. Do not include spaces or dashes. Enter 0 for no response.*	(${flw_number_typed} != '') and (${flw_number_typed} != '0')<br>--
Kele e onye aziza maka oge ya<br>*The respondent is finished, but there are still more questions for you to complete outside the home.*	${available} = 'yes'

Location and Questionnaire result

095. Ebe ana-eme nyocha<br>*Take a GPS point near the entrance to the household. Record location when the accuracy is smaller than 6 m.*	Mgbe niile
096. Ugboro ole ka ikpoturu ezi na ulo maka iju onye aziza nwanyi ajuju?	Mgbe niile<br>○ Oge nke izizi<br>○ Oge nke abua.<br>○ Oge nke ato
097. Kedu asusu ejiri wey jua ajujua?	009a = 1<br>○ Oyibo<br>○ Hausa<br>○ Igbo<br>○ Yoruba<br>○ Pidgin<br>○ Ndi ozo
