# Supplementary material for: Measurement of abortion safety using community-based surveys: Findings from three countries
Source: PLoS One. 2019 Nov 7;14(11):e0223146. doi: 10.1371/journal.pone.0223146 (PMC6837422; doi:10.1371/journal.pone.0223146)
Supplement: S4 Doc — (PDF) [file pone.0223146.s004.pdf]

S4 Doc.

NGR5-Female-Questionnaire-v36-aso.xlsx

001a. You dey for the correct household? EA: [EA entered in the Household Questionnaire] Structure #: [Structure entered in the Household Questionnaire] Household #: [Household entered in the Household Questionnaire]	Always<br>○ Yes<br>○ No
002. Enter your name below.<br>*Please record your name*	002 = 0<br>--
003b. Record the correct date and time.	003 = 0<br>Day: ----------------------------------<br>Month: ----------------------------------<br>Year: ----------------------------------

The following information is from the Household Questionnaire. Please review to make sure you are interviewing the correct respondent. [ODK will display the State, LGA, Enumeration Area, Structure Number, and Household Number entered into the Household Questionnaire linked to this Female Questionnaire.] Is the above information correct?	Always
State: ${level1_unlinked}	State: [STATE]<br>--
LGA: ${level2_unlinked}	LGA: [LGA]<br>--
Locality: ${level3_unlinked}	Locality: [LOCALITY]<br>--
Enumeration Area: [EA]	--
Structure number: [#]	--
Household number: [#]	--
004b. Is the above information correct?	004 = 0<br>○ Yes<br>○ No

005. CHECK: Na [Name of the interviewee] you dey wan interview now. E correct so?<br>*If misspelled, select "yes" and update the name in question "011."*<br>*If this is the wrong person, you have two options:*<br>*(1) exit and ignore changes to this form. Open the correct form.*<br>*Or*<br>*(2) find and interview the person whose name appears above.*	Always<br>○ Yes<br>○ No
006. Is the respondent present and available to be interviewed today?	Always<br>○ Yes<br>○ No
007. How well acquainted are you with the respondent?	006 = 1<br>○ Sabi the person wella<br>○ Well acquainted<br>○ Not well acquainted<br>○ Not acquainted
008. Has the respondent previously participated in PMA 2020 surveys?	006 = 1<br>○ Yes<br>○ No<br>○ Do not know<br>○ e no ansa
INFORMED CONSENT<br>*Find the woman wey her age don reach 15 and e never pass 49 for this female questionnaire. The interview wey you one do other people no suppose hear am. Oya read the salute:*	(${available} = 'yes') and (not(${unlinked}) or ${proceed_with_unlinked})
Hello. My name is ________________________________ and I am working for the Center for Research, Evaluation Resources,	(${available} = 'yes') and (not(${unlinked}) or ${proceed_with_unlinked})

and Development in collaboration with the Centre for Advanced Medical Research and Training. We are conducting a local survey about various health issues. We would very much appreciate your participation in this survey. This information will help us inform the government to better plan health services. Whatever information you provide will be kept strictly confidential and will not be shown to anyone other than members of our survey team.<br>Participation in this survey is voluntary, and if we should come to any question you don't want to answer, just let me know and I will go on to the next question; or you can stop the interview at any time. However, we hope that you will participate in this survey since your views are important.<br>I am going to ask you questions about your family and other household members. We would then like to ask a different set of questions to female members of this household who are between the ages of 15 and 49 years.<br>If you have any questions about the study and your right as a research participant, you may ask me now or you may also contact Dr. Elizabeth Omoluobi at Center for Research, Evaluation Resources and Development in Ile-ife, Nigeria at +2348033816486. At this time, do you want to ask me anything about the survey?	
009a. I fit start to talk now?	(${available} = 'yes') and (not(${unlinked}) or ${proceed_with_unlinked})<br>○ Yes<br>○ No
010. Interviewer's name Abeg type your name as witness to this agreement. You been enter "[Interviewer's name]."	${consent_obtained} and (${your_name_check} = 'no')<br>________________

Section 1 – Respondent's Background, Marital Status, Household characteristics

Now I wan ask about your life, where you from come and how you don take dey manage yourself.

	${consent_obtained}
101. Which month and year they born you? The age for the household roster na [AGE]<br>*Select 'Do not know' for month and '2020' for year to indicate 'No Response'.*	009a = 1
101. In what month and year were you born?<br>*Select 'Do not know' for month and '2020' for year to indicate 'No Response'.*	009a = 1
Month:	○ January<br>○ February<br>○ March<br>○ April<br>○ May<br>○ June<br>○ July<br>○ August<br>○ September<br>○ October<br>○ November<br>○ December<br>○ Do not know
Year:	Year: ________________

102. How many years you be for your last birthday?	009a = 1<br>________________
103. Wia you school reach?<br>*Only record formal schooling. Do not record bible or koranic school or short courses.*	009a = 1<br>○ I no go at all<br>○ Primary<br>○ Secondary<br>○ Higher

	○ e no ansa
104. You don marry or you dey live wit man as if say you don marry?<br>*Probe: if answer na NO, ask the woan wether she no dey inside marriage again or her husband don die.*	009a = 1<br>○ Yes, I dey inside marriage<br>○ Yes, I dey live with man<br>○ I no dey inside marriage now: Court don separate us/ I no dey with my husband again<br>○ I no dey with any body:My husband don die<br>○ Never ever marry before<br>○ e no ansa
105. You don marry or live wit man once or more than once?	104 ≠ 5<br>○ Only once<br>○ Pass once<br>○ e no ansa

	(${marriage_history} = 'more_than_once')
106a. Which months and year you start to live wit your FIRST husband / partner?<br>*Select 'Do not know' for month and '2020' for year to indicate 'No Response'.*	105=2
Month:	○ January<br>○ February<br>○ March<br>○ April<br>○ May<br>○ June<br>○ July<br>○ August<br>○ September<br>○ October<br>○ November<br>○ December<br>○ Do not know
Year:	Year: ________________

106b. CHECK: Based on the response you entered in 106a, the respondent was possibly 15 years old or younger at the time of her first marriage.<br>Did you enter 106a correctly?	age at marriage ≤15<br>○ Yes<br>○ No

	(${marriage_history} = 'once') or (${marriage_history} = 'more_than_once')
107a. Now I go like ask about wen you begin live with your CURRENT or MOST RECENT husband / partner. Which month and year be that?<br>*Select 'Do not know' for month and '2020' for year to indicate 'No Response'.*	105 = 1 or 2
Month:	○ January<br>○ February<br>○ March<br>○ April<br>○ May<br>○ June<br>○ July<br>○ August<br>○ September<br>○ October<br>○ November<br>○ December<br>○ Do not know
Year:	Year: ________________

107b. CHECK: Based on the response you entered in 107a, the respondent was possibly 15 years old or younger at the time of her current or most recent marriage.<br>Did you enter 107a correctly?	107a age at marriage ≤15<br>◯ Yes<br>◯ No
108. This your husband / partner get other wives or he dey live wit other women as if he married dem?	104 = 1 or 2<br>◯ Yes<br>◯ No<br>◯ Do not know<br>◯ e no ansa

Section 2 – Reproduction, Pregnancy & Fertility Preferences

Now I go like to ask about all the times u don born pikin for your life.

200. Now I go like to ask about all the pikin wey you don ever born for life. You don ever born?	009a = 1<br>◯ Yes<br>◯ No<br>◯ e no ansa
201. How many times you don born?<br>*Write -99 if she no answer*	200 = 1<br>____________________

	(${birth_events} > 1)
205. Which time you FIRST born pikin?<br>*Abeg record the date of the FIRST birth. The date should be found by calculating backwards from memorable events if needed.*<br>*Select 'Do not know' for month and '2020' for year to indicate 'No Response'.*	201 > 1
Month:	◯ January<br>◯ February<br>◯ March<br>◯ April<br>◯ May<br>◯ June<br>◯ July<br>◯ August<br>◯ September<br>◯ October<br>◯ November<br>◯ December<br>◯ Do not know
Year:	Year: ____________________

	(${birth_events} > 0)
206. When was your MOST RECENT birth?<br>*Select 'Do not know' for month and '2020' for year to indicate 'No Response'.*	201 > 1
Month:	◯ January<br>◯ February<br>◯ March<br>◯ April<br>◯ May<br>◯ June<br>◯ July<br>◯ August<br>◯ September<br>◯ October<br>◯ November<br>◯ December<br>◯ Do not know
Year:	Year: ____________________

210a. You get belle now?	${consent_obtained}<br>◯ Yes

	◯ No<br>◯ Unsure<br>◯ e no ansa

	${pregnant} = 'yes'
210b. How many months belle you get?	210a = 1
The most recent birth was: [Date of most recent birth]	${recent_birth} != ''
#####<br>*Please record the number of completed months. Enter -88 for do not know, -99 for No response.*	--

209. Which time your last menstrual period start?<br>*If you select days, weeks, months, or years, you will enter a number for X on the next screen.*	009a = 1<br>◯ X days ago<br>◯ X weeks ago<br>◯ X months ago<br>◯ X years ago<br>◯ She don stop kpata kpata to see her period/ or she don comot her womb<br>◯ Before pikin e born last<br>◯ She never see period before for her life<br>◯ e no ansa
209a. Enter [Menstrual Period]<br>*Enter 0 days for today, not 0 weeks/months/years.*	(${menstrual_period} = 'days') or (${menstrual_period} = 'weeks') or (${menstrual_period} = 'month …<br>--

	${ever_birth} = 'yes' or ${pregnant} = 'yes'
213a. Now I go like ask about your last pikin.	201 > 0 AND 210a ≠ 1 201 > 0 AND 210a ≠ 1 201 > 0 AND 210a ≠ 1
213b. Now I go like ask question about this belle you get.	210a = 1
The time you get belle, you bin wan get belle that time, did you want to wait until later, or you now wan get pikin at all?	(${birth_events} > 1 and ${pregnant} != 'yes') or (${ever_birth} = 'yes' and ${pregnant} = 'yes')
The time you get belle, you bin wan get belle that time, did you want to wait until later, or you now wan get pikin at all?	(${birth_events} = 1 and ${pregnant} != 'yes') or ((${ever_birth} = 'no') and (${pregnant} = 'yes'))
#####	◯ Then<br>◯ Later<br>◯ Not at all<br>◯ e no ansa

Now I have some questions about the future.	${consent_obtained}
211a. You go like to get pikin or you no wat to get pikin?	210a ≠ 1<br>◯ Have a child<br>◯ Prefer no children<br>◯ Says she can't get pregnant<br>◯ Never reason am/ no know<br>◯ e no ansa
211a. You go like to get pikin or you no wat to get pikin?	210a ≠ 1<br>◯ Have another child<br>◯ e don finish<br>◯ Says she can't get pregnant<br>◯ Never reason am/ no know<br>◯ e no ansa
211b. After the pikin wey you wan born, you go like to get another pikin or you no wan get another pikin?	210a = 1<br>◯ Have another child<br>◯ e don finish<br>◯ Says she can't get pregnant<br>◯ Never reason am/ no know<br>◯ e no ansa
212a. How long you wan na wait for now to born another pikin?<br>*If you select months or years, you will enter a number for X on the next screen.*	211a = 1<br>◯ X months

Select "Years" if more than 36 months.<br>*Please check that you correctly entered the value for months/years.*	○ X years<br>○ Soon/now<br>○ Says she can't get pregnant<br>○ Other<br>○ Don't know<br>○ e no ansa
212b. After you born this pikin you dey expect, how long you go wan wait before you born another pikin?<br>*If you select months or years, you will enter a number for X on the next screen.*<br>*Select "Years" if more than 36 months.*<br>*Please check that you correctly entered the value for months/years.*	211b = 1<br>○ X months<br>○ X years<br>○ Soon/now<br>○ Says she can't get pregnant<br>○ Other<br>○ Don't know<br>○ e no ansa
212c. Enter the number of [Months OR Years] wey you go like to wait:	${wait_birth_none} = 'months' or ${wait_birth_some} = 'months' or ${wait_birth_pregnant} = 'mont …<br>---

Section 3 – Contraception

Now I go like to talk about family planning - the various ways or methods that a couple fit use to delay or avoid a pregnancy.
An image will appear on the screen for some methods. If the respondent says that she has not heard of the method or if she hesitates to answer, read the probe aloud and show her the image, if available.

301a. You don ever hear of female sterilization?<br>PROBE: Women can have an operation to avoid having any more children.	009a = 1<br>○ Yes<br>○ No<br>○ e no ansa
301b. You don ever hear of male sterilization?<br>PROBE: Men can have an operation to avoid having any more children.	009a = 1<br>○ Yes<br>○ No<br>○ e no ansa
301c. You don ever hear of the contraceptive implant?<br>PROBE: Women can have one or several small rods placed in her upper arm by a doctor or nurse, which can prevent pregnancy for one or more years.<br>[implant_150x300.png]	009a = 1<br>○ Yes<br>○ No<br>○ e no ansa
301d. You don ever hear of the IUD?<br>PROBE: Women can have a loop or coil placed inside them by a doctor or a nurse.<br>[IUD_150x300.png]	009a = 1<br>○ Yes<br>○ No<br>○ e no ansa
301e. You don ever hear of injectables?<br>PROBE: Women fit get injection from people wey dey work for hospital wey go fit stop make dem get belle.<br>[sayana_depo_150x300.jpg]	009a = 1<br>○ Yes<br>○ No<br>○ e no ansa
301f. You don ever hear of the (birth control) pill?<br>PROBE: Women dey take tablet everyday make she no carry belle.<br>[pill_150x300.png]	009a = 1<br>○ Yes<br>○ No<br>○ e no ansa
301g. You don ever hear of emergency contraception?<br>PROBE: Tablet wey women dey take immidiately after dey meet man.	009a = 1<br>○ Yes<br>○ No<br>○ e no ansa
301h. You don hear of condoms?<br>PROBE: Men can put a rubber on their penis before dem sleep with woman.<br>[male_condom_150x300.png]	009a = 1<br>○ Yes<br>○ No<br>○ e no ansa
301i. You don hear of female condoms?	009a = 1

PROBE: Women dey put a rubber inside body before she sleep with man.<br>[female_condom_150x300.png]	○ Yes<br>○ No<br>○ e no ansa
301j. You don hear of diaphragm?<br>PROBE: E be like rubber wey dey like plate wey women dey put inside body before she sleep wit man.<br>[diaphragm_150x300.png]	009a = 1<br>○ Yes<br>○ No<br>○ e no ansa
301k. You don hear of foam or jelly method?<br>PROBE: Women fit rub cream or foam inside body before she sleep wit man make she no carry belle.<br>[spermicide_150x300.png]	009a = 1<br>○ Yes<br>○ No<br>○ e no ansa
301l. You don hear about standard days method or Cycle Beads?<br>PROBE: Woman fit use beads wey get color to no the days wey she fit get belle for the day wey she no fit get belle, she wit her man fit use condom or dem no go sleep.<br>[SDM-beads_only.png]	009a = 1<br>○ Yes<br>○ No<br>○ e no ansa
301m. You don hear of Lactational Amenorrhea Method or LAM?	009a = 1<br>○ Yes<br>○ No<br>○ e no ansa
301n. You don hear of rhythm method?<br>PROBE: Women fit no meet man for days of the month wey she no say if she sleep wit man she go carry belle.	009a = 1<br>○ Yes<br>○ No<br>○ e no ansa
301o. You don hear of withdrawal method?<br>PROBE: Man go commot hin penis from woman wen hin wan release.	009a = 1<br>○ Yes<br>○ No<br>○ e no ansa
301p. You don hear any other thing wey woman or man dey do so that hin no go get belle	009a = 1<br>○ Yes<br>○ No<br>○ e no ansa
302a. You and your Partner/Man dey do anything so that she no go carry belle.	210a ≠ 1 AND 009a = 1 210210a ≠ 1 AND 009a = 1 210a ≠ 1 AND 009a = 1 ≠ 1 AND 009a = 1 210a ≠ 1 AND 009a = 1<br>○ Yes<br>○ No<br>○ e no ansa

	${current_user} = 'yes'
302b. Which method wey you dey use?<br>PROBE: You dey use another thing?<br>*Mark all the ones wey she talk. Make sure say u waka go down to see all the different choice.*	302a = 1<br>☐ Female sterilization<br>☐ Male sterilization<br>☐ Implant<br>☐ IUD<br>☐ Injectables<br>☐ Pill<br>☐ Emergency Contraception<br>☐ Male condom<br>☐ Female condom<br>☐ Diaphragm<br>☐ Foam/Jelly<br>☐ Standard Days/Cycle beads<br>☐ LAM<br>☐ Rhythm method<br>☐ Withdrawal<br>☐ Other traditional method<br>☐ e no ansa

CALC_CM. CALCULATE: CURRENT METHOD<br>THIS WILL NOT APPEAR ON THE SCREEN<br>*ODK will identify the most effective method currently being used by the respondent by selecting the highest method in the choice list*	302a=1 AND 302b ≠-99<br>○ Female sterilization<br>○ Male sterilization<br>○ Implant

	◯ IUD<br>◯ Injectables<br>◯ Pill<br>◯ Emergency Contraception<br>◯ Male condom<br>◯ Female condom<br>◯ Diaphragm<br>◯ Foam/Jelly<br>◯ Standard Days/Cycle beads<br>◯ LAM<br>◯ Rhythm method<br>◯ Withdrawal<br>◯ Other traditional method<br>◯ e no ansa
LCL_301. PROBE: Dem bin use big syringe or small one take give you the injection?<br>*Show the image to the respondent.*<br>[sayana_depo_150x300.jpg]	CALC CM = 5<br>◯ Syringe<br>◯ Small needle (Sayana Press)<br>◯ e no ansa
302c. Your husband/partner know say you dey use [CURRENT METHOD]?	302a = 1<br>◯ Yes<br>◯ No<br>◯ e no ansa
302c. Your husband/partner know say you dey use anything to stop belle?	302a = -99<br>◯ Yes<br>◯ No<br>◯ e no ansa
303. This person wey give you this method tell you say e bin permanent?	302b = male or female sterilization<br>◯ Yes<br>◯ No<br>◯ e no ansa
305a. You talk say you no dey use anything to stop belle now. You think say you go use something wen time come in future?	302a ≠1 AND 210a ≠1<br>◯ Yes<br>◯ No<br>◯ e no ansa
305b. You think say you go fit use contraceptive method wey dey stop woman make she no get belle for time to come?	302a ≠1 AND 201a = 1<br>◯ Yes<br>◯ No<br>◯ e no ansa
306a. This 12 months wey pass, you don do something or any method wey fit make you no get belle.	302a ≠1<br>◯ Yes<br>◯ No<br>◯ e no ansa

	${recent_user} = 'yes'
306b. Which method you done use?<br>PROBE: You do any other tin?<br>*Choose the one wey good pass (the one wey high for the list).*	306a = 1<br>◯ Implant<br>◯ IUD<br>◯ Injectables<br>◯ Pill<br>◯ Emergency Contraception<br>◯ Male condom<br>◯ Female condom<br>◯ Diaphragm<br>◯ Foam/Jelly<br>◯ Standard Days/Cycle beads<br>◯ LAM<br>◯ Rhythm method<br>◯ Withdrawal<br>◯ Other traditional method<br>◯ e no ansa

LCL_302. PROBE: The injection na syringe or na small needle dem	306b = 5

take give you?<br>*Show the image to the respondent.*<br>[sayana_depo_150x300.jpg]	◌ Syringe<br>◌ Small needle (Sayana Press)<br>◌ e no ansa
307. Before you start to use [CURRENT METHOD / MOST RECENT METHOD], you been don discuss the plan to delay or avoid pregnancy with your husband/partner?	302a = 1 OR 306a = 1<br>◌ Yes<br>◌ No<br>◌ Do not know<br>◌ e no ansa
308. This method wey you dey use now, na you and your husband/partner agree or na only you decide?	302a = 1<br>◌ Mainly respondent<br>◌ na him husband mainly/ him partner mainly<br>◌ Joint decision<br>◌ Other<br>◌ e no ansa
TCI_302. Apart from you and your husband/partner, another person follow talk about family planning method?<br>PROBE: anybody again?<br>*Do not read options aloud. Select all that apply.*	302a = 1 OR 306a = 1<br>☐ Mother<br>☐ Mother in law<br>☐ Sister(s)<br>☐ Sister(s) in law<br>☐ Grandmother<br>☐ Friend(s)<br>☐ Health worker<br>☐ Community leader<br>☐ Religious leader<br>☐ Aunt<br>☐ Other relatives<br>☐ Other<br>☐ no pesin<br>☐ e no ansa
TCI_302x. 12 months wey pass, you don tell your friend or family person about family planning ?	302a = 1 OR 306a = 1<br>◌ Yes<br>◌ No<br>◌ Do not know<br>◌ e no ansa
308a. The last time wey you collect your [CURREN METHOD /MOST RECENT METHOD], how much you get to pay from your pocket, plus any fees wey you pay for the method, supplies or services, and transportation?<br>*Enter all prices in Naira. Zero is a possible answer. Enter -88 if respondent does not know, -99 for no response.*	302a = 1 OR 306a = 1<br>--

	${current_user} = 'yes'
309a. Since which month and year been don dey use [CURRENT METHOD / MOST RECENT METHOD] without stopping?<br>*you go try remember things wey don shele before make you for soji the mind of the person you dey follow yearn*	302a = 1
Most Recent Birth: [mm-yyyy]	${recent_birth} != ''
Current Marriage: [mm-yyyy]	${husband_cohabit_start_recent} != ''
Month:	◌ January<br>◌ February<br>◌ March<br>◌ April<br>◌ May<br>◌ June<br>◌ July<br>◌ August<br>◌ September<br>◌ October<br>◌ November<br>◌ December<br>◌ Do not know

Year:	Year:

	${recent_user} = 'yes'
309b. When you stop to use [CURRENT METHOD / MOST RECENT METHOD]?<br>*Abeg record the date. The date should be found by calculating backwards from memorable events if needed.*<br>*Select 'Do not know' for month and '2020' for year to indicate No Response.*	306a = 1
Month:	◯ January<br>◯ February<br>◯ March<br>◯ April<br>◯ May<br>◯ June<br>◯ July<br>◯ August<br>◯ September<br>◯ October<br>◯ November<br>◯ December<br>◯ Do not know
Year:	Year: ____________________

	${recent_user} = 'yes'
309c. In which month and year you start to use [CURRENT METHOD / MOST RECENT METHOD] before stopping?<br>*Calculate backwards from memorable events if needed.*<br>*Select 'Do not know' for month and '2020' for year to indicate No Response.*	306a = 1
Most Recent Birth: [mm-yyyy]	${recent_birth} != ''
Current Marriage: [mm-yyyy]	${husband_cohabit_start_recent} != ''
Month:	◯ January<br>◯ February<br>◯ March<br>◯ April<br>◯ May<br>◯ June<br>◯ July<br>◯ August<br>◯ September<br>◯ October<br>◯ November<br>◯ December<br>◯ Do not know
Year:	Year: ____________________

309d. CHECK: Just to make sure say I get am correct, you been use [CURRENT METHOD / MOST RECENT METHOD] continuously between [START DATE] and [END DATE] without stopping, shey e correct so?	306a = 1<br>◯ Yes<br>◯ No
GO BACK TO THE PREVIOUS SCREEN AND PROBE TO DETERMINE THE PERIOD OF MOST RECENT CONTINUOUS USE.<br>*Suggested probes: - When was the last time you used [METHOD]? - How long had you been using [METHOD] without stopping*	309d = 0
310. Why you stop to use [CURRENT METHOD / MOST RECENT METHOD?	306a = 1<br>☐ Infrequent sex / husband away<br>☐ Get belle as e dey use<br>☐ E wan get belle<br>☐ Husband / partner disapproved<br>☐ In want method wey ogbonge pass the one e dey use.

	☐ No method available<br>☐ Health concerns<br>☐ Fear of side effects<br>☐ Lack of access / too far<br>☐ E too cos<br>☐ E no easy to use<br>☐ Fatalistic<br>☐ Difficult to get pregnant / menopausal<br>☐ Interferes with body's processe<br>☐ Other<br>☐ Don't know<br>☐ e no ansa
	${current_or_recent_user} and (${current_recent_method} != 'LAM') and (${current_recent_method} != …
311a. You been first start to use [CURRENT METHOD / MOST RECENT METHOD] on [DATE FROM FQ309a OR 309c] Where you or your partner been get am at that time?<br>*Waka go down to see everything.*	(CALC_CM ≠ 14, 30, 31, 39, -99) OR (306b ≠ 14, 30, 31, 39, -99)<br>○ Government Hospital<br>○ Government Health Center<br>○ Family planning clinic<br>○ Mobile clinic (public)<br>○ TBA/Fieldworker (public)<br>○ Private hospital/clinic<br>○ Pharmacy<br>○ Chemist/PMS Store<br>○ Private doctor or nurse<br>○ Mobile clinic (private)<br>○ TBA/Fieldworker (private)<br>○ Shop<br>○ FBO/Church<br>○ Friend / relative<br>○ NGO<br>○ Market / hawking<br>○ Other<br>○ Do not know<br>○ e no ansa
312a. When you been collect your [CURRENT METHOD / MOST RECENT METHOD], the provider been tell you about side effects or problem wey you fit get with the method to delay or avoid getting belle?	311a ≠ .<br>○ Yes<br>○ No<br>○ e no ansa
312b. Dem bin tell you wetin you fit do if you get problem?	312a = 1<br>○ Yes<br>○ No<br>○ e no ansa
313. At that time, the family planning provider been tell you about methods of family planning rather than the [CURRENT METHOD / MOST RECENT METHOD] wey you fit use?	311a ≠ . OR 311b ≠ .<br>○ Yes<br>○ No<br>○ Do not know<br>○ e no ansa
314a. Wen you go there you bin get the one wey you wan to take stop belle?	311a ≠ .<br>○ Yes<br>○ No<br>○ e no ansa
314c. Wetin make you bin no get the method wey you want?	314a = 0<br>○ Method don finish dat day<br>○ Method no dey at all<br>○ The person wey dey ther no sabi give the method<br>○ The person wey dey there say make I use anoda one<br>○ I no qualify to use the method

	◌ I make up my mind say I no go greee use any method<br>◌ The money too much<br>◌ Other<br>◌ e no ansa
315a. Dat time una bin go there, na who talk last sey make una use dat method?	311a ≠ .<br>◌ Only U<br>◌ The person wey dey the place<br>◌ Your oga<br>◌ U and the person wey dey the place<br>◌ U and your oga<br>◌ Other<br>◌ Do not know<br>◌ e no ansa
315b. Na who talk last for dis method rhythm?	311b ≠ .<br>◌ Only U<br>◌ The person wey dey the place<br>◌ Your oga<br>◌ U and the person wey dey the place<br>◌ U and your oga<br>◌ Other<br>◌ Do not know<br>◌ e no ansa
315b. Na who talk last for dis method LAM?	311b ≠ .<br>◌ Only U<br>◌ The person wey dey the place<br>◌ Your oga<br>◌ U and the person wey dey the place<br>◌ U and your oga<br>◌ Other<br>◌ Do not know<br>◌ e no ansa
316. You get like return to this provider? Provider: [Type of provider selected in 311a or 311b]	311a ≠ 35 or 96<br>◌ Yes<br>◌ No<br>◌ Do not know<br>◌ e no ansa
317. You go like refer your family or friend to this provider / facility? Provider: [Type of provider selected in 311a or 311b]	311 a ≠ 34 or 96<br>◌ Yes<br>◌ No<br>◌ Do not know<br>◌ e no ansa
SW_1a. Right before you start to dey use [CURRENT METHOD / MOST RECENT METHOD] in [MOIS/ANNEE], you dey do something or using any method to delay or avoid getting belle?	302a = 1 OR 306a = 1<br>◌ Yes<br>◌ No<br>◌ e no ansa
SW_1b. Which method you bin dey use?	SW_1a = 1<br>◌ Implant<br>◌ IUD<br>◌ Injectables<br>◌ Pill<br>◌ Emergency Contraception<br>◌ Male condom<br>◌ Female condom<br>◌ Diaphragm<br>◌ Foam/Jelly<br>◌ Standard Days/Cycle beads

	◯ LAM<br>◯ Rhythm method<br>◯ Withdrawal<br>◯ Other traditional method<br>◯ e no ansa
PP_1. Since you born your child in [DATE OF MOST RECENT BIRTH], you don do something or used any method to delay or avoid getting belle?	child born in last 2 years AND 302a ≠ 1<br>◯ Yes<br>◯ No<br>◯ e no ansa
PP_2. How long after the birth in [DATE OF MOST RECENT BIRTH] did you start doing something or start using a method?<br>*Enter 0 days for today. You will enter a number for X on the next screen.*	PP_1 = 1 OR (302a = 1 AND child born in the last 2 years)<br>◯ X days after<br>◯ X weeks after<br>◯ X months after<br>◯ X years after<br>◯ e no ansa
PP_2. Enter [METHOD].<br>*If today, enter zero days only, not zero weeks/months/years.*	${pp_method_units} = 'days' or ${pp_method_units} = 'weeks' or ${pp_method_units} = 'months' or ${ …<br>--
PP_3. Wetin be the method?	PP_2 ≠ .<br>◯ Female sterilization<br>◯ Male sterilization<br>◯ Implant<br>◯ IUD<br>◯ Injectables<br>◯ Pill<br>◯ Emergency Contraception<br>◯ Male condom<br>◯ Female condom<br>◯ Diaphragm<br>◯ Foam/Jelly<br>◯ Standard Days/Cycle beads<br>◯ LAM<br>◯ Rhythm method<br>◯ Withdrawal<br>◯ Other traditional method<br>◯ e no ansa
LCL_PP. PROBE: The injection dem give you na thru syring or thru small needle?<br>*Show the image to the respondent.*<br>[sayana_depo_150x300.jpg]	PP_3 = 5<br>◯ Syringe<br>◯ Small needle (Sayana Press)<br>◯ e no ansa
319. You done ever do anything or try in any way to delay or so that you no go get belle?	306a ≠ 1 OR 302a ≠ 1<br>◯ Yes<br>◯ No<br>◯ e no ansa
320. How old you dey when you first use one method to delay or avoid getting belle? The respondent said she was [AGE] years old at her last birthday.<br>*Write the age for years Write -88 if the woman no know Write -99 if she no answer She no fit small pass 9 years o.*	302a = 1 OR 306a =1 OR 319 = 1<br>--
321. How many children wey dey alive you been get at that time, if any? Note: the respondent said that she gave birth [NUMBER OF LIFE BIRTHS] times in 201.<br>*Write -99 if she no answer*	Age in 320 ≥ 9 AND 200 = 1<br>--

	${fp_ever_used} = 'yes'
322. Na which method wey you bin first use dat time wey you no get belle?<br>*Do not read the method choices. Scroll to bottom to see all choices.*	319 = 1<br>◯ Female sterilization<br>◯ Male sterilization<br>◯ Implant<br>◯ IUD

	Injectables<br>Pill<br>Emergency Contraception<br>Male condom<br>Female condom<br>Diaphragm<br>Foam/Jelly<br>Standard Days/Cycle beads<br>LAM<br>Rhythm method<br>Withdrawal<br>Other traditional method<br>e no ansa

LCL_322a. PROBE: Weda the injection wey dem bin give her na thru syringe or small needle?<br>*Show the image to the respondent.*<br>[sayana_depo_150x300.jpg]	322 = 5<br>Syringe<br>Small needle (Sayana Press)<br>e no ansa
322a. In the last 12 months, You don take any medcine wey make you no carry belle?<br>PROBE:As an emergency measure after unprotected sexual intercourse women can take special pills at any time within three to five days to prevent pregnancy.	302b ≠ emergency contraception OR 306b ≠ 8<br>Yes<br>No<br>e no ansa

	((${current_user} = 'no')) and (((${more_children_none} = 'no_children') or …
323a. You say you no wan pikin now and you no dey take anything or do anything make you no get belle.	302a = 0 AND ((212a or 212b > 2 years) OR (211a or 211b = 2))
323a. You say you no wan get another pikin now and you no dey use anything wey you no go get belle.	302a = 0 AND ((212a or 212b > 2 years) OR (211a or 211b = 2))
323a. You say you no wan get another pikin now and you no dey use anything wey you no go get belle	302a = 0 AND ((212a or 212b > 2 years) OR (211a or 211b = 2))
323a. You say you no wan get another pikin now and you no dey use anything wey you no go get belle.	302a = 0 AND ((212a or 212b > 2 years) OR (211a or 211b = 2))
You fit tell me why you no dey use anything wey go make you no get belle?<br>PROBE: If any other thing?<br>*RECORD ALL REASONS MENTIONED*<br>*Cannot select "Not Married" if 104 is "Yes, currently married".*<br>*Scroll to bottom to see all choices.*	Not married<br>Infrequent sex / Not having sex<br>She don stop kpata kpata to see her period/ or she don comot her womb<br>Subfecund / Infecund<br>Not menstruated since last birth<br>feeding pikin with breastmilk<br>Husband away for multiple days<br>Up to God / fatalistic<br>Respondent opposed<br>Husband / partner opposed<br>Others opposed<br>Religious prohibition<br>Knows no method<br>Knows no source<br>Fear of side effects<br>Health concerns<br>Lack of access / too far<br>E too cos<br>Preferred method not available<br>No method available<br>E no easy to use<br>Interferes with body’s processe<br>Other<br>Don’t know<br>e no ansa

323b. You fit talk weda as you no dey use any protection, if na your decision,your husband/your man decision or una two decide am	302a ≠ 1<br>Mainly respondent

together?	○ na him husband mainly/ him partner mainly<br>○ Joint decision<br>○ Other<br>○ e no ansa
TCI_304. Apart from you and your husband/man, who again influences say make you no use prevention?<br>PROBE: If any other thing?<br>*Do not read options aloud. Select all that apply.*	306a = 0<br>☐ Mother<br>☐ Mother in law<br>☐ Sister(s)<br>☐ Sister(s) in law<br>☐ Grandmother<br>☐ Friend(s)<br>☐ Health worker<br>☐ Community leader<br>☐ Religious leader<br>☐ Aunt<br>☐ Other relatives<br>☐ Other<br>☐ no pesin<br>☐ e no ansa
TCI_304x. For the past 12 months, any of your friends or relatives don tell you to use a Family Planning method?	306a = 0<br>○ Yes<br>○ No<br>○ e no ansa
324. For the past 12 months, any community health worker don waka visit you and con talk to you about Family Planning ?	009a = 1<br>○ Yes<br>○ No<br>○ e no ansa
325a. For the past 12 months, you don visit any health facility or place to care for yourself?<br>*For any health services.*	009a = 1<br>○ Yes<br>○ No<br>○ e no ansa
325a. For the last 12 months, you don visit any health facility or place to take care of yourself or your children?<br>*For any health services.*	009a = 1<br>○ Yes<br>○ No<br>○ e no ansa
325b. E get any staff for the place wia you visit talk to you about family planning methods?	325a = 1<br>○ Yes<br>○ No<br>○ e no ansa
TCI_305. You don go for any community meeting for the past year wey dem talk about good tins about family planning?	009a = 1<br>○ Yes<br>○ No<br>○ e no ansa
TCI_306. You think say e get some people wey dey for your community wey go call you bad names or no go wan talk to you if dem hear say you dey use family planning method?	009a = 1<br>○ Yes<br>○ No<br>○ Do not know<br>○ e no ansa
TCI_307. You think say e get some people wey dey for your community wey go praise,encourage, or talk beta tin about you if dey hear say you bin use family planning method?	009a = 1<br>○ Yes<br>○ No<br>○ Do not know<br>○ e no ansa
TCI_308. For the past 12 months, you don heard any of the following people openly talk beta tins about family planning?<br>*Read all options wey dey your phone and select all that apply.*	009a = 1<br>☐ Government official (national level)<br>☐ State, LGA, or local leaders<br>☐ Religious leader<br>☐ None of the above

	~~e no ansa~~
TCI_309. For the past 12 months, you don hear any of the following people openly talk bad tins about family planning?<br>*Read all options wey dey your phone and select all that apply.*	009a = 1<br>☐ Government official (national level)<br>☐ State, LGA, or local leaders<br>☐ Religious leader<br>☐ None of the above<br>☐ e no ansa
TCI_309x. How many of your friends wey close to you or family people you think say dey use family planning: nobody, some of them, plenty of them, or all of them?	009a = 1<br>○ None<br>○ Some<br>○ Most<br>○ All<br>○ Do not know<br>○ e no ansa

			009a = 1
326. This last months wey just pass so, you done:			
	Yes	No	e no ansa
326a. Hear about family planning on the radio?	○	○	○
326b. See anything about family planning on the television?	○	○	○
326c. Read about family planning inside newspaper or magazine?	○	○	○
326d. Received a voice or text message about family planning inside your phone?	○	○	○
TCI_310. Read about family planning inside any small paper dem (brochure, leaflet, or flyer)?	○	○	○
TCI_311. See for paper wey dey for wall (a poster) for sign board (billboard) with a family planning message?	○	○	○

Section 4 – Sexual Activity

CHECK FOR THE PRESENCE OF OTHERS. BEFORE CONTINUING, MAKE EVERY EFFORT TO ENSURE PRIVACY.

Now I wan ask some questions about your sexual activity so that e fit help us understand some tins wey dey happen for life make I assure you say any ansa wey you give dey safe and I no go tell anybody. And if e reach any question wey you wan ansa just tell me i go jump am pass	${consent_obtained}

	${consent_obtained}
401a. How many years you dey wen you first sleep with man?	309a = 1
Current age: [AGE]	
Number of live births: [NUMBER OF LIFE BIRTHS]	${birth_events} > 0
The respondent is pregnant	${pregnant} = 'yes'
Enter the age in years.<br>*Enter -77 if she has never had sex. Enter -99 for no response. Enter -88 for do not know.*	--

	((${age_at_first_sex} >= 0) or (${age_at_first_sex} =-88) or (${age_at_first_sex} =-99))
402. Wen be the last time wey you sleep with man?	401a ≠ -77

402. Enter [# days / weeks / months / years].<br>*If today, enter zero days only, not zero weeks/months/years.*<br>*This suppose gree with the age of first sexual intercourse and the pregnancy status.*	401a ≠ -77<br>--
LCL_403. if you sleep with man only one time without protection, you think say you go carry belle?<br>*Read response options aloud.*	${consent_obtained}<br>○ Very likely<br>○ Somewhat likely<br>○ Equally likely and unlikely<br>○ Not likely<br>○ Don’t know

LCL_404. If you sleep with man two times a week regularly for one year without protection you think say you go get belle?<br>*Read response options aloud.*	○ e no ansa ${consent_obtained}<br>○ Very likely<br>○ Somewhat likely<br>○ Equally likely and unlikely<br>○ Not likely<br>○ Don't know<br>○ e no ansa

Section 6 – Menstrual Hygiene

Now I go how you dey manage your menstrual hygiene. This includes the use of absorbent materials; access to a private, clean, safe space; washing as required; and a place to dispose used materials.

602a. Na for wia you dey change your pads, cloths, or other sanitary materials?	(209 ≤ 90 days, 13 weeks or ≤ 3 months)<br>○ Flush/pour flush toilet<br>○ Ventilated improved pit latrine<br>○ Pit latrine with slab<br>○ Pit latrine without slab / open pit<br>○ Bucket toilet<br>○ Composting toilet<br>○ Hanging toilet /Hanging latrine<br>○ Sleeping area/bedroom<br>○ Backyard<br>○ No facility / bush / field<br>○ Other<br>○ e no ansa
Place: [MAIN PLACE FROM 602a] 602b. While managing your menstrual hygiene, was this place:<br>*Read each option aloud and select if yes.*	601 ≠ -99 nor null AND 602 ≠ -99 nor null<br>☐ Clean?<br>☐ Private?<br>☐ Safe?<br>☐ you fit lock am?<br>☐ water follow am come?<br>☐ soap follow am come?<br>☐ None of the above<br>☐ e no ansa
603. Wetin you use collect or absorb your blood for your period the last time e come?<br>PROBE: E get anything again?<br>*Do not read options aloud. Select all that apply.*	(209 ≤ 90 days, 13 weeks or ≤ 3 months)<br>☐ Disposable sanitary pad (commercial)<br>☐ Reusable sanitary pad<br>☐ New cloth<br>☐ Old cloth<br>☐ Cotton wool<br>☐ Diaper<br>☐ Tampons<br>☐ Toilet paper<br>☐ Underwear alone<br>☐ Bucket<br>☐ Other<br>☐ No materials used<br>☐ e no ansa
604a. You bin wash and reuse pads, cloths, or other sanitary materials wen your last period bin come?	603 = reusable sanitary pad, old cloth, underwear alone, or bucket<br>○ Yes<br>○ No<br>○ e no ansa
604b. That time wey your period bin com, sanitary material wey you bin wash dry before you use dem again?	604a = 1<br>○ Yes<br>○ No<br>○ e no ansa
605. You mentioned say you used [ODK will display the responses	603 = disposable sanitary pad, new cloth,

from 603] during your last menstrual period. Where you dispose the material when you use am finish? PROBE: Anywhere else?<br>*Do not read options aloud. Select all that apply.*	cotton wool, diaper, tampons, toilet paper, or other OR 604a =0<br>☐ Flush toilet<br>☐ Latrine<br>☐ Waste bin/trash bag<br>☐ Burning<br>☐ Bush/field<br>☐ Other<br>☐ e no ansa
606a. Apart from your own housework, e get another work wey you do for last month?	(209 ≤ 90 days, 13 weeks or ≤ 3 months)<br>○ Yes<br>○ No<br>○ e no ansa
606b. E get anyday wey your last period no allow you go work?	606a = 1<br>○ Yes<br>○ No<br>○ e no ansa
607a. You don go school at all for the 12 months wey don pass?	(209 ≤ 90 days, 13 weeks or ≤ 3 months)<br>○ Yes<br>○ No<br>○ e no ansa
607b. E get anyday wey you no go school for the past 12 months because you dey menstruate?	607a = 1<br>○ Yes<br>○ No<br>○ e no ansa

Section 7.1 – Confidantes

701. I wan ask you questions about your best female friends or relatives. Dis na women wey you fit tell your personal infomation and dem too dey tell you their own. How many of this kain people you get wey dey for Nigeria and dia age na between 15 and 49 years<br>*Enter -88 for do not know, -99 for no response.*	${consent_obtained}<br>--
702a. Abeg, imagine that your tight female friend or relative we dey live for this Nigeria. Remember say shego dey between 15 to 49 wey you dey tell very personal information and wey she dey tell you her very personal information too. to make am easy for you, you fit give me any fake name for am?	${friend_count} > 0<br>--
703a. How many years ${friend1_name} dey last year?<br>*Enter -88 for do not know, -99 for no response.*	${friend_count} > 0<br>--
704a. Were ${friend1_name} school reach ?	${friend_count} > 0<br>○ I no go at all<br>○ Primary<br>○ Secondary<br>○ Higher<br>○ e no ansa
702b. Abeg, imagine your seond tight female friend or relative we dey also live for this Nigeria wey dey between 15 to 49 wey you dey tell very personal information and wey she sef dey tell you her very personal information too. to make am easy for you, you fit give me any fake name for am?	${friend_count} > 1<br>--
703b. How many years ${friend2_name} dey last year?<br>*Enter -88 for do not know, -99 for no response.*	${friend_count} > 1<br>--
704b. were ${friend2_name} school reach?	${friend_count} > 1<br>○ I no go at all<br>○ Primary<br>○ Secondary<br>○ Higher<br>○ e no ansa

Section 7.2 – Abortion

CHECK FOR THE PRESENCE OF OTHERS. BEFORE CONTINUING, MAKE EVERY EFFORT TO ENSURE PRIVACY.

Question	Response
The next questions dem i wan ask na how women for your community dey carry comot belle. This one na common thing for naija, so we wan sabi how women dey take comot belle. Make I remind you say the mata wey we dey discuss here na private issue and nobody go hear any answer wey you give me now. So, if we reach any question wey you no like and you no wan ansa just tell me and i go jump am pass.<br>*Press OK to continue.*	${consent_obtained}<br>☐ na so
705. Sometimes some women dey fear say them don get belle wey them no won get belle, so dem go do something to comot the belle. This kin thing dey happen for your community wey you dey live well well?<br>*Read response options aloud.*	${consent_obtained}<br>○ Very common<br>○ Somewhat common<br>○ Not very common<br>○ Not at all common<br>○ Do not know<br>○ e no ansa
706. For the community wey you dey live now wetin woman way get belle or fear say she get belle go do to take comot am?<br>[**PROBE**: Anoda one dey ?]<br>*Do not read options aloud. Select all that apply.*	${consent_obtained}<br>☐ Surgical procedure<br>☐ Pills called mifepristone or misoprostol, for example Mariprist, Mifepak, Cytotec, Miso-Fem, or Misoclear<br>☐ Pills you take when you have a fever like antibiotics or anti-malarial medicine, for example quinine<br>☐ Emergency contraception pills, for example Postinor<br>☐ Other pills<br>☐ injection<br>☐ Traditional methods, like herbs<br>☐ Alcohol<br>☐ Salt, potash, maggi, or kanwa<br>☐ Lemon or lime<br>☐ Cough syrup<br>☐ Insert materials into the vagina<br>☐ Other<br>☐ Do not know<br>☐ e no ansa
707. Which one them dey use pass?	count-selected(${abt_ways}) > 1<br>○ Surgical procedure<br>○ Pills called mifepristone or misoprostol, for example Mariprist, Mifepak, Cytotec, Miso-Fem, or Misoclear<br>○ Pills you take when you have a fever like antibiotics or anti-malarial medicine, for example quinine<br>○ Emergency contraception pills, for example Postinor<br>○ Other pills<br>○ injection<br>○ Traditional methods, like herbs<br>○ Alcohol<br>○ Salt, potash, maggi, or kanwa<br>○ Lemon or lime<br>○ Cough syrup<br>○ Insert materials into the vagina<br>○ Other<br>○ Do not know

	○ e no ansa<br>~~selected(${abt_ways}, filter) or (filter = 'always')~~
708. From where them dey do this operation to commot the belle? Another one dey?<br>[**PROBE**: Anoda one dey ?]<br>*Do not read options aloud. Select all that apply.*	(selected(${abt_ways},'surgery'))<br>☐ Government Hospital<br>☐ Government Health Center<br>☐ Family planning clinic<br>☐ Mobile clinic (public)<br>☐ TBA/Fieldworker (public)<br>☐ Private hospital/clinic<br>☐ Pharmacy<br>☐ Chemist/PMS Store<br>☐ Private doctor or nurse<br>☐ Mobile clinic (private)<br>☐ TBA/Fieldworker (private)<br>☐ Shop<br>☐ FBO/Church<br>☐ Friend / relative<br>☐ NGO<br>☐ Market / hawking<br>☐ Other<br>☐ Do not know<br>☐ e no ansa
709. Which one them dey go well well?	count-selected(${abt_surg_where}) > 1<br>○ Government Hospital<br>○ Government Health Center<br>○ Family planning clinic<br>○ Mobile clinic (public)<br>○ TBA/Fieldworker (public)<br>○ Private hospital/clinic<br>○ Pharmacy<br>○ Chemist/PMS Store<br>○ Private doctor or nurse<br>○ Mobile clinic (private)<br>○ TBA/Fieldworker (private)<br>○ Shop<br>○ FBO/Church<br>○ Friend / relative<br>○ NGO<br>○ Market / hawking<br>○ Other<br>○ Do not know<br>○ e no ansa<br>selected(${abt_surg_where}, filter) or (filter = 'always')
710. Na for where them dey buy the medicine to take comot belle? Another one dey?	(selected(${abt_ways}, 'pills_abortion')) or (selected(${abt_ways}, 'pills_fever')) or (selected($ …<br>☐ Government Hospital<br>☐ Government Health Center<br>☐ Family planning clinic<br>☐ Mobile clinic (public)<br>☐ TBA/Fieldworker (public)<br>☐ Private hospital/clinic<br>☐ Pharmacy<br>☐ Chemist/PMS Store<br>☐ Private doctor or nurse<br>☐ Mobile clinic (private)<br>☐ TBA/Fieldworker (private)<br>☐ Shop<br>☐ FBO/Church<br>☐ Friend / relative<br>☐ NGO<br>☐ Market / hawking

	☐ Other<br>☐ Do not know<br>☐ e no ansa
711. Which one dey common?	count-selected(${abt_meds_where}) > 1<br>○ Government Hospital<br>○ Government Health Center<br>○ Family planning clinic<br>○ Mobile clinic (public)<br>○ TBA/Fieldworker (public)<br>○ Private hospital/clinic<br>○ Pharmacy<br>○ Chemist/PMS Store<br>○ Private doctor or nurse<br>○ Mobile clinic (private)<br>○ TBA/Fieldworker (private)<br>○ Shop<br>○ FBO/Church<br>○ Friend / relative<br>○ NGO<br>○ Market / hawking<br>○ Other<br>○ Do not know<br>○ e no ansa<br>selected(${abt_meds_where}, filter) or (filter = 'always')
712a.i. Now, I wan ask about that your friend ${friend1_name}. She don do anything to take comot belle when she carry belle or when she dey fear say she get belle?<br>*Probe to confirm whether the pregnancy removal was successful.*	○ Yes, I dey sure<br>○ Yes, E be like<br>○ No<br>○ Do not know<br>○ e no ansa
713a.i. Na which year e take happen?<br>*If indicates happened more than once, specify most recent time.*<br>*Enter 2020 for 'Do not know' or 'No response'.*	(${friend1_abt_yn} = 'yes') or (${friend1_abt_yn} = 'likely')<br>Year: ________________
714a.i. Sometimes, women dey do plenty plenty tins to take commot belle. N wetin ${friend1_name} do to take commot the belle?	(${friend1_abt_yn} = 'yes') or (${friend1_abt_yn} = 'likely')<br>○ Yes, I dey sure<br>○ Yes, E be like<br>○ No<br>○ Do not know<br>○ e no ansa
715a.i. Na wetin be the first thing she do to take comot the belle?	(${friend1_abt_mult_yn} = 'yes') or (${friend1_abt_mult_yn} = 'likely')<br>○ Surgical procedure<br>○ Pills called mifepristone or misoprostol, for example Mariprist, Mifepak, Cytotec, Miso-Fem, or Misoclear<br>○ Pills you take when you have a fever like antibiotics or anti-malarial medicine, for example quinine<br>○ Emergency contraception pills, for example Postinor<br>○ Other pills<br>○ injection<br>○ Traditional methods, like herbs<br>○ Alcohol<br>○ Salt, potash, maggi, or kanwa<br>○ Lemon or lime<br>○ Cough syrup<br>○ Insert materials into the vagina<br>○ Other

	◌ Do not know<br>◌ e no ansa
715a.i. Wetin she do wey con commot the belle?	(${friend1_abt_mult_yn} = 'no') or (${friend1_abt_mult_yn} = '-88')<br>◌ Surgical procedure<br>◌ Pills called mifepristone or misoprostol, for example Mariprist, Mifepak, Cytotec, Miso-Fem, or Misoclear<br>◌ Pills you take when you have a fever like antibiotics or anti-malarial medicine, for example quinine<br>◌ Emergency contraception pills, for example Postinor<br>◌ Other pills<br>◌ injection<br>◌ Traditional methods, like herbs<br>◌ Alcohol<br>◌ Salt, potash, maggi, or kanwa<br>◌ Lemon or lime<br>◌ Cough syrup<br>◌ Insert materials into the vagina<br>◌ Other<br>◌ Do not know<br>◌ e no ansa
716a.i. Were she go do the procedure?	(${friend1_abt_first} = 'surgery') or (${friend1_abt_only} = 'surgery')<br>◌ Government Hospital<br>◌ Government Health Center<br>◌ Family planning clinic<br>◌ Mobile clinic (public)<br>◌ TBA/Fieldworker (public)<br>◌ Private hospital/clinic<br>◌ Pharmacy<br>◌ Chemist/PMS Store<br>◌ Private doctor or nurse<br>◌ Mobile clinic (private)<br>◌ TBA/Fieldworker (private)<br>◌ Shop<br>◌ FBO/Church<br>◌ Friend / relative<br>◌ NGO<br>◌ Market / hawking<br>◌ Other<br>◌ Do not know<br>◌ e no ansa
717a.i. Were she for get the medicine to comot the belle?	(${friend1_abt_first} = 'pills_abortion') or (${friend1_abt_only} = 'pills_abortion') or (${friend …<br>◌ Government Hospital<br>◌ Government Health Center<br>◌ Family planning clinic<br>◌ Mobile clinic (public)<br>◌ TBA/Fieldworker (public)<br>◌ Private hospital/clinic<br>◌ Pharmacy<br>◌ Chemist/PMS Store<br>◌ Private doctor or nurse<br>◌ Mobile clinic (private)<br>◌ TBA/Fieldworker (private)<br>◌ Shop<br>◌ FBO/Church

	◌ Friend / relative<br>◌ NGO<br>◌ Market / hawking<br>◌ Other<br>◌ Do not know<br>◌ e no ansa
718a.i. Wetin be the last thing she do way con commot the belle?	(${friend1_abt_mult_yn} = 'yes') or (${friend1_abt_mult_yn} = 'likely')<br>◌ Surgical procedure<br>◌ Pills called mifepristone or misoprostol, for example Mariprist, Mifepak, Cytotec, Miso-Fem, or Misoclear<br>◌ Pills you take when you have a fever like antibiotics or anti-malarial medicine, for example quinine<br>◌ Emergency contraception pills, for example Postinor<br>◌ Other pills<br>◌ injection<br>◌ Traditional methods, like herbs<br>◌ Alcohol<br>◌ Salt, potash, maggi, or kanwa<br>◌ Lemon or lime<br>◌ Cough syrup<br>◌ Insert materials into the vagina<br>◌ Other<br>◌ Do not know<br>◌ e no ansa
719a.i. Na where she con go do the procedure?	${friend1_abt_last} = 'surgery'<br>◌ Government Hospital<br>◌ Government Health Center<br>◌ Family planning clinic<br>◌ Mobile clinic (public)<br>◌ TBA/Fieldworker (public)<br>◌ Private hospital/clinic<br>◌ Pharmacy<br>◌ Chemist/PMS Store<br>◌ Private doctor or nurse<br>◌ Mobile clinic (private)<br>◌ TBA/Fieldworker (private)<br>◌ Shop<br>◌ FBO/Church<br>◌ Friend / relative<br>◌ NGO<br>◌ Market / hawking<br>◌ Other<br>◌ Do not know<br>◌ e no ansa
720a.i. Na where she get the medicine to comot the belle?	(${friend1_abt_last} = 'pills_abortion') or (${friend1_abt_last} = 'pills_fever') or (${friend1_ab …<br>◌ Government Hospital<br>◌ Government Health Center<br>◌ Family planning clinic<br>◌ Mobile clinic (public)<br>◌ TBA/Fieldworker (public)<br>◌ Private hospital/clinic<br>◌ Pharmacy<br>◌ Chemist/PMS Store<br>◌ Private doctor or nurse<br>◌ Mobile clinic (private)

	◌ TBA/Fieldworker (private)<br>◌ Shop<br>◌ FBO/Church<br>◌ Friend / relative<br>◌ NGO<br>◌ Market / hawking<br>◌ Other<br>◌ Do not know<br>◌ e no ansa
721a.i. Shey ${friend1_name} bin get any wahala wey make am go hospital becos she dey try to comot the belle?<br>*If the respondent already reported the friend went to a health facility in the process of removing the pregnancy, we are interested in whether the friend went back to a health facility on a separate occasion to treat complications that she may have experienced.*	(${friend1_abt_yn} = 'yes') or (${friend1_abt_yn} = 'likely')<br>◌ Yes, I dey sure<br>◌ Yes, E be like<br>◌ No<br>◌ Do not know<br>◌ e no ansa
712a.ii. Apart from all dis one wey we don talk about. ${friend1_name} don do anything before to regulate her period when she dey fear say she get belle?<br>*Probe to confirm whether the period regulation was successful. If not, select 'no.'*	${friend1_abt_yn} = 'yes'<br>◌ Yes, I dey sure<br>◌ Yes, E be like<br>◌ No<br>◌ Do not know<br>◌ e no ansa
712a.ii. That your best friend ${friend1_name} do anything to bring out her period when she dey fear say she get belle?<br>*Probe to confirm whether the period regulation was successful. If not, select 'no.'*	${friend1_abt_yn} != 'yes'<br>◌ Yes, I dey sure<br>◌ Yes, E be like<br>◌ No<br>◌ Do not know<br>◌ e no ansa
713a.ii. Which year wey this tin happen last?<br>*If indicates happened more than once, specify most recent time.*<br>*Enter 2020 for 'Do not know' or 'No response'.*	(${friend1_reg_yn} = 'yes') or (${friend1_reg_yn} = 'likely')<br>Year: ____________________
714a.ii. Women dey do things to make their period come out wen den feel say belle don come. That your best friend ${friend1_name} do pass one thing to make am come out?	((${friend1_reg_year} > ${friend1_abt_year}) or (${friend1_abt_year} = '')) and ((${friend1_reg_yn …<br>◌ Yes, I dey sure<br>◌ Yes, E be like<br>◌ No<br>◌ Do not know<br>◌ e no ansa
715a.ii. Wetin she first do to make the period come out?	((${friend1_reg_year} > ${friend1_abt_year}) or (${friend1_abt_year} = '')) and ((${friend1_reg_mu …<br>◌ Surgical procedure<br>◌ Pills called mifepristone or misoprostol, for example Mariprist, Mifepak, Cytotec, Miso-Fem, or Misoclear<br>◌ Pills you take when you have a fever like antibiotics or anti-malarial medicine, for example quinine<br>◌ Emergency contraception pills, for example Postinor<br>◌ Other pills<br>◌ injection<br>◌ Traditional methods, like herbs<br>◌ Alcohol<br>◌ Salt, potash, maggi, or kanwa<br>◌ Lemon or lime<br>◌ Cough syrup<br>◌ Insert materials into the vagina<br>◌ Other<br>◌ Do not know

e no ansa

715a.ii. Wetin she do to bring her period back?

((${friend1_reg_year} > ${friend1_abt_year}) or (${friend1_abt_year} = '')) and ((${friend1_reg_mu …

- Surgical procedure
- Pills called mifepristone or misoprostol, for example Mariprist, Mifepak, Cytotec, Miso-Fem, or Misoclear
- Pills you take when you have a fever like antibiotics or anti-malarial medicine, for example quinine
- Emergency contraception pills, for example Postinor
- Other pills
- injection
- Traditional methods, like herbs
- Alcohol
- Salt, potash, maggi, or kanwa
- Lemon or lime
- Cough syrup
- Insert materials into the vagina
- Other
- Do not know
- e no ansa

716a.ii. Which place wey she go for the procedure?

((${friend1_reg_year} > ${friend1_abt_year}) or (${friend1_abt_year} = '')) and ((${friend1_reg_fi …

- Government Hospital
- Government Health Center
- Family planning clinic
- Mobile clinic (public)
- TBA/Fieldworker (public)
- Private hospital/clinic
- Pharmacy
- Chemist/PMS Store
- Private doctor or nurse
- Mobile clinic (private)
- TBA/Fieldworker (private)
- Shop
- FBO/Church
- Friend / relative
- NGO
- Market / hawking
- Other
- Do not know
- e no ansa

717a.ii. Where she get the medicine ?

((${friend1_reg_year} > ${friend1_abt_year}) or (${friend1_abt_year} = '')) and ((${friend1_reg_fi …

- Government Hospital
- Government Health Center
- Family planning clinic
- Mobile clinic (public)
- TBA/Fieldworker (public)
- Private hospital/clinic
- Pharmacy
- Chemist/PMS Store
- Private doctor or nurse
- Mobile clinic (private)
- TBA/Fieldworker (private)
- Shop
- FBO/Church

- Friend / relative
- NGO
- Market / hawking
- Other
- Do not know
- e no ansa

718a.ii. Wetin be the last thing she do wey make her period com out?

((${friend1_reg_year} > ${friend1_abt_year}) or (${friend1_abt_year} = '')) and ((${friend1_reg_mu …

- Surgical procedure
- Pills called mifepristone or misoprostol, for example Mariprist, Mifepak, Cytotec, Miso-Fem, or Misoclear
- Pills you take when you have a fever like antibiotics or anti-malarial medicine, for example quinine
- Emergency contraception pills, for example Postinor
- Other pills
- injection
- Traditional methods, like herbs
- Alcohol
- Salt, potash, maggi, or kanwa
- Lemon or lime
- Cough syrup
- Insert materials into the vagina
- Other
- Do not know
- e no ansa

719a.ii. Were she for go for the procedure?

((${friend1_reg_year} > ${friend1_abt_year}) or (${friend1_abt_year} = '')) and (${friend1_reg_las …

- Government Hospital
- Government Health Center
- Family planning clinic
- Mobile clinic (public)
- TBA/Fieldworker (public)
- Private hospital/clinic
- Pharmacy
- Chemist/PMS Store
- Private doctor or nurse
- Mobile clinic (private)
- TBA/Fieldworker (private)
- Shop
- FBO/Church
- Friend / relative
- NGO
- Market / hawking
- Other
- Do not know
- e no ansa

720a.ii. Were she go buy the medicine?

((${friend1_reg_year} > ${friend1_abt_year}) or (${friend1_abt_year} = '')) and ((${friend1_reg_la …

- Government Hospital
- Government Health Center
- Family planning clinic
- Mobile clinic (public)
- TBA/Fieldworker (public)
- Private hospital/clinic
- Pharmacy

	○ Chemist/PMS Store<br>○ Private doctor or nurse<br>○ Mobile clinic (private)<br>○ TBA/Fieldworker (private)<br>○ Shop<br>○ FBO/Church<br>○ Friend / relative<br>○ NGO<br>○ Market / hawking<br>○ Other<br>○ Do not know<br>○ e no ansa
721a.ii. That your best ${friend1_name} get any wahala wey make am go hospital for treatment becos she dey for see period?<br>*If the respondent already reported the friend went to a health facility in the process of regulating her period, we are interested in whether the friend went back to a health facility on a separate occasion to treat complications that she may have experienced.*	((${friend1_reg_year} > ${friend1_abt_year}) or (${friend1_abt_year} = '')) and ((${friend1_reg_yn …<br>○ Yes, I dey sure<br>○ Yes, E be like<br>○ No<br>○ Do not know<br>○ e no ansa
712b.i. I wan ask you questions about your ${friend2_name}. She don ever do anything to remove belle when she carry belle or when she dey fear say she get belle?<br>*Probe to confirm whether the pregnancy removal was successful.*	○ Yes, I dey sure<br>○ Yes, E be like<br>○ No<br>○ Do not know<br>○ e no ansa
713b.i. Which year this tin happen last?<br>*If indicates happened more than once, specify most recent time.*<br>*Enter 2020 for 'Do not know' or 'No response'.*	(${friend2_abt_yn} = 'yes') or (${friend2_abt_yn} = 'likely')<br>Year: ________________
714b.i. Sometimes, women dey do some kind things to make belle stop to dey grow. ${friend2_name} do pass one thing to commot her belle?	(${friend2_abt_yn} = 'yes') or (${friend2_abt_yn} = 'likely')<br>○ Yes, I dey sure<br>○ Yes, E be like<br>○ No<br>○ Do not know<br>○ e no ansa
715b.i. Wetin be the first thing wey she do to take comot the belle?	(${friend2_abt_mult_yn} = 'yes') or (${friend2_abt_mult_yn} = 'likely')<br>○ Surgical procedure<br>○ Pills called mifepristone or misoprostol, for example Mariprist,Mifepak, Cytotec, Miso-Fem, or Misoclear<br>○ Pills you take when you have a fever like antibiotics or anti-malarial medicine, for example quinine<br>○ Emergency contraception pills, for example Postinor<br>○ Other pills<br>○ injection<br>○ Traditional methods, like herbs<br>○ Alcohol<br>○ Salt, potash, maggi, or kanwa<br>○ Lemon or lime<br>○ Cough syrup<br>○ Insert materials into the vagina<br>○ Other<br>○ Do not know<br>○ e no ansa
715b.i. Wetin she do to comot the belle?	(${friend2_abt_mult_yn} = 'no') or (${friend2_abt_mult_yn} = '-88')<br>○ Surgical procedure

	◌ Pills called mifepristone or misoprostol, for example Mariprist, Mifepak, Cytotec, Miso-Fem, or Misoclear<br>◌ Pills you take when you have a fever like antibiotics or anti-malarial medicine, for example quinine<br>◌ Emergency contraception pills, for example Postinor<br>◌ Other pills<br>◌ injection<br>◌ Traditional methods, like herbs<br>◌ Alcohol<br>◌ Salt, potash, maggi, or kanwa<br>◌ Lemon or lime<br>◌ Cough syrup<br>◌ Insert materials into the vagina<br>◌ Other<br>◌ Do not know<br>◌ e no ansa
716b.i. Which place she go for the method?	(${friend2_abt_first} = 'surgery') or (${friend2_abt_only} = 'surgery')<br>◌ Government Hospital<br>◌ Government Health Center<br>◌ Family planning clinic<br>◌ Mobile clinic (public)<br>◌ TBA/Fieldworker (public)<br>◌ Private hospital/clinic<br>◌ Pharmacy<br>◌ Chemist/PMS Store<br>◌ Private doctor or nurse<br>◌ Mobile clinic (private)<br>◌ TBA/Fieldworker (private)<br>◌ Shop<br>◌ FBO/Church<br>◌ Friend / relative<br>◌ NGO<br>◌ Market / hawking<br>◌ Other<br>◌ Do not know<br>◌ e no ansa
717b.i. Which place she get the medicine?	(${friend2_abt_first} = 'pills_abortion') or (${friend2_abt_only} = 'pills_abortion') or (${friend …<br>◌ Government Hospital<br>◌ Government Health Center<br>◌ Family planning clinic<br>◌ Mobile clinic (public)<br>◌ TBA/Fieldworker (public)<br>◌ Private hospital/clinic<br>◌ Pharmacy<br>◌ Chemist/PMS Store<br>◌ Private doctor or nurse<br>◌ Mobile clinic (private)<br>◌ TBA/Fieldworker (private)<br>◌ Shop<br>◌ FBO/Church<br>◌ Friend / relative<br>◌ NGO<br>◌ Market / hawking<br>◌ Other<br>◌ Do not know

	○ e no ansa
718b.i. Wetin be the last tin she do wey make belle comot?	(${friend2_abt_mult_yn} = 'yes') or (${friend2_abt_mult_yn} = 'likely')<br>○ Surgical procedure<br>○ Pills called mifepristone or misoprostol, for example Mariprist, Mifepak, Cytotec, Miso-Fem, or Misoclear<br>○ Pills you take when you have a fever like antibiotics or anti-malarial medicine, for example quinine<br>○ Emergency contraception pills, for example Postinor<br>○ Other pills<br>○ injection<br>○ Traditional methods, like herbs<br>○ Alcohol<br>○ Salt, potash, maggi, or kanwa<br>○ Lemon or lime<br>○ Cough syrup<br>○ Insert materials into the vagina<br>○ Other<br>○ Do not know<br>○ e no ansa
719b.i. Na where she go comot the belle?	${friend2_abt_last} = 'surgery'<br>○ Government Hospital<br>○ Government Health Center<br>○ Family planning clinic<br>○ Mobile clinic (public)<br>○ TBA/Fieldworker (public)<br>○ Private hospital/clinic<br>○ Pharmacy<br>○ Chemist/PMS Store<br>○ Private doctor or nurse<br>○ Mobile clinic (private)<br>○ TBA/Fieldworker (private)<br>○ Shop<br>○ FBO/Church<br>○ Friend / relative<br>○ NGO<br>○ Market / hawking<br>○ Other<br>○ Do not know<br>○ e no ansa
720b.i. Na where she get the medicine to comot the belle?	(${friend2_abt_last} = 'pills_abortion') or (${friend2_abt_last} = 'pills_fever') or (${friend2_ab …<br>○ Government Hospital<br>○ Government Health Center<br>○ Family planning clinic<br>○ Mobile clinic (public)<br>○ TBA/Fieldworker (public)<br>○ Private hospital/clinic<br>○ Pharmacy<br>○ Chemist/PMS Store<br>○ Private doctor or nurse<br>○ Mobile clinic (private)<br>○ TBA/Fieldworker (private)<br>○ Shop<br>○ FBO/Church<br>○ Friend / relative<br>○ NGO

	◌ Market / hawking<br>◌ Other<br>◌ Do not know<br>◌ e no ansa
721b.i. Shey ${friend2_name} get any problem wey make am go health facility as she bin dey try to comot the belle?<br>*If the respondent don already say her friend go health facility go comot belle, we wan know if the friend go back for treatment for palava wey she dey see.*	(${friend2_abt_yn} = 'yes') or (${friend2_abt_yn} = 'likely')<br>◌ Yes, I dey sure<br>◌ Yes, E be like<br>◌ No<br>◌ Do not know<br>◌ e no ansa
712b.ii. Apart from all dis one wey we don talk about. ${friend2_name} don do anything before to regulate her period when she dey fear say she get belle?<br>*Probe to confirm whether the period regulation was successful. If not, select 'no.'*	${friend2_abt_yn} = 'yes'<br>◌ Yes, I dey sure<br>◌ Yes, E be like<br>◌ No<br>◌ Do not know<br>◌ e no ansa
712b.ii. Shey ${friend2_name} don do anything before to make her period come as she dey fear say she get belle?<br>*Probe to confirm whether the period regulation was successful. If not, select 'no.'*	${friend2_abt_yn} != 'yes'<br>◌ Yes, I dey sure<br>◌ Yes, E be like<br>◌ No<br>◌ Do not know<br>◌ e no ansa
713b.ii. Which year this one happen?<br>*If indicates happened more than once, specify most recent time.*<br>*Enter 2020 for 'Do not know' or 'No response'.*	(${friend2_reg_yn} = 'yes') or (${friend2_reg_yn} = 'likely')<br>Year: ________________________________
714b.ii. Plenty women dey do many things to make dere period come. You know werda ${friend2_name} do pass one thing to make her period come?	((${friend2_reg_year} > ${friend2_abt_year}) or (${friend2_abt_year} = '')) and ((${friend2_reg_yn …<br>◌ Yes, I dey sure<br>◌ Yes, E be like<br>◌ No<br>◌ Do not know<br>◌ e no ansa
715b.ii. Wetin be the first thing wey she do to make her period come out?	((${friend2_reg_year} > ${friend2_abt_year}) or (${friend2_abt_year} = '')) and ((${friend2_reg_mu …<br>◌ Surgical procedure<br>◌ Pills called mifepristone or misoprostol, for example Mariprist, Mifepak, Cytotec, Miso-Fem, or Misoclear<br>◌ Pills you take when you have a fever like antibiotics or anti-malarial medicine, for example quinine<br>◌ Emergency contraception pills, for example Postinor<br>◌ Other pills<br>◌ injection<br>◌ Traditional methods, like herbs<br>◌ Alcohol<br>◌ Salt, potash, maggi, or kanwa<br>◌ Lemon or lime<br>◌ Cough syrup<br>◌ Insert materials into the vagina<br>◌ Other<br>◌ Do not know<br>◌ e no ansa
715b.ii. Wetin she do weymake her period come out?	((${friend2_reg_year} > ${friend2_abt_year}) or (${friend2_abt_year} = '')) and ((${friend2_reg_mu …<br>◌ Surgical procedure

	○ Pills called mifepristone or misoprostol, for example Mariprist, Mifepak, Cytotec, Miso-Fem, or Misoclear<br>○ Pills you take when you have a fever like antibiotics or anti-malarial medicine, for example quinine<br>○ Emergency contraception pills, for example Postinor<br>○ Other pills<br>○ injection<br>○ Traditional methods, like herbs<br>○ Alcohol<br>○ Salt, potash, maggi, or kanwa<br>○ Lemon or lime<br>○ Cough syrup<br>○ Insert materials into the vagina<br>○ Other<br>○ Do not know<br>○ e no ansa
716b.ii. Wia she go do am?	((${friend2_reg_year} > ${friend2_abt_year}) or (${friend2_abt_year} = '')) and ((${friend2_reg_fi …<br>○ Government Hospital<br>○ Government Health Center<br>○ Family planning clinic<br>○ Mobile clinic (public)<br>○ TBA/Fieldworker (public)<br>○ Private hospital/clinic<br>○ Pharmacy<br>○ Chemist/PMS Store<br>○ Private doctor or nurse<br>○ Mobile clinic (private)<br>○ TBA/Fieldworker (private)<br>○ Shop<br>○ FBO/Church<br>○ Friend / relative<br>○ NGO<br>○ Market / hawking<br>○ Other<br>○ Do not know<br>○ e no ansa
717b.ii. Wia she go buy the medicine?	((${friend2_reg_year} > ${friend2_abt_year}) or (${friend2_abt_year} = '')) and ((${friend2_reg_fi …<br>○ Government Hospital<br>○ Government Health Center<br>○ Family planning clinic<br>○ Mobile clinic (public)<br>○ TBA/Fieldworker (public)<br>○ Private hospital/clinic<br>○ Pharmacy<br>○ Chemist/PMS Store<br>○ Private doctor or nurse<br>○ Mobile clinic (private)<br>○ TBA/Fieldworker (private)<br>○ Shop<br>○ FBO/Church<br>○ Friend / relative<br>○ NGO<br>○ Market / hawking<br>○ Other

	○ Do not know<br>○ e no ansa
718b.ii. Wetin be the last thing wey she do wey make the period come out?	((${friend2_reg_year} > ${friend2_abt_year}) or (${friend2_abt_year} = '')) and ((${friend2_reg_mu …<br>○ Surgical procedure<br>○ Pills called mifepristone or misoprostol, for example Mariprist, Mifepak, Cytotec, Miso-Fem, or Misoclear<br>○ Pills you take when you have a fever like antibiotics or anti-malarial medicine, for example quinine<br>○ Emergency contraception pills, for example Postinor<br>○ Other pills<br>○ injection<br>○ Traditional methods, like herbs<br>○ Alcohol<br>○ Salt, potash, maggi, or kanwa<br>○ Lemon or lime<br>○ Cough syrup<br>○ Insert materials into the vagina<br>○ Other<br>○ Do not know<br>○ e no ansa
719b.ii. Wia she go do am?	((${friend2_reg_year} > ${friend2_abt_year}) or (${friend2_abt_year} = '')) and (${friend2_reg_las …<br>○ Government Hospital<br>○ Government Health Center<br>○ Family planning clinic<br>○ Mobile clinic (public)<br>○ TBA/Fieldworker (public)<br>○ Private hospital/clinic<br>○ Pharmacy<br>○ Chemist/PMS Store<br>○ Private doctor or nurse<br>○ Mobile clinic (private)<br>○ TBA/Fieldworker (private)<br>○ Shop<br>○ FBO/Church<br>○ Friend / relative<br>○ NGO<br>○ Market / hawking<br>○ Other<br>○ Do not know<br>○ e no ansa
720b.ii. Wia she go buy the medicine?	((${friend2_reg_year} > ${friend2_abt_year}) or (${friend2_abt_year} = '')) and ((${friend2_reg_la …<br>○ Government Hospital<br>○ Government Health Center<br>○ Family planning clinic<br>○ Mobile clinic (public)<br>○ TBA/Fieldworker (public)<br>○ Private hospital/clinic<br>○ Pharmacy<br>○ Chemist/PMS Store<br>○ Private doctor or nurse<br>○ Mobile clinic (private)<br>○ TBA/Fieldworker (private)<br>○ Shop

	FBO/Church<br>Friend / relative<br>NGO<br>Market / hawking<br>Other<br>Do not know<br>e no ansa
721b.ii. Your second ${friend2_name} bin get any wahala wen she bin dey try to make her period to come out well wey make her go hospital to treat herself?<br>*If the respondent already reported the friend went to a health facility in the process of regulating her period, we are interested in whether the friend went back to a health facility on a separate occasion to treat complications that she may have experienced.*	((${friend2_reg_year} > ${friend2_abt_year}) or (${friend2_abt_year} = '')) and ((${friend2_reg_yn …<br>Yes, I dey sure<br>Yes, E be like<br>No<br>Do not know<br>e no ansa
722a. Now, I go ask you your own.You don ever do something to comot belle before or when you bin dey fear say you carry belle?<br>*Probe to confirm whether the pregnancy removal was successful.*	Yes<br>No<br>e no ansa
723a. Which year wey e happen last?<br>*If indicates happened more than once, specify most recent time.*<br>*Enter 2020 for 'Do not know' or 'No response'.*	(${self_abt_yn} = 'yes')<br>Year: ___________
724a. How many tins you do to comot the belle?	(${self_abt_yn} = 'yes')<br>Yes<br>No<br>e no ansa
725a. Wetin you first do?	(${self_abt_mult_yn} = 'yes')<br>Surgical procedure<br>Pills called mifepristone or misoprostol, for example Mariprist, Mifepak, Cytotec, Miso-Fem, or Misoclear<br>Pills you take when you have a fever like antibiotics or anti-malarial medicine, for example quinine<br>Emergency contraception pills, for example Postinor<br>Other pills<br>injection<br>Traditional methods, like herbs<br>Alcohol<br>Salt, potash, maggi, or kanwa<br>Lemon or lime<br>Cough syrup<br>Insert materials into the vagina<br>Other<br>Do not know<br>e no ansa
725a. Wetin you do?	(${self_abt_mult_yn} = 'no')<br>Surgical procedure<br>Pills called mifepristone or misoprostol, for example Mariprist, Mifepak, Cytotec, Miso-Fem, or Misoclear<br>Pills you take when you have a fever like antibiotics or anti-malarial medicine, for example quinine<br>Emergency contraception pills, for example Postinor<br>Other pills<br>injection<br>Traditional methods, like herbs

	◌ Alcohol<br>◌ Salt, potash, maggi, or kanwa<br>◌ Lemon or lime<br>◌ Cough syrup<br>◌ Insert materials into the vagina<br>◌ Other<br>◌ Do not know<br>◌ e no ansa
726a. Wia you go do am?	(${self_abt_first} = 'surgery') or (${self_abt_only} = 'surgery')<br>◌ Government Hospital<br>◌ Government Health Center<br>◌ Family planning clinic<br>◌ Mobile clinic (public)<br>◌ TBA/Fieldworker (public)<br>◌ Private hospital/clinic<br>◌ Pharmacy<br>◌ Chemist/PMS Store<br>◌ Private doctor or nurse<br>◌ Mobile clinic (private)<br>◌ TBA/Fieldworker (private)<br>◌ Shop<br>◌ FBO/Church<br>◌ Friend / relative<br>◌ NGO<br>◌ Market / hawking<br>◌ Other<br>◌ Do not know<br>◌ e no ansa
727a. Wia you go buy the medicines?	(${self_abt_first} = 'pills_abortion') or (${self_abt_only} = 'pills_abortion') or (${self_abt_fir …<br>◌ Government Hospital<br>◌ Government Health Center<br>◌ Family planning clinic<br>◌ Mobile clinic (public)<br>◌ TBA/Fieldworker (public)<br>◌ Private hospital/clinic<br>◌ Pharmacy<br>◌ Chemist/PMS Store<br>◌ Private doctor or nurse<br>◌ Mobile clinic (private)<br>◌ TBA/Fieldworker (private)<br>◌ Shop<br>◌ FBO/Church<br>◌ Friend / relative<br>◌ NGO<br>◌ Market / hawking<br>◌ Other<br>◌ Do not know<br>◌ e no ansa
728a. Wetin be the last thing wey you do to commot the belle?	(${self_abt_mult_yn} = 'yes')<br>◌ Surgical procedure<br>◌ Pills called mifepristone or misoprostol, for example Mariprist, Mifepak, Cytotec, Miso-Fem, or Misoclear<br>◌ Pills you take when you have a fever like antibiotics or anti-malarial medicine, for example quinine<br>◌ Emergency contraception pills, for example Postinor

	○ Other pills<br>○ injection<br>○ Traditional methods, like herbs<br>○ Alcohol<br>○ Salt, potash, maggi, or kanwa<br>○ Lemon or lime<br>○ Cough syrup<br>○ Insert materials into the vagina<br>○ Other<br>○ Do not know<br>○ e no ansa
729a. Wia you go do am?	${self_abt_last} = 'surgery'<br>○ Government Hospital<br>○ Government Health Center<br>○ Family planning clinic<br>○ Mobile clinic (public)<br>○ TBA/Fieldworker (public)<br>○ Private hospital/clinic<br>○ Pharmacy<br>○ Chemist/PMS Store<br>○ Private doctor or nurse<br>○ Mobile clinic (private)<br>○ TBA/Fieldworker (private)<br>○ Shop<br>○ FBO/Church<br>○ Friend / relative<br>○ NGO<br>○ Market / hawking<br>○ Other<br>○ Do not know<br>○ e no ansa
730a. Wia you buy the medicines?	(${self_abt_last} = 'pills_abortion') or (${self_abt_last} = 'pills_fever') or (${self_abt_last} = …<br>○ Government Hospital<br>○ Government Health Center<br>○ Family planning clinic<br>○ Mobile clinic (public)<br>○ TBA/Fieldworker (public)<br>○ Private hospital/clinic<br>○ Pharmacy<br>○ Chemist/PMS Store<br>○ Private doctor or nurse<br>○ Mobile clinic (private)<br>○ TBA/Fieldworker (private)<br>○ Shop<br>○ FBO/Church<br>○ Friend / relative<br>○ NGO<br>○ Market / hawking<br>○ Other<br>○ Do not know<br>○ e no ansa
731a. You bin get any wahala wey make you go hospital becos you dey try to comot the belle?<br>*If the respondent already reported she went to a health facility in the process of removing the pregnancy, we are interested in whether she went back to a health facility on a separate occasion to treat complications that she may have experienced.*	(${self_abt_yn} = 'yes')<br>○ Yes<br>○ No<br>○ Do not know<br>○ e no ansa
732a. You bin tell any of dis persin about the mata?<br>*Read the answer choices aloud. Select all that apply.*	(${self_abt_yn} = 'yes')<br>☐ Husband/male partner<br>☐ Sister

	☐ Brother<br>☐ Mother<br>☐ Father<br>☐ Other relative<br>☐ Friend 1: ${friend1_name}<br>☐ Friend 2: ${friend2_name}<br>☐ Other friend<br>☐ Other<br>☐ Do not know<br>☐ e no ansa<br>(${friend1_name} != '' and ${friend1_name} != '-99' and filter = 'friend1') or (${friend2_name} != '' and ${friend2_name} != '-99' and filter = 'friend2') or (filter = 'always')
722b. Apart from this one wen you don talk, you don ever do anything to make your period come out when you dey fear say you carry belle?<br>*Probe to confirm whether the period regulation was successful. If not, select 'no.'*	${self_abt_yn} = 'yes'<br>○ Yes<br>○ No<br>○ e no ansa
722b. U don ever do anything to make your period come out again when u fear say you get belle?<br>*Probe to confirm whether the period regulation was successful. If not, select 'no.'*	${self_abt_yn} != 'yes'<br>○ Yes<br>○ No<br>○ e no ansa
723b. Na which year e happen last?<br>*If indicates happened more than once, specify most recent time.*<br>*Enter 2020 for 'Do not know' or 'No response'.*	(${self_reg_yn} = 'yes')<br>Year: ______________________
724b. You do different things to make the period come out?	((${self_reg_year} > ${self_abt_year}) or (${self_abt_year} = '')) and ((${self_reg_yn} = 'yes'))<br>○ Yes<br>○ No<br>○ e no ansa
725b. Wetin you first do?	((${self_reg_year} > ${self_abt_year}) or (${self_abt_year} = '')) and ((${self_reg_mult_yn} = 'ye …<br>○ Surgical procedure<br>○ Pills called mifepristone or misoprostol, for example Mariprist, Mifepak, Cytotec, Miso-Fem, or Misoclear<br>○ Pills you take when you have a fever like antibiotics or anti-malarial medicine, for example quinine<br>○ Emergency contraception pills, for example Postinor<br>○ Other pills<br>○ injection<br>○ Traditional methods, like herbs<br>○ Alcohol<br>○ Salt, potash, maggi, or kanwa<br>○ Lemon or lime<br>○ Cough syrup<br>○ Insert materials into the vagina<br>○ Other<br>○ Do not know<br>○ e no ansa
725b. Wetin you do?	((${self_reg_year} > ${self_abt_year}) or (${self_abt_year} = '')) and ((${self_reg_mult_yn} = 'no …<br>○ Surgical procedure<br>○ Pills called mifepristone or misoprostol, for example Mariprist, Mifepak, Cytotec, Miso-Fem, or Misoclear

	◌ Pills you take when you have a fever like antibiotics or anti-malarial medicine, for example quinine<br>◌ Emergency contraception pills, for example Postinor<br>◌ Other pills<br>◌ injection<br>◌ Traditional methods, like herbs<br>◌ Alcohol<br>◌ Salt, potash, maggi, or kanwa<br>◌ Lemon or lime<br>◌ Cough syrup<br>◌ Insert materials into the vagina<br>◌ Other<br>◌ Do not know<br>◌ e no ansa
726b. Wia you go do am?	((${self_reg_year} > ${self_abt_year}) or (${self_abt_year} = '')) and ((${self_reg_first} = 'surg …<br>◌ Government Hospital<br>◌ Government Health Center<br>◌ Family planning clinic<br>◌ Mobile clinic (public)<br>◌ TBA/Fieldworker (public)<br>◌ Private hospital/clinic<br>◌ Pharmacy<br>◌ Chemist/PMS Store<br>◌ Private doctor or nurse<br>◌ Mobile clinic (private)<br>◌ TBA/Fieldworker (private)<br>◌ Shop<br>◌ FBO/Church<br>◌ Friend / relative<br>◌ NGO<br>◌ Market / hawking<br>◌ Other<br>◌ Do not know<br>◌ e no ansa
727b. Wia you buy the medicines?	((${self_reg_year} > ${self_abt_year}) or (${self_abt_year} = '')) and ((${self_reg_first} = 'pill …<br>◌ Government Hospital<br>◌ Government Health Center<br>◌ Family planning clinic<br>◌ Mobile clinic (public)<br>◌ TBA/Fieldworker (public)<br>◌ Private hospital/clinic<br>◌ Pharmacy<br>◌ Chemist/PMS Store<br>◌ Private doctor or nurse<br>◌ Mobile clinic (private)<br>◌ TBA/Fieldworker (private)<br>◌ Shop<br>◌ FBO/Church<br>◌ Friend / relative<br>◌ NGO<br>◌ Market / hawking<br>◌ Other<br>◌ Do not know<br>◌ e no ansa
728b. Wetin you do wey e come out finally?	((${self_reg_year} > ${self_abt_year}) or (${self_abt_year} = '')) and ((${self_reg_mult_yn} = 'ye …

- Surgical procedure
- Pills called mifepristone or misoprostol, for example Mariprist, Mifepak, Cytotec, Miso-Fem, or Misoclear
- Pills you take when you have a fever like antibiotics or anti-malarial medicine, for example quinine
- Emergency contraception pills, for example Postinor
- Other pills
- injection
- Traditional methods, like herbs
- Alcohol
- Salt, potash, maggi, or kanwa
- Lemon or lime
- Cough syrup
- Insert materials into the vagina
- Other
- Do not know
- e no ansa

729b. Wia you go do am?

((${self_reg_year} > ${self_abt_year}) or (${self_abt_year} = '')) and (${self_reg_last} = 'surger …

- Government Hospital
- Government Health Center
- Family planning clinic
- Mobile clinic (public)
- TBA/Fieldworker (public)
- Private hospital/clinic
- Pharmacy
- Chemist/PMS Store
- Private doctor or nurse
- Mobile clinic (private)
- TBA/Fieldworker (private)
- Shop
- FBO/Church
- Friend / relative
- NGO
- Market / hawking
- Other
- Do not know
- e no ansa

730b. Wia you go buy the medicines?

((${self_reg_year} > ${self_abt_year}) or (${self_abt_year} = '')) and ((${self_reg_last} = 'pills …

- Government Hospital
- Government Health Center
- Family planning clinic
- Mobile clinic (public)
- TBA/Fieldworker (public)
- Private hospital/clinic
- Pharmacy
- Chemist/PMS Store
- Private doctor or nurse
- Mobile clinic (private)
- TBA/Fieldworker (private)
- Shop
- FBO/Church
- Friend / relative
- NGO
- Market / hawking
- Other

	○ Do not know<br>○ e no ansa
731b. That time wen you bin try bring back your period, you bin get any problem wen make you go to health centre?<br>*If the respondent already reported she went to a health facility in the process of regulating her period, we are interested in whether she went back to a health facility on a separate occasion to treat complications that she may have experienced.*	((${self_reg_year} > ${self_abt_year}) or (${self_abt_year} = '')) and ((${self_reg_yn} = 'yes'))<br>○ Yes<br>○ No<br>○ Do not know<br>○ e no ansa
732b. You tell any of dis persin say you dey go there?<br>*Read the answer choices aloud. Select all that apply.*	((${self_reg_year} > ${self_abt_year}) or (${self_abt_year} = '')) and ((${self_reg_yn} = 'yes'))<br>☐ Husband/male partner<br>☐ Sister<br>☐ Brother<br>☐ Mother<br>☐ Father<br>☐ Other relative<br>☐ Friend 1: ${friend1_name}<br>☐ Friend 2: ${friend2_name}<br>☐ Other friend<br>☐ Other<br>☐ Do not know<br>☐ e no ansa<br>(${friend1_name} != '' and ${friend1_name} != '-99' and filter = 'friend1') or (${friend2_name} != '' and ${friend2_name} != '-99' and filter = 'friend2') or (filter = 'always')

	${consent_obtained}
Now I wan ask you some questions about removing belle. Abeg, tell me if you: agree well well, just agree, I no sure, disagree, no agree at all at all<br>*Check box to confirm scrolled to bottom.*	
Press OK to continue	○ na so

733. E dey okay for woman to commot belle if the belle dey worry her health.	${consent_obtained}<br>○ Agree well well<br>○ Just agree<br>○ I no sure<br>○ Disagree<br>○ No agree at all at all<br>○ e no ansa
734. E dey okay for woman to commot belle if dem force her sleep with her	${consent_obtained}<br>○ Agree well well<br>○ Just agree<br>○ I no sure<br>○ Disagree<br>○ No agree at all at all<br>○ e no ansa
735. E dey okay for woman to commot belle if she no fit take care of another pikin	${consent_obtained}<br>○ Agree well well<br>○ Just agree<br>○ I no sure<br>○ Disagree<br>○ No agree at all at all<br>○ e no ansa
736. E dey okay for woman to commot belle if she no want the pikin.	${consent_obtained}<br>○ Agree well well<br>○ Just agree<br>○ I no sure<br>○ Disagree<br>○ No agree at all at all<br>○ e no ansa

737. Woman wey commot belle don bring shame to her family.	${consent_obtained}<br>○ Agree well well<br>○ Just agree<br>○ I no sure<br>○ Disagree<br>○ No agree at all at all<br>○ e no ansa
738. Woman wey commot belle, make she no tell anybody?	${consent_obtained}<br>○ Agree well well<br>○ Just agree<br>○ I no sure<br>○ Disagree<br>○ No agree at all at all<br>○ e no ansa

Follow-up Consent

FLW_801. Thank you for the time wey you give us.<br>We go happy to hear how you commot your belle and how you make your period come out and the method wey you use and wia you do am. You go like to see another woman for this project for another day so that you go fit talk about wetin you don see and wetin you know	(${self_abt_yn} = 'yes') or (${self_reg_yn} = 'yes')<br>○ Yes<br>○ No<br>○ e no ansa
FLW_802. You get phone?	${flw_willing} = 'yes'<br>○ Yes<br>○ No<br>○ e no ansa
FLW_803. Abeg, you fit give me the number in case we wan talk to you later?<br>*Enter an 11-digit number without the country code. Do not include spaces or dashes. Enter 0 for no response.*	${flw_number_yn} = 'yes'<br>--
FLW_804. Tell me the number again?<br>*Enter an 11-digit number without the country code. Do not include spaces or dashes. Enter 0 for no response.*	(${flw_number_typed} != '') and (${flw_number_typed} != '0')<br>--
Thank the respondent for her time.<br>*The respondent don finish, but there are still more questions you go complete outside the home.*	${available} = 'yes'

Location and Questionnaire result

095. Location<br>*Make you take GPS point of the front of the house. Make you record location when the accuracy dey smaller than 6m. Na outside you fit collect GPS cordinates*	Always
096. How many times have you visited this household to interview this female respondent?	Always<br>○ 1st time<br>○ 2nd time<br>○ 3rd time
097. In what language was this interview conducted?	009a = 1<br>○ English<br>○ Hausa<br>○ Igbo<br>○ Yoruba<br>○ Pidgin<br>○ Other
