# Supplementary material for: Measurement of abortion safety using community-based surveys: Findings from three countries
Source: PLoS One. 2019 Nov 7;14(11):e0223146. doi: 10.1371/journal.pone.0223146 (PMC6837422; doi:10.1371/journal.pone.0223146)
Supplement: S5 Doc — (PDF) [file pone.0223146.s005.pdf]

S7 Doc.

NGR5-Female-Questionnaire-v36-aso.xlsx

001a. Nje idile to too lo wa? EA: [EA entered in the Household Questionnaire] Structure #: [Structure entered in the Household Questionnaire] Household #: [Household entered in the Household Questionnaire]	gbogbo gba<br>◯ Beeni<br>◯ Beeko
002. Te oruko re si isale<br>*Jowo ko oruko re*	002 = 0<br>--
003b. Ko Igba ati akoko to boju mu	003 = 0<br>Day: ----------------------------------<br>Month: ----------------------------------<br>Year: ----------------------------------

The following information is from the Household Questionnaire. Please review to make sure you are interviewing the correct respondent. [ODK will display the State, LGA, Enumeration Area, Structure Number, and Household Number entered into the Household Questionnaire linked to this Female Questionnaire.] Is the above information correct?	gbogbo gba
State: ${level1_unlinked}	Ipinle: [STATE]<br>--
LGA: ${level2_unlinked}	LGA: [LGA]<br>--
Locality: ${level3_unlinked}	Locality: [LOCALITY]<br>--
Enumeration Area: [EA]	--
Structure number: [#]	--
Household number: [#]	--
004b. Nje awon ohun won yen baramu?	004 = 0<br>◯ Beeni<br>◯ Beeko

005. CHECK: O ni la ti fe bere si ni se ibeere [Name of the interviewee]. N je beeni?<br>*Ti O ba si pe, mu "beeni" ki o si tun oruko ko ni ibeere "011". Ti o ba je eni ti ko ye, o ni nkan me ji la ti se:*<br>*(1) kuro, ki o si paro foomu*<br>*tabi*<br>*(2) wa eni ti oruko re yoo loke yi ki o si bere ibeere lowo re.*	gbogbo gba<br>◯ Beeni<br>◯ Beeko
006. Nje onitoun wa nile ati wipe osetan lati gba ki afi oro wanu wo looni?	gbogbo gba<br>◯ Beeni<br>◯ Beeko
007. Bawo lo se sun mo oludahun ibeere si?	006 = 1<br>◯ Osun mo daada<br>◯ Eni mimo<br>◯ ko mọ daada<br>◯ ko mọ rara
008. Nje oludahun ti ko pa ri ninu awon iwadi PMA2020 kankan latehinwa?	006 = 1<br>◯ Beeni<br>◯ Beeko<br>◯ N ko mo<br>◯ ko si esi
GBIGBA IYANDA<br>*Wa obinrin ti ojo ori wa laarin 15-49 ti o ro mo ibeere awon obinrin. Enikeni ko gbodo gbo ohun ti e n so. Ka awon ikini yii*	(${available} = 'yes') and (not(${unlinked}) or ${proceed_with_unlinked})
E nle. Oruko mi ni ____________________________________ mo n sise pelu Center for Research, Evaluation Resources, and Development. A nse iwadi ninu eyi ti a n bi awon obinrin nipa eto	(${available} = 'yes') and (not(${unlinked}) or ${proceed_with_unlinked})

ilera nipa omo bibi. Inu wa yio dun ti e ba le kopa nibi iwadi yi, yio ranwa lowo lati le bere fun eto ilera to pe ye lati odo ijoba. Iwadi yi yio gba iseju meedogun si ogun iseju. Awon idahun yin si ibeere yi yio je bonkele ti a ko ni fi han enikeni yato si awa ti a jo n se iwadi yii. Ki kopa yin ninu iwadi yii kii se tipa, ati pe bi aba bere ibere kan ti e ko fe dahun, e jeki n mo ma si koja lo si ori ibere miran; e le da iforo wani lenu wo na duro nigbakugba. Bakanna, a lero pe e o ko pa ninu iwadi yii nitori pe imoran yin ninu iwadi yi yio wulo fun wa pupo.<br>If you have any questions about the study and your right as a research participant, you may ask me now or you may also contact Dr. Elizabeth Omoluobi at Center for Research, Evaluation Resources and Development in Ile-ife, Nigeria at +2348033816486.<br>Ni asiko yi, se eni ohun ti e fe bere lodo mi nipa iwadi yii?	
009a. Se mo le bere iforo wani lenuwo na bayi ?	(${available} = 'yes') and (not(${unlinked}) or ${proceed_with_unlinked})<br>○ Beeni<br>○ Beeko
010. Oruko olubere Jowo ko oruko re gege bi olujeri si gbigba iyanda O ti ko oruko re lekan pe "[Interviewer's name]."	${consent_obtained} and (${your_name_check} = 'no')<br>________________

Section 1 – Respondent's Background, Marital Status, Household characteristics

Bayi Emi yoo fẹ lati beere nipa rẹ lẹhin ki o si latari ipo.

	${consent_obtained}
101. Inu osu ati odun wo ni won bi yin? Oju ori ninu iwe akosile idile ni [AGE]<br>*Select 'Do not know' for month and '2020' for year to indicate 'No Response'.*	009a = 1
101. Ninu osu ati odun wo ni a bi yin?<br>*Select 'Do not know' for month and '2020' for year to indicate 'No Response'.*	009a = 1
Month:	○ January<br>○ February<br>○ March<br>○ April<br>○ May<br>○ June<br>○ July<br>○ August<br>○ September<br>○ October<br>○ November<br>○ December<br>○ N ko mo
Odun:	Year: ________________

102. Omo odun melo ni yin nigba ti e se ojo ibi kehin?	009a = 1<br>________________
103. Kini ipele eko ti eka?<br>*jowo ko ile-iwe ti o lo ni kan. Mase ko ti ile iwe bibeli tabi ile keu*	009a = 1<br>○ Ko lo rii<br>○ Ile-iwe Alakobere<br>○ Ile-iwe sekondiri<br>○ Iwe giga<br>○ ko si esi
104. Se e e wa nile/lode oko bayi abi e n gbe pelu okunrin kan bi oko ati iyawo?<br>*Ibeere: Bi beko, beere lowo olukopa boya oun ati oko re ti ko ara won, boya won gba lati ma gbe lotooto, tabi opo ni.*	009a = 1<br>○ Beeni, owa ni ile oko<br>○ Beeni, o n gbe pelu okunrin<br>○ Ko Lọwọlọwọ ni Euroopu: Ìkọrasílẹ / yà

	○ Ko Lọwọlọwọ ni Euroopu: Opó<br>○ rara, ko si ni ile oko<br>○ ko si esi
105. Se e ti segbeyawo ri tabi gbe pelu okunrin kan leekan soso ri tabi ju bee lo?	104 ≠ 5<br>○ Ni e kan so so<br>○ Oju igba kan lo<br>○ ko si esi

	(${marriage_history} = 'more_than_once')
106a. Ninu osu ati odun wo ni e bere si gbe po pelu oko yin ti e koko fe rii/alabagbepo yin?<br>*Select 'Do not know' for month and '2020' for year to indicate 'No Response'.*	105=2
Month:	○ January<br>○ February<br>○ March<br>○ April<br>○ May<br>○ June<br>○ July<br>○ August<br>○ September<br>○ October<br>○ November<br>○ December<br>○ N ko mo
Odun:	Year: ______________

106b. Ṣayẹwo: lori awọn esi ti o ti tẹ ni 106a, awọn oludahun wà je omo ọdun meedogun kéré tabi po ni igba ti igbeyawo akoko<br>Ṣe o tẹ 106a bi o ti ye ko ri?	106a age at marriage ≤15<br>○ Beeni<br>○ Beeko

	(${marriage_history} = 'once') or (${marriage_history} = 'more_than_once')
107a. Ni bayi mo fe beere nipa igba ti e bere si ngbe pelu oko/alabagbepo ti e waa lodo re yii, tabi eyi ti e ni gbeyin? Ninu osu ati odun wo ni e bere si gbe papo?<br>*Select 'Do not know' for month and '2020' for year to indicate 'No Response'.*	105 = 1 or 2
Month:	○ January<br>○ February<br>○ March<br>○ April<br>○ May<br>○ June<br>○ July<br>○ August<br>○ September<br>○ October<br>○ November<br>○ December<br>○ N ko mo
Odun:	Year: ______________

107b. Ṣayẹwo: lori awọn esi ti o ti tẹ ni 107a, awọn oludahun wà je omo ọdun meedogun kéré tabi po ni igba ti igbeyawo akoko<br>Ṣe o tẹ 107a bi o ti ye ko ri?	107a age at marriage ≤15<br>○ Beeni<br>○ Beeko
108. Se oko yin/alabagbepo yin tun ni awon iyawo miran tabi awon obinrin miran ti won fe yato si yin?	104 = 1 or 2<br>○ Beeni<br>○ Beeko<br>○ N ko mo<br>○ ko si esi

Ipin keji – Oro ni pa bi a ti se n bimo, Oyun nini ati irufe omo ti won n wa

Nibayii maa fe beere nipa gbogbo ikunle ti e ti ni ri

200. Bayi mo fe bere nipa awon omo ti e ti bi ni gba aye yin. Se e ti bi mo ri?	009a = 1<br>○ Beeni<br>○ Beeko<br>○ ko si esi
201. How many times have you given birth?<br>*Ko -99 fun ko le si.*	200 = 1<br>________________

	(${birth_events} > 1)
205. Igba wo ni e bi omo akoko?<br>*Please record the date of the FIRST birth. The date should be found by calculating backwards from memorable events if needed.*<br>*Select 'Do not know' for month and '2020' for year to indicate 'No Response'.*	201 > 1
Month:	○ January<br>○ February<br>○ March<br>○ April<br>○ May<br>○ June<br>○ July<br>○ August<br>○ September<br>○ October<br>○ November<br>○ December<br>○ N ko mo
Odun:	Year: ________________

	(${birth_events} > 0)
206. Igba wo ni e bi omo abikeyin?<br>*Select 'Do not know' for month and '2020' for year to indicate 'No Response'.*	201 > 1
Month:	○ January<br>○ February<br>○ March<br>○ April<br>○ May<br>○ June<br>○ July<br>○ August<br>○ September<br>○ October<br>○ November<br>○ December<br>○ N ko mo
Odun:	Year: ________________

210a. Se e loyun bayi?	${consent_obtained}<br>○ Beeni<br>○ Beeko<br>○ Mi o ti mo<br>○ ko si esi

	${pregnant} = 'yes'
210b. Oyun yin di osu melo bayi?	210a = 1
The most recent birth was: [Date of most recent birth]	${recent_birth} != ''
#####<br>*Jowo ko nomba awon osu to pari. Ko -88 fun mi ko mo, -99 fun ko le si.*	________________

209. Igba wo ni nnkan osu ti e se kehin bere?	009a = 1

Ti o ba mu ojo, ose, osu, tabi odun, o gbodo ko iye to je fun X ni iwaju	X ojo to ti koja seyin<br>X ose toti ko ja seyin<br>X osu to ti koja seyin<br>X odun to ti koja seyin<br>O ti duro omo bibi tabi o ti yo ile omo re<br>Ki o to bi abi gbeyin<br>Koi se nkan osu ri<br>ko si esi
209a. Enter [Menstrual Period]<br>*Ko 0 fun ojo to ni, kii se 0 ose tabi osu tabi odun*	(${menstrual_period} = 'days') or (${menstrual_period} = 'weeks') or (${menstrual_period} = 'month …<br>--

	${ever_birth} = 'yes' or ${pregnant} = 'yes'
213a. Ni bayi mo fe beere nipa omo yin ti e bi kehin ni abiye.	201 > 0 AND 210a ≠ 1 201 > 0 AND 210a ≠ 1 201 > 0 AND 210a ≠ 1
213b. Ni bayi mo fe beere nipa oyun ti e ni bayi.	210a = 1
Lasiko ti e fi wa noinu oyun, se e fe ni oyun nigbana, tabi e fe duro fun gba die si lojo iwaji tabi e ko fe ni oyun mo rara	(${birth_events} > 1 and ${pregnant} != 'yes') or (${ever_birth} = 'yes' and ${pregnant} = 'yes')
Lasiko ti e fi wa noinu oyun, se e fe ni oyun nigbana, tabi e fe duro fun gba die si lojo iwaji tabi e ko fe ni oyun mo rara	(${birth_events} = 1 and ${pregnant} != 'yes') or ((${ever_birth} = 'no') and (${pregnant} = 'yes'))
#####	Nigba yen<br>Ni gba miran<br>Rara<br>ko si esi

Ni bayi mo ni awon ibere nipa ojo iwaju. Se e fe lati ni (omokan/omiran) tabi e ko fe bi omo sii mo?	${consent_obtained}
211a. Se o wu yin lati bi omo tabi lati ma bi omo kankan mo?	210a ≠ 1<br>nje eti bimo<br>Ko fe Omo kankan<br>O se pe oun ko le loyun<br>ko I ti pinu tabi mi o mo<br>ko si esi
211a. Se o wu yin lati bi omo miran tabi lati ma bi omo miran mo?	210a ≠ 1<br>Se etun fe bi omo miran si<br>Ko si mo<br>O se pe oun ko le loyun<br>ko I ti pinu tabi mi o mo<br>ko si esi
211b. Leyin omo ti e n reti bayi, se o wu yin lati bi omo miran tabi lati ma bi omo omiran mo?	210a = 1<br>Se etun fe bi omo miran si<br>Ko si mo<br>O se pe oun ko le loyun<br>ko I ti pinu tabi mi o mo<br>ko si esi
212a. Bawo ni o se wu yin ki o pe to si isisinyi ki e to bi omo?<br>*If you select months or years, you will enter a number for X on the next screen.*<br>*Select "Years" if more than 36 months.*<br>*Please check that you correctly entered the value for months/years.*	211a = 1<br>X osu<br>X odun<br>Laipe tabi nisinsiyi<br>O se pe oun ko le loyun<br>Omiran<br>N ko mo<br>ko si esi
212b. Leyin bibi omo ti e n reti bayi, bawo ni yo se wu yin ki o pe to ki e to bi omo miran?<br>*If you select months or years, you will enter a number for X on the next screen.*<br>*Select "Years" if more than 36 months.*<br>*Please check that you correctly entered the value for months/years.*	211b = 1<br>X osu<br>X odun<br>Laipe tabi nisinsiyi<br>O se pe oun ko le loyun<br>Omiran

	N ko mo<br>ko si esi
212c. Ko iye igba [Months OR Years] ti e fe lati duro:	${wait_birth_none} = 'months' or ${wait_birth_some} = 'months' or ${wait_birth_pregnant} = 'mont …<br>--

Section 3 – Contraception

Bayi mma ni lati soro lori ifetosomo bibi- orisirisi ona tabi ohun ti awon loko-laya ma na lo lati le dena aimatete tabi lati ma loyun.Awon aworan kan yoo ma yoo lori ero yi ti olu dahun si ibere ba so wipe ohun ko i ti gbo nipa re tabi ko tete dsahun kaa si ibere si ta ki o si fi aworan na won, ti o ba wa ni be

301a. Nje eti gbo nipa alasopa fun obinrin ri?	009a = 1<br>Beeni<br>Beeko<br>ko si esi
301b. Nje e ti gbo nipa alasopa fun okunrin ri?<br>PROBE:Okunrin le se ise abe lati dena omo bibi..	009a = 1<br>Beeni<br>Beeko<br>ko si esi
301c. Nje e ti gbo nipa alakibapa ri?<br>PROBE: Dokita tabi Noosi le fi awon ohun ose kan sibi igunpa obinrin lati dena oyun fun odun kan tabi ju bee lo.<br>[implant_150x300.png]	009a = 1<br>Beeni<br>Beeko<br>ko si esi
301d. Nje e ti gbo nipa sowa nbe (koili) ri?<br>PROBE: Obinrin le gba pe ki Dokita tabi Noosi fi koili soju ara oun.<br>[IUD_150x300.png]	009a = 1<br>Beeni<br>Beeko<br>ko si esi
301e. Nje e ti gbo nipa alabere ri?<br>PROBE: Obinrin le gba abere ajesara lowo awon onise isegun lati dena oyun nini fun osu kan tabi ju bee lo.<br>[sayana_depo_150x300.jpg]	009a = 1<br>Beeni<br>Beeko<br>ko si esi
301f. Nje e ti gbo nipa onikoro ri?<br>PROBE: Obinrin le maa lo egboigi onihoro lojoojumo ki o fi dena oyun.<br>[pill_150x300.png]	009a = 1<br>Beeni<br>Beeko<br>ko si esi
301g. Nje e ti gbo nipa ifetosomobibi oni pajawiri?<br>PROBE: As an emergency measure after unprotected sexual intercourse women can take special pills at any time within three to five days to prevent pregnancy.	009a = 1<br>Beeni<br>Beeko<br>ko si esi
301h. Nje e ti gbo nipa roba idaabobo ti okunrin ri?<br>PROBE: Okunrin le fi roba idaabobo soju ara ki o to ba obinrin lo po.<br>[male_condom_150x300.png]	009a = 1<br>Beeni<br>Beeko<br>ko si esi
301i. Nje e ti gbo nipa roba idaabobo ti obinrin ri?<br>PROBE: Obinrin le fi roba idaabobo soju ara ki o to ba okunrin lo po.<br>[female_condom_150x300.png]	009a = 1<br>Beeni<br>Beeko<br>ko si esi
301j. Nje o ti gbọ nipa diaphragm?<br>Ibere: Obirin le gbe nkan tinrin si oju ara won ṣaaju ki o to ni ibalopo.<br>[diaphragm_150x300.png]	009a = 1<br>Beeni<br>Beeko<br>ko si esi
301k. Nje e ti gbo nipa onifomu ri?<br>PROBE: Obinrin le fi awon ohun ipara kan soju ara ki o to ba okunrin lopo.<br>[spermicide_150x300.png]	009a = 1<br>Beeni<br>Beeko<br>ko si esi
301l. Nje e ti gbo nipa ifetosomobibi onileke ri?<br>PROBE: Obinrin le lo ileke ti a ti so po lati mo awon ojo ti o le loyun, ti o ba ba okunrin lo. Ni awon ojo ti o le loyun, oun ati	009a = 1<br>Beeni<br>Beeko

alabagbepo re nilo lati lo roba idaabobo tabi ki won ma ba ara won lo.<br>[SDM-beads_only.png]	○ ko si esi
301m. Laarin osu mefa ti obinrin ti bimo, o le lo awon ilana kan nipa fifun omo loyan leralera losan ati lale, ti nnkan osu re ko si ti pada. Nje e ti gbo nipa eyi ri?	009a = 1<br>○ Beeni<br>○ Beeko<br>○ ko si esi
301n. Nje e ti gbo nipa lilo ami oju ara pelu kalenda ri?<br>PROBE: Obinrin le ma loyun bi ko ba sunmo okunrin ni awon ojo kan ninu osu ti o je wi pe o seese ki o loyun.	009a = 1<br>○ Beeni<br>○ Beeko<br>○ ko si esi
301o. Nje e ti gbo nipa dida ato sita ri?<br>PROBE: Okunrin le sera, ki o sare yora lasiko ibalopo pelu obinrin.	009a = 1<br>○ Beeni<br>○ Beeko<br>○ ko si esi
301p. Nje e ti gbo nipa awon ilana tabi ona miran ti obinrin tabi okunrin le gba lati dena oyun nini?	009a = 1<br>○ Beeni<br>○ Beeko<br>○ ko si esi
302a. Nje eyin tabi alabagbepo yin n se nkankan lowo bayi tabi e n lo awon ona kan lati lora fun nini oyun tabi dena oyun?	210a ≠ 1 AND 009a = 1 210210a ≠ 1 AND 009a = 1 210a ≠ 1 AND 009a = 1 ≠ 1 AND 009a = 1 210a ≠ 1 AND 009a = 1<br>○ Beeni<br>○ Beeko<br>○ ko si esi

	${current_user} = 'yes'
302b. Ona wo ni e nlo? Probe: nkan miran?<br>*Mu gbogbo irufe ifeto somo bibi ti o so. LO SI ISALE lati ri gbogbo awon esi.*	302a = 1<br>☐ Alasopa fun obinrin<br>☐ Alasopa fun okunrin<br>☐ Alakibapa<br>☐ Sowa nbe (koili)<br>☐ Alabere<br>☐ Ogun lilo (onihoro)<br>☐ Onihoro pajawiri<br>☐ Roba idabobo ti okunrin<br>☐ Roba idabobo ti obinrin<br>☐ Fila enu ile omo<br>☐ Foomu tabi ororo ipara<br>☐ Ki ka ojo tabi Ileke idi<br>☐ Alasepe titi (LAM)<br>☐ ona ami oju ara<br>☐ Dida ato sita<br>☐ ọna ibile miiran<br>☐ ko si esi

CALC_CM. CALCULATE: CURRENT METHOD<br>THIS WILL NOT APPEAR ON THE SCREEN<br>*ODK will identify the most effective method currently being used by the respondent by selecting the highest method in the choice list*	302a=1 AND 302b ≠-99<br>○ Alasopa fun obinrin<br>○ Alasopa fun okunrin<br>○ Alakibapa<br>○ Sowa nbe (koili)<br>○ Alabere<br>○ Ogun lilo (onihoro)<br>○ Onihoro pajawiri<br>○ Roba idabobo ti okunrin<br>○ Roba idabobo ti obinrin<br>○ Fila enu ile omo<br>○ Foomu tabi ororo ipara<br>○ Ki ka ojo tabi Ileke idi<br>○ Alasepe titi (LAM)<br>○ ona ami oju ara<br>○ Dida ato sita<br>○ ọna ibile miiran

	ko si esi
LCL_301. Ibere: a awọn abẹrẹ ti a nṣakoso nipasẹ syringe tabi kekere abẹrẹ?<br>*Fi aworan han Oludahun*<br>[sayana_depo_150x300.jpg]	CALC CM = 5<br>Sirinji<br>abeere kekere (Sayana Press)<br>ko si esi
302c. Nje oko yin tabi enikeji yin mo wipe e n lo lowolowo [CURRENT METHOD]?	302a = 1<br>Beeni<br>Beeko<br>ko si esi
302c. Nje oko yin tabi enikeji yin mo wipe e n lo ohun ifetosomo bibi?	302a = -99<br>Beeni<br>Beeko<br>ko si esi
303. N je osise ile itoju fi ye yin tabi alabagbepo yin pe e ko ni bimo mo lehin ise abe naa?	302b = male or female sterilization<br>Beeni<br>Beeko<br>ko si esi
305a. E so wipe e ko lo ilana ifeto-somo bibi lowolowo. Nje e lero pe e o lo ilana ifeto-somo bibi kankan lati da oyun ninin duro tabi dena oyun nini nigbakugba ni ojo iwaju?	302a ≠1 AND 210a ≠1<br>Beeni<br>Beeko<br>ko si esi
305b. Nje e lero pe e o lo ilana ifeto-somo bibi kankan lati lora fun iloyun tabi dena oyun nini lojo iwaju?	302a ≠1 AND 201a = 1<br>Beeni<br>Beeko<br>ko si esi
306a. Laarin osu mejila seyin, nje e ti se ohun kan tabi lo ona kan lati lora fun iloyun tabi dena oyun nini?	302a ≠1<br>Beeni<br>Beeko<br>ko si esi

	${recent_user} = 'yes'
306b. Ilana wo ni e lo gbeyin? Probe: Ewo tun ni?<br>*Mu eyi irufe ifeto somo bibi ti o si se ju lo (eyi ti o po ju ninu awon ti o so). Lo si isale lati ri gbogbo awon esi.*	306a = 1<br>Alakibapa<br>Sowa nbe (koili)<br>Alabere<br>Ogun lilo (onihoro)<br>Onihoro pajawiri<br>Roba idabobo ti okunrin<br>Roba idabobo ti obinrin<br>Fila enu ile omo<br>Foomu tabi ororo ipara<br>Ki ka ojo tabi Ileke idi<br>Alasepe titi (LAM)<br>ona ami oju ara<br>Dida ato sita<br>ọna ibile miiran<br>ko si esi

LCL_302. Ibere: a awọn abẹrẹ ti a nṣakoso nipasẹ syringe tabi kekere abẹrẹ?<br>*Fi aworan han Oludahun*<br>[sayana_depo_150x300.jpg]	306b = 5<br>Sirinji<br>abeere kekere (Sayana Press)<br>ko si esi
307. Ki e to bere si ni lo [CURRENT METHOD / MOST RECENT METHOD], n je e ti ba oko tabi enikeji yin so ipinu lori fifi aye die le tabi lati ma loyun?	302a = 1 OR 306a = 1<br>Beeni<br>Beeko<br>N ko mo<br>ko si esi
308. Nje e so fun mi pe lilo ohun ifetosomo bibi je ipinu yin nikan, tabi ipinnu oko/enikeji yin nikan tabi eyin mejeji jo pinnu lapapo?	302a = 1<br>o kun oludahun<br>O kun ọkọ / alabaṣepọ<br>ipinnu lapapo

	○ Omiran<br>○ ko si esi
TCI_302. Yato si o ati ọkọ rẹ / alabaṣepọ, ti o miran ipa awọn ipinnu lati lo kan ebi igbogun ọna?<br>*Ma se ka awon asayan yii soke, mu gbogbo eyi to o ba ye*	302a = 1 OR 306a = 1<br>☐ Iya<br>☐ Iya iyawo<br>☐ Arabirin<br>☐ Egbon iyawo re<br>☐ Iya agba obinrin<br>☐ Oree<br>☐ Osise ile iwosan<br>☐ Olori awujo<br>☐ Olori Esin<br>☐ Egbon obirin<br>☐ Ebi miran<br>☐ Omiran<br>☐ Kosi eniyan kankan<br>☐ ko si esi
TCI_302x. Ni awọn kẹhin 12 osu, ti o niyanju eyikeyi ebi igbogun ọna lati ọrẹ rẹ ati / tabi awọn ibatan?	302a = 1 OR 306a = 1<br>○ Beeni<br>○ Beeko<br>○ N ko mo<br>○ ko si esi
308a. Nigba ti e gba eyi ti o keyin [CURRENT METHOD /MOST RECENT METHOD],elo ni e san lapo arayin, pelu iye ti e san fun irufe eyi, ti e gba tabi anfaani, ati ti moto ?<br>*Enter all prices in Naira. Zero is a possible answer. Enter -88 if respondent does not know, -99 for no response.*	302a = 1 OR 306a = 1<br>________________

	${current_user} = 'yes'
309a. Nigba ti e gba eyi to kehin ${current_recent_label}, elo ni e san lapo yin, pelu owo ti e san fun ohun idabobo, tie gba tabi anfaani, ati moto?<br>*Se akaseyin lati ojo ma le gbagbe kan ti o sele ti o ba pon dandan*	302a = 1
Most Recent Birth: [mm-yyyy]	${recent_birth} != ''
Current Marriage: [mm-yyyy]	${husband_cohabit_start_recent} != ''
Month:	○ January<br>○ February<br>○ March<br>○ April<br>○ May<br>○ June<br>○ July<br>○ August<br>○ September<br>○ October<br>○ November<br>○ December<br>○ N ko mo
Odun:	Year: ________________

	${recent_user} = 'yes'
309b. Ni gba wo le dawo duro lilo[CURRENT METHOD / MOST RECENT METHOD]?<br>*Please record the date. The date should be found by calculating backwards from memorable events if needed.*<br>*Select 'Do not know' for month and '2020' for year to indicate No Response.*	306a = 1
Month:	○ January<br>○ February<br>○ March<br>○ April<br>○ May<br>○ June

	July<br>August<br>September<br>October<br>November<br>December<br>N ko mo
Odun:	Year: ________________

	${recent_user} = 'yes'
309c. Ni osu ati odun wo leti bere si ni lo [CURRENT METHOD / MOST RECENT METHOD] ki e to dawo lilo duro?<br>*Calculate backwards from memorable events if needed.*<br>*Select 'Do not know' for month and '2020' for year to indicate No Response.*	306a = 1
Most Recent Birth: [mm-yyyy]	${recent_birth} != ''
Current Marriage: [mm-yyyy]	${husband_cohabit_start_recent} != ''
Month:	January<br>February<br>March<br>April<br>May<br>June<br>July<br>August<br>September<br>October<br>November<br>December<br>N ko mo
Odun:	Year: ________________

309d. AYEWO: Ki o le je idani loju, e lo [CURRENT METHOD / MOST RECENT METHOD] leralera laarin [START DATE] ati [END DATE] laisi daduro, see be ni?	306a = 1<br>Beeni<br>Beeko
PADA SI OJU EWE TI O KOJA, KI O SI BEERE IGBA TI O LO KEYIN<br>*Ibeere: - Igba wo ni O lo ona yii keyin [METHOD]? -Lati Igba wo ni o ti un lo [METHOD] lai dekun*	309d = 0
310. Ki lo fa ti e ko fi lo mo [CURRENT METHOD / MOST RECENT METHOD]?	306a = 1<br>Mi ki n ni ibalopo nigba kugba tabi oko mi ko ti lo irin ajo<br>Oloyun ni gba ti olo<br>o fe loyun<br>Oko tabi enikeji ko fowo si<br>O n wa ona ifeto somo bibi to sise gidigan<br>Irufe ifeto somo bibi kan ko si<br>Ilera to kan gbongbon<br>Eru ewu to ro mo<br>Ko ni anfaani tabi o ti jina ju<br>o ti won ju<br>ko se e lo<br>Ewu tabi ijamba<br>O soro lati loyun tabi ki dawo omo bibi duro<br>ko ba mi lara mu<br>Omiran<br>N ko mo<br>ko si esi

	${current_or_recent_user} and (${current_recent_method} != 'LAM') and

311a. E koko bere si ni lo [CURRENT METHOD / MOST RECENT METHOD] ni [DATE FROM FQ309a OR 309c] Ni bo ni eyin tabi enikeji yin ti gba ti gbe yen?<br>*Lo si isale lati ri gbogbo esi*	(${current_recent_method} !=<br>(CALC_CM ≠ 14, 30, 31, 39, -99) OR (306b ≠ 14, 30, 31, 39, -99)<br>○ Ile Iwosan Ijoba nla<br>○ Ile iwosan Ijoba alabode<br>○ Ile iwosan ifeto somo bibi<br>○ Ile iwosan agbe kiri (Titi Ijoba)<br>○ TBA/Fieldworker (public)<br>○ Ile iwosan Aladani<br>○ Ile Ise ita ogun<br>○ Ile Ita oogun/Chemisiti<br>○ Dokita tabi noosi Aladani<br>○ Ile iwosan agbe kiri (Ti Aladani)<br>○ TBA/Fieldworker (private)<br>○ Shobu<br>○ Ile ise awon onigbagbo/church<br>○ Oree/alabagbe po<br>○ NGO<br>○ Oja / olukiri oja<br>○ Omiran<br>○ N ko mo<br>○ Ko si esi
312a. Ni gba ti e gba [CURRENT METHOD / MOST RECENT METHOD], nje olupese so awon alebu tabi ohun ti o le sele si irufe ohun ifetosomo bibi lati le fi aaye tabi lati le se idiwo fun oyun nini?	311a ≠ .<br>○ Beeni<br>○ Beeko<br>○ ko si esi
312b. Nje won so ohun ti e le se bi e ba sakiyesi ipalara ati isoro tio le jeyo latara lilo ilana yi?	312a = 1<br>○ Beeni<br>○ Beeko<br>○ ko si esi
313. Ni gba na, nje olupse ohun ifetosomobibi lori awon orisirisi irufe ohun ifetosomo bibi leyin eyi [CURRENT METHOD / MOST RECENT METHOD] ti e le lo?	311a ≠ . OR 311b ≠ .<br>○ Beeni<br>○ Beeko<br>○ N ko mo<br>○ ko si esi
314a. Lasiko ibewo naa, nje e gba ilana eto ti e fe gba lati lora fun iloyun tabi dena oyun nini?	311a ≠ .<br>○ Beeni<br>○ Beeko<br>○ ko si esi
314c. Kini idi ti e ko fi ri eyi ti e fe?	314a = 0<br>○ irufe ohun ifeto somo bibi ti tan ni ojo yen<br>○ Ko si irufe ohun ifeto somo bibi<br>○ Olupese ko gba eko lori bi a se n funni ni irufe ifeto somo bibi<br>○ Olupese yan irufe miran<br>○ Ko yẹ fun ọna<br>○ opinu lati ma lo eto ifeto somo bibi<br>○ o ti won ju<br>○ Omiran<br>○ ko si esi
315a. Lasiko ibewo naa, tani o se pinu lori irufe ilana ifetosomobibi ti e gba?	311a ≠ .<br>○ Iwo nikan<br>○ Olupese<br>○ Eni<br>○ Iwo ati Olupese<br>○ Iwo ati Alabagbepo<br>○ Omiran<br>○ N ko mo<br>○ ko si esi
315b. Ta lo se ipinu nipa lilo ami oju ara ati kalenda	311b ≠ .

	○ Iwo nikan<br>○ Olupese<br>○ Eni<br>○ Iwo ati Olupese<br>○ Iwo ati Alabagbepo<br>○ Omiran<br>○ N ko mo<br>○ ko si esi
315b. Ta lo se ipinu nipa lilo LAM	311b ≠ .<br>○ Iwo nikan<br>○ Olupese<br>○ Eni<br>○ Iwo ati Olupese<br>○ Iwo ati Alabagbepo<br>○ Omiran<br>○ N ko mo<br>○ ko si esi
316. Nje e o pada si odo olupese na? Olupese: [Type of provider selected in 311a or 311b]	311a ≠ 35 or 96<br>○ Beeni<br>○ Beeko<br>○ N ko mo<br>○ ko si esi
317. Se e le je ki ebiyin tabi ore yin los is odo olupese yi? Olupese: [Type of provider selected in 311a or 311b]	311 a ≠ 34 or 96<br>○ Beeni<br>○ Beeko<br>○ N ko mo<br>○ ko si esi
SW_1a. SW_1a.Ki o to di pe ebere si lo [CURRENT METHOD / MOST RECENT METHOD] ni [MOIS/ANNEE], nje e lo ona kankan lati de na oyun?	302a = 1 OR 306a = 1<br>○ Beeni<br>○ Beeko<br>○ ko si esi
SW_1b. Eto woni en lo?	SW_1a = 1<br>○ Alakibapa<br>○ Sowa nbe (koili)<br>○ Alabere<br>○ Ogun lilo (onihoro)<br>○ Onihoro pajawiri<br>○ Roba idabobo ti okunrin<br>○ Roba idabobo ti obinrin<br>○ Fila enu ile omo<br>○ Foomu tabi ororo ipara<br>○ Ki ka ojo tabi Ileke idi<br>○ Alasepe titi (LAM)<br>○ ona ami oju ara<br>○ Dida ato sita<br>○ ọna ibile miiran<br>○ ko si esi
PP_1. PP_1.Lati igba ti e ti bi omo yin ni [DATE OF MOST RECENT BIRTH], nje e lo nkan kan lati dena oyun nini?	child born in last 2 years AND 302a ≠ 1<br>○ Beeni<br>○ Beeko<br>○ ko si esi
PP_2. O to igba wo leyin ibimo ni [DATE OF MOST RECENT BIRTH] le bere si lo nkan?<br>*Enter 0 days for today. You will enter a number for X on the next screen.*	PP_1 = 1 OR (302a = 1 AND child born in the last 2 years)<br>○ Lẹhin ọjọ X<br>○ Lẹhin awọn ọsẹ X<br>○ Lẹhin Oṣooṣu X<br>○ Lẹhin ọdun X<br>○ ko si esi
PP_2. Enter [METHOD].<br>*If today, enter zero days only, not zero weeks/months/years.*	${pp_method_units} = 'days' or ${pp_method_units} = 'weeks' or ${pp_method_units} = 'months' or ${ …

PP_3. Iru eto wo ni e lo?	--<br>○ Alasopa fun obinrin<br>○ Alasopa fun okunrin<br>○ Alakibapa<br>○ Sowa nbe (koili)<br>○ Alabere<br>○ Ogun lilo (onihoro)<br>○ Onihoro pajawiri<br>○ Roba idabobo ti okunrin<br>○ Roba idabobo ti obinrin<br>○ Fila enu ile omo<br>○ Foomu tabi ororo ipara<br>○ Ki ka ojo tabi Ileke idi<br>○ Alasepe titi (LAM)<br>○ ona ami oju ara<br>○ Dida ato sita<br>○ ọna ibile miiran<br>○ ko si esi
LCL_PP. PROBE: Je abere ti won fun yi nla ni tabi kekere?<br>*Fi aworan han Oludahun*<br>[sayana_depo_150x300.jpg]	PP_3 = 5<br>○ Sirinji<br>○ abeere kekere (Sayana Press)<br>○ ko si esi
319. Nje e ti se nkan tabi gbiyanju lona kan tabi omiran lati dena oyun tabi lora lati loyun?	306a ≠ 1 OR 302a ≠ 1<br>○ Beeni<br>○ Beeko<br>○ ko si esi
320. E to omo dun melo ni gba ti e koko lo ohun lati le se idaduro tabi lati le ma loyun? Oludahun si beere so wipe oun je omo odun [AGE] nigba ti se ojo ibi keyin.<br>*Te ojo ori in Odun Te-88 ti oludahun ko ba mo. Te -99 ti ko ba si idahun. Ko le kere ju omo odun mesan.*	302a = 1 OR 306a =1 OR 319 = 1<br>--
321. Omo me lo to wa laye leni nigba na, ti o ba wa? Kiyesi: oludahun si beere so pe oun bi iye omo [NUMBER OF LIFE BIRTHS] ni 201.<br>*Ko -99 fun ko le si.*	Age in 320 ≥ 9 AND 200 = 1<br>--

	${fp_ever_used} = 'yes'
322. Ona tabi ara wo ni e lo fun igba akoko lati lora fun iloyun tabi dena oyun nini?<br>*Ma se ka gbo ori ona ifeto somo bibi. Lo si isale lati ri pe gbogbo esi lo ri*	319 = 1<br>○ Alasopa fun obinrin<br>○ Alasopa fun okunrin<br>○ Alakibapa<br>○ Sowa nbe (koili)<br>○ Alabere<br>○ Ogun lilo (onihoro)<br>○ Onihoro pajawiri<br>○ Roba idabobo ti okunrin<br>○ Roba idabobo ti obinrin<br>○ Fila enu ile omo<br>○ Foomu tabi ororo ipara<br>○ Ki ka ojo tabi Ileke idi<br>○ Alasepe titi (LAM)<br>○ ona ami oju ara<br>○ Dida ato sita<br>○ ọna ibile miiran<br>○ ko si esi

LCL_322a. Ibere: a awọn abẹrẹ ti a nṣakoso nipasẹ syringe tabi kekere abẹrẹ?<br>*Fi aworan han Oludahun*<br>[sayana_depo_150x300.jpg]	322 = 5<br>○ Sirinji<br>○ abeere kekere (Sayana Press)<br>○ ko si esi
322a. Nje e ti lo ogun onihoho pajawiri ni gba kugba laarin osu mejila to ko ja?	302b ≠ emergency contraception OR 306b ≠ 8<br>○ Beeni

BERE SIWAJU SI: Fun ohun pajawiri ti obinrin le lo nigba ti o ba sese ni ibalopo lai lo ohun odabobo ti ogun oni kooro laarin ojo meta si marun lati le dena oyun nini.	○ Beeko<br>○ ko si esi

	((${current_user} = 'no')) and (((${more_children_none} = 'no_children') or …
323a. E so wipe eko fe bi omo ni kiakia ati wipe eko lo irufe idabo ti ole se idiwo fun oyun nini	302a = 0 AND ((212a or 212b > 2 years) OR (211a or 211b = 2))
323a. E so wipe e ko fe bi omo miran ni kiakia ati wipe e ko lo irufe idabobo ti o le se idena fun oyun nini	302a = 0 AND ((212a or 212b > 2 years) OR (211a or 211b = 2))
323a. E so wipe e ko fe bi omokomo ati wipe e ko lo irufe idabobo ti o le se idena fun oyun nini	302a = 0 AND ((212a or 212b > 2 years) OR (211a or 211b = 2))
323a. E so wipe e ko fe bi omo mo ati wipe e ko lo irufe idabobo ti o le se idena fun oyun nini	302a = 0 AND ((212a or 212b > 2 years) OR (211a or 211b = 2))
Nje e le so fun mi idi ti e ko fi n lo irufe idabobo lati se idiwo fun oyun nini?<br>Beere: Fun idi miran?<br>*RECORD ALL REASONS MENTIONED*<br>*Cannot select "Not Married" if 104 is "Yes, currently married".*<br>*Scroll to bottom to see all choices.*	☐ ko si ni ile oko<br>☐ Mi ki n ni ibalopo nigba kugba tabi mi ko ni ibalopo<br>☐ O ti duro omo bibi tabi o ti yo ile omo re<br>☐ Koi ti bi mo tabi ko le bimo<br>☐ ko se nkan osu lehin ibi to keyin<br>☐ Oyan mimu<br>☐ oko ko si ni ile fun ojo pupo<br>☐ Owo Olorun tabi Ewu<br>☐ Oludahun ibeere tako<br>☐ Oko tabi enikeji taa ko<br>☐ awọn miran lodi<br>☐ Esin ko fa ye gba<br>☐ Ko mo irufe ifeto somo bibi kankan<br>☐ Ko mo ibi ti o ti le gba ifeto somo bibi<br>☐ Eru ewu to ro mo<br>☐ Ilera to kan gbongbon<br>☐ Ko ni anfaani tabi o ti jina ju<br>☐ o ti won ju<br>☐ Ko si irufe ifeto somo bibi to fe<br>☐ Irufe ifeto somo bibi kan ko si<br>☐ ko se e lo<br>☐ ko ba mi lara mu<br>☐ Omiran<br>☐ N ko mo<br>☐ ko si esi

323b. Nje e le so fun mi pe ipinnu yin nikan lati ma lo ohun ifetosomo bibi, ipinnu oko yin/enikeji yin nikan ni tabi ipinnu eyin mejeji ni?	302a ≠ 1<br>○ o kun oludahun<br>○ O kun ọkọ / alabaṣepọ<br>○ ipinnu lapapo<br>○ Omiran<br>○ ko si esi
TCI_304. Yato si o ati ọkọ rẹ / alabaṣepọ, ti o miran ipa awọn ipinnu ko lati lo a ebi igbogun ọna?<br>*Ma se ka awon asayan yii soke, mu gbogbo eyi to o ba ye*	306a = 0<br>☐ Iya<br>☐ Iya iyawo<br>☐ Arabirin<br>☐ Egbon iyawo re<br>☐ Iya agba obinrin<br>☐ Oree<br>☐ Osise ile iwosan<br>☐ Olori awujo<br>☐ Olori Esin<br>☐ Egbon obirin<br>☐ Ebi miran<br>☐ Omiran

	☐ Kosi eniyan kankan<br>☐ ko si esi
TCI_304x. Ni awọn kẹhin 12 osu, ni o ni ore kan ati / tabi awọn ojulumo niyanju pe ki o lo a ebi igbogun ọna?	306a = 0<br>○ Beeni<br>○ Beeko<br>○ ko si esi
324. Laarin osu mejila to koja, nje awon eleto ilera abele bayin soro lori ifetosomo bibi?	009a = 1<br>○ Beeni<br>○ Beeko<br>○ ko si esi
325a. Laarin osu mejila seyin, n je e ti lo si ile iwosan tabi koyin si bikan fun itoju fun rara yin?<br>*Fun eyi keyi anfaani ilera*	009a = 1<br>○ Beeni<br>○ Beeko<br>○ ko si esi
325a. Laarin osu mejila sehin, n je e ti lo sile eto ilera lati gba itoju ara yin tabi awon omo yin?<br>*Fun eyi keyi anfaani ilera*	009a = 1<br>○ Beeni<br>○ Beeko<br>○ ko si esi
325b. Nje ikankan lara awon osise ile ise eto ilera ba yin soro nipa eto ifeto somo bibi?	325a = 1<br>○ Beeni<br>○ Beeko<br>○ ko si esi
TCI_305. Ti o lọ a awujo iṣẹlẹ ni odun to koja ibi ti ebi igbogun ti a laisi idanilowoko sísọ?	009a = 1<br>○ Beeni<br>○ Beeko<br>○ ko si esi
TCI_306. Ṣe o ro nibẹ ni o wa diẹ ninu awọn eniyan laarin yi awujo ti o yoo pe o buburu awọn orukọ tabi yago fun ile-iṣẹ rẹ ti o ba ti nwọn si mọ pe o ni won nipa lilo a ebi igbogun ọna?	009a = 1<br>○ Beeni<br>○ Beeko<br>○ N ko mo<br>○ ko si esi
TCI_307. Ṣe o ro nibẹ ni o wa diẹ ninu awọn eniyan laarin yi awujo ti o yoo iyìn, se iwuri fun, tabi soro laisi idanilowoko nipa ti o ba ti nwọn mọ ti o ni won nipa lilo a ebi igbogun ọna	009a = 1<br>○ Beeni<br>○ Beeko<br>○ N ko mo<br>○ ko si esi
TCI_308. Ninu awọn ti o ti kọja 12 osu, ti o ti gbọ eyikeyi ninu awọn wọnyi eniyan soro ni gbangba ni ojurere ti ebi igbogun?<br>*Ka gbogbo esi sita ki o si mu awon esi to ye*	009a = 1<br>☐ Oṣiṣẹ ijọba (ipele ti orilẹ-ede)<br>☐ Ipinle, Ijoba Ibile, or Olori Agbebe<br>☐ Olori Esin<br>☐ ko si esi ninu gbogbo awon esi<br>☐ ko si esi
TCI_309. Ninu awọn ti o ti kọja 12 osu, ti o ti gbọ eyikeyi ninu awọn wọnyi eniyan soro ni gbangba lodi ebi igbogun?<br>*Ka gbogbo esi sita ki o si mu awon esi to ye*	009a = 1<br>☐ Oṣiṣẹ ijọba (ipele ti orilẹ-ede)<br>☐ Ipinle, Ijoba Ibile, or Olori Agbebe<br>☐ Olori Esin<br>☐ ko si esi ninu gbogbo awon esi<br>☐ ko si esi
TCI_309x. Bawo ni ọpọlọpọ awọn ti rẹ sunmọ awọn ọrẹ ati ebi ṣe o ro lilo ebi igbogun: kò si, diẹ ninu awọn, julọ, tabi gbogbo?	009a = 1<br>○ Ko si<br>○ Die ninu won<br>○ Julo<br>○ Gbogbo ree<br>○ N ko mo<br>○ ko si esi

	009a = 1		
326. Laarin osu melo sehin, n je e ti:			
	Beeni	Beeko	ko si esi
326a. Gbo nipa ifeto-somo bibi lorii ero asoromagbesi?	○	○	○
326b. Se ri nkankan wo lori feto somo bibi lori ero	○	○	○

amowumaorna/telifisan? 326c. Se e ka ni pa ifeto somo bibi ninu iwe iroyin/tabi ti igbadegba?	○	○	○
326d. Se e gba atejise abi ipe lori ero alagbeka lori eto ifetosomobibi	○	○	○
TCI_310. Ka nipa ebi igbogun ni a panfuleti, leaflet, tabi flyer?	○	○	○
TCI_311. Ri a panini tabi Patako iwe pẹlu kan ebi igbogun ifiranṣẹ?	○	○	○

Section 4 – Sexual Activity

CHECK FOR THE PRESENCE OF OTHERS. BEFORE CONTINUING, MAKE EVERY EFFORT TO ENSURE PRIVACY.

Bayi ma ni lati bere awon ibere ran pe lori ibalopo lati le je ki a ni imo lori awon oro ti o se pataki si aye eda. E je kin fi dayin loju pe awon esi ko ni han si eda kankan. Ti a ba de irufe ibere ti e ko ba fe dahun, eko je ki n mo a ko fo ibere na si omiran	${consent_obtained}

	${consent_obtained}
401a. Omo odun melo ni yin nigba ti e koko sunmo okunrin?	309a = 1
Current age: [AGE]	
Iye omo to wa laye: [NUMBER OF LIFE BIRTHS]	${birth_events} > 0
Oludahun si ibeere ti loyun	${pregnant} = 'yes'
Ko ojo ori ni odun.<br>*Enter -77 if she has never had sex. Enter -99 for no response. Enter -88 for do not know.*	--

	((${age_at_first_sex} >= 0) or (${age_at_first_sex} =-88) or (${age_at_first_sex} =-99))
402. Igba wo ni e sunmo okunrin kehin?	401a ≠ -77

402. Enter [# days / weeks / months / years].<br>*Ti o ba je oni, te oodo ojo nikan, kii se odo ose/osu/odun*<br>*O gbodo ba ojo ori nigba ibalopo akoko mu ati ipo iloyun*	401a ≠ -77<br>--
LCL_403. Ni iwoye bi ara yin se ri, ti e ba ni ibalopo leekan lai lo idabo lati dena oyun nini, nje ero pe ele loyun?<br>*Ka awọn aṣayan idahun ni kiakia.*	${consent_obtained}<br>○ O seese<br>○ O seese di e<br>○ O seese ati ko tun seese<br>○ Ko seese<br>○ N ko mo<br>○ Ko si esi
LCL_404. Ni iwoye bi ara yin se ri, ti e ba ni ibalopo lera lera, bi eemeji laarin ose, fun odun kan gbako lai lo irufe idabo lati dena oyun ni, nje ero pe ele loyun bi?<br>*Ka awọn aṣayan idahun ni kiakia.*	${consent_obtained}<br>○ O seese<br>○ O seese di e<br>○ O seese ati ko tun seese<br>○ Ko seese<br>○ N ko mo<br>○ Ko si esi

Section 6 – Menstrual Hygiene

Bayi mo ma bere awon ibere kan lori bi e se n se imoto ara yin ni gba ti e ba n se nkan osu. Eyi wa ni ba mu pelu awon ohun ilo bi; anfaani si arayin nikan, mimo, ibi ti ko le wu; washing as required; and a place to dispose used materials.

602a. Ni bo gan ni e ti ma n paro paadi, aso, tabi ohun itoju abe obinrin miran?	(209 ≤ 90 days, 13 weeks or ≤ 3 months)<br>○ Shalanga olomi<br>○ shalanga alategbun<br>○ shalanaga o ni ideri<br>○ Shalanga ti ko ni ideri<br>○ shalanga o ni ke<br>○ Ile igbe ala bo mole<br>○ Ile iyagbe a la gbe ko

	○ Yara Orun<br>○ eyin ile<br>○ Ko si ibi / igbo / aaye<br>○ Omiran<br>○ ko si esi
Place: [MAIN PLACE FROM 602a] 602b. While managing your menstrual hygiene, was this place:<br>*Read each option aloud and select if yes.*	601 ≠ -99 nor null AND 602 ≠ -99 nor null<br>☐ Mimo?<br>☐ Aladani?<br>☐ O pa mo?<br>☐ O se tii?<br>☐ Omi wa nibe?<br>☐ Ose wa nibe?<br>☐ ko si esi ninu gbogbo awon esi<br>☐ ko si esi
603. Nigba ti e se nkan osu yin keyin, kini e lo lati gba tabi se idaduro eje nkan osu?<br>BERE: Se nkan mi tunku?<br>*Ma se ka awon asayan yii soke, mu gbogbo eyi to o ba ye*	(209 ≤ 90 days, 13 weeks or ≤ 3 months)<br>☐ ajudanu<br>☐ atunlo<br>☐ Aso titun<br>☐ Aso ogbo<br>☐ owu<br>☐ aso iledi<br>☐ owu<br>☐ iwe toileti<br>☐ Awotele nikan<br>☐ Bucketi<br>☐ Omiran<br>☐ Ko si ohun elo ti a lo<br>☐ ko si esi
604a. Se efo etun wa tun padi, aso ati ohun miran ti awon obinrin fi n se imototo oju ara lo nigba ti e n se nkan osu yin to ko ja?	603 = reusable sanitary pad, old cloth, underwear alone, or bucket<br>○ Beeni<br>○ Beeko<br>○ ko si esi
604b. Nigba ti e n se nkan osu yin to ko ja, se awon ohun ti a fi n se imototo oju ara obinrin ti e fo ki o to tun lo gbe tan ki e to tun won lo?	604a = 1<br>○ Beeni<br>○ Beeko<br>○ ko si esi
605. E so wipe e lo [ODK will display the responses from 603] nigba ti e se nkan osu yin to ko ja. Ni bo ni e da awon ohun ilo naa si nigba ti e lo won tan? BERE: Ki ni ohun to tun ku?<br>*Ma se ka awon asayan yii soke, mu gbogbo eyi to o ba ye*	603 = Padi ti a o ju nu leyin lilo, aso tuntun, owu, iledi omode, pepa fele, or other OR 604a =0<br>☐ ile igbe oyibo<br>☐ ile igbe<br>☐ apo idale nu<br>☐ Ji jona<br>☐ Igbo/ori papa<br>☐ Omiran<br>☐ ko si esi
606a. Leyin ise ile yin, nje e ti se ise kankan laarin osu to ko ja?	(209 ≤ 90 days, 13 weeks or ≤ 3 months)<br>○ Beeni<br>○ Beeko<br>○ ko si esi
606b. Nitori ikan osu ti e se kehin, nje o ni ojo ise kan kan ninu osu to ko ja ti e le lo?	606a = 1<br>○ Beeni<br>○ Beeko<br>○ ko si esi
607a. Nje e lo si ile iwe nigba kankan laarin osu mejila to ko ja?	(209 ≤ 90 days, 13 weeks or ≤ 3 months)<br>○ Beeni<br>○ Beeko<br>○ ko si esi
607b. Nitori asiko akoko rẹ, awọn ile-iwe ni o wa ni awọn oṣu mejila ti o ti kọja pe iwọ ko lọ si?	607a = 1<br>○ Beeni<br>○ Beeko

	○ ko si esi

Section 7.1 – Ore Alasiri

701. Bayi mo fe bere awon ibeere to da lori ore alasiri to sun mo yin gidigan, obinrin ti e ma ba so oro asiri ti oun na ma n so asiri ti re pelu yin. Bi melo ore bayi leni ni ile Nigeria ti ojo ori won koju 15 and 49? *Enter -88 for do not know, -99 for no response.*	${consent_obtained} --
702a. Ejowo eko woo orebinrin tosun mo yin ni ile Nigeria ti ojo ori re wa laarin 15 and 49 ti o je wipe e ma so awon oroyin ti oun na maa n so awon oro ara re pelu yin. Lati le je ka mo irufe ore na, ejowo se ele fun wa ni oruko inagije re tabi oruko ti e ma pe ti ki se oruko abiso re?	${friend_count} > 0 --
703a. Omo odun melo ni ${friend1_name} nigba ti o se ojo ibire keyin? *Enter -88 for do not know, -99 for no response.*	${friend_count} > 0 --
704a. Kini ipele eko to ga ju ti ${friend1_name} lori?	${friend_count} > 0<br>○ Ko lo rii<br>○ Ile-iwe Alakobere<br>○ Ile-iwe sekondiri<br>○ Iwe giga<br>○ ko si esi
702b. Ejowo eko woo orebinrin keji tosun mo yin ni ile Nigeria ti ojo ori re wa laarin 15 and 49. Lekan si, ogbodo je obinrin ti o je wipe e ma n so awon oroyin ti oun na maa n so awon oro ara re fun yin. Lati le je ka mo irufe ore na, ejowo se e le fun wa ni oruko inagije re tabi oruko ti e ma n pe ti ki se oruko abiso re?	${friend_count} > 1 --
703b. Omo odun melo ni ${friend2_name} nigba ti o se ojo ibire keyin? *Enter -88 for do not know, -99 for no response.*	${friend_count} > 1 --
704b. Kini ipele eko to ga ju ti ${friend2_name} lo?	${friend_count} > 1<br>○ Ko lo rii<br>○ Ile-iwe Alakobere<br>○ Ile-iwe sekondiri<br>○ Iwe giga<br>○ ko si esi

Section 7.2 – Oyun sise

CHECK FOR THE PRESENCE OF OTHERS. BEFORE CONTINUING, MAKE EVERY EFFORT TO ENSURE PRIVACY.

Awon ibeere to kan da lori awon ohun ti obinrin agbegbe yi n lo lati yoo oyun. Eyi je ohun iriri lori le ede Nigeria ati wipe a fe mo si waju si awon ohun ti obinrin n lo lati yo oyun. Mo fe ran yin leti wipe awon iwadi wa yoo je ohun odo bade ko tun ri ta mo ati wipe yoo wa laarin wa, ako si ni je ki enikeni mo awon esi ti e fun wa. Ti a ba de awon ibeere ti e ko fe dahun, ejowo e so fun mi ki n le tesiwaju si ibeere to tele. *Press OK to continue.*	${consent_obtained}<br>☐ odaa
705. Nigba miran awon obinrin nko aya soke pe awon ti loyun tabi loyun nigba ti ko too ati wipe won maa n se nkan lati yoo irufe oyun na. Ba wo ni eyi ri ni agbegbe ti e n gbe? *Read response options aloud.*	${consent_obtained}<br>○ O won poo<br>○ o won poo di e<br>○ Ko won poo gan<br>○ Ko won poo rara<br>○ N ko mo<br>○ Ko si esi
706. Ni agbegbe ti e n gbe bayii, iru ona wo ni awon obinrin ti won loyun tabi ko aya soke nitori oyun nini n gba lati yoo oyun tabi ki won se oyun? Ona miran wo lo tun wa? *Do not read options aloud. Select all that apply.*	${consent_obtained}<br>☐ Abee sisse<br>☐ Ogun oni koro ti oruko re n je mifepristone or misoprostol bi apere Mariprist, Mifepak, Cytotec, Miso-Fem, or Misoclear

☐ Ogun oni koro te maa n lo nigba ti e ba ni iba bii egbogi tabi ogun iba
☐ Ogun Onikooro Pajawiri
☐ Ogun koro miran
☐ Abeere
☐ Egbogi tabi awon ona ibile, bi agbo
☐ Ogogoro
☐ Iyo, eeru, magi, kahun
☐ Osan wewe
☐ Ogun iko olomi
☐ Fi fi nkan si inu oju ara obinrin
☐ Omiran
☐ N ko mo
☐ Ko si esi

---

707. Ewo ninu awon ona wonyi ni won ma n SABA lo?

count-selected(${abt_ways}) > 1

○ Abee sisse
○ Ogun oni koro ti oruko re n je mifepristone or misoprostol bi apere Mariprist, Mifepak, Cytotec, Miso-Fem, or Misoclear
○ Ogun oni koro te maa n lo nigba ti e ba ni iba bii egbogi tabi ogun iba
○ Ogun Onikooro Pajawiri
○ Ogun koro miran
○ Abeere
○ Egbogi tabi awon ona ibile, bi agbo
○ Ogogoro
○ Iyo, eeru, magi, kahun
○ Osan wewe
○ Ogun iko olomi
○ Fi fi nkan si inu oju ara obinrin
○ Omiran
○ N ko mo
○ Ko si esi

selected(${abt_ways}, filter) or (filter = 'always')

---

708. Nibo ni awon obinrin agbegbe yi ma n lo lati lo yo oyun nipa abe lilo? Nibo lotun wa?

Do not read options aloud. Select all that apply.

(selected(${abt_ways},'surgery'))

☐ Ile Iwosan Ijoba nla
☐ Ile iwosan Ijoba alabode
☐ Ile iwosan ifeto somo bibi
☐ Ile iwosan agbe kiri (Titi Ijoba)
☐ TBA/Fieldworker (public)
☐ Ile iwosan Aladani
☐ Ile Ise ita ogun
☐ Ile Ita oogun/Chemisiti
☐ Dokita tabi noosi Aladani
☐ Ile iwosan agbe kiri (Ti Aladani)
☐ TBA/Fieldworker (private)
☐ Shobu
☐ Ile ise awon onigbagbo/church
☐ Oree/alabagbe po
☐ NGO
☐ Oja / olukiri oja
☐ Omiran
☐ N ko mo
☐ Ko si esi

---

709. Ewo ninu awon ibi wonyi ni won ma n SABA lo?

count-selected(${abt_surg_where}) > 1

○ Ile Iwosan Ijoba nla
○ Ile iwosan Ijoba alabode
○ Ile iwosan ifeto somo bibi

- Ile iwosan agbe kiri (Titi Ijoba)
- TBA/Fieldworker (public)
- Ile iwosan Aladani
- Ile Ise ita ogun
- Ile Ita oogun/Chemisiti
- Dokita tabi noosi Aladani
- Ile iwosan agbe kiri (Ti Aladani)
- TBA/Fieldworker (private)
- Shobu
- Ile ise awon onigbagbo/church
- Oree/alabagbe po
- NGO
- Oja / olukiri oja
- Omiran
- N ko mo
- Ko si esi

selected(${abt_surg_where}, filter) or (filter = 'always')

---

710. Nibo ni awon obinrin ti ma n ri ogun ti won n lo lati yo oyun? Ibo lo tun wa?

(selected(${abt_ways}, 'pills_abortion')) or (selected(${abt_ways}, 'pills_fever')) or (selected($ …

- [ ] Ile Iwosan Ijoba nla
- [ ] Ile iwosan Ijoba alabode
- [ ] Ile iwosan ifeto somo bibi
- [ ] Ile iwosan agbe kiri (Titi Ijoba)
- [ ] TBA/Fieldworker (public)
- [ ] Ile iwosan Aladani
- [ ] Ile Ise ita ogun
- [ ] Ile Ita oogun/Chemisiti
- [ ] Dokita tabi noosi Aladani
- [ ] Ile iwosan agbe kiri (Ti Aladani)
- [ ] TBA/Fieldworker (private)
- [ ] Shobu
- [ ] Ile ise awon onigbagbo/church
- [ ] Oree/alabagbe po
- [ ] NGO
- [ ] Oja / olukiri oja
- [ ] Omiran
- [ ] N ko mo
- [ ] Ko si esi

---

711. Ewo ninu awon ona wonyi ni won maa n SABA lo?

count-selected(${abt_meds_where}) > 1

- Ile Iwosan Ijoba nla
- Ile iwosan Ijoba alabode
- Ile iwosan ifeto somo bibi
- Ile iwosan agbe kiri (Titi Ijoba)
- TBA/Fieldworker (public)
- Ile iwosan Aladani
- Ile Ise ita ogun
- Ile Ita oogun/Chemisiti
- Dokita tabi noosi Aladani
- Ile iwosan agbe kiri (Ti Aladani)
- TBA/Fieldworker (private)
- Shobu
- Ile ise awon onigbagbo/church
- Oree/alabagbe po
- NGO
- Oja / olukiri oja
- Omiran
- N ko mo
- Ko si esi

selected(${abt_meds_where}, filter) or (filter = 'always')

712a.i. Bayi, mo fe bere awon ibeere ra n pe si lori ore ${friend1_name}. Nje O ti se ohun kan ri lati yo oyun nigba to loyun tabi ko aya soke lori oyun nini?<br>*Probe to confirm whether the pregnancy removal was successful.*	○ Yes, I am certain<br>○ Yes, I think so<br>○ Beeko<br>○ N ko mo<br>○ ko si esi
713a.i. Ninu odun woo leyi se le keyin?<br>*If indicates happened more than once, specify most recent time.*<br>*Enter 2020 for 'Do not know' or 'No response'.*	(${friend1_abt_yn} = 'yes') or (${friend1_abt_yn} = 'likely')<br>Year: ____________________
714a.i. Awon obinrin ma n se awon nkankan lati dena oyun nini. Nje ${friend1_name} se ju ohun kan lo lati yo oyun?	(${friend1_abt_yn} = 'yes') or (${friend1_abt_yn} = 'likely')<br>○ Yes, I am certain<br>○ Yes, I think so<br>○ Beeko<br>○ N ko mo<br>○ ko si esi
715a.i. Kini OHUN AKOKO ti o se lati le yo oyun?	(${friend1_abt_mult_yn} = 'yes') or (${friend1_abt_mult_yn} = 'likely')<br>○ Abee sisse<br>○ Ogun oni koro ti oruko re n je mifepristone or misoprostol bi apere Mariprist, Mifepak, Cytotec, Miso-Fem, or Misoclear<br>○ Ogun oni koro te maa n lo nigba ti e ba ni iba bii egbogi tabi ogun iba<br>○ Ogun Onikooro Pajawiri<br>○ Ogun koro miran<br>○ Abeere<br>○ Egbogi tabi awon ona ibile, bi agbo<br>○ Ogogoro<br>○ Iyo, eeru, magi, kahun<br>○ Osan wewe<br>○ Ogun iko olomi<br>○ Fi fi nkan si inu oju ara obinrin<br>○ Omiran<br>○ N ko mo<br>○ Ko si esi
715a.i. Ki lo se lati yo oyun na?	(${friend1_abt_mult_yn} = 'no') or (${friend1_abt_mult_yn} = '-88')<br>○ Abee sisse<br>○ Ogun oni koro ti oruko re n je mifepristone or misoprostol bi apere Mariprist, Mifepak, Cytotec, Miso-Fem, or Misoclear<br>○ Ogun oni koro te maa n lo nigba ti e ba ni iba bii egbogi tabi ogun iba<br>○ Ogun Onikooro Pajawiri<br>○ Ogun koro miran<br>○ Abeere<br>○ Egbogi tabi awon ona ibile, bi agbo<br>○ Ogogoro<br>○ Iyo, eeru, magi, kahun<br>○ Osan wewe<br>○ Ogun iko olomi<br>○ Fi fi nkan si inu oju ara obinrin<br>○ Omiran<br>○ N ko mo<br>○ Ko si esi
716a.i. Ni bo lo fun awon ona na?	(${friend1_abt_first} = 'surgery') or (${friend1_abt_only} = 'surgery')<br>○ Ile Iwosan Ijoba nla

	○ Ile iwosan Ijoba alabode<br>○ Ile iwosan ifeto somo bibi<br>○ Ile iwosan agbe kiri (Titi Ijoba)<br>○ TBA/Fieldworker (public)<br>○ Ile iwosan Aladani<br>○ Ile Ise ita ogun<br>○ Ile Ita oogun/Chemisiti<br>○ Dokita tabi noosi Aladani<br>○ Ile iwosan agbe kiri (Ti Aladani)<br>○ TBA/Fieldworker (private)<br>○ Shobu<br>○ Ile ise awon onigbagbo/church<br>○ Oree/alabagbe po<br>○ NGO<br>○ Oja / olukiri oja<br>○ Omiran<br>○ N ko mo<br>○ Ko si esi
717a.i. Ni bo lo ti ri ogun na?	(${friend1_abt_first} = 'pills_abortion') or (${friend1_abt_only} = 'pills_abortion') or (${friend …<br>○ Ile Iwosan Ijoba nla<br>○ Ile iwosan Ijoba alabode<br>○ Ile iwosan ifeto somo bibi<br>○ Ile iwosan agbe kiri (Titi Ijoba)<br>○ TBA/Fieldworker (public)<br>○ Ile iwosan Aladani<br>○ Ile Ise ita ogun<br>○ Ile Ita oogun/Chemisiti<br>○ Dokita tabi noosi Aladani<br>○ Ile iwosan agbe kiri (Ti Aladani)<br>○ TBA/Fieldworker (private)<br>○ Shobu<br>○ Ile ise awon onigbagbo/church<br>○ Oree/alabagbe po<br>○ NGO<br>○ Oja / olukiri oja<br>○ Omiran<br>○ N ko mo<br>○ Ko si esi
718a.i. Kini ohun to se kehin ti o se OKUNFA fun yiyo oyun na ?	(${friend1_abt_mult_yn} = 'yes') or (${friend1_abt_mult_yn} = 'likely')<br>○ Abee sisse<br>○ Ogun oni koro ti oruko re n je mifepristone or misoprostol bi apere Mariprist, Mifepak, Cytotec, Miso-Fem, or Misoclear<br>○ Ogun oni koro te maa n lo nigba ti e ba ni iba bii egbogi tabi ogun iba<br>○ Ogun Onikooro Pajawiri<br>○ Ogun koro miran<br>○ Abeere<br>○ Egbogi tabi awon ona ibile, bi agbo<br>○ Ogogoro<br>○ Iyo, eeru, magi, kahun<br>○ Osan wewe<br>○ Ogun iko olomi<br>○ Fi fi nkan si inu oju ara obinrin<br>○ Omiran<br>○ N ko mo

719a.i. Ni bo lo fun ona na?	Ko si esi<br>${friend1_abt_last} = 'surgery'<br>Ile Iwosan Ijoba nla<br>Ile iwosan Ijoba alabode<br>Ile iwosan ifeto somo bibi<br>Ile iwosan agbe kiri (Titi Ijoba)<br>TBA/Fieldworker (public)<br>Ile iwosan Aladani<br>Ile Ise ita ogun<br>Ile Ita oogun/Chemisiti<br>Dokita tabi noosi Aladani<br>Ile iwosan agbe kiri (Ti Aladani)<br>TBA/Fieldworker (private)<br>Shobu<br>Ile ise awon onigbagbo/church<br>Oree/alabagbe po<br>NGO<br>Oja / olukiri oja<br>Omiran<br>N ko mo<br>Ko si esi
720a.i. Ni bo lo ti ri ogun na?	(${friend1_abt_last} = 'pills_abortion') or<br>(${friend1_abt_last} = 'pills_fever') or<br>(${friend1_ab …<br>Ile Iwosan Ijoba nla<br>Ile iwosan Ijoba alabode<br>Ile iwosan ifeto somo bibi<br>Ile iwosan agbe kiri (Titi Ijoba)<br>TBA/Fieldworker (public)<br>Ile iwosan Aladani<br>Ile Ise ita ogun<br>Ile Ita oogun/Chemisiti<br>Dokita tabi noosi Aladani<br>Ile iwosan agbe kiri (Ti Aladani)<br>TBA/Fieldworker (private)<br>Shobu<br>Ile ise awon onigbagbo/church<br>Oree/alabagbe po<br>NGO<br>Oja / olukiri oja<br>Omiran<br>N ko mo<br>Ko si esi
721a.i. Nje ${friend1_name} ni awon isoro to dojuko nigba ti o n yo oyun ti o mu o si ile iwosan?<br>*If the respondent already reported the friend went to a health facility in the process of removing the pregnancy, we are interested in whether the friend went back to a health facility on a separate occasion to treat complications that she may have experienced.*	(${friend1_abt_yn} = 'yes') or<br>(${friend1_abt_yn} = 'likely')<br>Yes, I am certain<br>Yes, I think so<br>Beeko<br>N ko mo<br>ko si esi
712a.ii. Leyin eyin, Nje ${friend1_name} o ti se ohun kan ri lati je ki nkan osu pada bo si po nigba ti o ko aya soke pe oun loyun?<br>*Probe to confirm whether the period regulation was successful. If not, select 'no.'*	${friend1_abt_yn} = 'yes'<br>Yes, I am certain<br>Yes, I think so<br>Beeko<br>N ko mo<br>ko si esi
712a.ii. Nje ${friend1_name} o ti se ohun kan ri lati je ki nkan osu pada bo si po nigba ti o ko aya soke pe oun loyun??<br>*Probe to confirm whether the period regulation was successful. If not, select 'no.'*	${friend1_abt_yn} != 'yes'<br>Yes, I am certain<br>Yes, I think so<br>Beeko<br>N ko mo<br>ko si esi

713a.ii. Ninu odun woo leyi se le keyin?<br>*If indicates happened more than once, specify most recent time.*<br>*Enter 2020 for 'Do not know' or 'No response'.*	(${friend1_reg_yn} = 'yes') or (${friend1_reg_yn} = 'likely')<br>Year: ________
714a.ii. Awon obinrin ma n se awon nkankan lati dena oyun nini. Nje ${friend1_name} se ju ohun kan lo lati yo oyun?	((${friend1_reg_year} > ${friend1_abt_year}) or (${friend1_abt_year} = '')) and ((${friend1_reg_yn …<br>◯ Yes, I am certain<br>◯ Yes, I think so<br>◯ Beeko<br>◯ N ko mo<br>◯ ko si esi
715a.ii. Kini OHUN AKOKO ti o se lati le yo oyun?	((${friend1_reg_year} > ${friend1_abt_year}) or (${friend1_abt_year} = '')) and ((${friend1_reg_mu …<br>◯ Abee sisse<br>◯ Ogun oni koro ti oruko re n je mifepristone or misoprostol bi apere Mariprist, Mifepak, Cytotec, Miso-Fem, or Misoclear<br>◯ Ogun oni koro te maa n lo nigba ti e ba ni iba bii egbogi tabi ogun iba<br>◯ Ogun Onikooro Pajawiri<br>◯ Ogun koro miran<br>◯ Abeere<br>◯ Egbogi tabi awon ona ibile, bi agbo<br>◯ Ogogoro<br>◯ Iyo, eeru, magi, kahun<br>◯ Osan wewe<br>◯ Ogun iko olomi<br>◯ Fi fi nkan si inu oju ara obinrin<br>◯ Omiran<br>◯ N ko mo<br>◯ Ko si esi
715a.ii. Ki lo se lati yo oyun na?	((${friend1_reg_year} > ${friend1_abt_year}) or (${friend1_abt_year} = '')) and ((${friend1_reg_mu …<br>◯ Abee sisse<br>◯ Ogun oni koro ti oruko re n je mifepristone or misoprostol bi apere Mariprist, Mifepak, Cytotec, Miso-Fem, or Misoclear<br>◯ Ogun oni koro te maa n lo nigba ti e ba ni iba bii egbogi tabi ogun iba<br>◯ Ogun Onikooro Pajawiri<br>◯ Ogun koro miran<br>◯ Abeere<br>◯ Egbogi tabi awon ona ibile, bi agbo<br>◯ Ogogoro<br>◯ Iyo, eeru, magi, kahun<br>◯ Osan wewe<br>◯ Ogun iko olomi<br>◯ Fi fi nkan si inu oju ara obinrin<br>◯ Omiran<br>◯ N ko mo<br>◯ Ko si esi
716a.ii. Ni bo lo fun fun awon ona na?	((${friend1_reg_year} > ${friend1_abt_year}) or (${friend1_abt_year} = '')) and ((${friend1_reg_fi …<br>◯ Ile Iwosan Ijoba nla<br>◯ Ile iwosan Ijoba alabode

	◌ Ile iwosan ifeto somo bibi<br>◌ Ile iwosan agbe kiri (Titi Ijoba)<br>◌ TBA/Fieldworker (public)<br>◌ Ile iwosan Aladani<br>◌ Ile Ise ita ogun<br>◌ Ile Ita oogun/Chemisiti<br>◌ Dokita tabi noosi Aladani<br>◌ Ile iwosan agbe kiri (Ti Aladani)<br>◌ TBA/Fieldworker (private)<br>◌ Shobu<br>◌ Ile ise awon onigbagbo/church<br>◌ Oree/alabagbe po<br>◌ NGO<br>◌ Oja / olukiri oja<br>◌ Omiran<br>◌ N ko mo<br>◌ Ko si esi
717a.ii. Ni bo lo ti ri ogun na?	((${friend1_reg_year} > ${friend1_abt_year}) or (${friend1_abt_year} = '')) and ((${friend1_reg_fi …<br>◌ Ile Iwosan Ijoba nla<br>◌ Ile iwosan Ijoba alabode<br>◌ Ile iwosan ifeto somo bibi<br>◌ Ile iwosan agbe kiri (Titi Ijoba)<br>◌ TBA/Fieldworker (public)<br>◌ Ile iwosan Aladani<br>◌ Ile Ise ita ogun<br>◌ Ile Ita oogun/Chemisiti<br>◌ Dokita tabi noosi Aladani<br>◌ Ile iwosan agbe kiri (Ti Aladani)<br>◌ TBA/Fieldworker (private)<br>◌ Shobu<br>◌ Ile ise awon onigbagbo/church<br>◌ Oree/alabagbe po<br>◌ NGO<br>◌ Oja / olukiri oja<br>◌ Omiran<br>◌ N ko mo<br>◌ Ko si esi
718a.ii. Kini ohun to se kehin ti o se OKUNFA fun yiyo oyun na ?	((${friend1_reg_year} > ${friend1_abt_year}) or (${friend1_abt_year} = '')) and ((${friend1_reg_mu …<br>◌ Abee sisse<br>◌ Ogun oni koro ti oruko re n je mifepristone or misoprostol bi apere Mariprist, Mifepak, Cytotec, Miso-Fem, or Misoclear<br>◌ Ogun oni koro te maa n lo nigba ti e ba ni iba bii egbogi tabi ogun iba<br>◌ Ogun Onikooro Pajawiri<br>◌ Ogun koro miran<br>◌ Abeere<br>◌ Egbogi tabi awon ona ibile, bi agbo<br>◌ Ogogoro<br>◌ Iyo, eeru, magi, kahun<br>◌ Osan wewe<br>◌ Ogun iko olomi<br>◌ Fi fi nkan si inu oju ara obinrin<br>◌ Omiran<br>◌ N ko mo<br>◌ Ko si esi

719a.ii. Ni bo le ti ri ona abayo?	((${friend1_reg_year} > ${friend1_abt_year}) or (${friend1_abt_year} = '')) and (${friend1_reg_las …<br>○ Ile Iwosan Ijoba nla<br>○ Ile iwosan Ijoba alabode<br>○ Ile iwosan ifeto somo bibi<br>○ Ile iwosan agbe kiri (Titi Ijoba)<br>○ TBA/Fieldworker (public)<br>○ Ile iwosan Aladani<br>○ Ile Ise ita ogun<br>○ Ile Ita oogun/Chemisiti<br>○ Dokita tabi noosi Aladani<br>○ Ile iwosan agbe kiri (Ti Aladani)<br>○ TBA/Fieldworker (private)<br>○ Shobu<br>○ Ile ise awon onigbagbo/church<br>○ Oree/alabagbe po<br>○ NGO<br>○ Oja / olukiri oja<br>○ Omiran<br>○ N ko mo<br>○ Ko si esi
720a.ii. Ni bo lo ti ri ogun na?	((${friend1_reg_year} > ${friend1_abt_year}) or (${friend1_abt_year} = '')) and ((${friend1_reg_la …<br>○ Ile Iwosan Ijoba nla<br>○ Ile iwosan Ijoba alabode<br>○ Ile iwosan ifeto somo bibi<br>○ Ile iwosan agbe kiri (Titi Ijoba)<br>○ TBA/Fieldworker (public)<br>○ Ile iwosan Aladani<br>○ Ile Ise ita ogun<br>○ Ile Ita oogun/Chemisiti<br>○ Dokita tabi noosi Aladani<br>○ Ile iwosan agbe kiri (Ti Aladani)<br>○ TBA/Fieldworker (private)<br>○ Shobu<br>○ Ile ise awon onigbagbo/church<br>○ Oree/alabagbe po<br>○ NGO<br>○ Oja / olukiri oja<br>○ Omiran<br>○ N ko mo<br>○ Ko si esi
721a.ii. Nje ${friend1_name} o ti se ohun kan ri lati je ki nkan osu pada bo si po nigba ti o ko aya soke pe oun loyun??<br>*If the respondent already reported the friend went to a health facility in the process of regulating her period, we are interested in whether the friend went back to a health facility on a separate occasion to treat complications that she may have experienced.*	((${friend1_reg_year} > ${friend1_abt_year}) or (${friend1_abt_year} = '')) and ((${friend1_reg_yn …<br>○ Yes, I am certain<br>○ Yes, I think so<br>○ Beeko<br>○ N ko mo<br>○ ko si esi
712b.i. Bayi, mo fe bere awon ibeere ra n pe si lori ore ${friend2_name}. Nje o ti se ohun kan ri lati yo oyun nigba to loyun tabi ko aya soke lori oyun nini?<br>*Probe to confirm whether the pregnancy removal was successful.*	○ Yes, I am certain<br>○ Yes, I think so<br>○ Beeko<br>○ N ko mo<br>○ ko si esi
713b.i. Ninu odun woo leyi se le keyin?<br>*If indicates happened more than once, specify most recent time.*<br>*Enter 2020 for 'Do not know' or 'No response'.*	(${friend2_abt_yn} = 'yes') or (${friend2_abt_yn} = 'likely')<br>Year: ___________________
714b.i. Awon obinrin ma n se awon nkankan lati dena oyun nini. Nje ${friend2_name} se ju ohun kan lo lati yo oyun?	(${friend2_abt_yn} = 'yes') or (${friend2_abt_yn} = 'likely')

	○ Yes, I am certain<br>○ Yes, I think so<br>○ Beeko<br>○ N ko mo<br>○ ~~ko si esi~~
715b.i. Kini OHUN AKOKO ti o se lati le yo oyun?	(${friend2_abt_mult_yn} = 'yes') or (${friend2_abt_mult_yn} = 'likely')<br>○ Abee sisse<br>○ Ogun oni koro ti oruko re n je mifepristone or misoprostol bi apere Mariprist, Mifepak, Cytotec, Miso-Fem, or Misoclear<br>○ Ogun oni koro te maa n lo nigba ti e ba ni iba bii egbogi tabi ogun iba<br>○ Ogun Onikooro Pajawiri<br>○ Ogun koro miran<br>○ Abeere<br>○ Egbogi tabi awon ona ibile, bi agbo<br>○ Ogogoro<br>○ Iyo, eeru, magi, kahun<br>○ Osan wewe<br>○ Ogun iko olomi<br>○ Fi fi nkan si inu oju ara obinrin<br>○ Omiran<br>○ N ko mo<br>○ Ko si esi
715b.i. Ki lo se lati yo oyun na?	(${friend2_abt_mult_yn} = 'no') or (${friend2_abt_mult_yn} = '-88')<br>○ Abee sisse<br>○ Ogun oni koro ti oruko re n je mifepristone or misoprostol bi apere Mariprist, Mifepak, Cytotec, Miso-Fem, or Misoclear<br>○ Ogun oni koro te maa n lo nigba ti e ba ni iba bii egbogi tabi ogun iba<br>○ Ogun Onikooro Pajawiri<br>○ Ogun koro miran<br>○ Abeere<br>○ Egbogi tabi awon ona ibile, bi agbo<br>○ Ogogoro<br>○ Iyo, eeru, magi, kahun<br>○ Osan wewe<br>○ Ogun iko olomi<br>○ Fi fi nkan si inu oju ara obinrin<br>○ Omiran<br>○ N ko mo<br>○ Ko si esi
716b.i. Ni bo lo fun awon ona na?	(${friend2_abt_first} = 'surgery') or (${friend2_abt_only} = 'surgery')<br>○ Ile Iwosan Ijoba nla<br>○ Ile iwosan Ijoba alabode<br>○ Ile iwosan ifeto somo bibi<br>○ Ile iwosan agbe kiri (Titi Ijoba)<br>○ TBA/Fieldworker (public)<br>○ Ile iwosan Aladani<br>○ Ile Ise ita ogun<br>○ Ile Ita oogun/Chemisiti<br>○ Dokita tabi noosi Aladani<br>○ Ile iwosan agbe kiri (Ti Aladani)<br>○ TBA/Fieldworker (private)

	Shobu<br>Ile ise awon onigbagbo/church<br>Oree/alabagbe po<br>NGO<br>Oja / olukiri oja<br>Omiran<br>N ko mo<br>Ko si esi
717b.i. Ni bo lo ti ri ogun na?	(${friend2_abt_first} = 'pills_abortion') or (${friend2_abt_only} = 'pills_abortion') or (${friend …<br>Ile Iwosan Ijoba nla<br>Ile iwosan Ijoba alabode<br>Ile iwosan ifeto somo bibi<br>Ile iwosan agbe kiri (Titi Ijoba)<br>TBA/Fieldworker (public)<br>Ile iwosan Aladani<br>Ile Ise ita ogun<br>Ile Ita oogun/Chemisiti<br>Dokita tabi noosi Aladani<br>Ile iwosan agbe kiri (Ti Aladani)<br>TBA/Fieldworker (private)<br>Shobu<br>Ile ise awon onigbagbo/church<br>Oree/alabagbe po<br>NGO<br>Oja / olukiri oja<br>Omiran<br>N ko mo<br>Ko si esi
718b.i. Kini ohun to se kehin ti o se OKUNFA fun yiyo oyun na ?	(${friend2_abt_mult_yn} = 'yes') or (${friend2_abt_mult_yn} = 'likely')<br>Abee sisse<br>Ogun oni koro ti oruko re n je mifepristone or misoprostol bi apere Mariprist, Mifepak, Cytotec, Miso-Fem, or Misoclear<br>Ogun oni koro te maa n lo nigba ti e ba ni iba bii egbogi tabi ogun iba<br>Ogun Onikooro Pajawiri<br>Ogun koro miran<br>Abeere<br>Egbogi tabi awon ona ibile, bi agbo<br>Ogogoro<br>Iyo, eeru, magi, kahun<br>Osan wewe<br>Ogun iko olomi<br>Fi fi nkan si inu oju ara obinrin<br>Omiran<br>N ko mo<br>Ko si esi
719b.i. Ni bo ni o lo fun ona na?	${friend2_abt_last} = 'surgery'<br>Ile Iwosan Ijoba nla<br>Ile iwosan Ijoba alabode<br>Ile iwosan ifeto somo bibi<br>Ile iwosan agbe kiri (Titi Ijoba)<br>TBA/Fieldworker (public)<br>Ile iwosan Aladani<br>Ile Ise ita ogun<br>Ile Ita oogun/Chemisiti<br>Dokita tabi noosi Aladani

	◯ Ile iwosan agbe kiri (Ti Aladani)<br>◯ TBA/Fieldworker (private)<br>◯ Shobu<br>◯ Ile ise awon onigbagbo/church<br>◯ Oree/alabagbe po<br>◯ NGO<br>◯ Oja / olukiri oja<br>◯ Omiran<br>◯ N ko mo<br>◯ Ko si esi
720b.i. Ni bo lo ti ri ogun na?	(${friend2_abt_last} = 'pills_abortion') or (${friend2_abt_last} = 'pills_fever') or (${friend2_ab …<br>◯ Ile Iwosan Ijoba nla<br>◯ Ile iwosan Ijoba alabode<br>◯ Ile iwosan ifeto somo bibi<br>◯ Ile iwosan agbe kiri (Titi Ijoba)<br>◯ TBA/Fieldworker (public)<br>◯ Ile iwosan Aladani<br>◯ Ile Ise ita ogun<br>◯ Ile Ita oogun/Chemisiti<br>◯ Dokita tabi noosi Aladani<br>◯ Ile iwosan agbe kiri (Ti Aladani)<br>◯ TBA/Fieldworker (private)<br>◯ Shobu<br>◯ Ile ise awon onigbagbo/church<br>◯ Oree/alabagbe po<br>◯ NGO<br>◯ Oja / olukiri oja<br>◯ Omiran<br>◯ N ko mo<br>◯ Ko si esi
721b.i. Nje ${friend2_name} ni awon isoro to dojuko nigba ti o n yo oyun ti o mu o si ile iwosan?<br>*Ti oludahun ba wipe ore re lo si ile iwosan lati yo oyun, o wu wa lati mo bo ya ore na lo si ile iwosan lati gba itoju lori idaamu miran ti o ba ni.*	(${friend2_abt_yn} = 'yes') or (${friend2_abt_yn} = 'likely')<br>◯ Yes, I am certain<br>◯ Yes, I think so<br>◯ Beeko<br>◯ N ko mo<br>◯ ko si esi
712b.ii. Leyin eyin, Nje ${friend2_name} o ti se ohun kan ri lati je ki nkan osu pada bo si po nigba ti o ko aya soke pe oun loyun?<br>*Probe to confirm whether the period regulation was successful. If not, select 'no.'*	${friend2_abt_yn} = 'yes'<br>◯ Yes, I am certain<br>◯ Yes, I think so<br>◯ Beeko<br>◯ N ko mo<br>◯ ko si esi
712b.ii. Nje ${friend2_name} o ti se ohun kan ri lati je ki nkan osu pada bo si po nigba ti o ko aya soke pe oun loyun?<br>*Probe to confirm whether the period regulation was successful. If not, select 'no.'*	${friend2_abt_yn} != 'yes'<br>◯ Yes, I am certain<br>◯ Yes, I think so<br>◯ Beeko<br>◯ N ko mo<br>◯ ko si esi
713b.ii. Ninu odun wo leyi se le keyin?<br>*If indicates happened more than once, specify most recent time.*<br>*Enter 2020 for 'Do not know' or 'No response'.*	(${friend2_reg_yn} = 'yes') or (${friend2_reg_yn} = 'likely')<br>Year: ______________________
714b.ii. Awon obinrin ma n se awon nkankan lati da nkan osu re pada bo si po. Nje ${friend2_name} se ju ohun kan lo lati da nkan osu e pada bo?	((${friend2_reg_year} > ${friend2_abt_year}) or (${friend2_abt_year} = '')) and ((${friend2_reg_yn …<br>◯ Yes, I am certain<br>◯ Yes, I think so<br>◯ Beeko<br>◯ N ko mo<br>◯ ko si esi

715b.ii. Kini OHUN AKOKO ti o se lati da nkan osu re pada bo si po?	((${friend2_reg_year} > ${friend2_abt_year}) or (${friend2_abt_year} = '')) and ((${friend2_reg_mu …<br>○ Abee sisse<br>○ Ogun oni koro ti oruko re n je mifepristone or misoprostol bi apere Mariprist, Mifepak, Cytotec, Miso-Fem, or Misoclear<br>○ Ogun oni koro te maa n lo nigba ti e ba ni iba bii egbogi tabi ogun iba<br>○ Ogun Onikooro Pajawiri<br>○ Ogun koro miran<br>○ Abeere<br>○ Egbogi tabi awon ona ibile, bi agbo<br>○ Ogogoro<br>○ Iyo, eeru, magi, kahun<br>○ Osan wewe<br>○ Ogun iko olomi<br>○ Fi fi nkan si inu oju ara obinrin<br>○ Omiran<br>○ N ko mo<br>○ Ko si esi
715b.ii. Ki lo se lati da nkan osu re pada bo si po?	((${friend2_reg_year} > ${friend2_abt_year}) or (${friend2_abt_year} = '')) and ((${friend2_reg_mu …<br>○ Abee sisse<br>○ Ogun oni koro ti oruko re n je mifepristone or misoprostol bi apere Mariprist, Mifepak, Cytotec, Miso-Fem, or Misoclear<br>○ Ogun oni koro te maa n lo nigba ti e ba ni iba bii egbogi tabi ogun iba<br>○ Ogun Onikooro Pajawiri<br>○ Ogun koro miran<br>○ Abeere<br>○ Egbogi tabi awon ona ibile, bi agbo<br>○ Ogogoro<br>○ Iyo, eeru, magi, kahun<br>○ Osan wewe<br>○ Ogun iko olomi<br>○ Fi fi nkan si inu oju ara obinrin<br>○ Omiran<br>○ N ko mo<br>○ Ko si esi
716b.ii. Ni bo ni o lo fun ona na?	((${friend2_reg_year} > ${friend2_abt_year}) or (${friend2_abt_year} = '')) and ((${friend2_reg_fi …<br>○ Ile Iwosan Ijoba nla<br>○ Ile iwosan Ijoba alabode<br>○ Ile iwosan ifeto somo bibi<br>○ Ile iwosan agbe kiri (Titi Ijoba)<br>○ TBA/Fieldworker (public)<br>○ Ile iwosan Aladani<br>○ Ile Ise ita ogun<br>○ Ile Ita oogun/Chemisiti<br>○ Dokita tabi noosi Aladani<br>○ Ile iwosan agbe kiri (Ti Aladani)<br>○ TBA/Fieldworker (private)<br>○ Shobu<br>○ Ile ise awon onigbagbo/church

	Oree/alabagbe po<br>NGO<br>Oja / olukiri oja<br>Omiran<br>N ko mo<br>Ko si esi
717b.ii. Ni bo lo ti ri ogun na?	((${friend2_reg_year} > ${friend2_abt_year}) or (${friend2_abt_year} = '')) and ((${friend2_reg_fi …<br>Ile Iwosan Ijoba nla<br>Ile iwosan Ijoba alabode<br>Ile iwosan ifeto somo bibi<br>Ile iwosan agbe kiri (Titi Ijoba)<br>TBA/Fieldworker (public)<br>Ile iwosan Aladani<br>Ile Ise ita ogun<br>Ile Ita oogun/Chemisiti<br>Dokita tabi noosi Aladani<br>Ile iwosan agbe kiri (Ti Aladani)<br>TBA/Fieldworker (private)<br>Shobu<br>Ile ise awon onigbagbo/church<br>Oree/alabagbe po<br>NGO<br>Oja / olukiri oja<br>Omiran<br>N ko mo<br>Ko si esi
718b.ii. Kini ohun to se kehin ti o se OKUNFA fun mumu nkan osu re pada bo si po ?	((${friend2_reg_year} > ${friend2_abt_year}) or (${friend2_abt_year} = '')) and ((${friend2_reg_mu …<br>Abee sisse<br>Ogun oni koro ti oruko re n je mifepristone or misoprostol bi apere Mariprist, Mifepak, Cytotec, Miso-Fem, or Misoclear<br>Ogun oni koro te maa n lo nigba ti e ba ni iba bii egbogi tabi ogun iba<br>Ogun Onikooro Pajawiri<br>Ogun koro miran<br>Abeere<br>Egbogi tabi awon ona ibile, bi agbo<br>Ogogoro<br>Iyo, eeru, magi, kahun<br>Osan wewe<br>Ogun iko olomi<br>Fi fi nkan si inu oju ara obinrin<br>Omiran<br>N ko mo<br>Ko si esi
719b.ii. Ni bo lo lo fun ona na?	((${friend2_reg_year} > ${friend2_abt_year}) or (${friend2_abt_year} = '')) and (${friend2_reg_las …<br>Ile Iwosan Ijoba nla<br>Ile iwosan Ijoba alabode<br>Ile iwosan ifeto somo bibi<br>Ile iwosan agbe kiri (Titi Ijoba)<br>TBA/Fieldworker (public)<br>Ile iwosan Aladani<br>Ile Ise ita ogun<br>Ile Ita oogun/Chemisiti

	○ Dokita tabi noosi Aladani<br>○ Ile iwosan agbe kiri (Ti Aladani)<br>○ TBA/Fieldworker (private)<br>○ Shobu<br>○ Ile ise awon onigbagbo/church<br>○ Oree/alabagbe po<br>○ NGO<br>○ Oja / olukiri oja<br>○ Omiran<br>○ N ko mo<br>○ Ko si esi
720b.ii. Ni bo lo ti ri ogun na?	((${friend2_reg_year} > ${friend2_abt_year}) or (${friend2_abt_year} = '')) and ((${friend2_reg_la …<br>○ Ile Iwosan Ijoba nla<br>○ Ile iwosan Ijoba alabode<br>○ Ile iwosan ifeto somo bibi<br>○ Ile iwosan agbe kiri (Titi Ijoba)<br>○ TBA/Fieldworker (public)<br>○ Ile iwosan Aladani<br>○ Ile Ise ita ogun<br>○ Ile Ita oogun/Chemisiti<br>○ Dokita tabi noosi Aladani<br>○ Ile iwosan agbe kiri (Ti Aladani)<br>○ TBA/Fieldworker (private)<br>○ Shobu<br>○ Ile ise awon onigbagbo/church<br>○ Oree/alabagbe po<br>○ NGO<br>○ Oja / olukiri oja<br>○ Omiran<br>○ N ko mo<br>○ Ko si esi
721b.ii. Nje ${friend2_name} ni awon isoro to dojuko nigba ti o fe je ki nkan osu e pada bo sipo ti o mu lo si ile iwosan?<br>*If the respondent already reported the friend went to a health facility in the process of regulating her period, we are interested in whether the friend went back to a health facility on a separate occasion to treat complications that she may have experienced.*	((${friend2_reg_year} > ${friend2_abt_year}) or (${friend2_abt_year} = '')) and ((${friend2_reg_yn …<br>○ Yes, I am certain<br>○ Yes, I think so<br>○ Beeko<br>○ N ko mo<br>○ ko si esi
722a. Bayi mo fe bere awon iriri yin. Nje eti se ohun kan lati yo oyun tabi se se oyun nigba ti e loyun tabi ko aya soke nigba ti e loyun<br>*Probe to confirm whether the pregnancy removal was successful.*	○ Beeni<br>○ Beeko<br>○ ko si esi
723a. Ninu odu wo ni eyi se le kehin?<br>*If indicates happened more than once, specify most recent time.*<br>*Enter 2020 for 'Do not know' or 'No response'.*	(${self_abt_yn} = 'yes')<br>Year: ________________
724a. Nje ese ju ohun kan lo lati yo oyun?	(${self_abt_yn} = 'yes')<br>○ Beeni<br>○ Beeko<br>○ ko si esi
725a. Ki le KOKO se gan?	(${self_abt_mult_yn} = 'yes')<br>○ Abee sisse<br>○ Ogun oni koro ti oruko re n je mifepristone or misoprostol bi apere Mariprist, Mifepak, Cytotec, Miso-Fem, or Misoclear<br>○ Ogun oni koro te maa n lo nigba ti e ba ni iba bii egbogi tabi ogun iba<br>○ Ogun Onikooro Pajawiri<br>○ Ogun koro miran

	Abeere<br>Egbogi tabi awon ona ibile, bi agbo<br>Ogogoro<br>Iyo, eeru, magi, kahun<br>Osan wewe<br>Ogun iko olomi<br>Fi fi nkan si inu oju ara obinrin<br>Omiran<br>N ko mo<br>Ko si esi
725a. Ki le se?	(${self_abt_mult_yn} = 'no')<br>Abee sisse<br>Ogun oni koro ti oruko re n je mifepristone or misoprostol bi apere Mariprist, Mifepak, Cytotec, Miso-Fem, or Misoclear<br>Ogun oni koro te maa n lo nigba ti e ba ni iba bii egbogi tabi ogun iba<br>Ogun Onikooro Pajawiri<br>Ogun koro miran<br>Abeere<br>Egbogi tabi awon ona ibile, bi agbo<br>Ogogoro<br>Iyo, eeru, magi, kahun<br>Osan wewe<br>Ogun iko olomi<br>Fi fi nkan si inu oju ara obinrin<br>Omiran<br>N ko mo<br>Ko si esi
726a. Ni bo le lo fun ona na?	(${self_abt_first} = 'surgery') or (${self_abt_only} = 'surgery')<br>Ile Iwosan Ijoba nla<br>Ile iwosan Ijoba alabode<br>Ile iwosan ifeto somo bibi<br>Ile iwosan agbe kiri (Titi Ijoba)<br>TBA/Fieldworker (public)<br>Ile iwosan Aladani<br>Ile Ise ita ogun<br>Ile Ita oogun/Chemisiti<br>Dokita tabi noosi Aladani<br>Ile iwosan agbe kiri (Ti Aladani)<br>TBA/Fieldworker (private)<br>Shobu<br>Ile ise awon onigbagbo/church<br>Oree/alabagbe po<br>NGO<br>Oja / olukiri oja<br>Omiran<br>N ko mo<br>Ko si esi
727a. Ni bo le ti ri ogun?	(${self_abt_first} = 'pills_abortion') or (${self_abt_only} = 'pills_abortion') or (${self_abt_fir …<br>Ile Iwosan Ijoba nla<br>Ile iwosan Ijoba alabode<br>Ile iwosan ifeto somo bibi<br>Ile iwosan agbe kiri (Titi Ijoba)<br>TBA/Fieldworker (public)<br>Ile iwosan Aladani

	○ Ile Ise ita ogun<br>○ Ile Ita oogun/Chemisiti<br>○ Dokita tabi noosi Aladani<br>○ Ile iwosan agbe kiri (Ti Aladani)<br>○ TBA/Fieldworker (private)<br>○ Shobu<br>○ Ile ise awon onigbagbo/church<br>○ Oree/alabagbe po<br>○ NGO<br>○ Oja / olukiri oja<br>○ Omiran<br>○ N ko mo<br>○ Ko si esi
728a. Kini ohun ti e se kehin to SOKUNFA yiyo oyun na?	(${self_abt_mult_yn} = 'yes')<br>○ Abee sisse<br>○ Ogun oni koro ti oruko re n je mifepristone or misoprostol bi apere Mariprist, Mifepak, Cytotec, Miso-Fem, or Misoclear<br>○ Ogun oni koro te maa n lo nigba ti e ba ni iba bii egbogi tabi ogun iba<br>○ Ogun Onikooro Pajawiri<br>○ Ogun koro miran<br>○ Abeere<br>○ Egbogi tabi awon ona ibile, bi agbo<br>○ Ogogoro<br>○ Iyo, eeru, magi, kahun<br>○ Osan wewe<br>○ Ogun iko olomi<br>○ Fi fi nkan si inu oju ara obinrin<br>○ Omiran<br>○ N ko mo<br>○ Ko si esi
729a. Ni bo le lo fun ona na?	${self_abt_last} = 'surgery'<br>○ Ile Iwosan Ijoba nla<br>○ Ile iwosan Ijoba alabode<br>○ Ile iwosan ifeto somo bibi<br>○ Ile iwosan agbe kiri (Titi Ijoba)<br>○ TBA/Fieldworker (public)<br>○ Ile iwosan Aladani<br>○ Ile Ise ita ogun<br>○ Ile Ita oogun/Chemisiti<br>○ Dokita tabi noosi Aladani<br>○ Ile iwosan agbe kiri (Ti Aladani)<br>○ TBA/Fieldworker (private)<br>○ Shobu<br>○ Ile ise awon onigbagbo/church<br>○ Oree/alabagbe po<br>○ NGO<br>○ Oja / olukiri oja<br>○ Omiran<br>○ N ko mo<br>○ Ko si esi
730a. Ni bo le ti ri ogun?	(${self_abt_last} = 'pills_abortion') or (${self_abt_last} = 'pills_fever') or (${self_abt_last} = …<br>○ Ile Iwosan Ijoba nla<br>○ Ile iwosan Ijoba alabode<br>○ Ile iwosan ifeto somo bibi<br>○ Ile iwosan agbe kiri (Titi Ijoba)<br>○ TBA/Fieldworker (public)

	◯ Ile iwosan Aladani<br>◯ Ile Ise ita ogun<br>◯ Ile Ita oogun/Chemisiti<br>◯ Dokita tabi noosi Aladani<br>◯ Ile iwosan agbe kiri (Ti Aladani)<br>◯ TBA/Fieldworker (private)<br>◯ Shobu<br>◯ Ile ise awon onigbagbo/church<br>◯ Oree/alabagbe po<br>◯ NGO<br>◯ Oja / olukiri oja<br>◯ Omiran<br>◯ N ko mo<br>◯ Ko si esi
731a. Nje eni awon isoro ti e dojuko nigba ti e fe yo oyun ti o mu yin lo si ile iwosan?<br>*If the respondent already reported she went to a health facility in the process of removing the pregnancy, we are interested in whether she went back to a health facility on a separate occasion to treat complications that she may have experienced.*	(${self_abt_yn} = 'yes')<br>◯ Beeni<br>◯ Beeko<br>◯ N ko mo<br>◯ ko si esi
732a. Nje e so fun enikeni ninu awon wonyi nipa iriri yin?<br>*Read the answer choices aloud. Select all that apply.*	(${self_abt_yn} = 'yes')<br>☐ Oko/Ore kunrin<br>☐ Sister<br>☐ Egbon Okunrin<br>☐ Iya<br>☐ Baba<br>☐ Ara Ile miran<br>☐ Ore Kiini: ${friend1_name}<br>☐ Ore Keji: ${friend2_name}<br>☐ Ore Miran<br>☐ Omiran<br>☐ N ko mo<br>☐ ko si esi<br>(${friend1_name} != '' and ${friend1_name} != '-99' and filter = 'friend1') or (${friend2_name} != '' and ${friend2_name} != '-99' and filter = 'friend2') or (filter = 'always')
722b. Yato si eyi, nje e se nkan kan ri lati da nkan osu yin padabo si po ni gba ti e ko aya soke pe eloyun?<br>*Probe to confirm whether the period regulation was successful. If not, select 'no.'*	${self_abt_yn} = 'yes'<br>◯ Beeni<br>◯ Beeko<br>◯ ko si esi
722b. Nje e ti ti lo se nkan kan ri lati le je ki nkan osu yin pada bo si po nigba ti e ko aya soke pe e loyun?<br>*Probe to confirm whether the period regulation was successful. If not, select 'no.'*	${self_abt_yn} != 'yes'<br>◯ Beeni<br>◯ Beeko<br>◯ ko si esi
723b. Ninu odu wo ni eyi se le kehin?<br>*If indicates happened more than once, specify most recent time.*<br>*Enter 2020 for 'Do not know' or 'No response'.*	(${self_reg_yn} = 'yes')<br>Year: ----------------------------------
724b. Nje ese ju ohun kan lo lati le je ki nkan osu yin pada bo si po?	((${self_reg_year} > ${self_abt_year}) or (${self_abt_year} = '')) and ((${self_reg_yn} = 'yes'))<br>◯ Beeni<br>◯ Beeko<br>◯ ko si esi
725b. Kini ohun akoko te se?	((${self_reg_year} > ${self_abt_year}) or (${self_abt_year} = '')) and ((${self_reg_mult_yn} = 'ye …<br>◯ Abee sisse<br>◯ Ogun oni koro ti oruko re n je mifepristone or misoprostol bi apere Mariprist, Mifepak, Cytotec, Miso-Fem, or Misoclear<br>◯ Ogun oni koro te maa n lo nigba ti

	e ba ni iba bii egbogi tabi ogun iba<br>Ogun Onikooro Pajawiri<br>Ogun koro miran<br>Abeere<br>Egbogi tabi awon ona ibile, bi agbo<br>Ogogoro<br>Iyo, eeru, magi, kahun<br>Osan wewe<br>Ogun iko olomi<br>Fi fi nkan si inu oju ara obinrin<br>Omiran<br>N ko mo<br>Ko si esi
725b. Ki le se?	((${self_reg_year} > ${self_abt_year}) or (${self_abt_year} = '')) and ((${self_reg_mult_yn} = 'no …<br>Abee sisse<br>Ogun oni koro ti oruko re n je mifepristone or misoprostol bi apere Mariprist, Mifepak, Cytotec, Miso-Fem, or Misoclear<br>Ogun oni koro te maa n lo nigba ti e ba ni iba bii egbogi tabi ogun iba<br>Ogun Onikooro Pajawiri<br>Ogun koro miran<br>Abeere<br>Egbogi tabi awon ona ibile, bi agbo<br>Ogogoro<br>Iyo, eeru, magi, kahun<br>Osan wewe<br>Ogun iko olomi<br>Fi fi nkan si inu oju ara obinrin<br>Omiran<br>N ko mo<br>Ko si esi
726b. Ni bo le lo fun ona na?	((${self_reg_year} > ${self_abt_year}) or (${self_abt_year} = '')) and ((${self_reg_first} = 'surg …<br>Ile Iwosan Ijoba nla<br>Ile iwosan Ijoba alabode<br>Ile iwosan ifeto somo bibi<br>Ile iwosan agbe kiri (Titi Ijoba)<br>TBA/Fieldworker (public)<br>Ile iwosan Aladani<br>Ile Ise ita ogun<br>Ile Ita oogun/Chemisiti<br>Dokita tabi noosi Aladani<br>Ile iwosan agbe kiri (Ti Aladani)<br>TBA/Fieldworker (private)<br>Shobu<br>Ile ise awon onigbagbo/church<br>Oree/alabagbe po<br>NGO<br>Oja / olukiri oja<br>Omiran<br>N ko mo<br>Ko si esi
727b. Ni bo le ti ri ogun?	((${self_reg_year} > ${self_abt_year}) or (${self_abt_year} = '')) and ((${self_reg_first} = 'pill …

	◌ Ile Iwosan Ijoba nla<br>◌ Ile iwosan Ijoba alabode<br>◌ Ile iwosan ifeto somo bibi<br>◌ Ile iwosan agbe kiri (Titi Ijoba)<br>◌ TBA/Fieldworker (public)<br>◌ Ile iwosan Aladani<br>◌ Ile Ise ita ogun<br>◌ Ile Ita oogun/Chemisiti<br>◌ Dokita tabi noosi Aladani<br>◌ Ile iwosan agbe kiri (Ti Aladani)<br>◌ TBA/Fieldworker (private)<br>◌ Shobu<br>◌ Ile ise awon onigbagbo/church<br>◌ Oree/alabagbe po<br>◌ NGO<br>◌ Oja / olukiri oja<br>◌ Omiran<br>◌ N ko mo<br>◌ Ko si esi
728b. Kini ohun ti e se kehin to sokun fa ki nkan osu yin pada bo sipo?	((${self_reg_year} > ${self_abt_year}) or (${self_abt_year} = '')) and ((${self_reg_mult_yn} = 'ye …<br>◌ Abee sisse<br>◌ Ogun oni koro ti oruko re n je mifepristone or misoprostol bi apere Mariprist, Mifepak, Cytotec, Miso-Fem, or Misoclear<br>◌ Ogun oni koro te maa n lo nigba ti e ba ni iba bii egbogi tabi ogun iba<br>◌ Ogun Onikooro Pajawiri<br>◌ Ogun koro miran<br>◌ Abeere<br>◌ Egbogi tabi awon ona ibile, bi agbo<br>◌ Ogogoro<br>◌ Iyo, eeru, magi, kahun<br>◌ Osan wewe<br>◌ Ogun iko olomi<br>◌ Fi fi nkan si inu oju ara obinrin<br>◌ Omiran<br>◌ N ko mo<br>◌ Ko si esi
729b. Ni bo le lo fun ona na?	((${self_reg_year} > ${self_abt_year}) or (${self_abt_year} = '')) and (${self_reg_last} = 'surger …<br>◌ Ile Iwosan Ijoba nla<br>◌ Ile iwosan Ijoba alabode<br>◌ Ile iwosan ifeto somo bibi<br>◌ Ile iwosan agbe kiri (Titi Ijoba)<br>◌ TBA/Fieldworker (public)<br>◌ Ile iwosan Aladani<br>◌ Ile Ise ita ogun<br>◌ Ile Ita oogun/Chemisiti<br>◌ Dokita tabi noosi Aladani<br>◌ Ile iwosan agbe kiri (Ti Aladani)<br>◌ TBA/Fieldworker (private)<br>◌ Shobu<br>◌ Ile ise awon onigbagbo/church<br>◌ Oree/alabagbe po<br>◌ NGO<br>◌ Oja / olukiri oja<br>◌ Omiran<br>◌ N ko mo

	◯ Ko si esi
730b. Ni bo le ti ri ogun?	((${self_reg_year} > ${self_abt_year}) or (${self_abt_year} = '')) and ((${self_reg_last} = 'pills …<br>◯ Ile Iwosan Ijoba nla<br>◯ Ile iwosan Ijoba alabode<br>◯ Ile iwosan ifeto somo bibi<br>◯ Ile iwosan agbe kiri (Titi Ijoba)<br>◯ TBA/Fieldworker (public)<br>◯ Ile iwosan Aladani<br>◯ Ile Ise ita ogun<br>◯ Ile Ita oogun/Chemisiti<br>◯ Dokita tabi noosi Aladani<br>◯ Ile iwosan agbe kiri (Ti Aladani)<br>◯ TBA/Fieldworker (private)<br>◯ Shobu<br>◯ Ile ise awon onigbagbo/church<br>◯ Oree/alabagbe po<br>◯ NGO<br>◯ Oja / olukiri oja<br>◯ Omiran<br>◯ N ko mo<br>◯ Ko si esi
731b. Nje e ni awon isoro ti e dojuko lati lo si ile iwosan fun itoju nigba ti o n yoo oyun?<br>*If the respondent already reported she went to a health facility in the process of regulating her period, we are interested in whether she went back to a health facility on a separate occasion to treat complications that she may have experienced.*	((${self_reg_year} > ${self_abt_year}) or (${self_abt_year} = '')) and ((${self_reg_yn} = 'yes'))<br>◯ Beeni<br>◯ Beeko<br>◯ N ko mo<br>◯ ko si esi
732b. Nje e so fun enikeni ninu awon wonyi nipa iriri yin?<br>*Read the answer choices aloud. Select all that apply.*	((${self_reg_year} > ${self_abt_year}) or (${self_abt_year} = '')) and ((${self_reg_yn} = 'yes'))<br>☐ Oko/Ore kunrin<br>☐ Sister<br>☐ Egbon Okunrin<br>☐ Iya<br>☐ Baba<br>☐ Ara Ile miran<br>☐ Ore Kiini: ${friend1_name}<br>☐ Ore Keji: ${friend2_name}<br>☐ Ore Miran<br>☐ Omiran<br>☐ N ko mo<br>☐ ko si esi<br>(${friend1_name} != '' and ${friend1_name} != '-99' and filter = 'friend1') or (${friend2_name} != '' and ${friend2_name} != '-99' and filter = 'friend2') or (filter = 'always')

	${consent_obtained}
Bayi afe bere awon ibeere gbogbo gbo lori oyun sise ati bi awon obinrin ti won ti se oyun tabi yo oyun se woo. Ejowo e so bi e se roo bi awon eyi: mo fara mo gan, mo fara mo, mo fara mo mi otun fara mo, mi o fara mo, mi o fara mo rara.<br>*Check box to confirm scrolled to bottom.*	
Danna OK a ci gaba	◯ odaa

733. O wa ni bamu fun awon obinrin lati se oyun tabi yo oyun ti o ba lewu fun emi won	${consent_obtained}<br>◯ Mo fara mo gan ni<br>◯ Mo fara mo<br>◯ Mo da duro<br>◯ Mi o fara mo<br>◯ Mi o fara mo gan ni<br>◯ ko si esi

734. O wa ni bamu fun awon obinrin lati se oyun tabi yo oyun ti won ba fi pa ba won lopo.	${consent_obtained}<br>Mo fara mo gan ni<br>Mo fara mo<br>Mo da duro<br>Mi o fara mo<br>Mi o fara mo gan ni<br>ko si esi
735. O wa ni bamu ki obinrin se oyun tabi yo oyun ti won ko ba le to omo miran	${consent_obtained}<br>Mo fara mo gan ni<br>Mo fara mo<br>Mo da duro<br>Mi o fara mo<br>Mi o fara mo gan ni<br>ko si esi
736. O wa ni bamu ki obinrin se oyun tabi yo oyun ti won ko ba fe bimo miran mo	${consent_obtained}<br>Mo fara mo gan ni<br>Mo fara mo<br>Mo da duro<br>Mi o fara mo<br>Mi o fara mo gan ni<br>ko si esi
737. Obinrin to se yun n mu itu ba ebi	${consent_obtained}<br>Mo fara mo gan ni<br>Mo fara mo<br>Mo da duro<br>Mi o fara mo<br>Mi o fara mo gan ni<br>ko si esi
738. Obinrin to ti se yun ko gbodo so fun enikeni	${consent_obtained}<br>Mo fara mo gan ni<br>Mo fara mo<br>Mo da duro<br>Mi o fara mo<br>Mi o fara mo gan ni<br>ko si esi

Gbigba iyanda lati pe

FLW_801. Ese fun akoko yin ti egba wa. A fe gbo gbo lati enu yin si lori iriri yin lori oyun sise pelu awon ipinnu ti e se lori awon ohun lati lo ati ibi ti o ye ki eyan lo lati se oyun ati bebe lo. Nje e le gba obinrin miran laye ninu awon ti o kopa ninu iwadi yi ni ojo iwaju lati le fi iriri han?	(${self_abt_yn} = 'yes') or (${self_reg_yn} = 'yes')<br>Beeni<br>Beeko<br>ko si esi
FLW_802. Nje eni ero ibani soro?	${flw_willing} = 'yes'<br>Beeni<br>Beeko<br>ko si esi
FLW_803. Se mo le gba nomba ero ibani soro yin nitori ti aba fe peyin lojo iwaju lati bayin soro?<br>*Enter an 11-digit number without the country code. Do not include spaces or dashes. Enter 0 for no response.*	${flw_number_yn} = 'yes'<br>--
FLW_804. Nje ele tun nomba na pe:<br>*Enter an 11-digit number without the country code. Do not include spaces or dashes. Enter 0 for no response.*	(${flw_number_typed} != '') and (${flw_number_typed} != '0')<br>--
Ki oludahun si ibeere fun akoko ti o fi sile fun yin<br>*The respondent is finished, but there are still more questions for you to complete outside the home.*	${available} = 'yes'

Agbegbe ati esi ibeere

095. Agbegbe<br>*Take a GPS point near the entrance to the household. Record location when the accuracy is smaller than 6 m.*	gbogbo gba
096. Igba melo ni o ti wa si idile yii lati wa se iforojomitoro pelu arabirin yii ti o je oludahun ibeere?	gbogbo gba<br>○ Igba akoko<br>○ Igba keji<br>○ Igba keta
097. Ede wo lo fi se iwadi yii?	009a = 1<br>○ Ede oyinbo<br>○ Hausa<br>○ Igbo<br>○ Yoruba<br>○ Pidgin<br>○ Omiran
