# Supplementary material for: Measurement of abortion safety using community-based surveys: Findings from three countries
Source: PLoS One. 2019 Nov 7;14(11):e0223146. doi: 10.1371/journal.pone.0223146 (PMC6837422; doi:10.1371/journal.pone.0223146)
Supplement: S6 Doc — (PDF) [file pone.0223146.s006.pdf]

S6 Doc.

CIR2-Female-Questionnaire-v6-jkp.xlsx

001a. Are you in the correct household? EA: [EA entered in the Household Questionnaire] Structure #: [Structure entered in the Household Questionnaire] Household #: [Household entered in the Household Questionnaire]	Always<br>○ Yes<br>○ No
002. Enter your ID below.<br>*Please record your ID*	002 = 0<br>____________________
003b. Record the correct date and time.	003 = 0<br>Day: ____________<br>Month: ____________<br>Year: ____________

The following information is from the Household Questionnaire. Please review to make sure you are interviewing the correct respondent. [ODK will display the LIST OF GEOGRAPHIES ENTERED, Enumeration Area, Structure Number, and Household Number entered into the Household Questionnaire linked to this Female Questionnaire.] Is the above information correct?	Always
Region: ${level1_unlinked}	____________________
Department: ${level2_unlinked}	____________________
Sub-prefecture: ${level3_unlinked}	____________________
Enumeration Area: [EA]	____________________
Structure number: [#]	____________________
Household number: [#]	____________________
004b. Is the above information correct?	004 = 0<br>○ Yes<br>○ No

005. CHECK: You should be attempting to interview [Name of the interviewee]. Is that correct?<br>*If misspelled, select “yes” and update the name in question “011.” If this is the wrong person, you have two options: (1) exit and ignore changes to this form. Open the correct form.*<br>*Or (2) find and interview the person whose name appears above.*	Always<br>○ Yes<br>○ No
006. Is the respondent present and available to be interviewed today?	Always<br>○ Yes<br>○ No
007. How well acquainted are you with the respondent?	006 = 1<br>○ Very well acquainted<br>○ Well acquainted<br>○ Not well acquainted<br>○ Not acquainted
008. Has the respondent previously participated in PMA 2020 surveys?	006 = 1<br>○ Yes<br>○ No<br>○ Do not know<br>○ No response
INFORMED CONSENT<br>*Find the woman between the age of 15-49 associated with this Female Respondent Questionnaire. The interview must have auditory privacy. Read the following greeting*	(${available} = 'yes') and (not(${unlinked}) or ${proceed_with_unlinked})
Bonjour. Je m’appelle ________________________________ et je travaille pour l’Institut National de la Statistique de Côte d’Ivoire (INS-Côte d'Ivoire). Nous menons une enquête locale auprès des femmes sur plusieurs questions de santé reproductive, dont les connaissances, les attitudes et l’utilisation de la contraception et de l’avortement. Certaines de ces questions, particulièrement celles portant sur	(${available} = 'yes') and (not(${unlinked}) or ${proceed_with_unlinked})

l'avortement, ont été ajoutées expressément à des fins de recherche. Nous apprécierons grandement votre participation à cette étude. Ces informations nous aideront à informer le gouvernement afin d'offrir de meilleurs services de santé. L'enquête dure généralement entre 30 et 40 minutes. Toute information que vous voudrez bien nous communiquer sera strictement confidentielle, et seules les données rendues complètement anonymes seront utilisées pour mener des analyses, en présenter les résultats et partager les données.

La participation à cette enquête, à la fois l'enquête standard et les questions de recherche, est entièrement volontaire. Si nous rencontrons une question à laquelle vous ne souhaitez pas répondre, faites-le moi savoir et je passerai à la suivante. Vous êtes libre de ne pas participer à cette enquête du tout, ou de l'interrompre à tout moment. Cependant, nous espérons que vous voudrez bien y participer car votre opinion est très importante.

Si vous avez des questions sur cette étude et sur vos droits en tant que participant à une recherche, vous pouvez me les poser ou contacter M. Aka Doré à l'Institut National de la Statistique de Côte d'Ivoire (INS-Côte d'Ivoire) au +225 09 34 14 94. Jusqu'ici, avez-vous des questions sur l'enquête ?

009a. Provide a paper copy of the Consent Form to the respondent and explain it. Then, ask: May I begin the interview now?	006 = 1<br>○ Yes<br>○ No

	(${begin_interview}='yes')
009b. Respondent's signature<br>*Please ask the respondent to sign or check the box in agreement of their participation.*	009a = 1<br>--

010. Interviewer's ID Please record your ID as a witness to the consent process. You previously entered "[Interviewer's ID]."	${consent_obtained} and (${your_name_check} = 'no')<br>--

Section 1 – Respondent's Background, Marital Status, Household characteristics

Now I would like to ask about your background and socioeconomic conditions.

	${consent_obtained}
101. In what month and year were you born? The age in the household roster is [AGE]	009a = 1
101. In what month and year were you born?	009a = 1
Month:	○ January<br>○ February<br>○ March<br>○ April<br>○ May<br>○ June<br>○ July<br>○ August<br>○ September<br>○ October<br>○ November<br>○ December<br>○ Do not know
Year:	Year: ----------------------------------

102. How old were you at your last birthday?	009a = 1<br>--
103. What is the highest level of school you attended?<br>*Only record formal schooling. Do not record bible or koranic school or short courses.*	009a = 1<br>○ Never attended<br>○ Primary<br>○ Secondary<br>○ Tertiary

	○ No response
PNSME_101. What is your religion?<br>*Select your currently practiced religion*	${consent_obtained}<br>○ Muslim<br>○ Catholic<br>○ Methodist<br>○ Evangelica<br>○ Other Christian Religion<br>○ Animast<br>○ Other religion<br>○ No religion<br>○ No response
PNSME_102. What ethnic group do you belong to?	${consent_obtained}<br>○ Akan<br>○ Mandé du sud<br>○ Mandé du nord<br>○ Gur<br>○ Krou<br>○ Other Ivorian<br>○ Other non-Ivorian<br>○ No response
104. Are you currently married or living together with a man as if married?<br>*Probe: If no, ask whether the respondent is divorced, separated, or widowed.*	009a = 1<br>○ Yes, currently married<br>○ Yes, living with a man<br>○ Not currently in union: Divorced / separated<br>○ Not currently in union: Widow<br>○ No, never in union<br>○ No response
105. Have you been married or lived with a man only once or more than once?	104 ≠ 5<br>○ Only once<br>○ More than once<br>○ No response

	(${marriage_history} = 'more_than_once')
106a. In what month and year did you start living with your FIRST husband / partner?<br>*Select 'Do not know' for month and '2020' for year to indicate 'No Response'.*	105=2
Month:	○ January<br>○ February<br>○ March<br>○ April<br>○ May<br>○ June<br>○ July<br>○ August<br>○ September<br>○ October<br>○ November<br>○ December<br>○ Do not know
Year:	Year: ________________

106b. CHECK: Based on the response you entered in 106a, the respondent was possibly 15 years old or younger at the time of her first marriage.<br>Did you enter 106a correctly?	106a age at marriage ≤15<br>○ Yes<br>○ No

	(${marriage_history} = 'once') or (${marriage_history} = 'more_than_once')
107a. Now I would like to ask about when you started living with your CURRENT or MOST RECENT husband / partner. In what month and year was that?	105 = 1 or 2

Select 'Do not know' for month and '2020' for year to indicate 'No Response'.	
Month:	○ January<br>○ February<br>○ March<br>○ April<br>○ May<br>○ June<br>○ July<br>○ August<br>○ September<br>○ October<br>○ November<br>○ December<br>○ Do not know
Year:	Year: ________________

107b. CHECK: Based on the response you entered in 107a, the respondent was possibly 15 years old or younger at the time of her current or most recent marriage.<br>Did you enter 107a correctly?	107a age at marriage ≤15<br>○ Yes<br>○ No
108. Does your husband / partner have other wives or does he live with other women as if married?	104 = 1 or 2<br>○ Yes<br>○ No<br>○ Do not know<br>○ No response
PNSME_103. What is the age of your husband / partner?<br>*Enter -88 for Do not know and -99 for No response.*	((${marital_status} = 'currently_married') or (${marital_status} = 'currently_living_with_man'))<br>________________
PNSME_104. What is the highest level of education achieved by your husband / spouse?	((${marital_status} = 'currently_married') or (${marital_status} = 'currently_living_with_man'))<br>○ Never attended<br>○ Primary<br>○ Secondary<br>○ Tertiary<br>○ No response

Section 2 – Reproduction, Pregnancy & Fertility Preferences

Now I would like to ask about all the births you have had during your life.

200. Have you ever given birth?	009a = 1<br>○ Yes<br>○ No<br>○ No response
201. How many times have you given birth?<br>*Enter -99 for no response.*	200 = 1<br>________________

	(${birth_events} > 1)
205. When was your FIRST birth?<br>*Please record the date of the FIRST birth. The date should be found by calculating backwards from memorable events if needed.*<br>*Select 'Do not know' for month and '2020' for year to indicate 'No Response'.*	201 > 1
Month:	○ January<br>○ February<br>○ March<br>○ April<br>○ May<br>○ June<br>○ July

	◯ August<br>◯ September<br>◯ October<br>◯ November<br>◯ December<br>◯ Do not know
Year:	Year: ________________

	(${birth_events} > 0)
206. When was your MOST RECENT birth?<br>*Select 'Do not know' for month and '2020' for year to indicate 'No Response'.*	201 > 1
Month:	◯ January<br>◯ February<br>◯ March<br>◯ April<br>◯ May<br>◯ June<br>◯ July<br>◯ August<br>◯ September<br>◯ October<br>◯ November<br>◯ December<br>◯ Do not know
Year:	Year: ________________

210a. Are you pregnant now?	009a = 1<br>◯ Yes<br>◯ No<br>◯ Unsure<br>◯ No response

	${pregnant} = 'yes'
210b. How many months pregnant are you?	210a = 1
The most recent birth was: [Date of most recent birth]	${recent_birth} != ''
#####<br>*Please record the number of completed months. Enter -88 for do not know, -99 for No response.*	________________

209. When did your last menstrual period start?<br>*If you select days, weeks, months, or years, you will enter a number for X on the next screen. Enter 0 days for today, not 0 weeks/months/years.*	009a = 1<br>◯ X days ago<br>◯ X weeks ago<br>◯ X months ago<br>◯ X years ago<br>◯ Menopausal / Hysterectomy<br>◯ Before last birth<br>◯ Never menstruated<br>◯ No response
209a. Enter [days / weeks / months / years]<br>*Enter 0 days for today, not 0 weeks/months/years.*	(${menstrual_period} = 'days') or (${menstrual_period} = 'weeks') or (${menstrual_period} = 'month …<br>________________

	${ever_birth} = 'yes' or ${pregnant} = 'yes'
213a. Now I would like to ask a question about your last birth.	201 > 0 AND 210a ≠ 1 201 > 0 AND 210a ≠ 1 201 > 0 AND 210a ≠ 1
213b. Now I would like to ask a question about your current pregnancy.	210a = 1
At the time you became pregnant, did you want to become pregnant then, did you want to wait until later, or did you not want to have any more children at all?	(201 > 1 AND 210a ≠ 1) OR (200 = 1 AND 201a = 1)
At the time you became pregnant, did you want to become pregnant then, did you want to wait until later, or did you not want to have any children at all?	(201 = 1 AND 210a ≠ 1) OR (200 = 2 AND 201a = 1)

#####	◯ Then<br>◯ Later<br>◯ Not at all<br>◯ No response

Now I have some questions about the future.	${consent_obtained}
211a. Would you like to have a child or would you prefer not to have any children?	210a ≠ 1<br>◯ Have a child<br>◯ Prefer no children<br>◯ Says she can't get pregnant<br>◯ Undecided / Do not know<br>◯ No response
211a. Would you like to have another child or would you prefer not to have any more children?	210a ≠ 1<br>◯ Have another child<br>◯ No more<br>◯ Says she can't get pregnant<br>◯ Undecided / Do not know<br>◯ No response
211b. After the child you are expecting now, would you like to have another child or would you prefer not to have any more children?	210a = 1<br>◯ Have another child<br>◯ No more<br>◯ Says she can't get pregnant<br>◯ Undecided / Do not know<br>◯ No response
212a. How long would you like to wait from now before the birth of a child?<br>*If you select months or years, you will enter a number for X on the next screen.*<br>*Select "Years" if more than 36 months.*<br>*Please check that you correctly entered the value for months/years.*	211a = 1<br>◯ X months<br>◯ X years<br>◯ Soon/now<br>◯ Says she can't get pregnant<br>◯ Other<br>◯ Do not know<br>◯ No response
212b. After the birth of the child you are expecting now, how long would you like to wait before the birth of another child?<br>*If you select months or years, you will enter a number for X on the next screen.*<br>*Select "Years" if more than 36 months.*<br>*Please check that you correctly entered the value for months/years.*	211b = 1<br>◯ X months<br>◯ X years<br>◯ Soon/now<br>◯ Says she can't get pregnant<br>◯ Other<br>◯ Do not know<br>◯ No response
212c. Enter the number of [Months OR Years] you would like to wait:	212a OR 212b = 'months' OR 'year'<br>--

Section 3 – Contraception

Now I would like to talk about family planning - the various ways or methods that a couple can use to delay or avoid a pregnancy.

An image will appear on the screen for some methods. If the respondent says that she has not heard of the method or if she hesitates to answer, read the probe aloud and show her the image, if available.

301a. Have you ever heard of female sterilization?<br>PROBE: Women can have an operation to avoid having any more children.	009a = 1<br>◯ Yes<br>◯ No<br>◯ No response
301b. Have you ever heard of male sterilization?<br>PROBE: Men can have an operation to avoid having any more children.	009a = 1<br>◯ Yes<br>◯ No<br>◯ No response
301c. Have you ever heard of the contraceptive implant?	009a = 1

PROBE: Women can have one or several small rods placed in her upper arm by a doctor or nurse, which can prevent pregnancy for one or more years.<br>[implant_150x300.png]	○ Yes<br>○ No<br>○ No response
301d. Have you ever heard of the IUD?<br>PROBE: Women can have a loop or coil placed inside them by a doctor or a nurse.<br>[IUD_150x300.png]	009a = 1<br>○ Yes<br>○ No<br>○ No response
301e. Have you ever heard of injectables?<br>PROBE: Women can have an injection by a health provider that stops them from becoming pregnant for one or more months.<br>[sayana_depo_150x300.jpg]	009a = 1<br>○ Yes<br>○ No<br>○ No response
301f. Have you ever heard of the (birth control) pill?<br>PROBE: Women can take a pill every day to avoid becoming pregnant.<br>[pill_150x300.png]	009a = 1<br>○ Yes<br>○ No<br>○ No response
301g. Have you ever heard of emergency contraception?<br>PROBE: As an emergency measure after unprotected sexual intercourse women can take special pills at any time within three to five days to prevent pregnancy.	009a = 1<br>○ Yes<br>○ No<br>○ No response
301h. Have you ever heard of condoms?<br>PROBE: Men can put a rubber sheath on their penis before sexual intercourse.<br>[male_condom_150x300.png]	009a = 1<br>○ Yes<br>○ No<br>○ No response
301i. Have you ever heard of female condoms?<br>PROBE: Women can put a sheath in their vagina before sexual intercourse.<br>[female_condom_150x300.png]	009a = 1<br>○ Yes<br>○ No<br>○ No response
301j. Have you ever heard of the diaphragm?<br>PROBE: Women can place a thin flexible disk in their vagina before sexual intercourse.<br>[diaphragm_150x300.png]	009a = 1<br>○ Yes<br>○ No<br>○ No response
301k. Have you ever heard of foam or jelly as a contraceptive method?<br>PROBE: Women can place a suppository, jelly, or cream in their vagina before sexual intercourse to prevent pregnancy.<br>[spermicide_150x300.png]	009a = 1<br>○ Yes<br>○ No<br>○ No response
301m. Have you ever heard of the Lactational Amenorrhea Method or LAM?	009a = 1<br>○ Yes<br>○ No<br>○ No response
301n. Have you ever heard of the rhythm method?<br>PROBE: Women can avoid pregnancy by not having sexual intercourse on the days of the month they think they can get pregnant.	009a = 1<br>○ Yes<br>○ No<br>○ No response
301o. Have you ever heard of the withdrawal method?<br>PROBE: Men can be careful and pull out before climax.	009a = 1<br>○ Yes<br>○ No<br>○ No response
301p. Have you ever heard of any other ways or methods that women or men can use to avoid pregnancy?	009a = 1<br>○ Yes<br>○ No<br>○ No response
302a. Are you or your partner currently doing something or using any method to delay or avoid getting pregnant?	210a ≠ 1 AND 009a = 1 210210a ≠ 1 AND 009a = 1 210a ≠ 1 AND 009a = 1 ≠ 1 AND 009a = 1 210a ≠ 1 AND 009a = 1<br>○ Yes<br>○ No<br>○ No response

	${current_user} = 'yes'
302b. Which method or methods are you using?	302a = 1

PROBE: Anything else?<br>*Select all methods mentioned. SCROLL TO THE BOTTOM to see all choices.*	☐ Female sterilization<br>☐ Male sterilization<br>☐ Implant<br>☐ IUD<br>☐ Injectables<br>☐ Pill<br>☐ Emergency Contraception<br>☐ Male condom<br>☐ Female condom<br>☐ Diaphragm<br>☐ Foam/Jelly<br>☐ Standard Days/Cycle beads<br>☐ LAM<br>☐ Rhythm method<br>☐ Withdrawal<br>☐ Other traditional method<br>☐ No response

CALC_CM. CALCULATE: CURRENT METHOD<br>THIS WILL NOT APPEAR ON THE SCREEN<br>*ODK will identify the most effective method currently being used by the respondent by selecting the highest method in the choice list.*	302a=1 AND 302b ≠-99<br>○ Female sterilization<br>○ Male sterilization<br>○ Implant<br>○ IUD<br>○ Injectables<br>○ Pill<br>○ Emergency Contraception<br>○ Male condom<br>○ Female condom<br>○ Diaphragm<br>○ Foam/Jelly<br>○ Standard Days/Cycle beads<br>○ LAM<br>○ Rhythm method<br>○ Withdrawal<br>○ Other traditional method<br>○ No response
LCL_301. PROBE: Was the injection administered via syringe or small needle?<br>*Show the image to the respondent.*<br>[sayana_depo_150x300.jpg]	CALC CM = 5<br>○ Syringe<br>○ Small needle (Sayana Press)<br>○ No Response
302c. Does your husband/partner know that you are using [CURRENT METHOD]?	302a = 1<br>○ Yes<br>○ No<br>○ No response
302c. Does your husband/partner know that you are using family planning?	302a = -99<br>○ Yes<br>○ No<br>○ No response
305a. You said that you are not currently using a contraceptive method. Do you think you will use a contraceptive method to delay or avoid getting pregnant at any time in the future?	302a ≠1 AND 210a ≠1<br>○ Yes<br>○ No<br>○ No response
305b. Do you think you will use a contraceptive method to delay or avoid getting pregnant at any time in the future?	302a ≠1 AND 201a = 1<br>○ Yes<br>○ No<br>○ No response
306a. In the last 12 months, have you ever done something or used a method to delay or avoid getting pregnant?	302a ≠1<br>○ Yes<br>○ No<br>○ No response

	${recent_user} = 'yes'

306b. Which method did you use most recently?<br>PROBE: Anything else?<br>*Select most effective method (highest method in list). Scroll to bottom to see all choices.*	306a = 1<br>◯ Male sterilization<br>◯ Implant<br>◯ IUD<br>◯ Injectables<br>◯ Pill<br>◯ Emergency Contraception<br>◯ Male condom<br>◯ Female condom<br>◯ Diaphragm<br>◯ Foam/Jelly<br>◯ Standard Days/Cycle beads<br>◯ LAM<br>◯ Rhythm method<br>◯ Withdrawal<br>◯ Other traditional method<br>◯ No response

LCL_302. PROBE: Was the injection administered via syringe or small needle?<br>*Show the image to the respondent.*<br>[sayana_depo_150x300.jpg]	306b = 5<br>◯ Syringe<br>◯ Small needle (Sayana Press)<br>◯ No Response
307. Before you started using [CURRENT METHOD / MOST RECENT METHOD], had you discussed the decision to delay or avoid pregnancy with your husband/partner?	302a = 1 OR 306a = 1<br>◯ Yes<br>◯ No<br>◯ Do not know<br>◯ No response
308. Would you say that using contraception is mainly your decision, mainly your husband/partner's decision or did you both decide together?	302a = 1<br>◯ Mainly respondent<br>◯ Mainly husband/partner<br>◯ Joint decision<br>◯ Other<br>◯ No response
308a. The last time you received your [CURREN METHOD /MOST RECENT METHOD], how much did you have to pay out of pocket, including any fees paid for the method, supplies or services, and transportation?<br>*Enter all prices in FCFA. Zero is a possible answer. Enter -88 if respondent does not know, -99 for no response.*	302a = 1 OR 306a = 1<br>_______________

	${current_user} = 'yes'
309a. Since what month and year have you been using [CURRENT METHOD / MOST RECENT METHOD] without stopping?<br>*Calculate backwards from memorable events if needed.*	302a = 1
Most Recent Birth: [mm-yyyy]	${recent_birth} != ''
Current Marriage: [mm-yyyy]	${husband_cohabit_start_recent} != ''
Month:	◯ January<br>◯ February<br>◯ March<br>◯ April<br>◯ May<br>◯ June<br>◯ July<br>◯ August<br>◯ September<br>◯ October<br>◯ November<br>◯ December<br>◯ Do not know
Year:	Year: _______________

	${recent_user} = 'yes'

309b. When did you stop using [CURRENT METHOD / MOST RECENT METHOD]?<br>*Please record the date. The date should be found by calculating backwards from memorable events if needed.*<br>*Select 'Do not know' for month and '2020' for year to indicate No Response.*	306a = 1
Month:	○ January<br>○ February<br>○ March<br>○ April<br>○ May<br>○ June<br>○ July<br>○ August<br>○ September<br>○ October<br>○ November<br>○ December<br>○ Do not know
Year:	Year: ________________

	${recent_user} = 'yes'
309c. In what month and year had you started using [CURRENT METHOD / MOST RECENT METHOD] before stopping?<br>*Calculate backwards from memorable events if needed.*<br>*Select 'Do not know' for month and '2020' for year to indicate No Response.*	306a = 1
Most Recent Birth: [mm-yyyy]	${recent_birth} != ''
Current Marriage: [mm-yyyy]	${husband_cohabit_start_recent} != ''
Month:	○ January<br>○ February<br>○ March<br>○ April<br>○ May<br>○ June<br>○ July<br>○ August<br>○ September<br>○ October<br>○ November<br>○ December<br>○ Do not know
Year:	Year: ________________

309d. CHECK: Just to make sure I have this correct, you used [CURRENT METHOD / MOST RECENT METHOD] continuously between [START DATE] and [END DATE] without stopping, is that correct?	306a = 1<br>○ Yes<br>○ No
GO BACK TO THE PREVIOUS SCREEN AND PROBE TO DETERMINE THE PERIOD OF MOST RECENT CONTINUOUS USE.<br>*Suggested probes: - When was the last time you used [METHOD]? - How long had you been using [METHOD] without stopping*	309d = 0
310. Why did you stop using [CURRENT METHOD / MOST RECENT METHOD?	306a = 1<br>☐ Infrequent sex / husband away<br>☐ Became pregnant while using<br>☐ Wanted to become pregnant<br>☐ Husband / partner disapproved<br>☐ Wanted a more effective method<br>☐ No method available<br>☐ Health concerns<br>☐ Fear of side effects

	☐ Lack of access / too far<br>☐ Costs too much<br>☐ Inconvenient to use<br>☐ Fatalistic<br>☐ Difficult to get pregnant / menopausal<br>☐ Interferes with body's processes<br>☐ Other<br>☐ Do not know<br>☐ No response

	${current_or_recent_user} and (${current_recent_method} != 'LAM') and (${current_recent_method} != …
311a. You first started using [CURRENT METHOD / MOST RECENT METHOD] on [DATE FROM FQ309a OR 309c] Where did you or your partner get it at that time?<br>*Scroll to bottom to see all choices.*	(CALC_CM ≠ 14, 30, 31, 39, -99) OR (306b ≠ 14, 30, 31, 39, -99)<br>○ Government hospital<br>○ Government health center<br>○ Family planning clinic<br>○ Mobile clinic (public)<br>○ Other public<br>○ Private hospital / clinic<br>○ Pharmacy<br>○ Private doctor<br>○ Mobile clinic (private)<br>○ Health agent<br>○ Other private<br>○ Store<br>○ Religious organizations<br>○ Community event<br>○ Friend / parent<br>○ Community health agent<br>○ Street vendor<br>○ Other<br>○ Do not know<br>○ No response
311a. Where did you or your partner get using ${current_recent_label} when you first started using it?<br>*Scroll to bottom to see all choices.*	(CALC_CM ≠ 14, 30, 31, 39, -99) OR (306b ≠ 14, 30, 31, 39, -99)<br>○ Government hospital<br>○ Government health center<br>○ Family planning clinic<br>○ Mobile clinic (public)<br>○ Other public<br>○ Private hospital / clinic<br>○ Pharmacy<br>○ Private doctor<br>○ Mobile clinic (private)<br>○ Health agent<br>○ Other private<br>○ Store<br>○ Religious organizations<br>○ Community event<br>○ Friend / parent<br>○ Community health agent<br>○ Street vendor<br>○ Other<br>○ Do not know<br>○ No response

312a. When you obtained your [CURRENT METHOD / MOST RECENT METHOD], were you told by the provider about side effects or problems you might have with a method to delay or avoid getting pregnant?	311a ≠ .<br>○ Yes<br>○ No<br>○ No response
	312a = 1

312b. Were you told what to do if you experienced side effects or problems?	○ Yes<br>○ No<br>○ No response
313. At that time, were you told by the family planning provider about methods of family planning other than the [CURRENT METHOD / MOST RECENT METHOD] that you could use?	311a ≠ . OR 311b ≠ .<br>○ Yes<br>○ No<br>○ Do not know<br>○ No response
314a. During that visit, did you obtain the method you wanted to delay or avoid getting pregnant?	311a ≠ .<br>○ Yes<br>○ No<br>○ No response
314c. Why didn't you obtain the method you wanted?	314a = 0<br>○ Method out of stock that day<br>○ Method not available at all<br>○ Provider not trained to provide the method<br>○ Provider recommended a different method<br>○ Not eligible for method<br>○ Decided not to adopt a method<br>○ Too costly<br>○ Other<br>○ No response
315a. During that visit, who made the final decision about what method you got?	311a ≠ .<br>○ You alone<br>○ Provider<br>○ Partner<br>○ You and provider<br>○ You and partner<br>○ Other<br>○ Do not know<br>○ No response
315b. Who made the final decision to use rhythm?	311b ≠ .<br>○ You alone<br>○ Provider<br>○ Partner<br>○ You and provider<br>○ You and partner<br>○ Other<br>○ Do not know<br>○ No response
315b. Who made the final decision to use LAM?	311b ≠ .<br>○ You alone<br>○ Provider<br>○ Partner<br>○ You and provider<br>○ You and partner<br>○ Other<br>○ Do not know<br>○ No response
316. Would you return to this provider? Provider: [Type of provider selected in 311a or 311b]	311a ≠ 35 or 96<br>○ Yes<br>○ No<br>○ Do not know<br>○ No response
317. Would you refer your relative or friend to this provider / facility? Provider: [Type of provider selected in 311a or 311b]	311 a ≠ 34 or 96<br>○ Yes<br>○ No<br>○ Do not know

	○ No response	
SW_1a. Right before you started using [CURRENT METHOD / MOST RECENT METHOD] in [MOIS/ANNEE], were you doing something or using any method to delay or avoid getting pregnant?	302a = 1 OR 306a = 1<br>○ Yes<br>○ No<br>○ No response	
SW_1b. Which method were you using?	SW_1a = 1<br>○ Male sterilization<br>○ Implant<br>○ IUD<br>○ Injectables<br>○ Pill<br>○ Emergency Contraception<br>○ Male condom<br>○ Female condom<br>○ Diaphragm<br>○ Foam/Jelly<br>○ Standard Days/Cycle beads<br>○ LAM<br>○ Rhythm method<br>○ Withdrawal<br>○ Other traditional method<br>○ No response	
PP_1. Since the birth of your child in [DATE OF MOST RECENT BIRTH], have you ever done something or used any method to delay or avoid getting pregnant?	child born in last 2 years AND 302a ≠ 1<br>○ Yes<br>○ No<br>○ No response	
PP_2. How long after the birth in [DATE OF MOST RECENT BIRTH] did you start doing something or start using a method?<br>*Enter 0 days for today. You will enter a number for X on the next screen.*	PP_1 = 1 OR (302a = 1 AND child born in the last 2 years)<br>○ X days after<br>○ X weeks after<br>○ X months after<br>○ X years after<br>○ No response	
PP_2. Enter [METHOD].<br>*If today, enter zero days only, not zero weeks/months/years.*	${pp_method_units} = 'days' or ${pp_method_units} = 'weeks' or ${pp_method_units} = 'months' or ${ …<br>--	
PP_3. What was the method?	PP_2 ≠ .<br>○ Female sterilization<br>○ Male sterilization<br>○ Implant<br>○ IUD<br>○ Injectables<br>○ Pill<br>○ Emergency Contraception<br>○ Male condom<br>○ Female condom<br>○ Diaphragm<br>○ Foam/Jelly<br>○ Standard Days/Cycle beads<br>○ LAM<br>○ Rhythm method<br>○ Withdrawal<br>○ Other traditional method<br>○ No response	
LCL_PP_302. PROBE: Was the injection administered via syringe or small needle?<br>*Show the image to the respondent.*<br>[sayana_depo_150x300.jpg]	PP_3 = 5<br>○ Syringe<br>○ Small needle (Sayana Press)<br>○ No Response	
319. Have you ever done anything or tried in any way to delay or avoid getting pregnant?	306a ≠ 1 OR 302a ≠ 1<br>○ Yes	

	○ No<br>○ No response
320. How old were you when you first used a method to delay or avoid getting pregnant? The respondent said she was [AGE] years old at her last birthday.<br>*Enter the age in years. Enter -88 if the respondent does not know. Enter -99 if there is no response. Cannot be younger than 9.*	302a = 1 OR 306a =1 OR 319 = 1<br>________________
321. How many living children did you have at that time, if any? Note: the respondent said that she gave birth [NUMBER OF LIFE BIRTHS] times in 201.<br>*Enter -99 for no response.*	Age in 320 ≥ 9 AND 200 = 1<br>________________

	${fp_ever_used} = 'yes'
322. Which method did you first use to delay or avoid getting pregnant?<br>*Do not read the method choices. Scroll to bottom to see all choices.*	319 = 1<br>○ Female sterilization<br>○ Male sterilization<br>○ Implant<br>○ IUD<br>○ Injectables<br>○ Pill<br>○ Emergency Contraception<br>○ Male condom<br>○ Female condom<br>○ Diaphragm<br>○ Foam/Jelly<br>○ Standard Days/Cycle beads<br>○ LAM<br>○ Rhythm method<br>○ Withdrawal<br>○ Other traditional method<br>○ No response

LCL_322a. PROBE: Was the injection administered via syringe or small needle?<br>*Show the image to the respondent.*<br>[sayana_depo_150x300.jpg]	322 = 5<br>○ Syringe<br>○ Small needle (Sayana Press)<br>○ No Response
322a. Have you used emergency contraception at any time in the last 12 months?<br>PROBE: As an emergency measure after unprotected sexual intercourse women can take special pills at any time within three to five days to prevent pregnancy.	302b ≠ emergency contraception OR 306b ≠ 8<br>○ Yes<br>○ No<br>○ No response

	((${current_user} = 'no')) and ((${more_children_none} = 'no_children') or …
323a. You said that you do not want a child soon and that you are not using a method to avoid pregnancy.	302a = 0 AND ((212a or 212b > 2 years) OR (211a or 211b = 2))
323a. You said that you do not want another child soon and that you are not using a method to avoid pregnancy.	302a = 0 AND ((212a or 212b > 2 years) OR (211a or 211b = 2))
323a. You said that you do not want any children and that you are not using a method to avoid pregnancy.	302a = 0 AND ((212a or 212b > 2 years) OR (211a or 211b = 2))
323a. You said that you do not want any more children and that you are not using a method to avoid pregnancy.	302a = 0 AND ((212a or 212b > 2 years) OR (211a or 211b = 2))
Can you tell me why you are not using a method to prevent pregnancy?<br>PROBE: Any other reason?<br>*RECORD ALL REASONS MENTIONED Cannot select "Not Married" if 104 is "Yes, currently married".*<br>*Scroll to bottom to see all choices.*	☐ Not married<br>☐ Infrequent sex / Not having sex<br>☐ Menopausal / Hysterectomy<br>☐ Subfecund / Infecund<br>☐ Not menstruated since last birth<br>☐ Breastfeeding<br>☐ Husband away for multiple days<br>☐ Up to God / fatalistic<br>☐ Respondent opposed<br>☐ Husband / partner opposed<br>☐ Others opposed<br>☐ Religious prohibition<br>☐ Knows no method

	☐ Knows no source<br>☐ Fear of side effects<br>☐ Health concerns<br>☐ Lack of access / too far<br>☐ Costs too much<br>☐ Preferred method not available<br>☐ No method available<br>☐ Inconvenient to use<br>☐ Interferes with body's processes<br>☐ Other<br>☐ Do not know<br>☐ No response

323b. Would you say that not using contraception is mainly your decision, mainly your husband/partner's decision or did you both decide together?	302a ≠ 1<br>○ Mainly respondent<br>○ Mainly husband/partner<br>○ Joint decision<br>○ Other<br>○ No response
324. In the last 12 months, were you visited by a community health worker who talked to you about family planning?	009a = 1<br>○ Yes<br>○ No<br>○ No response
325a. In the last 12 months, have you visited a health facility or camp for care for yourself?<br>*For any health services.*	009a = 1<br>○ Yes<br>○ No<br>○ No response
325a. In the last 12 months, have you visited a health facility or camp for care for yourself or your children?<br>*For any health services.*	009a = 1<br>○ Yes<br>○ No<br>○ No response
325b. Did any staff member at the health facility speak to you about family planning methods?	325a = 1<br>○ Yes<br>○ No<br>○ No response

	Yes	No	No response
	009a = 1		
326. In the last few months have you:			
326a. Heard about family planning on the radio?	○	○	○
326b. Seen anything about family planning on the television?	○	○	○
326c. Read about family planning in a newspaper or magazine?	○	○	○
326d. Received a voice or text message about family planning on a mobile phone?	○	○	○

Section 4 – Sexual Activity

CHECK FOR THE PRESENCE OF OTHERS. BEFORE CONTINUING, MAKE EVERY EFFORT TO ENSURE PRIVACY.

Now I would like to ask some questions about sexual activity in order to gain a better understanding of some important life issues. Let me assure you again that your answers are completely confidential and will not be told to anyone. If we should come to any question that you don't want to answer, just let me know and we will go to the next question.	${consent_obtained}

	${consent_obtained}
401a. How old were you when you first had sexual intercourse?	309a = 1
Current age: [AGE]	
Number of live births: [NUMBER OF LIFE BIRTHS]	${birth_events} > 0
The respondent is pregnant	${pregnant} = 'yes'

Enter the age in years.<br>*Enter -77 if she has never had sex. Enter -99 for no response. Enter -88 for do not know.*	--

	((${age_at_first_sex} >= 0) or (${age_at_first_sex} =-88) or (${age_at_first_sex} =-99))
402. When was the last time you had sexual intercourse?	401a ≠ -77

402. Enter [# days / weeks / months / years].<br>*If today, enter zero days only, not zero weeks/months/years.*<br>*Must agree with the age of first sexual intercourse and the pregnancy status.*	401a ≠ -77<br>--
LCL_403. In your opinion, if you have sex only once without using birth control, will you get pregnant?<br>*Read response options aloud.*	${consent_obtained}<br>○ Definitely yes<br>○ Maybe yes<br>○ Maybe no<br>○ Definitely no<br>○ Do not know<br>○ No response
LCL_404. In your opinion, if you have sex regularly, say twice a week, for a year without using birth control, will you get pregnant?<br>*Read response options aloud.*	${consent_obtained}<br>○ Definitely yes<br>○ Maybe yes<br>○ Maybe no<br>○ Definitely no<br>○ Do not know<br>○ No response

Section 6 – Menstrual Hygiene

Now I'm going to ask you about menstrual hygiene management. This includes the use of absorbent materials; access to a private, clean, safe space; washing as required; and a place to dispose used materials.

602a. Where do you most often change your used pads, cloths, or other sanitary materials?	(209 ≤ 90 days, 13 weeks or ≤ 3 months)<br>○ Flush/pour flush toilet<br>○ Ventilated improved pit latrine<br>○ Pit latrine with slab<br>○ Pit latrine without slab / open pit<br>○ Bucket toilet<br>○ Composting toilet<br>○ Hanging toilet /Hanging latrine<br>○ Shower/washing area<br>○ Sleeping area/bedroom<br>○ Backyard<br>○ No facility / bush / field<br>○ Other<br>○ No response
Place: [MAIN PLACE FROM 602a] 602b. While managing your menstrual hygiene, was this place:<br>*Read each option aloud and select if yes.*	601 ≠ -99 nor null AND 602 ≠ -99 nor null<br>☐ Clean?<br>☐ Private?<br>☐ Safe?<br>☐ Able to be locked?<br>☐ Supplied with water?<br>☐ Supplied with soap?<br>☐ None of the above<br>☐ No response
603. During your last menstrual period, what did you use to collect or absorb your menstrual blood?<br>PROBE: Anything else?<br>*Do not read options aloud. Select all that apply.*	(209 ≤ 90 days, 13 weeks or ≤ 3 months)<br>☐ Disposable sanitary pad (commercial)<br>☐ Reusable sanitary pad<br>☐ New cloth<br>☐ Old cloth<br>☐ Cotton wool

	☐ Diaper<br>☐ Tampons<br>☐ Toilet paper<br>☐ Underwear alone<br>☐ Bucket<br>☐ Other<br>☐ No materials used<br>☐ No response
604a. Did you wash and reuse pads, cloths, or other sanitary materials during your last menstrual period?	603 = reusable sanitary pad, old cloth, underwear alone, or bucket<br>○ Yes<br>○ No<br>○ No response
604b. During your last menstrual period, were the sanitary materials that you washed and reused completely dried before each reuse?	604a = 1<br>○ Yes<br>○ No<br>○ No response
605. You mentioned that you used [ODK will display the responses from 603] during your last menstrual period. Where did you dispose of these materials after use? PROBE: Anywhere else?<br>*Do not read options aloud. Select all that apply.*	603 = disposable sanitary pad, new cloth, cotton wool, diaper, tampons, toilet paper, or other OR 604a =0<br>☐ Flush toilet<br>☐ Latrine<br>☐ Waste bin/trash bag<br>☐ Burning<br>☐ Bush/field<br>☐ Other<br>☐ No response
606a. Aside from your own housework, have you done any work in the last month?	(209 ≤ 90 days, 13 weeks or ≤ 3 months)<br>○ Yes<br>○ No<br>○ No response
606b. Due to your last menstrual period, were there any work days in the last month that you did not attend?	606a = 1<br>○ Yes<br>○ No<br>○ No response
607a. Did you attend school at any time in the past 12 months?	102 = 15 - 24 AND 103 ≠ never<br>○ Yes<br>○ No<br>○ No response
607b. Due to your menstrual period, were there any school days in the past 12 months that you did not attend?	607a = 1<br>○ Yes<br>○ No<br>○ No response

Section 7.1 – Confidantes

701. Now I want to ask some questions about your closest female friends or relatives. These are women whom you share secrets with and who also share theirs with you. How many female friends or relatives like this do you have in Cote d'Ivoire who are between the ages of 15 and 49?<br>*Enter -88 for do not know, -99 for no response.*	${consent_obtained}<br>--
702a. Please picture your closest female friend or relative in Cote d'Ivoire. Again, this is a woman between the ages of 15 and 49 with whom you share secrets and who also shares hers with you. For ease of referencing this woman, please provide a fake name.	${friend_count} > 0<br>--
703a. How old was ${friend1_name} at her last birthday?<br>*Enter -88 for do not know, -99 for no response.*	${friend_count} > 0<br>--
704a. What is the highest level of school ${friend1_name} has ever attended?	${friend_count} > 0<br>○ Never attended<br>○ Primary

	○ Secondary<br>○ Tertiary<br>○ No response
702b. Please picture your next closest female friend or relative in Cote d'Ivoire. Again, this is a woman between the ages of 15 and 49 with whom you share secrets and who also shares hers with you. For ease of referencing this woman, please provide a fake name.	${friend_count} > 1<br>--
703b. How old was ${friend2_name} at her last birthday?<br>*Enter -88 for do not know, -99 for no response.*	${friend_count} > 1<br>--
704b. What is the highest level of school ${friend2_name} has ever attended?	${friend_count} > 1<br>○ Never attended<br>○ Primary<br>○ Secondary<br>○ Tertiary<br>○ No response

Section 7.2 – Abortion

CHECK FOR THE PRESENCE OF OTHERS. BEFORE CONTINUING, MAKE EVERY EFFORT TO ENSURE PRIVACY.

The next series of questions are about things women in your community do to remove a pregnancy. This is a common experience in Cote d'Ivoire and we simply want to better understand what women do to remove a pregnancy. I want to remind you that this survey is completely confidential and anonymous and your responses will not be shared with anyone. If we should come to any question that you don't want to answer, just let me know and I will skip to the next question.<br>*Press OK to continue.*	${consent_obtained}<br>☐ OK
705. Sometimes women are worried they are pregnant or get pregnant when they do not want to be and they do something to remove the pregnancy. How common is this in the place where you currently live?<br>*Read response options aloud.*	${consent_obtained}<br>○ Very common<br>○ Somewhat common<br>○ Not very common<br>○ Not at all<br>○ Do not know<br>○ No response
706a. In the place where you currently live, what are the ways a woman who is pregnant or worried that she is pregnant can remove a pregnancy? Anything else?<br>*Do not read options aloud. Select all that apply.*	${consent_obtained} and (${abt_common} != 'not_common')<br>☐ Surgical procedure (curettage, MVA, etc.)<br>☐ Pills called mifepristone or misoprostol (Cytotec)<br>☐ Medicines you take when you have a fever like antibiotics or anti-malarial medicine (Palujecte, Novaquine, Cyphaquine<br>☐ Other pills<br>☐ Traditional methods that are not inserted into the vagina (herbs, potions, concoctions)<br>☐ Ingested industrial products (bleach, Coke-Nescafé mix, etc.)<br>☐ Insert materials into the vagina (stem, herb ball, kanigban, etc.)<br>☐ Other<br>☐ Do not know<br>☐ No response
706b. Nonetheless, in your opinion, what could a woman in the place where you live do to remove a pregnancy when she is pregnant or worried that she is pregnant? Anything else?	${consent_obtained} and (${abt_common} = 'not_common')<br>☐ Surgical procedure (curettage, MVA, etc.)

	☐ Pills called mifepristone or misoprostol (Cytotec)<br>☐ Medicines you take when you have a fever like antibiotics or anti-malarial medicine (Palujecte, Novaquine, Cyphaquine<br>☐ Other pills<br>☐ Traditional methods that are not inserted into the vagina (herbs, potions, concoctions)<br>☐ Ingested industrial products (bleach, Coke-Nescafé mix, etc.)<br>☐ Insert materials into the vagina (stem, herb ball, kanigban, etc.)<br>☐ Other<br>☐ Do not know<br>☐ No response
707. Which of these ways is the most common?<br>*Select only one response*	count-selected(${abt_ways}) > 1<br>○ Surgical procedure (curettage, MVA, etc.)<br>○ Pills called mifepristone or misoprostol (Cytotec)<br>○ Medicines you take when you have a fever like antibiotics or anti-malarial medicine (Palujecte, Novaquine, Cyphaquine<br>○ Other pills<br>○ Traditional methods that are not inserted into the vagina (herbs, potions, concoctions)<br>○ Ingested industrial products (bleach, Coke-Nescafé mix, etc.)<br>○ Insert materials into the vagina (stem, herb ball, kanigban, etc.)<br>○ Other<br>○ Do not know<br>○ No response<br>selected(${abt_ways}, filter_list) or (filter_list = 'always')
708. Where do women go for a surgical procedure to remove a pregnancy? Anywhere else?<br>*Do not read options aloud. Select all that apply.*	(selected(${abt_ways},'surgery'))<br>☐ Government hospital<br>☐ Government health center<br>☐ Family planning clinic<br>☐ Mobile clinic (public)<br>☐ Other public<br>☐ Private hospital / clinic<br>☐ Pharmacy<br>☐ Private doctor<br>☐ Mobile clinic (private)<br>☐ Health agent<br>☐ Other private<br>☐ Store<br>☐ Religious organizations<br>☐ Community event<br>☐ Friend / parent<br>☐ Community health agent<br>☐ Street vendor<br>☐ Other<br>☐ Do not know<br>☐ No response
709. Which of these places is the most common?<br>*Select only one response*	count-selected(${abt_surg_where}) > 1<br>○ Government hospital

	○ Government health center<br>○ Family planning clinic<br>○ Mobile clinic (public)<br>○ Other public<br>○ Private hospital / clinic<br>○ Pharmacy<br>○ Private doctor<br>○ Mobile clinic (private)<br>○ Health agent<br>○ Other private<br>○ Store<br>○ Religious organizations<br>○ Community event<br>○ Friend / parent<br>○ Community health agent<br>○ Street vendor<br>○ Other<br>○ Do not know<br>○ No response<br>selected(${abt_surg_where}, filter_list) or (filter_list = 'always')
710. Where do women get the medicines to remove a pregnancy? Anywhere else?<br>*Do not read options aloud. Select all that apply.*	(selected(${abt_ways}, 'pills_abortion')) or (selected(${abt_ways}, 'pills_fever')) or (selected($ …<br>☐ Government hospital<br>☐ Government health center<br>☐ Family planning clinic<br>☐ Mobile clinic (public)<br>☐ Other public<br>☐ Private hospital / clinic<br>☐ Pharmacy<br>☐ Private doctor<br>☐ Mobile clinic (private)<br>☐ Health agent<br>☐ Other private<br>☐ Store<br>☐ Religious organizations<br>☐ Community event<br>☐ Friend / parent<br>☐ Community health agent<br>☐ Street vendor<br>☐ Other<br>☐ Do not know<br>☐ No response
711. Which of these sources is the most common?<br>*Select only one response*	count-selected(${abt_meds_where}) > 1<br>○ Government hospital<br>○ Government health center<br>○ Family planning clinic<br>○ Mobile clinic (public)<br>○ Other public<br>○ Private hospital / clinic<br>○ Pharmacy<br>○ Private doctor<br>○ Mobile clinic (private)<br>○ Health agent<br>○ Other private<br>○ Store<br>○ Religious organizations<br>○ Community event<br>○ Friend / parent<br>○ Community health agent<br>○ Street vendor

	◌ Other<br>◌ Do not know<br>◌ No response<br>selected(${abt_meds_where}, filter_list) or (filter_list = 'always')
712a.i. Now I want to ask some more questions about ${friend1_name}. Has she ever done something to remove a pregnancy when she was pregnant or worried she was pregnant?<br>*Probe to confirm whether the pregnancy removal was successful. If not, select 'no.'*	◌ Yes, I am certain<br>◌ Yes, I think so<br>◌ No<br>◌ Do not know<br>◌ No response
713a.i. In what year did this last happen?<br>*If indicates happened more than once, specify most recent time.*<br>*Enter 2020 for 'Do not know' or 'No response'.*	(${friend1_abt_yn} = 'yes') or (${friend1_abt_yn} = 'likely')<br>Year: ___________________
714a.i. Women sometimes do many things to stop a pregnancy from continuing. Did ${friend1_name} do more than one thing to try to remove the pregnancy?	(${friend1_abt_yn} = 'yes') or (${friend1_abt_yn} = 'likely')<br>◌ Yes, I am certain<br>◌ Yes, I think so<br>◌ No<br>◌ Do not know<br>◌ No response
715a.i. What was the first thing she did to try to remove the pregnancy?	(${friend1_abt_mult_yn} = 'yes') or (${friend1_abt_mult_yn} = 'likely')<br>◌ Surgical procedure (curettage, MVA, etc.)<br>◌ Pills called mifepristone or misoprostol (Cytotec)<br>◌ Medicines you take when you have a fever like antibiotics or anti-malarial medicine (Palujecte, Novaquine, Cyphaquine<br>◌ Other pills<br>◌ Traditional methods that are not inserted into the vagina (herbs, potions, concoctions)<br>◌ Ingested industrial products (bleach, Coke-Nescafé mix, etc.)<br>◌ Insert materials into the vagina (stem, herb ball, kanigban, etc.)<br>◌ Other<br>◌ Do not know<br>◌ No response
715a.i. What did she do that removed the pregnancy?	(${friend1_abt_mult_yn} = 'no') or (${friend1_abt_mult_yn} = '-88')<br>◌ Surgical procedure (curettage, MVA, etc.)<br>◌ Pills called mifepristone or misoprostol (Cytotec)<br>◌ Medicines you take when you have a fever like antibiotics or anti-malarial medicine (Palujecte, Novaquine, Cyphaquine<br>◌ Other pills<br>◌ Traditional methods that are not inserted into the vagina (herbs, potions, concoctions)<br>◌ Ingested industrial products (bleach, Coke-Nescafé mix, etc.)<br>◌ Insert materials into the vagina (stem, herb ball, kanigban, etc.)<br>◌ Other<br>◌ Do not know<br>◌ No response

716a.i. Where did she go for the procedure?	(${friend1_abt_first} = 'surgery') or (${friend1_abt_only} = 'surgery')<br>○ Government hospital<br>○ Government health center<br>○ Family planning clinic<br>○ Mobile clinic (public)<br>○ Other public<br>○ Private hospital / clinic<br>○ Pharmacy<br>○ Private doctor<br>○ Mobile clinic (private)<br>○ Health agent<br>○ Other private<br>○ Store<br>○ Religious organizations<br>○ Community event<br>○ Friend / parent<br>○ Community health agent<br>○ Street vendor<br>○ Other<br>○ Do not know<br>○ No response
717a.i. Where did she get the medicines?	(${friend1_abt_first} = 'pills_abortion') or (${friend1_abt_only} = 'pills_abortion') or (${friend ...<br>○ Government hospital<br>○ Government health center<br>○ Family planning clinic<br>○ Mobile clinic (public)<br>○ Other public<br>○ Private hospital / clinic<br>○ Pharmacy<br>○ Private doctor<br>○ Mobile clinic (private)<br>○ Health agent<br>○ Other private<br>○ Store<br>○ Religious organizations<br>○ Community event<br>○ Friend / parent<br>○ Community health agent<br>○ Street vendor<br>○ Other<br>○ Do not know<br>○ No response
718a.i. What was the last thing she did that ultimately removed the pregnancy?	(${friend1_abt_mult_yn} = 'yes') or (${friend1_abt_mult_yn} = 'likely')<br>○ Surgical procedure (curettage, MVA, etc.)<br>○ Pills called mifepristone or misoprostol (Cytotec)<br>○ Medicines you take when you have a fever like antibiotics or anti-malarial medicine (Palujecte, Novaquine, Cyphaquine<br>○ Other pills<br>○ Traditional methods that are not inserted into the vagina (herbs, potions, concoctions)<br>○ Ingested industrial products (bleach, Coke-Nescafé mix, etc.)<br>○ Insert materials into the vagina

	(stem, herb ball, kanigban, etc.)<br>◯ Other<br>◯ Do not know<br>◯ No response
719a.i. Where did she go for the procedure?	${friend1_abt_last} = 'surgery'<br>◯ Government hospital<br>◯ Government health center<br>◯ Family planning clinic<br>◯ Mobile clinic (public)<br>◯ Other public<br>◯ Private hospital / clinic<br>◯ Pharmacy<br>◯ Private doctor<br>◯ Mobile clinic (private)<br>◯ Health agent<br>◯ Other private<br>◯ Store<br>◯ Religious organizations<br>◯ Community event<br>◯ Friend / parent<br>◯ Community health agent<br>◯ Street vendor<br>◯ Other<br>◯ Do not know<br>◯ No response
720a.i. Where did she get the medicines?	(${friend1_abt_last} = 'pills_abortion') or (${friend1_abt_last} = 'pills_fever') or (${friend1_ab …<br>◯ Government hospital<br>◯ Government health center<br>◯ Family planning clinic<br>◯ Mobile clinic (public)<br>◯ Other public<br>◯ Private hospital / clinic<br>◯ Pharmacy<br>◯ Private doctor<br>◯ Mobile clinic (private)<br>◯ Health agent<br>◯ Other private<br>◯ Store<br>◯ Religious organizations<br>◯ Community event<br>◯ Friend / parent<br>◯ Community health agent<br>◯ Street vendor<br>◯ Other<br>◯ Do not know<br>◯ No response
721a.i. Did ${friend1_name} have any issues and go to a health facility for treatment in the process of removing the pregnancy?<br>*If the respondent already reported the friend went to a health facility in the process of removing the pregnancy, we are interested in whether the friend went back to a health facility on a separate occasion to treat complications that she may have experienced.*	(${friend1_abt_yn} = 'yes') or (${friend1_abt_yn} = 'likely')<br>◯ Yes, I am certain<br>◯ Yes, I think so<br>◯ No<br>◯ Do not know<br>◯ No response
712a.ii. Besides this event, has ${friend1_name} ever done something to regulate her period when she was worried she was pregnant?<br>*Probe to confirm whether the period regulation was successful. If not, select 'no.'*	(${friend1_abt_yn} = 'yes') or (${friend1_abt_yn} = 'likely')<br>◯ Yes, I am certain<br>◯ Yes, I think so<br>◯ No<br>◯ Do not know<br>◯ No response

712a.ii. Has ${friend1_name} ever done something to regulate her period when she was worried she was pregnant?<br>*Probe to confirm whether the period regulation was successful. If not, select 'no.'*	(${friend1_abt_yn} != 'yes') and (${friend1_abt_yn} != 'likely')<br>○ Yes, I am certain<br>○ Yes, I think so<br>○ No<br>○ Do not know<br>○ No response
713a.ii. In what year did this last happen?<br>*If indicates happened more than once, specify most recent time.*<br>*Enter 2020 for 'Do not know' or 'No response'.*	(${friend1_reg_yn} = 'yes') or (${friend1_reg_yn} = 'likely')<br>Year: ________________
714a.ii. Women sometimes do many things to regulate their period. Did ${friend1_name} do more than one thing to try to regulate her period?	((${friend1_reg_year} > ${friend1_abt_year}) or (${friend1_abt_year} = '')) and ((${friend1_reg_yn …<br>○ Yes, I am certain<br>○ Yes, I think so<br>○ No<br>○ Do not know<br>○ No response
715a.ii. What was the first thing she did to try to regulate her period?	((${friend1_reg_year} > ${friend1_abt_year}) or (${friend1_abt_year} = '')) and ((${friend1_reg_mu …<br>○ Surgical procedure (curettage, MVA, etc.)<br>○ Pills called mifepristone or misoprostol (Cytotec)<br>○ Medicines you take when you have a fever like antibiotics or anti-malarial medicine (Palujecte, Novaquine, Cyphaquine<br>○ Other pills<br>○ Traditional methods that are not inserted into the vagina (herbs, potions, concoctions)<br>○ Ingested industrial products (bleach, Coke-Nescafé mix, etc.)<br>○ Insert materials into the vagina (stem, herb ball, kanigban, etc.)<br>○ Other<br>○ Do not know<br>○ No response
715a.ii. What did she do that regulated her period?	((${friend1_reg_year} > ${friend1_abt_year}) or (${friend1_abt_year} = '')) and ((${friend1_reg_mu …<br>○ Surgical procedure (curettage, MVA, etc.)<br>○ Pills called mifepristone or misoprostol (Cytotec)<br>○ Medicines you take when you have a fever like antibiotics or anti-malarial medicine (Palujecte, Novaquine, Cyphaquine<br>○ Other pills<br>○ Traditional methods that are not inserted into the vagina (herbs, potions, concoctions)<br>○ Ingested industrial products (bleach, Coke-Nescafé mix, etc.)<br>○ Insert materials into the vagina (stem, herb ball, kanigban, etc.)<br>○ Other<br>○ Do not know<br>○ No response

716a.ii. Where did she go for the procedure?	((${friend1_reg_year} > ${friend1_abt_year}) or (${friend1_abt_year} = '')) and ((${friend1_reg_fi …	○ Government hospital<br>○ Government health center<br>○ Family planning clinic<br>○ Mobile clinic (public)<br>○ Other public<br>○ Private hospital / clinic<br>○ Pharmacy<br>○ Private doctor<br>○ Mobile clinic (private)<br>○ Health agent<br>○ Other private<br>○ Store<br>○ Religious organizations<br>○ Community event<br>○ Friend / parent<br>○ Community health agent<br>○ Street vendor<br>○ Other<br>○ Do not know<br>○ No response
717a.ii. Where did she get the medicines?	((${friend1_reg_year} > ${friend1_abt_year}) or (${friend1_abt_year} = '')) and ((${friend1_reg_fi …	○ Government hospital<br>○ Government health center<br>○ Family planning clinic<br>○ Mobile clinic (public)<br>○ Other public<br>○ Private hospital / clinic<br>○ Pharmacy<br>○ Private doctor<br>○ Mobile clinic (private)<br>○ Health agent<br>○ Other private<br>○ Store<br>○ Religious organizations<br>○ Community event<br>○ Friend / parent<br>○ Community health agent<br>○ Street vendor<br>○ Other<br>○ Do not know<br>○ No response
718a.ii. What was the last thing she did that ultimately caused her period to return?	((${friend1_reg_year} > ${friend1_abt_year}) or (${friend1_abt_year} = '')) and ((${friend1_reg_mu …	○ Surgical procedure (curettage, MVA, etc.)<br>○ Pills called mifepristone or misoprostol (Cytotec)<br>○ Medicines you take when you have a fever like antibiotics or anti-malarial medicine (Palujecte, Novaquine, Cyphaquine<br>○ Other pills<br>○ Traditional methods that are not inserted into the vagina (herbs, potions, concoctions)<br>○ Ingested industrial products (bleach, Coke-Nescafé mix, etc.)

	◌ Insert materials into the vagina (stem, herb ball, kanigban, etc.)<br>◌ Other<br>◌ Do not know<br>◌ No response
719a.ii. Where did she go for the procedure?	((${friend1_reg_year} > ${friend1_abt_year}) or (${friend1_abt_year} = '')) and (${friend1_reg_las …<br>◌ Government hospital<br>◌ Government health center<br>◌ Family planning clinic<br>◌ Mobile clinic (public)<br>◌ Other public<br>◌ Private hospital / clinic<br>◌ Pharmacy<br>◌ Private doctor<br>◌ Mobile clinic (private)<br>◌ Health agent<br>◌ Other private<br>◌ Store<br>◌ Religious organizations<br>◌ Community event<br>◌ Friend / parent<br>◌ Community health agent<br>◌ Street vendor<br>◌ Other<br>◌ Do not know<br>◌ No response
720a.ii. Where did she get the medicines?	((${friend1_reg_year} > ${friend1_abt_year}) or (${friend1_abt_year} = '')) and ((${friend1_reg_la …<br>◌ Government hospital<br>◌ Government health center<br>◌ Family planning clinic<br>◌ Mobile clinic (public)<br>◌ Other public<br>◌ Private hospital / clinic<br>◌ Pharmacy<br>◌ Private doctor<br>◌ Mobile clinic (private)<br>◌ Health agent<br>◌ Other private<br>◌ Store<br>◌ Religious organizations<br>◌ Community event<br>◌ Friend / parent<br>◌ Community health agent<br>◌ Street vendor<br>◌ Other<br>◌ Do not know<br>◌ No response
721a.ii. Did ${friend1_name} have any issues and go to a health facility for treatment in the process of regulating her period?<br>*If the respondent already reported the friend went to a health facility in the process of regulating her period, we are interested in whether the friend went back to a health facility on a separate occasion to treat complications that she may have experienced.*	((${friend1_reg_year} > ${friend1_abt_year}) or (${friend1_abt_year} = '')) and ((${friend1_reg_yn …<br>◌ Yes, I am certain<br>◌ Yes, I think so<br>◌ No<br>◌ Do not know<br>◌ No response
712b.i. Now I want to ask some more questions about ${friend2_name}. Has she ever done something to remove a pregnancy when she was pregnant or worried she was pregnant?<br>*Probe to confirm whether the pregnancy removal was successful. If not,*	◌ Yes, I am certain<br>◌ Yes, I think so<br>◌ No<br>◌ Do not know

select 'no.'	◌ No response
713b.i. In what year did this last happen?<br>*If indicates happened more than once, specify most recent time.*<br>*Enter 2020 for 'Do not know' or 'No response'.*	(${friend2_abt_yn} = 'yes') or (${friend2_abt_yn} = 'likely')<br>Year: ________________
714b.i. Women sometimes do many things to stop a pregnancy from continuing. Did ${friend2_name} do more than one thing to try to remove the pregnancy?	(${friend2_abt_yn} = 'yes') or (${friend2_abt_yn} = 'likely')<br>◌ Yes, I am certain<br>◌ Yes, I think so<br>◌ No<br>◌ Do not know<br>◌ No response
715b.i. What was the first thing she did to try to remove the pregnancy?	(${friend2_abt_mult_yn} = 'yes') or (${friend2_abt_mult_yn} = 'likely')<br>◌ Surgical procedure (curettage, MVA, etc.)<br>◌ Pills called mifepristone or misoprostol (Cytotec)<br>◌ Medicines you take when you have a fever like antibiotics or anti-malarial medicine (Palujecte, Novaquine, Cyphaquine<br>◌ Other pills<br>◌ Traditional methods that are not inserted into the vagina (herbs, potions, concoctions)<br>◌ Ingested industrial products (bleach, Coke-Nescafé mix, etc.)<br>◌ Insert materials into the vagina (stem, herb ball, kanigban, etc.)<br>◌ Other<br>◌ Do not know<br>◌ No response
715b.i. What did she do that removed the pregnancy?	(${friend2_abt_mult_yn} = 'no') or (${friend2_abt_mult_yn} = '-88')<br>◌ Surgical procedure (curettage, MVA, etc.)<br>◌ Pills called mifepristone or misoprostol (Cytotec)<br>◌ Medicines you take when you have a fever like antibiotics or anti-malarial medicine (Palujecte, Novaquine, Cyphaquine<br>◌ Other pills<br>◌ Traditional methods that are not inserted into the vagina (herbs, potions, concoctions)<br>◌ Ingested industrial products (bleach, Coke-Nescafé mix, etc.)<br>◌ Insert materials into the vagina (stem, herb ball, kanigban, etc.)<br>◌ Other<br>◌ Do not know<br>◌ No response
716b.i. Where did she go for the procedure?	(${friend2_abt_first} = 'surgery') or (${friend2_abt_only} = 'surgery')<br>◌ Government hospital<br>◌ Government health center<br>◌ Family planning clinic<br>◌ Mobile clinic (public)<br>◌ Other public<br>◌ Private hospital / clinic

	Pharmacy<br>Private doctor<br>Mobile clinic (private)<br>Health agent<br>Other private<br>Store<br>Religious organizations<br>Community event<br>Friend / parent<br>Community health agent<br>Street vendor<br>Other<br>Do not know<br>No response
717b.i. Where did she get the medicines?	(${friend2_abt_first} = 'pills_abortion') or (${friend2_abt_only} = 'pills_abortion') or (${friend …<br>Government hospital<br>Government health center<br>Family planning clinic<br>Mobile clinic (public)<br>Other public<br>Private hospital / clinic<br>Pharmacy<br>Private doctor<br>Mobile clinic (private)<br>Health agent<br>Other private<br>Store<br>Religious organizations<br>Community event<br>Friend / parent<br>Community health agent<br>Street vendor<br>Other<br>Do not know<br>No response
718b.i. What was the last thing she did that ultimately removed the pregnancy?	(${friend2_abt_mult_yn} = 'yes') or (${friend2_abt_mult_yn} = 'likely')<br>Surgical procedure (curettage, MVA, etc.)<br>Pills called mifepristone or misoprostol (Cytotec)<br>Medicines you take when you have a fever like antibiotics or anti-malarial medicine (Palujecte, Novaquine, Cyphaquine<br>Other pills<br>Traditional methods that are not inserted into the vagina (herbs, potions, concoctions)<br>Ingested industrial products (bleach, Coke-Nescafé mix, etc.)<br>Insert materials into the vagina (stem, herb ball, kanigban, etc.)<br>Other<br>Do not know<br>No response
719b.i. Where did she go for the procedure?	${friend2_abt_last} = 'surgery'<br>Government hospital<br>Government health center<br>Family planning clinic

	◯ Mobile clinic (public)<br>◯ Other public<br>◯ Private hospital / clinic<br>◯ Pharmacy<br>◯ Private doctor<br>◯ Mobile clinic (private)<br>◯ Health agent<br>◯ Other private<br>◯ Store<br>◯ Religious organizations<br>◯ Community event<br>◯ Friend / parent<br>◯ Community health agent<br>◯ Street vendor<br>◯ Other<br>◯ Do not know<br>◯ No response
720b.i. Where did she get the medicines?	(${friend2_abt_last} = 'pills_abortion') or (${friend2_abt_last} = 'pills_fever') or (${friend2_ab …<br>◯ Government hospital<br>◯ Government health center<br>◯ Family planning clinic<br>◯ Mobile clinic (public)<br>◯ Other public<br>◯ Private hospital / clinic<br>◯ Pharmacy<br>◯ Private doctor<br>◯ Mobile clinic (private)<br>◯ Health agent<br>◯ Other private<br>◯ Store<br>◯ Religious organizations<br>◯ Community event<br>◯ Friend / parent<br>◯ Community health agent<br>◯ Street vendor<br>◯ Other<br>◯ Do not know<br>◯ No response
721b.i. Did ${friend2_name} have any issues and go to a health facility for treatment in the process of removing the pregnancy?<br>*If the respondent already reported the friend went to a health facility in the process of removing the pregnancy, we are interested in whether the friend went back to a health facility on a separate occasion to treat complications that she may have experienced.*	(${friend2_abt_yn} = 'yes') or (${friend2_abt_yn} = 'likely')<br>◯ Yes, I am certain<br>◯ Yes, I think so<br>◯ No<br>◯ Do not know<br>◯ No response
712b.ii. Besides this event, has ${friend2_name} ever done something to regulate her period when she was worried she was pregnant?<br>*Probe to confirm whether the period regulation was successful. If not, select 'no.'*	(${friend2_abt_yn} = 'yes') or (${friend2_abt_yn} = 'likely')<br>◯ Yes, I am certain<br>◯ Yes, I think so<br>◯ No<br>◯ Do not know<br>◯ No response
712b.ii. Has ${friend2_name} ever done something to regulate her period when she was worried she was pregnant?<br>*Probe to confirm whether the period regulation was successful. If not, select 'no.'*	(${friend2_abt_yn} != 'yes') and (${friend2_abt_yn} != 'likely')<br>◯ Yes, I am certain<br>◯ Yes, I think so<br>◯ No<br>◯ Do not know<br>◯ No response
713b.ii. In what year did this last happen?	(${friend2_reg_yn} = 'yes') or

If indicates happened more than once, specify most recent time. Enter 2020 for 'Do not know' or 'No response'.	(${friend2_reg_yn} = 'likely') Year:
714b.ii. Women sometimes do many things to regulate their period. Did ${friend2_name} do more than one thing to try to regulate her period?	((${friend2_reg_year} > ${friend2_abt_year}) or (${friend2_abt_year} = '')) and ((${friend2_reg_yn ... ◯ Yes, I am certain<br>◯ Yes, I think so<br>◯ No<br>◯ Do not know<br>◯ No response
715b.ii. What was the first thing she did to try to regulate her period?	((${friend2_reg_year} > ${friend2_abt_year}) or (${friend2_abt_year} = '')) and ((${friend2_reg_mu ... ◯ Surgical procedure (curettage, MVA, etc.)<br>◯ Pills called mifepristone or misoprostol (Cytotec)<br>◯ Medicines you take when you have a fever like antibiotics or anti-malarial medicine (Palujecte, Novaquine, Cyphaquine<br>◯ Other pills<br>◯ Traditional methods that are not inserted into the vagina (herbs, potions, concoctions)<br>◯ Ingested industrial products (bleach, Coke-Nescafé mix, etc.)<br>◯ Insert materials into the vagina (stem, herb ball, kanigban, etc.)<br>◯ Other<br>◯ Do not know<br>◯ No response
715b.ii. What did she do that regulated her period?	((${friend2_reg_year} > ${friend2_abt_year}) or (${friend2_abt_year} = '')) and ((${friend2_reg_mu ... ◯ Surgical procedure (curettage, MVA, etc.)<br>◯ Pills called mifepristone or misoprostol (Cytotec)<br>◯ Medicines you take when you have a fever like antibiotics or anti-malarial medicine (Palujecte, Novaquine, Cyphaquine<br>◯ Other pills<br>◯ Traditional methods that are not inserted into the vagina (herbs, potions, concoctions)<br>◯ Ingested industrial products (bleach, Coke-Nescafé mix, etc.)<br>◯ Insert materials into the vagina (stem, herb ball, kanigban, etc.)<br>◯ Other<br>◯ Do not know<br>◯ No response
716b.ii. Where did she go for the procedure?	((${friend2_reg_year} > ${friend2_abt_year}) or (${friend2_abt_year} = '')) and ((${friend2_reg_fi ... ◯ Government hospital<br>◯ Government health center<br>◯ Family planning clinic<br>◯ Mobile clinic (public)<br>◯ Other public<br>◯ Private hospital / clinic

	○ Pharmacy<br>○ Private doctor<br>○ Mobile clinic (private)<br>○ Health agent<br>○ Other private<br>○ Store<br>○ Religious organizations<br>○ Community event<br>○ Friend / parent<br>○ Community health agent<br>○ Street vendor<br>○ Other<br>○ Do not know<br>○ No response
717b.ii. Where did she get the medicines?	((${friend2_reg_year} > ${friend2_abt_year}) or (${friend2_abt_year} = '')) and ((${friend2_reg_fi …<br>○ Government hospital<br>○ Government health center<br>○ Family planning clinic<br>○ Mobile clinic (public)<br>○ Other public<br>○ Private hospital / clinic<br>○ Pharmacy<br>○ Private doctor<br>○ Mobile clinic (private)<br>○ Health agent<br>○ Other private<br>○ Store<br>○ Religious organizations<br>○ Community event<br>○ Friend / parent<br>○ Community health agent<br>○ Street vendor<br>○ Other<br>○ Do not know<br>○ No response
718b.ii. What was the last thing she did that ultimately caused her period to return?	((${friend2_reg_year} > ${friend2_abt_year}) or (${friend2_abt_year} = '')) and ((${friend2_reg_mu …<br>○ Surgical procedure (curettage, MVA, etc.)<br>○ Pills called mifepristone or misoprostol (Cytotec)<br>○ Medicines you take when you have a fever like antibiotics or anti-malarial medicine (Palujecte, Novaquine, Cyphaquine<br>○ Other pills<br>○ Traditional methods that are not inserted into the vagina (herbs, potions, concoctions)<br>○ Ingested industrial products (bleach, Coke-Nescafé mix, etc.)<br>○ Insert materials into the vagina (stem, herb ball, kanigban, etc.)<br>○ Other<br>○ Do not know<br>○ No response
719b.ii. Where did she go for the procedure?	((${friend2_reg_year} > ${friend2_abt_year}) or (${friend2_abt_year} = '')) and (${friend2_reg_las …<br>○ Government hospital

	◌ Government health center<br>◌ Family planning clinic<br>◌ Mobile clinic (public)<br>◌ Other public<br>◌ Private hospital / clinic<br>◌ Pharmacy<br>◌ Private doctor<br>◌ Mobile clinic (private)<br>◌ Health agent<br>◌ Other private<br>◌ Store<br>◌ Religious organizations<br>◌ Community event<br>◌ Friend / parent<br>◌ Community health agent<br>◌ Street vendor<br>◌ Other<br>◌ Do not know<br>◌ No response
720b.ii. Where did she get the medicines?	((${friend2_reg_year} > ${friend2_abt_year}) or (${friend2_abt_year} = '')) and ((${friend2_reg_la …<br>◌ Government hospital<br>◌ Government health center<br>◌ Family planning clinic<br>◌ Mobile clinic (public)<br>◌ Other public<br>◌ Private hospital / clinic<br>◌ Pharmacy<br>◌ Private doctor<br>◌ Mobile clinic (private)<br>◌ Health agent<br>◌ Other private<br>◌ Store<br>◌ Religious organizations<br>◌ Community event<br>◌ Friend / parent<br>◌ Community health agent<br>◌ Street vendor<br>◌ Other<br>◌ Do not know<br>◌ No response
721b.ii. Did ${friend2_name} have any issues and go to a health facility for treatment in the process of regulating her period?<br>*If the respondent already reported the friend went to a health facility in the process of regulating her period, we are interested in whether the friend went back to a health facility on a separate occasion to treat complications that she may have experienced.*	((${friend2_reg_year} > ${friend2_abt_year}) or (${friend2_abt_year} = '')) and ((${friend2_reg_yn …<br>◌ Yes, I am certain<br>◌ Yes, I think so<br>◌ No<br>◌ Do not know<br>◌ No response
722a. Now I would like to ask about your own experience. Have you ever done something to remove a pregnancy when you were pregnant or worried you were pregnant?<br>*Probe to confirm whether the pregnancy removal was successful. If not, select 'no.'*	◌ Yes<br>◌ No<br>◌ No response
723a. In what year did this last happen?<br>*If indicates happened more than once, specify most recent time.*<br>*Enter 2020 for 'Do not know' or 'No response'.*	(${self_abt_yn} = 'yes')<br>Year: ------------------------------------
724a. Did you do more than one thing to try to remove the pregnancy?	(${self_abt_yn} = 'yes')<br>◌ Yes<br>◌ No<br>◌ No response

725a. What did you first do?	(${self_abt_mult_yn} = 'yes')<br>○ Surgical procedure (curettage, MVA, etc.)<br>○ Pills called mifepristone or misoprostol (Cytotec)<br>○ Medicines you take when you have a fever like antibiotics or anti-malarial medicine (Palujecte, Novaquine, Cyphaquine<br>○ Other pills<br>○ Traditional methods that are not inserted into the vagina (herbs, potions, concoctions)<br>○ Ingested industrial products (bleach, Coke-Nescafé mix, etc.)<br>○ Insert materials into the vagina (stem, herb ball, kanigban, etc.)<br>○ Other<br>○ Do not know<br>○ No response
725a. What did you do?	(${self_abt_mult_yn} = 'no')<br>○ Surgical procedure (curettage, MVA, etc.)<br>○ Pills called mifepristone or misoprostol (Cytotec)<br>○ Medicines you take when you have a fever like antibiotics or anti-malarial medicine (Palujecte, Novaquine, Cyphaquine<br>○ Other pills<br>○ Traditional methods that are not inserted into the vagina (herbs, potions, concoctions)<br>○ Ingested industrial products (bleach, Coke-Nescafé mix, etc.)<br>○ Insert materials into the vagina (stem, herb ball, kanigban, etc.)<br>○ Other<br>○ Do not know<br>○ No response
726a. Where did you go for the procedure?	(${self_abt_first} = 'surgery') or (${self_abt_only} = 'surgery')<br>○ Government hospital<br>○ Government health center<br>○ Family planning clinic<br>○ Mobile clinic (public)<br>○ Other public<br>○ Private hospital / clinic<br>○ Pharmacy<br>○ Private doctor<br>○ Mobile clinic (private)<br>○ Health agent<br>○ Other private<br>○ Store<br>○ Religious organizations<br>○ Community event<br>○ Friend / parent<br>○ Community health agent<br>○ Street vendor<br>○ Other<br>○ Do not know<br>○ No response

727a. Where did you get the medicines?	(${self_abt_first} = 'pills_abortion') or (${self_abt_only} = 'pills_abortion') or (${self_abt_fir …<br>○ Government hospital<br>○ Government health center<br>○ Family planning clinic<br>○ Mobile clinic (public)<br>○ Other public<br>○ Private hospital / clinic<br>○ Pharmacy<br>○ Private doctor<br>○ Mobile clinic (private)<br>○ Health agent<br>○ Other private<br>○ Store<br>○ Religious organizations<br>○ Community event<br>○ Friend / parent<br>○ Community health agent<br>○ Street vendor<br>○ Other<br>○ Do not know<br>○ No response
728a. What was the last thing you did that ultimately removed the pregnancy?	(${self_abt_mult_yn} = 'yes')<br>○ Surgical procedure (curettage, MVA, etc.)<br>○ Pills called mifepristone or misoprostol (Cytotec)<br>○ Medicines you take when you have a fever like antibiotics or anti-malarial medicine (Palujecte, Novaquine, Cyphaquine<br>○ Other pills<br>○ Traditional methods that are not inserted into the vagina (herbs, potions, concoctions)<br>○ Ingested industrial products (bleach, Coke-Nescafé mix, etc.)<br>○ Insert materials into the vagina (stem, herb ball, kanigban, etc.)<br>○ Other<br>○ Do not know<br>○ No response
729a. Where did you go for the procedure?	${self_abt_last} = 'surgery'<br>○ Government hospital<br>○ Government health center<br>○ Family planning clinic<br>○ Mobile clinic (public)<br>○ Other public<br>○ Private hospital / clinic<br>○ Pharmacy<br>○ Private doctor<br>○ Mobile clinic (private)<br>○ Health agent<br>○ Other private<br>○ Store<br>○ Religious organizations<br>○ Community event<br>○ Friend / parent<br>○ Community health agent<br>○ Street vendor

	◯ Other<br>◯ Do not know<br>◯ No response
730a. Where did you get the medicines?	(${self_abt_last} = 'pills_abortion') or (${self_abt_last} = 'pills_fever') or (${self_abt_last} = …<br>◯ Government hospital<br>◯ Government health center<br>◯ Family planning clinic<br>◯ Mobile clinic (public)<br>◯ Other public<br>◯ Private hospital / clinic<br>◯ Pharmacy<br>◯ Private doctor<br>◯ Mobile clinic (private)<br>◯ Health agent<br>◯ Other private<br>◯ Store<br>◯ Religious organizations<br>◯ Community event<br>◯ Friend / parent<br>◯ Community health agent<br>◯ Street vendor<br>◯ Other<br>◯ Do not know<br>◯ No response
731a. Did you have any issues and go to a health facility for treatment in the process of removing the pregnancy?<br>*If the respondent already reported she went to a health facility in the process of removing the pregnancy, we are interested in whether she went back to a health facility on a separate occasion to treat complications that she may have experienced.*	(${self_abt_yn} = 'yes')<br>◯ Yes<br>◯ No<br>◯ Do not know<br>◯ No response
732a. Did you tell any of the following people about this experience?<br>*Read the answer choices aloud. Select all that apply.*	(${self_abt_yn} = 'yes')<br>☐ Husband/male partner<br>☐ Sister<br>☐ Brother<br>☐ Mother<br>☐ Father<br>☐ Other relative<br>☐ Friend 1: ${friend1_name}<br>☐ Friend 2: ${friend2_name}<br>☐ Other friend<br>☐ Other person<br>☐ No one<br>☐ Do not know<br>☐ No response<br>(${friend1_name} != '' and ${friend1_name} != '-99' and filter_list = 'friend1') or (${friend2_name} != '' and ${friend2_name} != '-99' and filter_list = 'friend2') or (filter_list = 'always')
722b. Besides this event, have you ever done something to regulate your period when you were worried you were pregnant?<br>*Probe to confirm whether the period regulation was successful. If not, select 'no.'*	${self_abt_yn} = 'yes'<br>◯ Yes<br>◯ No<br>◯ No response
722b. Have you ever done something to regulate your period when you were worried you were pregnant?<br>*Probe to confirm whether the period regulation was successful. If not, select 'no.'*	${self_abt_yn} != 'yes'<br>◯ Yes<br>◯ No<br>◯ No response
723b. In what year did this last happen?<br>*If indicates happened more than once, specify most recent time.*<br>*Enter 2020 for 'Do not know' or 'No response'.*	(${self_reg_yn} = 'yes')<br>Year: ____________________
724b. Did you do more than one thing to try to regulate your	((${self_reg_year} > ${self_abt_year}) or (${self_abt_year} = '')) and ((${self_reg_yn} =

period?	'yes'))<br>○ Yes<br>○ No<br>○ No response
725b. What did you first do?	((${self_reg_year} > ${self_abt_year}) or (${self_abt_year} = '')) and ((${self_reg_mult_yn} = 'ye …<br>○ Surgical procedure (curettage, MVA, etc.)<br>○ Pills called mifepristone or misoprostol (Cytotec)<br>○ Medicines you take when you have a fever like antibiotics or anti-malarial medicine (Palujecte, Novaquine, Cyphaquine<br>○ Other pills<br>○ Traditional methods that are not inserted into the vagina (herbs, potions, concoctions)<br>○ Ingested industrial products (bleach, Coke-Nescafé mix, etc.)<br>○ Insert materials into the vagina (stem, herb ball, kanigban, etc.)<br>○ Other<br>○ Do not know<br>○ No response
725b. What did you do?	((${self_reg_year} > ${self_abt_year}) or (${self_abt_year} = '')) and ((${self_reg_mult_yn} = 'no …<br>○ Surgical procedure (curettage, MVA, etc.)<br>○ Pills called mifepristone or misoprostol (Cytotec)<br>○ Medicines you take when you have a fever like antibiotics or anti-malarial medicine (Palujecte, Novaquine, Cyphaquine<br>○ Other pills<br>○ Traditional methods that are not inserted into the vagina (herbs, potions, concoctions)<br>○ Ingested industrial products (bleach, Coke-Nescafé mix, etc.)<br>○ Insert materials into the vagina (stem, herb ball, kanigban, etc.)<br>○ Other<br>○ Do not know<br>○ No response
726b. Where did you go for the procedure?	((${self_reg_year} > ${self_abt_year}) or (${self_abt_year} = '')) and ((${self_reg_first} = 'surg …<br>○ Government hospital<br>○ Government health center<br>○ Family planning clinic<br>○ Mobile clinic (public)<br>○ Other public<br>○ Private hospital / clinic<br>○ Pharmacy<br>○ Private doctor<br>○ Mobile clinic (private)<br>○ Health agent<br>○ Other private

	Store<br>Religious organizations<br>Community event<br>Friend / parent<br>Community health agent<br>Street vendor<br>Other<br>Do not know<br>No response
727b. Where did you get the medicines?	((${self_reg_year} > ${self_abt_year}) or (${self_abt_year} = '')) and ((${self_reg_first} = 'pill …<br>Government hospital<br>Government health center<br>Family planning clinic<br>Mobile clinic (public)<br>Other public<br>Private hospital / clinic<br>Pharmacy<br>Private doctor<br>Mobile clinic (private)<br>Health agent<br>Other private<br>Store<br>Religious organizations<br>Community event<br>Friend / parent<br>Community health agent<br>Street vendor<br>Other<br>Do not know<br>No response
728b. What was the last thing you did that ultimately caused your period to return?	((${self_reg_year} > ${self_abt_year}) or (${self_abt_year} = '')) and ((${self_reg_mult_yn} = 'ye …<br>Surgical procedure (curettage, MVA, etc.)<br>Pills called mifepristone or misoprostol (Cytotec)<br>Medicines you take when you have a fever like antibiotics or anti-malarial medicine (Palujecte, Novaquine, Cyphaquine<br>Other pills<br>Traditional methods that are not inserted into the vagina (herbs, potions, concoctions)<br>Ingested industrial products (bleach, Coke-Nescafé mix, etc.)<br>Insert materials into the vagina (stem, herb ball, kanigban, etc.)<br>Other<br>Do not know<br>No response
729b. Where did you go for the procedure?	((${self_reg_year} > ${self_abt_year}) or (${self_abt_year} = '')) and (${self_reg_last} = 'surger …<br>Government hospital<br>Government health center<br>Family planning clinic<br>Mobile clinic (public)<br>Other public<br>Private hospital / clinic

	○ Pharmacy<br>○ Private doctor<br>○ Mobile clinic (private)<br>○ Health agent<br>○ Other private<br>○ Store<br>○ Religious organizations<br>○ Community event<br>○ Friend / parent<br>○ Community health agent<br>○ Street vendor<br>○ Other<br>○ Do not know<br>○ No response
730b. Where did you get the medicines?	((${self_reg_year} > ${self_abt_year}) or (${self_abt_year} = '')) and ((${self_reg_last} = 'pills …<br>○ Government hospital<br>○ Government health center<br>○ Family planning clinic<br>○ Mobile clinic (public)<br>○ Other public<br>○ Private hospital / clinic<br>○ Pharmacy<br>○ Private doctor<br>○ Mobile clinic (private)<br>○ Health agent<br>○ Other private<br>○ Store<br>○ Religious organizations<br>○ Community event<br>○ Friend / parent<br>○ Community health agent<br>○ Street vendor<br>○ Other<br>○ Do not know<br>○ No response
731b. Did you have any issues and go to a health facility for treatment in the process of regulating your period?<br>*If the respondent already reported she went to a health facility in the process of regulating her period, we are interested in whether she went back to a health facility on a separate occasion to treat complications that she may have experienced.*	((${self_reg_year} > ${self_abt_year}) or (${self_abt_year} = '')) and ((${self_reg_yn} = 'yes'))<br>○ Yes<br>○ No<br>○ Do not know<br>○ No response
732b. Did you tell any of the following people about this experience?<br>*Read the answer choices aloud. Select all that apply.*	((${self_reg_year} > ${self_abt_year}) or (${self_abt_year} = '')) and ((${self_reg_yn} = 'yes'))<br>☐ Husband/male partner<br>☐ Sister<br>☐ Brother<br>☐ Mother<br>☐ Father<br>☐ Other relative<br>☐ Friend 1: ${friend1_name}<br>☐ Friend 2: ${friend2_name}<br>☐ Other friend<br>☐ Other person<br>☐ No one<br>☐ Do not know<br>☐ No response<br>(${friend1_name} != '' and ${friend1_name} != '-99' and filter_list = 'friend1') or (${friend2_name} != '' and ${friend2_name} != '-99' and filter_list = 'friend2') or (filter_list =

	'always')
PNSME_701. What were the reasons that motivated you to remove the pregnancy for the last time ?	(${self_abt_yn} = 'yes')<br>☐ Husband’s refusal (refusal to accept the child)<br>☐ No means (to take care of the child)<br>☐ Rape<br>☐ Incest<br>☐ Mother has health issues<br>☐ Possible health issues of the foetus<br>☐ Not ready to take responsibility<br>☐ Too immature or too young to have a child<br>☐ Wants to continue school<br>☐ Woman’s parents want her to have an abortion<br>☐ Relationship issues or wants to avoid being a single mother.<br>☐ Has all the children she wanted<br>☐ Worried about how having a child might affect her life.<br>☐ Does not want others to know that she has had sexual intercourse or that she is pregnant<br>☐ Other<br>☐ None of the above<br>☐ No response
PNSME_702a. Besides this time, have you removed other pregnancies ?	(${self_abt_yn} = 'yes')<br>○ Yes<br>○ No<br>○ Do not know<br>○ No response
PNSME_702b. How many times?<br>*Enter -88 for do not know, -99 for no response.*	${abt_other_yn} = 'yes'<br>____________________
PNSME_703. In what year did this happen for the first time ?<br>*Enter 2020 for no response*	${abt_other_yn} = 'yes'<br>Year: ________________

	${consent_obtained}
Now we want to ask you some general questions about removing a pregnancy. Please provide your responses in the form of: strongly agree, agree, neither agree nor disagree, disagree, strongly disagree.<br>*Check box to confirm scrolled to bottom.*	
Press OK to continue.	○ OK

733. It is okay for a woman to remove a pregnancy if continuing the pregnancy puts her health at risk.	${consent_obtained}<br>○ Strongly agree<br>○ Agree<br>○ Neither agree nor disagree<br>○ Disagree<br>○ Strongly disagree<br>○ No response
734. It is okay for a woman to remove a pregnancy if the pregnancy is a result of rape.	${consent_obtained}<br>○ Strongly agree<br>○ Agree<br>○ Neither agree nor disagree<br>○ Disagree<br>○ Strongly disagree<br>○ No response
PNSME_734. It is acceptable for a woman to remove a pregnancy	${consent_obtained}

if this pregnancy was the result of incestuous sexual intercourse.	◯ Strongly agree<br>◯ Agree<br>◯ Neither agree nor disagree<br>◯ Disagree<br>◯ Strongly disagree<br>◯ No response
735. It is okay for a woman to remove a pregnancy if she cannot afford to raise another child.	${consent_obtained}<br>◯ Strongly agree<br>◯ Agree<br>◯ Neither agree nor disagree<br>◯ Disagree<br>◯ Strongly disagree<br>◯ No response
736. It is okay for a woman to remove a pregnancy if she does not want to have a child.	${consent_obtained}<br>◯ Strongly agree<br>◯ Agree<br>◯ Neither agree nor disagree<br>◯ Disagree<br>◯ Strongly disagree<br>◯ No response
737. A woman who removes a pregnancy brings shame to her family.	${consent_obtained}<br>◯ Strongly agree<br>◯ Agree<br>◯ Neither agree nor disagree<br>◯ Disagree<br>◯ Strongly disagree<br>◯ No response
738. A woman who removes a pregnancy should not tell anyone.	${consent_obtained}<br>◯ Strongly agree<br>◯ Agree<br>◯ Neither agree nor disagree<br>◯ Disagree<br>◯ Strongly disagree<br>◯ No response
PNSME_704a. Do you know if there is a law on abortion in Cote d'Ivoire ?	${consent_obtained}<br>◯ Yes<br>◯ No<br>◯ Do not know<br>◯ No response
PNSME_704b. Are there instances when it is legal to have an abortion in Côte d'Ivoire ?	${abt_law} = 'yes'<br>◯ Yes<br>◯ No<br>◯ Do not know<br>◯ No response
FLW_801. Thank you for the time you have kindly granted us.<br>We are very interested in hearing more about your pregnancy removal or period regulation experience, including your decision making around what method to use and where to go for services, among other things. Would you be willing to meet with another woman from the project at a later date to talk about this experience?	(${self_abt_yn} = 'yes') or (${self_reg_yn} = 'yes')<br>◯ Yes<br>◯ No<br>◯ No response
FLW_802. Do you own a phone?	${flw_willing} = 'yes'<br>◯ Yes<br>◯ No<br>◯ No response
FLW_803. Can I have your primary phone number in case we would like to follow up with you in the future?<br>*Enter an 8-digit number without the country code. Do not include spaces or dashes. Enter 0 for no response.*	${flw_number_yn} = 'yes'<br>--
FLW_804. Can you repeat the number again?<br>*Enter an 8-digit number without the country code. Do not include spaces*	(${flw_number_typed} != '') and (${flw_number_typed} != '0')

or dashes. Enter 0 for no response.

Thank the respondent for her time.

The respondent is finished, but there are still more questions for you to complete outside the home.

[illegible]

Location and Questionnaire result

095. Location

Take a GPS point near the entrance to the household. Record location when the accuracy is smaller than 6 m.

Always

096. How many times have you visited this household to interview this female respondent?

Always

- 1st time
- 2nd time
- 3rd time

097. In what language was this interview conducted?

009a = 1

- English
- French
- Arabic
- Baoule
- Senoufo
- Yacouba
- Agni
- Attie
- Guere
- Bete
- Dioula
- Abbey
- Mahou
- Wobe
- Lobi
- Other

099. Questionnaire Result

Record the result of the questionnaire.

Always

- Completed
- Not at home
- Postponed
- Refused
- Partly completed
- Incapacitated
