# Supplementary material for: Measurement of abortion safety using community-based surveys: Findings from three countries
Source: PLoS One. 2019 Nov 7;14(11):e0223146. doi: 10.1371/journal.pone.0223146 (PMC6837422; doi:10.1371/journal.pone.0223146)
Supplement: S7 Doc — (PDF) [file pone.0223146.s007.pdf]

S7 Doc.

CIR2-Female-Questionnaire-v6

001a. Êtes-vous dans le bon ménage ? ZD: [ZD saisie dans le Questionnaire Ménage] Numéro de Structure: [Structure saisie dans le Questionnaire Ménage] Numéro du Ménage: [Ménage saisi dans le Questionnaire Ménage]	Toujours<br>Oui<br>Non
002. Indiquez votre numéro d'identification ci-dessous.<br>*Veuillez saisir votre numéro d'identification*	002 = 0<br>--------------------------------
003b. Veuillez saisir la date et l'heure	003 = 0<br>Jour: --------------------------<br>Mois: --------------------------<br>Année: --------------------------

004a. Les informations suivantes proviennent du Questionnaire ménage. Veuillez les relire pour vous assurer que vous interrogez la bonne personne.	Toujours
Région: ${level1_unlinked}	--------------------------------
Département: ${level2_unlinked}	--------------------------------
Sous-prefecture: ${level3_unlinked}	--------------------------------
Zone de dénombrement: [ZD]	--------------------------------
Numéro de la Structure: [#]	-------------------------------
Numéro du Ménage: [#]	-------------------------------
004b. Les informations ci-dessus, sont-elles correctes?	004 = 0<br>Oui<br>Non

005. CONTRÔLE: La personne que vous allez interroger est [Nom de l'enquêtée]. Est-ce correct?<br>*Si le prénom est mal orthographié, sélectionnez "oui" et actualisez le prénom à la question 011.*<br>*S'il s'agit de la mauvaise personne, vous avez deux options:*<br>*(1) Sortir et ignorer les changements apportés à ce questionnaire. Ouvrir le bon questionnaire*<br>*Ou*<br>*(2) Trouver et interroger la personne dont le nom s'est affiché précédemment.*	Toujours<br>Oui<br>Non
006. La personne à interroger est-elle présente et disponible pour répondre au questionnaire aujourd'hui ?	Toujours<br>Oui<br>Non
007. Connaissez-vous l'enquêtée?	006 = 1<br>Oui, très bien<br>Oui, bien<br>Oui, mais pas très bien<br>Non
	006 = 1

008. L'enquêtée a-t-elle déjà participée à une enquête PMA2020 ?	◯ Oui<br>◯ Non<br>◯ Ne sait pas<br>◯ Pas de réponse
CONSENTEMENT ECLAIRE<br>*Veuillez trouver la femme entre 15 et 49 ans associée à ce Questionnaire Femme. L'entretien doit se dérouler en privé. Veuillez lire le message suivant à la personne interrogée:*	(${available} = 'yes') and (not(${unlinked}) or ${proceed_with_unlinked})
Bonjour. Je m'appelle _________________________________ et je travaille pour PARTENAIRE PAYS en collaboration avec AUTRES PARTENAIRES. Nous menons actuellement une enquête nationale sur plusieurs thèmes liés à la santé reproductive des femmes. Nous apprécierons beaucoup que vous fassiez partie de cette enquête. Les informations que nous collecterons aideront à informer le gouvernement à mieux planifier les services de santé. Le questionnaire prend généralement entre 15 et 20 minutes. Toutes les informations que vous nous donnerez seront strictement confidentielles et ne seront partagées avec personne d'autre que les membres de notre équipe. La participation à cette enquête est volontaire, et s'il y a une question à laquelle vous ne souhaitez pas répondre, faîtes-le moi savoir et je passerai à la suivante; ou vous pouvez également interrompre l'entretien à tout moment. Cependant, nous espérons que vous accepterez de participer à cette enquête car votre point de vue est important. Avant de continuer, avez-vous des questions sur cette enquête ?	(${available} = 'yes') and (not(${unlinked}) or ${proceed_with_unlinked})
Veuillez remettre un exemplaire papier du Formulaire de Consentement à la personne interrogée et lui expliquer. Puis, demandez : Puis-je commencer l'interview à présent ?	006 = 1<br>◯ Oui<br>◯ Non

	(${begin_interview}='yes')
009b. La signature de l'enquêtée<br>*Veuillez demander à l'enquêté(e) de signer ou de cocher la case en accord de leur participation.*	009a = 1<br>--------------------------------

010. Nom de l'enquêtrice. Veuillez saisir votre nom en tant que témoin du consentement. Vous avez auparavant saisi "[Nom de l'enquêtrice saisi dans le Questionnaire Ménage]."	${consent_obtained} and (${your_name_check} = 'no')<br>--------------------------------

Section 1 – Information sur l'Enquêtée, Statut Conjugal et Caractéristiques du Ménage

J'aimerais d'abord vous poser des questions concernant vos conditions de vie socio-économiques.

	${consent_obtained}
101. En quel mois et quelle année êtes-vous née ? L'âge indiqué sur le Questionnaire ménage est [ÂGE].	009a = 1
101. En quel mois et quelle année êtes-vous née ?	009a = 1
Mois:	◯ Janvier

	◯ Février<br>◯ Mars<br>◯ Avril<br>◯ Mai<br>◯ Juin<br>◯ Juillet<br>◯ Août<br>◯ Septembre<br>◯ Octobre<br>◯ Novembre<br>◯ Décembre<br>◯ Ne sait pas
Année:	Année:<br>_________________________

102. Quel âge aviez-vous à votre dernier anniversaire?	009a = 1<br>_______________________________
103. Quel est le plus haut niveau d'études que vous avez atteint?<br>*N'enregistrez que la scolarisation formelle. Ne pas enregistrer les cours de catéchisme, ni l'école coranique, ni les cours de courte durée.*	009a = 1<br>◯ Jamais scolarisée<br>◯ Primaire<br>◯ Secondaire<br>◯ Supérieur<br>◯ Pas de réponse
PNSME_101. Quelle est votre religion ?<br>*Prenez la religion que vous pratiquez actuellement.*	${consent_obtained}<br>◯ Musulmane<br>◯ Catholique<br>◯ Méthodiste<br>◯ Evangélique<br>◯ Autre religion chrétienne<br>◯ Animiste<br>◯ Autre religion<br>◯ Pas de religion<br>◯ Pas de réponse
PNSME_102. A quel groupe ethnique appartenez-vous?	${consent_obtained}<br>◯ Akan<br>◯ Mandé du sud<br>◯ Mandé du nord<br>◯ Gur<br>◯ Krou<br>◯ Autre ivoirien(ne)<br>◯ Non ivoirien(ne)<br>◯ Pas de réponse
104. Êtes-vous actuellement mariée ou vivez-vous avec un homme comme si vous étiez mariés ?<br>*Relancer: Si la réponse est "non", demandez si l'enquêtée est divorcée, séparée ou veuve.*	009a = 1<br>◯ Oui, actuellement mariée<br>◯ Oui, vit avec un homme<br>◯ Pas en union actuellement: divorcée / séparée<br>◯ Pas en union

	actuellement: veuve<br>◯ Non, jamais en union<br>◯ Pas de réponse
105. Avez-vous déjà été mariée ou avez-vous déjà vécu avec un homme seulement une fois ou plus d'une fois ?	104 ≠ 5<br>◯ Seulement une fois<br>◯ Plus d'une fois<br>◯ Pas de réponse

	(${marriage_history} = 'more_than_once')
106a. En quel mois et quelle année avez-vous commencé à vivre avec votre PREMIER mari/conjoint ?<br>*Sélectionnez « Ne sait pas » pour le mois et « 2020 » pour l'année afin d'indiquer « Pas de réponse. »*	105 = 2
Mois:	◯ Janvier<br>◯ Février<br>◯ Mars<br>◯ Avril<br>◯ Mai<br>◯ Juin<br>◯ Juillet<br>◯ Août<br>◯ Septembre<br>◯ Octobre<br>◯ Novembre<br>◯ Décembre<br>◯ Ne sait pas
Année:	Année:<br>_________________________

106b. CONTRÔLE: Selon la réponse que vous avez inscrit à 106a, l'enquêtée avait moins de 15 ans lors de son premier mariage. Avez-vous saisi la bonne réponse?	106a âge au marriage ≤15<br>◯ Oui<br>◯ Non

	(${marriage_history} = 'once') or (${marriage_history} = 'more_than_once')
107a. Maintenant j'aimerais vous demander à partir de quand vous avez commencé à vivre avec votre mari/conjoint ACTUEL ou LE PLUS RECENT. C'était en quel mois et en quelle année ?<br>*Sélectionnez « Ne sait pas » pour le mois et « 2020 » pour l'année afin d'indiquer « Pas de réponse. »*	105 = 1 or 2
Mois:	◯ Janvier<br>◯ Février<br>◯ Mars<br>◯ Avril<br>◯ Mai<br>◯ Juin<br>◯ Juillet<br>◯ Août

	◯ Septembre<br>◯ Octobre<br>◯ Novembre<br>◯ Décembre<br>◯ Ne sait pas
Année:	Année:<br>-------------------------

107b. CONTRÔLE: Selon la réponse que vous avez inscrit pour107a, l'enquêtée avait moins de 15 ans lors de son premier mariage. Avez-vous saisi la bonne réponse?	107a âge au mariage ≤15<br>◯ Oui<br>◯ Non
108. Votre mari/conjoint a t-il d'autres femmes ou vit-il avec d'autres femmes comme s'il était marié ?	104 = 1 or 2<br>◯ Oui<br>◯ Non<br>◯ Ne sait pas<br>◯ Pas de réponse
PNSME_103. Quel âge a votre mari/conjoint ?<br>*Saisissez -88 si « Ne sait pas » et -99 pour pas de réponse.*	((${marital_status} = 'currently_married') or (${marital_status} = 'currently_living_with_man'))<br>-------------------------------
PNSME_104. Quel est le plus haut niveau d'études atteint par votre mari/conjoint?	((${marital_status} = 'currently_married') or (${marital_status} = 'currently_living_with_man'))<br>◯ Jamais scolarisée<br>◯ Primaire<br>◯ Secondaire<br>◯ Supérieur<br>◯ Pas de réponse

Section 2 – Reproduction, grossesses et intentions de fécondité

J'aimerais d'abord vous poser des questions concernant toutes les naissances que vous avez eues au cours de votre vie.

200. J'aimerais maintenant vous poser des questions concernant toutes les naissances que vous avez eues durant votre vie. Avez-vous déjà donné naissance?	009a = 1<br>◯ Oui<br>◯ Non<br>◯ Pas de réponse
201. Combien de fois avez-vous donné naissance ?<br>*Saisir -99 si pas de réponse.*	200 = 1<br>-------------------------------

	(${birth_events} > 1)
205. Quand avez-vous accouché pour la PREMIERE fois ?<br>*Saisir la date de la PREMIERE naissance vivante. Celle-ci peut être calculée en remontant le temps à partir d'événements mémorables si nécessaire.*<br>*Sélectionnez « Ne sait pas » pour le mois et « 2020 » pour l'année afin d'indiquer « Pas de réponse. »*	201 > 1
Mois:	◯ Janvier<br>◯ Février

	Mars<br>Avril<br>Mai<br>Juin<br>Juillet<br>Août<br>Septembre<br>Octobre<br>Novembre<br>Décembre<br>Ne sait pas
Année:	Année:<br>_________________________

	(${birth_events} > 0)
206. Quand avez-vous accouché pour la DERNIERE fois ?<br>*Saisir la date de la DERNIERE naissance vivante. Celle-ci peut être calculée en remontant le temps à partir d'événements mémorables si nécessaire.*<br>*Sélectionnez « Ne sait pas » pour le mois et « 2020 » pour l'année afin d'indiquer « Pas de réponse. »*	201 > 1
Mois:	Janvier<br>Février<br>Mars<br>Avril<br>Mai<br>Juin<br>Juillet<br>Août<br>Septembre<br>Octobre<br>Novembre<br>Décembre<br>Ne sait pas
Année:	Année:<br>_________________________

210a. Êtes-vous actuellement enceinte?	009a = 1<br>Oui<br>Non<br>Pas sûre<br>Pas de réponse

	${pregnant} = 'yes'
210b. De combien de mois êtes-vous enceinte?	210a = 1
La naissance la plus recente etait le: [Date de la naissance la plus récente]	${recent_birth} != ''
#####<br>*Veuillez saisir le nombre de mois révolus. Saisir - 88 si ne sait pas et -99 si pas de réponse.*	______________________________

209. Quand vos dernières règles ont-elles	009a = 1

commencé ?<br>*Si vous choisissez jours, semaines, mois ou années, vous devrez saisir un chiffre sur le prochain écran. Saisir 0 jours pour aujourd'hui, pas 0 semaine/mois/ année*	◯ Il y a X jours<br>◯ Il y a X semaines<br>◯ Il y a X mois<br>◯ Il y a X années<br>◯ Ménopause / Hystérectomie<br>◯ Avant la dernière naissance<br>◯ Jamais eu de règles<br>◯ Pas de réponse
209a. Saisir X: [jours / semaines/ mois/ années]<br>*Saisir 0 jours pour aujourd'hui, pas 0 semaine/mois/année.*	(${menstrual_period} = 'days') or (${menstrual_period} = 'weeks') or (${menstrual_period} = 'month …<br>-------------------------------

	${ever_birth} = 'yes' or ${pregnant} = 'yes'
213a. J'aimerais maintenant vous poser une question sur votre dernière naissance.	201 > 0 ET 210a ≠ 1 201 > 0 ET 210a ≠ 1 201 > 0 ET 210a ≠ 1
213b. J'aimerais maintenant vous poser une question sur votre grossesse actuelle.	210a = 1
Quand vous êtes tombée enceinte, vouliez-vous être enceinte à ce moment là, est ce que vous vouliez avoir un enfant plus tard, ou est-ce que vous ne vouliez pas/plus avoir d'enfants du tout?	(201 > 1 ET 210a ≠ 1) OU (200 = 1 ET 201a = 1)
Quand vous êtes tombée enceinte, vouliez-vous être enceinte à ce moment là, est ce que vous vouliez avoir en enfant plus tard, ou est-ce que vous ne vouliez pas/plus avoir d'enfants du tout?	(201 = 1 ET 210a ≠ 1) ou (200 = 2 ET 201a = 1)
#####	◯ À ce moment<br>◯ Plus tard<br>◯ Pas d'enfants du tout<br>◯ Pas de réponse

Je voudrais maintenant vous poser quelques questions sur l'avenir.	${consent_obtained}
211a. Voudriez-vous avoir un enfant ou préférez-vous ne pas avoir d'enfant ?	210a ≠ 1<br>◯ Avoir un enfant<br>◯ Ne veux pas d'enfants<br>◯ Ne peut plus concevoir<br>◯ Indécise / Ne sait pas<br>◯ Pas de réponse
211a. Voudriez-vous avoir un autre enfant ou préférez-vous ne plus avoir d'enfant ?	210a ≠ 1<br>◯ Avoir un autre enfant<br>◯ Ne plus en avoir<br>◯ Ne peut plus concevoir<br>◯ Indécise / Ne sait pas<br>◯ Pas de réponse
211b. Après la naissance de l'enfant que vous attendez maintenant, voudriez-vous avoir un autre enfant, ou préfèreriez-vous ne plus avoir d'enfants?	210a = 1<br>◯ Avoir un autre enfant<br>◯ Ne plus en avoir<br>◯ Ne peut plus concevoir

	○ Indécise / Ne sait pas<br>○ Pas de réponse
212a. Combien de temps voudriez-vous attendre à partir de maintenant avant votre prochaine naissance?<br>*Si vous choisissiez mois ou années, vous devrez saisir un chiffre à l'écran suivant.*<br>*Choisissez « Années » si plus de 36 mois.*<br>*Assurez-vous que vous avez bien saisi le nombre de mois/années.*	211a = 1<br>○ X mois<br>○ X années<br>○ Bientôt / Maintenant<br>○ Dit ne pas pouvoir tomber enceinte<br>○ Autre<br>○ Ne sait pas<br>○ Pas de réponse
212b. Après la naissance de l'enfant que vous attendez maintenant, combien de temps voudriez-vous attendre avant la naissance d'un autre enfant?<br>*Si vous choisissiez mois ou années, vous devrez saisir un chiffre à l'écran suivant.*<br>*Choisissez « Années » si plus de 36 mois.*<br>*Assurez-vous que vous avez bien saisi le nombre de mois/années.*	211b = 1<br>○ X mois<br>○ X années<br>○ Bientôt / Maintenant<br>○ Dit ne pas pouvoir tomber enceinte<br>○ Autre<br>○ Ne sait pas<br>○ Pas de réponse
212c. Saisir: X [Mois OU Années] que vous aimeriez attendre:	212a OU 212b = 'mois' OR 'année'<br>________________________________

Section 3 – Contraception

J'aimerais maintenant vous poser des questions sur la planification familiale – les différents moyens ou méthodes qu'un couple peut utiliser pour retarder ou éviter une grossesse.

ODK affichera une image à l'écran pour quelques méthodes. Si l'enquêtée dit qu'elle n'a pas entendu parler de la méthode ou si elle hésite à répondre, veuillez lire la description de la méthode puis lui montrer l'image, si applicable.

301a. Avez-vous déjà entendu parler de la stérilisation féminine ? RELANCER : Les femmes peuvent avoir une opération pour ne plus avoir d'enfants.	009a = 1<br>○ Oui<br>○ Non<br>○ Pas de réponse
301b. Avez-vous déjà entendu parler de la stérilisation masculine ? RELANCER : Les hommes peuvent avoir une opération pour ne plus avoir d'enfants.	009a = 1<br>○ Oui<br>○ Non<br>○ Pas de réponse
301c. Avez-vous déjà entendu parler des implants contraceptifs ? RELANCER : Les femmes peuvent se faire insérer par un médecin ou une infirmière un bâtonnet ou plus sous la peau du haut du bras pour les empêcher de tomber enceinte, pendant une année ou plus<br>[implant_150x300.png]	009a = 1<br>○ Oui<br>○ Non<br>○ Pas de réponse
301d. Avez-vous déjà entendu parler des DIU / Stérilet?<br>RELANCER: Les femmes peuvent avoir un stérilet	009a = 1<br>○ Oui<br>○ Non

qu'un médecin ou une infirmière leur place dans l'utérus.<br>[IUD_150x300.png]	Pas de réponse
301e. Avez-vous déjà entendu parler des injectables ?<br>RELANCER : Les femmes peuvent avoir une injection faite par du personnel de santé qui les empêche de tomber enceinte pendant un mois ou plus.<br>[sayana_depo_150x300.jpg]	009a = 1<br>Oui<br>Non<br>Pas de réponse
301f. Avez-vous déjà entendu parler de la pilule (contraceptive) ?<br>RELANCER : Les femmes peuvent prendre une pilule chaque jour pour éviter de tomber enceinte.<br>[pill_150x300.png]	009a = 1<br>Oui<br>Non<br>Pas de réponse
301g. Avez-vous déjà entendu parler de la pilule du lendemain / contraception d'urgence?<br>RELANCER : Les femmes peuvent prendre pendant trois jours après des rapports sexuels non protégés des pilules spéciales qui les empêchent de tomber enceintes.	009a = 1<br>Oui<br>Non<br>Pas de réponse
301h. Avez-vous déjà entendu parler des condoms (préservatifs masculins)?<br>RELANCER : Les hommes peuvent mettre une capote en caoutchouc sur leur pénis avant les rapports sexuels.<br>[male_condom_150x300.png]	009a = 1<br>Oui<br>Non<br>Pas de réponse
301i. Avez-vous déjà entendu parler des condoms/préservatifs féminins?<br>RELANCER: Les femmes peuvent placer un fourreau dans leur vagin avant les rapports sexuels.<br>[female_condom_150x300.png]	009a = 1<br>Oui<br>Non<br>Pas de réponse
301j. Avez-vous déjà entendu parler du diaphragme / cape cervicale ?<br>RELANCER: Les femmes peuvent placer une rondelle de latex ou un petit "bonnet" sur le col de l'utérus avant les rapports sexuels.<br>[diaphragm_150x300.png]	009a = 1<br>Oui<br>Non<br>Pas de réponse
301k. Avez-vous déjà entendu parler des comprimés, la mousse, ou la gelée contraceptive ?<br>RELANCER : Les femmes peuvent mettre dans leur vagin une crème avant tout rapport sexuel afin de tuer les spermatozoïdes de l'homme. Cette crème peut être aussi mise sur le diaphragme.<br>[spermicide_150x300.png]	009a = 1<br>Oui<br>Non<br>Pas de réponse
301m. Avez-vous déjà entendu parler de la méthode d'allaitement exclusif ou MAMA?	009a = 1<br>Oui<br>Non<br>Pas de réponse
301n. Avez-vous déjà entendu parler de la méthode du rythme ? RELANCER : Pour éviter une grossesse, les femmes n'ont pas de rapports sexuels les jours du mois où elles pensent qu'elles peuvent tomber enceintes.	009a = 1<br>Oui<br>Non<br>Pas de réponse
301o. Avez-vous déjà entendu parler de la méthode	009a = 1

du retrait / coït interrompu ? RELANCER : Les hommes peuvent faire attention et se retirer avant l'éjaculation.	◯ Oui<br>◯ Non<br>◯ Pas de réponse
301p. Avez-vous entendu parler d'autres moyens ou méthodes qu'une femme ou un homme peut utiliser pour éviter une grossesse ?	`009a = 1`<br>◯ Oui<br>◯ Non<br>◯ Pas de réponse
302a. Est ce que vous, ou votre partenaire faites actuellement quelque chose ou utilisez-vous actuellement une méthode pour retarder ou éviter une grossesse?	`210a ≠ 1 ET 009a = 1 210a ≠ 1 ET 009a = 1 210a ≠ 1 ET 009a = 1 ≠ 1 ET 009a = 1 210a ≠ 1 ET 009a = 1`<br>◯ Oui<br>◯ Non<br>◯ Pas de réponse

	`${current_user} = 'yes'`
302b. Que faîtes-vous pour retarder ou éviter une grossesse? RELANCEZ Faites-vous quelque chose d'autre?<br>*Sélectionnez toutes les méthodes mentionnées. Assurez-vous de faire dérouler la liste JUSQU'EN BAS pour voire tous les choix possibles*	`302a = 1`<br>☐ Stérilisation féminine<br>☐ Stérilisation masculine<br>☐ Implants<br>☐ DIU / Stérilet<br>☐ Injectables<br>☐ Pilule<br>☐ Pilule du lendemain / Contraception d'urgence<br>☐ Préservatif masculin<br>☐ Préservatif féminin<br>☐ Diaphragme<br>☐ Mousse / Gelée spermicide<br>☐ Méthode des jours fixes / Collier du cycle<br>☐ MAMA<br>☐ Méthode du rythme<br>☐ Retrait<br>☐ Autres méthodes traditionnelles<br>☐ Pas de réponse

CALC CM. CALCULE D'ODK : METHODE ACTUELLE<br>CE TEXTE N'APPARAITRA PAS SUR L'ECRAN ODK.<br>*ODK identifiera la méthode la plus efficace que l'enquêtée utilise en sélectionnant la méthode le plus dessus de la liste.*	`302a = 1 ET 302b ≠ -99`<br>◯ Stérilisation féminine<br>◯ Stérilisation masculine<br>◯ Implants<br>◯ DIU / Stérilet<br>◯ Injectables<br>◯ Pilule<br>◯ Pilule du lendemain / Contraception d'urgence<br>◯ Préservatif masculin<br>◯ Préservatif féminin<br>◯ Diaphragme<br>◯ Mousse / Gelée

	spermicide<br>◯ Méthode des jours fixes / Collier du cycle<br>◯ MAMA<br>◯ Méthode du rythme<br>◯ Retrait<br>◯ Autres méthodes traditionnelles<br>◯ Pas de réponse
LCL_301. RELANCER : Est-ce que l'injection a été administrée par seringue ou par petite aiguille ?<br>*Veuillez montrer l'image à l'enquêtée.*<br>[sayana_depo_150x300.jpg]	CALC CM = 5<br>◯ Seringue<br>◯ Petite aiguille (Sayana Press)<br>◯ Pas de réponse
Votre mari/conjoint sait-il que vous utilisez [METHODE ACTUELLE] ?	302a = 1<br>◯ Oui<br>◯ Non<br>◯ Pas de réponse
302c. Votre mari/conjoint sait-il que vous utilisez planification familiale.	302a = -99<br>◯ Oui<br>◯ Non<br>◯ Pas de réponse
305a. Vous avez dit que vous n'utilisez pas de méthode contraceptive en ce moment. Pensez-vous que vous utiliserez une méthode contraceptive pour retarder ou éviter de tomber enceinte dans le futur ?	302a ≠1 ET 210a ≠1<br>◯ Oui<br>◯ Non<br>◯ Pas de réponse
305b. Pensez-vous que vous utiliserez une méthode contraceptive pour retarder ou éviter de tomber enceinte dans le futur ?	302a ≠1 ET 201a = 1<br>◯ Oui<br>◯ Non<br>◯ Pas de réponse
306a. Au cours des 12 derniers mois, avez-vous utilisé une méthode ou fait quelque chose pour retarder ou éviter une grossesse?	302a ≠1<br>◯ Oui<br>◯ Non<br>◯ Pas de réponse

	${recent_user} = 'yes'
306b. Quelles méthodes avez-vous utilisé le plus récemment? RELANCER: Y en a-t-il une autre?<br>*Sélectionnez la méthode la plus efficace (par ordre d'efficacité décroissante dans la liste). Faire dérouler la liste jusqu'en bas pour voir tous les choix possibles.*	306a = 1<br>◯ Stérilisation masculine<br>◯ Implants<br>◯ DIU / Stérilet<br>◯ Injectables<br>◯ Pilule<br>◯ Pilule du lendemain / Contraception d'urgence<br>◯ Préservatif masculin<br>◯ Préservatif féminin<br>◯ Diaphragme<br>◯ Mousse / Gelée spermicide<br>◯ Méthode des jours fixes / Collier du cycle

	○ MAMA<br>○ Méthode du rythme<br>○ Retrait<br>○ Autres méthodes traditionnelles<br>○ Pas de réponse

LCL_306c. RELANCER : Est-ce que l'injection a été administrée par seringue ou par petite aiguille ?<br>*Veuillez montrer l'image à l'enquêtée.*<br>[sayana_depo_150x300.jpg]	306b = 5<br>○ Seringue<br>○ Petite aiguille (Sayana Press)<br>○ Pas de réponse
307. Avant de commencer à utiliser [METHODE ACTUELLE / LA PLUS RECENTE], avez-vous discuté de la décision de retarder ou éviter la grossesse avec votre mari/conjoint ?	302a = 1 OU 306a = 1<br>○ Oui<br>○ Non<br>○ Ne sait pas<br>○ Pas de réponse
308. Diriez-vous que la décision d'utiliser la contraception était plus ou moins la votre, plus ou moins celle de votre mari/conjoint, ou bien avez-vous décidé ensemble ?	302a = 1<br>○ Prise principalement par l'enquêtée<br>○ Prise principalement par le mari/conjoint<br>○ Prise ensemble<br>○ Autre<br>○ Pas de réponse
308a. La dernière fois que vous avez obtenu [METHODE ACTUELLE / LA PLUS RECENTE], combien avez-vous dû payer, dont les frais pour la méthode elle-même, les produits ou les services nécessaires à son utilisation, ainsi que pour le transport ?<br>*Saisir tous les prix en FCFA. Zéro est une réponse possible. Saisir -88 si ne sait pas. Saisir -99 si pas de réponse.*	302a = 1 OU 306a = 1<br>________________

	${current_user} = 'yes'
309a. Depuis quand avez-vous commencé à utiliser [METHODE ACTUELLE / LA PLUS RECENTE], sans arrêter?<br>*Calculer la date en remontant le temps à partir d'évènements mémorables si nécessaire.*	302a = 1
Naissance la plus récente : [mm-aaaa]	${recent_birth} != ''
Mariage actuel : [mm-aaaa]	${husband_cohabit_start_recent} != ''
Mois:	○ Janvier<br>○ Février<br>○ Mars<br>○ Avril<br>○ Mai<br>○ Juin<br>○ Juillet<br>○ Août<br>○ Septembre

	○ Octobre<br>○ Novembre<br>○ Décembre<br>○ Ne sait pas
Année:	Année:<br>_________________________

	${recent_user} = 'yes'
309b. Quand avez-vous arrêté d'utiliser [METHODE ACTUELLE / LA PLUS RECENTE]?<br>*Veuillez saisir la date. Calculez la date en remontant le temps à partir d'événements mémorables si nécessaire.*<br>*Sélectionnez « Ne sait pas » pour le mois et « 2020 » pour l'année afin d'indiquer « Pas de réponse. »*	306a = 1
Mois:	○ Janvier<br>○ Février<br>○ Mars<br>○ Avril<br>○ Mai<br>○ Juin<br>○ Juillet<br>○ Août<br>○ Septembre<br>○ Octobre<br>○ Novembre<br>○ Décembre<br>○ Ne sait pas
Année:	Année:<br>_________________________

	${recent_user} = 'yes'
309c. Dans quel mois et quelle année avez-vous commencé à utiliser [METHODE ACTUELLE / LA PLUS RECENTE] avant de l'arrêter ?<br>*Calculer la date en remontant le temps à partir d'évènements mémorables si nécessaire.*<br>*Sélectionnez « Ne sait pas » pour le mois et « 2020 » pour l'année afin d'indiquer « Pas de réponse. »*	306a = 1
Naissance la plus récente : [mm-aaaa]	${recent_birth} != ''
Mariage actuel : [mm-aaaa]	${husband_cohabit_start_recent} != ''
Mois:	○ Janvier<br>○ Février<br>○ Mars<br>○ Avril<br>○ Mai<br>○ Juin<br>○ Juillet<br>○ Août<br>○ Septembre<br>○ Octobre

	◯ Novembre<br>◯ Décembre<br>◯ Ne sait pas
Année:	Année:<br>-------------------------

309d. CONTROLE : Pour être sûre que j'ai compris, vous avez utilisé [METHODE LA PLUS RECENTE] en continu entre [DATE DE COMMENCER] et [DATE D'ARRETER] sans arrêter, est-ce correct ?	`306a = 1`<br>◯ Oui<br>◯ Non
REVENIR EN ARRIERE AU DERNIER ECRAN ET RELANCER POUR DETERMINER LA PERIODE LA PLUS RECENTE DE L'UTILISATION EN CONTINUE.<br>*Suggestions pour relancer : - Quand avez-vous utilisé [METHODE] la dernière fois ? - Depuis combien de temps avez-vous utilisé [METHODE] sans arrêter ?*	`309d = 0`
310. Pourquoi avez-vous arrêté d'utiliser [METHODE LA PLUS RECENTE]?	`306a = 1`<br>☐ Peu de relations sexuelles / Conjoint absent<br>☐ Est tombée enceinte en l'utilisant<br>☐ Voulait tomber enceinte<br>☐ Mari / Conjoint en désaccord<br>☐ Voulait une méthode plus efficace<br>☐ Aucune méthode disponible<br>☐ Préoccupations de santé<br>☐ Peur des effets secondaires<br>☐ Manque d'accès / trop loin<br>☐ Coûte trop cher<br>☐ Utilisation peu pratique<br>☐ Fataliste<br>☐ Des difficultés à tomber enceinte / ménopausée<br>☐ Interfère avec les processus du corps<br>☐ Autre<br>☐ Ne sait pas<br>☐ Pas de réponse

	`${current_or_recent_user} and (${current_recent_method} != 'LAM') and (${current_recent_method} != …`
311a. Vous avez commencé à utiliser [METHODE ACTUELLE / PLUS RECENTE] en [DATE DE QF309a ou 309c]. Où l'avez-vous ou votre partenaire obtenue à ce moment-là ? Où l'avez-vous ou votre partenaire obtenue à ce moment-là ?	`(CALC_CM ≠ 14, 30, 31, 39, -99) OU (306b ≠ 14, 30, 31, 39, -99)`<br>◯ Hôpital gouvernemental<br>◯ Centre de santé

Faire défiler la liste jusqu'en bas pour voir toutes les réponses possibles	gouvernemental<br>◯ Clinique PF<br>◯ Stratégie / Clinique mobile (secteur public)<br>◯ Autre secteur public<br>◯ Hôpital/Clinique privée<br>◯ Pharmacie<br>◯ Médecin privé<br>◯ Stratégie / Clinique mobile (secteur privé)<br>◯ Agent de santé<br>◯ Autre secteur médical privé<br>◯ Boutique<br>◯ Institution religieuse<br>◯ Evènement communautaire<br>◯ Ami(e)/Parent(e)<br>◯ Agent de santé communautaire<br>◯ Vendeur de la rue<br>◯ Autre<br>◯ Ne sait pas<br>◯ Pas de réponse
311a. Où votre mari/conjoint ou vous-mêmes avez-vous obtenu ${current_recent_label} quand vous avez commencé à utiliser cette méthode?<br>*Faire défiler la liste jusqu'en bas pour voir toutes les réponses possibles*	(CALC_CM ≠ 14, 30, 31, 39, -99) OU (306b ≠ 14, 30, 31, 39, -99)<br>◯ Hôpital gouvernemental<br>◯ Centre de santé gouvernemental<br>◯ Clinique PF<br>◯ Stratégie / Clinique mobile (secteur public)<br>◯ Autre secteur public<br>◯ Hôpital/Clinique privée<br>◯ Pharmacie<br>◯ Médecin privé<br>◯ Stratégie / Clinique mobile (secteur privé)<br>◯ Agent de santé<br>◯ Autre secteur médical privé<br>◯ Boutique<br>◯ Institution religieuse<br>◯ Evènement communautaire<br>◯ Ami(e)/Parent(e)<br>◯ Agent de santé communautaire<br>◯ Vendeur de la rue

	Autre<br>Ne sait pas<br>Pas de réponse
312a. Quand vous avez obtenu [METHODE ACTUELLE / PLUS RECENTE], l'agent de santé ou de planification familiale vous a-t-il parlé des effets secondaires ou des problèmes que vous pourriez avoir en utilisant une méthode pour retarder ou éviter une grossesse ?	311a ≠ .<br>Oui<br>Non<br>Pas de réponse
312b. Vous a-t-on dit ce qu'il fallait faire si vous aviez ces effets secondaires ou des problèmes ?	312a = 1<br>Oui<br>Non<br>Pas de réponse
313. À ce moment là, vous a-t-on parlé d'autres méthodes que [METHODE ACTUELLE / PLUS RECENTE] que vous pourriez utiliser?	311a ≠ . OU 311b ≠ .<br>Oui<br>Non<br>Ne sait pas<br>Pas de réponse
314a. Pendant cette visite, avez-vous obtenu la méthode que vous souhaitiez pour éviter ou retarder une grossesse ?	311a ≠ .<br>Oui<br>Non<br>Pas de réponse
314c. Pourquoi n'avez-vous pas obtenu la méthode que vous souhaitiez?	314a = 0<br>Rupture de stock ce jour-là<br>Méthode pas disponible<br>Prestataire pas formé pour fournir cette méthode<br>Prestataire recommandait une autre méthode<br>Pas éligible pour cette méthode<br>A décidé de ne pas utiliser de méthode<br>Trop cher<br>Autre<br>Pas de réponse
315a. Pendant cette visite, qui a pris la décision concernant sur la méthode adoptée ?	311a ≠ .<br>Enquêtée seule<br>Prestataire<br>Partenaire<br>Enquêtée et prestataire<br>Enquêtée et partenaire<br>Autre<br>Ne sait pas<br>Pas de réponse
315b. Qui a pris la décision d'utiliser la méthode du rythme ?	311b ≠ .<br>Enquêtée seule<br>Prestataire<br>Partenaire

	○ Enquêtée et prestataire<br>○ Enquêtée et partenaire<br>○ Autre<br>○ Ne sait pas<br>○ Pas de réponse
315b. Qui a pris la décision d'utiliser MAMA ?	311b ≠ .<br>○ Enquêtée seule<br>○ Prestataire<br>○ Partenaire<br>○ Enquêtée et prestataire<br>○ Enquêtée et partenaire<br>○ Autre<br>○ Ne sait pas<br>○ Pas de réponse
316. Retourneriez-vous voir ce prestataire de santé? Prestataire : [Type de prestataire sélectionné à 311a ou 311b]	311a ≠ 35 ou 96<br>○ Oui<br>○ Non<br>○ Ne sait pas<br>○ Pas de réponse
317. Recommanderiez-vous ce prestataire à un/e ami(e) ou un membre de votre famille ? Prestataire : [Type de prestataire sélectionné à 311a ou 311b]	311 a ≠ 34 ou 96<br>○ Oui<br>○ Non<br>○ Ne sait pas<br>○ Pas de réponse
SW_1a. Juste avant de commencer à utiliser [METHODE ACTUELLE/ RECENTE] en [MOIS/ANNEE], faisiez-vous quelque chose ou utilisiez-vous une méthode pour retarder ou éviter de tomber enceinte ?	302a = 1 OU 306a = 1<br>○ Oui<br>○ Non<br>○ Pas de réponse
SW_1b. Quelle méthode utilisiez-vous ?	SW_1a = 1<br>○ Stérilisation masculine<br>○ Implants<br>○ DIU / Stérilet<br>○ Injectables<br>○ Pilule<br>○ Pilule du lendemain / Contraception d'urgence<br>○ Préservatif masculin<br>○ Préservatif féminin<br>○ Diaphragme<br>○ Mousse / Gelée spermicide<br>○ Méthode des jours fixes / Collier du cycle<br>○ MAMA<br>○ Méthode du rythme<br>○ Retrait<br>○ Autres méthodes traditionnelles<br>○ Pas de réponse

PP_1. Depuis la naissance de votre enfant le [DATE DE LA NAISSANCE LA PLUS RECENTE], avez-vous fait quelque chose ou utilisé une méthode pour retarder ou éviter de tomber enceinte ?	enfant né dans les deux dernières années ET 302a ≠ 1<br>○ Oui<br>○ Non<br>○ Pas de réponse
PP_2. Combien de temps après la naissance le [DATE DE LA NAISSANCE LA PLUS RÉCENTE] avez-vous commencé à faire quelque chose ou à utiliser une méthode ?<br>*Saisissez 0 jour pour aujourd'hui Vous saisirez un numéro pour X sur l'écran suivant*	PP_1 = 1 OU (302a = 1 ET enfant né dans les deux dernières années)<br>○ X jours<br>○ X semaines<br>○ X mois<br>○ X ans<br>○ Pas de réponse
PP_2. Saisir [METHODE].<br>*Si aujourd'hui, saisissez 0 jour uniquement, pas 0 semaines/mois/années*	${pp_method_units} = 'days' or ${pp_method_units} = 'weeks' or ${pp_method_units} = 'months' or ${ …<br>________________________________
PP_3. Quelle était la méthode ?	PP_2 ≠ .<br>○ Stérilisation féminine<br>○ Stérilisation masculine<br>○ Implants<br>○ DIU / Stérilet<br>○ Injectables<br>○ Pilule<br>○ Pilule du lendemain / Contraception d'urgence<br>○ Préservatif masculin<br>○ Préservatif féminin<br>○ Diaphragme<br>○ Mousse / Gelée spermicide<br>○ Méthode des jours fixes / Collier du cycle<br>○ MAMA<br>○ Méthode du rythme<br>○ Retrait<br>○ Autres méthodes traditionnelles<br>○ Pas de réponse
LCL_PP. RELANCER : Est-ce que l'injection a été administrée avec une seringue ou une petite aiguille ?<br>*Veuillez montrer l'image à l'enquêtée.*<br>[sayana_depo_150x300.jpg]	PP_3 = 5<br>○ Seringue<br>○ Petite aiguille (Sayana Press)<br>○ Pas de réponse
319. Avez-vous déjà utilisé quelque chose ou essayé par divers moyens de retarder ou d'éviter une grossesse ?	306a ≠ 1 OU 302a ≠ 1<br>○ Oui<br>○ Non<br>○ Pas de réponse

320. Quel âge aviez-vous lorsque vous avez essayé une méthode vous permettant de retarder ou éviter une grossesse pour la première fois ? L'enquêtée a indiqué qu'elle avait eu [ÂGE] ans lors de son dernier anniversaire.<br>*Saisir l'âge en année. Saisir -88 si ne sait pas ou -99 si pas de réponse. L'âge indiqué ne peut pas être inférieur à 9 ans.*	302a = 1 OU 306a =1 OU 319 = 1<br>________________
321. Combien d'enfants en vie aviez-vous à ce moment-là ? Notez: L'enquêtée a indiqué qu'elle avait donné naissance [nombre des naissances vivantes] fois à 201.<br>*Saisir -99 si pas de réponse.*	Âge à 320 ≥ 9 ET 200 = 1<br>________________

	${fp_ever_used} = 'yes'
322. Quelle méthode avez-vous utilisé pour retarder ou éviter une grossesse la première fois?<br>*Ne lisez pas les réponses. Descendre jusqu'en bas pour voir tous les choix possibles.*	319 = 1<br>◯ Stérilisation féminine<br>◯ Stérilisation masculine<br>◯ Implants<br>◯ DIU / Stérilet<br>◯ Injectables<br>◯ Pilule<br>◯ Pilule du lendemain / Contraception d'urgence<br>◯ Préservatif masculin<br>◯ Préservatif féminin<br>◯ Diaphragme<br>◯ Mousse / Gelée spermicide<br>◯ Méthode des jours fixes / Collier du cycle<br>◯ MAMA<br>◯ Méthode du rythme<br>◯ Retrait<br>◯ Autres méthodes traditionnelles<br>◯ Pas de réponse

LCL_322a. RELANCER : Est-ce que l'injection a été administrée par seringue ou par petite aiguille ?<br>*Veuillez montrer l'image à l'enquêtée.*<br>[sayana_depo_150x300.jpg]	322 = 5<br>◯ Seringue<br>◯ Petite aiguille (Sayana Press)<br>◯ Pas de réponse
322a. Avez-vous utilisé la contraception d'urgence à un moment ou un autre au cours des 12 derniers mois ?<br>RELANCER: Comme mesure d'urgence après un rapport sexuel non protégé, les femmes peuvent prendre une pilule spéciale à n'importe quel moment dans les 3 à 5 jours suivant le rapport sexuel pour éviter de tomber enceintes.	302b ≠ contraception d'urgence OU 306b ≠ 8<br>◯ Oui<br>◯ Non<br>◯ Pas de réponse

	((${current_user} = 'no')) and (((${more_children_none} = 'no_children') or …
	302a = 0 ET ((212a ou 212b

323a. Vous avez dit que vous ne souhaitiez un enfant bientôt avoir d'enfant et que vous n'utilisez pas de méthode contraceptive pour éviter une grossesse.	> 2 ans) OU (211a ou 211b = 2))
323a. Vous avez dit que vous ne souhaitiez un enfant bientôt avoir d'enfant et que vous n'utilisez pas de méthode contraceptive pour éviter une grossesse.	302a = 0 ET ((212a ou 212b > 2 years) OU (211a ou 211b = 2))
323a. Vous avez dit que vous ne souhaitiez pas avoir d'enfant et que vous n'utilisez pas de méthode contraceptive pour éviter une grossesse.	302a = 0 ET ((212a ou 212b > 2 ans) OU (211a ou 211b = 2))
323a. Vous avez dit que vous ne souhaitiez plus avoir d'enfant et que vous n'utilisez pas de méthode contraceptive pour éviter une grossesse.	302a = 0 ET ((212a ou 212b > 2 ans) OU (211a ou 211b = 2))
Pouvez-vous me dire pourquoi vous n'utilisez pas une méthode?<br>*SELECTIONNER TOUTES LES RESPONSES MENTIONNEES.*<br>*Vous ne pouvez pas sélectionner "Pas mariée" si la réponse à 104 est "Oui, actuellement mariée".*<br>*Faire défiler la liste jusqu'en bas pour voir toutes les réponses possibles.*	☐ Pas mariée<br>☐ Peu/pas de rapports sexuels<br>☐ Ménopause / Hystérectomie<br>☐ Infertile / peu fertile<br>☐ N'a pas eu de règles depuis la dernière naissance<br>☐ Allaitement<br>☐ Conjoint absent depuis plusieurs jours<br>☐ S'en remet à Dieu / Fataliste<br>☐ Enquêtée opposée<br>☐ Mari / conjoint opposé<br>☐ Autres personnes opposées<br>☐ Prohibition religieuse<br>☐ Ne connaît aucune méthode<br>☐ Ne sait pas où se procurer<br>☐ Peur des effets secondaires<br>☐ Préoccupations de santé<br>☐ Manque d'accès / Trop loin<br>☐ Coûte trop cher<br>☐ Méthode préférée non disponible<br>☐ Aucune méthode disponible<br>☐ Utilisation peu pratique<br>☐ Interfère avec les processus du corps<br>☐ Autre<br>☐ Ne sait pas<br>☐ Pas de réponse

323b. Diriez-vous que la décision de ne pas utiliser la contraception était plus ou moins la vôtre, plus ou moins celle de votre mari/conjoint, ou bien avez-vous décidé ensemble?	302a ≠ 1<br>○ Prise principalement par l'enquêtée<br>○ Prise principalement par le mari/conjoint<br>○ Prise ensemble<br>○ Autre<br>○ Pas de réponse
324. Au cours des 12 derniers mois, est-ce que vous avez reçu la visite d'un agent de santé qui vous a parlé de planification familiale ?	009a = 1<br>○ Oui<br>○ Non<br>○ Pas de réponse
325a. Au cours des 12 derniers mois, êtes-vous allée dans un établissement de santé pour recevoir des soins pour vous-même?<br>*Cela peut être pour n'importe quel service*	009a = 1<br>○ Oui<br>○ Non<br>○ Pas de réponse
325a. Au cours des 12 derniers mois, êtes-vous allée dans un établissement de santé pour recevoir des soins pour vous-même ou pour vos enfants?<br>*Cela peut être pour n'importe quel service*	009a = 1<br>○ Oui<br>○ Non<br>○ Pas de réponse
325b. Est ce qu'un membre de l'établissement de santé vous a parlé de méthodes de planification familiale ?	325a = 1<br>○ Oui<br>○ Non<br>○ Pas de réponse

${consent_obtained}

Au cours des derniers mois, avez-vous:

	Oui	Non	Pas de réponse
326a. Entendu parler du planning familial à la radio?	○	○	○
326b. Vu quelque chose sur le planning familial à la télévision?	○	○	○
326c. Lu quelque chose à propos du planning familial dans un magazine ou dans les journaux?	○	○	○
326d. Reçu un message vocale ou par message à propos de la planification familiale sur un téléphone portable?	○	○	○

Section 4 – L'histoire sexuelle

VERIFIEZ LA PRESENCE D'AUTRES PERSONNES AVANT DE CONTINUER. FAITES TOUS LES EFFORTS POUR ASSURER L'ISOLEMENT DE L'ECOUTE DES AUTRES.

À présent, je souhaiterais vous poser quelques questions sur vos rapports sexuels pour mieux comprendre certaines questions vitales importantes. Je voudrais vous assurer encore une fois que vos réponses sont complètement confidentielles et ne seront communiquées à personne. Si nous rencontrons une question à laquelle vous ne souhaitez pas répondre, faites-le moi savoir et nous passerons à la suivante.	${consent_obtained}

	${consent_obtained}
401a. Quel âge aviez-vous quand vous avez eu des rapports sexuels pour la première fois?	309a = 1
âge: [ÂGE]	
Naissances vivantes: [NOMBRE DE NAIISANCE VIVANTE]	${birth_events} > 0
L'enquetee est enceinte.	${pregnant} = 'yes'
Saisir l'âge en années<br>*Saisir -77 si elle n'a jamais eu de rapport sexuel. Saisir -88 si ne sait pas. Saisir -99 si pas de réponse*	------------------------------

	((${age_at_first_sex} >= 0) or (${age_at_first_sex} =-88) or (${age_at_first_sex} =-99))
402. Quand avez-vous eu des rapports sexuels pour la dernière fois ?	401a ≠ -77

402. Saisir X: [# jours / semaines / mois / années].<br>*Si aujourdhui, saisir seulement 0 jours (pas zéro semaines / mois ou/ années).*<br>*Doit être cohérent avec l'âge du premier rapport sexuel et le statut de grossesse.*	401a ≠ -77<br>------------------------------
LCL_403. Selon vous, si vous avez une fois des rapports sexuels sans utiliser la contraception, est-ce que vous tomberez enceinte?<br>*Lire les choix de réponse à voix haute.*	${consent_obtained}<br>○ Oui, sûrement<br>○ Oui, peut-être<br>○ Non, peut-être<br>○ Non, sûrement<br>○ Ne sait pas<br>○ Pas de réponse
LCL_404. Selon vous, si vous avez des rapports sexuels réguliers, disons deux fois par semaine, pendant un an sans utiliser la contraception, est-ce que vous tomberez enceinte?<br>*Lire les choix de réponse à voix haute.*	${consent_obtained}<br>○ Oui, sûrement<br>○ Oui, peut-être<br>○ Non, peut-être<br>○ Non, sûrement<br>○ Ne sait pas<br>○ Pas de réponse

Section 6 – Hygiène menstruelle

Maintenant, je voudrais vous poser quelques questions sur votre hygiène menstruelle. Ceci inclut l'utilisation des matériels absorbants ; l'accès à un endroit privé, propre, et en sécurité ; le lavage du corps si nécessaire ; et l'endroit pour jeter des matériels utilisés.

602a. Quel endroit utilisez-vous le plus souvent pour changer vos serviettes hygiéniques, les chiffons ou d'autres matériels hygiéniques ?	(209 ≤ 90 jours, 13 semaines ou ≤ 3 mois)<br>○ Des toilettes à chasse d'eau<br>○ Latrines ventilées améliorées<br>○ Latrines à fosse avec dalle<br>○ Latrines à fosse sans dalle

	Un seau<br>Des toilettes à compostage<br>Des toilettes ou des latrines suspendues<br>Douche<br>Là où on dort/une chambre<br>La parcelle<br>Pas de toilettes / buissons / nature<br>Autre<br>Pas de réponse
L'ENDROIT PRINCIPAL : [L'ENDROIT PRINCIPAL DE 602a] 602b. En prenant soins de votre hygiène menstruelle, cet endroit était-il :<br>*Veuillez lire chaque modalité dans la liste.*	`601 ≠ -99 ni nul ET 602 ≠ -99 ni nul`<br>Propre ?<br>Privé ?<br>En sécurité ?<br>Peut être fermé à clé ?<br>Stocké avec de l'eau ?<br>Stocké avec du savon ?<br>Aucuns des éléments ci-dessus<br>Pas de réponse
603. Lors de vos dernières règles, qu'est ce que vous avez utilisé pour la collecte ou l'absorption du sang menstruel ? RELANCER : Autres choses ?<br>*Ne lisez pas les options à voix haute. Veuillez sélectionner tous les éléments pertinents.*	`(209 ≤ 90 jours, 13 semaines ou ≤ 3 mois)`<br>Serviette hygiénique jetable (commerciale)<br>Serviette hygiénique réutilisable<br>Nouveau tissu/pagne<br>Vieux tissu/pagne<br>Laine de coton<br>Couche<br>Tampon<br>Papier toilette<br>Sous-vêtement seul<br>Seau<br>Autre<br>N'a pas utilisé de matériel<br>Pas de réponse
604a. Avez-vous lavé et réutilisé les serviettes hygiéniques, les chiffons, ou d'autres matériels sanitaires lors de vos dernières règles ?	`603 = serviette hygiénique réutilisable, vieux tissu/pagne, sous-vêtement seul, sceau`<br>Oui<br>Non<br>Pas de réponse
604b. Lors de vos dernières règles, les matériels sanitaires que vous avez lavé et réutilisé étaient-ils	`604a = 1`<br>Oui

complètement séchés chaque fois avant de les réutiliser ?	○ Non<br>○ Pas de réponse
605. Vous avez mentionné que vous avez utilisé [ODK affichera les réponses de 603] lors de vos dernières règles. Où avez-vous jeté ces matériels après utilisation? RELANCER : Autres endroits ?<br>*Ne lisez pas les options à voix haute. Veuillez sélectionner tous les éléments pertinents.*	`603 = disposable sanitary pad, new cloth, cotton wool, diaper, tampons, toilet paper, or other OU 604a =0`<br>☐ Toilette à chasse d'eau<br>☐ Latrine<br>☐ Poubelle/sac-poubelle<br>☐ Ils étaient brûlés<br>☐ Buissons/nature<br>☐ Autre<br>☐ Pas de réponse
606a. Hormis le ménage de votre propre maison, avez-vous travaillé le mois dernier ?	`(209 ≤ 90 jours, 13 semaines ou ≤ 3 mois)`<br>○ Oui<br>○ Non<br>○ Pas de réponse
606b. Est-ce que vous n'avez pas pu aller au travail le mois dernier parce que vous aviez vos règles ?	`606a = 1`<br>○ Oui<br>○ Non<br>○ Pas de réponse
607a. Etes-vous allée à l'école à un moment ou un autre au cours des 12 derniers mois ?	`102 = 15 - 24 ET 103 ≠ jamais`<br>○ Oui<br>○ Non<br>○ Pas de réponse
607b. Est-ce que vous n'avez pas pu aller à l'école à un moment ou un autre dans les 12 derniers mois parce que vous étiez en période de menstruation ?	`607a = 1`<br>○ Oui<br>○ Non<br>○ Pas de réponse

Section 7.1 – Confidentes

701. J'aimerais maintenant vous poser quelques questions sur vos plus proches amies et membres de votre famille filles, c'est- à dire des amies, des sœurs, des cousines, votre mère ou des tantes, à qui vous confiez vos secrets et qui vous confient les leurs. Combien d'amies et membres de votre famille filles de ce genre avez-vous en Côte d'Ivoire qui ont entre 15 et 49 ans ?<br>*Saisir -88 pour Ne sait pas, -99 pour Pas de réponse.*	`${consent_obtained}`<br>------------------------------
702a. Pensez à votre amie ou membre de votre famille la plus proche en Côte d'Ivoire qui a entre 15 et 49 ans, à qui vous confiez vos secrets et qui vous confient les siennes. Pour nous référer à cette personne plus facilement, pourriez-vous me donner un faux nom ?	`${friend_count} > 0`<br>------------------------------
703a. Quel âge a ${friend1_name} lors de son dernier anniversaire ?<br>*Saisir -88 pour Ne sait pas, -99 pour Pas de réponse.*	`${friend_count} > 0`<br>------------------------------
704a. Quel est le plus haut niveau d'étude atteint	`${friend_count} > 0`

par ${friend1_name} ?	○ Jamais scolarisée<br>○ Primaire<br>○ Secondaire<br>○ Supérieur<br>○ Pas de réponse
702b. Pensez maintenant à votre deuxième amie ou membre de votre famille la plus proche en Côte d'Ivoire qui a entre 15 et 49 ans et à qui vous confiez vos secrets, et qui vous confie les siennes. Pour nous référer à cette personne plus facilement, pouvez-vous me donner un faux nom ?	${friend_count} > 1<br>-------------------------------
703b. Quel âge a ${friend2_name} lors de son dernier anniversaire ?<br>*Saisir -88 pour Ne sait pas, -99 pour Pas de réponse.*	${friend_count} > 1<br>-------------------------------
704b. Quel est le plus haut niveau d'études atteint par ${friend2_name} ?	${friend_count} > 1<br>○ Jamais scolarisée<br>○ Primaire<br>○ Secondaire<br>○ Supérieur<br>○ Pas de réponse

Section 7.2 – Avortement

VERIFIEZ LA PRESENCE D'AUTRES PERSONNES AVANT DE CONTINUER. FAITES TOUS LES EFFORTS POUR ASSURER L'ISOLEMENT DE L'ECOUTE DES AUTRES.

La série de questions suivante porte sur les choses que les femmes dans votre communauté font pour faire passer une grossesse. Ceci est un phénomène qui existe en Côte d'Ivoire et nous souhaitons simplement mieux comprendre ce que les femmes font pour faire passer une grossesse. Je voudrais vous rappeler que ces questions sont entièrement confidentielles et anonymes. Vos réponses personnelles ne seront communiquées à personne. S'il y a une question à laquelle vous ne souhaitez pas répondre, faites-le moi simplement savoir et je passerai à la question suivante.<br>*Appuyer sur OK pour continuer.*	${consent_obtained}<br>☐ OK
705. Parfois les femmes peuvent tomber enceintes quand elles ne le souhaitent pas, et font alors quelque chose pour faire passer la grossesse. Est-ce que cela est fréquent dans votre milieu où vous habitez?<br>*Lire les choix de réponse à voix haute.*	${consent_obtained}<br>○ Très fréquent<br>○ Assez fréquent<br>○ Peu fréquent<br>○ Pas du tout<br>○ Ne sait pas<br>○ Pas de réponse
706a. Dans le milieu où vous habitez actuellement, que peuvent faire les femmes pour faire passer une grossesse quand elles sont enceintes, ou elles pensent qu'elles sont enceintes ? Rien d'autre ?<br>*Ne pas lire les choix de réponse à voix haute. Sélectionnez toutes les réponses qui conviennent.*	${consent_obtained} and (${abt_common} != 'not_common')<br>☐ Intervention chirurgicale (curetage, AMIU, etc.)<br>☐ Comprimés appelées mifepristone ou misoprostol (Cytotec)<br>☐ Médicaments qu'on

	prend quand on a de la fièvre, comme des antibiotiques ou un médicament anti-palu<br>☐ Autres comprimés<br>☐ Méthodes traditionnelles (plantes, potions, décoctions, etc.)<br>☐ Produits industriels ingérés (eau de javel, coca-nescafé, etc.)<br>☐ Insertion de matériel dans le vagin (tige, boule d'herbes, kanigban, etc.)<br>☐ Autre<br>☐ Ne sait pas<br>☐ Pas de réponse
706b. Neanmoins, selon vous, qu'est ce que les femmes dans le milieu où vous habitez pourraient faire pour faire passer une grossesse quand elles sont enceintes, ou elles pensent qu'elles sont enceintes ? Rien d'autre ?	`${consent_obtained} and (${abt_common} = 'not_common')`<br>☐ Intervention chirurgicale (curetage, AMIU, etc.)<br>☐ Comprimés appelées mifepristone ou misoprostol (Cytotec)<br>☐ Médicaments qu'on prend quand on a de la fièvre, comme des antibiotiques ou un médicament anti-palu<br>☐ Autres comprimés<br>☐ Méthodes traditionnelles (plantes, potions, décoctions, etc.)<br>☐ Produits industriels ingérés (eau de javel, coca-nescafé, etc.)<br>☐ Insertion de matériel dans le vagin (tige, boule d'herbes, kanigban, etc.)<br>☐ Autre<br>☐ Ne sait pas<br>☐ Pas de réponse
707. Quel est le moyen le plus courant ?<br>*Sélectionner une seule réponse*	`count-selected(${abt_ways}) > 1`<br>○ Intervention chirurgicale (curetage, AMIU, etc.)<br>○ Comprimés appelées mifepristone ou misoprostol (Cytotec)<br>○ Médicaments qu'on prend quand on a de la fièvre, comme des

	antibiotiques ou un médicament anti-palu<br>◯ Autres comprimés<br>◯ Méthodes traditionnelles (plantes, potions, décoctions, etc.)<br>◯ Produits industriels ingérés (eau de javel, coca-nescafé, etc.)<br>◯ Insertion de matériel dans le vagin (tige, boule d'herbes, kanigban, etc.)<br>◯ Autre<br>◯ Ne sait pas<br>◯ Pas de réponse<br>`selected(${abt_ways}, filter_list) or (filter_list = 'always')`
708. Où vont les femmes pour avoir une intervention chirurgicale pour faire passer la grossesse ? Y a t-il d'autres endroits ?<br>*Ne pas lire les choix de réponse à voix haute. Sélectionnez toutes les réponses qui conviennent.*	`(selected(${abt_ways},'surgery'))`<br>☐ Hôpital gouvernemental<br>☐ Centre de santé gouvernemental<br>☐ Clinique PF<br>☐ Stratégie / Clinique mobile (secteur public)<br>☐ Autre secteur public<br>☐ Hôpital/Clinique privée<br>☐ Pharmacie<br>☐ Médecin privé<br>☐ Stratégie / Clinique mobile (secteur privé)<br>☐ Agent de santé<br>☐ Autre secteur médical privé<br>☐ Boutique<br>☐ Institution religieuse<br>☐ Evènement communautaire<br>☐ Ami(e)/Parent(e)<br>☐ Agent de santé communautaire<br>☐ Vendeur de la rue<br>☐ Autre<br>☐ Ne sait pas<br>☐ Pas de réponse
709.Quel est l' endroit le plus courant ?<br>*Sélectionner une seule réponse*	`count-selected(${abt_surg_where}) > 1`<br>◯ Hôpital gouvernemental<br>◯ Centre de santé gouvernemental<br>◯ Clinique PF

- Stratégie / Clinique mobile (secteur public)
- Autre secteur public
- Hôpital/Clinique privée
- Pharmacie
- Médecin privé
- Stratégie / Clinique mobile (secteur privé)
- Agent de santé
- Autre secteur médical privé
- Boutique
- Institution religieuse
- Evènement communautaire
- Ami(e)/Parent(e)
- Agent de santé communautaire
- Vendeur de la rue
- Autre
- Ne sait pas
- Pas de réponse

`selected(${abt_surg_where}, filter_list) or (filter_list = 'always')`

---

710. Où est-ce que les femmes obtiennent les médicaments pour faire passer une grossesse ? Y a t-il d'autres endroits ?

Ne pas lire les choix de réponse à voix haute. Sélectionnez toutes les réponses qui conviennent.

`(selected(${abt_ways}, 'pills_abortion')) or (selected(${abt_ways}, 'pills_fever')) or (selected($ …`

- [ ] Hôpital gouvernemental
- [ ] Centre de santé gouvernemental
- [ ] Clinique PF
- [ ] Stratégie / Clinique mobile (secteur public)
- [ ] Autre secteur public
- [ ] Hôpital/Clinique privée
- [ ] Pharmacie
- [ ] Médecin privé
- [ ] Stratégie / Clinique mobile (secteur privé)
- [ ] Agent de santé
- [ ] Autre secteur médical privé
- [ ] Boutique
- [ ] Institution religieuse
- [ ] Evènement communautaire
- [ ] Ami(e)/Parent(e)
- [ ] Agent de santé communautaire
- [ ] Vendeur de la rue

	☐ Autre<br>☐ Ne sait pas<br>☐ Pas de réponse
711. Quel est l'endroit le plus courant ?<br>*Sélectionner une seule réponse*	`count-selected(${abt_meds_where}) > 1`<br>○ Hôpital gouvernemental<br>○ Centre de santé gouvernemental<br>○ Clinique PF<br>○ Stratégie / Clinique mobile (secteur public)<br>○ Autre secteur public<br>○ Hôpital/Clinique privée<br>○ Pharmacie<br>○ Médecin privé<br>○ Stratégie / Clinique mobile (secteur privé)<br>○ Agent de santé<br>○ Autre secteur médical privé<br>○ Boutique<br>○ Institution religieuse<br>○ Evènement communautaire<br>○ Ami(e)/Parent(e)<br>○ Agent de santé communautaire<br>○ Vendeur de la rue<br>○ Autre<br>○ Ne sait pas<br>○ Pas de réponse<br>`selected(${abt_meds_where}, filter_list) or (filter_list = 'always')`
712a.i. J'aimerais vous poser à présent quelques questions supplémentaires sur ${friend1_name}. A-t-elle déjà fait quelque chose pour faire passer une grossesse parce qu'elle était enceinte ou pensait qu'elle était enceinte ?<br>*Relancez pour confirmer si elle a réussi à faire passer sa grossesse. Si elle n'a pas réussi, sélectionnez « non ».*	○ Oui, j'en suis sûre<br>○ Oui, je crois<br>○ Non<br>○ Ne sait pas<br>○ Pas de réponse
713a.i. En quelle année cela s'est-il passé la dernière fois ?<br>*Si elle indique que cela s'est passé plus d'une fois, précisez que la question porte sur la dernière fois.*	`(${friend1_abt_yn} = 'yes') or (${friend1_abt_yn} = 'likely')`<br>Année:<br>_________________________
714a.i. Les femmes peuvent parfois faire plusieurs choses pour tenter de faire passer une grossesse. Est-ce que ${friend1_name} a fait plusieurs choses pour faire passer sa grossesse?	`(${friend1_abt_yn} = 'yes') or (${friend1_abt_yn} = 'likely')`<br>○ Oui, j'en suis sûre<br>○ Oui, je crois

	◯ Non<br>◯ Ne sait pas<br>◯ Pas de réponse
715a.i. Qu'a-t-elle fait en premier pour faire passer sa grossesse ?	`(${friend1_abt_mult_yn} = 'yes') or (${friend1_abt_mult_yn} = 'likely')`<br>◯ Intervention chirurgicale (curetage, AMIU, etc.)<br>◯ Comprimés appelées mifepristone ou misoprostol (Cytotec)<br>◯ Médicaments qu'on prend quand on a de la fièvre, comme des antibiotiques ou un médicament anti-palu<br>◯ Autres comprimés<br>◯ Méthodes traditionnelles (plantes, potions, décoctions, etc.)<br>◯ Produits industriels ingérés (eau de javel, coca-nescafé, etc.)<br>◯ Insertion de matériel dans le vagin (tige, boule d'herbes, kanigban, etc.)<br>◯ Autre<br>◯ Ne sait pas<br>◯ Pas de réponse
715a.i. Qu'a-t-elle fait pour faire passer sa grossesse?	`(${friend1_abt_mult_yn} = 'no') or (${friend1_abt_mult_yn} = '-88')`<br>◯ Intervention chirurgicale (curetage, AMIU, etc.)<br>◯ Comprimés appelées mifepristone ou misoprostol (Cytotec)<br>◯ Médicaments qu'on prend quand on a de la fièvre, comme des antibiotiques ou un médicament anti-palu<br>◯ Autres comprimés<br>◯ Méthodes traditionnelles (plantes, potions, décoctions, etc.)<br>◯ Produits industriels ingérés (eau de javel, coca-nescafé, etc.)<br>◯ Insertion de matériel dans le vagin (tige, boule d'herbes, kanigban, etc.)

	○ Autre<br>○ Ne sait pas<br>○ Pas de réponse
716a.i. Où est-elle allée pour cette intervention ?	(${friend1_abt_first} = 'surgery') or (${friend1_abt_only} = 'surgery')<br>○ Hôpital gouvernemental<br>○ Centre de santé gouvernemental<br>○ Clinique PF<br>○ Stratégie / Clinique mobile (secteur public)<br>○ Autre secteur public<br>○ Hôpital/Clinique privée<br>○ Pharmacie<br>○ Médecin privé<br>○ Stratégie / Clinique mobile (secteur privé)<br>○ Agent de santé<br>○ Autre secteur médical privé<br>○ Boutique<br>○ Institution religieuse<br>○ Evènement communautaire<br>○ Ami(e)/Parent(e)<br>○ Agent de santé communautaire<br>○ Vendeur de la rue<br>○ Autre<br>○ Ne sait pas<br>○ Pas de réponse
717a.i. Où a-t-elle obtenu les produits?	(${friend1_abt_first} = 'pills_abortion') or (${friend1_abt_only} = 'pills_abortion') or (${friend …<br>○ Hôpital gouvernemental<br>○ Centre de santé gouvernemental<br>○ Clinique PF<br>○ Stratégie / Clinique mobile (secteur public)<br>○ Autre secteur public<br>○ Hôpital/Clinique privée<br>○ Pharmacie<br>○ Médecin privé<br>○ Stratégie / Clinique mobile (secteur privé)<br>○ Agent de santé<br>○ Autre secteur médical

	privé<br>◯ Boutique<br>◯ Institution religieuse<br>◯ Evènement communautaire<br>◯ Ami(e)/Parent(e)<br>◯ Agent de santé communautaire<br>◯ Vendeur de la rue<br>◯ Autre<br>◯ Ne sait pas<br>◯ Pas de réponse
718a.i. Qu'a t-elle fait en dernier pour faire passer sa grossesse ?	`(${friend1_abt_mult_yn} = 'yes') or (${friend1_abt_mult_yn} = 'likely')`<br>◯ Intervention chirurgicale (curetage, AMIU, etc.)<br>◯ Comprimés appelées mifepristone ou misoprostol (Cytotec)<br>◯ Médicaments qu'on prend quand on a de la fièvre, comme des antibiotiques ou un médicament anti-palu<br>◯ Autres comprimés<br>◯ Méthodes traditionnelles (plantes, potions, décoctions, etc.)<br>◯ Produits industriels ingérés (eau de javel, coca-nescafé, etc.)<br>◯ Insertion de matériel dans le vagin (tige, boule d'herbes, kanigban, etc.)<br>◯ Autre<br>◯ Ne sait pas<br>◯ Pas de réponse
719a.i. Où est-elle allée pour cette intervention ?	`${friend1_abt_last} = 'surgery'`<br>◯ Hôpital gouvernemental<br>◯ Centre de santé gouvernemental<br>◯ Clinique PF<br>◯ Stratégie / Clinique mobile (secteur public)<br>◯ Autre secteur public<br>◯ Hôpital/Clinique privée<br>◯ Pharmacie<br>◯ Médecin privé<br>◯ Stratégie / Clinique mobile (secteur privé)

	◯ Agent de santé<br>◯ Autre secteur médical privé<br>◯ Boutique<br>◯ Institution religieuse<br>◯ Evènement communautaire<br>◯ Ami(e)/Parent(e)<br>◯ Agent de santé communautaire<br>◯ Vendeur de la rue<br>◯ Autre<br>◯ Ne sait pas<br>◯ Pas de réponse
720a.i. Où a-t-elle obtenu les produits ?	`(${friend1_abt_last} = 'pills_abortion') or (${friend1_abt_last} = 'pills_fever') or (${friend1_ab …`<br>◯ Hôpital gouvernemental<br>◯ Centre de santé gouvernemental<br>◯ Clinique PF<br>◯ Stratégie / Clinique mobile (secteur public)<br>◯ Autre secteur public<br>◯ Hôpital/Clinique privée<br>◯ Pharmacie<br>◯ Médecin privé<br>◯ Stratégie / Clinique mobile (secteur privé)<br>◯ Agent de santé<br>◯ Autre secteur médical privé<br>◯ Boutique<br>◯ Institution religieuse<br>◯ Evènement communautaire<br>◯ Ami(e)/Parent(e)<br>◯ Agent de santé communautaire<br>◯ Vendeur de la rue<br>◯ Autre<br>◯ Ne sait pas<br>◯ Pas de réponse
721a.i. Est-ce que ${friend1_name} a eu des problèmes lorsqu'elle a fait passer sa grossesse, la conduisant dans une structure de santé pour se faire soigner ?<br>*Si la répondante a déjà déclaré que son amie est allée dans une structure de santé lorsqu'elle a fait passer sa grossesse, nous cherchons à savoir si elle y est retournée à un autre moment en raison de*	`(${friend1_abt_yn} = 'yes') or (${friend1_abt_yn} = 'likely')`<br>◯ Oui, j'en suis sûre<br>◯ Oui, je crois<br>◯ Non<br>◯ Ne sait pas

complications qu'elle aurait pu rencontrer.	◯ Pas de réponse
712a.ii. Hormis cet événement, ${friend1_name} a-t-elle déjà fait quelque chose pour faire revenir ses règles parce qu'elle pensait qu'elle était enceinte ?<br>*Relancez pour confirmer si elle a réussi à faire revenir ses règles. Si elle n'a pas réussi, sélectionnez « non ».*	`(${friend1_abt_yn} = 'yes') or (${friend1_abt_yn} = 'likely')`<br>◯ Oui, j'en suis sûre<br>◯ Oui, je crois<br>◯ Non<br>◯ Ne sait pas<br>◯ Pas de réponse
712a.ii. ${friend1_name} a t-elle déjà fait quelque chose pour faire revenir ses règles parce qu'elle pensait qu'elle était enceinte?<br>*Relancez pour confirmer si elle a réussi à faire revenir ses règles. Si elle n'a pas réussi, sélectionnez « non ».*	`(${friend1_abt_yn} != 'yes') and (${friend1_abt_yn} != 'likely')`<br>◯ Oui, j'en suis sûre<br>◯ Oui, je crois<br>◯ Non<br>◯ Ne sait pas<br>◯ Pas de réponse
713a.ii En quelle année cela s'est-il passé la dernière fois?<br>*Si elle indique que cela s'est passé plus d'une fois, précisez que la question porte sur la dernière fois.*	`(${friend1_reg_yn} = 'yes') or (${friend1_reg_yn} = 'likely')`<br>Année:<br>__________________________
714a.ii Les femmes peuvent parfois faire plusieurs choses pour faire revenir leurs règles. ${friend1_name} a t-elle fait plus d'une chose pour faire revenir ses règles?	`((${friend1_reg_year} > ${friend1_abt_year}) or (${friend1_abt_year} = '')) and ((${friend1_reg_yn …`<br>◯ Oui, j'en suis sûre<br>◯ Oui, je crois<br>◯ Non<br>◯ Ne sait pas<br>◯ Pas de réponse
715a.ii Qu'a t-elle fait en premier pour faire revenir ses règles?	`((${friend1_reg_year} > ${friend1_abt_year}) or (${friend1_abt_year} = '')) and ((${friend1_reg_mu …`<br>◯ Intervention chirurgicale (curetage, AMIU, etc.)<br>◯ Comprimés appelées mifepristone ou misoprostol (Cytotec)<br>◯ Médicaments qu'on prend quand on a de la fièvre, comme des antibiotiques ou un médicament anti-palu<br>◯ Autres comprimés<br>◯ Méthodes traditionnelles (plantes, potions, décoctions, etc.)<br>◯ Produits industriels ingérés (eau de javel, coca-

	nescafé, etc.)<br>◯ Insertion de matériel dans le vagin (tige, boule d'herbes, kanigban, etc.)<br>◯ Autre<br>◯ Ne sait pas<br>◯ Pas de réponse
715a.ii Qu'a t-elle fait pour faire revenir ses règles ?	((${friend1_reg_year} > ${friend1_abt_year}) or (${friend1_abt_year} = '')) and ((${friend1_reg_mu …<br>◯ Intervention chirurgicale (curetage, AMIU, etc.)<br>◯ Comprimés appelées mifepristone ou misoprostol (Cytotec)<br>◯ Médicaments qu'on prend quand on a de la fièvre, comme des antibiotiques ou un médicament anti-palu<br>◯ Autres comprimés<br>◯ Méthodes traditionnelles (plantes, potions, décoctions, etc.)<br>◯ Produits industriels ingérés (eau de javel, coca-nescafé, etc.)<br>◯ Insertion de matériel dans le vagin (tige, boule d'herbes, kanigban, etc.)<br>◯ Autre<br>◯ Ne sait pas<br>◯ Pas de réponse
716a.ii Où est-elle allée pour cette intervention ?	((${friend1_reg_year} > ${friend1_abt_year}) or (${friend1_abt_year} = '')) and ((${friend1_reg_fi …<br>◯ Hôpital gouvernemental<br>◯ Centre de santé gouvernemental<br>◯ Clinique PF<br>◯ Stratégie / Clinique mobile (secteur public)<br>◯ Autre secteur public<br>◯ Hôpital/Clinique privée<br>◯ Pharmacie<br>◯ Médecin privé<br>◯ Stratégie / Clinique mobile (secteur privé)<br>◯ Agent de santé<br>◯ Autre secteur médical

	privé<br>○ Boutique<br>○ Institution religieuse<br>○ Evènement communautaire<br>○ Ami(e)/Parent(e)<br>○ Agent de santé communautaire<br>○ Vendeur de la rue<br>○ Autre<br>○ Ne sait pas<br>○ Pas de réponse
717a.ii Où a t-elle obtenu les produits ?	((${friend1_reg_year} > ${friend1_abt_year}) or (${friend1_abt_year} = '')) and ((${friend1_reg_fi …<br>○ Hôpital gouvernemental<br>○ Centre de santé gouvernemental<br>○ Clinique PF<br>○ Stratégie / Clinique mobile (secteur public)<br>○ Autre secteur public<br>○ Hôpital/Clinique privée<br>○ Pharmacie<br>○ Médecin privé<br>○ Stratégie / Clinique mobile (secteur privé)<br>○ Agent de santé<br>○ Autre secteur médical privé<br>○ Boutique<br>○ Institution religieuse<br>○ Evènement communautaire<br>○ Ami(e)/Parent(e)<br>○ Agent de santé communautaire<br>○ Vendeur de la rue<br>○ Autre<br>○ Ne sait pas<br>○ Pas de réponse
718a.ii Qu'a t-elle fait en dernier qui lui a finalement permis de faire revenir ses règles ?	((${friend1_reg_year} > ${friend1_abt_year}) or (${friend1_abt_year} = '')) and ((${friend1_reg_mu …<br>○ Intervention chirurgicale (curetage, AMIU, etc.)<br>○ Comprimés appelées mifepristone ou misoprostol (Cytotec)

	Médicaments qu'on prend quand on a de la fièvre, comme des antibiotiques ou un médicament anti-palu<br>Autres comprimés<br>Méthodes traditionnelles (plantes, potions, décoctions, etc.)<br>Produits industriels ingérés (eau de javel, coca-nescafé, etc.)<br>Insertion de matériel dans le vagin (tige, boule d'herbes, kanigban, etc.)<br>Autre<br>Ne sait pas<br>Pas de réponse
719a.ii Où est-elle allée pour cette intervention ?	((${friend1_reg_year} > ${friend1_abt_year}) or (${friend1_abt_year} = '')) and (${friend1_reg_las …<br>Hôpital gouvernemental<br>Centre de santé gouvernemental<br>Clinique PF<br>Stratégie / Clinique mobile (secteur public)<br>Autre secteur public<br>Hôpital/Clinique privée<br>Pharmacie<br>Médecin privé<br>Stratégie / Clinique mobile (secteur privé)<br>Agent de santé<br>Autre secteur médical privé<br>Boutique<br>Institution religieuse<br>Evènement communautaire<br>Ami(e)/Parent(e)<br>Agent de santé communautaire<br>Vendeur de la rue<br>Autre<br>Ne sait pas<br>Pas de réponse
720a.ii Où a t-elle obtenu les produits ?	((${friend1_reg_year} > ${friend1_abt_year}) or (${friend1_abt_year} = '')) and ((${friend1_reg_la …

	Hôpital gouvernemental<br>Centre de santé gouvernemental<br>Clinique PF<br>Stratégie / Clinique mobile (secteur public)<br>Autre secteur public<br>Hôpital/Clinique privée<br>Pharmacie<br>Médecin privé<br>Stratégie / Clinique mobile (secteur privé)<br>Agent de santé<br>Autre secteur médical privé<br>Boutique<br>Institution religieuse<br>Evènement communautaire<br>Ami(e)/Parent(e)<br>Agent de santé communautaire<br>Vendeur de la rue<br>Autre<br>Ne sait pas<br>Pas de réponse
721a.ii ${friend1_name} a eu des problèmes lorsqu'elle a fait revenir ses règles, la conduisant à aller dans une structure de santé pour se faire soigner ?<br>*Si la répondante a déjà déclaré que son amie est allée dans une structure de santé lorsqu'elle a fait revenir ses règles, nous cherchons à savoir si elle y est retournée à un autre moment en raison de complications qu'elle aurait pu rencontrer.*	`((${friend1_reg_year} > ${friend1_abt_year}) or (${friend1_abt_year} = '')) and ((${friend1_reg_yn …`<br>Oui, j'en suis sûre<br>Oui, je crois<br>Non<br>Ne sait pas<br>Pas de réponse
712b.i. J'aimerais vous poser à présent quelques questions supplémentaires sur ${friend2_name}. A-t-elle déjà fait quelque chose pour faire passer une grossesse parce qu'elle était enceinte ou pensait qu'elle était enceinte?<br>*Relancez pour confirmer si elle a réussi à faire passer sa grossesse. Si elle n'a pas réussi, sélectionnez « non ».*	Oui, j'en suis sûre<br>Oui, je crois<br>Non<br>Ne sait pas<br>Pas de réponse
713b.i. En quelle année cela s'est-il passé la dernière fois ?<br>*Si elle indique que cela s'est passé plus d'une fois, précisez que la question porte sur la dernière fois.*	`(${friend2_abt_yn} = 'yes') or (${friend2_abt_yn} = 'likely')`<br>Année:<br>_________________________
714b.i. Les femmes peuvent parfois faire plusieurs choses pour tenter de faire passer une grossesse. Est-ce que ${friend2_name} a fait plusieurs choses pour faire passer sa grossesse?	`(${friend2_abt_yn} = 'yes') or (${friend2_abt_yn} = 'likely')`<br>Oui, j'en suis sûre<br>Oui, je crois

	◯ Non<br>◯ Ne sait pas<br>◯ Pas de réponse
715b.i. Qu'a-t-elle fait en premier pour faire passer sa grossesse ?	(${friend2_abt_mult_yn} = 'yes') or (${friend2_abt_mult_yn} = 'likely')<br>◯ Intervention chirurgicale (curetage, AMIU, etc.)<br>◯ Comprimés appelées mifepristone ou misoprostol (Cytotec)<br>◯ Médicaments qu'on prend quand on a de la fièvre, comme des antibiotiques ou un médicament anti-palu<br>◯ Autres comprimés<br>◯ Méthodes traditionnelles (plantes, potions, décoctions, etc.)<br>◯ Produits industriels ingérés (eau de javel, coca-nescafé, etc.)<br>◯ Insertion de matériel dans le vagin (tige, boule d'herbes, kanigban, etc.)<br>◯ Autre<br>◯ Ne sait pas<br>◯ Pas de réponse
715b.i. Qu'a-t-elle fait pour faire passer sa grossesse?	(${friend2_abt_mult_yn} = 'no') or (${friend2_abt_mult_yn} = '-88')<br>◯ Intervention chirurgicale (curetage, AMIU, etc.)<br>◯ Comprimés appelées mifepristone ou misoprostol (Cytotec)<br>◯ Médicaments qu'on prend quand on a de la fièvre, comme des antibiotiques ou un médicament anti-palu<br>◯ Autres comprimés<br>◯ Méthodes traditionnelles (plantes, potions, décoctions, etc.)<br>◯ Produits industriels ingérés (eau de javel, coca-nescafé, etc.)<br>◯ Insertion de matériel dans le vagin (tige, boule d'herbes, kanigban, etc.)

	○ Autre<br>○ Ne sait pas<br>○ Pas de réponse
716b.i. Où est-elle allée pour cette intervention ?	(${friend2_abt_first} = 'surgery') or (${friend2_abt_only} = 'surgery')<br>○ Hôpital gouvernemental<br>○ Centre de santé gouvernemental<br>○ Clinique PF<br>○ Stratégie / Clinique mobile (secteur public)<br>○ Autre secteur public<br>○ Hôpital/Clinique privée<br>○ Pharmacie<br>○ Médecin privé<br>○ Stratégie / Clinique mobile (secteur privé)<br>○ Agent de santé<br>○ Autre secteur médical privé<br>○ Boutique<br>○ Institution religieuse<br>○ Evènement communautaire<br>○ Ami(e)/Parent(e)<br>○ Agent de santé communautaire<br>○ Vendeur de la rue<br>○ Autre<br>○ Ne sait pas<br>○ Pas de réponse
717b.i. Où a-t-elle obtenu les produits?	(${friend2_abt_first} = 'pills_abortion') or (${friend2_abt_only} = 'pills_abortion') or (${friend …<br>○ Hôpital gouvernemental<br>○ Centre de santé gouvernemental<br>○ Clinique PF<br>○ Stratégie / Clinique mobile (secteur public)<br>○ Autre secteur public<br>○ Hôpital/Clinique privée<br>○ Pharmacie<br>○ Médecin privé<br>○ Stratégie / Clinique mobile (secteur privé)<br>○ Agent de santé<br>○ Autre secteur médical

	privé<br>○ Boutique<br>○ Institution religieuse<br>○ Evènement communautaire<br>○ Ami(e)/Parent(e)<br>○ Agent de santé communautaire<br>○ Vendeur de la rue<br>○ Autre<br>○ Ne sait pas<br>○ Pas de réponse
718b.i. Qu'a t-elle fait en dernier pour faire passer sa grossesse ?	`(${friend2_abt_mult_yn} = 'yes') or (${friend2_abt_mult_yn} = 'likely')`<br>○ Intervention chirurgicale (curetage, AMIU, etc.)<br>○ Comprimés appelées mifepristone ou misoprostol (Cytotec)<br>○ Médicaments qu'on prend quand on a de la fièvre, comme des antibiotiques ou un médicament anti-palu<br>○ Autres comprimés<br>○ Méthodes traditionnelles (plantes, potions, décoctions, etc.)<br>○ Produits industriels ingérés (eau de javel, coca-nescafé, etc.)<br>○ Insertion de matériel dans le vagin (tige, boule d'herbes, kanigban, etc.)<br>○ Autre<br>○ Ne sait pas<br>○ Pas de réponse
719b.i. Où est-elle allée pour cette intervention ?	`${friend2_abt_last} = 'surgery'`<br>○ Hôpital gouvernemental<br>○ Centre de santé gouvernemental<br>○ Clinique PF<br>○ Stratégie / Clinique mobile (secteur public)<br>○ Autre secteur public<br>○ Hôpital/Clinique privée<br>○ Pharmacie<br>○ Médecin privé<br>○ Stratégie / Clinique mobile (secteur privé)

	○ Agent de santé<br>○ Autre secteur médical privé<br>○ Boutique<br>○ Institution religieuse<br>○ Evènement communautaire<br>○ Ami(e)/Parent(e)<br>○ Agent de santé communautaire<br>○ Vendeur de la rue<br>○ Autre<br>○ Ne sait pas<br>○ Pas de réponse
720b.i. Où a-t-elle obtenu les produits ?	`(${friend2_abt_last} = 'pills_abortion') or (${friend2_abt_last} = 'pills_fever') or (${friend2_ab …`<br>○ Hôpital gouvernemental<br>○ Centre de santé gouvernemental<br>○ Clinique PF<br>○ Stratégie / Clinique mobile (secteur public)<br>○ Autre secteur public<br>○ Hôpital/Clinique privée<br>○ Pharmacie<br>○ Médecin privé<br>○ Stratégie / Clinique mobile (secteur privé)<br>○ Agent de santé<br>○ Autre secteur médical privé<br>○ Boutique<br>○ Institution religieuse<br>○ Evènement communautaire<br>○ Ami(e)/Parent(e)<br>○ Agent de santé communautaire<br>○ Vendeur de la rue<br>○ Autre<br>○ Ne sait pas<br>○ Pas de réponse
721b.i. Est-ce que ${friend2_name} a eu des problèmes lorsqu'elle a fait passer sa grossesse, la conduisant dans une structure de santé pour se faire soigner ?<br>*Si la répondante a déjà déclaré que son amie est allée dans une structure de santé lorsqu'elle a fait passer sa grossesse, nous cherchons à savoir si elle y est retournée à un autre moment en raison de*	`(${friend2_abt_yn} = 'yes') or (${friend2_abt_yn} = 'likely')`<br>○ Oui, j'en suis sûre<br>○ Oui, je crois<br>○ Non

complications qu'elle aurait pu rencontrer.	Ne sait pas<br>Pas de réponse
712b.ii. Hormis cet événement, ${friend2_name} a-t-elle déjà fait quelque chose pour faire revenir ses règles parce qu'elle pensait qu'elle était enceinte ?<br>*Relancez pour confirmer si elle a réussi à faire revenir ses règles. Si elle n'a pas réussi, sélectionnez « non ».*	`(${friend2_abt_yn} = 'yes') or (${friend2_abt_yn} = 'likely')`<br>Oui, j'en suis sûre<br>Oui, je crois<br>Non<br>Ne sait pas<br>Pas de réponse
712b.ii. ${friend2_name} a t-elle déjà fait quelque chose pour faire revenir ses règles parce qu'elle pensait qu'elle était enceinte?<br>*Relancez pour confirmer si elle a réussi à faire revenir ses règles. Si elle n'a pas réussi, sélectionnez « non ».*	`(${friend2_abt_yn} != 'yes') and (${friend2_abt_yn} != 'likely')`<br>Oui, j'en suis sûre<br>Oui, je crois<br>Non<br>Ne sait pas<br>Pas de réponse
713b.ii En quelle année cela s'est-il passé la dernière fois?<br>*Si elle indique que cela s'est passé plus d'une fois, précisez que la question porte sur la dernière fois.*	`(${friend2_reg_yn} = 'yes') or (${friend2_reg_yn} = 'likely')`<br>Année:<br>_________________________
714b.ii Les femmes peuvent parfois faire plusieurs choses pour faire revenir leurs règles. ${friend2_name} a t-elle fait plus d'une chose pour faire revenir ses règles?	`((${friend2_reg_year} > ${friend2_abt_year}) or (${friend2_abt_year} = '')) and ((${friend2_reg_yn …`<br>Oui, j'en suis sûre<br>Oui, je crois<br>Non<br>Ne sait pas<br>Pas de réponse
715b.ii Qu'a t-elle fait en premier pour faire revenir ses règles?	`((${friend2_reg_year} > ${friend2_abt_year}) or (${friend2_abt_year} = '')) and ((${friend2_reg_mu …`<br>Intervention chirurgicale (curetage, AMIU, etc.)<br>Comprimés appelées mifepristone ou misoprostol (Cytotec)<br>Médicaments qu'on prend quand on a de la fièvre, comme des antibiotiques ou un médicament anti-palu<br>Autres comprimés<br>Méthodes traditionnelles (plantes, potions, décoctions, etc.)<br>Produits industriels ingérés (eau de javel, coca-

	nescafé, etc.)<br>◯ Insertion de matériel dans le vagin (tige, boule d'herbes, kanigban, etc.)<br>◯ Autre<br>◯ Ne sait pas<br>◯ Pas de réponse
715b.ii Qu'a t-elle fait pour faire revenir ses règles ?	`((${friend2_reg_year} > ${friend2_abt_year}) or (${friend2_abt_year} = '')) and ((${friend2_reg_mu …`<br>◯ Intervention chirurgicale (curetage, AMIU, etc.)<br>◯ Comprimés appelées mifepristone ou misoprostol (Cytotec)<br>◯ Médicaments qu'on prend quand on a de la fièvre, comme des antibiotiques ou un médicament anti-palu<br>◯ Autres comprimés<br>◯ Méthodes traditionnelles (plantes, potions, décoctions, etc.)<br>◯ Produits industriels ingérés (eau de javel, coca-nescafé, etc.)<br>◯ Insertion de matériel dans le vagin (tige, boule d'herbes, kanigban, etc.)<br>◯ Autre<br>◯ Ne sait pas<br>◯ Pas de réponse
716b.ii Où est-elle allée pour cette intervention ?	`((${friend2_reg_year} > ${friend2_abt_year}) or (${friend2_abt_year} = '')) and ((${friend2_reg_fi …`<br>◯ Hôpital gouvernemental<br>◯ Centre de santé gouvernemental<br>◯ Clinique PF<br>◯ Stratégie / Clinique mobile (secteur public)<br>◯ Autre secteur public<br>◯ Hôpital/Clinique privée<br>◯ Pharmacie<br>◯ Médecin privé<br>◯ Stratégie / Clinique mobile (secteur privé)<br>◯ Agent de santé<br>◯ Autre secteur médical

	privé<br>Boutique<br>Institution religieuse<br>Evènement communautaire<br>Ami(e)/Parent(e)<br>Agent de santé communautaire<br>Vendeur de la rue<br>Autre<br>Ne sait pas<br>Pas de réponse
717b.ii Où a t-elle obtenu les produits ?	`((${friend2_reg_year} > ${friend2_abt_year}) or (${friend2_abt_year} = '')) and ((${friend2_reg_fi …`<br>Hôpital gouvernemental<br>Centre de santé gouvernemental<br>Clinique PF<br>Stratégie / Clinique mobile (secteur public)<br>Autre secteur public<br>Hôpital/Clinique privée<br>Pharmacie<br>Médecin privé<br>Stratégie / Clinique mobile (secteur privé)<br>Agent de santé<br>Autre secteur médical privé<br>Boutique<br>Institution religieuse<br>Evènement communautaire<br>Ami(e)/Parent(e)<br>Agent de santé communautaire<br>Vendeur de la rue<br>Autre<br>Ne sait pas<br>Pas de réponse
718b.ii Qu'a t-elle fait en dernier qui lui a finalement permis de faire revenir ses règles ?	`((${friend2_reg_year} > ${friend2_abt_year}) or (${friend2_abt_year} = '')) and ((${friend2_reg_mu …`<br>Intervention chirurgicale (curetage, AMIU, etc.)<br>Comprimés appelées mifepristone ou misoprostol (Cytotec)

	◯ Médicaments qu'on prend quand on a de la fièvre, comme des antibiotiques ou un médicament anti-palu<br>◯ Autres comprimés<br>◯ Méthodes traditionnelles (plantes, potions, décoctions, etc.)<br>◯ Produits industriels ingérés (eau de javel, coca-nescafé, etc.)<br>◯ Insertion de matériel dans le vagin (tige, boule d'herbes, kanigban, etc.)<br>◯ Autre<br>◯ Ne sait pas<br>◯ Pas de réponse
719b.ii Où est-elle allée pour cette intervention ?	`((${friend2_reg_year} > ${friend2_abt_year}) or (${friend2_abt_year} = '')) and (${friend2_reg_las …`<br>◯ Hôpital gouvernemental<br>◯ Centre de santé gouvernemental<br>◯ Clinique PF<br>◯ Stratégie / Clinique mobile (secteur public)<br>◯ Autre secteur public<br>◯ Hôpital/Clinique privée<br>◯ Pharmacie<br>◯ Médecin privé<br>◯ Stratégie / Clinique mobile (secteur privé)<br>◯ Agent de santé<br>◯ Autre secteur médical privé<br>◯ Boutique<br>◯ Institution religieuse<br>◯ Evènement communautaire<br>◯ Ami(e)/Parent(e)<br>◯ Agent de santé communautaire<br>◯ Vendeur de la rue<br>◯ Autre<br>◯ Ne sait pas<br>◯ Pas de réponse
720b.ii Où a t-elle obtenu les produits ?	`((${friend2_reg_year} > ${friend2_abt_year}) or (${friend2_abt_year} = '')) and`

	((${friend2_reg_la …<br>Hôpital gouvernemental<br>Centre de santé gouvernemental<br>Clinique PF<br>Stratégie / Clinique mobile (secteur public)<br>Autre secteur public<br>Hôpital/Clinique privée<br>Pharmacie<br>Médecin privé<br>Stratégie / Clinique mobile (secteur privé)<br>Agent de santé<br>Autre secteur médical privé<br>Boutique<br>Institution religieuse<br>Evènement communautaire<br>Ami(e)/Parent(e)<br>Agent de santé communautaire<br>Vendeur de la rue<br>Autre<br>Ne sait pas<br>Pas de réponse
721b.ii ${friend2_name} a eu des problèmes lorsqu'elle a fait revenir ses règles, la conduisant à aller dans une structure de santé pour se faire soigner ?<br>*Si la répondante a déjà déclaré que son amie est allée dans une structure de santé lorsqu'elle a fait revenir ses règles, nous cherchons à savoir si elle y est retournée à un autre moment en raison de complications qu'elle aurait pu rencontrer.*	((${friend2_reg_year} > ${friend2_abt_year}) or (${friend2_abt_year} = '')) and ((${friend2_reg_yn …<br>Oui, j'en suis sûre<br>Oui, je crois<br>Non<br>Ne sait pas<br>Pas de réponse
722a. J'aimerais maintenant vous poser des questions sur votre propre expérience. Avez-vous déjà fait quelque chose pour faire passer une grossesse parce que vous étiez enceinte ou vous pensiez que vous étiez enceinte?<br>*Relancez pour confirmer si elle a réussi à faire passer la grossesse. Si elle n'a pas réussi, sélectionnez « non ».*	Oui<br>Non<br>Pas de réponse
723a. En quelle année cela s'est-il passé pour la dernière fois ?<br>*Si elle indique que cela s'est passé plusieurs fois, préciser que la question porte sur la fois la plus récente.*	(${self_abt_yn} = 'yes')<br>Année:<br>_________________________
724a. Avez-vous fait plusieurs choses pour tenter de faire passer une grossesse?	(${self_abt_yn} = 'yes')<br>Oui<br>Non<br>Pas de réponse
	(${self_abt_mult_yn} =

725a. Qu’avez-vous fait en premier ?	'yes')<br>◯ Intervention chirurgicale (curetage, AMIU, etc.)<br>◯ Comprimés appelées mifepristone ou misoprostol (Cytotec)<br>◯ Médicaments qu’on prend quand on a de la fièvre, comme des antibiotiques ou un médicament anti-palu<br>◯ Autres comprimés<br>◯ Méthodes traditionnelles (plantes, potions, décoctions, etc.)<br>◯ Produits industriels ingérés (eau de javel, coca-nescafé, etc.)<br>◯ Insertion de matériel dans le vagin (tige, boule d’herbes, kanigban, etc.)<br>◯ Autre<br>◯ Ne sait pas<br>◯ Pas de réponse
725a. Qu’avez-vous fait ?	(${self_abt_mult_yn} = 'no')<br>◯ Intervention chirurgicale (curetage, AMIU, etc.)<br>◯ Comprimés appelées mifepristone ou misoprostol (Cytotec)<br>◯ Médicaments qu’on prend quand on a de la fièvre, comme des antibiotiques ou un médicament anti-palu<br>◯ Autres comprimés<br>◯ Méthodes traditionnelles (plantes, potions, décoctions, etc.)<br>◯ Produits industriels ingérés (eau de javel, coca-nescafé, etc.)<br>◯ Insertion de matériel dans le vagin (tige, boule d’herbes, kanigban, etc.)<br>◯ Autre<br>◯ Ne sait pas<br>◯ Pas de réponse
726a. Où êtes-vous allée pour cette intervention ?	(${self_abt_first} = 'surgery') or (${self_abt_only} = 'surgery')

	◯ Hôpital gouvernemental<br>◯ Centre de santé gouvernemental<br>◯ Clinique PF<br>◯ Stratégie / Clinique mobile (secteur public)<br>◯ Autre secteur public<br>◯ Hôpital/Clinique privée<br>◯ Pharmacie<br>◯ Médecin privé<br>◯ Stratégie / Clinique mobile (secteur privé)<br>◯ Agent de santé<br>◯ Autre secteur médical privé<br>◯ Boutique<br>◯ Institution religieuse<br>◯ Evènement communautaire<br>◯ Ami(e)/Parent(e)<br>◯ Agent de santé communautaire<br>◯ Vendeur de la rue<br>◯ Autre<br>◯ Ne sait pas<br>◯ Pas de réponse
727a. Où avez-vous obtenu les produits ?	`(${self_abt_first} = 'pills_abortion') or (${self_abt_only} = 'pills_abortion') or (${self_abt_fir …`<br>◯ Hôpital gouvernemental<br>◯ Centre de santé gouvernemental<br>◯ Clinique PF<br>◯ Stratégie / Clinique mobile (secteur public)<br>◯ Autre secteur public<br>◯ Hôpital/Clinique privée<br>◯ Pharmacie<br>◯ Médecin privé<br>◯ Stratégie / Clinique mobile (secteur privé)<br>◯ Agent de santé<br>◯ Autre secteur médical privé<br>◯ Boutique<br>◯ Institution religieuse<br>◯ Evènement communautaire<br>◯ Ami(e)/Parent(e)<br>◯ Agent de santé

	communautaire<br>Vendeur de la rue<br>Autre<br>Ne sait pas<br>Pas de réponse
728a. Qu'avez-vous fait en dernier pour faire passer la grossesse ?	(${self_abt_mult_yn} = 'yes')<br>Intervention chirurgicale (curetage, AMIU, etc.)<br>Comprimés appelées mifepristone ou misoprostol (Cytotec)<br>Médicaments qu'on prend quand on a de la fièvre, comme des antibiotiques ou un médicament anti-palu<br>Autres comprimés<br>Méthodes traditionnelles (plantes, potions, décoctions, etc.)<br>Produits industriels ingérés (eau de javel, coca-nescafé, etc.)<br>Insertion de matériel dans le vagin (tige, boule d'herbes, kanigban, etc.)<br>Autre<br>Ne sait pas<br>Pas de réponse
729a. Où êtes-vous allée pour cette intervention ?	${self_abt_last} = 'surgery'<br>Hôpital gouvernemental<br>Centre de santé gouvernemental<br>Clinique PF<br>Stratégie / Clinique mobile (secteur public)<br>Autre secteur public<br>Hôpital/Clinique privée<br>Pharmacie<br>Médecin privé<br>Stratégie / Clinique mobile (secteur privé)<br>Agent de santé<br>Autre secteur médical privé<br>Boutique<br>Institution religieuse<br>Evènement communautaire

	○ Ami(e)/Parent(e)<br>○ Agent de santé communautaire<br>○ Vendeur de la rue<br>○ Autre<br>○ Ne sait pas<br>○ Pas de réponse
730a. Où avez-vous obtenu les produits ?	(${self_abt_last} = 'pills_abortion') or (${self_abt_last} = 'pills_fever') or (${self_abt_last} = …<br>○ Hôpital gouvernemental<br>○ Centre de santé gouvernemental<br>○ Clinique PF<br>○ Stratégie / Clinique mobile (secteur public)<br>○ Autre secteur public<br>○ Hôpital/Clinique privée<br>○ Pharmacie<br>○ Médecin privé<br>○ Stratégie / Clinique mobile (secteur privé)<br>○ Agent de santé<br>○ Autre secteur médical privé<br>○ Boutique<br>○ Institution religieuse<br>○ Evènement communautaire<br>○ Ami(e)/Parent(e)<br>○ Agent de santé communautaire<br>○ Vendeur de la rue<br>○ Autre<br>○ Ne sait pas<br>○ Pas de réponse
731a. Avez-vous eu des problèmes lorsque vous avez fait passer la grossesse, vous conduisant à aller dans une structure de santé pour vous faire soigner ?<br>*Si la répondante a déjà déclaré être allée dans une structure de santé lorsqu’elle a fait passer sa grossesse, nous voulons savoir si elle y est retournée à un autre moment en raison de complications qu’elle aurait pu rencontrer.*	(${self_abt_yn} = 'yes')<br>○ Oui<br>○ Non<br>○ Ne sait pas<br>○ Pas de réponse
732a. Avez-vous parlé de cette expérience à l’une des personnes suivantes ?<br>*Lisez les choix de réponse à voix haute. Sélectionnez toutes les réponses qui conviennent.*	(${self_abt_yn} = 'yes')<br>☐ Epoux/partenaire masculin<br>☐ Sœur<br>☐ Frère<br>☐ Mère<br>☐ Père

	☐ Autre membre de la famille<br>☐ Amie 1: ${friend1_name}<br>☐ Amie 2: ${friend2_name}<br>☐ Autre amie<br>☐ Autre personne<br>☐ Aucune personne<br>☐ Ne sait pas<br>☐ Pas de réponse<br>`(${friend1_name} != '' and ${friend1_name} != '-99' and filter_list = 'friend1') or (${friend2_name} != '' and ${friend2_name} != '-99' and filter_list = 'friend2') or (filter_list = 'always')`
722b. Hormis cet événement, avez-vous déjà fait quelque chose pour faire revenir vos règles parce que vous pensiez que vous étiez enceinte ?<br>*Relancez pour confirmer si elle a réussi à faire revenir les règles. Si elle n'a pas réussi, sélectionnez « non ».*	`${self_abt_yn} = 'yes'`<br>○ Oui<br>○ Non<br>○ Pas de réponse
722b. Avez-vous déjà fait quelque chose pour faire revenir vos règles parce que vous pensiez que vous étiez enceinte ?<br>*Relancez pour confirmer si elle a réussi à faire revenir les règles. Si elle n'a pas réussi, sélectionnez « non ».*	`${self_abt_yn} != 'yes'`<br>○ Oui<br>○ Non<br>○ Pas de réponse
723b.ii En quelle année cela s'est-il passé la dernière fois?<br>*Si elle indique que cela s'est passé plus d'une fois, précisez que la question porte sur la dernière fois.*	`(${self_reg_yn} = 'yes')`<br>Année:<br>..............................
724b. Avez-vous fait plus d'une chose pour faire revenir vos règles?	`((${self_reg_year} > ${self_abt_year}) or (${self_abt_year} = '')) and ((${self_reg_yn} = 'yes'))`<br>○ Oui<br>○ Non<br>○ Pas de réponse
725b. Qu'avez-vous fait en premier ?	`((${self_reg_year} > ${self_abt_year}) or (${self_abt_year} = '')) and ((${self_reg_mult_yn} = 'ye …`<br>○ Intervention chirurgicale (curetage, AMIU, etc.)<br>○ Comprimés appelées mifepristone ou misoprostol (Cytotec)<br>○ Médicaments qu'on prend quand on a de la fièvre, comme des antibiotiques ou un médicament anti-palu<br>○ Autres comprimés<br>○ Méthodes traditionnelles (plantes, potions, décoctions,

	etc.)<br>○ Produits industriels ingérés (eau de javel, coca-nescafé, etc.)<br>○ Insertion de matériel dans le vagin (tige, boule d'herbes, kanigban, etc.)<br>○ Autre<br>○ Ne sait pas<br>○ Pas de réponse
725b. Qu'avez-vous fait ?	((${self_reg_year} > ${self_abt_year}) or (${self_abt_year} = '')) and ((${self_reg_mult_yn} = 'no …<br>○ Intervention chirurgicale (curetage, AMIU, etc.)<br>○ Comprimés appelées mifepristone ou misoprostol (Cytotec)<br>○ Médicaments qu'on prend quand on a de la fièvre, comme des antibiotiques ou un médicament anti-palu<br>○ Autres comprimés<br>○ Méthodes traditionnelles (plantes, potions, décoctions, etc.)<br>○ Produits industriels ingérés (eau de javel, coca-nescafé, etc.)<br>○ Insertion de matériel dans le vagin (tige, boule d'herbes, kanigban, etc.)<br>○ Autre<br>○ Ne sait pas<br>○ Pas de réponse
726b. Où êtes-vous allée pour cette intervention ?	((${self_reg_year} > ${self_abt_year}) or (${self_abt_year} = '')) and ((${self_reg_first} = 'surg …<br>○ Hôpital gouvernemental<br>○ Centre de santé gouvernemental<br>○ Clinique PF<br>○ Stratégie / Clinique mobile (secteur public)<br>○ Autre secteur public<br>○ Hôpital/Clinique privée<br>○ Pharmacie<br>○ Médecin privé<br>○ Stratégie / Clinique mobile (secteur privé)

	Agent de santé<br>Autre secteur médical privé<br>Boutique<br>Institution religieuse<br>Evènement communautaire<br>Ami(e)/Parent(e)<br>Agent de santé communautaire<br>Vendeur de la rue<br>Autre<br>Ne sait pas<br>Pas de réponse
727b. Où avez-vous obtenu les produits ?	`((${self_reg_year} > ${self_abt_year}) or (${self_abt_year} = '')) and ((${self_reg_first} = 'pill …`<br>Hôpital gouvernemental<br>Centre de santé gouvernemental<br>Clinique PF<br>Stratégie / Clinique mobile (secteur public)<br>Autre secteur public<br>Hôpital/Clinique privée<br>Pharmacie<br>Médecin privé<br>Stratégie / Clinique mobile (secteur privé)<br>Agent de santé<br>Autre secteur médical privé<br>Boutique<br>Institution religieuse<br>Evènement communautaire<br>Ami(e)/Parent(e)<br>Agent de santé communautaire<br>Vendeur de la rue<br>Autre<br>Ne sait pas<br>Pas de réponse
728b. Qu’avez-vous fait en dernier qui vous a finalement permis de faire revenir vos règles ?	`((${self_reg_year} > ${self_abt_year}) or (${self_abt_year} = '')) and ((${self_reg_mult_yn} = 'ye …`<br>Intervention chirurgicale (curetage, AMIU, etc.)<br>Comprimés appelées

	mifepristone ou misoprostol (Cytotec)<br>◯ Médicaments qu'on prend quand on a de la fièvre, comme des antibiotiques ou un médicament anti-palu<br>◯ Autres comprimés<br>◯ Méthodes traditionnelles (plantes, potions, décoctions, etc.)<br>◯ Produits industriels ingérés (eau de javel, coca-nescafé, etc.)<br>◯ Insertion de matériel dans le vagin (tige, boule d'herbes, kanigban, etc.)<br>◯ Autre<br>◯ Ne sait pas<br>◯ Pas de réponse
729b. Où êtes-vous allée pour cette intervention ?	`((${self_reg_year} > ${self_abt_year}) or (${self_abt_year} = '')) and (${self_reg_last} = 'surger …`<br>◯ Hôpital gouvernemental<br>◯ Centre de santé gouvernemental<br>◯ Clinique PF<br>◯ Stratégie / Clinique mobile (secteur public)<br>◯ Autre secteur public<br>◯ Hôpital/Clinique privée<br>◯ Pharmacie<br>◯ Médecin privé<br>◯ Stratégie / Clinique mobile (secteur privé)<br>◯ Agent de santé<br>◯ Autre secteur médical privé<br>◯ Boutique<br>◯ Institution religieuse<br>◯ Evènement communautaire<br>◯ Ami(e)/Parent(e)<br>◯ Agent de santé communautaire<br>◯ Vendeur de la rue<br>◯ Autre<br>◯ Ne sait pas<br>◯ Pas de réponse
730b. Où avez-vous obtenu les produits ?	`((${self_reg_year} > ${self_abt_year}) or (${self_abt_year} = ''))`

	`and ((${self_reg_last} = 'pills …`<br>◯ Hôpital gouvernemental<br>◯ Centre de santé gouvernemental<br>◯ Clinique PF<br>◯ Stratégie / Clinique mobile (secteur public)<br>◯ Autre secteur public<br>◯ Hôpital/Clinique privée<br>◯ Pharmacie<br>◯ Médecin privé<br>◯ Stratégie / Clinique mobile (secteur privé)<br>◯ Agent de santé<br>◯ Autre secteur médical privé<br>◯ Boutique<br>◯ Institution religieuse<br>◯ Evènement communautaire<br>◯ Ami(e)/Parent(e)<br>◯ Agent de santé communautaire<br>◯ Vendeur de la rue<br>◯ Autre<br>◯ Ne sait pas<br>◯ Pas de réponse
731b. Avez-vous eu des problèmes lorsque vous avez fait revenir ses règles, vous conduisant à aller dans une structure de santé pour vous faire soigner ?<br>*Si la répondante a déjà déclaré être allée dans une structure de santé lorsqu'elle a fait revenir ses règles, nous voulons savoir si elle y est retournée à un autre moment en raison de complications qu'elle aurait pu rencontrer.*	`((${self_reg_year} > ${self_abt_year}) or (${self_abt_year} = '')) and ((${self_reg_yn} = 'yes'))`<br>◯ Oui<br>◯ Non<br>◯ Ne sait pas<br>◯ Pas de réponse
732b. Avez-vous parlé de cette expérience à l'une des personnes suivantes ?<br>*Lisez les choix de réponse à voix haute. Sélectionnez toutes les réponses qui conviennent.*	`((${self_reg_year} > ${self_abt_year}) or (${self_abt_year} = '')) and ((${self_reg_yn} = 'yes'))`<br>☐ Epoux/partenaire masculin<br>☐ Sœur<br>☐ Frère<br>☐ Mère<br>☐ Père<br>☐ Autre membre de la famille<br>☐ Amie 1: ${friend1_name}<br>☐ Amie 2: ${friend2_name}<br>☐ Autre amie

	☐ Autre personne<br>☐ Aucune personne<br>☐ Ne sait pas<br>☐ Pas de réponse<br>(${friend1_name} != '' and ${friend1_name} != '-99' and filter_list = 'friend1') or (${friend2_name} != '' and ${friend2_name} != '-99' and filter_list = 'friend2') or (filter_list = 'always')
PNSME_701. Quelles étaient les raisons qui vous ont motivé de faire passer la grossesse la dernière fois?	(${self_abt_yn} = 'yes')<br>☐ Refus du conjoint (refus d’accepter l’enfant)<br>☐ Pas de moyen (pour s’occuper de l’enfant)<br>☐ Viol<br>☐ Inceste<br>☐ Mère a des problèmes de santé<br>☐ Problèmes de santé fœtale possibles<br>☐ Pas prêts à assumer la responsabilité<br>☐ Trop immature ou trop jeune pour avoir un enfant<br>☐ Veut poursuivre sa scolarité<br>☐ Parents de la femme veulent qu'elle avorte<br>☐ Problèmes relationnels ou veut éviter la monoparentalité.<br>☐ A tous les enfants qu'elle voulait<br>☐ Inquiète sure comment le fait d'avoir un bébé changerait sa vie.<br>☐ Ne veut pas que les autres sachent qu'elle a eu des relations sexuels ou qu'elle est enceinte<br>☐ Autre<br>☐ Aucun des éléments ci-dessus<br>☐ Pas de réponse
PNSME_702a. En dehors de cet évènement, avez-vous fait passer d'autres grossesses ?	(${self_abt_yn} = 'yes')<br>○ Oui<br>○ Non<br>○ Ne sait pas<br>○ Pas de réponse
PNSME_702b. Combien de fois ?	${abt_other_yn} = 'yes'

~~*Saisir -88 pour Ne sait pas, -99 pour Pas de réponse.*~~

PNSME_703. En quelle année cela s'est-il passé pour la première fois ?<br>*Saisissez 2020 pour Pas de réponse.*	Année:

	${consent_obtained}
Nous aimerions à présent vous poser des questions générales sur le fait de faire passer une grossesse. Pouvez-vous me dire si vous êtes tout à fait d'accord, d'accord, ni en accord, ni en désaccord, pas d'accord, ou pas du tout d'accord avec les propositions suivantes ?<br>*Cochez la case pour confirmer que vous avez défiler la liste jusqu'en bas.*	
Appuyez 'OK' pour continuer	○ OK

733. Il est acceptable pour une femme de faire passer une grossesse si la grossesse pose un risque de santé.	${consent_obtained}<br>○ Tout à fait d'accord<br>○ D'accord<br>○ Ni en accord, ni en désaccord<br>○ Pas d'accord<br>○ Pas du tout d'accord<br>○ Pas de réponse
734. Il est acceptable pour une femme de faire passer une grossesse si cette grossesse est le résultat d'un viol.	${consent_obtained}<br>○ Tout à fait d'accord<br>○ D'accord<br>○ Ni en accord, ni en désaccord<br>○ Pas d'accord<br>○ Pas du tout d'accord<br>○ Pas de réponse
PNSME_734. Il est acceptable pour une femme de faire passer une grossesse si cette grossesse est le résultat d'un rapport sexuel incestueux.	${consent_obtained}<br>○ Tout à fait d'accord<br>○ D'accord<br>○ Ni en accord, ni en désaccord<br>○ Pas d'accord<br>○ Pas du tout d'accord<br>○ Pas de réponse
735. Il est acceptable pour une femme de faire passer une grossesse si elle n'a pas les moyens d'élever un autre enfant.	${consent_obtained}<br>○ Tout à fait d'accord<br>○ D'accord<br>○ Ni en accord, ni en désaccord<br>○ Pas d'accord<br>○ Pas du tout d'accord<br>○ Pas de réponse
736. Il est acceptable pour une femme de faire passer une grossesse si elle ne veut pas avoir d'autres enfants.	${consent_obtained}<br>○ Tout à fait d'accord<br>○ D'accord<br>○ Ni en accord, ni en désaccord

	◯ Pas d'accord<br>◯ Pas du tout d'accord<br>◯ Pas de réponse
737. Une femme qui fait passer une grossesse met la honte sur sa famille.	${consent_obtained}<br>◯ Tout à fait d'accord<br>◯ D'accord<br>◯ Ni en accord, ni en désaccord<br>◯ Pas d'accord<br>◯ Pas du tout d'accord<br>◯ Pas de réponse
738. Une femme qui fait passer la grossesse ne devrait le dire à personne.	${consent_obtained}<br>◯ Tout à fait d'accord<br>◯ D'accord<br>◯ Ni en accord, ni en désaccord<br>◯ Pas d'accord<br>◯ Pas du tout d'accord<br>◯ Pas de réponse
PNSME_704a. Savez-vous s'il y a une loi sur l'avortement en Côte d'Ivoire ?	${consent_obtained}<br>◯ Oui<br>◯ Non<br>◯ Ne sait pas<br>◯ Pas de réponse
PNSME_704b. Existe-t-il des circonstances dans lesquelles l'avortement est légal en Côte d'Ivoire ?	${abt_law} = 'yes'<br>◯ Oui<br>◯ Non<br>◯ Ne sait pas<br>◯ Pas de réponse
FLW_801. Je vous remercie pour le temps que vous avez bien voulu nous accorder.<br>Cela nous intéresserait beaucoup d'en savoir plus sur ce que vous avez vécu pour faire passer votre grossesse ou faire revenir vos règles, y compris votre prise de décision sur la méthode à utiliser et sur où obtenir des services, entre autres. Seriez-vous d'accord pour vous réunir avec une autre femme du projet à une date ultérieure pour parler de votre expérience ?	(${self_abt_yn} = 'yes') or (${self_reg_yn} = 'yes')<br>◯ Oui<br>◯ Non<br>◯ Pas de réponse
FLW_802. Avez-vous un téléphone ?	${flw_willing} = 'yes'<br>◯ Oui<br>◯ Non<br>◯ Pas de réponse
FLW_803. Puis-je avoir votre numéro de téléphone principal au cas où nous souhaiterions vous recontacter ?<br>*Saisissez un numéro de 8 chiffres sans le code du pays. Veuillez ne pas inclure des espaces ni des tirets. Saisir -99 pour Pas de réponse.*	${flw_number_yn} = 'yes'<br>--------------------------------
FLW_804. Pouvez-vous répéter le numéro encore une fois ?<br>*Entrez un numéro à 8 chiffres sans l'indicatif du pays.*	(${flw_number_typed} != '') and (${flw_number_typed} != '0')

N'incluez pas d'espaces ou de tirets. Entrez 0 pour Pas de réponse.	
Remerciez la personne interrogée pour le temps qu'elle a bien voulu vous accorder.<br>*L'enquêtée a terminé, mais il vous reste d'autres questions à compléter en dehors de la maison*	${available} = 'yes'

Géoréférencement et résultats du Questionnaire

095. Géoréférencement<br>*Veuillez prendre des coordonnées GPS près de l'entrée de la maison. Saisir les coordonnées quand leur exactitude est inférieure à 6m.*	Toujours
096. Combien de fois avez-vous rendu visite à ce ménage pour interroger cette femme?	Toujours<br>○ 1ère fois<br>○ 2ème fois<br>○ 3ème fois
097. En quelle langue avez-vous effectué cette enquête ?	009a = 1<br>○ Anglais<br>○ Français<br>○ Arabe<br>○ Baoulé<br>○ Sénoufo<br>○ Yacouba<br>○ Agni<br>○ Attié<br>○ Guéré<br>○ Bété<br>○ Dioula<br>○ Abbey<br>○ Mahou<br>○ Wobé<br>○ Lobi<br>○ Autre
098. Résultat du Questionnaire<br>*Sélectionnez le résultat du Questionnaire femme*	Toujours<br>○ Complété<br>○ Pas à la maison<br>○ Différé<br>○ Refusé<br>○ Complété en partie<br>○ Incapacité
