# Supplementary material for: Measurement of abortion safety using community-based surveys: Findings from three countries
Source: PLoS One. 2019 Nov 7;14(11):e0223146. doi: 10.1371/journal.pone.0223146 (PMC6837422; doi:10.1371/journal.pone.0223146)
Supplement: S8 Doc — (PDF) [file pone.0223146.s008.pdf]

S8 Doc.

RJR4-Female-Questionnaire-v10-jkp.xlsx

Question	Response
001a. Are you in the correct household? EA: [EA entered in the Household Questionnaire] Structure #: [Structure entered in the Household Questionnaire] Household #: [Household entered in the Household Questionnaire]	Always<br>○ Yes<br>○ No
002. Enter your ID below.<br>*Please record your ID*	002 = 0<br>--
003b. Record the correct date and time.	003 = 0<br>Day: -----------------------------------<br>Month: -----------------------------------<br>Year: -----------------------------------

The following information is from the Household Questionnaire. Please review to make sure you are interviewing the correct respondent. [ODK will display the LIST OF GEOGRAPHIES ENTERED, Enumeration Area, Structure Number, and Household Number entered into the Household Questionnaire linked to this Female Questionnaire.] Is the above information correct?	Always
District: ${level1_unlinked}	--
Tehsil / Taluk: ${level2_unlinked}	--
City / Town / Village: ${level3_unlinked}	--
Enumeration Area: ${EA_unlinked}	--
Structure number: ${structure_unlinked}	--
Household number: ${household_unlinked}	--
004b. Is the above information correct?	004 = 0<br>○ Yes<br>○ No

005. CHECK: You should be attempting to interview [Name of the interviewee]. Is that correct?<br>*If misspelled, select "yes" and update the name in question "011." If this is the wrong person, you have two options: (1) exit and ignore changes to this form. Open the correct form.*<br>*Or (2) find and interview the person whose name appears above.*	Always<br>○ Yes<br>○ No
006. Is the respondent present and available to be interviewed today?	Always<br>○ Yes<br>○ No
007. How well acquainted are you with the respondent?	006 = 1<br>○ Very well acquainted<br>○ Well acquainted<br>○ Not well acquainted<br>○ Not acquainted
008. Has the respondent previously participated in PMA 2020 surveys?	006 = 1<br>○ Yes<br>○ No<br>○ Do not know<br>○ No response
INFORMED CONSENT<br>*Find the woman between the age of 15-49 associated with this Female Respondent Questionnaire. The interview must have auditory privacy. Read the following greeting*	(${available} = 'yes') and (not(${unlinked}) or ${proceed_with_unlinked})
Namaskar! My name is _______________ and I am working for the IIHMR University Jaipur in collaboration with local Partners. I belong to this/nearby village. We are conducting a local survey that asks women about various reproductive health issues, including knowledge, attitudes, and use of contraception contraception and abortion, the result of which would help to identify gaps in family planning and health services that affect regular availability and	(${available} = 'yes')

quality of information, services, and products. Some of these questions, in particular the abortion questions, have been added expressly for research purposes. This survey is conducted across Rajasthan. From every enumeration area 35 households have been randomly selected your house was one of those households selected. Though we would very much appreciate your participation in the survey, your refusal to take part in this survey will have no repercussions on you. Information provided by you would help us inform the government to better plan health services. There is no physical risk associated with participating in this survey; however, some participants may experience distress in sharing information on personal and sometimes sensitive topics. If you agree to take part in this study, it will not benefit you directly. However, we believe that information gathered in this study will help us and other groups like us to develop programs that will improve the lives of men and women in communities in Rajasthan and across India, thus we hope that you would take part in this survey. Whatever information you provide will be kept confidential and only fully de-identified data will be used when conducting analyses, presenting results, or sharing data. We will never use your name while analyzing the data. The tracking sheet and the database with your responses will be kept on password-protected computers and on a secure server. Participation in this study, both the standard survey and the research questions, is entirely voluntary, if you do not wish to answer any question feel free to inform me and I would skip to the next question. You can choose not to participate at all or to end the interview at any point. The survey usually takes between 30 to 40 minutes to complete. If you have any questions about the study and your right as a research participant, you may ask me now or you may also contact Dr. Anoop Khanna at IIHMR University, in Jaipur, Rajasthan at +91-141-3924738.	
009a. Provide a paper copy of the Consent Form to the respondent and explain it. Then, ask: May I begin the interview now?	006 = 1<br>○ Yes<br>○ No

	(${begin_interview}='yes')
009b. Respondent's signature<br>*Please ask the respondent to sign or check the box in agreement of their participation.*	009a = 1<br>--

010. Interviewer's ID Please record your ID as a witness to the consent process. You previously entered "[Interviewer's ID]."	${consent_obtained} and (${your_name_check} = 'no')<br>--

Section 1 – Respondent's Background, Marital Status, Household characteristics

Now I would like to ask about your background and socioeconomic conditions.

	${consent_obtained}
101. In what month and year were you born? The age in the household roster is [AGE]	009a = 1
101. In what month and year were you born?	009a = 1
Month:	○ Do not know<br>○ January<br>○ February<br>○ March<br>○ April<br>○ May<br>○ June<br>○ July<br>○ August<br>○ September<br>○ October<br>○ November<br>○ December
Year:	

102. How old were you at your last birthday?	Year: 009a = 1
103. What is the highest level of school you attended?<br>*Only record formal schooling. Do not record bible or koranic school or short courses.*	009a = 1<br>○ Never attended<br>○ Primary<br>○ Secondary<br>○ Higher secondary<br>○ Graduate and above<br>○ No response
AH101a. Are you still attending school?	(${school} = 'primary') or (${school} = 'secondary') or (${school} = 'higher') or (${school} = 'po …<br>○ Yes<br>○ No<br>○ No response
AH101b. Do you plan to return to school?	${enrolled} = 'no'<br>○ Yes<br>○ No<br>○ Do not know<br>○ No response
AH102. At what age did you leave school?<br>*Enter -99 for no response.*	${enrolled} = 'no'
104. Are you currently married or living together with a man as if married?<br>*Probe: If no, ask whether the respondent is divorced, separated, or widowed.*	009a = 1<br>○ Yes, currently married<br>○ Yes, married, gauna not performed<br>○ Yes, living with a man<br>○ Not currently in union: Divorced / separated<br>○ Not currently in union: Widow<br>○ No, never in union<br>○ No response
105. Have you been married or lived with a man only once or more than once?	104 ≠ 5<br>○ Only once<br>○ More than once<br>○ No response

	(${marriage_history} = 'more_than_once')
106a. In what month and year did you start living with your FIRST husband / partner?<br>*Select 'Do not know' for month and '2020' for year to indicate 'No Response'.*	105=2
Month:	○ Do not know<br>○ January<br>○ February<br>○ March<br>○ April<br>○ May<br>○ June<br>○ July<br>○ August<br>○ September<br>○ October<br>○ November<br>○ December
Year:	Year:

106b. CHECK: Based on the response you entered in 106a, the respondent was possibly 15 years old or younger than 15 at the time of her first marriage.<br>Did you enter 106a correctly?	106a age at marriage ≤15<br>○ Yes<br>○ No

	(${marriage_history} = 'once') or (${marriage_history} = 'more_than_once')
107a. Now I would like to ask about when you started living with your CURRENT or MOST RECENT husband / partner. In what month and year was that?<br>*Select 'Do not know' for month and '2020' for year to indicate 'No Response'.*	105 = 1 or 2
Month:	○ Do not know<br>○ January<br>○ February<br>○ March<br>○ April<br>○ May<br>○ June<br>○ July<br>○ August<br>○ September<br>○ October<br>○ November<br>○ December
Year:	Year: ________________

107b. CHECK: Based on the response you entered in 107a, the respondent was possibly 15 years old or younger at the time of her current or most recent marriage.<br>Did you enter 107a correctly?	107a age at marriage ≤15<br>○ Yes<br>○ No
108. Does your husband / partner have other wives or does he live with other women as if married?	104 = 1 or 2<br>○ Yes<br>○ No<br>○ Do not know<br>○ No response

Section 2 – Reproduction, Pregnancy & Fertility Preferences

Now I would like to ask about all the births you have had during your life.

200. Have you ever given birth?	009a = 1<br>○ Yes<br>○ No<br>○ No response
201. How many times have you given birth?<br>*Enter -99 for no response.*	200 = 1<br>________________

	(${birth_events} > 1)
205. When was your FIRST birth?<br>*Please record the date of the FIRST birth. The date should be found by calculating backwards from memorable events if needed.*<br>*Select 'Do not know' for month and '2020' for year to indicate 'No Response'.*	201 > 1
Month:	○ Do not know<br>○ January<br>○ February<br>○ March<br>○ April<br>○ May<br>○ June<br>○ July<br>○ August<br>○ September<br>○ October<br>○ November<br>○ December
Year:	Year: ________________

	(${birth_events} > 0)
206. When was your MOST RECENT birth?<br>*Select 'Do not know' for month and '2020' for year to indicate 'No Response'.*	201 > 1
Month:	Do not know<br>January<br>February<br>March<br>April<br>May<br>June<br>July<br>August<br>September<br>October<br>November<br>December
Year:	Year: ________________

210a. Are you pregnant now?	009a = 1<br>Yes<br>No<br>Unsure<br>No response

	${pregnant} = 'yes'
210b. How many months pregnant are you?	210a = 1
The most recent birth was: [Date of most recent birth]	${recent_birth} != ''
#####<br>*Please record the number of completed months. Enter -88 for do not know, -99 for No response.*	________________

209. When did your last menstrual period start?<br>*If you select days, weeks, months, or years, you will enter a number for X on the next screen. Enter 0 days for today, not 0 weeks/months/years.*	009a = 1<br>X days ago<br>X weeks ago<br>X months ago<br>X years ago<br>Menopausal / Hysterectomy<br>Before last birth<br>Never menstruated<br>No response
209a. Enter [days / weeks / months / years]<br>*Enter 0 days for today, not 0 weeks/months/years.*	(${menstrual_period} = 'days') or (${menstrual_period} = 'weeks') or (${menstrual_period} = 'month …<br>________________

	${ever_birth} = 'yes' or ${pregnant} = 'yes'
213a. Now I would like to ask a question about your last birth.	201 > 0 AND 210a ≠ 1 201 > 0 AND 210a ≠ 1 201 > 0 AND 210a ≠ 1
213b. Now I would like to ask a question about your current pregnancy.	210a = 1
At the time you became pregnant, did you want to become pregnant then, did you want to wait until later, or did you not want to have any more children at all?	(201 > 1 AND 210a ≠ 1) OR (200 = 1 AND 201a = 1)
At the time you became pregnant, did you want to become pregnant then, did you want to wait until later, or did you not want to have any children at all?	(201 = 1 AND 210a ≠ 1) OR (200 = 2 AND 201a = 1)
#####	Then<br>Later<br>Not at all<br>No response

Now I have some questions about the future.	${consent_obtained}
211a. Would you like to have a child or would you prefer not to	210a ≠ 1

have any children?	○ Have a child<br>○ Prefer no children<br>○ Says she can't get pregnant<br>○ Undecided / Don't know<br>○ No response
211a. Would you like to have another child or would you prefer not to have any more children?	210a ≠ 1<br>○ Have another child<br>○ No more<br>○ Says she can't get pregnant<br>○ Undecided / Don't know<br>○ No response
211b. After the child you are expecting now, would you like to have another child or would you prefer not to have any more children?	210a = 1<br>○ Have another child<br>○ No more<br>○ Says she can't get pregnant<br>○ Undecided / Don't know<br>○ No response
212a. How long would you like to wait from now before the birth of a child?<br>*If you select months or years, you will enter a number for X on the next screen.*<br>*Select "Years" if more than 36 months.*<br>*Please check that you correctly entered the value for months/years.*	211a = 1<br>○ X months<br>○ X years<br>○ Soon/now<br>○ Says she can't get pregnant<br>○ Other<br>○ Don't know<br>○ No response
212b. After the birth of the child you are expecting now, how long would you like to wait before the birth of another child?<br>*If you select months or years, you will enter a number for X on the next screen.*<br>*Select "Years" if more than 36 months.*<br>*Please check that you correctly entered the value for months/years.*	211b = 1<br>○ X months<br>○ X years<br>○ Soon/now<br>○ Says she can't get pregnant<br>○ Other<br>○ Don't know<br>○ No response
212c. Enter the number of [Months OR Years] you would like to wait:	212a OR 212b = 'months' OR 'year'<br>________________

Section 3 – Contraception

Now I would like to talk about family planning - the various ways or methods that a couple can use to delay or avoid a pregnancy.

An image will appear on the screen for some methods. If the respondent says that she has not heard of the method or if she hesitates to answer, read the probe aloud and show her the image, if available.

301a. Have you ever heard of female sterilization?<br>PROBE: Women can have an operation to avoid having any more children.	009a = 1<br>○ Yes<br>○ No<br>○ No response
301b. Have you ever heard of male sterilization?<br>PROBE: Men can have an operation to avoid having any more children.	009a = 1<br>○ Yes<br>○ No<br>○ No response
301c. Have you ever heard of the contraceptive implant?<br>PROBE: Women can have one or several small rods placed in her upper arm by a doctor or nurse, which can prevent pregnancy for one or more years.<br>[implant_150x300.png]	009a = 1<br>○ Yes<br>○ No<br>○ No response
301d. Have you ever heard of the IUD / PPIUD?<br>PROBE: Women can have a loop or coil placed inside them by a doctor or a nurse.<br>[IUD_150x300.png]	009a = 1<br>○ Yes<br>○ No<br>○ No response

301e. Have you ever heard of injectables?<br>PROBE: Women can have an injection by a health provider that stops them from becoming pregnant for one or more months.<br>[sayana_depo_150x300.jpg]	009a = 1<br>○ Yes<br>○ No<br>○ No response
301f. Have you ever heard of the (birth control) pill?<br>PROBE: Women can take a pill every day to avoid becoming pregnant.<br>[pill_150x300.png]	009a = 1<br>○ Yes<br>○ No<br>○ No response
301g. Have you ever heard of emergency contraception?<br>PROBE: As an emergency measure after unprotected sexual intercourse women can take special pills at any time within 3-5 days to prevent pregnancy.	009a = 1<br>○ Yes<br>○ No<br>○ No response
301h. Have you ever heard of male condoms / nirodh?<br>PROBE: Men can put a rubber sheath on their penis before sexual intercourse.<br>[male_condom_150x300.png]	009a = 1<br>○ Yes<br>○ No<br>○ No response
301i. Have you ever heard of female condoms?<br>PROBE: Women can put a sheath in their vagina before sexual intercourse.<br>[female_condom_150x300.png]	009a = 1<br>○ Yes<br>○ No<br>○ No response
301l. Have you ever heard of the standard days method or Cycle Beads?<br>PROBE: A Woman can use a string of colored beads to know the days she can get pregnant. On the days she can get pregnant, she and her partner use a condom or do not have sexual intercourse.<br>[SDM-beads_only.png]	009a = 1<br>○ Yes<br>○ No<br>○ No response
301m. Have you ever heard of the Lactational Amenorrhea Method or LAM?	009a = 1<br>○ Yes<br>○ No<br>○ No response
301n. Have you ever heard of the rhythm method?<br>PROBE: Women can avoid pregnancy by not having sexual intercourse on the days of the month they think they can get pregnant.	009a = 1<br>○ Yes<br>○ No<br>○ No response
301o. Have you ever heard of the withdrawal method?<br>PROBE: Men can be careful and pull out before climax.	009a = 1<br>○ Yes<br>○ No<br>○ No response
301p. Have you ever heard of any other ways or methods that women or men can use to avoid pregnancy?	009a = 1<br>○ Yes<br>○ No<br>○ No response
302a. Are you or your partner currently doing something or using any method to delay or avoid getting pregnant?	210a ≠ 1 AND 009a = 1 210210a ≠ 1 AND 009a = 1 210a ≠ 1 AND 009a = 1 ≠ 1 AND 009a = 1 210a ≠ 1 AND 009a = 1<br>○ Yes<br>○ No<br>○ No response

	${current_user} = 'yes'
302b. Which method or methods are you using?<br>PROBE: Anything else?<br>*Select all methods mentioned. SCROLL TO THE BOTTOM to see all choices.*	302a = 1<br>☐ Female sterilization<br>☐ Male sterilization<br>☐ Implant<br>☐ IUD / PPIUD<br>☐ Injectables<br>☐ Pill<br>☐ Emergency Contraception<br>☐ Male condom / nirodh<br>☐ Female condom<br>☐ Standard Days/Cycle beads<br>☐ LAM

	Rhythm method<br>Withdrawal<br>Other traditional method<br>No response

LCL_301. Have you ever been sterilized?<br>*If yes, go back and select that she is a current user and select the method female sterilization*	(${consent_obtained}) and (${pregnant} != 'yes') and ((${current_user} != 'yes') or not(selected($ …<br>Yes<br>No<br>No response
CALC_CM. CALCULATE: CURRENT METHOD<br>THIS WILL NOT APPEAR ON THE SCREEN<br>*ODK will identify the most effective method currently being used by the respondent by selecting the highest method in the choice list.*	302a=1 AND 302b ≠-99<br>Female sterilization<br>Male sterilization<br>Implant<br>IUD / PPIUD<br>Injectables<br>Pill<br>Emergency Contraception<br>Male condom / nirodh<br>Female condom<br>Standard Days/Cycle beads<br>LAM<br>Rhythm method<br>Withdrawal<br>Other traditional method<br>No response
302c. Does your husband/partner know that you are using [CURRENT METHOD]?	302a = 1<br>Yes<br>No<br>No response
302c. Does your husband/partner know that you are using family planning?	302a = -99<br>Yes<br>No<br>No response
303. Did the provider tell you or your partner that this method was permanent?	302b = male or female sterilization<br>Yes<br>No<br>No response
305a. You said that you are not currently using a contraceptive method. Do you think you will use a contraceptive method to delay or avoid getting pregnant at any time in the future?	302a ≠1 AND 210a ≠1<br>Yes<br>No<br>No response
305b. Do you think you will use a contraceptive method to delay or avoid getting pregnant at any time in the future?	302a ≠1 AND 201a = 1<br>Yes<br>No<br>No response
306a. In the last 12 months, have you ever done something or used a method to delay or avoid getting pregnant?	302a ≠1<br>Yes<br>No<br>No response

	${recent_user} = 'yes'
306b. Which method did you use most recently?<br>PROBE: Anything else?<br>*Select most effective method (highest method in list). Scroll to bottom to see all choices.*	306a = 1<br>Implant<br>IUD / PPIUD<br>Injectables<br>Pill<br>Emergency Contraception<br>Male condom / nirodh<br>Female condom<br>Standard Days/Cycle beads

- LAM
- Rhythm method
- Withdrawal
- Other modern method
- No response

307. Before you started using [CURRENT METHOD / MOST RECENT METHOD], had you discussed the decision to delay or avoid pregnancy with your husband/partner?

302a = 1 OR 306a = 1

- Yes
- No
- Do not know
- No response

308. Would you say that using contraception is mainly your decision, mainly your husband/partner's decision or did you both decide together?

302a = 1

- Mainly respondent
- Mainly husband/partner
- Joint decision
- Other
- No response

308a. The last time you received your [CURREN METHOD /MOST RECENT METHOD], how much did you have to pay out of pocket, including any fees paid for the method, supplies or services, and transportation?

Enter all prices in rupees. Enter -88 if respondent does not know, -99 for no response.

302a = 1 OR 306a = 1

	${current_user} = 'yes'
309a. Since what month and year have you been using [CURRENT METHOD / MOST RECENT METHOD] without stopping?<br>*Calculate backwards from memorable events if needed.*	302a = 1
Most Recent Birth: [mm-yyyy]	${recent_birth} != ''
Current Marriage: [mm-yyyy]	${husband_cohabit_start_recent} != ''
Month:	○ Do not know<br>○ January<br>○ February<br>○ March<br>○ April<br>○ May<br>○ June<br>○ July<br>○ August<br>○ September<br>○ October<br>○ November<br>○ December
Year:	Year: ________________

	${recent_user} = 'yes'
309b. When did you stop using [CURRENT METHOD / MOST RECENT METHOD]?<br>*Please record the date. The date should be found by calculating backwards from memorable events if needed.*<br>*Select 'Do not know' for month and '2020' for year to indicate No Response.*	306a = 1
Month:	○ Do not know<br>○ January<br>○ February<br>○ March<br>○ April<br>○ May<br>○ June<br>○ July<br>○ August<br>○ September

	◯ October<br>◯ November<br>◯ December
Year:	Year: ________________

	${recent_user} = 'yes'
309c. In what month and year had you started using [CURRENT METHOD / MOST RECENT METHOD] before stopping?<br>*Calculate backwards from memorable events if needed.*<br>*Select 'Do not know' for month and '2020' for year to indicate No Response.*	306a = 1
Most Recent Birth: [mm-yyyy]	${recent_birth} != ''
Current Marriage: [mm-yyyy]	${husband_cohabit_start_recent} != ''
Month:	◯ Do not know<br>◯ January<br>◯ February<br>◯ March<br>◯ April<br>◯ May<br>◯ June<br>◯ July<br>◯ August<br>◯ September<br>◯ October<br>◯ November<br>◯ December
Year:	Year: ________________

309d. CHECK: Just to make sure I have this correct, you used [CURRENT METHOD / MOST RECENT METHOD] continuously between [START DATE] and [END DATE] without stopping, is that correct?	306a = 1<br>◯ Yes<br>◯ No
GO BACK TO THE PREVIOUS SCREEN AND PROBE TO DETERMINE THE PERIOD OF MOST RECENT CONTINUOUS USE.<br>*Suggested probes: - When was the last time you used [METHOD]? - How long had you been using [METHOD] without stopping*	309d = 0
310. Why did you stop using [CURRENT METHOD / MOST RECENT METHOD?	306a = 1<br>☐ Infrequent sex / husband away<br>☐ Became pregnant while using<br>☐ Wanted to become pregnant<br>☐ Husband / partner disapproved<br>☐ Wanted a more effective method<br>☐ No method available<br>☐ Health concerns<br>☐ Fear of side effects<br>☐ Lack of access / too far<br>☐ Costs too much<br>☐ Inconvenient to use<br>☐ Fatalistic<br>☐ Difficult to get pregnant / menopausal<br>☐ Interferes with body's processes<br>☐ Other<br>☐ Don't know<br>☐ No response

	${current_or_recent_user} and (${current_recent_method} != 'LAM') and (${current_recent_method} != …
311a. You first started using [CURRENT METHOD / MOST RECENT METHOD] on [DATE FROM FQ309a OR 309c] Where did you or your partner get it at that time?<br>*Scroll to bottom to see all choices.*	(CALC_CM ≠ 14, 30, 31, 39, -99) OR (306b ≠ 14, 30, 31, 39, -99)<br>◯ Govt./Municipal Hospital<br>◯ Govt. Dispensary

	UFWC/UHC/UHP<br>CHC/Rural Hospital/PHC<br>Sub-Centre/ANM<br>Govt. Mobile clinic<br>Camp<br>Anganwadi/ICDS Centre<br>ASHA<br>Other Community-Based Worker<br>NGO or Trust Hospital/Clinic<br>Pvt hospital<br>Pvt. Doctor/Clinic<br>Pvt. Mobile Clinic<br>Vaidya/Hakim/Homeopath (Ayush)<br>Traditional Healer<br>Pharmacy/Drugstore<br>Dai (TBA)<br>Shop<br>Friend / parent / relative<br>Other<br>Do not know<br>No response
312a. When you obtained your [CURRENT METHOD / MOST RECENT METHOD], were you told by the provider about side effects or problems you might have with a method to delay or avoid getting pregnant?	311a ≠ .<br>Yes<br>No<br>No response
312b. Were you told what to do if you experienced side effects or problems?	312a = 1<br>Yes<br>No<br>No response
313. At that time, were you told by the family planning provider about methods of family planning other than the [CURRENT METHOD / MOST RECENT METHOD] that you could use?	311a ≠ . OR 311b ≠ .<br>Yes<br>No<br>Do not know<br>No response
314a. During that visit, did you obtain the method you wanted to delay or avoid getting pregnant?	311a ≠ .<br>Yes<br>No<br>No response
314c. Why didn't you obtain the method you wanted?	314a = 0<br>Method out of stock that day<br>Method not available at all<br>Provider not trained to provide the method<br>Provider recommended a different method<br>Not eligible for method<br>Decided not to adopt a method<br>Too costly<br>Other<br>No response
315a. During that visit, who made the final decision about what method you got?	311a ≠ .<br>You alone<br>Provider<br>Partner<br>You and provider<br>You and partner<br>Other<br>Do not know<br>No response
315b. Who made the final decision to use rhythm?	311b ≠ .

	◯ You alone<br>◯ Provider<br>◯ Partner<br>◯ You and provider<br>◯ You and partner<br>◯ Other<br>◯ Do not know<br>◯ No response
315b. Who made the final decision to use LAM?	311b ≠ .<br>◯ You alone<br>◯ Provider<br>◯ Partner<br>◯ You and provider<br>◯ You and partner<br>◯ Other<br>◯ Do not know<br>◯ No response
316. Would you return to this provider? Provider: [Type of provider selected in 311a or 311b]	311a ≠ 35 or 96<br>◯ Yes<br>◯ No<br>◯ Do not know<br>◯ No response
317. Would you refer your relative or friend to this provider / facility? Provider: [Type of provider selected in 311a or 311b]	311 a ≠ 34 or 96<br>◯ Yes<br>◯ No<br>◯ Do not know<br>◯ No response
SW_1a. Right before you started using [CURRENT METHOD / MOST RECENT METHOD] in [MOIS/ANNEE], were you doing something or using any method to delay or avoid getting pregnant?	302a = 1 OR 306a = 1<br>◯ Yes<br>◯ No<br>◯ No response
SW_1b. Which method were you using?	SW_1a = 1<br>◯ Implant<br>◯ IUD / PPIUD<br>◯ Injectables<br>◯ Pill<br>◯ Emergency Contraception<br>◯ Male condom / nirodh<br>◯ Female condom<br>◯ Standard Days/Cycle beads<br>◯ LAM<br>◯ Rhythm method<br>◯ Withdrawal<br>◯ Other modern method<br>◯ No response
PP_1. Since the birth of your child in [DATE OF MOST RECENT BIRTH], have you ever done something or used any method to delay or avoid getting pregnant?	child born in last 2 years AND 302a ≠ 1<br>◯ Yes<br>◯ No<br>◯ No response
PP_2. How long after the birth in [DATE OF MOST RECENT BIRTH] did you start doing something or start using a method?<br>*Enter 0 days for today. You will enter a number for X on the next screen.*	PP_1 = 1 OR (302a = 1 AND child born in the last 2 years)<br>◯ X days after<br>◯ X weeks after<br>◯ X months after<br>◯ X years after<br>◯ No response
PP_2. Enter [METHOD].<br>*If today, enter zero days only, not zero weeks/months/years.*	${pp_method_units} = 'days' or ${pp_method_units} = 'weeks' or ${pp_method_units} = 'months' or ${ …<br>--

PP_3. What was the method?	PP_2 ≠ .<br>◯ Female sterilization<br>◯ Male sterilization<br>◯ Implant<br>◯ IUD / PPIUD<br>◯ Injectables<br>◯ Pill<br>◯ Emergency Contraception<br>◯ Male condom / nirodh<br>◯ Female condom<br>◯ Standard Days/Cycle beads<br>◯ LAM<br>◯ Rhythm method<br>◯ Withdrawal<br>◯ Other traditional method<br>◯ No response
319. Have you ever done anything or tried in any way to delay or avoid getting pregnant?	306a ≠ 1 OR 302a ≠ 1<br>◯ Yes<br>◯ No<br>◯ No response
320. How old were you when you first used a method to delay or avoid getting pregnant? The respondent said she was [AGE] years old at her last birthday.<br>*Enter the age in years. Enter -88 if the respondent does not know. Enter -99 if there is no response. Cannot be younger than 9.*	302a = 1 OR 306a =1 OR 319 = 1<br>--
321. How many living children did you have at that time, if any? Note: the respondent said that she gave birth [NUMBER OF LIFE BIRTHS] times in 201.<br>*Enter -99 for no response.*	Age in 320 ≥ 9 AND 200 = 1<br>--

	${fp_ever_used} = 'yes'
322. Which method did you first use to delay or avoid getting pregnant?<br>*Do not read the method choices. Scroll to bottom to see all choices.*	319 = 1<br>◯ Female sterilization<br>◯ Male sterilization<br>◯ Implant<br>◯ IUD / PPIUD<br>◯ Injectables<br>◯ Pill<br>◯ Emergency Contraception<br>◯ Male condom / nirodh<br>◯ Female condom<br>◯ Standard Days/Cycle beads<br>◯ LAM<br>◯ Rhythm method<br>◯ Withdrawal<br>◯ Other traditional method<br>◯ No response

322a. Have you used emergency contraception at any time in the last 12 months?<br>PROBE: As an emergency measure after unprotected sexual intercourse women can take special pills at any time within 3-5 days to prevent pregnancy.	302b ≠ emergency contraception OR 306b ≠ 8<br>◯ Yes<br>◯ No<br>◯ No response

	((${current_user} = 'no')) and (((${more_children_none} = 'no_children') or …
323a. You said that you do not want a child soon and that you are not using a method to avoid pregnancy.	302a = 0 AND ((212a or 212b > 2 years) OR (211a or 211b = 2))
323a. You said that you do not want another child soon and that you are not using a method to avoid pregnancy.	302a = 0 AND ((212a or 212b > 2 years) OR (211a or 211b = 2))
323a. You said that you do not want any children and that you are not using a method to avoid pregnancy.	302a = 0 AND ((212a or 212b > 2 years) OR (211a or 211b = 2))
323a. You said that you do not want any more children and that you are not using a method to avoid pregnancy.	302a = 0 AND ((212a or 212b > 2 years) OR (211a or 211b = 2))
Can you tell me why you are not using a method to prevent	☐ Not married

pregnancy?<br>PROBE: Any other reason?<br>*RECORD ALL REASONS MENTIONED Cannot select "Not Married" if 104 is "Yes, currently married".*<br>*Scroll to bottom to see all choices.*	☐ Infrequent sex / Not having sex<br>☐ Menopausal / Hysterectomy<br>☐ Subfecund / Infecund<br>☐ Not menstruated since last birth<br>☐ Breastfeeding<br>☐ Husband away for multiple days<br>☐ Up to God / fatalistic<br>☐ Respondent opposed<br>☐ Husband / partner opposed<br>☐ Others opposed<br>☐ Religious prohibition<br>☐ Knows no method<br>☐ Knows no source<br>☐ Fear of side effects<br>☐ Health concerns<br>☐ Lack of access / too far<br>☐ Costs too much<br>☐ Preferred method not available<br>☐ No method available<br>☐ Inconvenient to use<br>☐ Interferes with body's processes<br>☐ Other<br>☐ Don't know<br>☐ No response

323b. Would you say that not using contraception is mainly your decision, mainly your husband/partner's decision or did you both decide together?	302a ≠ 1<br>○ Mainly respondent<br>○ Mainly husband/partner<br>○ Joint decision<br>○ Other<br>○ No response
324. In the last 12 months, were you visited by a anganwadi worker, ASHA, or other community health worker who talked to you about family planning?	${consent_obtained}<br>○ Yes<br>○ No<br>○ No response
325a. In the last 12 months, have you visited a health facility or camp for care for yourself?<br>*For any health services.*	009a = 1<br>○ Yes<br>○ No<br>○ No response
325a. In the last 12 months, have you visited a health facility or camp for care for yourself or your children?<br>*For any health services.*	009a = 1<br>○ Yes<br>○ No<br>○ No response
325b. Did any staff member at the health facility speak to you about family planning methods?	325a = 1<br>○ Yes<br>○ No<br>○ No response

009a = 1

326. In the last few months have you:

	Yes	No	No response
326a. Heard about family planning on the radio?	○	○	○
326b. Seen anything about family planning on the television?	○	○	○
326c. Read about family planning in a newspaper or magazine?	○	○	○
326d. Received a voice or text message about family planning on a mobile phone?	○	○	○

Section 4 – Sexual Activity

CHECK FOR THE PRESENCE OF OTHERS. BEFORE CONTINUING, MAKE EVERY EFFORT

TO ENSURE PRIVACY.

Now I would like to ask some questions about sexual activity in order to gain a better understanding of some important life issues. Let me assure you again that your answers are completely confidential and will not be told to anyone. If we should come to any question that you don't want to answer, just let me know and we will go to the next question.	${consent_obtained}

	${consent_obtained}
401a. How old were you when you first had sexual intercourse?	309a = 1
Current age: [AGE]	
Number of live births: [NUMBER OF LIFE BIRTHS]	${birth_events} > 0
The respondent is pregnant	${pregnant} = 'yes'
Enter the age in years. *Enter -77 if she has never had sex. Enter -99 for no response. Enter -88 for do not know.*	--

	((${age_at_first_sex} >= 0) or (${age_at_first_sex} =-88) or (${age_at_first_sex} =-99))
402. When was the last time you had sexual intercourse?	401a ≠ -77

402. Enter [# days / weeks / months / years]. *If today, enter 0 days only, not 0 weeks/months/years.* *Must agree with the age of first sexual intercourse and the pregnancy status.*	401a ≠ -77 --
LCL_403. Depending on your body, if you have sex only once without using birth control, will you get pregnant? *Read response options aloud.*	${consent_obtained} ○ Definitely yes ○ Maybe yes ○ Maybe no ○ Definitely no ○ Don't know ○ No response
LCL_404. Depending on your body, if you have sex regularly, say twice a week, for a year without using birth control, will you get pregnant? *Read response options aloud.*	${consent_obtained} ○ Definitely yes ○ Maybe yes ○ Maybe no ○ Definitely no ○ Don't know ○ No response

Adolescent Health

AH_1. At what age can girls start marrying legally where you live?	(${consent_obtained}) and (${age} <= 19) --
AH_2. How much were you involved in the decision to get married – very much, not very much or not at all?	(${age} <= 19) and ((${marital_status} = 'currently_married') or (${marital_status} = 'currently_l … ○ Very much ○ Not very much ○ Not at all ○ No response
AH_3. How much do you think you will be involved in the decision to get married – very much, not very much or not at all?	(${age} <= 19) and (${marital_status} = 'never_married') ○ Very much ○ Not very much ○ Not at all ○ No response
AH_4. Did you drop out of school permanently because you got married?	(${age} <= 19) and ((${marital_status} = 'currently_married') or (${marital_status} = 'currently_l … ○ Yes ○ No ○ No response

AH_5. For the following question, please tell me if you strongly agree, agree, neither agree nor disagree, disagree, or strongly disagree:<br>I will wait to get married until I complete my education.	(${age} <= 19) and (${marital_status} = 'never_married') and((${enrolled} = 'yes') or (${return} …<br>○ Strongly agree<br>○ Agree<br>○ Neither agree nor disagree<br>○ Disagree<br>○ Strongly disagree<br>○ No response
AH_6. Looking back to the first time you had sexual intercourse, do you think you would have preferred to: have waited longer before having sex with anyone, not have waited so long, or was it the right time?	(${age} <= 19) and ((${marital_status} = 'currently_married') or (${marital_status} = 'currently_l …<br>○ Waited longer<br>○ Should not have waited<br>○ It was the right time<br>○ Not applicable<br>○ No response
AH_7. The first time you had sexual intercourse, would you say you and your partner were both equally willing to have sexual intercourse the first time or was one of you more willing than the other?	(${first_sex_timing} != 'na') and (${age} <= 19) and ((${marital_status} = 'currently_married') or …<br>○ Equally willing<br>○ Respondent more willing<br>○ Partner more willing<br>○ No response
AH_8. Which of these applied to you at the first time you had sex:<br>*Read the answer choices aloud. Select all that apply.*	(${first_sex_timing} != 'na') and (${age} <= 19) and ((${marital_status} = 'currently_married') or …<br>☐ I was curious<br>☐ I was carried away<br>☐ I was under the influence of a substance<br>☐ I was doing what was expected of me<br>☐ I was forced against my will<br>☐ None of the above<br>☐ No response
AH_9. The first time you had sex, how much were you involved in the decision to have sex – very much, not very much or not at all?	(${first_sex_timing} != 'na') and (${age} <= 19) and ((${marital_status} = 'currently_married') or …<br>○ Very much<br>○ Not very much<br>○ Not at all<br>○ No response
AH_10. Did you and your partner want to avoid a pregnancy the first time you had sexual intercourse?	(${first_sex_timing} != 'na') and (${age} <= 19) and ((${marital_status} = 'currently_married') or …<br>○ Yes<br>○ No<br>○ No response
AH_11. Did you or your partner do something or use any method to delay or avoid getting pregnant?	${first_sex_avoid_preg} = 'yes'<br>○ Yes<br>○ No<br>○ No response
AH_12. What method did you use?	${first_sex_method_yn} = 'yes'<br>○ Female sterilization<br>○ Male sterilization<br>○ Implant<br>○ IUD / PPIUD<br>○ Injectables<br>○ Pill<br>○ Emergency Contraception<br>○ Male condom / nirodh<br>○ Female condom

	◯ Standard Days/Cycle beads<br>◯ LAM<br>◯ Rhythm method<br>◯ Withdrawal<br>◯ Other traditional method<br>◯ No response
AH_13. How much do you think you will be involved in the decision to have sex for the first time – very much, not very much or not at all?	(${age} <= 19) and (${marital_status} = 'never_married')<br>◯ Very much<br>◯ Not very much<br>◯ Not at all<br>◯ No response
AH_14. For the following question, please tell me if you strongly agree, agree, neither agree nor disagree, disagree, or strongly disagree:<br>I wanted to have my first child as soon as possible because I wanted to be a mother.	(${age} <= 19) and ((${ever_birth} = 'yes') or (${pregnant} = 'yes'))<br>◯ Strongly agree<br>◯ Agree<br>◯ Neither agree nor disagree<br>◯ Disagree<br>◯ Strongly disagree<br>◯ No response
AH_15. For the following question, please tell me if you strongly agree, agree, neither agree nor disagree, disagree, or strongly disagree:<br>I want to have a child as soon as possible because I want to be a mother.	(${age} <= 19) and (${ever_birth} = 'no') and (${pregnant} = 'no')<br>◯ Strongly agree<br>◯ Agree<br>◯ Neither agree nor disagree<br>◯ Disagree<br>◯ Strongly disagree<br>◯ No response
AH_16. Did you drop out of school permanently because of your pregnancy?	(${age} <= 19) and (${ever_birth} = 'no') and (${pregnant} = 'no') and (${return} = 'no')<br>◯ Yes<br>◯ No<br>◯ No response
AH_17. Do you plan to complete your education before you have your first child?	(${age} <= 19) and (${marital_status} = 'never_married')<br>◯ Yes<br>◯ No<br>◯ Do not know<br>◯ No response
AH_18. Do you think you will use a contraceptive method to delay or avoid getting pregnant in the next 12 months?	(${consent_obtained}) and (${age} <= 19) and (${pregnant} != 'yes') and (${current_user} != 'yes') …<br>◯ Yes<br>◯ No<br>◯ Do not know<br>◯ No response
AH_19. Do you know a place where you can obtain a method of contraception?	(${consent_obtained}) and (${age} <= 19)<br>◯ Yes<br>◯ No<br>◯ No response

	(${consent_obtained}) and (${age} <= 19)
For the following questions, please tell me if you strongly agree, agree, neither agree nor disagree, disagree, or strongly disagree:	
AH_20. Using contraception allows young women to prepare for a family.	◯ Strongly agree<br>◯ Agree<br>◯ Neither agree nor disagree<br>◯ Disagree<br>◯ Strongly disagree<br>◯ No response
AH_21. Using contraception, a young couple can love each other with peace of mind.	◯ Strongly agree<br>◯ Agree<br>◯ Neither agree nor disagree

	Disagree<br>Strongly disagree<br>No response
AH_22. Contraception is only for married women.	Strongly agree<br>Agree<br>Neither agree nor disagree<br>Disagree<br>Strongly disagree<br>No response
AH_23. Contraception is only for women who don't want any more children.	Strongly agree<br>Agree<br>Neither agree nor disagree<br>Disagree<br>Strongly disagree<br>No response
AH_24. Using contraception can cause infertility or harm a woman's health.	Strongly agree<br>Agree<br>Neither agree nor disagree<br>Disagree<br>Strongly disagree<br>No response
AH_25. Adolescents or young women who use contraception are seen as promiscuous.	Strongly agree<br>Agree<br>Neither agree nor disagree<br>Disagree<br>Strongly disagree<br>No response
AH_26. I would feel too shy or embarrassed to get contraception at a clinic or elsewhere.	Strongly agree<br>Agree<br>Neither agree nor disagree<br>Disagree<br>Strongly disagree<br>No response

	(${consent_obtained}) and (${age} <= 19)
I would be interested in using the following options in the future if they were legally available:	
AH_27. A method of contraception I could self-inject every three months.	Yes<br>Maybe<br>No<br>No response
AH_28. A contraceptive pill I could get at the pharmacy without a prescription.	Yes<br>Maybe<br>No<br>No response
AH_29. A pill I could get without a prescription to bring back my period if I was worried I was pregnant.	Yes<br>Maybe<br>No<br>No response

AH_30. Have you heard or seen any messages in the media, or elsewhere about child marriage in the last 12 months?	${consent_obtained}<br>Yes<br>No<br>No response
AH_31. In your opinion, how much should the girl influence the decision about when to get married – very much, not very much or not at all?	${consent_obtained}<br>Very much<br>Not very much<br>Not at all<br>No response

AH_32. How much should the girl influence the decision to start a family – very much, not very much or not at all?	${consent_obtained}<br>○ Very much<br>○ Not very much<br>○ Not at all<br>○ No response
AH_33. How many years of education should girls complete?<br>*Record answer in years.*<br>*Enter -88 for do not know. Enter -99 for no response.*	${consent_obtained}<br>--
AH_34. How many years of education should boys complete?<br>*Record answer in years.*<br>*Enter -88 for do not know. Enter -99 for no response.*	${consent_obtained}<br>--

Section 7.1 – Confidantes

701. Now I want to ask some questions about your closest female friends or relatives. These are women whom you share very personal information with and who also share their very personal information with you. How many female friends or relatives like this do you have in Rajasthan who are between the ages of 15 and 49?<br>*Enter -88 for do not know, -99 for no response.*	${consent_obtained}<br>--
702a. Please picture your closest female friend or relative in Rajasthan. Again, this is a woman between the ages of 15 and 49 with whom you share very personal information and who also shares her very personal information with you. For ease of referencing this woman, please provide a fake name.	${friend_count} > 0<br>--
703a. How old was ${friend1_name} at her last birthday?<br>*Enter -88 for do not know, -99 for no response.*	${friend_count} > 0<br>--
704a. What is the highest level of school ${friend1_name} has ever attended?	${friend_count} > 0<br>○ Never attended<br>○ Primary<br>○ Secondary<br>○ Higher secondary<br>○ Graduate and above<br>○ No response
702b. Please picture your next closest female friend or relative in Rajasthan. Again, this is a woman between the ages of 15 and 49 with whom you share very personal information and who also shares very personal information with you. For ease of referencing this woman, please provide a fake name.	${friend_count} > 1<br>--
703b. How old was ${friend2_name} at her last birthday?<br>*Enter -88 for do not know, -99 for no response.*	${friend_count} > 1<br>--
704b. What is the highest level of school ${friend2_name} has ever attended?	${friend_count} > 1<br>○ Never attended<br>○ Primary<br>○ Secondary<br>○ Higher secondary<br>○ Graduate and above<br>○ No response

Section 7.2 – Abortion

CHECK FOR THE PRESENCE OF OTHERS. BEFORE CONTINUING, MAKE EVERY EFFORT TO ENSURE PRIVACY.

The next series of questions are about things women in your community do to remove a pregnancy. This is a common experience in Rajasthan and we simply want to better understand what women do to remove a pregnancy. I want to remind you that this survey is completely confidential and anonymous and your responses will not be shared with anyone. If we should come to any question that you don't want to answer, just let me know and I will skip to the next question.<br>*Press OK to continue.*	${consent_obtained}<br>☐ OK

705. Sometimes women are worried they are pregnant or get pregnant when they do not want to be and they do something to remove the pregnancy. How common is this in the community where you currently live?<br>*Read response options aloud.*	${consent_obtained}<br>○ Very common<br>○ Somewhat common<br>○ Not very common<br>○ Not at all common<br>○ Do not know<br>○ No response
706. In the community where you currently live, what are the ways a woman who is pregnant or worried that she is pregnant can remove a pregnancy? Anything else?<br>*Do not read options aloud. Select all that apply.*	${consent_obtained}<br>☐ Surgical procedure, like D&C or "cleaning"<br>☐ Pills called mifepristone or misoprostol, for example "500 pills" or "700 pills"<br>☐ Pills you take when you have a fever like antibiotics or anti-malarial medicine, for example Combiflam, Paracetamol, or pills that come in a green pack<br>☐ Other pills<br>☐ Traditional methods, like herbs<br>☐ Home remedies<br>☐ Insert materials into the vagina<br>☐ Other<br>☐ Do not know<br>☐ No response
707. Which of these ways is the most common?	count-selected(${abt_ways}) > 1<br>○ Surgical procedure, like D&C or "cleaning"<br>○ Pills called mifepristone or misoprostol, for example "500 pills" or "700 pills"<br>○ Pills you take when you have a fever like antibiotics or anti-malarial medicine, for example Combiflam, Paracetamol, or pills that come in a green pack<br>○ Other pills<br>○ Traditional methods, like herbs<br>○ Home remedies<br>○ Insert materials into the vagina<br>○ Other<br>○ Do not know<br>○ No response<br>selected(${abt_ways}, filter_list) or (filter_list = 'always')
708. Where do women go for a surgical procedure, like D&C or "cleaning", to remove a pregnancy? Anywhere else?<br>*Do not read options aloud. Select all that apply.*	(selected(${abt_ways},'surgery'))<br>☐ Govt./Municipal Hospital<br>☐ Govt. Dispensary<br>☐ UFWC/UHC/UHP<br>☐ CHC/Rural Hospital/PHC<br>☐ Sub-Centre/ANM<br>☐ Govt. Mobile clinic<br>☐ Camp<br>☐ Anganwadi/ICDS Centre<br>☐ ASHA<br>☐ Other Community-Based Worker<br>☐ NGO or Trust Hospital/Clinic<br>☐ Pvt hospital<br>☐ Pvt. Doctor/Clinic<br>☐ Pvt. Mobile Clinic<br>☐ Vaidya/Hakim/Homeopath (Ayush)

	☐ Traditional Healer<br>☐ Pharmacy/Drugstore<br>☐ Dai (TBA)<br>☐ Shop<br>☐ Friend / parent / relative<br>☐ Other<br>☐ Do not know<br>☐ No response
709. Which of these places is the most common?	count-selected(${abt_surg_where}) > 1<br>○ Govt./Municipal Hospital<br>○ Govt. Dispensary<br>○ UFWC/UHC/UHP<br>○ CHC/Rural Hospital/PHC<br>○ Sub-Centre/ANM<br>○ Govt. Mobile clinic<br>○ Camp<br>○ Anganwadi/ICDS Centre<br>○ ASHA<br>○ Other Community-Based Worker<br>○ NGO or Trust Hospital/Clinic<br>○ Pvt hospital<br>○ Pvt. Doctor/Clinic<br>○ Pvt. Mobile Clinic<br>○ Vaidya/Hakim/Homeopath (Ayush)<br>○ Traditional Healer<br>○ Pharmacy/Drugstore<br>○ Dai (TBA)<br>○ Shop<br>○ Friend / parent / relative<br>○ Other<br>○ Do not know<br>○ No response<br>selected(${abt_surg_where}, filter_list) or (filter_list = 'always')
710. Where do women get the medicines to remove a pregnancy or have an abortion? Anywhere else?	(selected(${abt_ways}, 'pills_abortion')) or (selected(${abt_ways}, 'pills_fever')) or (selected($ …<br>☐ Govt./Municipal Hospital<br>☐ Govt. Dispensary<br>☐ UFWC/UHC/UHP<br>☐ CHC/Rural Hospital/PHC<br>☐ Sub-Centre/ANM<br>☐ Govt. Mobile clinic<br>☐ Camp<br>☐ Anganwadi/ICDS Centre<br>☐ ASHA<br>☐ Other Community-Based Worker<br>☐ NGO or Trust Hospital/Clinic<br>☐ Pvt hospital<br>☐ Pvt. Doctor/Clinic<br>☐ Pvt. Mobile Clinic<br>☐ Vaidya/Hakim/Homeopath (Ayush)<br>☐ Traditional Healer<br>☐ Pharmacy/Drugstore<br>☐ Dai (TBA)<br>☐ Shop<br>☐ Friend / parent / relative<br>☐ Other<br>☐ Do not know<br>☐ No response
	count-selected(${abt_meds_where}) > 1

711. Which of these sources is the most common?	○ Govt./Municipal Hospital<br>○ Govt. Dispensary<br>○ UFWC/UHC/UHP<br>○ CHC/Rural Hospital/PHC<br>○ Sub-Centre/ANM<br>○ Govt. Mobile clinic<br>○ Camp<br>○ Anganwadi/ICDS Centre<br>○ ASHA<br>○ Other Community-Based Worker<br>○ NGO or Trust Hospital/Clinic<br>○ Pvt hospital<br>○ Pvt. Doctor/Clinic<br>○ Pvt. Mobile Clinic<br>○ Vaidya/Hakim/Homeopath (Ayush)<br>○ Traditional Healer<br>○ Pharmacy/Drugstore<br>○ Dai (TBA)<br>○ Shop<br>○ Friend / parent / relative<br>○ Other<br>○ Do not know<br>○ No response<br>selected(${abt_meds_where}, filter_list) or (filter_list = 'always')
712a.i. Now I want to ask some more questions about ${friend1_name}. Has she ever done something to remove a pregnancy when she was pregnant or worried she was pregnant?<br>*Probe to confirm whether the pregnancy removal was successful. If not, select 'no.'*	○ Yes, I am certain<br>○ Yes, I think so<br>○ No<br>○ Do not know<br>○ No response
713a.i. In what year did this last happen?<br>*If indicates happened more than once, specify most recent time.*<br>*Enter 2020 for 'Do not know' or 'No response'.*	(${friend1_abt_yn} = 'yes') or (${friend1_abt_yn} = 'likely')<br>Year: ___________________
714a.i. Women sometimes do many things to stop a pregnancy from continuing. Did ${friend1_name} do more than one thing to try to remove the pregnancy?	(${friend1_abt_yn} = 'yes') or (${friend1_abt_yn} = 'likely')<br>○ Yes, I am certain<br>○ Yes, I think so<br>○ No<br>○ Do not know<br>○ No response
715a.i. What was the first thing she did to try to remove the pregnancy?	(${friend1_abt_mult_yn} = 'yes') or (${friend1_abt_mult_yn} = 'likely')<br>○ Surgical procedure, like D&C or "cleaning"<br>○ Pills called mifepristone or misoprostol, for example "500 pills" or "700 pills"<br>○ Pills you take when you have a fever like antibiotics or anti-malarial medicine, for example Combiflam, Paracetamol, or pills that come in a green pack<br>○ Other pills<br>○ Traditional methods, like herbs<br>○ Home remedies<br>○ Insert materials into the vagina<br>○ Other<br>○ Do not know<br>○ No response
715a.i. What did she do that removed the pregnancy?	(${friend1_abt_mult_yn} = 'no') or (${friend1_abt_mult_yn} = '-88')

	◌ Surgical procedure, like D&C or "cleaning"<br>◌ Pills called mifepristone or misoprostol, for example "500 pills" or "700 pills"<br>◌ Pills you take when you have a fever like antibiotics or anti-malarial medicine, for example Combiflam, Paracetamol, or pills that come in a green pack<br>◌ Other pills<br>◌ Traditional methods, like herbs<br>◌ Home remedies<br>◌ Insert materials into the vagina<br>◌ Other<br>◌ Do not know<br>◌ No response
716a.i. Where did she go for the procedure?	(${friend1_abt_first} = 'surgery') or (${friend1_abt_only} = 'surgery')<br>◌ Govt./Municipal Hospital<br>◌ Govt. Dispensary<br>◌ UFWC/UHC/UHP<br>◌ CHC/Rural Hospital/PHC<br>◌ Sub-Centre/ANM<br>◌ Govt. Mobile clinic<br>◌ Camp<br>◌ Anganwadi/ICDS Centre<br>◌ ASHA<br>◌ Other Community-Based Worker<br>◌ NGO or Trust Hospital/Clinic<br>◌ Pvt hospital<br>◌ Pvt. Doctor/Clinic<br>◌ Pvt. Mobile Clinic<br>◌ Vaidya/Hakim/Homeopath (Ayush)<br>◌ Traditional Healer<br>◌ Pharmacy/Drugstore<br>◌ Dai (TBA)<br>◌ Shop<br>◌ Friend / parent / relative<br>◌ Other<br>◌ Do not know<br>◌ No response
717a.i. Where did she get the medicines?	(${friend1_abt_first} = 'pills_abortion') or (${friend1_abt_only} = 'pills_abortion') or (${friend …<br>◌ Govt./Municipal Hospital<br>◌ Govt. Dispensary<br>◌ UFWC/UHC/UHP<br>◌ CHC/Rural Hospital/PHC<br>◌ Sub-Centre/ANM<br>◌ Govt. Mobile clinic<br>◌ Camp<br>◌ Anganwadi/ICDS Centre<br>◌ ASHA<br>◌ Other Community-Based Worker<br>◌ NGO or Trust Hospital/Clinic<br>◌ Pvt hospital<br>◌ Pvt. Doctor/Clinic<br>◌ Pvt. Mobile Clinic<br>◌ Vaidya/Hakim/Homeopath (Ayush)<br>◌ Traditional Healer<br>◌ Pharmacy/Drugstore

	Dai (TBA)<br>Shop<br>Friend / parent / relative<br>Other<br>Do not know<br>No response
718a.i. What was the last thing she did that ultimately removed the pregnancy?	(${friend1_abt_mult_yn} = 'yes') or (${friend1_abt_mult_yn} = 'likely')<br>Surgical procedure, like D&C or "cleaning"<br>Pills called mifepristone or misoprostol, for example "500 pills" or "700 pills"<br>Pills you take when you have a fever like antibiotics or anti-malarial medicine, for example Combiflam, Paracetamol, or pills that come in a green pack<br>Other pills<br>Traditional methods, like herbs<br>Home remedies<br>Insert materials into the vagina<br>Other<br>Do not know<br>No response
719a.i. Where did she go for the procedure?	${friend1_abt_last} = 'surgery'<br>Govt./Municipal Hospital<br>Govt. Dispensary<br>UFWC/UHC/UHP<br>CHC/Rural Hospital/PHC<br>Sub-Centre/ANM<br>Govt. Mobile clinic<br>Camp<br>Anganwadi/ICDS Centre<br>ASHA<br>Other Community-Based Worker<br>NGO or Trust Hospital/Clinic<br>Pvt hospital<br>Pvt. Doctor/Clinic<br>Pvt. Mobile Clinic<br>Vaidya/Hakim/Homeopath (Ayush)<br>Traditional Healer<br>Pharmacy/Drugstore<br>Dai (TBA)<br>Shop<br>Friend / parent / relative<br>Other<br>Do not know<br>No response
720a.i. Where did she get the medicines?	(${friend1_abt_last} = 'pills_abortion') or (${friend1_abt_last} = 'pills_fever') or (${friend1_ab …<br>Govt./Municipal Hospital<br>Govt. Dispensary<br>UFWC/UHC/UHP<br>CHC/Rural Hospital/PHC<br>Sub-Centre/ANM<br>Govt. Mobile clinic<br>Camp<br>Anganwadi/ICDS Centre<br>ASHA<br>Other Community-Based Worker

	◌ NGO or Trust Hospital/Clinic<br>◌ Pvt hospital<br>◌ Pvt. Doctor/Clinic<br>◌ Pvt. Mobile Clinic<br>◌ Vaidya/Hakim/Homeopath (Ayush)<br>◌ Traditional Healer<br>◌ Pharmacy/Drugstore<br>◌ Dai (TBA)<br>◌ Shop<br>◌ Friend / parent / relative<br>◌ Other<br>◌ Do not know<br>◌ No response
721a.i. Did ${friend1_name} have any issues and go to a health facility for treatment in the process of removing the pregnancy?<br>*If the respondent already reported the friend went to a health facility in the process of removing the pregnancy, we are interested in whether the friend went back to a health facility on a separate occasion to treat complications that she may have experienced.*	(${friend1_abt_yn} = 'yes') or (${friend1_abt_yn} = 'likely')<br>◌ Yes, I am certain<br>◌ Yes, I think so<br>◌ No<br>◌ Do not know<br>◌ No response
712a.ii. Besides this event, has ${friend1_name} ever done something to regulate her period when she was worried she was pregnant?<br>*Probe to confirm whether the period regulation was successful. If not, select 'no.'*	${friend1_abt_yn} = 'yes'<br>◌ Yes, I am certain<br>◌ Yes, I think so<br>◌ No<br>◌ Do not know<br>◌ No response
712a.ii. Has ${friend1_name} ever done something to regulate her period when she was worried she was pregnant?<br>*Probe to confirm whether the period regulation was successful. If not, select 'no.'*	${friend1_abt_yn} != 'yes'<br>◌ Yes, I am certain<br>◌ Yes, I think so<br>◌ No<br>◌ Do not know<br>◌ No response
713a.ii. In what year did this last happen?<br>*If indicates happened more than once, specify most recent time.*<br>*Enter 2020 for 'Do not know' or 'No response'.*	(${friend1_reg_yn} = 'yes') or (${friend1_reg_yn} = 'likely')<br>Year: ________________________
714a.ii. Women sometimes do many things to regulate their period. Did ${friend1_name} do more than one thing to try to regulate her period?	((${friend1_reg_year} > ${friend1_abt_year}) or (${friend1_abt_year} = '')) and ((${friend1_reg_yn …<br>◌ Yes, I am certain<br>◌ Yes, I think so<br>◌ No<br>◌ Do not know<br>◌ No response
715a.ii. What was the first thing she did to try to regulate her period?	((${friend1_reg_year} > ${friend1_abt_year}) or (${friend1_abt_year} = '')) and ((${friend1_reg_mu …<br>◌ Surgical procedure, like D&C or "cleaning"<br>◌ Pills called mifepristone or misoprostol, for example "500 pills" or "700 pills"<br>◌ Pills you take when you have a fever like antibiotics or anti-malarial medicine, for example Combiflam, Paracetamol, or pills that come in a green pack<br>◌ Other pills<br>◌ Traditional methods, like herbs<br>◌ Home remedies<br>◌ Insert materials into the vagina<br>◌ Other

	○ Do not know<br>○ No response
715a.ii. What did she do that regulated her period?	((${friend1_reg_year} > ${friend1_abt_year}) or (${friend1_abt_year} = '')) and ((${friend1_reg_mu …<br>○ Surgical procedure, like D&C or "cleaning"<br>○ Pills called mifepristone or misoprostol, for example "500 pills" or "700 pills"<br>○ Pills you take when you have a fever like antibiotics or anti-malarial medicine, for example Combiflam, Paracetamol, or pills that come in a green pack<br>○ Other pills<br>○ Traditional methods, like herbs<br>○ Home remedies<br>○ Insert materials into the vagina<br>○ Other<br>○ Do not know<br>○ No response
716a.ii. Where did she go for the procedure?	((${friend1_reg_year} > ${friend1_abt_year}) or (${friend1_abt_year} = '')) and ((${friend1_reg_fi …<br>○ Govt./Municipal Hospital<br>○ Govt. Dispensary<br>○ UFWC/UHC/UHP<br>○ CHC/Rural Hospital/PHC<br>○ Sub-Centre/ANM<br>○ Govt. Mobile clinic<br>○ Camp<br>○ Anganwadi/ICDS Centre<br>○ ASHA<br>○ Other Community-Based Worker<br>○ NGO or Trust Hospital/Clinic<br>○ Pvt hospital<br>○ Pvt. Doctor/Clinic<br>○ Pvt. Mobile Clinic<br>○ Vaidya/Hakim/Homeopath (Ayush)<br>○ Traditional Healer<br>○ Pharmacy/Drugstore<br>○ Dai (TBA)<br>○ Shop<br>○ Friend / parent / relative<br>○ Other<br>○ Do not know<br>○ No response
717a.ii. Where did she get the medicines?	((${friend1_reg_year} > ${friend1_abt_year}) or (${friend1_abt_year} = '')) and ((${friend1_reg_fi …<br>○ Govt./Municipal Hospital<br>○ Govt. Dispensary<br>○ UFWC/UHC/UHP<br>○ CHC/Rural Hospital/PHC<br>○ Sub-Centre/ANM<br>○ Govt. Mobile clinic<br>○ Camp<br>○ Anganwadi/ICDS Centre<br>○ ASHA<br>○ Other Community-Based Worker<br>○ NGO or Trust Hospital/Clinic

	○ Pvt hospital<br>○ Pvt. Doctor/Clinic<br>○ Pvt. Mobile Clinic<br>○ Vaidya/Hakim/Homeopath (Ayush)<br>○ Traditional Healer<br>○ Pharmacy/Drugstore<br>○ Dai (TBA)<br>○ Shop<br>○ Friend / parent / relative<br>○ Other<br>○ Do not know<br>○ No response
718a.ii. What was the last thing she did that ultimately caused her period to return?	((${friend1_reg_year} > ${friend1_abt_year}) or (${friend1_abt_year} = '')) and ((${friend1_reg_mu …<br>○ Surgical procedure, like D&C or "cleaning"<br>○ Pills called mifepristone or misoprostol, for example "500 pills" or "700 pills"<br>○ Pills you take when you have a fever like antibiotics or anti-malarial medicine, for example Combiflam, Paracetamol, or pills that come in a green pack<br>○ Other pills<br>○ Traditional methods, like herbs<br>○ Home remedies<br>○ Insert materials into the vagina<br>○ Other<br>○ Do not know<br>○ No response
719a.ii. Where did she go for the procedure?	((${friend1_reg_year} > ${friend1_abt_year}) or (${friend1_abt_year} = '')) and (${friend1_reg_las …<br>○ Govt./Municipal Hospital<br>○ Govt. Dispensary<br>○ UFWC/UHC/UHP<br>○ CHC/Rural Hospital/PHC<br>○ Sub-Centre/ANM<br>○ Govt. Mobile clinic<br>○ Camp<br>○ Anganwadi/ICDS Centre<br>○ ASHA<br>○ Other Community-Based Worker<br>○ NGO or Trust Hospital/Clinic<br>○ Pvt hospital<br>○ Pvt. Doctor/Clinic<br>○ Pvt. Mobile Clinic<br>○ Vaidya/Hakim/Homeopath (Ayush)<br>○ Traditional Healer<br>○ Pharmacy/Drugstore<br>○ Dai (TBA)<br>○ Shop<br>○ Friend / parent / relative<br>○ Other<br>○ Do not know<br>○ No response
720a.ii. Where did she get the medicines?	((${friend1_reg_year} > ${friend1_abt_year}) or (${friend1_abt_year} = '')) and ((${friend1_reg_la …<br>○ Govt./Municipal Hospital<br>○ Govt. Dispensary

	◯ UFWC/UHC/UHP<br>◯ CHC/Rural Hospital/PHC<br>◯ Sub-Centre/ANM<br>◯ Govt. Mobile clinic<br>◯ Camp<br>◯ Anganwadi/ICDS Centre<br>◯ ASHA<br>◯ Other Community-Based Worker<br>◯ NGO or Trust Hospital/Clinic<br>◯ Pvt hospital<br>◯ Pvt. Doctor/Clinic<br>◯ Pvt. Mobile Clinic<br>◯ Vaidya/Hakim/Homeopath (Ayush)<br>◯ Traditional Healer<br>◯ Pharmacy/Drugstore<br>◯ Dai (TBA)<br>◯ Shop<br>◯ Friend / parent / relative<br>◯ Other<br>◯ Do not know<br>◯ No response
721a.ii. Did ${friend1_name} have any issues and go to a health facility for treatment in the process of regulating her period?<br>*If the respondent already reported the friend went to a health facility in the process of regulating her period, we are interested in whether the friend went back to a health facility on a separate occasion to treat complications that she may have experienced.*	((${friend1_reg_year} > ${friend1_abt_year}) or (${friend1_abt_year} = '')) and ((${friend1_reg_yn …<br>◯ Yes, I am certain<br>◯ Yes, I think so<br>◯ No<br>◯ Do not know<br>◯ No response
712b.i. Now I want to ask some more questions about ${friend2_name}. Has she ever done something to remove a pregnancy when she was pregnant or worried she was pregnant?<br>*Probe to confirm whether the pregnancy removal was successful. If not, select 'no.'*	◯ Yes, I am certain<br>◯ Yes, I think so<br>◯ No<br>◯ Do not know<br>◯ No response
713b.i. In what year did this last happen?<br>*If indicates happened more than once, specify most recent time.*<br>*Enter 2020 for 'Do not know' or 'No response'.*	(${friend2_abt_yn} = 'yes') or (${friend2_abt_yn} = 'likely')<br>Year: ________________
714b.i. Women sometimes do many things to stop a pregnancy from continuing. Did ${friend2_name} do more than one thing to try to remove the pregnancy?	(${friend2_abt_yn} = 'yes') or (${friend2_abt_yn} = 'likely')<br>◯ Yes, I am certain<br>◯ Yes, I think so<br>◯ No<br>◯ Do not know<br>◯ No response
715b.i. What was the first thing she did to try to remove the pregnancy?	(${friend2_abt_mult_yn} = 'yes') or (${friend2_abt_mult_yn} = 'likely')<br>◯ Surgical procedure, like D&C or "cleaning"<br>◯ Pills called mifepristone or misoprostol, for example "500 pills" or "700 pills"<br>◯ Pills you take when you have a fever like antibiotics or anti-malarial medicine, for example Combiflam, Paracetamol, or pills that come in a green pack<br>◯ Other pills<br>◯ Traditional methods, like herbs<br>◯ Home remedies<br>◯ Insert materials into the vagina<br>◯ Other<br>◯ Do not know

	○ No response
715b.i. What did she do that removed the pregnancy?	(${friend2_abt_mult_yn} = 'no') or (${friend2_abt_mult_yn} = '-88')<br>○ Surgical procedure, like D&C or "cleaning"<br>○ Pills called mifepristone or misoprostol, for example "500 pills" or "700 pills"<br>○ Pills you take when you have a fever like antibiotics or anti-malarial medicine, for example Combiflam, Paracetamol, or pills that come in a green pack<br>○ Other pills<br>○ Traditional methods, like herbs<br>○ Home remedies<br>○ Insert materials into the vagina<br>○ Other<br>○ Do not know<br>○ No response
716b.i. Where did she go for the procedure?	(${friend2_abt_first} = 'surgery') or (${friend2_abt_only} = 'surgery')<br>○ Govt./Municipal Hospital<br>○ Govt. Dispensary<br>○ UFWC/UHC/UHP<br>○ CHC/Rural Hospital/PHC<br>○ Sub-Centre/ANM<br>○ Govt. Mobile clinic<br>○ Camp<br>○ Anganwadi/ICDS Centre<br>○ ASHA<br>○ Other Community-Based Worker<br>○ NGO or Trust Hospital/Clinic<br>○ Pvt hospital<br>○ Pvt. Doctor/Clinic<br>○ Pvt. Mobile Clinic<br>○ Vaidya/Hakim/Homeopath (Ayush)<br>○ Traditional Healer<br>○ Pharmacy/Drugstore<br>○ Dai (TBA)<br>○ Shop<br>○ Friend / parent / relative<br>○ Other<br>○ Do not know<br>○ No response
717b.i. Where did she get the medicines?	(${friend2_abt_first} = 'pills_abortion') or (${friend2_abt_only} = 'pills_abortion') or (${friend ...<br>○ Govt./Municipal Hospital<br>○ Govt. Dispensary<br>○ UFWC/UHC/UHP<br>○ CHC/Rural Hospital/PHC<br>○ Sub-Centre/ANM<br>○ Govt. Mobile clinic<br>○ Camp<br>○ Anganwadi/ICDS Centre<br>○ ASHA<br>○ Other Community-Based Worker<br>○ NGO or Trust Hospital/Clinic<br>○ Pvt hospital<br>○ Pvt. Doctor/Clinic<br>○ Pvt. Mobile Clinic

	Vaidya/Hakim/Homeopath (Ayush)<br>Traditional Healer<br>Pharmacy/Drugstore<br>Dai (TBA)<br>Shop<br>Friend / parent / relative<br>Other<br>Do not know<br>No response
718b.i. What was the last thing she did that ultimately removed the pregnancy?	(${friend2_abt_mult_yn} = 'yes') or (${friend2_abt_mult_yn} = 'likely')<br>Surgical procedure, like D&C or "cleaning"<br>Pills called mifepristone or misoprostol, for example "500 pills" or "700 pills"<br>Pills you take when you have a fever like antibiotics or anti-malarial medicine, for example Combiflam, Paracetamol, or pills that come in a green pack<br>Other pills<br>Traditional methods, like herbs<br>Home remedies<br>Insert materials into the vagina<br>Other<br>Do not know<br>No response
719b.i. Where did she go for the procedure?	${friend2_abt_last} = 'surgery'<br>Govt./Municipal Hospital<br>Govt. Dispensary<br>UFWC/UHC/UHP<br>CHC/Rural Hospital/PHC<br>Sub-Centre/ANM<br>Govt. Mobile clinic<br>Camp<br>Anganwadi/ICDS Centre<br>ASHA<br>Other Community-Based Worker<br>NGO or Trust Hospital/Clinic<br>Pvt hospital<br>Pvt. Doctor/Clinic<br>Pvt. Mobile Clinic<br>Vaidya/Hakim/Homeopath (Ayush)<br>Traditional Healer<br>Pharmacy/Drugstore<br>Dai (TBA)<br>Shop<br>Friend / parent / relative<br>Other<br>Do not know<br>No response
720b.i. Where did she get the medicines?	(${friend2_abt_last} = 'pills_abortion') or (${friend2_abt_last} = 'pills_fever') or (${friend2_ab …<br>Govt./Municipal Hospital<br>Govt. Dispensary<br>UFWC/UHC/UHP<br>CHC/Rural Hospital/PHC<br>Sub-Centre/ANM<br>Govt. Mobile clinic<br>Camp

	◌ Anganwadi/ICDS Centre<br>◌ ASHA<br>◌ Other Community-Based Worker<br>◌ NGO or Trust Hospital/Clinic<br>◌ Pvt hospital<br>◌ Pvt. Doctor/Clinic<br>◌ Pvt. Mobile Clinic<br>◌ Vaidya/Hakim/Homeopath (Ayush)<br>◌ Traditional Healer<br>◌ Pharmacy/Drugstore<br>◌ Dai (TBA)<br>◌ Shop<br>◌ Friend / parent / relative<br>◌ Other<br>◌ Do not know<br>◌ No response
721b.i. Did ${friend2_name} have any issues and go to a health facility for treatment in the process of removing the pregnancy?<br>*If the respondent already reported the friend went to a health facility in the process of removing the pregnancy, we are interested in whether the friend went back to a health facility on a separate occasion to treat complications that she may have experienced.*	(${friend2_abt_yn} = 'yes') or (${friend2_abt_yn} = 'likely')<br>◌ Yes, I am certain<br>◌ Yes, I think so<br>◌ No<br>◌ Do not know<br>◌ No response
712b.ii. Besides this event, has ${friend2_name} ever done something to regulate her period when she was worried she was pregnant?<br>*Probe to confirm whether the period regulation was successful. If not, select 'no.'*	◌ Yes, I am certain<br>◌ Yes, I think so<br>◌ No<br>◌ Do not know<br>◌ No response
712b.ii. Has ${friend2_name} ever done something to regulate her period when she was worried she was pregnant?<br>*Probe to confirm whether the period regulation was successful. If not, select 'no.'*	◌ Yes, I am certain<br>◌ Yes, I think so<br>◌ No<br>◌ Do not know<br>◌ No response
713b.ii. In what year did this last happen?<br>*If indicates happened more than once, specify most recent time.*<br>*Enter 2020 for 'Do not know' or 'No response'.*	(${friend2_reg_yn} = 'yes') or (${friend2_reg_yn} = 'likely')<br>Year: ______________________
714b.ii. Women sometimes do many things to regulate their period. Did ${friend2_name} do more than one thing to try to regulate her period?	((${friend2_reg_year} > ${friend2_abt_year}) or (${friend2_abt_year} = '')) and ((${friend2_reg_yn …<br>◌ Yes, I am certain<br>◌ Yes, I think so<br>◌ No<br>◌ Do not know<br>◌ No response
715b.ii. What was the first thing she did to try to regulate her period?	((${friend2_reg_year} > ${friend2_abt_year}) or (${friend2_abt_year} = '')) and ((${friend2_reg_mu …<br>◌ Surgical procedure, like D&C or "cleaning"<br>◌ Pills called mifepristone or misoprostol, for example "500 pills" or "700 pills"<br>◌ Pills you take when you have a fever like antibiotics or anti-malarial medicine, for example Combiflam, Paracetamol, or pills that come in a green pack<br>◌ Other pills<br>◌ Traditional methods, like herbs<br>◌ Home remedies<br>◌ Insert materials into the vagina

	○ Other<br>○ Do not know<br>○ No response
715b.ii. What did she do that regulated her period?	((${friend2_reg_year} > ${friend2_abt_year}) or (${friend2_abt_year} = '')) and ((${friend2_reg_mu …<br>○ Surgical procedure, like D&C or "cleaning"<br>○ Pills called mifepristone or misoprostol, for example "500 pills" or "700 pills"<br>○ Pills you take when you have a fever like antibiotics or anti-malarial medicine, for example Combiflam, Paracetamol, or pills that come in a green pack<br>○ Other pills<br>○ Traditional methods, like herbs<br>○ Home remedies<br>○ Insert materials into the vagina<br>○ Other<br>○ Do not know<br>○ No response
716b.ii. Where did she go for the procedure?	((${friend2_reg_year} > ${friend2_abt_year}) or (${friend2_abt_year} = '')) and ((${friend2_reg_fi …<br>○ Govt./Municipal Hospital<br>○ Govt. Dispensary<br>○ UFWC/UHC/UHP<br>○ CHC/Rural Hospital/PHC<br>○ Sub-Centre/ANM<br>○ Govt. Mobile clinic<br>○ Camp<br>○ Anganwadi/ICDS Centre<br>○ ASHA<br>○ Other Community-Based Worker<br>○ NGO or Trust Hospital/Clinic<br>○ Pvt hospital<br>○ Pvt. Doctor/Clinic<br>○ Pvt. Mobile Clinic<br>○ Vaidya/Hakim/Homeopath (Ayush)<br>○ Traditional Healer<br>○ Pharmacy/Drugstore<br>○ Dai (TBA)<br>○ Shop<br>○ Friend / parent / relative<br>○ Other<br>○ Do not know<br>○ No response
717b.ii. Where did she get the medicines?	((${friend2_reg_year} > ${friend2_abt_year}) or (${friend2_abt_year} = '')) and ((${friend2_reg_fi …<br>○ Govt./Municipal Hospital<br>○ Govt. Dispensary<br>○ UFWC/UHC/UHP<br>○ CHC/Rural Hospital/PHC<br>○ Sub-Centre/ANM<br>○ Govt. Mobile clinic<br>○ Camp<br>○ Anganwadi/ICDS Centre<br>○ ASHA<br>○ Other Community-Based Worker<br>○ NGO or Trust Hospital/Clinic

	○ Pvt hospital<br>○ Pvt. Doctor/Clinic<br>○ Pvt. Mobile Clinic<br>○ Vaidya/Hakim/Homeopath (Ayush)<br>○ Traditional Healer<br>○ Pharmacy/Drugstore<br>○ Dai (TBA)<br>○ Shop<br>○ Friend / parent / relative<br>○ Other<br>○ Do not know<br>○ No response
718b.ii. What was the last thing she did that ultimately caused her period to return?	((${friend2_reg_year} > ${friend2_abt_year}) or (${friend2_abt_year} = '')) and ((${friend2_reg_mu …<br>○ Surgical procedure, like D&C or "cleaning"<br>○ Pills called mifepristone or misoprostol, for example "500 pills" or "700 pills"<br>○ Pills you take when you have a fever like antibiotics or anti-malarial medicine, for example Combiflam, Paracetamol, or pills that come in a green pack<br>○ Other pills<br>○ Traditional methods, like herbs<br>○ Home remedies<br>○ Insert materials into the vagina<br>○ Other<br>○ Do not know<br>○ No response
719b.ii. Where did she go for the procedure?	((${friend2_reg_year} > ${friend2_abt_year}) or (${friend2_abt_year} = '')) and (${friend2_reg_las …<br>○ Govt./Municipal Hospital<br>○ Govt. Dispensary<br>○ UFWC/UHC/UHP<br>○ CHC/Rural Hospital/PHC<br>○ Sub-Centre/ANM<br>○ Govt. Mobile clinic<br>○ Camp<br>○ Anganwadi/ICDS Centre<br>○ ASHA<br>○ Other Community-Based Worker<br>○ NGO or Trust Hospital/Clinic<br>○ Pvt hospital<br>○ Pvt. Doctor/Clinic<br>○ Pvt. Mobile Clinic<br>○ Vaidya/Hakim/Homeopath (Ayush)<br>○ Traditional Healer<br>○ Pharmacy/Drugstore<br>○ Dai (TBA)<br>○ Shop<br>○ Friend / parent / relative<br>○ Other<br>○ Do not know<br>○ No response
720b.ii. Where did she get the medicines?	((${friend2_reg_year} > ${friend2_abt_year}) or (${friend2_abt_year} = '')) and ((${friend2_reg_la …<br>○ Govt./Municipal Hospital

	◌ Govt. Dispensary<br>◌ UFWC/UHC/UHP<br>◌ CHC/Rural Hospital/PHC<br>◌ Sub-Centre/ANM<br>◌ Govt. Mobile clinic<br>◌ Camp<br>◌ Anganwadi/ICDS Centre<br>◌ ASHA<br>◌ Other Community-Based Worker<br>◌ NGO or Trust Hospital/Clinic<br>◌ Pvt hospital<br>◌ Pvt. Doctor/Clinic<br>◌ Pvt. Mobile Clinic<br>◌ Vaidya/Hakim/Homeopath (Ayush)<br>◌ Traditional Healer<br>◌ Pharmacy/Drugstore<br>◌ Dai (TBA)<br>◌ Shop<br>◌ Friend / parent / relative<br>◌ Other<br>◌ Do not know<br>◌ No response
721b.ii. Did ${friend2_name} have any issues and go to a health facility for treatment in the process of regulating her period?<br>*If the respondent already reported the friend went to a health facility in the process of regulating her period, we are interested in whether the friend went back to a health facility on a separate occasion to treat complications that she may have experienced.*	((${friend2_reg_year} > ${friend2_abt_year}) or (${friend2_abt_year} = '')) and ((${friend2_reg_yn …<br>◌ Yes, I am certain<br>◌ Yes, I think so<br>◌ No<br>◌ Do not know<br>◌ No response
722a. Now I would like to ask about your own experience. Have you ever done something to remove a pregnancy when you were pregnant or worried you were pregnant?<br>*Probe to confirm whether the pregnancy removal was successful. If not, select 'no.'*	◌ Yes<br>◌ No<br>◌ No response
723a. In what year did this last happen?<br>*If indicates happened more than once, specify most recent time.*<br>*Enter 2020 for 'Do not know' or 'No response'.*	(${self_abt_yn} = 'yes')<br>Year: ____________________
724a. Did you do more than one thing to try to remove the pregnancy?	(${self_abt_yn} = 'yes')<br>◌ Yes<br>◌ No<br>◌ No response
725a. What did you first do?	(${self_abt_mult_yn} = 'yes')<br>◌ Surgical procedure, like D&C or "cleaning"<br>◌ Pills called mifepristone or misoprostol, for example "500 pills" or "700 pills"<br>◌ Pills you take when you have a fever like antibiotics or anti-malarial medicine, for example Combiflam, Paracetamol, or pills that come in a green pack<br>◌ Other pills<br>◌ Traditional methods, like herbs<br>◌ Home remedies<br>◌ Insert materials into the vagina<br>◌ Other<br>◌ Do not know<br>◌ No response
725a. What did you do?	(${self_abt_mult_yn} = 'no')<br>◌ Surgical procedure, like D&C or

	"cleaning"<br>◌ Pills called mifepristone or misoprostol, for example "500 pills" or "700 pills"<br>◌ Pills you take when you have a fever like antibiotics or anti-malarial medicine, for example Combiflam, Paracetamol, or pills that come in a green pack<br>◌ Other pills<br>◌ Traditional methods, like herbs<br>◌ Home remedies<br>◌ Insert materials into the vagina<br>◌ Other<br>◌ Do not know<br>◌ No response
726a. Where did you go for the procedure?	(${self_abt_first} = 'surgery') or (${self_abt_only} = 'surgery')<br>◌ Govt./Municipal Hospital<br>◌ Govt. Dispensary<br>◌ UFWC/UHC/UHP<br>◌ CHC/Rural Hospital/PHC<br>◌ Sub-Centre/ANM<br>◌ Govt. Mobile clinic<br>◌ Camp<br>◌ Anganwadi/ICDS Centre<br>◌ ASHA<br>◌ Other Community-Based Worker<br>◌ NGO or Trust Hospital/Clinic<br>◌ Pvt hospital<br>◌ Pvt. Doctor/Clinic<br>◌ Pvt. Mobile Clinic<br>◌ Vaidya/Hakim/Homeopath (Ayush)<br>◌ Traditional Healer<br>◌ Pharmacy/Drugstore<br>◌ Dai (TBA)<br>◌ Shop<br>◌ Friend / parent / relative<br>◌ Other<br>◌ Do not know<br>◌ No response
727a. Where did you get the medicines?	(${self_abt_first} = 'pills_abortion') or (${self_abt_only} = 'pills_abortion') or (${self_abt_fir …<br>◌ Govt./Municipal Hospital<br>◌ Govt. Dispensary<br>◌ UFWC/UHC/UHP<br>◌ CHC/Rural Hospital/PHC<br>◌ Sub-Centre/ANM<br>◌ Govt. Mobile clinic<br>◌ Camp<br>◌ Anganwadi/ICDS Centre<br>◌ ASHA<br>◌ Other Community-Based Worker<br>◌ NGO or Trust Hospital/Clinic<br>◌ Pvt hospital<br>◌ Pvt. Doctor/Clinic<br>◌ Pvt. Mobile Clinic<br>◌ Vaidya/Hakim/Homeopath (Ayush)<br>◌ Traditional Healer<br>◌ Pharmacy/Drugstore<br>◌ Dai (TBA)

	◌ Shop<br>◌ Friend / parent / relative<br>◌ Other<br>◌ Do not know<br>◌ No response
728a. What was the last thing you did that ultimately removed the pregnancy?	(${self_abt_mult_yn} = 'yes')<br>◌ Surgical procedure, like D&C or "cleaning"<br>◌ Pills called mifepristone or misoprostol, for example "500 pills" or "700 pills"<br>◌ Pills you take when you have a fever like antibiotics or anti-malarial medicine, for example Combiflam, Paracetamol, or pills that come in a green pack<br>◌ Other pills<br>◌ Traditional methods, like herbs<br>◌ Home remedies<br>◌ Insert materials into the vagina<br>◌ Other<br>◌ Do not know<br>◌ No response
729a. Where did you go for the procedure?	${self_abt_last} = 'surgery'<br>◌ Govt./Municipal Hospital<br>◌ Govt. Dispensary<br>◌ UFWC/UHC/UHP<br>◌ CHC/Rural Hospital/PHC<br>◌ Sub-Centre/ANM<br>◌ Govt. Mobile clinic<br>◌ Camp<br>◌ Anganwadi/ICDS Centre<br>◌ ASHA<br>◌ Other Community-Based Worker<br>◌ NGO or Trust Hospital/Clinic<br>◌ Pvt hospital<br>◌ Pvt. Doctor/Clinic<br>◌ Pvt. Mobile Clinic<br>◌ Vaidya/Hakim/Homeopath (Ayush)<br>◌ Traditional Healer<br>◌ Pharmacy/Drugstore<br>◌ Dai (TBA)<br>◌ Shop<br>◌ Friend / parent / relative<br>◌ Other<br>◌ Do not know<br>◌ No response
730a. Where did you get the medicines?	(${self_abt_last} = 'pills_abortion') or (${self_abt_last} = 'pills_fever') or (${self_abt_last} = …<br>◌ Govt./Municipal Hospital<br>◌ Govt. Dispensary<br>◌ UFWC/UHC/UHP<br>◌ CHC/Rural Hospital/PHC<br>◌ Sub-Centre/ANM<br>◌ Govt. Mobile clinic<br>◌ Camp<br>◌ Anganwadi/ICDS Centre<br>◌ ASHA<br>◌ Other Community-Based Worker<br>◌ NGO or Trust Hospital/Clinic<br>◌ Pvt hospital

	◌ Pvt. Doctor/Clinic<br>◌ Pvt. Mobile Clinic<br>◌ Vaidya/Hakim/Homeopath (Ayush)<br>◌ Traditional Healer<br>◌ Pharmacy/Drugstore<br>◌ Dai (TBA)<br>◌ Shop<br>◌ Friend / parent / relative<br>◌ Other<br>◌ Do not know<br>◌ No response
731a. Did you have any issues and go to a health facility for treatment in the process of removing the pregnancy?<br>*If the respondent already reported she went to a health facility in the process of removing the pregnancy, we are interested in whether she went back to a health facility on a separate occasion to treat complications that she may have experienced.*	(${self_abt_yn} = 'yes')<br>◌ Yes<br>◌ No<br>◌ Do not know<br>◌ No response
732a. Did you tell any of the following people about this experience?<br>*Read the answer choices aloud. Select all that apply.*	(${self_abt_yn} = 'yes')<br>☐ Husband/male partner<br>☐ Sister<br>☐ Brother<br>☐ Mother<br>☐ Father<br>☐ Other relative<br>☐ Friend 1: ${friend1_name}<br>☐ Friend 2: ${friend2_name}<br>☐ Other friend<br>☐ Other<br>☐ Do not know<br>☐ No response<br>(${friend1_name} != '' and ${friend1_name} != '-99' and filter_list = 'friend1') or (${friend2_name} != '' and ${friend2_name} != '-99' and filter_list = 'friend2') or (filter_list = 'always')
722b. Besides this event, have you ever done something to regulate your period when you were worried you were pregnant?<br>*Probe to confirm whether the period regulation was successful. If not, select 'no.'*	${self_abt_yn} = 'yes'<br>◌ Yes<br>◌ No<br>◌ No response
722b. Have you ever done something to regulate your period when you were worried you were pregnant?<br>*Probe to confirm whether the period regulation was successful. If not, select 'no.'*	${self_abt_yn} != 'yes'<br>◌ Yes<br>◌ No<br>◌ No response
723b. In what year did this last happen?<br>*If indicates happened more than once, specify most recent time.*<br>*Enter 2020 for 'Do not know' or 'No response'.*	(${self_reg_yn} = 'yes')<br>Year: ___________________
724b. Did you do more than one thing to try to regulate your period?	((${self_reg_year} > ${self_abt_year}) or (${self_abt_year} = '')) and ((${self_reg_yn} = 'yes'))<br>◌ Yes<br>◌ No<br>◌ No response
725b. What did you first do?	((${self_reg_year} > ${self_abt_year}) or (${self_abt_year} = '')) and ((${self_reg_mult_yn} = 'ye …<br>◌ Surgical procedure, like D&C or "cleaning"<br>◌ Pills called mifepristone or misoprostol, for example "500 pills" or "700 pills"<br>◌ Pills you take when you have a fever like antibiotics or anti-malarial medicine, for example Combiflam,

	Paracetamol, or pills that come in a green pack<br>◯ Other pills<br>◯ Traditional methods, like herbs<br>◯ Home remedies<br>◯ Insert materials into the vagina<br>◯ Other<br>◯ Do not know<br>◯ No response
725b. What did you do?	((${self_reg_year} > ${self_abt_year}) or (${self_abt_year} = '')) and ((${self_reg_mult_yn} = 'no …<br>◯ Surgical procedure, like D&C or "cleaning"<br>◯ Pills called mifepristone or misoprostol, for example "500 pills" or "700 pills"<br>◯ Pills you take when you have a fever like antibiotics or anti-malarial medicine, for example Combiflam, Paracetamol, or pills that come in a green pack<br>◯ Other pills<br>◯ Traditional methods, like herbs<br>◯ Home remedies<br>◯ Insert materials into the vagina<br>◯ Other<br>◯ Do not know<br>◯ No response
726b. Where did you go for the procedure?	((${self_reg_year} > ${self_abt_year}) or (${self_abt_year} = '')) and ((${self_reg_first} = 'surg …<br>◯ Govt./Municipal Hospital<br>◯ Govt. Dispensary<br>◯ UFWC/UHC/UHP<br>◯ CHC/Rural Hospital/PHC<br>◯ Sub-Centre/ANM<br>◯ Govt. Mobile clinic<br>◯ Camp<br>◯ Anganwadi/ICDS Centre<br>◯ ASHA<br>◯ Other Community-Based Worker<br>◯ NGO or Trust Hospital/Clinic<br>◯ Pvt hospital<br>◯ Pvt. Doctor/Clinic<br>◯ Pvt. Mobile Clinic<br>◯ Vaidya/Hakim/Homeopath (Ayush)<br>◯ Traditional Healer<br>◯ Pharmacy/Drugstore<br>◯ Dai (TBA)<br>◯ Shop<br>◯ Friend / parent / relative<br>◯ Other<br>◯ Do not know<br>◯ No response
727b. Where did you get the medicines?	((${self_reg_year} > ${self_abt_year}) or (${self_abt_year} = '')) and ((${self_reg_first} = 'pill …<br>◯ Govt./Municipal Hospital<br>◯ Govt. Dispensary<br>◯ UFWC/UHC/UHP<br>◯ CHC/Rural Hospital/PHC

	Sub-Centre/ANM<br>Govt. Mobile clinic<br>Camp<br>Anganwadi/ICDS Centre<br>ASHA<br>Other Community-Based Worker<br>NGO or Trust Hospital/Clinic<br>Pvt hospital<br>Pvt. Doctor/Clinic<br>Pvt. Mobile Clinic<br>Vaidya/Hakim/Homeopath (Ayush)<br>Traditional Healer<br>Pharmacy/Drugstore<br>Dai (TBA)<br>Shop<br>Friend / parent / relative<br>Other<br>Do not know<br>No response
728b. What was the last thing you did that ultimately caused your period to return?	((${self_reg_year} > ${self_abt_year}) or (${self_abt_year} = '')) and ((${self_reg_mult_yn} = 'ye …<br>Surgical procedure, like D&C or "cleaning"<br>Pills called mifepristone or misoprostol, for example "500 pills" or "700 pills"<br>Pills you take when you have a fever like antibiotics or anti-malarial medicine, for example Combiflam, Paracetamol, or pills that come in a green pack<br>Other pills<br>Traditional methods, like herbs<br>Home remedies<br>Insert materials into the vagina<br>Other<br>Do not know<br>No response
729b. Where did you go for the procedure?	((${self_reg_year} > ${self_abt_year}) or (${self_abt_year} = '')) and (${self_reg_last} = 'surger …<br>Govt./Municipal Hospital<br>Govt. Dispensary<br>UFWC/UHC/UHP<br>CHC/Rural Hospital/PHC<br>Sub-Centre/ANM<br>Govt. Mobile clinic<br>Camp<br>Anganwadi/ICDS Centre<br>ASHA<br>Other Community-Based Worker<br>NGO or Trust Hospital/Clinic<br>Pvt hospital<br>Pvt. Doctor/Clinic<br>Pvt. Mobile Clinic<br>Vaidya/Hakim/Homeopath (Ayush)<br>Traditional Healer<br>Pharmacy/Drugstore<br>Dai (TBA)<br>Shop<br>Friend / parent / relative<br>Other

	○ Do not know<br>○ No response
730b. Where did you get the medicines?	((${self_reg_year} > ${self_abt_year}) or (${self_abt_year} = '')) and ((${self_reg_last} = 'pills …<br>○ Govt./Municipal Hospital<br>○ Govt. Dispensary<br>○ UFWC/UHC/UHP<br>○ CHC/Rural Hospital/PHC<br>○ Sub-Centre/ANM<br>○ Govt. Mobile clinic<br>○ Camp<br>○ Anganwadi/ICDS Centre<br>○ ASHA<br>○ Other Community-Based Worker<br>○ NGO or Trust Hospital/Clinic<br>○ Pvt hospital<br>○ Pvt. Doctor/Clinic<br>○ Pvt. Mobile Clinic<br>○ Vaidya/Hakim/Homeopath (Ayush)<br>○ Traditional Healer<br>○ Pharmacy/Drugstore<br>○ Dai (TBA)<br>○ Shop<br>○ Friend / parent / relative<br>○ Other<br>○ Do not know<br>○ No response
731b. Did you have any issues and go to a health facility for treatment in the process of regulating your period?<br>*If the respondent already reported she went to a health facility in the process of regulating her period, we are interested in whether she went back to a health facility on a separate occasion to treat complications that she may have experienced.*	((${self_reg_year} > ${self_abt_year}) or (${self_abt_year} = '')) and ((${self_reg_yn} = 'yes'))<br>○ Yes<br>○ No<br>○ Do not know<br>○ No response
732b. Did you tell any of the following people about this experience?<br>*Read the answer choices aloud. Select all that apply.*	((${self_reg_year} > ${self_abt_year}) or (${self_abt_year} = '')) and ((${self_reg_yn} = 'yes'))<br>☐ Husband/male partner<br>☐ Sister<br>☐ Brother<br>☐ Mother<br>☐ Father<br>☐ Other relative<br>☐ Friend 1: ${friend1_name}<br>☐ Friend 2: ${friend2_name}<br>☐ Other friend<br>☐ Other<br>☐ Do not know<br>☐ No response<br>(${friend1_name} != '' and ${friend1_name} != '-99' and filter_list = 'friend1') or (${friend2_name} != '' and ${friend2_name} != '-99' and filter_list = 'friend2') or (filter_list = 'always')

	${consent_obtained}
Now we want to ask you some general questions about removing a pregnancy. Please provide your responses in the form of: strongly agree, agree, neither agree nor disagree, disagree, strongly disagree.<br>*Check box to confirm scrolled to bottom.*	
Press OK to continue.	○ OK

733. It is okay for a woman to remove a pregnancy if continuing the pregnancy puts her health at risk.	${consent_obtained}<br>Strongly agree<br>Agree<br>Neither agree nor disagree<br>Disagree<br>Strongly disagree<br>No response
734. It is okay for a woman to remove a pregnancy if the pregnancy is a result of rape.	${consent_obtained}<br>Strongly agree<br>Agree<br>Neither agree nor disagree<br>Disagree<br>Strongly disagree<br>No response
735. It is okay for a woman to remove a pregnancy if she cannot afford to raise another child.	${consent_obtained}<br>Strongly agree<br>Agree<br>Neither agree nor disagree<br>Disagree<br>Strongly disagree<br>No response
736. It is okay for a woman to remove a pregnancy if she does not want to have a child.	${consent_obtained}<br>Strongly agree<br>Agree<br>Neither agree nor disagree<br>Disagree<br>Strongly disagree<br>No response
737. A woman who removes a pregnancy brings shame to her family.	${consent_obtained}<br>Strongly agree<br>Agree<br>Neither agree nor disagree<br>Disagree<br>Strongly disagree<br>No response
738. A woman who removes a pregnancy should not tell anyone.	${consent_obtained}<br>Strongly agree<br>Agree<br>Neither agree nor disagree<br>Disagree<br>Strongly disagree<br>No response
739. Are there circumstances when removing a pregnancy is legal in India?	${consent_obtained}<br>Yes<br>No<br>Do not know<br>No response

Follow-up Consent

FLW_801. Thank you for the time you have kindly granted us.<br>We are very interested in hearing more about your pregnancy removal or period regulation experience, including your decision making around what method to use and where to go for services, among other things. Would you be willing to meet with another woman from the project at a later date to talk about this experience?	(${self_abt_yn} = 'yes') or (${self_reg_yn} = 'yes')<br>Yes<br>No<br>No response
FLW_802. Do you own a phone?	${flw_willing} = 'yes'<br>Yes<br>No<br>No response
FLW_803. Can I have your primary phone number in case we	${flw_number_yn} = 'yes'

would like to follow up with you in the future?<br>*Enter an 10-digit number without the country code. Do not include spaces or dashes. Enter 0 for no response.*	--
FLW_804. Can you repeat the number again?<br>*Enter an 10-digit number without the country code. Do not include spaces or dashes. Enter 0 for no response.*	(${flw_number_typed} != '') and (${flw_number_typed} != '0')<br>--
Thank the respondent for her time.<br>*The respondent is finished, but there are still more questions for you to complete outside the home.*	${available} = 'yes'

Location and Questionnaire result

095. Location<br>*Take a GPS point near the entrance to the household. Record location when the accuracy is smaller than 6 m.*	Always
096. How many times have you visited this household to interview this female respondent?	Always<br>○ First time<br>○ Second time<br>○ Third time
097. In what language was this interview conducted?	009a = 1<br>○ English<br>○ Hindi<br>○ Other
099. Questionnaire Result<br>*Record the result of the questionnaire.*	Always<br>○ Completed<br>○ Not at home<br>○ Postponed<br>○ Refused<br>○ Partly completed<br>○ Incapacitated
