# Supplementary material for: Measurement of abortion safety using community-based surveys: Findings from three countries
Source: PLoS One. 2019 Nov 7;14(11):e0223146. doi: 10.1371/journal.pone.0223146 (PMC6837422; doi:10.1371/journal.pone.0223146)
Supplement: S9 Doc — (PDF) [file pone.0223146.s009.pdf]

RJR4-Female-Questionnaire-v10

001a. क्या आप ठीक घर केसामने है?<br>EA: ${EA} ढांचा #: ${structure} परिवार #: ${household}	not(${unlinked})<br>◯ हाँ<br>◯ नहीं
002. नीचे अपनी आई.डी. दर्ज करे<br>*अपनी आई डी दर्ज करें*	(${your_name_check} = 'no')<br>____________________
003b. सही दिनांक और समय रिकॉर्ड करें।	${system_date_check} = 'no' or today() < date("2018-01-01") or today() > date("2019-01-01")<br>Day: ________________<br>Month: ________________<br>Year: ________________

004a. निम्न जानकारी घर-परिवार प्रश्नावली से है। यह सुनिश्चित करने के लिए कृपया पुनः देख लें कि आप सही उत्तरदाता से साक्षात्कार कर रहे हैं	not(${unlinked})
जिला: ${level1_unlinked}	____________________
तहसील / तालुक: ${level2_unlinked}	____________________
शहर/ कस्बा/ गाँव: ${level3_unlinked}	____________________
गणना क्षेत्र ${EA_unlinked}	____________________
ढांचा नंबर: ${structure_unlinked}	____________________
घर परिवार संख्या ${household_unlinked}	____________________
004b. क्या ऊपर दी गई सूचना सही है?	◯ हाँ<br>◯ नहीं

005. सुनिश्चित करें: मैं ${firstname} का साक्षात्कार करने का प्रयास कर रही हुं। क्या यह सही है?<br>*यदि यहाँ नाम की गलत वर्तनी (स्पेलिंग) हो तो, हाँ का चुनाव कीजिये और प्रश्न "011" में नाम को सही करें।*<br>*यदि सम्बन्धित व्यक्ति नहीं है तो आपकेपास दो विकल्प है:*<br>*(1) बाहर निकलें और इस फार्म में किए गए परिवर्तनों को नज़रंदाज़ करें। सही फार्म खोलें।*<br>*या*<br>*(2) जिस व्यक्ति का नाम ऊपर प्रदर्शित (डिस्प्ले) हुआ था उसे ही ढूंढें और उसका साक्षात्कार करें।*	not(${unlinked})<br>◯ हाँ<br>◯ नहीं
006. क्या उत्तरदाता आज साक्षात्कार केलिए मौजूद और उपलब्ध है?	◯ हाँ<br>◯ नहीं
007. आप उत्तरदाता से कितनी अच्छी तरह से परिचित हैं?	${available} = 'yes' and (not(${unlinked}) or ${proceed_with_unlinked})<br>◯ बहुत अच्छी तरह से परिचित<br>◯ अच्छी तरह से परिचित<br>◯ अच्छी तरह से परिचित नहीं<br>◯ परिचित नहीं
008. क्या यह उत्तरदाता PMA2020 सर्वेक्षण में पहले भी भाग ले चुकी हैं?	(${available} = 'yes') and (not(${unlinked}) or ${proceed_with_unlinked})<br>◯ हाँ<br>◯ नहीं<br>◯ पता नहीं<br>◯ कोई जवाब नहीं
सूचित सहमति<br>*इस महिला प्रश्नावली से सम्बन्धित 15 - 49 आयु केबीच की महिला को ढूँढिए। गोपनीयता बरती जाए कि इस साक्षात्कार को कोई अन्य न सुने। निम्नलिखित अभिवादन पढ़ें।*	(${available} = 'yes') and (not(${unlinked}) or ${proceed_with_unlinked})
नमस्कार! मेरा नाम ______________ है और मै भारतीय स्वास्थ्य प्रबंध	(${available} = 'yes')

शोध विश्वविद्यालय और उनकेस्थानीय सहयोगी स्वयंसेवी संगठन केलिए कार्य कर रही हूँ।मैं इसी/पास केगाँव की हूँ।हम एक स्थानीय सर्वे कर रहे है जिसकेअंतर्गत महिलाओ केप्रजनन स्वास्थ्य से सम्बन्धित विभिन्न मुद्दों पर पूछताछ करेंगे जो की परिवार नियोजन और स्वास्थ्य सेवाओं केउन अंतरालों को पहचानने में मदद करेगा जो की उत्पादों की निरंतर उपलब्धता, जानकारी, सेवाओं, और गुणवत्ता को प्रभावित करते है । यह सर्वे पूरे राजस्थान में किया जा रहा है। प्रत्येक गणना क्षेत्र से 35 घरों को लिया गया है और इसी कड़ी में आपका घर चुना गया है।आपकी सहभागिता हमारे लिए बहुत ज़रूरी है लेकिन यह आपकी इच्छा पर निर्भर है, अगर आप मना भी करती है तो इसका कोई दुष्प्रभाव आप पर नहीं पड़ेगा। आपकेद्वारा दी जाने वाली जानकारी सरकार को बेहतर स्वास्थ्य योजना बनाने में मदद करेगी। इसमें यूँ तो कोई खतरा नहीं है लेकिन व्यक्तिगत व सवेदनशील विषयों पर जानकारी साझा करने पर प्रतिभागियों को कुछ तनाव हो सकता है। इसका कोई सीधा लाभ भी आपको नहीं मिलेगा परन्तु हम यह मानते है कि यह अध्ययन हमें व हमारे जैसे कई समूहों को ऐसे कार्यक्रम विकसित करने में मदद करेगा जो कि राजस्थान व भारत में रह रहे समुदायों केलोगों का जीवन उन्नत बना सकेगा। अतः हमें उम्मीद है कि आप इस सर्वेक्षण में भाग लेंगी। जो कुछ भी जानकारी आप प्रदान करती हैं वह पूरी तरह से गोपनीय रखी जाएगी और हमारी सर्वेक्षण टीम केसदस्यों केअलावा अन्य किसी को नहीं दिखायी जायेगी। आकड़ो के विश्लेषण में हम आपकेनाम का उपयोग नहीं करेंगे। आपकेजवाबों से बने आकड़ो केडाटा बेस को पासवर्ड से सुरक्षित किये कम्प्यूटर और सर्वर पर ही रखा जायेगा। इस सर्वेक्षण में भाग लेना स्वैच्छिक है, और आप किसी भी सवाल का जवाब नहीं देना चाहती हैं तो बस मुझे बता दीजिये और मैं अगले प्रश्न पर चली जाउंगी या आप किसी भी समय साक्षात्कार रोक सकती है। सामान्यतः इस सर्वेक्षण को पूरा करने में 30 से 40 मिनिट लगते है। एक अनुसन्धान में भागीदार होने केनाते यदि इससे सम्बंधित कुछ प्रश्न है तो आप इसी समय मुझसे पूछ सकते हैं या भारतीय स्वास्थ्य प्रबंध शोध विश्वविद्यालय जयपुर राजस्थान केडॉ. अनूप खन्ना से 91.141.3924738 पर संपर्ककर सकते है।	
009a. उत्तरदाता को सहमति पत्र की छायाप्रति देवें और विस्तार से समझाएं। फिर पूछें क्या मैं आपका साक्षात्कार शुरू कर सकती/सकता हूँ?	${available} = 'yes'<br>◯ हाँ<br>◯ नहीं

	(${begin_interview}='yes')
009b. उत्तरदाता केहस्ताक्षर<br>*कृपया उत्तरदाता से अपनी भागीदरी की सहमति केलिए हस्ताक्षर या खाने को चेक करने को कहें*	______________________

010. साक्षातकर्ता की आई डी :<br>कृपया अपनी आईडी सहमति प्रक्रिया केगवाह केरूप में दर्ज करें। आपने पहले भी परिवार प्रश्नावली केप्रश्न संख्या "${name_typed}." दर्ज किया है।	${consent_obtained} and (${your_name_check} = 'no')<br>______________________

भाग 1 - उत्तरदाता की पृष्ठभूमि, वैवाहिक स्थिति, घर-परिवार की विशेषताएं

अब मैं आपकी पृष्ठभूमि और सामाजिक आर्थिक स्थिति केबारे में पूछना चाहती हूं।

	${consent_obtained}
101. आप किस महीने और साल में पैदा हुईं थी?<br>घर-परिवार प्रश्नावली में उम्र ${age} वर्ष है	not(${unlinked})
101. आप किस महीने और साल में पैदा हुईं थी?	${unlinked}
माह:	◯ पता नहीं<br>◯ जनवरी<br>◯ फरवरी<br>◯ मार्च<br>◯ अप्रैल<br>◯ मई<br>◯ जून<br>◯ जुलाई<br>◯ अगस्त<br>◯ सितम्बर<br>◯ अक्टूबर<br>◯ नवम्बर<br>◯ दिसम्बर
वर्ष:	Year: ______________

102. अपने पिछले जन्मदिन पर आप कितने वर्ष की थीं?	${consent_obtained}<br>------------------------------------
103. आपने उच्चतम शिक्षा कहाँ तक प्राप्त की है?<br>*केवल ओपचारिक शिक्षा ही दर्ज करें बाइबल,कुरान या लघु-अवधि कोर्स को दर्ज न करें*	${consent_obtained}<br>○ कोई शिक्षा प्राप्त नहीं की<br>○ प्राथमिक<br>○ माध्यमिक<br>○ उच्च माध्यमिक<br>○ स्नातक या उससे ज्यादा<br>○ कोई जवाब नहीं
AH101a. क्या आप अब भी स्कूल/कॉलेज जा रही हैं?	(${school} = 'primary') or (${school} = 'secondary') or (${school} = 'higher') or (${school} = 'po …<br>○ हाँ<br>○ नहीं<br>○ कोई जवाब नहीं
AH101b. क्या आप आगे पढाई करेंगी?	${enrolled} = 'no'<br>○ हाँ<br>○ नहीं<br>○ पता नहीं<br>○ कोई जवाब नहीं
AH102. आपने किस उम्र में स्कूल जाना छोड़ा?<br>*कोई जवाब नही केलिए -99 दर्ज करें*	${enrolled} = 'no'<br>------------------------------------
104. क्या आप वर्तमान में विवाहित है या शादी केरूप में एक पुरुष केसाथ रह रही हैं?<br>*गहराई से जांचे:यदी उत्तरदाता नहीं में उत्तर दे तो सुनिश्चित करें की वह अलग तलाकशुदा या विधवा तो नहीं*	${consent_obtained}<br>○ हाँ, वर्तमान में विवाहित हूं<br>○ विवाहित, परन्तु गौना नहीं हुआ<br>○ हाँ, एक आदमी केसाथ रहती हूँ<br>○ वर्तमान में तलाकशुदा / अलग<br>○ विधवा<br>○ नहीं, कभी साथ नहीं रही<br>○ कोई जवाब नहीं
105. क्या एक या एक से अधिक बार आपकी शादी हुई है अथवा किसी पुरुष केसाथ रही हैं?	${consent_obtained} and ${marital_status} != 'never_married'<br>○ एक बार<br>○ एक से अधिक बार<br>○ कोई जवाब नहीं

	(${marriage_history} = 'more_than_once')
106a. किस महीने और साल में आपने अपने पहले पति / साथी केसाथ रहना शुरू किया?<br>*'कोई जवाब नहीं' केलिए माह में 'पता नहीं' और वर्ष में '2020' चुनें*	
माह:	○ पता नहीं<br>○ जनवरी<br>○ फरवरी<br>○ मार्च<br>○ अप्रैल<br>○ मई<br>○ जून<br>○ जुलाई<br>○ अगस्त<br>○ सितम्बर<br>○ अक्टूबर<br>○ नवम्बर<br>○ दिसम्बर
वर्ष:	Year: ------------------------------

106b. जांचे:प्रश्न 106a में दिए गए उत्तर केअनुसार,उत्तरदाता अपने प्रथम विवाह केसमय 15 वर्ष या 15 वर्ष से कम थी<br>क्या आपने 106a सही से दर्ज किया?	${young_marriage_first}<br>◯ हाँ<br>◯ नहीं

	(${marriage_history} = 'once') or (${marriage_history} = 'more_than_once')
107a. अब मैं आपसे पूछना चाहूंगी कि आपने अपने वर्तमान पति / साथी के साथ कब से रहना शुरु किया। कौन से महीने और साल से?<br>*'कोई जवाब नहीं' केलिए माह में 'पता नहीं' और वर्ष में '2020' चुनें*	
माह:	◯ पता नहीं<br>◯ जनवरी<br>◯ फरवरी<br>◯ मार्च<br>◯ अप्रैल<br>◯ मई<br>◯ जून<br>◯ जुलाई<br>◯ अगस्त<br>◯ सितम्बर<br>◯ अक्टूबर<br>◯ नवम्बर<br>◯ दिसम्बर
वर्ष:	Year: ______________________________

107b. जांचें- 107a में आपकेद्वारा दर्ज उत्तर केअनुसार उत्तरदाता अपनी वर्तमान/हाल ही में हुई शादी केसमय 15 वर्ष या उससे कम की थी।<br>क्या आपने 107a ठीक दर्ज किया है?	${young_marriage_recent}<br>◯ हाँ<br>◯ नहीं
108. क्या आपकेपति की अन्य पत्नियाँ हैं या वे विवाहितों की तरह अन्य महिलाओं केसाथ रहते हैं ?	((${marital_status} = 'currently_married') or (${marital_status} = 'currently_living_with_man'))<br>◯ हाँ<br>◯ नहीं<br>◯ पता नहीं<br>◯ कोई जवाब नहीं

भाग 2 - प्रजनन, गर्भावस्था और जननक्षमता प्राथमिकताएं

अब मैं आपसे उन बच्चों केबारे में पूछना चाहूंगी जिन्हें आपने अपने जीवनकाल में जन्म दिया है।

200. क्याआपने कभी किसी बच्चे को जन्म दिया है?	${consent_obtained}<br>◯ हाँ<br>◯ नहीं<br>◯ कोई जवाब नहीं
201. आपने कितनी बार जन्म दिया है?<br>*कोई जवाब नही केलिए -99 दर्ज करें*	${ever_birth} = 'yes'<br>____________________________________

	(${birth_events} > 1)
205. आपने पहला जन्म कब दिया था?<br>*कृपया पहले जीवित जन्म की तारीख रिकॉर्ड करें। तारीख जानने केलिए यदि आवश्यक हो तो यादगार घटनाओं से आगे या पीछे की गणना की जानी चाहिए।*<br>*'कोई जवाब नहीं' केलिए माह में 'पता नहीं' और वर्ष में '2020' चुनें*	
माह:	◯ पता नहीं<br>◯ जनवरी<br>◯ फरवरी<br>◯ मार्च<br>◯ अप्रैल<br>◯ मई<br>◯ जून<br>◯ जुलाई

	◯ अगस्त<br>◯ सितम्बर<br>◯ अक्टूबर<br>◯ नवम्बर<br>◯ दिसम्बर
वर्ष:	Year: ______________________________

	(${birth_events} > 0)
206. अभी हाल ही में आपने कब जन्म दिया?<br>*'कोई जवाब नहीं' केलिए माह में 'पता नहीं' और वर्ष में '2020' चुनें*	
माह:	◯ पता नहीं<br>◯ जनवरी<br>◯ फरवरी<br>◯ मार्च<br>◯ अप्रैल<br>◯ मई<br>◯ जून<br>◯ जुलाई<br>◯ अगस्त<br>◯ सितम्बर<br>◯ अक्टूबर<br>◯ नवम्बर<br>◯ दिसम्बर
वर्ष:	Year: ______________________________

210a. क्या आप अभी/वर्तमान में गर्भवती हैं ?	${consent_obtained}<br>◯ हाँ<br>◯ नहीं<br>◯ अस्पष्ट<br>◯ कोई जवाब नहीं

	${pregnant} = 'yes'
210b. आप कितने महीने से गर्भवती हैं?	
हाल में दिया गया जन्म: ${rec_birth_date} को हुआ था	${recent_birth} != ''
#####<br>*दर्ज करें कितने माह पूरे हो चुकेहैं. अगर कोई जवाब नहीं, -99 दर्ज करें, अगर पता नहीं, -88 दर्ज करें*	______________________________

209. आपकी पिछली माहवारी शुरू कब हुई?<br>*यदि आप दिन, सप्ताह, माह या वर्ष चुनते हैं तो आपको अगली सक्रीन पर X के स्थान पर एक संख्या दर्ज करनी होगी। आज केलिए 0 दिन दर्ज करें न कि 0 सप्ताह/माह/साल।*	${consent_obtained}<br>◯ X दिनों पहले<br>◯ X सप्ताहों पहले<br>◯ X महीनों पहले<br>◯ X वर्षों पहले<br>◯ मासिक धर्म बंद / गर्भाशय आपरेशन से निकाल लिया गया है<br>◯ पिछले जन्म से पूर्व<br>◯ कभी माहवारी नहीं हुई<br>◯ कोई जवाब नहीं
209a. दर्ज करें ${menstrual_period_lab}<br>*आज केलिए 0 दिन दर्ज करें ना कि 0 सप्ताह / माह / वर्ष।*	(${menstrual_period} = 'days') or (${menstrual_period} = 'weeks') or (${menstrual_period} = 'month …<br>______________________________

	${ever_birth} = 'yes' or ${pregnant} = 'yes'
213a. अब मैं आपसे पिछले शिशु केजन्म केबारे में कुछ प्रश्न पूछना चाहती हूं।	(${ever_birth} = 'yes') and (${pregnant} != 'yes')
213b. अब मैं आपकी वर्तमान गर्भावस्था केबारे में कुछ सवाल पूछना चाहती हूं।	(${pregnant} = 'yes')

जिस समय आप गर्भवती हुईं, क्या आप गर्भवती होना चाहती थीं, या आप कुछ समय बाद तक प्रतीक्षा करना चाहती थीं या आप अधिक बच्चे को जन्म देना नहीं चाहती थीं ?	(${birth_events} > 1 and ${pregnant} != 'yes') or (${ever_birth} = 'yes' and ${pregnant} = 'yes')
जिस समय आप गर्भवती हुईं, क्या आप गर्भवती होना चाहती थीं, या आप कुछ समय बाद तक प्रतीक्षा करना चाहती थीं या आप कोई बच्चे को जन्म देना नहीं चाहती थीं ?	(${birth_events} = 1 and ${pregnant} != 'yes') or ((${ever_birth} = 'no') and (${pregnant} = 'yes'))
#####	○ तभी<br>○ बाद में<br>○ बिलकुल भी नहीं<br>○ कोई जवाब नहीं

अब मैं भविष्य केबारे में कुछ सवाल पूछूंगी।	${consent_obtained}
211a. क्या आप कोई बच्चा/बच्चे चाहेंगी या आप कोई और बच्चा/बच्चे नहीं चाहेंगी ?	${ever_birth} = 'no' and ${pregnant} != 'yes' and ${consent_obtained}<br>○ बच्चा चाहती है<br>○ कोई बच्चा नहीं<br>○ वह कहती हैं गर्भवती नहीं हो सकतीं<br>○ अनिश्चित/ जानते नहीं<br>○ कोई जवाब नहीं
211a. क्या आप और बच्चे चाहेगीं या आप कोई और बच्चा/बच्चे नहीं चाहेंगी?	${ever_birth} = 'yes' and ${pregnant} != 'yes'<br>○ और बच्चे चाहेंगी<br>○ और बच्चा नहीं चाहेंगी<br>○ वह कहती हैं गर्भवती नहीं हो सकतीं<br>○ अनिश्चित/ जानते नहीं<br>○ कोई जवाब नहीं
211b. अभी होनेवाले बच्चे केबाद आप और बच्चा चाहेगीं, या क्या आप चाहेगीं और अधिक बच्चे न हों?	${pregnant} = 'yes'<br>○ और बच्चे चाहेंगी<br>○ और बच्चा नहीं चाहेंगी<br>○ वह कहती हैं गर्भवती नहीं हो सकतीं<br>○ अनिश्चित/ जानते नहीं<br>○ कोई जवाब नहीं
212a. आप बच्चे केलिए कितनी प्रतीक्षा करना चाहती हैं?<br>*अगर आप महीनों या वर्षों का चयन करते हैंए तो अगली स्क्रीन पर ग् केजगह एक नंबर दर्ज करें।*<br>*अगर 36 महीनों से अधिक होंए श्सालश् का चयन करें।*<br>*जाँच लें कि आपने महीनों/वर्षों की उचित संख्या डाली हैं।*	${more_children_none} = 'have_child'<br>○ X महीना<br>○ X वर्ष<br>○ जल्द ही / अभी<br>○ वह कहती हैं गर्भवती नहीं हो सकतीं<br>○ अन्य<br>○ अनिश्चित/जानती नहीं<br>○ कोई जवाब नहीं
212b. आप अभी होने वाले व भविष्य में होने वाले बच्चे केबीच कितने समय का अन्तराल चाहती हैं?<br>*अगर आप महीनों या वर्षों का चयन करते हैंए तो अगली स्क्रीन पर ग् केजगह एक नंबर दर्ज करें।*<br>*अगर 36 महीनों से अधिक होंए श्सालश् का चयन करें।*<br>*जाँच लें कि आपने महीनों/वर्षों की उचित संख्या डाली हैं।*	${more_children_pregnant} = 'have_child'<br>○ X महीना<br>○ X वर्ष<br>○ जल्द ही / अभी<br>○ वह कहती हैं गर्भवती नहीं हो सकतीं<br>○ अन्य<br>○ अनिश्चित/जानती नहीं<br>○ कोई जवाब नहीं
212c. ${waitchild} की संख्या दर्ज करें कि आप कितना समय प्रतीक्षा करना चाहते हैं:	${wait_birth_none} = 'months' or ${wait_birth_some} = 'months' or ${wait_birth_pregnant} = 'mont …<br>------------------------------------

भाग 3 - गर्भनिरोध:

अब मैं आपसे परिवार नियोजन केबारे में बात करना चाहती हूँ- ऐसे विभिन्न उपाय/विधियाँ जिनकेप्रयोग से गर्भधारण को टाल/देरी कर सकते हैं।

301a. क्या आपने कभी महिला नसबंदी केबारे में सुना है?<br>विवरण: यह ओपरेशन महिला करवा सकती है जिससे और आगे बच्चे पैदा करने से रोका जा सकता है।	${consent_obtained}<br>○ हाँ<br>○ नहीं<br>○ कोई जवाब नहीं
301b. क्या आपने कभी पुरुष नसबंदी केबारे में सुना है?<br>विवरण: यह ओपरेशन पुरूष करवा सकते है, जिससे और आगे बच्चे पैदा करने को रोका जा सकता है।	${consent_obtained}<br>○ हाँ<br>○ नहीं<br>○ कोई जवाब नहीं
301c. क्या आपने कभी गर्भनिरोधक इम्प्लांट लगवाने केबारे में सुना है?<br>विवरण: महिलायें डॉक्टर या नर्स से अपनी ऊपरी भुजा में एक या कई छोटे छड़ रख सकतें है जो एक या अधिक वर्षों केलिए गर्भ धारण को रोक सकतें है।<br>[implant_150x300.png]	${consent_obtained}<br>○ हाँ<br>○ नहीं<br>○ कोई जवाब नहीं
301d. क्या आपने कभी आईयूडी/ पी पी आई यू डी केबारे में सुना है?<br>विवरण: महिलाएं डॉक्टर या नर्स की मदद से गर्भाशय केअंदर T केआकार की कॉपर धातु की छड़ रख सकती है| [IUD_150x300.png]	${consent_obtained}<br>○ हाँ<br>○ नहीं<br>○ कोई जवाब नहीं
301e. क्या आपने कभी गर्भनिरोधक इंजेक्शन (इन्जेक्टिबल्स) केबारे में सुना है?<br>विवरण: महिलाएं स्वास्थ्य सेवा प्रदाता द्वारा एक इंजेक्शन लगवा सकतीं है जो एक या अधिक महीनों केलिए गर्भ धारण को रोक सकता है।<br>[sayana_depo_150x300.jpg]	${consent_obtained}<br>○ हाँ<br>○ नहीं<br>○ कोई जवाब नहीं
301f. क्या आपने कभी (जन्म नियंत्रण) गोली केबारे में सुना है?<br>विवरण: महिलाएं गर्भवती होने से बचने केलिए हर दिन एक गोली ले सकती हैं।<br>[pill_150x300.png]	${consent_obtained}<br>○ हाँ<br>○ नहीं<br>○ कोई जवाब नहीं
301g. क्या आपने कभी आपातकालीन गर्भनिरोधक केबारे में सुना है?<br>पूछें: असुरक्षित संभोग केबाद गर्भावस्था को रोकने केलिए महिलाऐं एक आपातकालीन उपाय केरूप में ये विशेष गोलियाँ 3-5 दिनों केभीतर किसी भी समय ले सकती हैं।	${consent_obtained}<br>○ हाँ<br>○ नहीं<br>○ कोई जवाब नहीं
301h. क्या आपने कभी पुरुष कंडोम/निरोध केबारे में सुना है?<br>विवरण: पुरुष संभोग से पहले अपने लिंग पर एक पतली झिल्ली चढ़ा सकते हैं।<br>[male_condom_150x300.png]	${consent_obtained}<br>○ हाँ<br>○ नहीं<br>○ कोई जवाब नहीं
301i. क्या आपने कभी महिला कंडोम केबारे में सुना है?<br>विवरण: महिलाऐं संभोग से पहले उनकी योनी में एक झिल्ली डाल सकती हैं।<br>[female_condom_150x300.png]	${consent_obtained}<br>○ हाँ<br>○ नहीं<br>○ कोई जवाब नहीं
301l. क्या आपने कभी मानक दिवस विधि या साईकिल बीड्स केबारे में सुना है ?<br>विवरण: एक औरत कौन से दिनों में गर्भवती हो सकती है, यह पता करने के लिए एक रंगीन मोतियों की माला का उपयोग किया जा सकता है। जिन दिनों में वह गर्भधारण कर सकती है उन दिनों में संभोग केदौरान कंडोम का इस्तेमाल करें या संभोग ना करें।<br>[SDM-beads_only.png]	${consent_obtained}<br>○ हाँ<br>○ नहीं<br>○ कोई जवाब नहीं
301m. क्या आपने कभी स्तनपान अन्तराल विधि (LAM) केबारे में सुना है?	${consent_obtained}<br>○ हाँ<br>○ नहीं<br>○ कोई जवाब नहीं
301n. क्या आपने कभी लय विधि (रिदम मेथड) केबारे में सुना है?<br>विवरण:जिन दिनों में गर्भ धारण हो सकता है उनमें संभोग नहीं करकेमहिला गर्भधारण से बच सकती है।	${consent_obtained}<br>○ हाँ<br>○ नहीं<br>○ कोई जवाब नहीं
301o. क्या आपने कभी बाह्य स्खलन (विथड्रावल) विधि केबारे में सुना है ?<br>विवरण: पुरुष सावधान रह सकते हैं और चरमोत्कर्ष केसमय बाहर निकाल सकते हैं	${consent_obtained}<br>○ हाँ<br>○ नहीं<br>○ कोई जवाब नहीं

301p. क्या आपने कभी किन्हीं अन्य विधियों केबारे में सुना है, महिला या पुरूष जिनका उपयोग करकेगर्भधारण टाल सकते हैं?	${consent_obtained}<br>○ हाँ<br>○ नहीं<br>○ कोई जवाब नहीं
302a. क्या आप या आपकेसाथी वर्तमान में गर्भवती होने से टालने केलिए या देरी करने केलिए कुछ कर रहे हैं या किसी भी विधि का उपयोग कर रहे हैं ?	${pregnant} != 'yes' and ${consent_obtained}<br>○ हाँ<br>○ नहीं<br>○ कोई जवाब नहीं

	${current_user} = 'yes'
302b. आप कौन सी विधि या विधियों का उपयोग कर रहे हैं?<br>पूछें: कोई और?<br>*उल्लिखित सभी तरीकों का चयन करें। सभी विकल्प देखने केलिए नीचे स्क्रॉल करें।*	☐ महिला नसबंदी<br>☐ पुरुष नसबंदी<br>☐ छड़(इम्प्लांट)<br>☐ आईयूडी / पिपिआईयूडी<br>☐ इंजेक्शन<br>☐ गोली<br>☐ आपातकालीन गर्भनिरोधक<br>☐ पुरुष कंडोम / निरोध<br>☐ महिला कंडोम<br>☐ मानकदिन/ साइकिल बीड्स<br>☐ लैम<br>☐ रिदम मेथड<br>☐ बाह्य स्खलन(विद्ड्रावल)<br>☐ अन्य पारंपरिक तरीके<br>☐ कोई जवाब नहीं

LCL_301. क्या आपने कभी नसबंदी करवायी है?<br>*यदि हाँ, तो पीछे जाकर इस महिला को वर्तमान उपभोक्ता केरूप में दर्ज करें और वर्तमान विधि में महिला नसबंदी का चयन करें*	(${consent_obtained}) and (${pregnant} != 'yes') and ((${current_user} != 'yes') or not(selected($ …<br>○ हाँ<br>○ नहीं<br>○ कोई जवाब नहीं
CALC_CM. CALCULATE: वर्तमान विधि<br>यह स्क्रीन पर नहीं दर्शाया जायेगा।<br>*ODK उत्तरदाता केद्वारा वर्तमान में इस्तेमाल की जा रही विधियों में से सबसे प्रभावी विधि को चॉइस लिस्ट में से चुन लेगा।*	0<br>○ महिला नसबंदी<br>○ पुरुष नसबंदी<br>○ छड़(इम्प्लांट)<br>○ आईयूडी / पिपिआईयूडी<br>○ इंजेक्शन<br>○ गोली<br>○ आपातकालीन गर्भनिरोधक<br>○ पुरुष कंडोम / निरोध<br>○ महिला कंडोम<br>○ मानकदिन/ साइकिल बीड्स<br>○ लैम<br>○ रिदम मेथड<br>○ बाह्य स्खलन(विद्ड्रावल)<br>○ अन्य पारंपरिक तरीके<br>○ कोई जवाब नहीं
302c. क्या आपकेपति/साथी जानते हैं की आप ${current_method_label} का इस्तेमाल कर रही हैं?	(${current_method} != '') and (${current_method} != '-99') and (${current_method_most_effective} …<br>○ हाँ<br>○ नहीं<br>○ कोई जवाब नहीं
302c. क्या आपकेपति/साथी को पता है कि आप परिवार नियोजन का इस्तेमाल कर रही हैं?	(${current_method} = '-99')<br>○ हाँ

	नहीं<br>कोई जवाब नहीं
303. क्या आपकेप्रदाता ने आपको या आपकेसाथी को बताया था कि यह विधि स्थायी है?	selected(${current_method},'female_sterilizi<br>or<br>selected(${current_method},'male_sterilizat<br>…<br>हाँ<br>नहीं<br>कोई जवाब नहीं
305a. आपने कहा कि वर्तमान में आप गर्भनिरोधक विधि का उपयोग नहीं कर रहे हैं। क्या आप सोचते हैं कि भविष्य में किसी भी समय गर्भवती होने से बचने केलिए या देरी करने केलिए गर्भ निरोधक विधि का उपयोग करेंगे ?	${consent_obtained} and (${current_user} != 'yes') and (${pregnant} != 'yes')<br>हाँ<br>नहीं<br>कोई जवाब नहीं
305b. क्या आप भविष्य में किसी भी समय गर्भवती होने से टालने केलिए या देरी करने केलिए गर्भनिरोधक विधि का उपयोग करेगें?	${consent_obtained} and (${current_user} != 'yes') and (${pregnant} = 'yes')<br>हाँ<br>नहीं<br>कोई जवाब नहीं
306a. पिछले 12 महीनों में, क्या आपने गर्भवती होने से टालने केलिए या देरी करने केलिए कुछ किया या गर्भनिरोधक विधि का उपयोग किया?	${consent_obtained} and (${current_user} != 'yes')<br>हाँ<br>नहीं<br>कोई जवाब नहीं

	${recent_user} = 'yes'
306b. आपने हाल ही में कौन सी विधी अपनायी?<br>जांच करें: और कुछ?<br>*सबसे प्रभावी तरीकेका चयन करें। सभी विकल्प देखने केलिए नीचे स्क्राल करें।*	छड़(इम्प्लांट)<br>आईयूडी / पिपिआईयूडी<br>इंजेक्शन<br>गोली<br>आपातकालीन गर्भनिरोधक<br>पुरुष कंडोम / निरोध<br>महिला कंडोम<br>मानकदिन/ साइकिल बीड्स<br>लैम<br>रिदम मेथड<br>बाह्य स्खलन(विद्ड्रावल)<br>अन्य आधुनिक विधियां<br>कोई जवाब नहीं

307. जब आपने ${current_recent_label} विधि का प्रयोग करना आरम्भ किया तो क्या आपने अपने पति/साथी केसाथ गर्भ को रोकने केलिए या देरी करने केबारे में चर्चा की थी ?	${current_or_recent_user}<br>हाँ<br>नहीं<br>पता नहीं<br>कोई जवाब नहीं
308. गर्भनिरोधक विधि का प्रयोग करना मुख्य रूप से आपका निर्णय था, या मुख्य रूप से आपकेपति/साथी का या आप दोनों का संयुक्त निर्णय था?	${current_user} = 'yes'<br>मुख्यतः उत्तरदाता का<br>मुख्यतः पति\साथी का<br>सयुंक्त निर्णय था<br>अन्य<br>कोई जवाब नहीं
308a. जब पिछली बार आपने ${current_recent_label} प्राप्त किया, तब आपने अपनी जेब से कितना पैसा खर्च किया? इसमें विधि को प्राप्त करने केलिए सभी दिए गए शुल्क, उत्पाद, सेवाएं या परिवहन शामिल हैं।<br>*रुपयों में सभी कीमतों को दर्ज करें। अगर पता नहीं, -88 दर्ज करें, अगर कोई जवाब नहीं, -99 दर्ज करें।*	(${current_or_recent_user}) and (${current_recent_method} != 'LAM') and (${current_recent_method …<br>-------------------------------------

	${current_user} = 'yes'
309a. किस महीने एवं वर्ष से आप बिना रुके ${current_recent_label} विधि का प्रयोग कर रहीं हैं।	

यादगार घटनाओ से पीछे की तरफ गणना करे|	
हाल में दिया गया जन्म: ${rec_birth_date}	${recent_birth} != ''
वर्तमान शादी: ${rec_husband_date}	${husband_cohabit_start_recent} != ''
माह:	○ पता नहीं<br>○ जनवरी<br>○ फरवरी<br>○ मार्च<br>○ अप्रैल<br>○ मई<br>○ जून<br>○ जुलाई<br>○ अगस्त<br>○ सितम्बर<br>○ अक्टूबर<br>○ नवम्बर<br>○ दिसम्बर
वर्ष:	Year: ________________

	${recent_user} = 'yes'
309b. आपने ${current_recent_label} का प्रयोग कब बन्द किया ?<br>*तारीख को दर्ज करें।*<br>*तारीख जानने केलिए यदि जरूरत हो तो यादगार घटनाओं केआधार पर पीछे की उम्र की गणना की जानी चाहिए।*<br>*'कोई जवाब नहीं' केलिए माह में 'पता नहीं' और वर्ष में '2020' चुनें*	
माह:	○ पता नहीं<br>○ जनवरी<br>○ फरवरी<br>○ मार्च<br>○ अप्रैल<br>○ मई<br>○ जून<br>○ जुलाई<br>○ अगस्त<br>○ सितम्बर<br>○ अक्टूबर<br>○ नवम्बर<br>○ दिसम्बर
वर्ष:	Year: ________________

	${recent_user} = 'yes'
309c. प्रयोग बंद करने से पूर्व किस महीने एवं वर्ष से आपने ${current_recent_label} का प्रयोग करना आरम्भ किया?<br>*यादगार घटनाओ से पीछे की तरफ गणना करे|*<br>*'कोई जवाब नहीं' केलिए माह में 'पता नहीं' और वर्ष में '2020' चुनें*	
हाल में दिया गया जन्म: ${rec_birth_date}	${recent_birth} != ''
वर्तमान शादी: ${rec_husband_date}	${husband_cohabit_start_recent} != ''
माह:	○ पता नहीं<br>○ जनवरी<br>○ फरवरी<br>○ मार्च<br>○ अप्रैल<br>○ मई<br>○ जून<br>○ जुलाई<br>○ अगस्त<br>○ सितम्बर

	○ अक्टूबर<br>○ नवम्बर<br>○ दिसम्बर
वर्ष:	Year: ____________________________

309d. जाँचें: मैं सुनिश्चित करना चाहती हूँ कि मैं सहीं हूँ: आपने बिना रुकावट के${current_recent_label} का प्रयोग लगातार ${ante_start_using_full_lab} से ${stop_using_full_lab} तक किया है। क्या मै सही हूँ?	(${recent_user} = 'yes') and (${begin_using} != '2020-01-01') and (${stop_using} != '2020-01-01')<br>○ हाँ<br>○ नहीं
पिछली स्क्रीन पर जाएं और सबसे हाल ही में प्रयोग ली गयी विधि की अवधि सुनिश्चित करने हेतु गहराई से जाँचें।<br>*सुझावित जाँचः - अंतिम बार आपने [METHOD] का प्रयोग कब किया था? - आपने [METHOD] का प्रयोग बिना रुकेकितने समय तक किया?*	${recent_start_stop_check} = 'no'
310. आपने क्यों ${current_recent_label} का उपयोग बंद किया?	${consent_obtained} and (${recent_user} = 'yes')<br>☐ अनियमित सेक्स/ पति दूर<br>☐ उपयोग करते समय गर्भधारण<br>☐ गर्भवती होना चाहती थी<br>☐ पति/साथी की अस्वीकृति<br>☐ ज्यादा प्रभावी तरीका चाहती थी<br>☐ कोई विधि उपलब्ध नहीं<br>☐ स्वास्थ्य संबंधी समस्याएं<br>☐ दुष्प्रभाव का डर<br>☐ पहुंच से बाहर/ बहुत दूर<br>☐ बहुत अधिक क़ीमत<br>☐ उपयोग में असुविधा<br>☐ भाग्यवादी<br>☐ गर्भधारण करना कठिन / मासिक धर्म बंद<br>☐ शरीर की प्रक्रियाओं केसाथ हस्तक्षेप<br>☐ अन्य<br>☐ पता नहीं<br>☐ कोई जवाब नहीं

	${current_or_recent_user} and (${current_recent_method} != 'LAM') and (${current_recent_method} != …
311a. आपने ${current_recent_label} का उपयोग ${start_date_lab} से शुरू किया।<br>आपने या आपकेसाथी ने इसे उस समय कहाँ से प्राप्त किया?<br>*सभी विकल्प देखने केलिए नीचे स्क्रॉल करें।*	○ सरकारी/ नगर पालिका अस्पताल<br>○ सरकारी औषधालय<br>○ यूएफडब्लूसी/यूएचसी/यूएचपी<br>○ सीएचसी / ग्रामीण अस्पताल /पी एच सी<br>○ उप-केन्द्र / एएनएम<br>○ सरकारी मोबाइल क्लिनिक<br>○ कैम्प<br>○ आंगनवाड़ी / आईसीडीएस केंद्र<br>○ आशा<br>○ अन्य समुदाय आधारित कार्यकर्ता<br>○ गैर सरकारी (NGO) संगठन या ट्रस्ट अस्पताल / क्लिनिक<br>○ निजी अस्पताल<br>○ निजी डाक्टर/क्लीनिक<br>○ निजी मोबाईल क्लीनिक<br>○ वैद्य/हकीम/होम्योपैथ चिकित्सक<br>○ पारंपरिक नीम हकीम<br>○ फार्मेसी / दवा की दुकान<br>○ प्रशिक्षित दाई (टीबीए)

	○ दुकान<br>○ मित्र/माता-पिता/ रिश्तेदार<br>○ अन्य<br>○ पता नहीं<br>○ कोई जवाब नहीं
312a. आपने जब ${current_recent_label} को प्राप्त किया तो गर्भधारण टालने केलिए या देरी करने केलिए उस विधि से होने वाले दुष्प्रभाव या समस्याओं केबारे में क्या प्रदाता ने आपको बताया था?	${fp_provider_rw} != ''<br>○ हाँ<br>○ नहीं<br>○ कोई जवाब नहीं
312b. क्या आपको बताया गया था कि दुष्प्रभाव या समस्याओं की स्थिति में क्या करना है?	${fp_side_effects} = 'yes'<br>○ हाँ<br>○ नहीं<br>○ कोई जवाब नहीं
313. उस समय परिवार नियोजन प्रदाता द्वारा क्या यह बताया गया था कि आप ${current_recent_label} केअलावा अन्य परिवार नियोजन के तरीकों का भी प्रयोग कर सकते है?	(${fp_provider_rw} != '')<br>○ हाँ<br>○ नहीं<br>○ पता नहीं<br>○ कोई जवाब नहीं
314a. जब आप गए तब क्या आपको वही विधि मिल गई जो आप चाहते थे ?	(${fp_provider_rw} != '')<br>○ हाँ<br>○ नहीं<br>○ कोई जवाब नहीं
314c. जो विधि आप चाहते थे वही विधि आपने क्यों प्राप्त नहीं की ?	${fp_obtain_desired} = 'no'<br>○ उस दिन विधि स्टाक में नहीं थी<br>○ विधि कभी भी उपलब्ध नहीं थी<br>○ प्रदाता विधि प्रदान करने केलिए प्रशिक्षित नहीं था<br>○ प्रदाता ने एक अलग विधि की सिफारिश की<br>○ विधि को प्रयोग करने योग्य नहीं<br>○ एक विधि को नहीं अपनाने का फैसला<br>○ बहुत महंगा<br>○ अन्य<br>○ कोई जवाब नहीं
315a. इस विजिट केदौरान, विधि चुनने में अंतिम फैसला किसका था?	(${fp_provider_rw} != '')<br>○ आपका स्वयं का<br>○ प्रदाता का<br>○ साथी (सहयोगी) का<br>○ आपका और प्रदाता का<br>○ आप और साथी (सहयोगी) का<br>○ अन्य<br>○ पता नहीं<br>○ कोई जवाब नहीं
315b. रिद्हम विधि प्रयोग करने का अंतिम निर्णय किसने लिया था?	${current_recent_method} = 'rhythm'<br>○ आपका स्वयं का<br>○ प्रदाता का<br>○ साथी (सहयोगी) का<br>○ आपका और प्रदाता का<br>○ आप और साथी (सहयोगी) का<br>○ अन्य<br>○ पता नहीं<br>○ कोई जवाब नहीं
315b. लैम विधि प्रयोग करने का अंतिम निर्णय किसने लिया था?	${current_recent_method} = 'LAM'<br>○ आपका स्वयं का

	◯ प्रदाता का<br>◯ साथी (सहयोगी) का<br>◯ आपका और प्रदाता का<br>◯ आप और साथी (सहयोगी) का<br>◯ अन्य<br>◯ पता नहीं<br>◯ कोई जवाब नहीं
316. क्या आप इस प्रदाता केपास वापस जायेंगे ?<br>प्रदाता: ${provider_label}	(${fp_provider_rw} != '') and (${fp_provider_rw} != '-99') and (${fp_provider_rw} != 'friend_relat …<br>◯ हाँ<br>◯ नहीं<br>◯ पता नहीं<br>◯ कोई जवाब नहीं
317. क्या आप अपने रिश्तेदार या दोस्त को इस प्रदाता/ सेवा केंद्र पर जाने केलिए सिफारिश करेगें?<br>प्रदाता: ${provider_label}	(${fp_provider_rw} != '') and (${fp_provider_rw} != '-99') and (${fp_provider_rw} != 'friend_relat …<br>◯ हाँ<br>◯ नहीं<br>◯ पता नहीं<br>◯ कोई जवाब नहीं
SW_1a. ${current_recent_label} का इस्तेमाल ${current_recent_start} से ठीक पहले क्या आप गर्भावस्था से बचने के लिए कुछ कर रही थीं या किसी विधि का इस्तेमाल कर रही थीं?	${current_or_recent_user}<br>◯ हाँ<br>◯ नहीं<br>◯ कोई जवाब नहीं
SW_1b. आप किस विधि का इस्तेमाल कर रही थीं?	${penultimate_method_yn} = 'yes'<br>◯ छड़(इम्प्लांट)<br>◯ आईयूडी / पिपिआईयूडी<br>◯ इंजेक्शन<br>◯ गोली<br>◯ आपातकालीन गर्भनिरोधक<br>◯ पुरुष कंडोम / निरोध<br>◯ महिला कंडोम<br>◯ मानकदिन/ साइकिल बीड्स<br>◯ लैम<br>◯ रिदम मेथड<br>◯ बाह्य स्खलन(विद्ड्रावल)<br>◯ अन्य आधुनिक विधियां<br>◯ कोई जवाब नहीं
PP_1. ${rec_birth_date} में आपकेबच्चे केजन्म केबाद क्या आपने गर्भावस्था से बचने केलिए कुछ किया या किसी विधि का इस्तेमाल किया?	(${recent_birth} <= ${today}) and ((${today} - ${recent_birth}) < 2*366) and (${current_user} != …<br>◯ हाँ<br>◯ नहीं<br>◯ कोई जवाब नहीं
PP_2. ${rec_birth_date} में आपकेद्वारा दिए गए जन्म केकितने समय बाद आपने किसी विधि का इस्तेमाल करना शुरु किया?<br>*आज केलिए 0 दिन दर्ज करें अगली स्क्रीन पर आप X केस्थान पर एक संख्या दर्ज करेंगे।*	(${pp_method_yn} = 'yes') or ((${current_user} = 'yes') and (${recent_birth} <= ${today}) and (($ …<br>◯ X दिन बाद<br>◯ X सप्ताह बाद<br>◯ X माह बाद<br>◯ X वर्ष बाद<br>◯ कोई जवाब नहीं
PP_2. ${pp_method_lab} दर्ज करें।<br>*यदि आज है तो शून्य दिन दर्ज करें, ना कि शून्य सप्ताह/माह/वर्ष।*	${pp_method_units} = 'days' or ${pp_method_units} = 'weeks' or ${pp_method_units} =

	'months' or ${ …
PP_3. वह विधि क्या थी?	${pp_method_units} = 'days' or ${pp_method_units} = 'weeks' or ${pp_method_units} = 'months' or ${ …<br>◯ महिला नसबंदी<br>◯ पुरुष नसबंदी<br>◯ छड़(इम्प्लांट)<br>◯ आईयूडी / पिपिआईयूडी<br>◯ इंजेक्शन<br>◯ गोली<br>◯ आपातकालीन गर्भनिरोधक<br>◯ पुरुष कंडोम / निरोध<br>◯ महिला कंडोम<br>◯ मानकदिन/ साइकिल बीड्स<br>◯ लैम<br>◯ रिदम मेथड<br>◯ बाह्य स्खलन(विद्ड्रावल)<br>◯ अन्य पारंपरिक तरीके<br>◯ कोई जवाब नहीं
319. क्या आपने कभी भी गर्भधारण नहीं करने/टालने केलिए किसी भी प्रकार की कोशिश की थी ?	${consent_obtained} and not(${current_or_recent_user})<br>◯ हाँ<br>◯ नहीं<br>◯ कोई जवाब नहीं
320. आपकी उम्र कितनी थी जब आपने पहली बार गर्भावस्था को टालने के लिए या देरी करने केलिए किसी विधि का इस्तेमाल किया?<br>उत्तरदाता पिछले जन्मदिन पर ${age} साल की थी।<br>*उम्र वर्ष में दर्ज करें। अगर उत्तरदाता को पता नहीं, -88 दर्ज करें। अगर कोई जवाब नहीं, -99 दर्ज करें।*<br>*उम्र 9 वर्ष से छोटी नहीं हो सकती।*	${fp_ever_used} = 'yes'<br>------------------------------------
321. आपकेउस समय कितने बच्चे जीवित थे, यदि कोई थे तो?<br>नोट: उत्तरदाता ने 201 में कहा है की उसने ${birth_events} बार जन्म दिया है।<br>*कोई जवाब नही केलिए -99 दर्ज करें*	(${age_at_first_use} > 0) and (${birth_events} > 0)<br>------------------------------------

	${fp_ever_used} = 'yes'
322. आपने पहली बार गर्भावस्था को टालने अथवा रोकने केलिए कौन सा तरीका इस्तेमाल किया था।<br>*विधि विकल्प नहीं पढ़ियें । सभी विकल्प देखने केलिए नीचे स्क्रॉल करें।*	◯ महिला नसबंदी<br>◯ पुरुष नसबंदी<br>◯ छड़(इम्प्लांट)<br>◯ आईयूडी / पिपिआईयूडी<br>◯ इंजेक्शन<br>◯ गोली<br>◯ आपातकालीन गर्भनिरोधक<br>◯ पुरुष कंडोम / निरोध<br>◯ महिला कंडोम<br>◯ मानकदिन/ साइकिल बीड्स<br>◯ लैम<br>◯ रिदम मेथड<br>◯ बाह्य स्खलन(विद्ड्रावल)<br>◯ अन्य पारंपरिक तरीके<br>◯ कोई जवाब नहीं

322a क्या आपने पिछले 12 माह में आपातकालीन गर्भनिरोधक का इस्तेमाल किया है?<br>पूछें: असुरक्षित संभोग केबाद गर्भावस्था को रोकने केलिए महिलाऐं एक आपातकालीन उपाय केरूप में ये विशेष गोलियाँ 3-5 दिनों केभीतर किसी भी समय ले सकती हैं।	(${current_recent_method} != 'emergency') and ${consent_obtained}<br>◯ हाँ<br>◯ नहीं<br>◯ कोई जवाब नहीं

	((${current_user} = 'no')) and (((${more_children_none} = 'no_children') or …
323a. आपने कहा कि आपको बच्चा जल्दी नहीं चाहिए व आप गर्भावस्था को रोकने केलिए कोई परिवार नियोजन की विधि का उपयोग भी नहीं कर रही है	((${wait_birth_none} = 'months') or (${wait_birth_none} = 'years')) and (${more_children_none} = ' …
323a. आपने कहा कि आपको और अधिक बच्चे जल्दी नहीं चाहिए व आप गर्भावस्था को रोकने केलिए कोई परिवार नियोजन की विधि का उपयोग भी नहीं कर रही है	((${wait_birth_none} = 'months') or (${wait_birth_none} = 'years')) and (${more_children_none} = ' …
323a. आपने कहा कि आपको कोई बच्चा नहीं चाहिए व आप गर्भावस्था को रोकने केलिए कोई परिवार नियोजन की विधि का उपयोग भी नहीं कर रही है	(${more_children_none} = 'no_children') and (${ever_birth} = 'no')
323a. आपने कहा कि आपको और बच्चे नहीं चाहिए व आप गर्भावस्था को रोकने केलिए कोई परिवार नियोजन की विधि का उपयोग भी नहीं कर रही है	(${more_children_some} = 'no_children') and (${ever_birth} = 'yes')
क्या आप हमें इसका मुख्य कारण बता सकती हैं कि गर्भावस्था को रोकने के लिए आप किसी विधि का उपयोग क्यों नहीं कर रहीं हैं।<br>गहराई से पूछें: कोई भी अन्य कारण है?<br>*उल्लिखित सभी कारणों का चयन करें। "जानती नहीं" या "कोई जवाब नहीं" अन्य विकल्पों केसाथ चयन नहीं कर सकते। अगर 104 का उत्तर "हाँ, वर्तमान में शादीशुदा है" तो " शादीशुदा नहीं" चयन नहीं कर सकते। सभी विकल्प देखने के लिए नीचे स्क्रॉल करें।*	☐ शादीशुदा नहीं<br>☐ कम बार सेक्स/ पति दूर<br>☐ मासिक धर्म बंद / गर्भाशय आपरेशन से निकाल लिया गया है<br>☐ उपजाऊ नहीं<br>☐ पिछले जन्म केबाद से माहवारी नहीं<br>☐ स्तनपान<br>☐ पति कई दिनों से दूर है<br>☐ भगवान केऊपर / भाग्यवादी<br>☐ उत्तरदाता ने विरोध किया<br>☐ पति / पार्टनर ने विरोध किया<br>☐ दूसरों ने विरोध किया<br>☐ धार्मिक निषेध<br>☐ कोई विधि नहीं जानती<br>☐ कोई स्रोत नहीं जानती<br>☐ दुष्प्रभाव का डर<br>☐ स्वास्थ्य संबंधी समस्याएं<br>☐ पहुंच से बाहर/ बहुत दूर<br>☐ बहुत अधिक क़ीमत<br>☐ पसंदीदा तरीका उपलब्ध नहीं<br>☐ कोई विधि उपलब्ध नहीं<br>☐ उपयोग में असुविधा<br>☐ शरीर की प्रक्रियाओं केसाथ हस्तक्षेप<br>☐ अन्य<br>☐ पता नहीं<br>☐ कोई जवाब नहीं

323b. गर्भनिरोधक विधि का प्रयोग नहीं करना मुख्य रूप से आपका स्वयं का निर्णय था, या मुख्य रूप से आपकेपति/साथी का या आप दोनों का संयुक्त निर्णय था?	(${current_user} != 'yes') and ${consent_obtained}<br>○ मुख्यतः उत्तरदाता का<br>○ मुख्यतः पति\साथी का<br>○ सयुंक्त निर्णय था<br>○ अन्य<br>○ कोई जवाब नहीं
324. पिछले 12 महीनों में, परिवार नियोजन केबारे में आप से कोई आंगनवाड़ी कार्यकर्ता, आशा (ASHA), या अन्य सामुदायिक स्वास्थ्य कार्यकर्ता द्वारा बात की गयी?	${consent_obtained}<br>○ हाँ<br>○ नहीं<br>○ कोई जवाब नहीं
325a. पिछले 12 महीनों में, आपने अपने या अपने बच्चों की देखभाल या किन्ही भी स्वास्थ्य सेवोकेलिए केलिए किसी स्वास्थ्य केंद्र या शिविर (कैंप) का दौरा किया?<br>*किन्हीं भी स्वास्थ्य सेवाओं केलिए*	not(${ever_birth} = 'yes') and ${consent_obtained}<br>○ हाँ<br>○ नहीं<br>○ कोई जवाब नहीं
325a. पिछले 12 महीनों में, आपने अपने या अपने बच्चों की देखभाल या	${ever_birth} = 'yes'

किन्ही भी स्वास्थ्य सवाओं केलिए किसी स्वास्थ्य केंद्र या शिविर (कैंप) का दौरा किया?<br>*किन्हीं भी स्वास्थ्य सेवाओं केलिए*	◯ हाँ<br>◯ नहीं<br>◯ कोई जवाब नहीं
325b. उस स्वास्थ्य सुविधा पर किसी भी स्टाफ सदस्य ने आपसे परिवार नियोजन केसाधनों केबारे में बात की?	(${visited_fac_none} = 'yes') or (${visited_fac_some} = 'yes')<br>◯ हाँ<br>◯ नहीं<br>◯ कोई जवाब नहीं

			${consent_obtained}
326. पिछले कुछ महीनों में आपने:			
	हाँ	नहीं	कोई जवाब नहीं
326a. रेडियो पर परिवार नियोजन केबारे में सुना है?	◯	◯	◯
326b. टेलीविजन पर परिवार नियोजन केबारे में कुछ भी देखा है?	◯	◯	◯
326c. किसी अखबार या पत्रिका में परिवार नियोजन केबारे में पढ़ा है ?	◯	◯	◯
326d. मोबाईल फोन पर परिवार नियोजन केबारे में कोई रिकार्डेड या लिखित सन्देश प्राप्त किया है?	◯	◯	◯

भाग 4 - यौन गतिविधियाँ

उपस्थिति जांचे

अब मैं जीवन केकुछ महत्वपूर्ण मुद्दों पर बेहतर समझ बनाने केलिए आपसे यौन गतिविधियों से सम्बंधित कुछ सवाल पूछूंगी। मैं आप को फिर से आश्वासित करना चाहती हूँ कि आपकेजवाबों को गोपनीय रखा जायेगा और किसी केसाथ साझा नहीं किया जायेगा। यदि कोई ऐसा प्रश्न हो जिसका आप जवाब न देना चाहें तो मुझे बता दीजियेगा, मैं अगले प्रश्न पर चली जाऊँगी।	${consent_obtained}

	${consent_obtained}
401a. आपकी उम्र कितनी थी जब आपने पहली बार संभोग किया?	
वर्तमान आयु: ${age}	
जीवित जन्मो की संख्या: ${birth_events}	${birth_events} > 0
उत्तरदाता गर्भवती है	${pregnant} = 'yes'
उम्र वर्ष में दर्ज करें।<br>*अगर उत्तरदाता ने कभी संभोग नहीं किया, -77 दर्ज करें। अगर उत्तरदाता को पता नहीं, -88 दर्ज करें। अगर उत्तरदाता केपास कोई जवाब नहीं है, -99 दर्ज करें।*	------------------------------------

	((${age_at_first_sex} >= 0) or (${age_at_first_sex} =-88) or (${age_at_first_sex} =-99))
402. पिछली बार कब आपने संभोग किया?	

402. ${last_time_sex_lab}. दर्ज करें<br>*यदि जवाब "आज" है तो 0 दिन दर्ज करें, 0 सप्ताह, माह और वर्ष नहीं*<br>*पहले सम्भोग की उम्र और गर्भावस्था कि स्थिति से मेल खाना चाहिए*	${last_time_sex} = 'days' or ${last_time_sex} = 'weeks' or ${last_time_sex} = 'months' or ${last_t …<br>------------------------------------
LCL_403. यदि आप एक बार बिना किसी गर्भनिरोध का प्रयोग किये यौन सम्बन्ध बनाती हैं तो आपकेअनुसार आपकेगर्भवती होने की कितनी सम्भावनाएं है?<br>*सभी विकल्पों को पढ़े*	${consent_obtained}<br>◯ निश्चित रूप से हाँ<br>◯ शायद हाँ<br>◯ शायद नहीं<br>◯ निश्चित रूप से नहीं<br>◯ पता नहीं<br>◯ कोई जवाब नहीं
LCL_404. यदि आप बिना गर्भनिरोधक केएक वर्ष तक नियमित रूप से सप्ताह में दो बार, यौन सम्बन्ध बनाएं तो आपकेअनुसार आपकेगर्भवती होने की कितनी सम्भावनाएं है?<br>*सभी विकल्पों को पढ़े*	${consent_obtained}<br>◯ निश्चित रूप से हाँ<br>◯ शायद हाँ<br>◯ शायद नहीं<br>◯ निश्चित रूप से नहीं<br>◯ पता नहीं<br>◯ कोई जवाब नहीं

किशोरी स्वास्थय

AH_1. जहाँ आप रहते है वहां किस उम्र में लड़कियाँ कानूनन विवाह कर सकती हैं?	(${consent_obtained}) and (${age} <= 19)<br><br>------------------------------------
AH_2. आप अपनी शादी केनिर्णय में कितना शामिल थीं?	(${age} <= 19) and ((${marital_status} = 'currently_married') or (${marital_status} = 'currently_l …<br>○ बहुत शामिल<br>○ बहुत शामिल नहीं<br>○ बिलकुल भी नहीं<br>○ कोई जवाब नहीं
AH_3. आपकेअनुसार आप अपने शादी केनिर्णय में कितना शामिल रहेंगी – बहुत शामिल, बहुत शामिल नहीं/बिलकुल भी नहीं?	(${age} <= 19) and (${marital_status} = 'never_married')<br>○ बहुत शामिल<br>○ बहुत शामिल नहीं<br>○ बिलकुल भी नहीं<br>○ कोई जवाब नहीं
AH_4. क्या शादी की वजह से आपने स्थायी रूप से विद्यालय/कॉलेज जाना बंद कर दिया?	(${age} <= 19) and ((${marital_status} = 'currently_married') or (${marital_status} = 'currently_l …<br>○ हाँ<br>○ नहीं<br>○ कोई जवाब नहीं
AH_5. कृपया निम्न प्रश्न का उत्तर; पूर्णतः सहमत, सहमत, न सहमत न ही असहमत, असहमत या पूर्णतः असहमत केरूप में दीजियेl<br>मैं अपनी पढ़ाई ख़त्म होने तक शादी केलिए रुकुंगीl	(${age} <= 19) and (${marital_status} = 'never_married') and((${enrolled} = 'yes') or (${return} …<br>○ पुर्णतः सहमत<br>○ सहमत<br>○ न सहमत न ही असहमत<br>○ असहमत<br>○ पुर्णतः असहमत<br>○ कोई जवाब नहीं
AH_6. पहली बार जब आपने सम्भोग किया क्या आपको लगता है कि आपको किसी केसाथ सम्भोग करने केलिए और इंतज़ार करना चाहिए था, इतना इंतज़ार नहीं करना चाहिए था; या वह ठीक समय था?	(${age} <= 19) and ((${marital_status} = 'currently_married') or (${marital_status} = 'currently_l …<br>○ और इंतज़ार करना चाहिए था<br>○ इतना इंतज़ार नहीं करना चाहिए था<br>○ वह ठीक समय था<br>○ लागू नहीं होता<br>○ कोई जवाब नहीं
AH_7. जब आपने पहली बार सम्भोग किया तब आप दोनों समान रूप से इच्छुक थे या आप में से कोई एक अधिक इच्छुक था?	(${first_sex_timing} != 'na') and (${age} <= 19) and ((${marital_status} = 'currently_married') or …<br>○ समान रूप से इच्छुक<br>○ उत्तरदाता अधिक इच्छुक<br>○ साथी अधिक इच्छुक<br>○ कोई जवाब नहीं
AH_8. आपकेपहली बार सम्भोग केअनुभव को ध्यान में रखते हुए इनमे से कौन से कथन आप पर लागू होते हैं:<br>*सभी विकल्पों को जोर से पढ़े तथा लागू होने वाले सभी विकल्पों का चयन करें*	(${first_sex_timing} != 'na') and (${age} <= 19) and ((${marital_status} = 'currently_married') or …<br>☐ मैं उत्सुक थी<br>☐ मैं भावनाओं में बह गयी थी

	☐ मैं किसी पदार्थ केप्रभाव में थी<br>☐ मुझसे जैसी अपेक्षा थी मैं वही कर रही थी<br>☐ मुझसे ज़बरदस्ती की गयी थी<br>☐ उपरोक्त में से कोई नहीं<br>☐ कोई जवाब नहीं
AH_9. आपने जब पहली बार सम्भोग किया, तब उस निर्णय में आप कितना शामिल थीं-बहुत ज्यादा,बहुत ज्यादा नहीं या बिलकुल भी नहीं	(${first_sex_timing} != 'na') and (${age} <= 19) and ((${marital_status} = 'currently_married') or …<br>○ बहुत शामिल<br>○ बहुत शामिल नहीं<br>○ बिलकुल भी नहीं<br>○ कोई जवाब नहीं
AH_10. जब आपने पहली बार सम्भोग किया था तब क्या आप और आपके साथी गर्भावस्था को टालना चाहते थे?	(${first_sex_timing} != 'na') and (${age} <= 19) and ((${marital_status} = 'currently_married') or …<br>○ हाँ<br>○ नहीं<br>○ कोई जवाब नहीं
AH_11. क्या आपने या आपकेसाथी ने गर्भधारण से बचने केलिए कुछ किया या किसी विधि का उपयोग किया था?	${first_sex_avoid_preg} = 'yes'<br>○ हाँ<br>○ नहीं<br>○ कोई जवाब नहीं
AH_12. आपने कौनसी विधि का इस्तेमाल किया था?	${first_sex_method_yn} = 'yes'<br>○ महिला नसबंदी<br>○ पुरुष नसबंदी<br>○ छड़(इम्प्लांट)<br>○ आईयूडी / पिपिआईयूडी<br>○ इंजेक्शन<br>○ गोली<br>○ आपातकालीन गर्भनिरोधक<br>○ पुरुष कंडोम / निरोध<br>○ महिला कंडोम<br>○ मानकदिन/ साइकिल बीड्स<br>○ लैम<br>○ रिदम मेथड<br>○ बाह्य स्खलन(विद्ड्रावल)<br>○ अन्य पारंपरिक तरीके<br>○ कोई जवाब नहीं
AH_13. आपकेमुताबिक आप अपने प्रथम सम्भोग केनिर्णय में कितना शामिल रहेंगी-बहुत ज्यादा,बहुत ज्यादा नहीं या बिलकुल भी नहीं	(${age} <= 19) and (${marital_status} = 'never_married')<br>○ बहुत शामिल<br>○ बहुत शामिल नहीं<br>○ बिलकुल भी नहीं<br>○ कोई जवाब नहीं
AH_14. कृपया निम्न प्रश्न का उत्तर; पूर्णतः सहमत, सहमत, न सहमत न ही असहमत, असहमत या पूर्णतः असहमत केरूप में दीजिये।<br>मैं जल्दी से जल्दी बच्चे को जन्म देना चाहती थी क्योंकि मैं माँ बनना चाहती थी।	(${age} <= 19) and ((${ever_birth} = 'yes') or (${pregnant} = 'yes'))<br>○ पुर्णतः सहमत<br>○ सहमत<br>○ न सहमत न ही असहमत<br>○ असहमत<br>○ पुर्णतः असहमत<br>○ कोई जवाब नहीं
AH_15. कृपया निम्न प्रश्न का उत्तर; पूर्णतः सहमत, सहमत, न सहमत न ही असहमत, असहमत या पूर्णतः असहमत केरूप में दीजिये।	(${age} <= 19) and (${ever_birth} = 'no') and (${pregnant} = 'no')

मैं जल्दी से जल्दी बच्चे को जन्म देना चाहती हूं क्योंकि मैं माँ बनना चाहती हूं।	पुर्णतः सहमत<br>सहमत<br>न सहमत न ही असहमत<br>असहमत<br>पुर्णतः असहमत<br>कोई जवाब नहीं
AH_16. क्या गर्भावस्था केकारण आपने स्थायी रूप से स्कूल जाना छोड़ दिया था?	(${age} <= 19) and (${ever_birth} = 'no') and (${pregnant} = 'no') and (${return} = 'no')<br>हाँ<br>नहीं<br>कोई जवाब नहीं
AH_17. अपने पहले बच्चे को जन्म देने से पहले क्या आप अपनी पढ़ाई पूरी करेंगी?	(${age} <= 19) and (${marital_status} = 'never_married')<br>हाँ<br>नहीं<br>पता नहीं<br>कोई जवाब नहीं
AH_18. क्या आपको लगता है कि आप अगले 12 माह में किसी गर्भनिरोधक विधि का प्रयोग गर्भावस्था को टालने या देरी करने केलिए करेंगी?	(${consent_obtained}) and (${age} <= 19) and (${pregnant} != 'yes') and (${current_user} != 'yes') …<br>हाँ<br>नहीं<br>पता नहीं<br>कोई जवाब नहीं
AH_19. क्या आपको कोई ऐसी जगह पता है, जहाँ से आप परिवार नियोजन विधि को प्राप्त कर सकती हैं?	(${consent_obtained}) and (${age} <= 19)<br>हाँ<br>नहीं<br>कोई जवाब नहीं

	(${consent_obtained}) and (${age} <= 19)
कृपया निम्न प्रश्नों केलिए उत्तर; पूर्णतः सहमत, सहमत, न सहमत न ही असहमत, असहमत या पूर्णतः असहमत केरूप में दीजिये।	
AH_20. गर्भनिरोधक विधि का प्रयोग करने से युवा महिलाएं स्वयं को परिवार केलिए तैयार कर सकती हैं।	पुर्णतः सहमत<br>सहमत<br>न सहमत न ही असहमत<br>असहमत<br>पुर्णतः असहमत<br>कोई जवाब नहीं
AH_21. गर्भनिरोधक केइस्तमाल से साथ युवा दंपत्ति एक दूसरे केसाथ चिंतामुक्त होकर सम्बन्ध बना सकते हैं।	पुर्णतः सहमत<br>सहमत<br>न सहमत न ही असहमत<br>असहमत<br>पुर्णतः असहमत<br>कोई जवाब नहीं
AH_22. गर्भनिरोध केवल शादीशुदा महिलाओं केलिए है।	पुर्णतः सहमत<br>सहमत<br>न सहमत न ही असहमत<br>असहमत<br>पुर्णतः असहमत<br>कोई जवाब नहीं
AH_23. गर्भनिरोध केवल उन महिलाओं केलिए हैं जो और बच्चे नहीं चाहती हैं।	पुर्णतः सहमत<br>सहमत<br>न सहमत न ही असहमत

	असहमत<br>पुर्णतः असहमत<br>कोई जवाब नहीं
AH_24. गर्भनिरोधक केइस्तेमाल से महिलाओं में प्रजनन क्षमता में कमी हो सकती है या स्वास्थय को हानि पहुँच सकती है।	पुर्णतः सहमत<br>सहमत<br>न सहमत न ही असहमत<br>असहमत<br>पुर्णतः असहमत<br>कोई जवाब नहीं
AH_25. किशोरी या युवा महिलाएं जो गर्भनिरोधक का इस्तेमाल करती हैं उन्हें अच्छी नजर से नहीं देखा जाता है।	पुर्णतः सहमत<br>सहमत<br>न सहमत न ही असहमत<br>असहमत<br>पुर्णतः असहमत<br>कोई जवाब नहीं
AH_26. क्लिनिक या कहीं और से गर्भनिरोधक लेने में मुझे अत्यन्त संकोच होगा।	पुर्णतः सहमत<br>सहमत<br>न सहमत न ही असहमत<br>असहमत<br>पुर्णतः असहमत<br>कोई जवाब नहीं

	(${consent_obtained}) and (${age} <= 19)
यदि कानूनी रूप से उपलब्ध हों तो भविष्य में निम्न विकल्पों का इस्तेमाल करना चाहूंगी:	
AH_27. ऐसी गर्भनिरोधक विधि जो मैं हर तीन महीने में स्वयं को इंजेक्शन लगाकर प्रयोग कर सकूँ।	हाँ<br>शायद<br>नहीं<br>कोई जवाब नहीं
AH_28. ऐसी गर्भनिरोधक गोली जो कि मुझे बिना दवा केपर्चे केदवाओं की दुकान से प्राप्त हो सके।	हाँ<br>शायद<br>नहीं<br>कोई जवाब नहीं
AH_29. ऐसी गोली जो माहवारी वापस लाने में मदद करे यदि मैं गर्भावस्था को लेकर चिंतित हूँ और बिना दवा का पर्चा दिखाए मिल जाए।	हाँ<br>शायद<br>नहीं<br>कोई जवाब नहीं

AH_30. क्या आपने पिछले 12 माह में किसी मीडिया या अन्य कहीं बाल विवाह से सम्बंधित कोई सन्देश सुना या देखा है?	${consent_obtained}<br>हाँ<br>नहीं<br>कोई जवाब नहीं
AH_31. आपकेअनुसार ,स्वयं की शादी केसम्बन्ध में लड़की केनिर्णय को कितना शामिल किया जाता है -बहुत शामिल ,बहुत शामिल नहीं या बिलकुल भी नहीं?	${consent_obtained}<br>बहुत शामिल<br>बहुत शामिल नहीं<br>बिलकुल भी नहीं<br>कोई जवाब नहीं
AH_32. परिवार शुरू करने केसम्बन्ध में लड़की केनिर्णय को कितना शामिल किया जाता है -बहुत शामिल ,बहुत शामिल नहीं या बिलकुल भी नहीं?	${consent_obtained}<br>बहुत शामिल<br>बहुत शामिल नहीं<br>बिलकुल भी नहीं<br>कोई जवाब नहीं
AH_33. लडकियाँ कितनी पढ़ी लिखी होनी चाहिए?<br>*उत्तर सालों में दर्ज करे*<br>*पता नहीं केलिए -88 दर्ज करें कोई जवाब नहीं केलिए -99 दर्ज करे*	${consent_obtained}<br>------------------------------------

AH_34. लड़केकितने पढ़ा-लिखा होना चाहिए?<br>*उत्तर सालों में दर्ज करे*<br>*पता नहीं केलिए -88 दर्ज करें कोई जवाब नहीं केलिए -99 दर्ज करे*	${consent_obtained}<br>------------------------------------

खण्ड 7.1 विश्वासपात्र मित्र

701. अब मैं आपकी सबसे घनिष्ठ सहेली और रिश्तेदारों केबारे में सवाल पूछूंगी, जिससे आप अपनी निजी बातें साझा करती हैं, और वह भी आपसे अपनी सभी बातें साझा करती हो । राजस्थान में आपकी ऐसी कितनी सहेलीयाँ हैं और जो 15-49 साल की उम्र की हैं?<br>*पता नहीं केलिए -88 दर्ज करें कोई जवाब नहीं केलिए -99 दर्ज करे*	${consent_obtained}<br>------------------------------------
702a. राजस्थान में अपनी सबसे घनिष्ठ मित्र की कल्पना कीजिये जिसकी उम्र 15-49 केबीच हो और जिसकेसाथ आप अपनी निजी बातें साझा करती हों। साक्षात्कार की आसानी केलिए अपनी इस सहेली को आप कोई भी अन्य नाम दे दीजिये।	${friend_count} > 0<br>------------------------------------
703a. ${friend1_name} अपने पिछले जन्मदिन पर कितने वर्ष की थी?<br>*पता नहीं केलिए -88 दर्ज करें कोई जवाब नहीं केलिए -99 दर्ज करे*	${friend_count} > 0<br>------------------------------------
704a. ${friend1_name} केशिक्षा का सबसे उच्चतम स्तर क्या है?	${friend_count} > 0<br>◯ कोई शिक्षा प्राप्त नहीं की<br>◯ प्राथमिक<br>◯ माध्यमिक<br>◯ उच्च माध्यमिक<br>◯ स्नातक या उससे ज्यादा<br>◯ कोई जवाब नहीं
702b. राजस्थान में अपनी दूसरी सबसे घनिष्ठ मित्र की कल्पना कीजिये जिसकी उम्र 15-49 केबीच हो और जिसकेसाथ आप अपनी निजी बातें साझा करती हों। साक्षात्कार की आसानी केलिए अपनी इस सहेली को आप कोई भी अन्य नाम दे दीजिये।	${friend_count} > 1<br>------------------------------------
703b. ${friend2_name} अपने पिछले जन्मदिन पर कितने वर्ष की थी?<br>*पता नहीं केलिए -88 दर्ज करें कोई जवाब नहीं केलिए -99 दर्ज करे*	${friend_count} > 1<br>------------------------------------
704b. ${friend2_name} केशिक्षा का सबसे उच्चतम स्तर क्या है?	${friend_count} > 1<br>◯ कोई शिक्षा प्राप्त नहीं की<br>◯ प्राथमिक<br>◯ माध्यमिक<br>◯ उच्च माध्यमिक<br>◯ स्नातक या उससे ज्यादा<br>◯ कोई जवाब नहीं

खण्ड 7.2 गर्भपात

उपस्थिति जांचे

अगले कुछ सवाल आपकेसमुदाय में गर्भ समापन से सम्बंधित है। गर्भ समापन राजस्थान में बहुत प्रचलित है और हम केवल यह बेहतर समझना चाहते हैं कि महिलाएं अपनी माहवारी वापस शुरू करने केलिए क्या करती हैं। मैं आपको पुनः याद दिलाना चाहती हूँ कि यह सर्वे पूरी तरह गोपनीय है और आपके जवाबों को किसी से साझा नहीं किया जायेगा। यदि कोई ऐसा प्रश्न हो जिसका आप जवाब न देना चाहें तो मुझे बता दीजियेगा, हम अगले प्रश्न पर चले जायेंगे।<br>*जारी रखने केलिए OK दबाएँ*	${consent_obtained}<br>☐ ओ.के
705. कभी-कभी महिलाएं इस बात से चिंतित हो जाती है की वे गर्भवती तो नहीं हो गयी है या गर्भवती नहीं होना चाहती हैं और वह गर्भ समापन केलिए कुछ उपाय करती हैं। यह आपकेसमुदाय में कितना सामान्य है?<br>*सभी विकल्पों को पढ़े*	${consent_obtained}<br>◯ बहुत सामान्य<br>◯ कुछ सामान्य<br>◯ ज्यादा सामान्य नहीं<br>◯ बिलकुल सामान्य नहीं<br>◯ पता नहीं<br>◯ कोई जवाब नहीं
706. आपकेसमुदाय में महिलाएं जो या तो गर्भवती हैं या कहीं वे गर्भवती है तो नहीं इस बात से चिंतित हैं, किन तरीकों से अपना गर्भ समापन करवाती हैं? अन्य कोई?	${consent_obtained}<br>☐ ऑपरेशन प्रक्रिया जैसे की डी एन सी,सफाई

विकल्पों को न पढ़ें एक से ज्यादा जवाब चुन सकते हैं	☐ मिसोप्रोस्टोल या मीफीप्रिस्टोन नाम की गोलियों केद्वारा जैसे की 500/700 की गोली<br>☐ गोलियाँ जो कि बुखार या मलेरिया के इलाज केलिए ली जाती हैं जैसे की पेरासिटामोल,कोम्बिफ्लेम या हरे पत्ते वाली दवाई<br>☐ अन्य गोलियाँ<br>☐ पारंपरिक विधियाँ जैसे जड़ी बूटीयाँ<br>☐ घरेलू नुस्खे<br>☐ योनी में सामग्री डालकर<br>☐ अन्य<br>☐ पता नहीं<br>☐ कोई जवाब नहीं
707. इनमे से सबसे प्रचलित तरीका कौनसा है?	`count-selected(${abt_ways}) > 1`<br>○ ऑपरेशन प्रक्रिया जैसे की डी एन सी,सफाई<br>○ मिसोप्रोस्टोल या मीफीप्रिस्टोन नाम की गोलियों केद्वारा जैसे की 500/700 की गोली<br>○ गोलियाँ जो कि बुखार या मलेरिया के इलाज केलिए ली जाती हैं जैसे की पेरासिटामोल,कोम्बिफ्लेम या हरे पत्ते वाली दवाई<br>○ अन्य गोलियाँ<br>○ पारंपरिक विधियाँ जैसे जड़ी बूटीयाँ<br>○ घरेलू नुस्खे<br>○ योनी में सामग्री डालकर<br>○ अन्य<br>○ पता नहीं<br>○ कोई जवाब नहीं<br>`selected(${abt_ways}, filter_list) or (filter_list = 'always')`
708. महिलाएं गर्भ समापन केऑपरेशन, जैसे डीएंडसी या "सफाई", केलिए कहाँ जाती हैं? कोई और जगह?<br>*विकल्पों को न पढ़ें एक से ज्यादा जवाब चुन सकते हैं*	`(selected(${abt_ways},'surgery'))`<br>☐ सरकारी/ नगर पालिका अस्पताल<br>☐ सरकारी औषधालय<br>☐ यूएफडब्लूसी/यूएचसी/यूएचपी<br>☐ सीएचसी / ग्रामीण अस्पताल /पी एच सी<br>☐ उप-केन्द्र / एएनएम<br>☐ सरकारी मोबाइल क्लिनिक<br>☐ कैम्प<br>☐ आंगनवाड़ी / आईसीडीएस केंद्र<br>☐ आशा<br>☐ अन्य समुदाय आधारित कार्यकर्ता<br>☐ गैर सरकारी (NGO) संगठन या ट्रस्ट अस्पताल / क्लिनिक<br>☐ निजी अस्पताल<br>☐ निजी डाक्टर/क्लीनिक<br>☐ निजी मोबाईल क्लीनिक<br>☐ वैद्य/हकीम/होम्योपैथ चिकित्सक<br>☐ पारंपरिक नीम हकीम<br>☐ फार्मेसी / दवा की दुकान<br>☐ प्रशिक्षित दाई (टीबीए)<br>☐ दुकान<br>☐ मित्र/माता-पिता/ रिश्तेदार<br>☐ अन्य

	☐ पता नहीं<br>☐ कोई जवाब नहीं
709. इनमे से सबसे प्रचलित जगह कौनसी है?	count-selected(${abt_surg_where}) > 1<br>○ सरकारी/ नगर पालिका अस्पताल<br>○ सरकारी औषधालय<br>○ यूएफडब्लूसी/यूएचसी/यूएचपी<br>○ सीएचसी / ग्रामीण अस्पताल /पी एच सी<br>○ उप-केन्द्र / एएनएम<br>○ सरकारी मोबाइल क्लिनिक<br>○ कैम्प<br>○ आंगनवाड़ी / आईसीडीएस केंद्र<br>○ आशा<br>○ अन्य समुदाय आधारित कार्यकर्ता<br>○ गैर सरकारी (NGO) संगठन या ट्रस्ट अस्पताल / क्लिनिक<br>○ निजी अस्पताल<br>○ निजी डाक्टर/क्लीनिक<br>○ निजी मोबाईल क्लीनिक<br>○ वैद्य/हकीम/होम्योपैथ चिकित्सक<br>○ पारंपरिक नीम हकीम<br>○ फार्मेसी / दवा की दुकान<br>○ प्रशिक्षित दाई (टीबीए)<br>○ दुकान<br>○ मित्र/माता-पिता/ रिश्तेदार<br>○ अन्य<br>○ पता नहीं<br>○ कोई जवाब नहीं<br>selected(${abt_surg_where}, filter_list) or (filter_list = 'always')
710. महिलायें गर्भ समापन केलिए गोलियां कहाँ से लेती हैं? कोई और जगह?	(selected(${abt_ways}, 'pills_abortion')) or (selected(${abt_ways}, 'pills_fever')) or (selected($ …<br>☐ सरकारी/ नगर पालिका अस्पताल<br>☐ सरकारी औषधालय<br>☐ यूएफडब्लूसी/यूएचसी/यूएचपी<br>☐ सीएचसी / ग्रामीण अस्पताल /पी एच सी<br>☐ उप-केन्द्र / एएनएम<br>☐ सरकारी मोबाइल क्लिनिक<br>☐ कैम्प<br>☐ आंगनवाड़ी / आईसीडीएस केंद्र<br>☐ आशा<br>☐ अन्य समुदाय आधारित कार्यकर्ता<br>☐ गैर सरकारी (NGO) संगठन या ट्रस्ट अस्पताल / क्लिनिक<br>☐ निजी अस्पताल<br>☐ निजी डाक्टर/क्लीनिक<br>☐ निजी मोबाईल क्लीनिक<br>☐ वैद्य/हकीम/होम्योपैथ चिकित्सक<br>☐ पारंपरिक नीम हकीम<br>☐ फार्मेसी / दवा की दुकान<br>☐ प्रशिक्षित दाई (टीबीए)<br>☐ दुकान

	☐ मित्र/माता-पिता/ रिश्तेदार<br>☐ अन्य<br>☐ पता नहीं<br>☐ कोई जवाब नहीं
711. इनमे से कौनसी जगह सबसे प्रचलित है?	count-selected(${abt_meds_where}) > 1<br>○ सरकारी/ नगर पालिका अस्पताल<br>○ सरकारी औषधालय<br>○ यूएफडब्लूसी/यूएचसी/यूएचपी<br>○ सीएचसी / ग्रामीण अस्पताल /पी एच सी<br>○ उप-केन्द्र / एएनएम<br>○ सरकारी मोबाइल क्लिनिक<br>○ कैम्प<br>○ आंगनवाड़ी / आईसीडीएस केंद्र<br>○ आशा<br>○ अन्य समुदाय आधारित कार्यकर्ता<br>○ गैर सरकारी (NGO) संगठन या ट्रस्ट अस्पताल / क्लिनिक<br>○ निजी अस्पताल<br>○ निजी डाक्टर/क्लीनिक<br>○ निजी मोबाईल क्लीनिक<br>○ वैद्य/हकीम/होम्योपैथ चिकित्सक<br>○ पारंपरिक नीम हकीम<br>○ फार्मेसी / दवा की दुकान<br>○ प्रशिक्षित दाई (टीबीए)<br>○ दुकान<br>○ मित्र/माता-पिता/ रिश्तेदार<br>○ अन्य<br>○ पता नहीं<br>○ कोई जवाब नहीं<br>selected(${abt_meds_where}, filter_list) or (filter_list = 'always')
712a.i. अब मैं आपसे${friend1_name} केबारे में कुछ और सवाल भी पूछना चाहूँगी। क्या वह कभी चिंतित थी कि वह गर्भवती हो सकती हैं या हो गयी थी और तब क्या कभी उन्होंने अपनी गर्भावस्था को समाप्त करने केलिए कुछ किया था?<br>*गहराई से जांचे की गर्भविस्था ख़त्म होने में सफलता प्राप्त हुयी यदि नहीं तो 'नहीं'दर्ज करें।*	○ हाँ मैं निश्चित हूँ<br>○ हाँ, शायद<br>○ नहीं<br>○ पता नहीं<br>○ कोई जवाब नहीं
713a.i. यह आखरी बार किस वर्ष में हुआ था?<br>*यदि एक से ज्यादा बार हुआ तो सबसे आखिर वाले को इंगित करें*<br>*'पता नहीं'व 'कोई जवाब नहीं 'केलिए 2020 दर्ज करे*	(${friend1_abt_yn} = 'yes') or (${friend1_abt_yn} = 'likely')<br>Year: ______________________________
714a.i. महिलाएं कभी-कभी गर्भ धारण को रोकने केलिए बहुत चीज़ें करती हैं। क्या ${friend1_name} ने कभी गर्भ समापन केलिए एक से ज्यादा बार कुछ किया था?	(${friend1_abt_yn} = 'yes') or (${friend1_abt_yn} = 'likely')<br>○ हाँ मैं निश्चित हूँ<br>○ हाँ, शायद<br>○ नहीं<br>○ पता नहीं<br>○ कोई जवाब नहीं
715a.i. उन्होंने गर्भ समापन केलिए सबसे पहले क्या करने का प्रयत्न किया था?	(${friend1_abt_mult_yn} = 'yes') or (${friend1_abt_mult_yn} = 'likely')<br>○ ऑपरेशन प्रक्रिया जैसे की डी एन सी,सफाई<br>○ मिसोप्रोस्टोल या मीफीप्रिस्टोन नाम की गोलियों केद्वारा जैसे की 500/700 की गोली<br>○ गोलियाँ जो कि बुखार या मलेरिया के

	इलाज केलिए ली जाती हैं जैसे की पेरासिटामोल,कोम्बिफ्लेम या हरे पत्ते वाली दवाई<br>○ अन्य गोलियाँ<br>○ पारंपरिक विधियाँ जैसे जड़ी बूटीयाँ<br>○ घरेलू नुस्खे<br>○ योनी में सामग्री डालकर<br>○ अन्य<br>○ पता नहीं<br>○ कोई जवाब नहीं
715a.i. उन्होंने क्या किया जिससे उनका गर्भ समापन हो गया?	(${friend1_abt_mult_yn} = 'no') or (${friend1_abt_mult_yn} = '-88')<br>○ ऑपरेशन प्रक्रिया जैसे की डी एन सी,सफाई<br>○ मिसोप्रोस्टोल या मीफीप्रिस्टोन नाम की गोलियों केद्वारा जैसे की 500/700 की गोली<br>○ गोलियाँ जो कि बुखार या मलेरिया के इलाज केलिए ली जाती हैं जैसे की पेरासिटामोल,कोम्बिफ्लेम या हरे पत्ते वाली दवाई<br>○ अन्य गोलियाँ<br>○ पारंपरिक विधियाँ जैसे जड़ी बूटीयाँ<br>○ घरेलू नुस्खे<br>○ योनी में सामग्री डालकर<br>○ अन्य<br>○ पता नहीं<br>○ कोई जवाब नहीं
716a.i. वो उस प्रक्रिया केलिए कहाँ गयी?	(${friend1_abt_first} = 'surgery') or (${friend1_abt_only} = 'surgery')<br>○ सरकारी/ नगर पालिका अस्पताल<br>○ सरकारी औषधालय<br>○ यूएफडब्लूसी/यूएचसी/यूएचपी<br>○ सीएचसी / ग्रामीण अस्पताल /पी एच सी<br>○ उप-केन्द्र / एएनएम<br>○ सरकारी मोबाइल क्लिनिक<br>○ कैम्प<br>○ आंगनवाड़ी / आईसीडीएस केंद्र<br>○ आशा<br>○ अन्य समुदाय आधारित कार्यकर्ता<br>○ गैर सरकारी (NGO) संगठन या ट्रस्ट अस्पताल / क्लिनिक<br>○ निजी अस्पताल<br>○ निजी डाक्टर/क्लीनिक<br>○ निजी मोबाईल क्लीनिक<br>○ वैद्य/हकीम/होम्योपैथ चिकित्सक<br>○ पारंपरिक नीम हकीम<br>○ फार्मेसी / दवा की दुकान<br>○ प्रशिक्षित दाई (टीबीए)<br>○ दुकान<br>○ मित्र/माता-पिता/ रिश्तेदार<br>○ अन्य<br>○ पता नहीं<br>○ कोई जवाब नहीं

717a.i. उन्हें दवाईयाँ कहाँ से मिली?	(${friend1_abt_first} = 'pills_abortion') or (${friend1_abt_only} = 'pills_abortion') or (${friend …<br>◯ सरकारी/ नगर पालिका अस्पताल<br>◯ सरकारी औषधालय<br>◯ यूएफडब्लूसी/यूएचसी/यूएचपी<br>◯ सीएचसी / ग्रामीण अस्पताल /पी एच सी<br>◯ उप-केन्द्र / एएनएम<br>◯ सरकारी मोबाइल क्लिनिक<br>◯ कैम्प<br>◯ आंगनवाड़ी / आईसीडीएस केंद्र<br>◯ आशा<br>◯ अन्य समुदाय आधारित कार्यकर्ता<br>◯ गैर सरकारी (NGO) संगठन या ट्रस्ट अस्पताल / क्लिनिक<br>◯ निजी अस्पताल<br>◯ निजी डाक्टर/क्लीनिक<br>◯ निजी मोबाईल क्लीनिक<br>◯ वैद्य/हकीम/होम्योपैथ चिकित्सक<br>◯ पारंपरिक नीम हकीम<br>◯ फार्मेसी / दवा की दुकान<br>◯ प्रशिक्षित दाई (टीबीए)<br>◯ दुकान<br>◯ मित्र/माता-पिता/ रिश्तेदार<br>◯ अन्य<br>◯ पता नहीं<br>◯ कोई जवाब नहीं
718a.i. उन्होनें आखिर में क्या किया जिससे अंत में उनका गर्भ समापन हो गया?	(${friend1_abt_mult_yn} = 'yes') or (${friend1_abt_mult_yn} = 'likely')<br>◯ ऑपरेशन प्रक्रिया जैसे की डी एन सी,सफाई<br>◯ मिसोप्रोस्टोल या मीफीप्रिस्टोन नाम की गोलियों केद्वारा जैसे की 500/700 की गोली<br>◯ गोलियाँ जो कि बुखार या मलेरिया के इलाज केलिए ली जाती हैं जैसे की पेरासिटामोल,कोम्बिफ्लेम या हरे पत्ते वाली दवाई<br>◯ अन्य गोलियाँ<br>◯ पारंपरिक विधियाँ जैसे जड़ी बूटीयाँ<br>◯ घरेलू नुस्खे<br>◯ योनी में सामग्री डालकर<br>◯ अन्य<br>◯ पता नहीं<br>◯ कोई जवाब नहीं
719a.i. वो उस प्रक्रिया केलिए कहाँ गयी?	${friend1_abt_last} = 'surgery'<br>◯ सरकारी/ नगर पालिका अस्पताल<br>◯ सरकारी औषधालय<br>◯ यूएफडब्लूसी/यूएचसी/यूएचपी<br>◯ सीएचसी / ग्रामीण अस्पताल /पी एच सी<br>◯ उप-केन्द्र / एएनएम<br>◯ सरकारी मोबाइल क्लिनिक<br>◯ कैम्प

	◯ आंगनवाड़ी / आईसीडीएस केंद्र<br>◯ आशा<br>◯ अन्य समुदाय आधारित कार्यकर्ता<br>◯ गैर सरकारी (NGO) संगठन या ट्रस्ट अस्पताल / क्लिनिक<br>◯ निजी अस्पताल<br>◯ निजी डाक्टर/क्लीनिक<br>◯ निजी मोबाईल क्लीनिक<br>◯ वैद्य/हकीम/होम्योपैथ चिकित्सक<br>◯ पारंपरिक नीम हकीम<br>◯ फार्मेसी / दवा की दुकान<br>◯ प्रशिक्षित दाई (टीबीए)<br>◯ दुकान<br>◯ मित्र/माता-पिता/ रिश्तेदार<br>◯ अन्य<br>◯ पता नहीं<br>◯ कोई जवाब नहीं
720a.i. उन्हें दवाईयाँ कहाँ से मिली?	`(${friend1_abt_last} = 'pills_abortion') or (${friend1_abt_last} = 'pills_fever') or (${friend1_ab …`<br>◯ सरकारी/ नगर पालिका अस्पताल<br>◯ सरकारी औषधालय<br>◯ यूएफडब्लूसी/यूएचसी/यूएचपी<br>◯ सीएचसी / ग्रामीण अस्पताल /पी एच सी<br>◯ उप-केन्द्र / एएनएम<br>◯ सरकारी मोबाइल क्लिनिक<br>◯ कैम्प<br>◯ आंगनवाड़ी / आईसीडीएस केंद्र<br>◯ आशा<br>◯ अन्य समुदाय आधारित कार्यकर्ता<br>◯ गैर सरकारी (NGO) संगठन या ट्रस्ट अस्पताल / क्लिनिक<br>◯ निजी अस्पताल<br>◯ निजी डाक्टर/क्लीनिक<br>◯ निजी मोबाईल क्लीनिक<br>◯ वैद्य/हकीम/होम्योपैथ चिकित्सक<br>◯ पारंपरिक नीम हकीम<br>◯ फार्मेसी / दवा की दुकान<br>◯ प्रशिक्षित दाई (टीबीए)<br>◯ दुकान<br>◯ मित्र/माता-पिता/ रिश्तेदार<br>◯ अन्य<br>◯ पता नहीं<br>◯ कोई जवाब नहीं
721a.i. क्या ${friend1_name} को गर्भ समापन की प्रक्रिया में कोई समस्या हुई और किसी स्वास्थय केंद्र पर इलाज केलिए गयी?<br>*यदि उत्तरदाता कहती है की उसकी दोस्त गर्भावस्था को समाप्त करने केदौरान किसी स्वास्थय केंद्र में गयी थी, तो हम यह जानने में रूचि रखते हैं कि क्या वह किसी अन्य अवसर पर किसी समस्या केइलाज केलिए किसी स्वास्थय केंद्र पर गयी थी।*	`(${friend1_abt_yn} = 'yes') or (${friend1_abt_yn} = 'likely')`<br>◯ हाँ मैं निश्चित हूँ<br>◯ हाँ, शायद<br>◯ नहीं<br>◯ पता नहीं<br>◯ कोई जवाब नहीं
712a.ii. इस घटना केअलावा, क्या ${friend1_name} ने कभी अपनी माहवारी नियमित करने केलिए कभी कुछ किया, जब वह अपनी गर्भावस्था को लेकर चिंतित थीं?<br>*गहराई से जांचे की माहवारी नियमित करने में सफलता प्राप्त हुयी। यदि नहीं तो*	`${friend1_abt_yn} = 'yes'`<br>◯ हाँ मैं निश्चित हूँ<br>◯ हाँ, शायद<br>◯ नहीं

'नहीं" दर्ज करें|	◯ पता नहीं<br>◯ कोई जवाब नहीं
712a.ii. क्या ${friend1_name} जब इस बात केलिए चिंतित थी की वह गर्भवती है तो,उसने कभी अपनी माहवारी नियमित करने केलिए कुछ किया था?<br>*गहराई से जांचे की माहवारी नियमित करने में सफलता प्राप्त हुयी| यदि नहीं तो 'नहीं" दर्ज करें|*	`${friend1_abt_yn} != 'yes'`<br>◯ हाँ मैं निश्चित हूँ<br>◯ हाँ, शायद<br>◯ नहीं<br>◯ पता नहीं<br>◯ कोई जवाब नहीं
713a.ii. यह आखरी बार किस वर्ष में हुआ था?<br>*यदि एक से ज्यादा बार हुआ तो सबसे आखिर वाले को इंगित करें*<br>*'पता नहीं"व 'कोई जवाब नहीं 'केलिए 2020 दर्ज करे*	`(${friend1_reg_yn} = 'yes') or (${friend1_reg_yn} = 'likely')`<br>Year: ------------------------------
714a.ii. महिलाएं कभी-कभी माहवारी को नियमित करने केलिए बहुत चीज़ें करती हैं। क्या ${friend1_name} ने कभी अपनी माहवारी नियमित करने के लिए एक से ज्यादा बार कुछ किया था?	`((${friend1_reg_year} > ${friend1_abt_year}) or (${friend1_abt_year} = '')) and ((${friend1_reg_yn …`<br>◯ हाँ मैं निश्चित हूँ<br>◯ हाँ, शायद<br>◯ नहीं<br>◯ पता नहीं<br>◯ कोई जवाब नहीं
715a.ii. उन्होंने अपनी माहवारी नियमित करने केलिए सबसे पहले क्या किया?	`((${friend1_reg_year} > ${friend1_abt_year}) or (${friend1_abt_year} = '')) and ((${friend1_reg_mu …`<br>◯ ऑपरेशन प्रक्रिया जैसे की डी एन सी,सफाई<br>◯ मिसोप्रोस्टोल या मीफीप्रिस्टोन नाम की गोलियों केद्वारा जैसे की 500/700 की गोली<br>◯ गोलियाँ जो कि बुखार या मलेरिया के इलाज केलिए ली जाती हैं जैसे की पेरासिटामोल,कोम्बिफ्लेम या हरे पत्ते वाली दवाई<br>◯ अन्य गोलियाँ<br>◯ पारंपरिक विधियाँ जैसे जड़ी बूटीयाँ<br>◯ घरेलू नुस्खे<br>◯ योनी में सामग्री डालकर<br>◯ अन्य<br>◯ पता नहीं<br>◯ कोई जवाब नहीं
715a.ii. उन्होंने अपनी माहवारी नियमित करने केलिए क्या किया?	`((${friend1_reg_year} > ${friend1_abt_year}) or (${friend1_abt_year} = '')) and ((${friend1_reg_mu …`<br>◯ ऑपरेशन प्रक्रिया जैसे की डी एन सी,सफाई<br>◯ मिसोप्रोस्टोल या मीफीप्रिस्टोन नाम की गोलियों केद्वारा जैसे की 500/700 की गोली<br>◯ गोलियाँ जो कि बुखार या मलेरिया के इलाज केलिए ली जाती हैं जैसे की पेरासिटामोल,कोम्बिफ्लेम या हरे पत्ते वाली दवाई<br>◯ अन्य गोलियाँ<br>◯ पारंपरिक विधियाँ जैसे जड़ी बूटीयाँ<br>◯ घरेलू नुस्खे<br>◯ योनी में सामग्री डालकर<br>◯ अन्य<br>◯ पता नहीं<br>◯ कोई जवाब नहीं
716a.ii. वो उस प्रक्रिया केलिए कहाँ गयी?	`((${friend1_reg_year} >`

	${friend1_abt_year}) or (${friend1_abt_year} = '')) and ((${friend1_reg_fi … ○ सरकारी/ नगर पालिका अस्पताल<br>○ सरकारी औषधालय<br>○ यूएफडब्लूसी/यूएचसी/यूएचपी<br>○ सीएचसी / ग्रामीण अस्पताल /पी एच सी<br>○ उप-केन्द्र / एएनएम<br>○ सरकारी मोबाइल क्लिनिक<br>○ कैम्प<br>○ आंगनवाड़ी / आईसीडीएस केंद्र<br>○ आशा<br>○ अन्य समुदाय आधारित कार्यकर्ता<br>○ गैर सरकारी (NGO) संगठन या ट्रस्ट अस्पताल / क्लिनिक<br>○ निजी अस्पताल<br>○ निजी डाक्टर/क्लीनिक<br>○ निजी मोबाईल क्लीनिक<br>○ वैद्य/हकीम/होम्योपैथ चिकित्सक<br>○ पारंपरिक नीम हकीम<br>○ फार्मेसी / दवा की दुकान<br>○ प्रशिक्षित दाई (टीबीए)<br>○ दुकान<br>○ मित्र/माता-पिता/ रिश्तेदार<br>○ अन्य<br>○ पता नहीं<br>○ कोई जवाब नहीं
717a.ii. उन्हें दवाईयाँ कहाँ से मिली?	((${friend1_reg_year} > ${friend1_abt_year}) or (${friend1_abt_year} = '')) and ((${friend1_reg_fi … ○ सरकारी/ नगर पालिका अस्पताल<br>○ सरकारी औषधालय<br>○ यूएफडब्लूसी/यूएचसी/यूएचपी<br>○ सीएचसी / ग्रामीण अस्पताल /पी एच सी<br>○ उप-केन्द्र / एएनएम<br>○ सरकारी मोबाइल क्लिनिक<br>○ कैम्प<br>○ आंगनवाड़ी / आईसीडीएस केंद्र<br>○ आशा<br>○ अन्य समुदाय आधारित कार्यकर्ता<br>○ गैर सरकारी (NGO) संगठन या ट्रस्ट अस्पताल / क्लिनिक<br>○ निजी अस्पताल<br>○ निजी डाक्टर/क्लीनिक<br>○ निजी मोबाईल क्लीनिक<br>○ वैद्य/हकीम/होम्योपैथ चिकित्सक<br>○ पारंपरिक नीम हकीम<br>○ फार्मेसी / दवा की दुकान<br>○ प्रशिक्षित दाई (टीबीए)<br>○ दुकान<br>○ मित्र/माता-पिता/ रिश्तेदार<br>○ अन्य<br>○ पता नहीं<br>○ कोई जवाब नहीं
718a.ii. उन्होनें आखरी क्या चीज़ की जिससे अंत में उनकी माहवारी वापस आ गयी?	((${friend1_reg_year} > ${friend1_abt_year}) or

```
($[friend1_abt_year} = ''))
   and (($[friend1_reg_mu …
```

- ऑपरेशन प्रक्रिया जैसे की डी एन सी,सफाई
- मिसोप्रोस्टोल या मीफीप्रिस्टोन नाम की गोलियों केद्वारा जैसे की 500/700 की गोली
- गोलियाँ जो कि बुखार या मलेरिया के इलाज केलिए ली जाती हैं जैसे की पेरासिटामोल,कोम्बिफ्लेम या हरे पत्ते वाली दवाई
- अन्य गोलियाँ
- पारंपरिक विधियाँ जैसे जड़ी बूटीयाँ
- घरेलू नुस्खे
- योनी में सामग्री डालकर
- अन्य
- पता नहीं
- कोई जवाब नहीं

719a.ii. वो उस प्रक्रिया केलिए कहाँ गयी?

```
(($[friend1_reg_year} >
 ${friend1_abt_year}) or
($[friend1_abt_year} = ''))
   and ($[friend1_reg_las …
```

- सरकारी/ नगर पालिका अस्पताल
- सरकारी औषधालय
- यूएफडब्लूसी/यूएचसी/यूएचपी
- सीएचसी / ग्रामीण अस्पताल /पी एच सी
- उप-केन्द्र / एएनएम
- सरकारी मोबाइल क्लिनिक
- कैम्प
- आंगनवाड़ी / आईसीडीएस केंद्र
- आशा
- अन्य समुदाय आधारित कार्यकर्ता
- गैर सरकारी (NGO) संगठन या ट्रस्ट अस्पताल / क्लिनिक
- निजी अस्पताल
- निजी डाक्टर/क्लीनिक
- निजी मोबाईल क्लीनिक
- वैद्य/हकीम/होम्योपैथ चिकित्सक
- पारंपरिक नीम हकीम
- फार्मेसी / दवा की दुकान
- प्रशिक्षित दाई (टीबीए)
- दुकान
- मित्र/माता-पिता/ रिश्तेदार
- अन्य
- पता नहीं
- कोई जवाब नहीं

720a.ii. उन्हें दवाईयाँ कहाँ से मिली?

```
(($[friend1_reg_year} >
 ${friend1_abt_year}) or
($[friend1_abt_year} = ''))
   and (($[friend1_reg_la …
```

- सरकारी/ नगर पालिका अस्पताल
- सरकारी औषधालय
- यूएफडब्लूसी/यूएचसी/यूएचपी
- सीएचसी / ग्रामीण अस्पताल /पी एच सी
- उप-केन्द्र / एएनएम
- सरकारी मोबाइल क्लिनिक
- कैम्प
- आंगनवाड़ी / आईसीडीएस केंद्र

	◯ आशा<br>◯ अन्य समुदाय आधारित कार्यकर्ता<br>◯ गैर सरकारी (NGO) संगठन या ट्रस्ट अस्पताल / क्लिनिक<br>◯ निजी अस्पताल<br>◯ निजी डाक्टर/क्लीनिक<br>◯ निजी मोबाईल क्लीनिक<br>◯ वैद्य/हकीम/होम्योपैथ चिकित्सक<br>◯ पारंपरिक नीम हकीम<br>◯ फार्मेसी / दवा की दुकान<br>◯ प्रशिक्षित दाई (टीबीए)<br>◯ दुकान<br>◯ मित्र/माता-पिता/ रिश्तेदार<br>◯ अन्य<br>◯ पता नहीं<br>◯ कोई जवाब नहीं
721a.ii. क्या ${friend1_name} को अपनी माहवारी नियमित करने की प्रक्रिया में कोई समस्या हुई और किसी स्वास्थ्य केंद्र पर इलाज केलिए गयी?<br>*यदि उत्तरदाता कहती है की उसकी दोस्त माहवारी को नियमित करने केदौरान किसी स्वास्थय केंद्र में गयी थी, तो हम यह जानने में रूचि रखते हैं कि क्या वह किसी अन्य अवसर पर किसी समस्या केइलाज केलिए किसी स्वास्थय केंद्र पर गयी थी।*	`((${friend1_reg_year} > ${friend1_abt_year}) or (${friend1_abt_year} = '')) and ((${friend1_reg_yn …`<br>◯ हाँ मैं निश्चित हूँ<br>◯ हाँ, शायद<br>◯ नहीं<br>◯ पता नहीं<br>◯ कोई जवाब नहीं
712b.i. अब मैं आपसे ${friend2_name} केबारे में कुछ और सवाल भी पूछना चाहूँगी। क्या वह कभी चिंतित थी कि वह गर्भवती हो सकती हैं या हो गयी थी और तब क्या कभी उन्होंने अपनी गर्भावस्था को समाप्त करने केलिए कुछ किया था?<br>*गहराई से जांचे की गर्भविस्था ख़त्म होने में सफलता प्राप्त हुयी यदि नहीं तो 'नहीं"दर्ज करें।*	◯ हाँ मैं निश्चित हूँ<br>◯ हाँ, शायद<br>◯ नहीं<br>◯ पता नहीं<br>◯ कोई जवाब नहीं
713b.i. यह आखरी बार किस वर्ष में हुआ था?<br>*यदि एक से ज्यादा बार हुआ तो सबसे आखिर वाले को इंगित करें*<br>*'पता नहीं"व 'कोई जवाब नहीं 'केलिए 2020 दर्ज करे*	`(${friend2_abt_yn} = 'yes') or (${friend2_abt_yn} = 'likely')`<br>Year: ______________________________
714b.i. महिलाएं कभी-कभी गर्भ धारण को रोकने केलिए बहुत चीज़ें करती हैं। क्या ${friend2_name} ने कभी गर्भ समापन केलिए एक से ज्यादा बार कुछ किया था?	`(${friend2_abt_yn} = 'yes') or (${friend2_abt_yn} = 'likely')`<br>◯ हाँ मैं निश्चित हूँ<br>◯ हाँ, शायद<br>◯ नहीं<br>◯ पता नहीं<br>◯ कोई जवाब नहीं
715b.i. उन्होंने गर्भ समापन केलिए सबसे पहले क्या करने का प्रयत्न किया था?	`(${friend2_abt_mult_yn} = 'yes') or (${friend2_abt_mult_yn} = 'likely')`<br>◯ ऑपरेशन प्रक्रिया जैसे की डी एन सी,सफाई<br>◯ मिसोप्रोस्टोल या मीफीप्रिस्टोन नाम की गोलियों केद्वारा जैसे की 500/700 की गोली<br>◯ गोलियाँ जो कि बुखार या मलेरिया के इलाज केलिए ली जाती हैं जैसे की पेरासिटामोल,कोम्बिफ्लेम या हरे पत्ते वाली दवाई<br>◯ अन्य गोलियाँ<br>◯ पारंपरिक विधियाँ जैसे जड़ी बूटीयाँ<br>◯ घरेलू नुस्खे<br>◯ योनी में सामग्री डालकर<br>◯ अन्य<br>◯ पता नहीं

	◯ कोई जवाब नहीं
715b.i. उन्होंने क्या किया जिससे उनका गर्भ समापन हो गया?	(${friend2_abt_mult_yn} = 'no') or (${friend2_abt_mult_yn} = '-88')<br>◯ ऑपरेशन प्रक्रिया जैसे की डी एन सी,सफाई<br>◯ मिसोप्रोस्टोल या मीफीप्रिस्टोन नाम की गोलियों केद्वारा जैसे की 500/700 की गोली<br>◯ गोलियाँ जो कि बुखार या मलेरिया के इलाज केलिए ली जाती हैं जैसे की पेरासिटामोल,कोम्बिफ्लेम या हरे पत्ते वाली दवाई<br>◯ अन्य गोलियाँ<br>◯ पारंपरिक विधियाँ जैसे जड़ी बूटीयाँ<br>◯ घरेलू नुस्खे<br>◯ योनी में सामग्री डालकर<br>◯ अन्य<br>◯ पता नहीं<br>◯ कोई जवाब नहीं
716b.i. वो उस प्रक्रिया केलिए कहाँ गयी?	(${friend2_abt_first} = 'surgery') or (${friend2_abt_only} = 'surgery')<br>◯ सरकारी/ नगर पालिका अस्पताल<br>◯ सरकारी औषधालय<br>◯ यूएफडब्लूसी/यूएचसी/यूएचपी<br>◯ सीएचसी / ग्रामीण अस्पताल /पी एच सी<br>◯ उप-केन्द्र / एएनएम<br>◯ सरकारी मोबाइल क्लिनिक<br>◯ कैम्प<br>◯ आंगनवाड़ी / आईसीडीएस केंद्र<br>◯ आशा<br>◯ अन्य समुदाय आधारित कार्यकर्ता<br>◯ गैर सरकारी (NGO) संगठन या ट्रस्ट अस्पताल / क्लिनिक<br>◯ निजी अस्पताल<br>◯ निजी डाक्टर/क्लीनिक<br>◯ निजी मोबाईल क्लीनिक<br>◯ वैद्य/हकीम/होम्योपैथ चिकित्सक<br>◯ पारंपरिक नीम हकीम<br>◯ फार्मेसी / दवा की दुकान<br>◯ प्रशिक्षित दाई (टीबीए)<br>◯ दुकान<br>◯ मित्र/माता-पिता/ रिश्तेदार<br>◯ अन्य<br>◯ पता नहीं<br>◯ कोई जवाब नहीं
717b.i. उन्हें दवाईयाँ कहाँ से मिली?	(${friend2_abt_first} = 'pills_abortion') or (${friend2_abt_only} = 'pills_abortion') or (${friend …<br>◯ सरकारी/ नगर पालिका अस्पताल<br>◯ सरकारी औषधालय<br>◯ यूएफडब्लूसी/यूएचसी/यूएचपी<br>◯ सीएचसी / ग्रामीण अस्पताल /पी एच सी

	○ उप-केन्द्र / एएनएम<br>○ सरकारी मोबाइल क्लिनिक<br>○ कैम्प<br>○ आंगनवाड़ी / आईसीडीएस केंद्र<br>○ आशा<br>○ अन्य समुदाय आधारित कार्यकर्ता<br>○ गैर सरकारी (NGO) संगठन या ट्रस्ट अस्पताल / क्लिनिक<br>○ निजी अस्पताल<br>○ निजी डाक्टर/क्लीनिक<br>○ निजी मोबाईल क्लीनिक<br>○ वैद्य/हकीम/होम्योपैथ चिकित्सक<br>○ पारंपरिक नीम हकीम<br>○ फार्मेसी / दवा की दुकान<br>○ प्रशिक्षित दाई (टीबीए)<br>○ दुकान<br>○ मित्र/माता-पिता/ रिश्तेदार<br>○ अन्य<br>○ पता नहीं<br>○ कोई जवाब नहीं
718b.i. उन्होनें आखिर में क्या किया जिससे अंत में उनका गर्भ समापन हो गया?	(${friend2_abt_mult_yn} = 'yes') or (${friend2_abt_mult_yn} = 'likely')<br>○ ऑपरेशन प्रक्रिया जैसे की डी एन सी,सफाई<br>○ मिसोप्रोस्टोल या मीफीप्रिस्टोन नाम की गोलियों केद्वारा जैसे की 500/700 की गोली<br>○ गोलियाँ जो कि बुखार या मलेरिया के इलाज केलिए ली जाती हैं जैसे की पेरासिटामोल,कोम्बिफ्लेम या हरे पत्ते वाली दवाई<br>○ अन्य गोलियाँ<br>○ पारंपरिक विधियाँ जैसे जड़ी बूटीयाँ<br>○ घरेलू नुस्खे<br>○ योनी में सामग्री डालकर<br>○ अन्य<br>○ पता नहीं<br>○ कोई जवाब नहीं
719b.i. वो उस प्रक्रिया केलिए कहाँ गयी?	${friend2_abt_last} = 'surgery'<br>○ सरकारी/ नगर पालिका अस्पताल<br>○ सरकारी औषधालय<br>○ यूएफडब्लूसी/यूएचसी/यूएचपी<br>○ सीएचसी / ग्रामीण अस्पताल /पी एच सी<br>○ उप-केन्द्र / एएनएम<br>○ सरकारी मोबाइल क्लिनिक<br>○ कैम्प<br>○ आंगनवाड़ी / आईसीडीएस केंद्र<br>○ आशा<br>○ अन्य समुदाय आधारित कार्यकर्ता<br>○ गैर सरकारी (NGO) संगठन या ट्रस्ट अस्पताल / क्लिनिक<br>○ निजी अस्पताल<br>○ निजी डाक्टर/क्लीनिक<br>○ निजी मोबाईल क्लीनिक

	○ वैद्य/हकीम/होम्योपैथ चिकित्सक<br>○ पारंपरिक नीम हकीम<br>○ फार्मेसी / दवा की दुकान<br>○ प्रशिक्षित दाई (टीबीए)<br>○ दुकान<br>○ मित्र/माता-पिता/ रिश्तेदार<br>○ अन्य<br>○ पता नहीं<br>○ कोई जवाब नहीं
720b.i. उन्हें दवाईयाँ कहाँ से मिली?	(${friend2_abt_last} = 'pills_abortion') or (${friend2_abt_last} = 'pills_fever') or (${friend2_ab …<br>○ सरकारी/ नगर पालिका अस्पताल<br>○ सरकारी औषधालय<br>○ यूएफडब्लूसी/यूएचसी/यूएचपी<br>○ सीएचसी / ग्रामीण अस्पताल /पी एच सी<br>○ उप-केन्द्र / एएनएम<br>○ सरकारी मोबाइल क्लिनिक<br>○ कैम्प<br>○ आंगनवाड़ी / आईसीडीएस केंद्र<br>○ आशा<br>○ अन्य समुदाय आधारित कार्यकर्ता<br>○ गैर सरकारी (NGO) संगठन या ट्रस्ट अस्पताल / क्लिनिक<br>○ निजी अस्पताल<br>○ निजी डाक्टर/क्लीनिक<br>○ निजी मोबाईल क्लीनिक<br>○ वैद्य/हकीम/होम्योपैथ चिकित्सक<br>○ पारंपरिक नीम हकीम<br>○ फार्मेसी / दवा की दुकान<br>○ प्रशिक्षित दाई (टीबीए)<br>○ दुकान<br>○ मित्र/माता-पिता/ रिश्तेदार<br>○ अन्य<br>○ पता नहीं<br>○ कोई जवाब नहीं
721b.i. क्या ${friend2_name} को अपनी गर्भ समापन की प्रक्रिया में कोई समस्या हुई और किसी स्वास्थय केंद्र पर इलाज केलिए गयी?<br>*यदि उत्तरदाता कहती है की उसकी दोस्त गर्भावस्था को समाप्त करने केदौरान किसी स्वास्थय केंद्र में गयी थी, तो हम यह जानने में रूचि रखते हैं कि क्या वह किसी अन्य अवसर पर किसी समस्या केइलाज केलिए किसी स्वास्थय केंद्र पर गयी थी।*	(${friend2_abt_yn} = 'yes') or (${friend2_abt_yn} = 'likely')<br>○ हाँ मैं निश्चित हूँ<br>○ हाँ, शायद<br>○ नहीं<br>○ पता नहीं<br>○ कोई जवाब नहीं
712b.ii. इस घटना केअलावा, क्या ${friend2_name} ने कभी अपनी माहवारी नियमित करने केलिए कभी कुछ किया, जब वह अपनी गर्भावस्था को लेकर चिंतित थीं?<br>*गहराई से जांचे की माहवारी नियमित करने में सफलता प्राप्त हुयी। यदि नहीं तो 'नहीं" दर्ज करें।*	○ हाँ मैं निश्चित हूँ<br>○ हाँ, शायद<br>○ नहीं<br>○ पता नहीं<br>○ कोई जवाब नहीं
712b.ii. क्या ${friend2_name} जब इस बात केलिए चिंतित थी की वह गर्भवती है तो,उसने कभी अपनी माहवारी नियमित करने केलिए कुछ किया था?<br>*गहराई से जांचे की माहवारी नियमित करने में सफलता प्राप्त हुयी। यदि नहीं तो 'नहीं" दर्ज करें।*	○ हाँ मैं निश्चित हूँ<br>○ हाँ, शायद<br>○ नहीं<br>○ पता नहीं<br>○ कोई जवाब नहीं
713b.ii. यह आखरी बार किस वर्ष में हुआ था?	(${friend2_reg_yn} = 'yes') or (${friend2_reg_yn} = 'likely')

यदि एक से ज्यादा बार हुआ तो सबसे आखिर वाले को इंगित करें *'पता नहीं' व 'कोई जवाब नहीं' केलिए 2020 दर्ज करे*	Year: ______________________________
714b.ii. महिलाएं कभी-कभी माहवारी को नियमित करने केलिए बहुत चीज़ें करती हैं। क्या ${friend2_name} ने कभी अपनी माहवारी नियमित करने के लिए एक से ज्यादा बार कुछ किया था?	((${friend2_reg_year} > ${friend2_abt_year}) or (${friend2_abt_year} = '')) and ((${friend2_reg_yn …<br>○ हाँ मैं निश्चित हूँ<br>○ हाँ, शायद<br>○ नहीं<br>○ पता नहीं<br>○ कोई जवाब नहीं
715b.ii. उन्होंने अपनी माहवारी नियमित करने केलिए सबसे पहले क्या किया?	((${friend2_reg_year} > ${friend2_abt_year}) or (${friend2_abt_year} = '')) and ((${friend2_reg_mu …<br>○ ऑपरेशन प्रक्रिया जैसे की डी एन सी,सफाई<br>○ मिसोप्रोस्टोल या मीफीप्रिस्टोन नाम की गोलियों केद्वारा जैसे की 500/700 की गोली<br>○ गोलियाँ जो कि बुखार या मलेरिया के इलाज केलिए ली जाती हैं जैसे की पेरासिटामोल,कोम्बिफ्लेम या हरे पत्ते वाली दवाई<br>○ अन्य गोलियाँ<br>○ पारंपरिक विधियाँ जैसे जड़ी बूटीयाँ<br>○ घरेलू नुस्खे<br>○ योनी में सामग्री डालकर<br>○ अन्य<br>○ पता नहीं<br>○ कोई जवाब नहीं
715b.ii. उन्होंने अपनी माहवारी नियमित करने केलिए क्या किया?	((${friend2_reg_year} > ${friend2_abt_year}) or (${friend2_abt_year} = '')) and ((${friend2_reg_mu …<br>○ ऑपरेशन प्रक्रिया जैसे की डी एन सी,सफाई<br>○ मिसोप्रोस्टोल या मीफीप्रिस्टोन नाम की गोलियों केद्वारा जैसे की 500/700 की गोली<br>○ गोलियाँ जो कि बुखार या मलेरिया के इलाज केलिए ली जाती हैं जैसे की पेरासिटामोल,कोम्बिफ्लेम या हरे पत्ते वाली दवाई<br>○ अन्य गोलियाँ<br>○ पारंपरिक विधियाँ जैसे जड़ी बूटीयाँ<br>○ घरेलू नुस्खे<br>○ योनी में सामग्री डालकर<br>○ अन्य<br>○ पता नहीं<br>○ कोई जवाब नहीं
716b.ii. वो उस प्रक्रिया केलिए कहाँ गयी?	((${friend2_reg_year} > ${friend2_abt_year}) or (${friend2_abt_year} = '')) and ((${friend2_reg_fi …<br>○ सरकारी/ नगर पालिका अस्पताल<br>○ सरकारी औषधालय<br>○ यूएफडब्लूसी/यूएचसी/यूएचपी<br>○ सीएचसी / ग्रामीण अस्पताल /पी एच सी<br>○ उप-केन्द्र / एएनएम<br>○ सरकारी मोबाइल क्लिनिक

	◯ कैम्प<br>◯ आंगनवाड़ी / आईसीडीएस केंद्र<br>◯ आशा<br>◯ अन्य समुदाय आधारित कार्यकर्ता<br>◯ गैर सरकारी (NGO) संगठन या ट्रस्ट अस्पताल / क्लिनिक<br>◯ निजी अस्पताल<br>◯ निजी डाक्टर/क्लीनिक<br>◯ निजी मोबाईल क्लीनिक<br>◯ वैद्य/हकीम/होम्योपैथ चिकित्सक<br>◯ पारंपरिक नीम हकीम<br>◯ फार्मेसी / दवा की दुकान<br>◯ प्रशिक्षित दाई (टीबीए)<br>◯ दुकान<br>◯ मित्र/माता-पिता/ रिश्तेदार<br>◯ अन्य<br>◯ पता नहीं<br>◯ कोई जवाब नहीं
717b.ii. उन्हें दवाईयाँ कहाँ से मिली?	((${friend2_reg_year} > ${friend2_abt_year}) or (${friend2_abt_year} = '')) and ((${friend2_reg_fi …<br>◯ सरकारी/ नगर पालिका अस्पताल<br>◯ सरकारी औषधालय<br>◯ यूएफडब्लूसी/यूएचसी/यूएचपी<br>◯ सीएचसी / ग्रामीण अस्पताल /पी एच सी<br>◯ उप-केन्द्र / एएनएम<br>◯ सरकारी मोबाइल क्लिनिक<br>◯ कैम्प<br>◯ आंगनवाड़ी / आईसीडीएस केंद्र<br>◯ आशा<br>◯ अन्य समुदाय आधारित कार्यकर्ता<br>◯ गैर सरकारी (NGO) संगठन या ट्रस्ट अस्पताल / क्लिनिक<br>◯ निजी अस्पताल<br>◯ निजी डाक्टर/क्लीनिक<br>◯ निजी मोबाईल क्लीनिक<br>◯ वैद्य/हकीम/होम्योपैथ चिकित्सक<br>◯ पारंपरिक नीम हकीम<br>◯ फार्मेसी / दवा की दुकान<br>◯ प्रशिक्षित दाई (टीबीए)<br>◯ दुकान<br>◯ मित्र/माता-पिता/ रिश्तेदार<br>◯ अन्य<br>◯ पता नहीं<br>◯ कोई जवाब नहीं
718b.ii. उन्होनें आखरी क्या चीज़ की जिससे अंत में उनकी माहवारी वापस आ गयी?	((${friend2_reg_year} > ${friend2_abt_year}) or (${friend2_abt_year} = '')) and ((${friend2_reg_mu …<br>◯ ऑपरेशन प्रक्रिया जैसे की डी एन सी,सफाई<br>◯ मिसोप्रोस्टोल या मीफीप्रिस्टोन नाम की गोलियों केद्वारा जैसे की 500/700 की गोली<br>◯ गोलियाँ जो कि बुखार या मलेरिया के इलाज केलिए ली जाती हैं जैसे की पेरासिटामोल,कोम्बिफ्लेम या हरे पत्ते वाली

	दवाई<br>◯ अन्य गोलियाँ<br>◯ पारंपरिक विधियाँ जैसे जड़ी बूटीयाँ<br>◯ घरेलू नुस्खे<br>◯ योनी में सामग्री डालकर<br>◯ अन्य<br>◯ पता नहीं<br>◯ कोई जवाब नहीं
719b.ii. वो उस प्रक्रिया केलिए कहाँ गयी?	((${friend2_reg_year} > ${friend2_abt_year}) or (${friend2_abt_year} = '')) and (${friend2_reg_las …<br>◯ सरकारी/ नगर पालिका अस्पताल<br>◯ सरकारी औषधालय<br>◯ यूएफडब्लूसी/यूएचसी/यूएचपी<br>◯ सीएचसी / ग्रामीण अस्पताल /पी एच सी<br>◯ उप-केन्द्र / एएनएम<br>◯ सरकारी मोबाइल क्लिनिक<br>◯ कैम्प<br>◯ आंगनवाड़ी / आईसीडीएस केंद्र<br>◯ आशा<br>◯ अन्य समुदाय आधारित कार्यकर्ता<br>◯ गैर सरकारी (NGO) संगठन या ट्रस्ट अस्पताल / क्लिनिक<br>◯ निजी अस्पताल<br>◯ निजी डाक्टर/क्लीनिक<br>◯ निजी मोबाईल क्लीनिक<br>◯ वैद्य/हकीम/होम्योपैथ चिकित्सक<br>◯ पारंपरिक नीम हकीम<br>◯ फार्मेसी / दवा की दुकान<br>◯ प्रशिक्षित दाई (टीबीए)<br>◯ दुकान<br>◯ मित्र/माता-पिता/ रिश्तेदार<br>◯ अन्य<br>◯ पता नहीं<br>◯ कोई जवाब नहीं
720b.ii. उन्हें दवाईयाँ कहाँ से मिली?	((${friend2_reg_year} > ${friend2_abt_year}) or (${friend2_abt_year} = '')) and ((${friend2_reg_la …<br>◯ सरकारी/ नगर पालिका अस्पताल<br>◯ सरकारी औषधालय<br>◯ यूएफडब्लूसी/यूएचसी/यूएचपी<br>◯ सीएचसी / ग्रामीण अस्पताल /पी एच सी<br>◯ उप-केन्द्र / एएनएम<br>◯ सरकारी मोबाइल क्लिनिक<br>◯ कैम्प<br>◯ आंगनवाड़ी / आईसीडीएस केंद्र<br>◯ आशा<br>◯ अन्य समुदाय आधारित कार्यकर्ता<br>◯ गैर सरकारी (NGO) संगठन या ट्रस्ट अस्पताल / क्लिनिक<br>◯ निजी अस्पताल<br>◯ निजी डाक्टर/क्लीनिक<br>◯ निजी मोबाईल क्लीनिक<br>◯ वैद्य/हकीम/होम्योपैथ चिकित्सक

	◯ पारंपरिक नीम हकीम<br>◯ फार्मेसी / दवा की दुकान<br>◯ प्रशिक्षित दाई (टीबीए)<br>◯ दुकान<br>◯ मित्र/माता-पिता/ रिश्तेदार<br>◯ अन्य<br>◯ पता नहीं<br>◯ कोई जवाब नहीं
721b.ii. क्या ${friend2_name} को अपनी माहवारी नियमित करने की प्रक्रिया में कोई समस्या हुई और किसी स्वास्थय केंद्र पर इलाज केलिए गयी?<br>*यदि उत्तरदाता कहती है की उसकी दोस्त माहवारी को नियमित करने केदौरान किसी स्वास्थय केंद्र में गयी थी, तो हम यह जानने में रूचि रखते हैं कि क्या वह किसी अन्य अवसर पर किसी समस्या केइलाज केलिए किसी स्वास्थय केंद्र पर गयी थी।*	`((${friend2_reg_year} > ${friend2_abt_year}) or (${friend2_abt_year} = '')) and ((${friend2_reg_yn …`<br>◯ हाँ मैं निश्चित हूँ<br>◯ हाँ, शायद<br>◯ नहीं<br>◯ पता नहीं<br>◯ कोई जवाब नहीं
722a. अब मैं आपसे आपकेअनुभवों केबारे में कुछ और सवाल भी पूछना चाहूँगी। जब आप गर्भवती थी या चिंतित थीं कि आप गर्भवती हो सकती हैं, तब क्या कभी आपने गर्भ समापन केलिए कुछ किया था?<br>*गहराई से जांचे की गर्भावस्था ख़त्म होने में सफलता प्राप्त हुयी यदि नहीं तो 'नहीं"दर्ज करें।*	◯ हाँ<br>◯ नहीं<br>◯ कोई जवाब नहीं
723a. यह आखरी बार किस वर्ष में हुआ था?<br>*यदि एक से ज्यादा बार हुआ तो सबसे आखिर वाले को इंगित करें*<br>*'पता नहीं"व 'कोई जवाब नहीं 'केलिए 2020 दर्ज करे*	`(${self_abt_yn} = 'yes')`<br>Year: ______________________________
724a. क्या आपने कभी अपने गर्भ समापन केलिए एक से ज्यादा बार कुछ किया था?	`(${self_abt_yn} = 'yes')`<br>◯ हाँ<br>◯ नहीं<br>◯ कोई जवाब नहीं
725a. आपने सबसे पहले क्या किया?	`(${self_abt_mult_yn} = 'yes')`<br>◯ ऑपरेशन प्रक्रिया जैसे की डी एन सी,सफाई<br>◯ मिसोप्रोस्टोल या मीफीप्रिस्टोन नाम की गोलियों केद्वारा जैसे की 500/700 की गोली<br>◯ गोलियाँ जो कि बुखार या मलेरिया के इलाज केलिए ली जाती हैं जैसे की पेरासिटामोल,कोम्बिफ्लेम या हरे पत्ते वाली दवाई<br>◯ अन्य गोलियाँ<br>◯ पारंपरिक विधियाँ जैसे जड़ी बूटीयाँ<br>◯ घरेलू नुस्खे<br>◯ योनी में सामग्री डालकर<br>◯ अन्य<br>◯ पता नहीं<br>◯ कोई जवाब नहीं
725a. आपने क्या किया?	`(${self_abt_mult_yn} = 'no')`<br>◯ ऑपरेशन प्रक्रिया जैसे की डी एन सी,सफाई<br>◯ मिसोप्रोस्टोल या मीफीप्रिस्टोन नाम की गोलियों केद्वारा जैसे की 500/700 की गोली<br>◯ गोलियाँ जो कि बुखार या मलेरिया के इलाज केलिए ली जाती हैं जैसे की पेरासिटामोल,कोम्बिफ्लेम या हरे पत्ते वाली दवाई<br>◯ अन्य गोलियाँ<br>◯ पारंपरिक विधियाँ जैसे जड़ी बूटीयाँ<br>◯ घरेलू नुस्खे

- योनी में सामग्री डालकर
- अन्य
- पता नहीं
- कोई जवाब नहीं

726a. आप उस प्रक्रिया केलिए कहाँ गयी?	(${self_abt_first} = 'surgery') or (${self_abt_only} = 'surgery')<br>○ सरकारी/ नगर पालिका अस्पताल<br>○ सरकारी औषधालय<br>○ यूएफडब्लूसी/यूएचसी/यूएचपी<br>○ सीएचसी / ग्रामीण अस्पताल /पी एच सी<br>○ उप-केन्द्र / एएनएम<br>○ सरकारी मोबाइल क्लिनिक<br>○ कैम्प<br>○ आंगनवाड़ी / आईसीडीएस केंद्र<br>○ आशा<br>○ अन्य समुदाय आधारित कार्यकर्ता<br>○ गैर सरकारी (NGO) संगठन या ट्रस्ट अस्पताल / क्लिनिक<br>○ निजी अस्पताल<br>○ निजी डाक्टर/क्लीनिक<br>○ निजी मोबाईल क्लीनिक<br>○ वैद्य/हकीम/होम्योपैथ चिकित्सक<br>○ पारंपरिक नीम हकीम<br>○ फार्मेसी / दवा की दुकान<br>○ प्रशिक्षित दाई (टीबीए)<br>○ दुकान<br>○ मित्र/माता-पिता/ रिश्तेदार<br>○ अन्य<br>○ पता नहीं<br>○ कोई जवाब नहीं
727a. आपको दवाईयाँ कहाँ से मिली?	(${self_abt_first} = 'pills_abortion') or (${self_abt_only} = 'pills_abortion') or (${self_abt_fir …<br>○ सरकारी/ नगर पालिका अस्पताल<br>○ सरकारी औषधालय<br>○ यूएफडब्लूसी/यूएचसी/यूएचपी<br>○ सीएचसी / ग्रामीण अस्पताल /पी एच सी<br>○ उप-केन्द्र / एएनएम<br>○ सरकारी मोबाइल क्लिनिक<br>○ कैम्प<br>○ आंगनवाड़ी / आईसीडीएस केंद्र<br>○ आशा<br>○ अन्य समुदाय आधारित कार्यकर्ता<br>○ गैर सरकारी (NGO) संगठन या ट्रस्ट अस्पताल / क्लिनिक<br>○ निजी अस्पताल<br>○ निजी डाक्टर/क्लीनिक<br>○ निजी मोबाईल क्लीनिक<br>○ वैद्य/हकीम/होम्योपैथ चिकित्सक<br>○ पारंपरिक नीम हकीम<br>○ फार्मेसी / दवा की दुकान<br>○ प्रशिक्षित दाई (टीबीए)<br>○ दुकान<br>○ मित्र/माता-पिता/ रिश्तेदार

	◯ अन्य<br>◯ पता नहीं<br>◯ कोई जवाब नहीं
728a. आपने आखिर में क्या किया जिससे अंत में आपकी माहवारी वापस आ गयी?	(${self_abt_mult_yn} = 'yes')<br>◯ ऑपरेशन प्रक्रिया जैसे की डी एन सी,सफाई<br>◯ मिसोप्रोस्टोल या मीफीप्रिस्टोन नाम की गोलियों केद्वारा जैसे की 500/700 की गोली<br>◯ गोलियाँ जो कि बुखार या मलेरिया के इलाज केलिए ली जाती हैं जैसे की पेरासिटामोल,कोम्बिफ्लेम या हरे पत्ते वाली दवाई<br>◯ अन्य गोलियाँ<br>◯ पारंपरिक विधियाँ जैसे जड़ी बूटीयाँ<br>◯ घरेलू नुस्खे<br>◯ योनी में सामग्री डालकर<br>◯ अन्य<br>◯ पता नहीं<br>◯ कोई जवाब नहीं
729a. आप उस प्रक्रिया केलिए कहाँ गयी?	${self_abt_last} = 'surgery'<br>◯ सरकारी/ नगर पालिका अस्पताल<br>◯ सरकारी औषधालय<br>◯ यूएफडब्लूसी/यूएचसी/यूएचपी<br>◯ सीएचसी / ग्रामीण अस्पताल /पी एच सी<br>◯ उप-केन्द्र / एएनएम<br>◯ सरकारी मोबाइल क्लिनिक<br>◯ कैम्प<br>◯ आंगनवाड़ी / आईसीडीएस केंद्र<br>◯ आशा<br>◯ अन्य समुदाय आधारित कार्यकर्ता<br>◯ गैर सरकारी (NGO) संगठन या ट्रस्ट अस्पताल / क्लिनिक<br>◯ निजी अस्पताल<br>◯ निजी डाक्टर/क्लीनिक<br>◯ निजी मोबाईल क्लीनिक<br>◯ वैद्य/हकीम/होम्योपैथ चिकित्सक<br>◯ पारंपरिक नीम हकीम<br>◯ फार्मेसी / दवा की दुकान<br>◯ प्रशिक्षित दाई (टीबीए)<br>◯ दुकान<br>◯ मित्र/माता-पिता/ रिश्तेदार<br>◯ अन्य<br>◯ पता नहीं<br>◯ कोई जवाब नहीं
730a. आपको दवाईयाँ कहाँ से मिली?	(${self_abt_last} = 'pills_abortion') or (${self_abt_last} = 'pills_fever') or (${self_abt_last} = …<br>◯ सरकारी/ नगर पालिका अस्पताल<br>◯ सरकारी औषधालय<br>◯ यूएफडब्लूसी/यूएचसी/यूएचपी<br>◯ सीएचसी / ग्रामीण अस्पताल /पी एच सी<br>◯ उप-केन्द्र / एएनएम<br>◯ सरकारी मोबाइल क्लिनिक

	○ कैम्प<br>○ आंगनवाड़ी / आईसीडीएस केंद्र<br>○ आशा<br>○ अन्य समुदाय आधारित कार्यकर्ता<br>○ गैर सरकारी (NGO) संगठन या ट्रस्ट अस्पताल / क्लिनिक<br>○ निजी अस्पताल<br>○ निजी डाक्टर/क्लीनिक<br>○ निजी मोबाईल क्लीनिक<br>○ वैद्य/हकीम/होम्योपैथ चिकित्सक<br>○ पारंपरिक नीम हकीम<br>○ फार्मेसी / दवा की दुकान<br>○ प्रशिक्षित दाई (टीबीए)<br>○ दुकान<br>○ मित्र/माता-पिता/ रिश्तेदार<br>○ अन्य<br>○ पता नहीं<br>○ कोई जवाब नहीं
731a. क्या आपको अपनी गर्भावस्था को समाप्त करने की प्रक्रिया में कोई समस्या हुई और किसी स्वास्थय केंद्र पर इलाज केलिए गयी?<br>*यदि उत्तरदाता कहती है कि वह गर्भावस्था को समाप्त करने केदौरान किसी स्वास्थय केंद्र में गयी थी, तो हम यह जानने में रुचि रखते हैं कि क्या वह किसी अन्य अवसर पर किसी समस्या केइलाज केलिए किसी स्वास्थय केंद्र पर गयी थी।*	(${self_abt_yn} = 'yes')<br>○ हाँ<br>○ नहीं<br>○ पता नहीं<br>○ कोई जवाब नहीं
732a. क्या आपने निम्न में से किसी व्यक्ति को इस अनुभव केबारे में बताया?<br>*सभी विकल्पों को जोर से पढ़े तथा लागू होने वाले सभी विकल्पों का चयन करें*	(${self_abt_yn} = 'yes')<br>☐ पति/पुरुष साथी<br>☐ बहन<br>☐ भाई<br>☐ माँ<br>☐ पिता<br>☐ अन्य रिश्तेदार<br>☐ मित्र 1: ${friend1_name}<br>☐ मित्र 2: ${friend2_name}<br>☐ अन्य दोस्त<br>☐ अन्य<br>☐ पता नहीं<br>☐ कोई जवाब नहीं<br>(${friend1_name} != '' and ${friend1_name} != '-99' and filter_list = 'friend1') or (${friend2_name} != '' and ${friend2_name} != '-99' and filter_list = 'friend2') or (filter_list = 'always')
722b. इस घटना केअलावा, क्या आपने कभी अपनी माहवारी नियमित करने केलिए कभी कुछ किया, जब आप अपनी गर्भावस्था को लेकर चिंतित थीं?<br>*गहराई से जांचे की माहवारी नियमित करने में सफलता प्राप्त हुयी। यदि नहीं तो 'नहीं" दर्ज करें।*	${self_abt_yn} = 'yes'<br>○ हाँ<br>○ नहीं<br>○ कोई जवाब नहीं
722b. जब आप अपनी गर्भावस्था को ले कर चिंतित थी ,तो क्या आपने अपनी माहवारी नियमित करने केलिए कभी कुछ किया?<br>*गहराई से जांचे की माहवारी नियमित करने में सफलता प्राप्त हुयी। यदि नहीं तो 'नहीं" दर्ज करें।*	${self_abt_yn} != 'yes'<br>○ हाँ<br>○ नहीं<br>○ कोई जवाब नहीं
723b. यह आखरी बार किस वर्ष में हुआ था?<br>*यदि एक से ज्यादा बार हुआ तो सबसे आखिर वाले को इंगित करें*<br>*'पता नहीं"व 'कोई जवाब नहीं'केलिए 2020 दर्ज करे*	(${self_reg_yn} = 'yes')<br>Year: ------------------------------
724b. क्या आपने कभी अपनी माहवारी नियमित करने केलिए एक से ज्यादा बार कुछ किया था?	((${self_reg_year} > ${self_abt_year}) or (${self_abt_year} = '')) and ((${self_reg_yn} = 'yes'))

	◯ हाँ<br>◯ नहीं<br>◯ कोई जवाब नहीं
725b. आपने सबसे पहले क्या किया?	((${self_reg_year} > ${self_abt_year}) or (${self_abt_year} = '')) and ((${self_reg_mult_yn} = 'ye …<br>◯ ऑपरेशन प्रक्रिया जैसे की डी एन सी,सफाई<br>◯ मिसोप्रोस्टोल या मीफीप्रिस्टोन नाम की गोलियों केद्वारा जैसे की 500/700 की गोली<br>◯ गोलियाँ जो कि बुखार या मलेरिया के इलाज केलिए ली जाती हैं जैसे की पेरासिटामोल,कोम्बिफ्लेम या हरे पत्ते वाली दवाई<br>◯ अन्य गोलियाँ<br>◯ पारंपरिक विधियाँ जैसे जड़ी बूटीयाँ<br>◯ घरेलू नुस्खे<br>◯ योनी में सामग्री डालकर<br>◯ अन्य<br>◯ पता नहीं<br>◯ कोई जवाब नहीं
725b. आपने क्या किया?	((${self_reg_year} > ${self_abt_year}) or (${self_abt_year} = '')) and ((${self_reg_mult_yn} = 'no …<br>◯ ऑपरेशन प्रक्रिया जैसे की डी एन सी,सफाई<br>◯ मिसोप्रोस्टोल या मीफीप्रिस्टोन नाम की गोलियों केद्वारा जैसे की 500/700 की गोली<br>◯ गोलियाँ जो कि बुखार या मलेरिया के इलाज केलिए ली जाती हैं जैसे की पेरासिटामोल,कोम्बिफ्लेम या हरे पत्ते वाली दवाई<br>◯ अन्य गोलियाँ<br>◯ पारंपरिक विधियाँ जैसे जड़ी बूटीयाँ<br>◯ घरेलू नुस्खे<br>◯ योनी में सामग्री डालकर<br>◯ अन्य<br>◯ पता नहीं<br>◯ कोई जवाब नहीं
726b. आप उस प्रक्रिया केलिए कहाँ गयी?	((${self_reg_year} > ${self_abt_year}) or (${self_abt_year} = '')) and ((${self_reg_first} = 'surg …<br>◯ सरकारी/ नगर पालिका अस्पताल<br>◯ सरकारी औषधालय<br>◯ यूएफडब्लूसी/यूएचसी/यूएचपी<br>◯ सीएचसी / ग्रामीण अस्पताल /पी एच सी<br>◯ उप-केन्द्र / एएनएम<br>◯ सरकारी मोबाइल क्लिनिक<br>◯ कैम्प<br>◯ आंगनवाड़ी / आईसीडीएस केंद्र<br>◯ आशा<br>◯ अन्य समुदाय आधारित कार्यकर्ता<br>◯ गैर सरकारी (NGO) संगठन या ट्रस्ट अस्पताल / क्लिनिक<br>◯ निजी अस्पताल<br>◯ निजी डाक्टर/क्लीनिक

	निजी मोबाईल क्लीनिक<br>वैद्य/हकीम/होम्योपैथ चिकित्सक<br>पारंपरिक नीम हकीम<br>फार्मेसी / दवा की दुकान<br>प्रशिक्षित दाई (टीबीए)<br>दुकान<br>मित्र/माता-पिता/ रिश्तेदार<br>अन्य<br>पता नहीं<br>कोई जवाब नहीं
727b. आपको दवाईयाँ कहाँ से मिली?	((${self_reg_year} > ${self_abt_year}) or (${self_abt_year} = '')) and ((${self_reg_first} = 'pill …<br>सरकारी/ नगर पालिका अस्पताल<br>सरकारी औषधालय<br>यूएफडब्लूसी/यूएचसी/यूएचपी<br>सीएचसी / ग्रामीण अस्पताल /पी एच सी<br>उप-केन्द्र / एएनएम<br>सरकारी मोबाइल क्लिनिक<br>कैम्प<br>आंगनवाड़ी / आईसीडीएस केंद्र<br>आशा<br>अन्य समुदाय आधारित कार्यकर्ता<br>गैर सरकारी (NGO) संगठन या ट्रस्ट अस्पताल / क्लिनिक<br>निजी अस्पताल<br>निजी डाक्टर/क्लीनिक<br>निजी मोबाईल क्लीनिक<br>वैद्य/हकीम/होम्योपैथ चिकित्सक<br>पारंपरिक नीम हकीम<br>फार्मेसी / दवा की दुकान<br>प्रशिक्षित दाई (टीबीए)<br>दुकान<br>मित्र/माता-पिता/ रिश्तेदार<br>अन्य<br>पता नहीं<br>कोई जवाब नहीं
728b. आपने आखिर में क्या किया जिससे अंत में आपकी माहवारी वापस आ गयी?	((${self_reg_year} > ${self_abt_year}) or (${self_abt_year} = '')) and ((${self_reg_mult_yn} = 'ye …<br>ऑपरेशन प्रक्रिया जैसे की डी एन सी,सफाई<br>मिसोप्रोस्टोल या मीफीप्रिस्टोन नाम की गोलियों केद्वारा जैसे की 500/700 की गोली<br>गोलियाँ जो कि बुखार या मलेरिया के इलाज केलिए ली जाती हैं जैसे की पेरासिटामोल,कोम्बिफ्लेम या हरे पत्ते वाली दवाई<br>अन्य गोलियाँ<br>पारंपरिक विधियाँ जैसे जड़ी बूटीयाँ<br>घरेलू नुस्खे<br>योनी में सामग्री डालकर<br>अन्य<br>पता नहीं

	कोई जवाब नहीं
729b. आप उस प्रक्रिया केलिए कहाँ गयी?	((${self_reg_year} > ${self_abt_year}) or (${self_abt_year} = '')) and (${self_reg_last} = 'surger … सरकारी/ नगर पालिका अस्पताल<br>सरकारी औषधालय<br>यूएफडब्लूसी/यूएचसी/यूएचपी<br>सीएचसी / ग्रामीण अस्पताल /पी एच सी<br>उप-केन्द्र / एएनएम<br>सरकारी मोबाइल क्लिनिक<br>कैम्प<br>आंगनवाड़ी / आईसीडीएस केंद्र<br>आशा<br>अन्य समुदाय आधारित कार्यकर्ता<br>गैर सरकारी (NGO) संगठन या ट्रस्ट अस्पताल / क्लिनिक<br>निजी अस्पताल<br>निजी डाक्टर/क्लीनिक<br>निजी मोबाईल क्लीनिक<br>वैद्य/हकीम/होम्योपैथ चिकित्सक<br>पारंपरिक नीम हकीम<br>फार्मेसी / दवा की दुकान<br>प्रशिक्षित दाई (टीबीए)<br>दुकान<br>मित्र/माता-पिता/ रिश्तेदार<br>अन्य<br>पता नहीं<br>कोई जवाब नहीं
730b. आपको दवाईयाँ कहाँ से मिली?	((${self_reg_year} > ${self_abt_year}) or (${self_abt_year} = '')) and ((${self_reg_last} = 'pills … सरकारी/ नगर पालिका अस्पताल<br>सरकारी औषधालय<br>यूएफडब्लूसी/यूएचसी/यूएचपी<br>सीएचसी / ग्रामीण अस्पताल /पी एच सी<br>उप-केन्द्र / एएनएम<br>सरकारी मोबाइल क्लिनिक<br>कैम्प<br>आंगनवाड़ी / आईसीडीएस केंद्र<br>आशा<br>अन्य समुदाय आधारित कार्यकर्ता<br>गैर सरकारी (NGO) संगठन या ट्रस्ट अस्पताल / क्लिनिक<br>निजी अस्पताल<br>निजी डाक्टर/क्लीनिक<br>निजी मोबाईल क्लीनिक<br>वैद्य/हकीम/होम्योपैथ चिकित्सक<br>पारंपरिक नीम हकीम<br>फार्मेसी / दवा की दुकान<br>प्रशिक्षित दाई (टीबीए)<br>दुकान<br>मित्र/माता-पिता/ रिश्तेदार<br>अन्य<br>पता नहीं<br>कोई जवाब नहीं

731b. क्या आपको को अपनी माहवारी नियमित करने की प्रक्रिया में कोई समस्या हुई और किसी स्वास्थ्य केंद्र पर इलाज केलिए गयी?

यदि उत्तरदाता कहती है की वह अपनी माहवारी को नियमित करने केदौरान किसी स्वास्थ्य केंद्र में गयी थी, तो हम यह जानने में रूचि रखते हैं कि क्या वह किसी अन्य अवसर पर किसी समस्या केइलाज केलिए किसी स्वास्थ्य केंद्र पर गयी थी।

((${self_reg_year} > ${self_abt_year}) or (${self_abt_year} = '')) and ((${self_reg_yn} = 'yes'))

- हाँ
- नहीं
- पता नहीं
- कोई जवाब नहीं

732b. क्या आपने निम्न में से किसी व्यक्ति को इस अनुभव केबारे में बताया?

सभी विकल्पों को जोर से पढ़े तथा लागू होने वाले सभी विकल्पों का चयन करें

((${self_reg_year} > ${self_abt_year}) or (${self_abt_year} = '')) and ((${self_reg_yn} = 'yes'))

- पति/पुरुष साथी
- बहन
- भाई
- माँ
- पिता
- अन्य रिश्तेदार
- मित्र 1: ${friend1_name}
- मित्र 2: ${friend2_name}
- अन्य दोस्त
- अन्य
- पता नहीं
- कोई जवाब नहीं

(${friend1_name} != '' and ${friend1_name} != '-99' and filter_list = 'friend1') or (${friend2_name} != '' and ${friend2_name} != '-99' and filter_list = 'friend2') or (filter_list = 'always')

	${consent_obtained}
अब हम आपसे गर्भावस्था को हटाने केलिए कुछ सामान्य प्रश्न पूछेंगे जिसका जवाब आपको पुर्णतःसहमत,सहमत,नहीं सहमत नहीं असहमत,असहमत व पूर्णत असहमत में देना है<br>*सुनिश्चित करने केकेलिए इसमें निशान लगाये*	
जारी रखने केलिए OK दबाएँ	ओ.के

733. यदि गर्भवती होने से महिला केस्वास्थ्य को हानि पहुँच सकती है तो क्या ऐसे में महिला केलिए गर्भपात करवाना सही है।

${consent_obtained}

- पुर्णतः सहमत
- सहमत
- न सहमत न ही असहमत
- असहमत
- पुर्णतः असहमत
- कोई जवाब नहीं

734. यदि गर्भावस्था बलात्कार का परिणाम है तो क्या ऐसे में महिला केलिए गर्भपात करवाना सही है।

${consent_obtained}

- पुर्णतः सहमत
- सहमत
- न सहमत न ही असहमत
- असहमत
- पुर्णतः असहमत
- कोई जवाब नहीं

735. यदि महिला बच्चे का भरण-पोषण करने में असमर्थ है तो ऐसे में महिला केलिए क्या गर्भपात करवाना सही है।

${consent_obtained}

- पुर्णतः सहमत
- सहमत
- न सहमत न ही असहमत
- असहमत
- पुर्णतः असहमत
- कोई जवाब नहीं

736. यदि महिला बच्चा नहीं चाहती तो ऐसे में क्या महिला केलिए गर्भपात

${consent_obtained}

करवाना सही है।	पुर्णतः सहमत<br>सहमत<br>न सहमत न ही असहमत<br>असहमत<br>पुर्णतः असहमत<br>कोई जवाब नहीं
737. यदि महिला गर्भपात करवाती है तो क्या वह उसकेपरिवार केलिए शर्म की बात है।	${consent_obtained}<br>पुर्णतः सहमत<br>सहमत<br>न सहमत न ही असहमत<br>असहमत<br>पुर्णतः असहमत<br>कोई जवाब नहीं
738. महिला यदि गर्भपात करवाती है तो क्या उसे यह बात किसी को नहीं बतानी चाहिए।	${consent_obtained}<br>पुर्णतः सहमत<br>सहमत<br>न सहमत न ही असहमत<br>असहमत<br>पुर्णतः असहमत<br>कोई जवाब नहीं
739. क्या भारत में गर्भ समापन किन्ही परिस्थितियों में क़ानूनी रूप से मान्य है?	${consent_obtained}<br>हाँ<br>नहीं<br>पता नहीं<br>कोई जवाब नहीं

अनुवर्तन सहमती

FLW_801. अपना समय देने केलिए आपका बहुत धन्यवाद।<br>हम आपकेगर्भपात सम्बन्धी अनुभव जानने में रूचि रखते हैं जिसमे विधि चुनने और विधि लेने केलिए कहाँ जाना है तय करने में आपका निर्णय लेना इत्यादि शामिल है। क्या आप इस परियोजना में कार्यरत किसी अन्य महिला से बाद में अपने अनुभवों केबारे में बात करना चाहेंगीं?	(${self_abt_yn} = 'yes') or (${self_reg_yn} = 'yes')<br>हाँ<br>नहीं<br>कोई जवाब नहीं
FLW_802. क्या आपकेपास फ़ोन है?	${flw_willing} = 'yes'<br>हाँ<br>नहीं<br>कोई जवाब नहीं
FLW_803. क्या मैं आपका मुख्य नम्बर ले सकती हूँ?<br>*10 अंको का नम्बर दर्ज करें "कोई जवाब नहीं" केलिए 0 दर्ज करें*	${flw_number_yn} = 'yes'<br>--------------------------------------
FLW_804. क्या आप नम्बर एक बार पुन:दोहरा सकती है?<br>*10 अंको का नम्बर दर्ज करें "कोई जवाब नहीं" केलिए 0 दर्ज करें*	(${flw_number_typed} != '') and (${flw_number_typed} != '0')<br>--------------------------------------
उत्तरदाता को उनकेद्वारा दिये गये समय केलिए धन्यवाद दें।<br>*आपको घर केबाहर भी कुछ सवाल पूरे करने हैं*	${available} = 'yes'

स्थान और प्रश्नावली परिणाम

095. स्थान<br>*घर केप्रवेश द्वार केपास एक जी.पी.एस बिन्दु लें या स्थान रिकार्ड करें जब एक्यूरेसी 6 मीटर से कम हो।*	
096. कितनी बार आपने इस महिला उत्तरदाता केसाक्षात्कार केलिए इस घर का दौरा किया?	not(${unlinked}) or ${proceed_with_unlinked}<br>पहली बार<br>दूसरी बार<br>तीसरी बार
097. साक्षात्कार किस भाषा में किया गया है?	${consent_obtained}

- अंग्रेजी
- हिंदी
- अन्य

099. प्रश्नावली परिणाम
प्रश्नावली का परिणाम दर्ज करें

- पूरा हुआ
- घर पर नहीं
- स्थगित
- इनकार किया
- आंशिक रूप से पूरा
- अक्षम
